# Homoeologous exchange is a major cause of gene presence/absence variation in the amphidiploid *Brassica napus*

**DOI:** 10.1111/pbi.12867

**Published:** 2018-01-10

**Authors:** Bhavna Hurgobin, Agnieszka A. Golicz, Philipp E. Bayer, Chon‐Kit Kenneth Chan, Soodeh Tirnaz, Aria Dolatabadian, Sarah V. Schiessl, Birgit Samans, Juan D. Montenegro, Isobel A. P. Parkin, J. Chris Pires, Boulos Chalhoub, Graham J. King, Rod Snowdon, Jacqueline Batley, David Edwards

**Affiliations:** ^1^ School of Biological Sciences and Institute of Agriculture The University of Western Australia Crawley WA Australia; ^2^ School of Agriculture and Food Sciences University of Queensland St. Lucia QLD Australia; ^3^ Plant Molecular Biology and Biotechnology Laboratory Faculty of Veterinary and Agricultural Sciences University of Melbourne Melbourne VIC Australia; ^4^ Department of Plant Breeding IFZ Research Centre for Biosystems, Land Use and Nutrition Justus Liebig University Giessen Germany; ^5^ Agriculture and Agri‐Food Canada Saskatoon SK Canada; ^6^ Division of Biological Sciences University of Missouri Columbia MO USA; ^7^ Institute of System and Synthetic Biology, Organization and Evolution of Complex Genomes Institut National de la Recherche agronomique, Genopole Centre National de la Recherche Scientifique Université d'Evry Val d'Essonne Université Paris‐Saclay Evry France; ^8^ Southern Cross Plant Science Southern Cross University Lismore NSW Australia

**Keywords:** *Brassica napus*, canola, pangenome, recombination, genome structure

## Abstract

Homoeologous exchanges (HEs) have been shown to generate novel gene combinations and phenotypes in a range of polyploid species. Gene presence/absence variation (PAV) is also a major contributor to genetic diversity. In this study, we show that there is an association between these two events, particularly in recent *Brassica napus* synthetic accessions, and that these represent a novel source of genetic diversity, which can be captured for the improvement of this important crop species. By assembling the pangenome of *B. napus,* we show that 38% of the genes display PAV behaviour, with some of these variable genes predicted to be involved in important agronomic traits including flowering time, disease resistance, acyl lipid metabolism and glucosinolate metabolism. This study is a first and provides a detailed characterization of the association between HEs and PAVs in *B. napus* at the pangenome level.

## Introduction

*Brassica napus* is a recent allotetraploid species that was formed as a result of spontaneous interspecific hybridization between *Brassica oleracea* and *Brassica rapa* (Nagaharu, 1935). It exists primarily as an oilseed crop, but fodder types and vegetable forms (swedes and kale) are also grown. The *B. napus* gene pool includes synthetic lines, which are produced from interspecific crossing between *B. rapa* and *B. oleracea* (Gaeta *et al*., 2007), and is a source of novel genetic diversity (Cheung *et al*., 2009; Gaeta *et al*., 2007; Osborn *et al*., 2003; Sharpe *et al*., 1995), which is valuable for crop breeding. The meiotic chromosome pairing that occurs between homoeologous chromosomes which share a high degree of sequence identity leads to increased homoeologous exchanges (HEs) and gene conversion events (Gaeta and Chris Pires, 2010; Stein *et al*., 2017), and synthetic *B. napus* has been shown to exhibit a higher frequency of HEs than nonsynthetic *B. napus* (Liu *et al*., 2014; Rousseau‐Gueutin *et al*., 2017; Sharpe *et al*., 1995), making it an interesting model to study the impact of polyploidization on genome structure (Chalhoub *et al*., 2014; Clarke *et al*., 2016; Gaeta *et al*., 2007; Nicolas *et al*., 2012; Osborn *et al*., 2003; Parkin *et al*., 1995; Schmutzer *et al*., 2015; Stein *et al*., 2017; Szadkowski *et al*., 2011; Udall *et al*., 2005) and agronomic traits (Rousseau‐Gueutin *et al*., 2017; Schiessl *et al*., 2017a; Stein *et al*., 2017; Zou *et al*., 2011).

Two public reference genomes corresponding to the winter oilseed cultivars Darmor‐*bzh* (Bayer *et al*., 2017; Chalhoub *et al*., 2014) and Tapidor (Bayer *et al*., 2017) are currently available; however, it was unknown how well these references represent the genetic diversity found in *B. napus*. The pangenome represents the set of genes for a species, composed of core genes, which are present in all individuals, and variable genes, which are only present in some individuals. The concept of the pangenome was introduced by Tettelin *et al*. (2005), who produced the first pangenome for the bacterial species *Streptococcus agalactiae*. However, pangenomics is increasingly being applied to higher organisms, including maize (Hirsch *et al*., 2014), soybean (Li *et al*., 2014), wheat (Montenegro *et al*., 2017), *B. rapa* (Lin *et al*., 2014), *B. oleracea* (Golicz *et al*., 2016), rice (Schatz *et al*., 2014) and *Medicago truncatula* (Zhou *et al*., 2017).

In this study, we analyse data from a collection of 53 synthetic and nonsynthetic accessions (Schmutzer *et al*., 2015; Snowdon *et al*., 2015) to produce the first estimate of the *B. napus* pangenome, and investigate the role of HEs in *B. napus* genomic diversity. These accessions come from diverse geographical locations, comprising a range of morphotypes including oilseeds, fodder and vegetable types. We identify core and variable genes and predict the size of the pangenome and core genome for this species. We also assess the variable gene content in relation to HEs and their potential association with agronomic traits. Our results highlight the potential of using resynthesized *B. napus* accessions as a source of novel genetic structural variation for breeding improved varieties.

## Results

### Assembly and annotation of the *B. napus* pangenome

The *B. napus* pangenome was constructed using 33 nonsynthetic accessions and 20 synthetic accessions using a mapping and assembly approach previously applied for *B. oleracea* (Golicz *et al*., 2016) and bread wheat (Montenegro *et al*., 2017). An improved version (v. 8.1) of the public Darmor‐*bzh* genome assembly (Bayer *et al*., 2017) was used as the starting reference. The resulting pangenome size was 1044 Mbp (Table S1) and contained 94 013 predicted genes (Table S2), compared with 850 Mbp and 80 382 genes in the Darmor‐*bzh* v8.1 assembly. Validation of the assembly identified more than 97% complete Benchmarking Universal Single‐Copy Orthologs (BUSCOs) (Simao *et al*., 2015) suggesting a high level of completeness (Table S3).

### Properties of the core and variable genome

**Figure 1:**
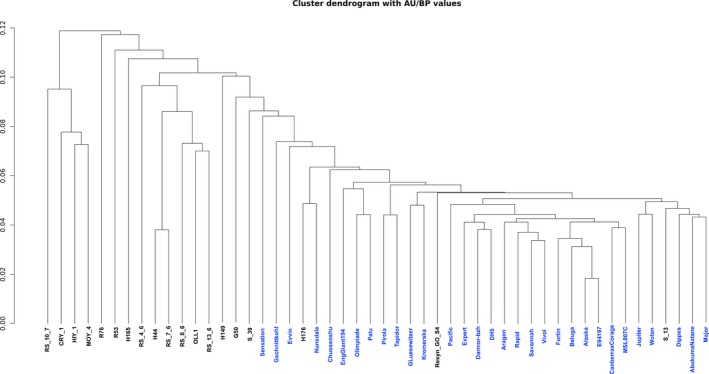
Dendrogram depicting the relationship between the accessions based on gene PAV results. The accessions R99, Start and Skziverskij are excluded due to low read mapping coverage. Synthetic accessions are shown in black while nonsynthetics are shown in blue.

**Figure 2:**
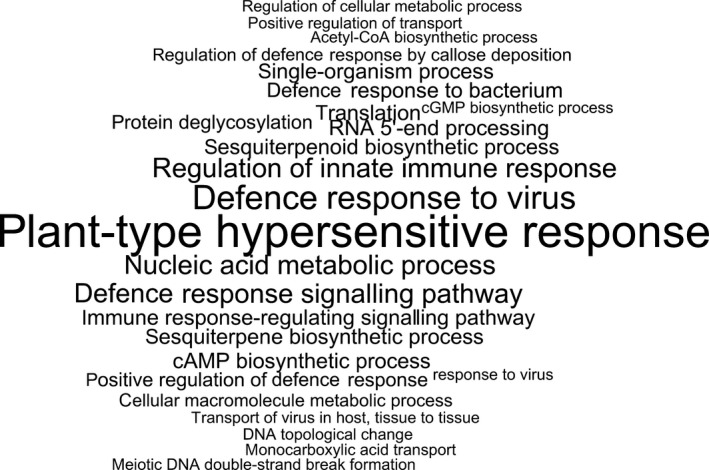
GO enrichment analysis of variable genes. Significantly enriched GO terms among variable genes using all pangenome genes as background. Font size is proportional to –log (*P*).

**Figure 3:**
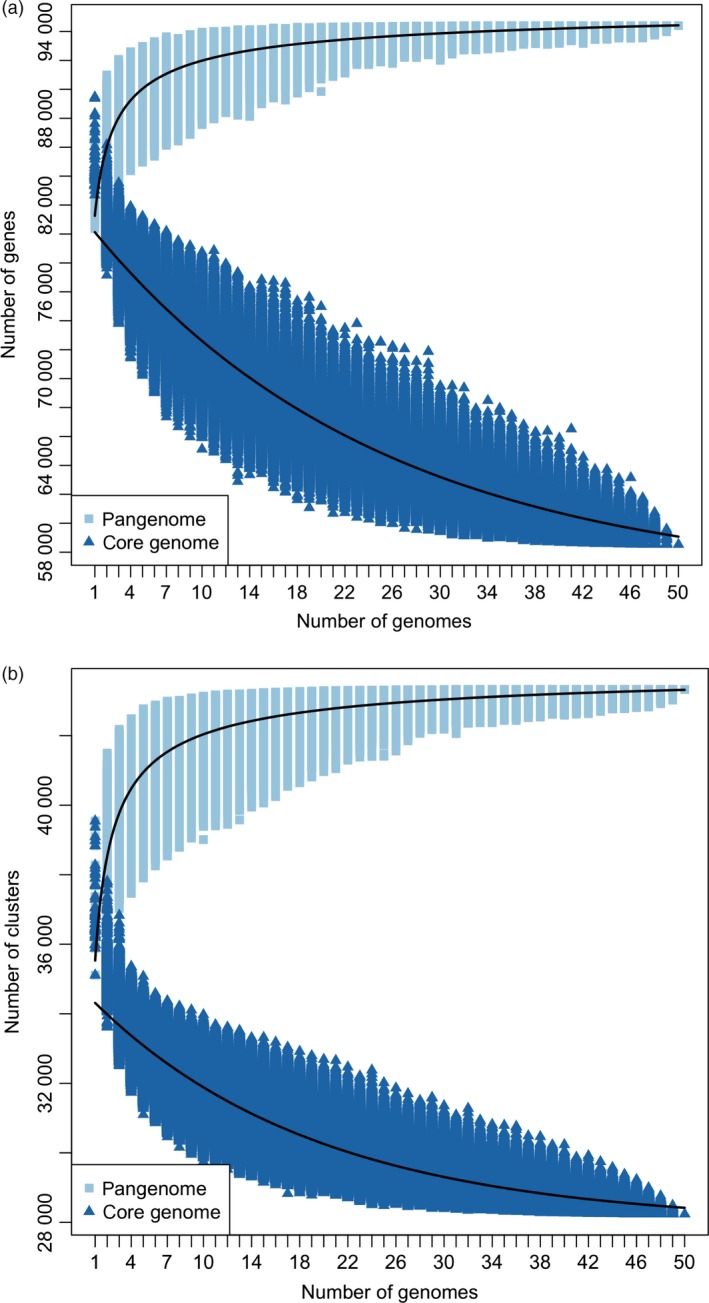
Pangenome modelling. Model describing the size of core and pangenome with every genome added using all (a) genes and (b) orthologous gene clusters. The calculated values depend on the number of genomes used in the analysis. The combinations of genomes were obtained according to the following formula: 50!/(*n*!(50−*n*)!), *n* = [1,50]. Pangenome curve: *y* = A*x* ^B^ + C. Core genome curve: *y* = Ae^B*x*^ + C.

The majority of the pangenome is composed of core genes (62%, 58 532), while the remaining (38%, 35 481) are variable (Table S4). Variable genes are shorter than core genes, with fewer exons per gene, a similar finding to pangenome studies in other species (Bush *et al*., 2014; Schatz *et al*., 2014). A total of 43 327 orthologous gene clusters were identified, of which 28 239 (65.2%) are core and 15 088 (34.8%) are variable. The synthetic accessions demonstrate the greatest presence/absence variation (Figure 1), with an average of 22 uniquely present and 435 uniquely absent genes (Table S5). Functional and GO enrichment analysis reveals that the variable genome is enriched with genes predicted to be involved in disease resistance (Figure 2; Tables S6, S7 and S8). Modelling of the pangenome and core genome suggests a closed (restricted) pangenome with a finite number of genes and gene clusters, consistent with pangenome analyses in other plant species including maize (Hirsch *et al*., 2014) and soybean (Li *et al*., 2014). The size of the pangenome expanded with each additional accession to a total of 94 013 genes (43 327 gene clusters) (Figure 3a). Modelling this growth predicts a total pangenome size of 95 730 ± 11 genes (44 050 ± 2 gene clusters), while the core genome decreases from 58 532 genes (28 239 gene clusters), with a predicted core genome size of 55 850 ± 21 genes (27 960 ± 1 gene clusters) (Figure 3b). A total of 4 875 729 SNPs were identified in the pangenome, with a SNP density of 4.65 SNPs/Kbp (Tables S9 and S10), and 14.6% of these SNPs were identified in the newly assembled contigs providing a novel source of molecular markers for genetic analysis. Core genes had a higher proportion of synonymous SNPs and a lower proportion of nonsynonymous and non‐sense SNPs compared to variable genes (Table S11). These results are similar to those obtained in a pangenome study of wild soybean accessions (Li *et al*., 2014) and reflect the reduced conservation of variable genes.

### Association of gene presence/absence variation with homoeologous exchanges

**Figure 4:**
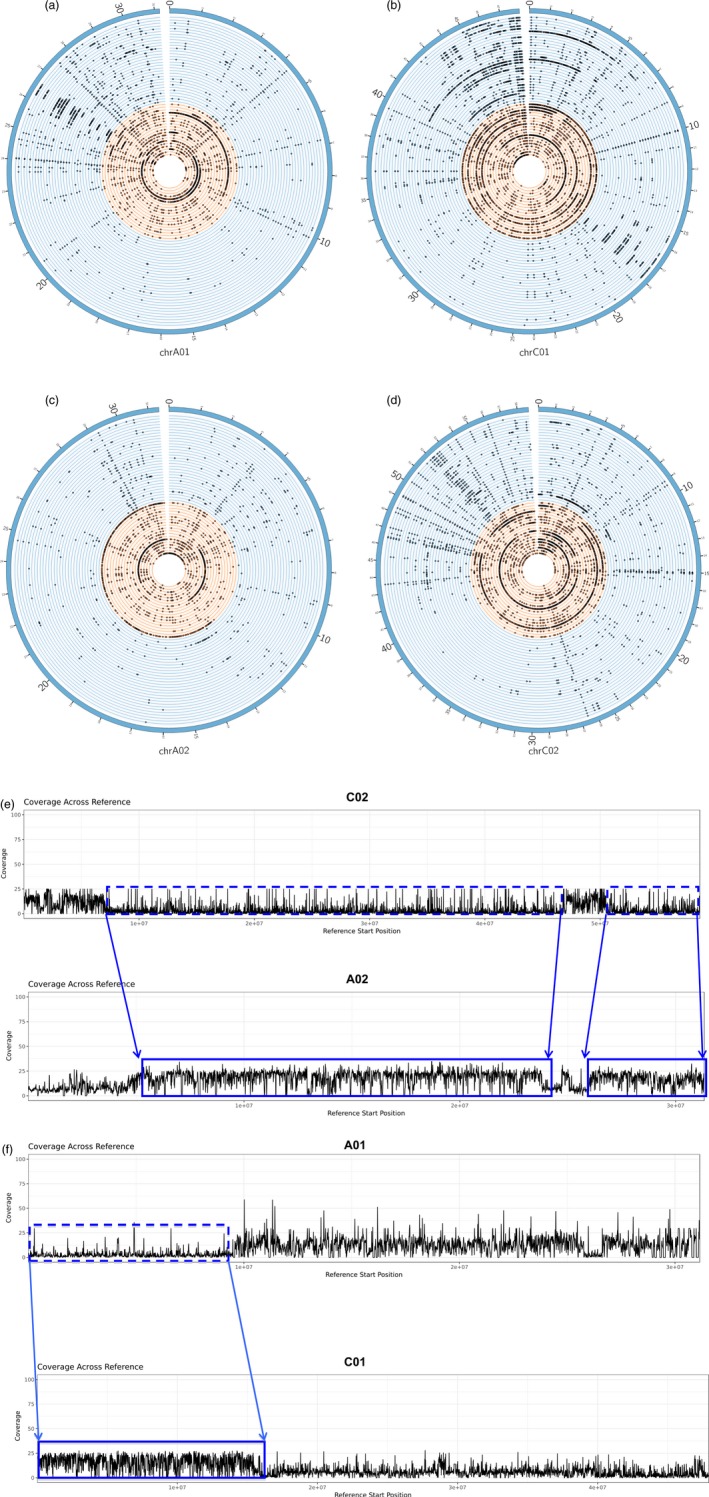
Association between gene PAV with HEs with respect to the synthetic accession H165. Circos plots showing patterns of gene loss on pairs of homoeologous chromosomes (a,b) A01‐C01 and (c,d) A02‐C02. Nonsynthetic accessions are shown as blue circular lines while the synthetic accessions are shown as orange circular lines. The dots on the circular lines denote the genes which were identified as lost in the synthetic and nonsynthetic accessions. H165 appears as accession number 35 on the plot (orange section). Regions containing genes, which appear to be lost (box with blue dashed line) but have corresponding homoeologous duplicated copies (box with solid blue line) are shown. The blue arrows delimit the approximate locations of the HEs in the genome, and the direction of the arrows indicates the direction in which the HEs have occurred; HEs have occurred from (e) C02 to A02 and (f) A01 to C01. In all cases, a reduction in mean read mapping coverage was observed in regions where the genes were lost and a corresponding doubling of coverage was observed in regions where the duplicated homeolog copies of the lost genes were located. The read mapping coverage with respect to H165 is approximately 12×. Images are not drawn to scale.

Two types of gene PAV events were detected on the Darmor‐*bzh* portion of the pangenome: non‐HE gene PAVs, where individual genes are lost, and HE‐related gene PAVs, where groups of genes are lost through replacement of their corresponding genomic region by a homoeologous segment of the genome. All accessions used in this study displayed non‐HE PAVs, while 30 of the 53 accessions also exhibited HE‐related PAVs (Figure S1; Table S19; Data S1). The HE‐related PAVs were localized near the start and end of chromosomes and occurred more frequently and on a larger scale in the synthetics. The majority of HE events occurred on chromosomes C01, C02, C03, C08 and C09 and their homoeologous chromosomes, A01, A02, A03, A09 and A09. While the nonreciprocal exchange occurred mostly from the C genome to the A genome, several HEs were also observed in the opposite direction, and these occurred exclusively in the synthetics. An example of HE based gene loss is presented in Figure 4 for the synthetic accession H165. The extent of chromosome rearrangements in this accession illustrates the extent of genome‐scale variation in synthetic *B. napus*.

Functional annotation of the HE‐related PAV genes suggests their involvement in defence, stress and auxin pathways (Table S12), similar to observations of variable genes in other species, although their association with HEs has not been previously reported. HEs have been shown to impact agronomic traits (Rousseau‐Gueutin *et al*., 2017; Schiessl *et al*., 2017a,b; Stein *et al*., 2017; Szadkowski *et al*., 2010; Zou *et al*., 2011), including the flowering time regulators *PHYTOCHROME A (PHYA)*, gibberellin 3‐beta‐dioxygenase (*GA3ox1*) and *FLOWERING LOCUS C* (*FLC*) (Schiessl *et al*., 2017b). Here, we confirm that *PHYA* and *GA3ox1* are present on chromosome A09, but absent on the homoeologous portion of chromosome C08 in the majority of swede accessions from the ERANET‐ASSYST population as previously reported (Schiessl *et al*., 2017b), suggesting a potential split between swede and nonswede types (Data S2). The swede accessions share a HE event affecting one of the four homologous pairs of *FLC*, with the *FLC* gene on chromosome A10 duplicated in these lines, and the corresponding homologue on chromosome C09 is absent, as previously reported (Chalhoub *et al*., 2014; Schiessl *et al*., 2017a). Duplication of the *FLC* gene on A10 has been linked to the strong vernalization requirement of accessions, which have an active vernalization system in place (Schiessl *et al*., 2017a). One of the two swede accessions used for the pangenome construction (Sensation NZ) hosts this HE event while the other (Fortin Family) does not, highlighting the differences that may exist within morphotypes. The presence of such HEs, which lead to variation in *FLC* allele content, could be responsible for the climatic and geographical adaptation of *B. napus* after allopolyploidization.

### Gene presence/absence variation in relation to genes for agronomic traits

A total of 307 disease resistance genes (*R*‐genes) were identified, of which 94 are core (30.6%) and the remaining 213 are variable (69.4%). Almost half (146, 47.6%) are located in the newly assembled contigs, while the remaining 52.4% are in the Darmor‐*bzh* v8.1 reference assembly. The nonsynthetics have lost more disease resistance genes (average of 126) compared to the synthetics (average of 107) (Table S13). The majority of accessions used to build the pangenome were found to carry the susceptible allele *lepr3/rlm2* for blackleg infection, which is caused by the fungus *Leptosphaeria maculans* (Larkan *et al*., 2013, 2014, 2015), while the nonsynthetic accessions Tapidor and English Giant 194 and the synthetic Resyn‐Go S4 were found to carry the resistant allele (Figure S2). In contrast to the gene sequences, the region harbouring the resistant and susceptible alleles of this gene was identified as being conserved in all accessions (Figure S3). The genes BnaA03g43460.1D2 and BnaA08g08960.1D2 were identified as potential orthologues of the *B. rapa* clubroot genes, *CRa* and *crr1a* (Hatakeyama *et al*., 2013; Suwabe *et al*., 2012; Ueno *et al*., 2012), respectively. Gene PAV and read mapping coverage analysis suggest that while BnaA03g43460.1D2 is variable (Figure S4), BnaA08g08960.1D2 is found in all accessions (Figure S5). Defence response genes have previously been shown to demonstrate PAV in several plant species (Gonzalez *et al*., 2013; Li *et al*., 2014; Zhou *et al*., 2017). It has been suggested that defence response genes may have been deleted in the Brassica genome following whole genome triplication, as multiple copies of these genes may be disadvantageous (Liu *et al*., 2014; Lysak *et al*., 2006, 2005). In contrast, strong selection pressure in the presence of pathogens may lead to retention and conservation of some gene copies.

Genes involved in acyl lipid and GSL metabolism also displayed PAV behaviour. A total of 1466 core and 427 variable acyl lipid metabolism genes were identified (Table S14) while a total of 227 GSL biosynthesis genes (180 core and 47 variable) and 96 breakdown genes (79 core and 17 variable) were found (Table S15). When considering the unique instance of each acyl lipid and GSL metabolism gene, the synthetics had lost more (388 acyl lipid genes and 46 GSL metabolism genes) compared to the nonsynthetics (181 acyl lipid genes and 21 GSL metabolism genes). Gene PAV with respect to GSL metabolism has previously been linked to HE events (Chalhoub *et al*., 2014), and such variation at the gene level can lead to phenotypic variation for traits under natural and artificial selection, resulting in the selection of HEs contributing beneficial gene variants.

## Discussion

Synthetic *B. napus* has previously been shown to demonstrate greater genetic diversity than nonsynthetic accessions (Golicz *et al*., 2016; Li *et al*., 2014), and this difference has been attributed to the incorporation of novel alleles from diverse progenitor genomes. In this study, we show that this diversity is amplified by PAV, with many of the variable genes due to HE events in the new synthetic accessions. These HE‐related PAV events are useful to understand the association between genome structural rearrangement and phenotypic variation, particularly the role of genome duplications or deletions spanning genes with trait‐related dosage effects. The observation that synthetic accessions experience HEs on a larger scale and more frequently than their nonsynthetic counterparts suggests that they have the potential to increase the genetic diversity of *B. napus* accessions producing novel allele combinations and associated phenotypic variation beyond the addition of novel allelic variants (Chalhoub *et al*., 2014; Rousseau‐Gueutin *et al*., 2017; Sharpe and Lydiate, 2003). This study also highlights the potential of synthetic accessions to understand the basis of recombination frequency and genomic variation changes in polyploids in general, a process that may be important for heterosis, niche exploitation and speciation (Gaeta and Chris Pires, 2010).

The abundant PAV of *R*‐genes in the synthetic accessions highlights their potential for the introgression of candidate disease resistance genes in *B. napus*, supporting adaptation of this important crop to diverse environments and pests. This study also illustrates the value of the pangenome in capturing additional information not contained within a single reference, with almost half of the candidate *R*‐genes identified being in the variable genome. This information can be exploited to further characterize the relationships between candidate *R*‐genes and resistance/susceptibility among accessions.

## Methods

### Pangenome assembly

The *B. napus* pangenome was constructed using a mapping and assembly approach. This consisted of three main steps: mapping genomic sequence reads to the reference sequence, assembly of the unmapped reads and the production of a new reference by adding the newly assembled contigs to the reference. Mapping of the reads was performed using Bowtie2 (Langmead and Salzberg, 2012) v2.2.6 (–end‐to‐end –sensitive) against the Darmor‐*bzh* v8.1 reference genome assembly and assembly was performed using MaSuRCA (Zimin *et al*., 2013) v3.1.3 with default parameters other than (cgwErrorRate = 0.15 (within the recommended range 0.10–0.15); ovlMemory = 8GB). Detailed information on the reads used for mapping and assembly can be found in Table S16. Benchmarking Universal Single‐Copy Orthologs (BUSCO) was used to evaluate the completeness of the pangenome using default parameters. Additionally, a series of validation steps were performed to assess the quality of the assembly (Figures S6–S8).

### Removal of contaminants

The newly assembled contigs were compared with NCBI nt database (01/08/2016) (blastn ‐task dc‐megablast ‐template_length 18 ‐template_type coding_and_optimal ‐max_target_seqs 2 ‐*e*‐value 1e−5) using BLAST+ (Camacho *et al*., 2009) v2.2.31. Contigs, which had best hits (pid > 90 and (alignment length/query length) ≥ 50) against nongreen plants, mitochondrial and chloroplast sequences were considered to be contamination and excluded from further analysis. The assembled contigs were compared with a collection of Illumina adapters obtained from the adapter database distributed with Trimmomatic, and the adapter database obtained from https://github.com/csf-ngs/fastqc/blob/master/Contaminants/contaminant_list.txt. Adapters at the end of contigs were trimmed while those within contigs were masked.

### Annotation of the pangenome

Newly assembled contigs >1000 bp were annotated using MAKER2 (Holt and Yandell, 2011) v2.31.3. The transposable element file within MAKER ‘te_proteins.fasta’ was used for repeat masking. *De novo* gene predictions were based on AUGUSTUS (Stanke *et al*., 2004) and SNAP (Korf, 2004). EST evidence was based on the 95k ESTs (http://brassica.nbi.ac.uk/array_info.html), and *B. rapa*,* B. oleracea* and *B. napus* unigenes downloaded from UniGene (ftp://ftp.ncbi.nlm.nih.gov/repository/UniGene). Protein evidence was based on Brassicaceae proteins downloaded from RefSeq. Publicly available RNA‐Seq data (SRA accession numbers PRJEB5461 and ERA036824) were included as additional evidence. Annotated genes with an AED score of 1, genes carrying transposase‐related (TE) domains identified by InterProScan (Jones *et al*., 2014) version 5.20‐59.0 and genes shorter than 100 bp in nucleotide space were removed from subsequent analysis. A total of 137 TE domains were used (Table S17).

### Gene presence/absence variation

Gene PAV discovery was performed using the SGSGeneLoss package (Golicz *et al*., 2015) (lostCutoff = 0.05 and minCov = 2). Reads were mapped to the pangenome using Bowtie2 (–end‐to‐end –sensitive), and reads mapping in proper pairs were retained using SAMtools (Li *et al*., 2009) v 1.2 (samtools view ‐bS ‐f 2). Gene PAV was also determined in the 280 accessions from the ERANET‐ASSYST *B. napus* diversity set. The analysis performed on the ERANET‐ASSYST diversity set in this pangenome study is distinct from the analysis performed by Schiessl *et al*. (2017b). Different thresholds (minCov = 300 and lostCutoff = 0.70) were applied due to the high read depth coverage of the accessions in the diversity set. To estimate the relationship between the accessions used in the pangenome study, a dendrogram was constructed based on the gene presence/absence results, which were converted into a binary matrix (‘1’ representing present genes and ‘0’ representing absent genes). The R package PVClust was used to infer phylogeny. 1000 resamplings were used for bootstrap and *P*‐values calculations.

### Identification of homoeologous exchanges (HEs)

A combination of read mapping coverage and BLAST comparisons were used to detect HEs. The same mappings produced for the PAV gene detection step were used. The per base depth of coverage was calculated for each accession and for each chromosome stemming from the Darmor‐*bzh* portion of the assembly using BEDTools (Quinlan and Hall, 2010) v 2.25.0 (bedtools genomecov ‐d). A BLAST comparison (*e*‐value 1e−50) was performed on groups of consecutive genes, which appeared to be absent in a particular accession. If the genes had best hits located on the homoeologous chromosome (pid > 90% and alignment length of target gene = the alignment length of query gene ± 10 bp), the best hits were roughly arranged consecutively and were labelled as present by SGSGeneLoss, then these genes would be considered to be HE copies of the absent genes. A second check was performed by calculating the average depth of read coverage in bins of 1000 bp; if genes appeared to be lost and the coverage of mapped reads in the region encompassing the genes was zero or close to zero, but if the coverage was more than 1.5 times the average coverage in the region where the gene duplication occurred, that is in the HE region, then the genes would be considered to have undergone an HE event.

### Gene ontology enrichment analysis

Functional annotation of the pangenome was carried out using Blast2GO (Conesa *et al*., 2005) command line v2.5. The pangenome genes were compared with the proteins in the Viridiplantae database and preformatted to comply with Blast2GO naming requirements. Comparisons were made using BLAST. Gene ontology (GO) enrichment analysis of the variable genes (absent and present) was performed in R using the topGO package (Alexa and Rahnenfuhrer, 2010) using Fisher's exact test with method ‘elim’ used to adjust for multiple comparisons.

### Gene clustering

Genes were clustered using OrthoMCL (Fischer *et al*., 2011) v2.09 (default parameters). The pangenome genes were clustered with *Arabidopsis thaliana* genes (TAIR 10) (http://www.arabidopsis.org/portals/genAnnotation), and gene families were divided into core and variable. A gene family was considered to be core if at least one gene in the family was present in all the accessions. The gene family was considered variable if the whole gene family was missing from at least one accession.

### Pangenome modelling

Curves corresponding to pangenome size and core genome size with respect to genes and gene families were fitted in R (R Development Core Team, 2011) using the nlsLM function (nonlinear least squares based on a modified version of the Levenberg–Marquardt algorithm) from the package minpack.lm. The combinations of genomes were obtained according to the following formula: 50!/(*n*!(50−*n*)!), *n* = [1,50]. The pangenome size was modelled using the power law regression: *y* = A*x* ^B^ + C (Tettelin *et al*., 2005; Zhao *et al*., 2014) while the core genome size was modelled using the exponential regression *y* = Ae^B*x*^ + C. Due to the large number of genomes used in this study, an alternative to calculating all possible combinations of genomes was used; in this case, only 100 000 combinations of genomes (where possible) were calculated for each genome. The model was fitted using all 100 000 points for each genome.

### Identification of candidate *R*‐genes

*R‐*genes in the pangenome were identified using InterProScan based on the domains from Pfam and Coils databases. The domain names are listed in Table S18. All possible amino acid sequences of *LepR3, Rlm2*,* lepr3/rlm2 CRa* and *crr1a* were downloaded from NCBI. A BLAST search (blastp ‐*e*‐value 1e−50) was first performed on the protein sequences of these genes against the pangenome proteins. In the case of *CRa* and *crr1a* where the query protein sequences were only partially covered by their candidate *B. napus* orthologs, a BLAST search (tblastn ‐*e*‐value 1e−50 and pid > 90%) was performed on the genes against the pangenome assembly to identify the target regions while making sure that the candidate orthologs were present in those regions. Similarly, to delimit the exact location of *lepr3/rlm2* in the pangenome, a BLAST search was performed on the *B. napus* homologue of *lepr3* (BnaA10g20720D) from the Darmor‐*bzh* v4.0 reference genome assembly against the pangenome (blastp ‐*e*‐value 1e−50 and pid > 90%). To identify whether the accessions used to build the pangenome carried the susceptible or resistant alleles of the blackleg genes, the read mappings from the SNP discovery step were used. For each accession, the nucleotide sequence corresponding to the candidate region was extracted from the BAM file using SAMtools (samtools mpileup ‐uAIf) and BCFtools (Li *et al*., 2009) v 1.2 (bcftools call ‐m ‐o). To obtain the protein sequence corresponding to the extracted region, TransDecoder v 3.0 (https://transdecoder.github.io/) was used to predict the longest open‐reading frame (ORF). Multiple sequence alignments were performed on the predicted protein sequences from all the accessions using MUSCLE (Edgar, 2004), and the alignments were visualized in Jalview (Waterhouse *et al*., 2009).

### Targeted gene family analysis

Genes for lipid biosynthesis genes, GSL metabolism and *FLC* genes were retrieved from the Darmor‐*bzh* v4.0 reference genome. Their *A. thaliana* orthologs were obtained from the *A. thaliana* metabolic pathway database (ftp://ftp.plantcyc.org/Pathways/Data_dumps/PMN11_June2016/aracyc_pathways.20160601, version downloaded on 28.07.2016). A BLAST comparison (*e*‐value 1e−5) was performed on the Darmor‐*bzh* genes with the *B. napus* pangenome, and the best hits were selected for targeted gene families analysed. The *A. thaliana* orthologs were compared with the orthologous gene clusters, and the pangenome target genes were identified as follows: if a cluster contained *A. thaliana* and *B. napus* genes, and the *A. thaliana* genes belonged to a pathway, then all the associated *B. napus* genes were assigned to that pathway. In addition, if a *B. napus* gene's best *A. thaliana* BLAST hit was directly involved in a pathway, then the *B. napus* gene was considered to be involved in that pathway as well.

### SNP discovery and annotation

Mappings used for gene PAV discovery were also used for SNP discovery. Additionally, duplicates were marked using Picard tools (https://sourceforge.net/projects/picard) v2.2.1 MarkDuplicates. SNPs were discovered using BCFtools ‘call’ command (‐v ‐m) using the output from SAMtools mpileup (‐q 30 ‐Q 20 ‐g ‐I). The resulting files were filtered using vcflib (https://github.com/vcflib/vcflib) (vcffilter ‐f ‘DP > 10 & QUAL > 30’). Heterozygous SNPs were considered potential artefacts and removed. SNPs were categorized as coding, synonymous, nonsynonymous and non‐sense using the R package VariantAnnotation (Obenchain *et al*., 2014) v 1.13.46. Only genes with at least one coding SNP were considered in the analysis.

## Data availability

All the data used in this study are available on SRA through accessions numbers PRJEB5974, PRJEB6069, PRJNA342383, PRJEB5841, ERA036824 and SRP087610. All results stemming from this study are available at http://brassicagenome.net/. All other data supporting the findings of this study are available in this article and its Supplementary Information or available from the corresponding author on request.

## Authors' contributions

BH, DE and JB conceived this study. BH designed the experiments, performed the experiments and wrote the manuscript. AG developed the SGSGeneLoss package and assisted with gene PAV analysis. PB produced the Darmor v8.1 assembly and assisted with the pangenome assembly. CKC helped with code debugging and created a genome browser to visualize the results of this study. ST and AD assisted with the *R*‐genes analysis. SVS and RS provided sequence capture data for 280 accessions of the ERANET‐ASSYST population with respect to the flowering time regulatory genes mentioned in this manuscript. JDM assisted with pangenome modelling. DE and JB helped design and provided critical revisions of the manuscript. BS, IAPP, JCP, BC, GJK and RS provided critical revisions of the manuscript. All authors read and approved the final manuscript.

## Competing financial interests

The authors declare no competing financial interests.

## Supporting information


**Figure S1** Circos plots showing patterns of lost genes on the Darmor‐*bzh* portion of the pangenome on the A genome (a–j), the C genome (k–s) and unplaced contigs (t).
**Figure S2** Phylogenetic relationship of the allelic variants of the blackleg gene.
**Figure S3** Read mapping coverage in region harbouring *lepR3/rlm2* on chromosome A10 of the pangenome for the non‐synthetics (a and b) and synthetics (c).
**Figure S4** Read mapping coverage in the region harbouring BnaA03 g43460.1D2, which was identified as the potential candidate ortholog of the *B. rapa* gene, *CRa* on chromosome A03 of the pangenome in (a and b) non‐synthetics and (c) synthetics.
**Figure S5** Read mapping coverage in the region harbouring BnA08 g08960.1D2, which was identified as the potential candidate ortholog of the *B. rapa* gene, *crr1a* on chromosome A08 of the pangenome in (a and b) non‐synthetics and (c) synthetics.
**Figure S6** For each of the accessions, the percentage of reads mapping to the Darmor‐*bzh* v8.1 portion of the pangenome only and the entire pangenome was calculated.
**Figure S7** Summary of coverage statistics when mapping reads from a given accession to the contigs contributed by this accession to the pangenome during iterative mapping and assembly.
**Figure S8** The fraction of the pangenome which had reads mapping (coverage ≥ 1) for each of the accessions.

**Table S1** Assembly statistics.
**Table S2** Number of genes annotated and used in the analysis.
**Table S3** BUSCO results to assess the completeness of the pangenome assembly.
**Table S4** Gene PAV with respect to the synthetic and non‐synthetic accessions.
**Table S5** Number of uniquely present and absent genes in (a) non‐synthetics and (b) synthetics.
**Table S6.** Gene ontology (GO) enrichment of uniquely present genes.
**Table S7** GO enrichment of uniquely absent genes.
**Table S8** GO enrichment of variable genes for the biological process category.
**Table S9** Number of SNPs and SNP density across the pangenome.
**Table S10** Number of private SNPs in (a) non‐synthetics and (b) synthetics.
**Table S11** Properties of CDS SNPs in core and variable genomes.
**Table S12** GO enrichment of HE PAV genes.
**Table S13** Number of *R‐*genes absent in (a) non‐synthetics and (b) synthetics.
**Table S14** Number of acyl lipid metabolism genes absent (a) non‐synthetics and (b) synthetics.
**Table S15** Number of GSL metabolism genes absent in (a) non‐synthetics and (b) synthetics.
**Table S16** Reads used for pangenome assembly.
**Table S17** Pfam domains used to identify TE genes.
**Table S18** Domains used to identify *R*‐genes. The Coils database was used to identify CC domains.
**Table S19** Order of accession on Circos plot for (a) non‐synthetics and (b) synthetics.

**Data S1** Results of the HE analysis across the Darmor‐*bzh* portion of the pangenome.

**Data S2** Gene PAV of the flowering time regulators *FLC*,* PHYA* and *GA3ox1* in the ERANET‐ASSYST *B. napus* diversity set. Swede lines are shown in red.
