# Supplementary material for: Homoeologous exchange is a major cause of gene presence/absence variation in the amphidiploid Brassica napus
Source: Plant Biotechnol J. 2018 Jan 10;16(7):1265–74. doi: 10.1111/pbi.12867 (PMC5999312; doi:10.1111/pbi.12867)
Supplement: Supplementary file 1 — Figure S1 Circos plots showing patterns of lost genes on the Darmor‐bzh portion of the pangenome on the A genome (a–j), the C genome (k–s) and unplaced contigs (t). Figure S2 Phylogenetic relationship of the allelic variants of the blackleg gene. Figure S3 Read mapping coverage in region harbouring lepR3/rlm2 on chromosome A10 of the pangenome for the non‐synthetics (a and b) and synthetics (c). Figure S4 Read mapping coverage in the region harbouring BnaA03 g43460.1D2, which was identified as the potential candidate ortholog of the B. rapa gene, CRa on chromosome A03 of the pangenome in (a and b) non‐synthetics and (c) synthetics. Figure S5 Read mapping coverage in the region harbouring BnA08 g08960.1D2, which was identified as the potential candidate ortholog of the B. rapa gene, crr1a on chromosome A08 of the pangenome in (a and b) non‐synthetics and (c) synthetics. Figure S6 For each of the accessions, the percentage of reads mapping to the Darmor‐bzh v8.1 portion of the pangenome only and the entire pangenome was calculated. Figure S7 Summary of coverage statistics when mapping reads from a given accession to the contigs contributed by this accession to the pangenome during iterative mapping and assembly. Figure S8 The fraction of the pangenome which had reads mapping (coverage ≥ 1) for each of the accessions. [file PBI-16-1265-s004.pdf]

a

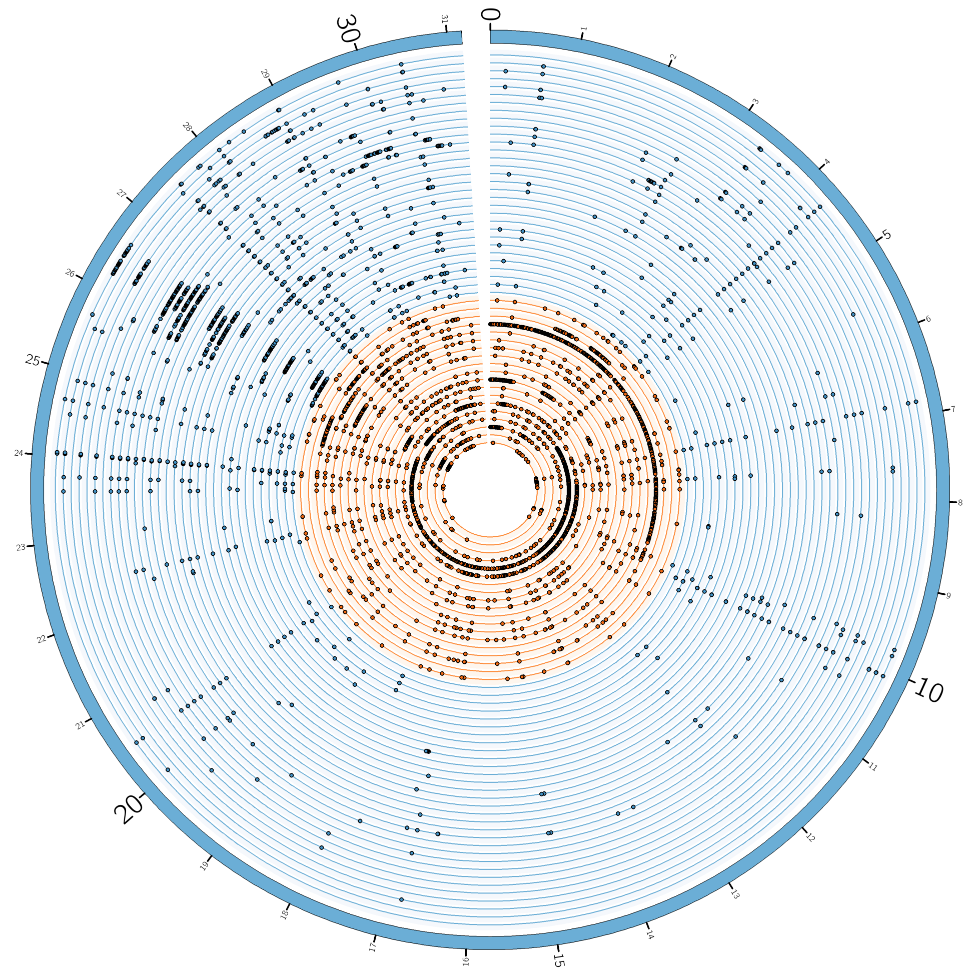

b

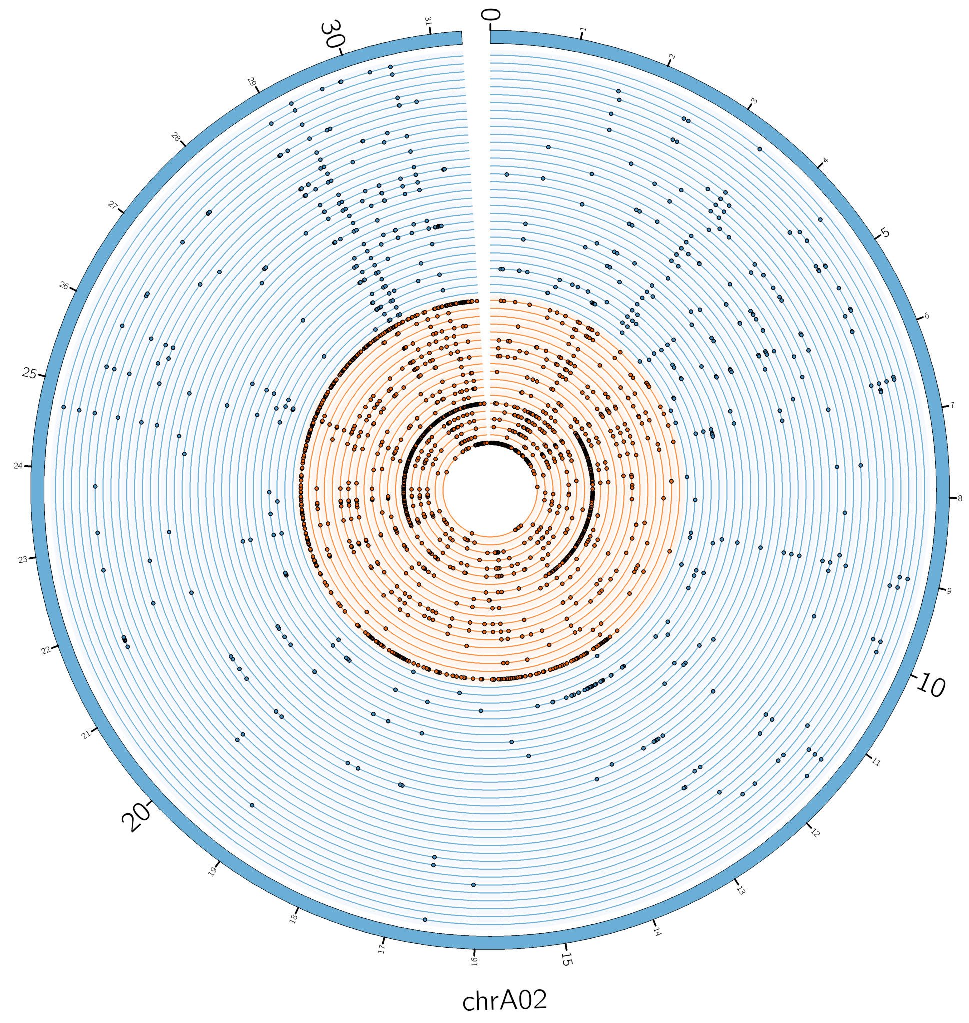

0
1
2
3
4
5
6
7
8
9
10
11
12
13
14
15
16
17
18
19
20
21
22
23
24
25
26
27
28
29
30
31
chrA02

c

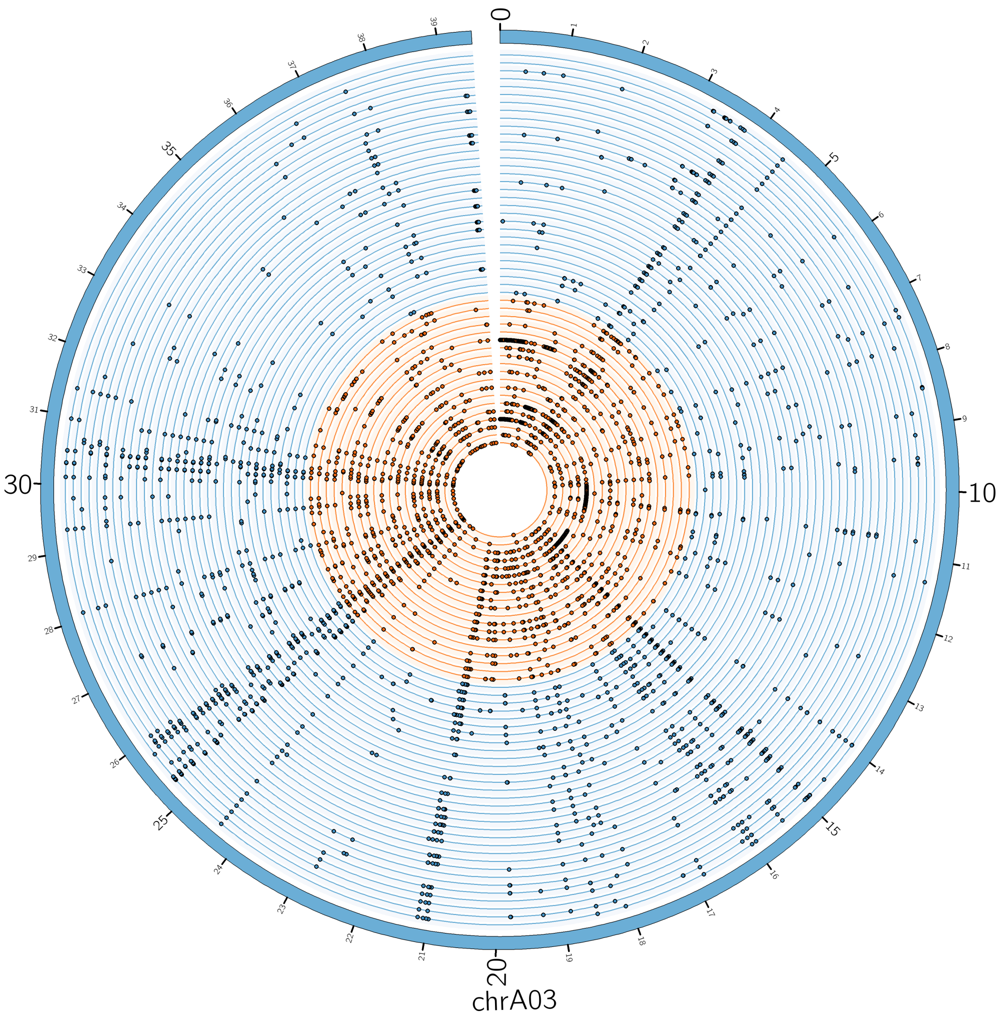

d

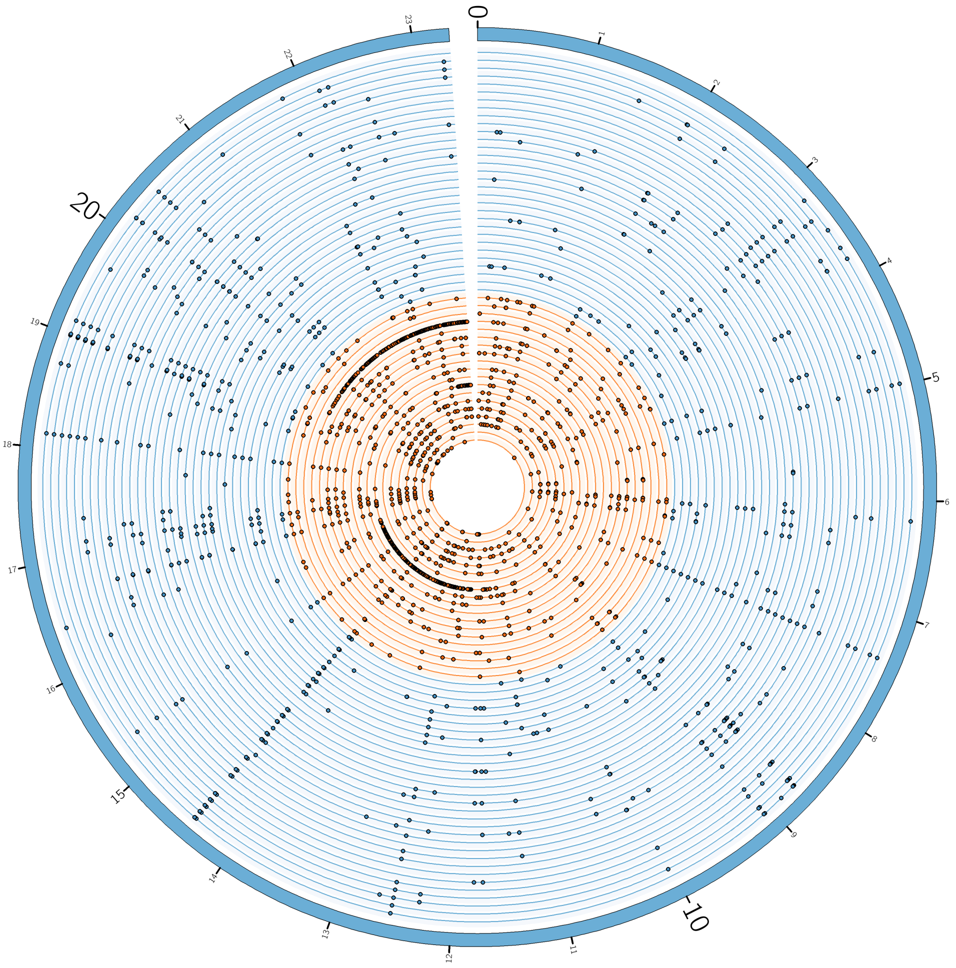

e

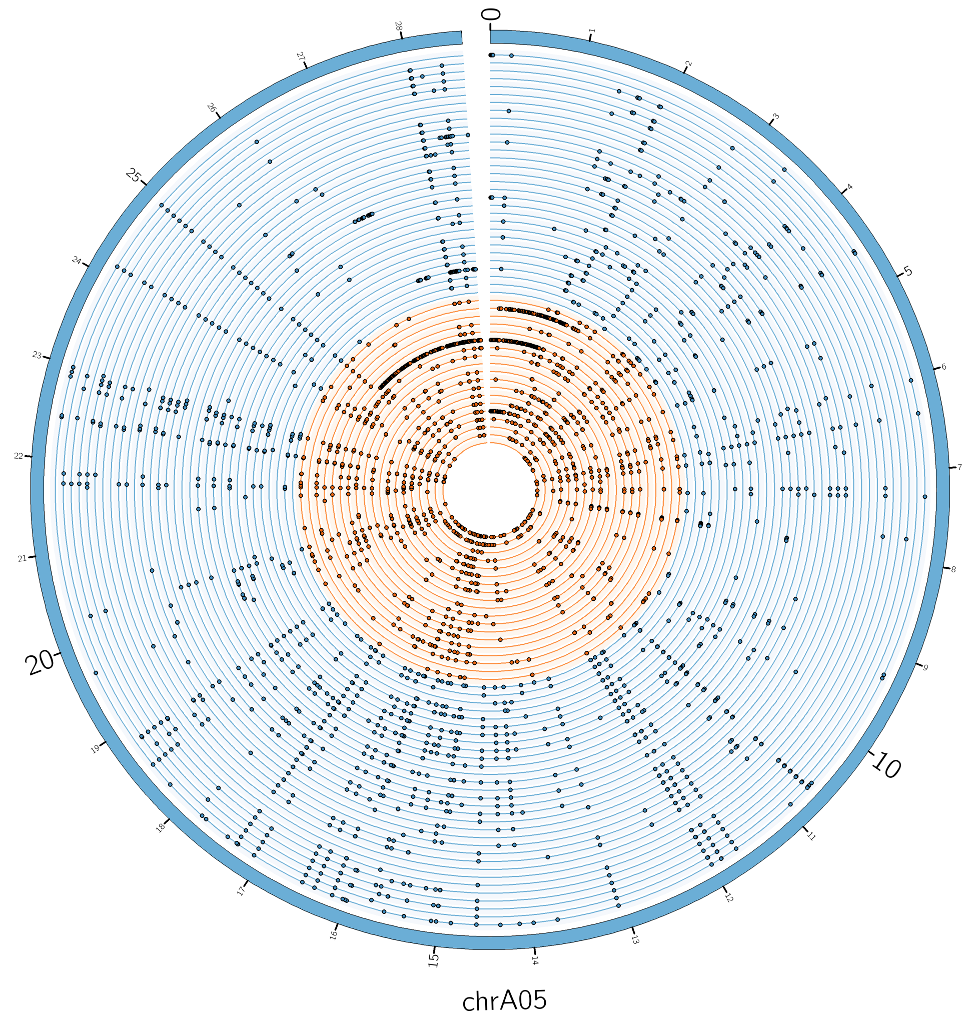

f

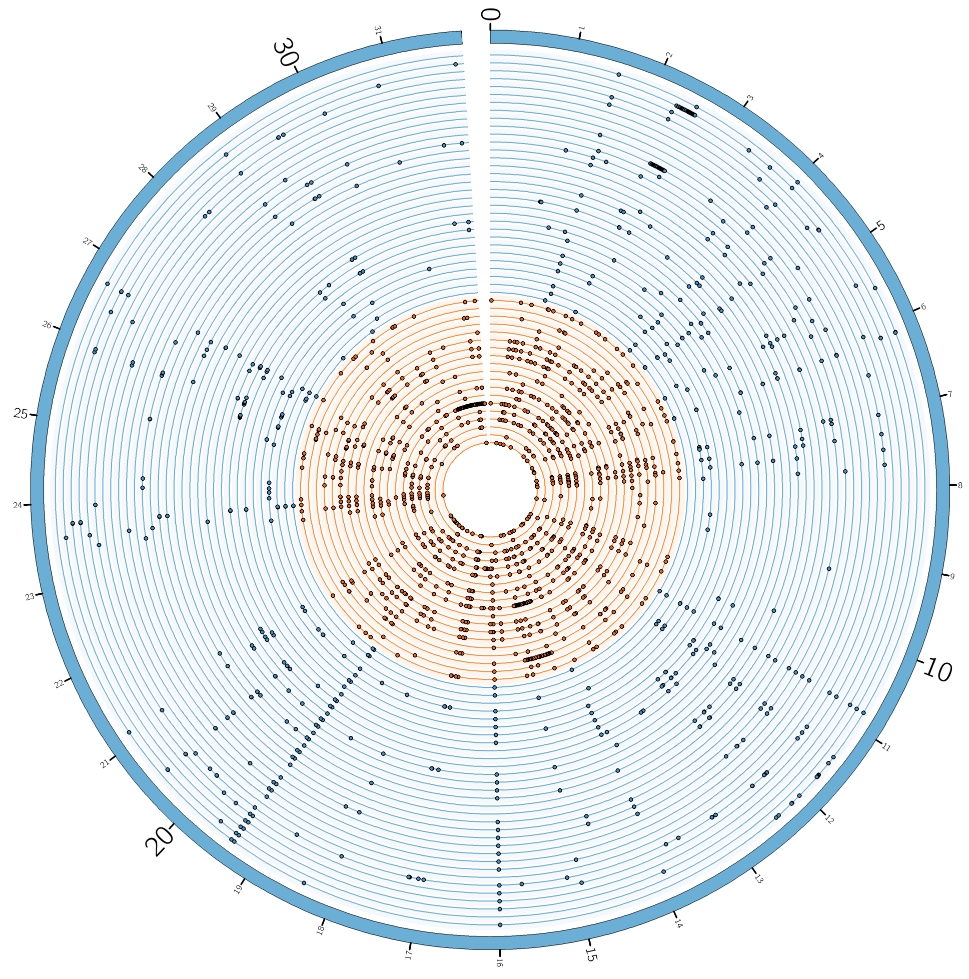

g

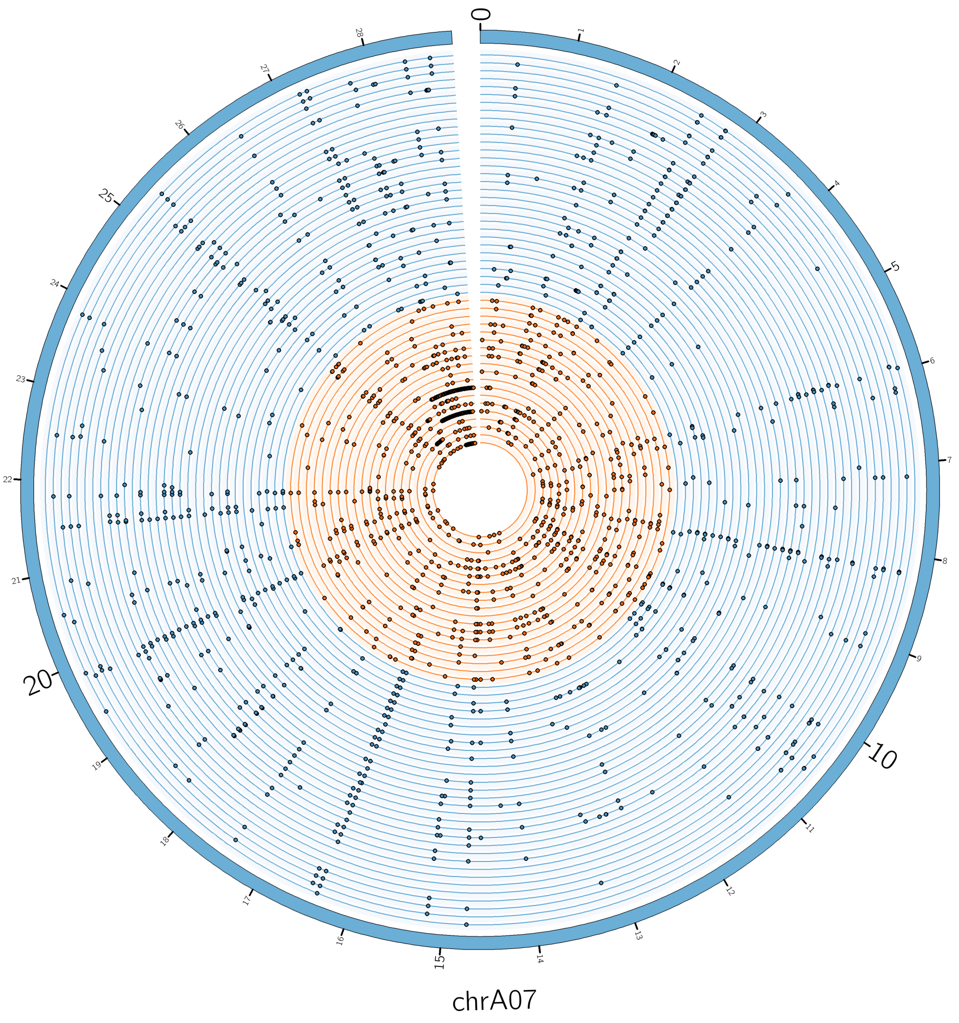

h

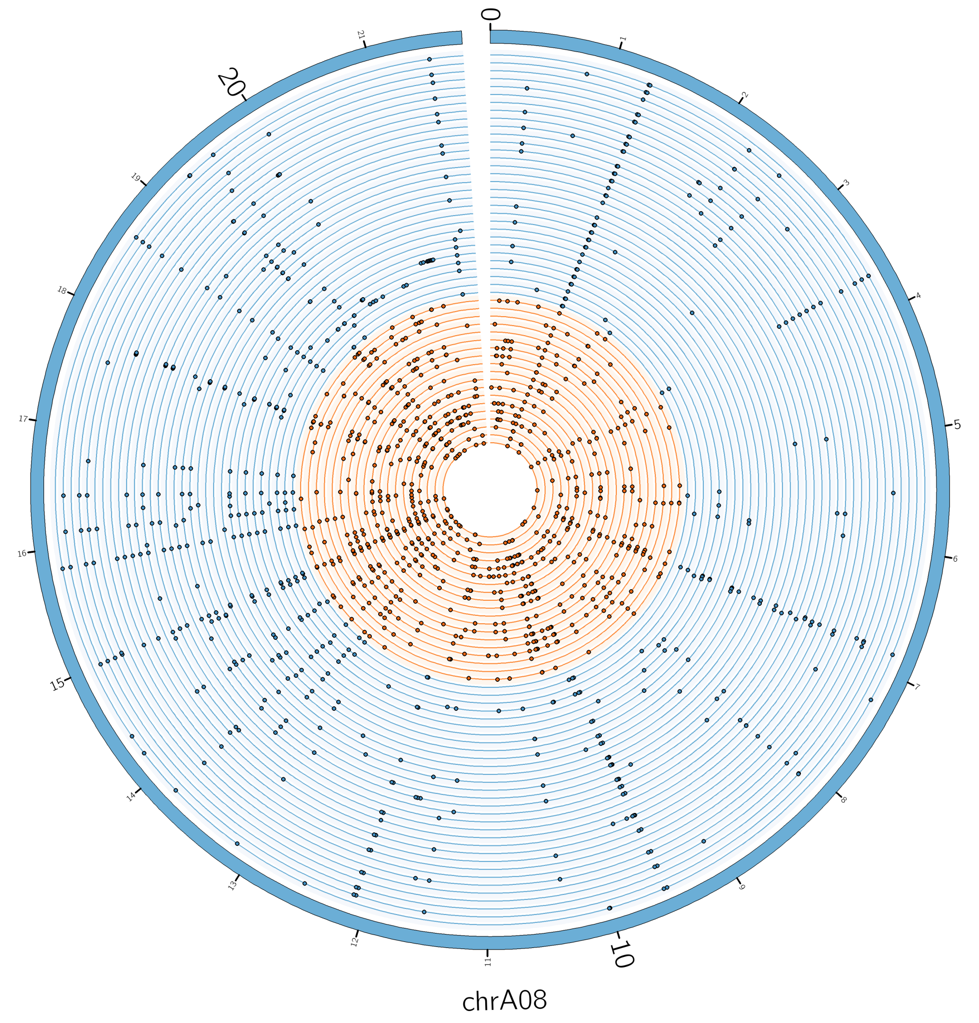

i

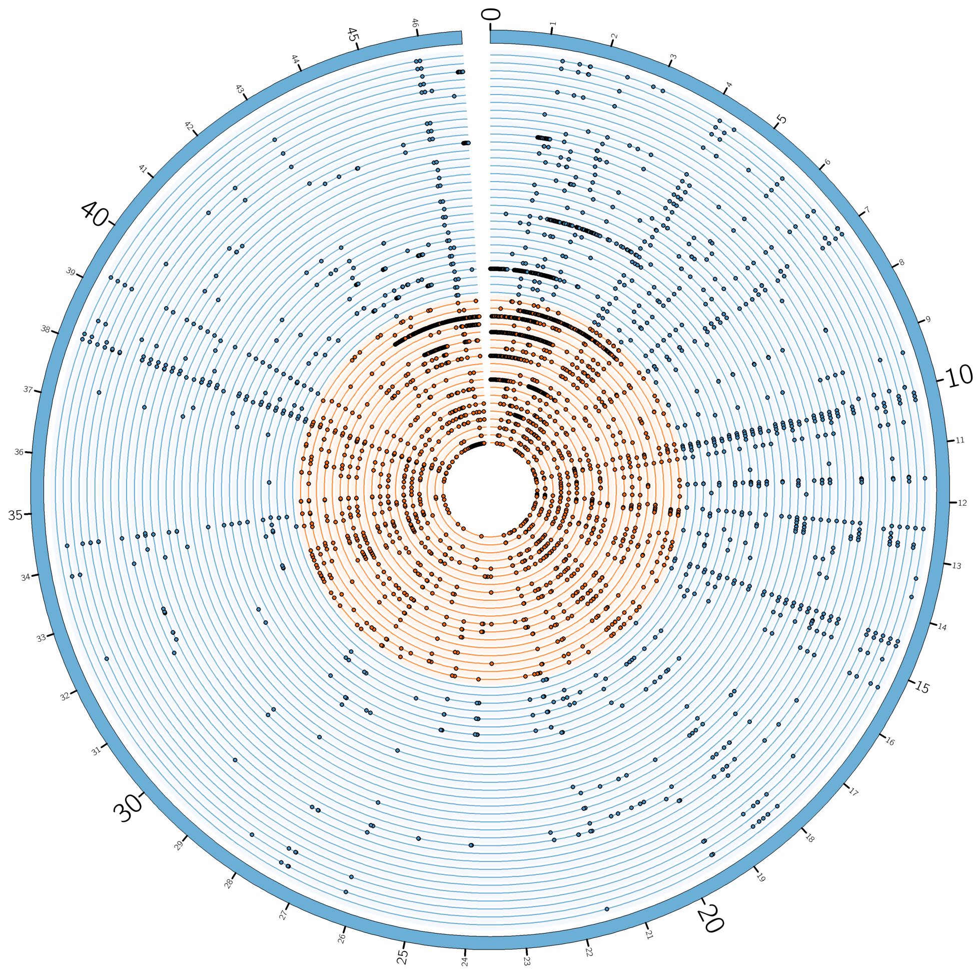

j

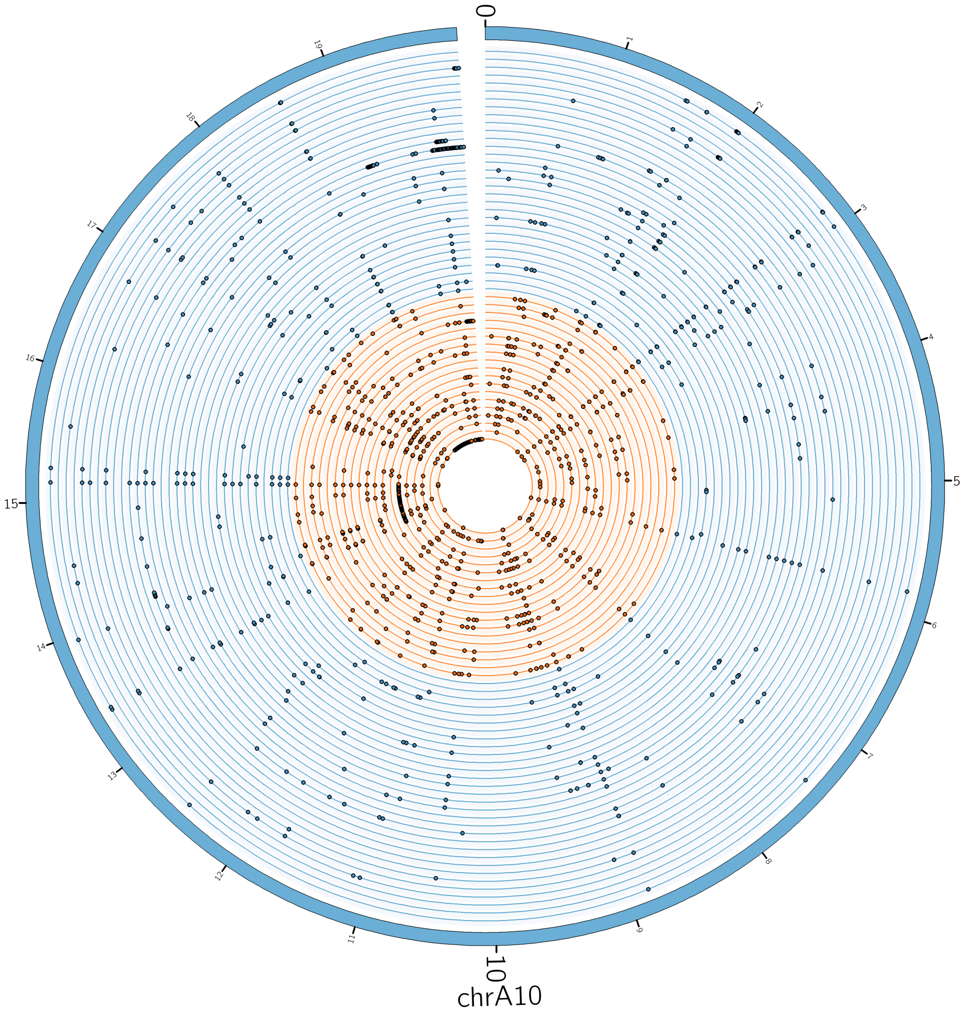

k

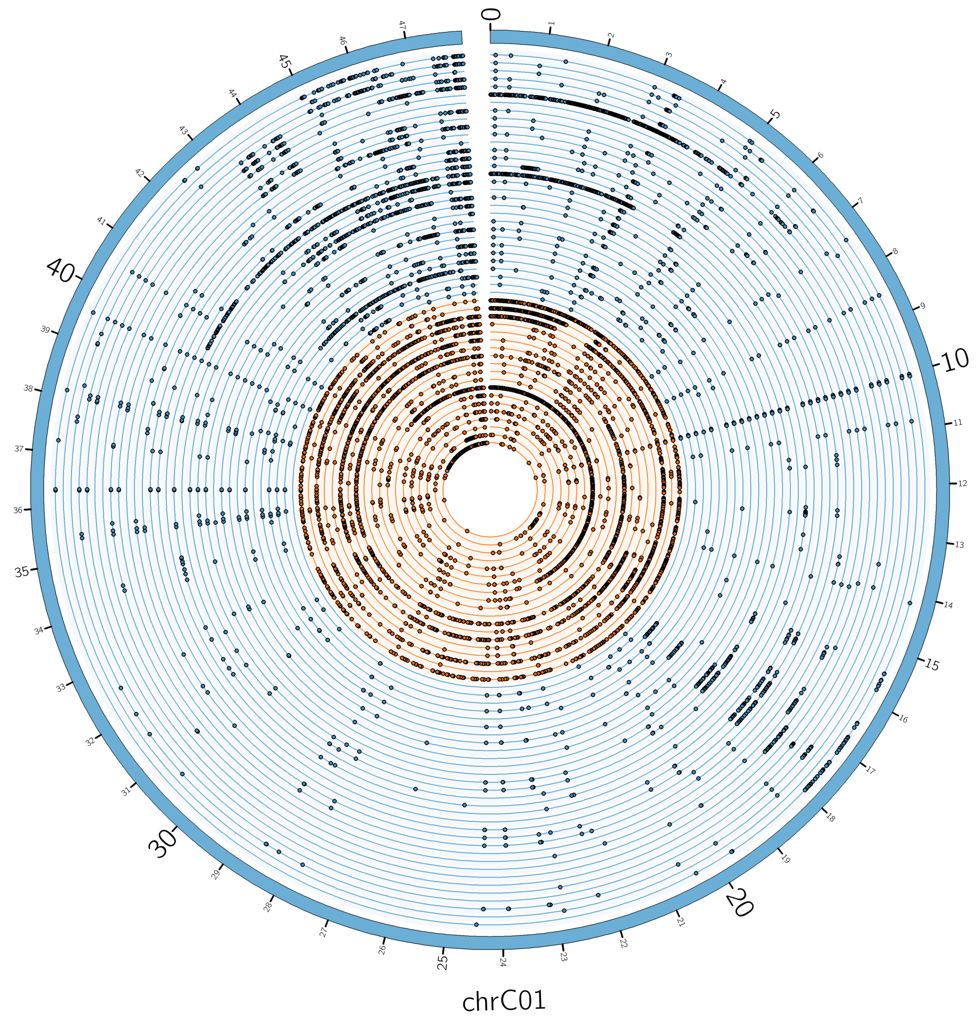

l

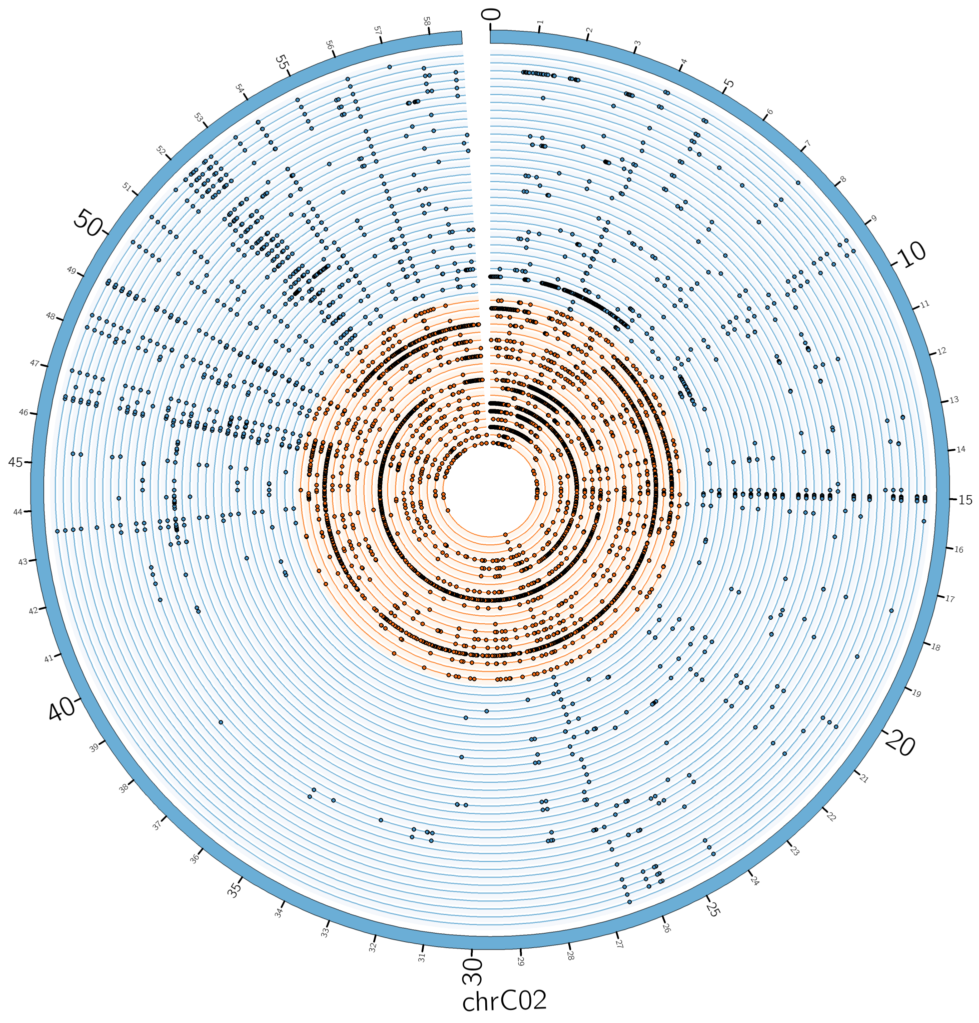

m

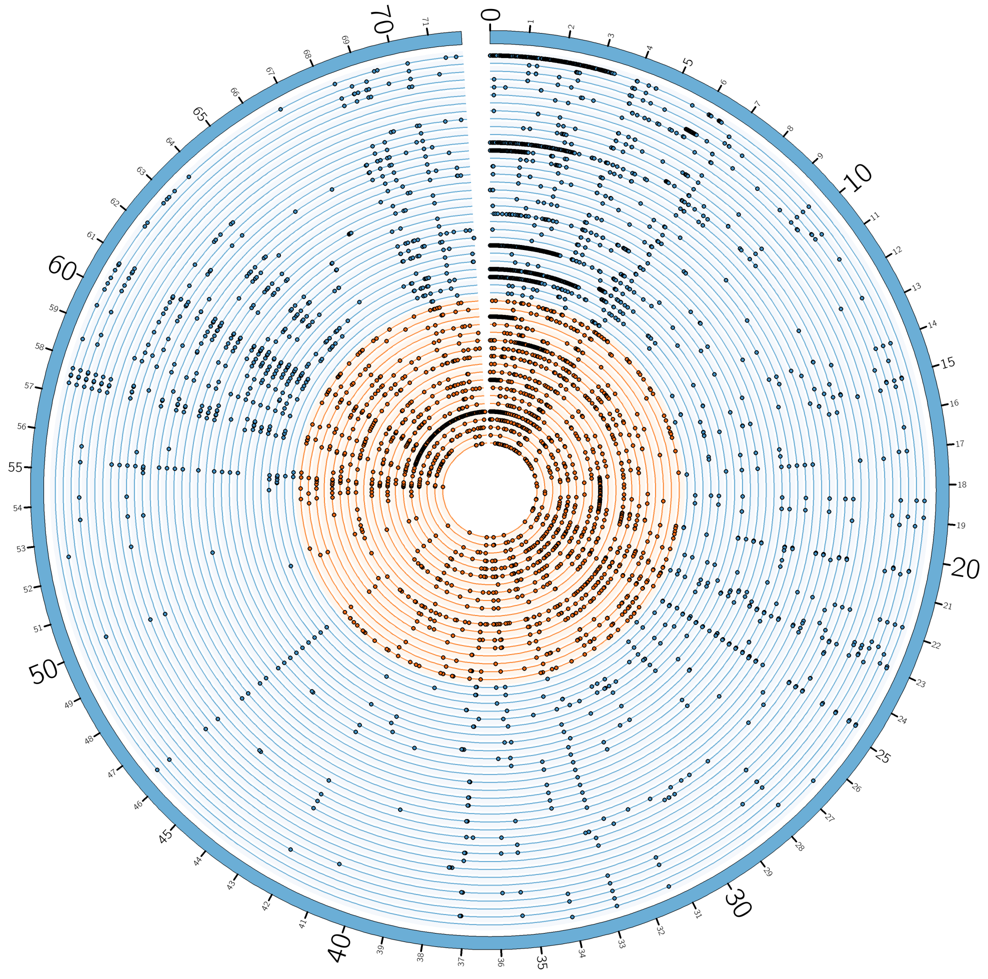

n

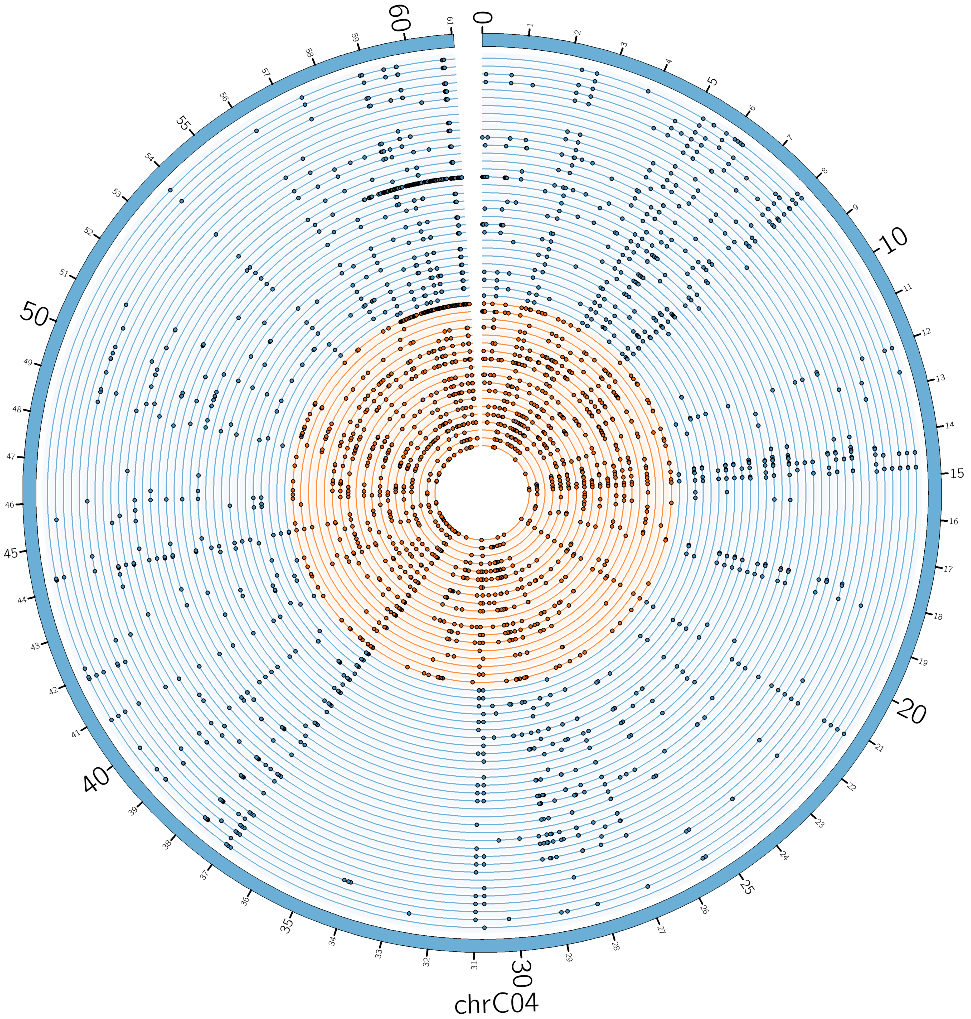

o

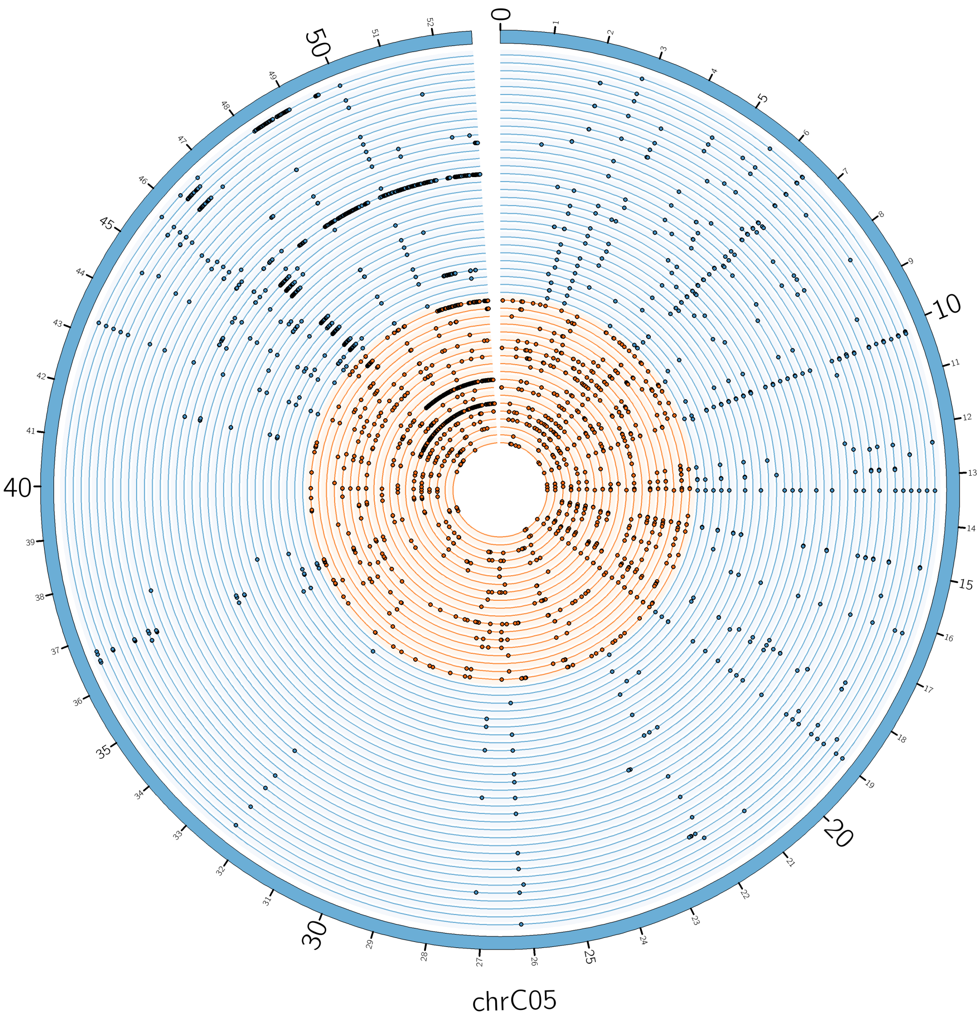

p

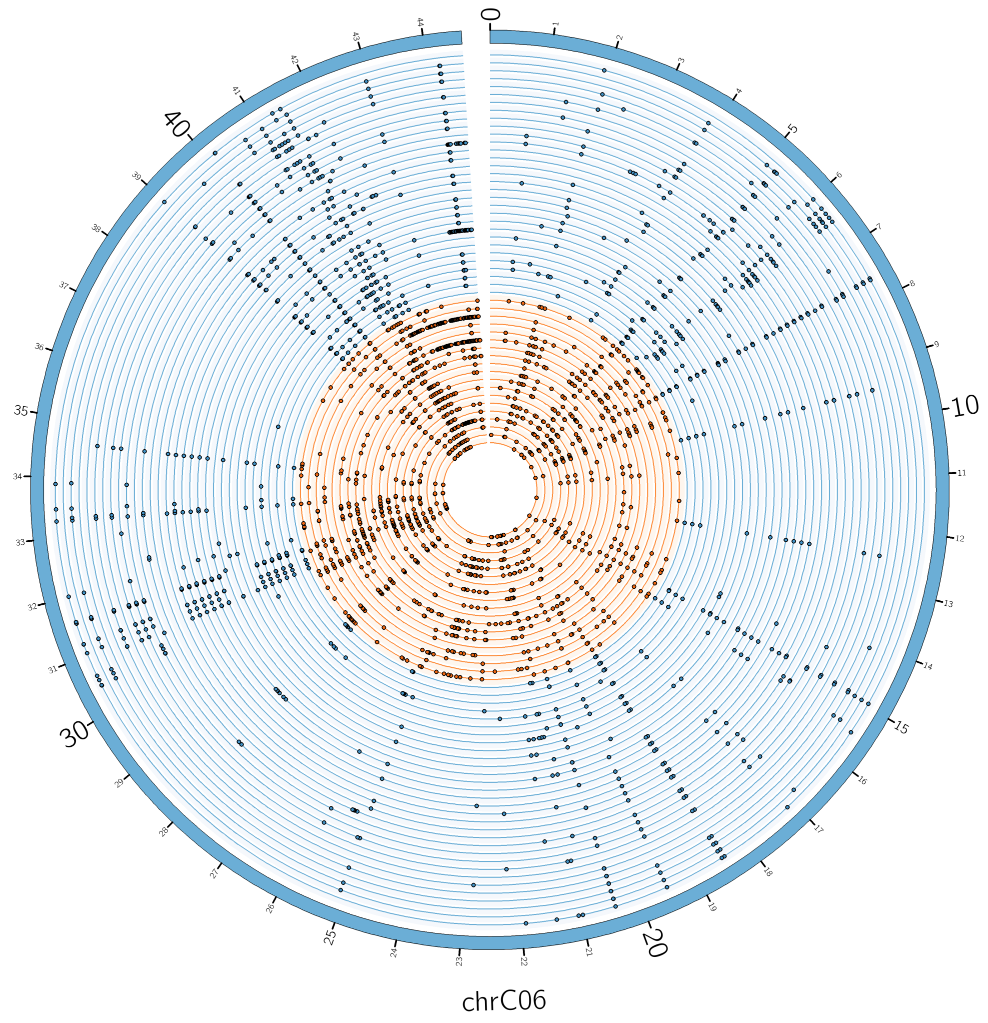

q

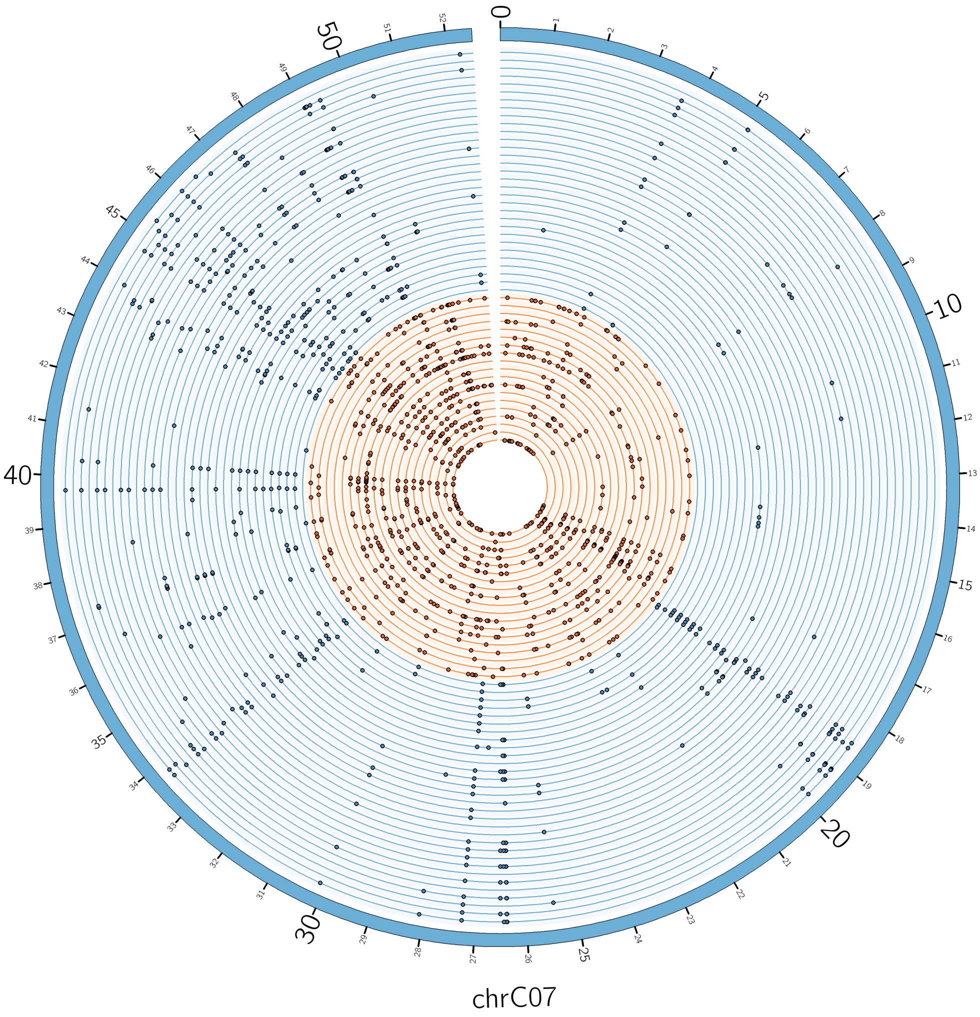

r

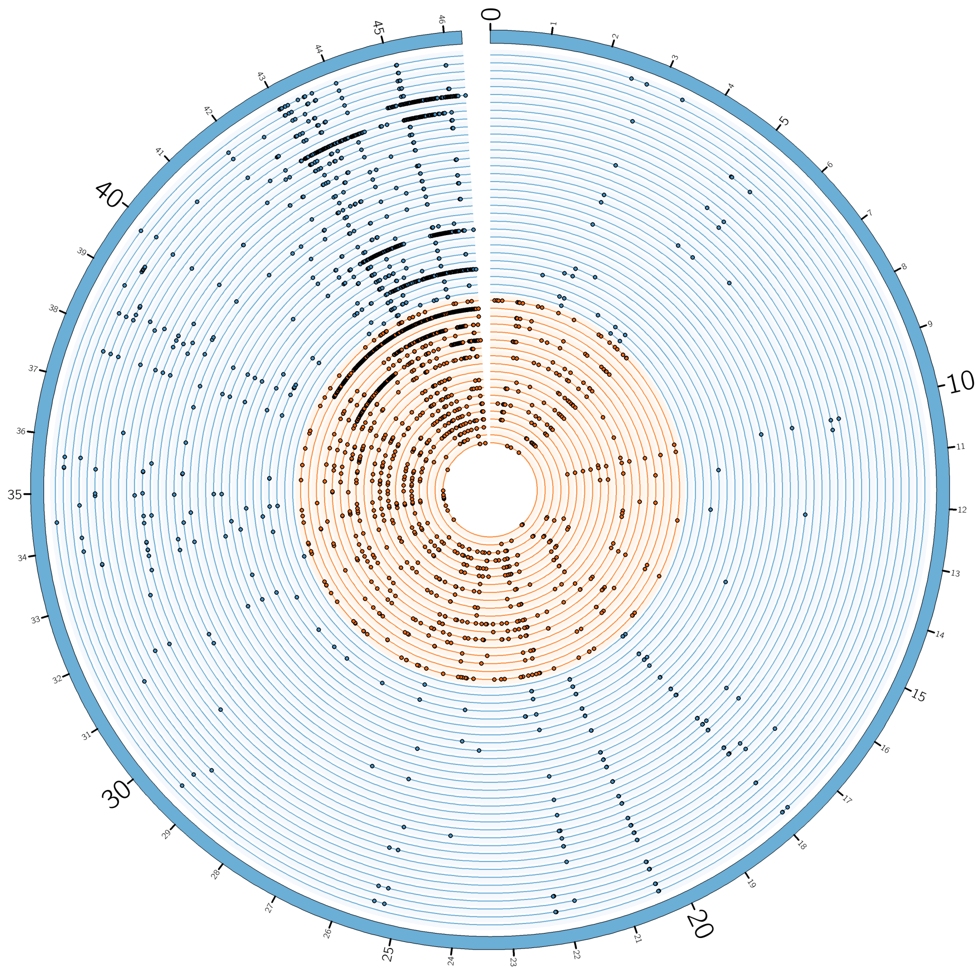

s

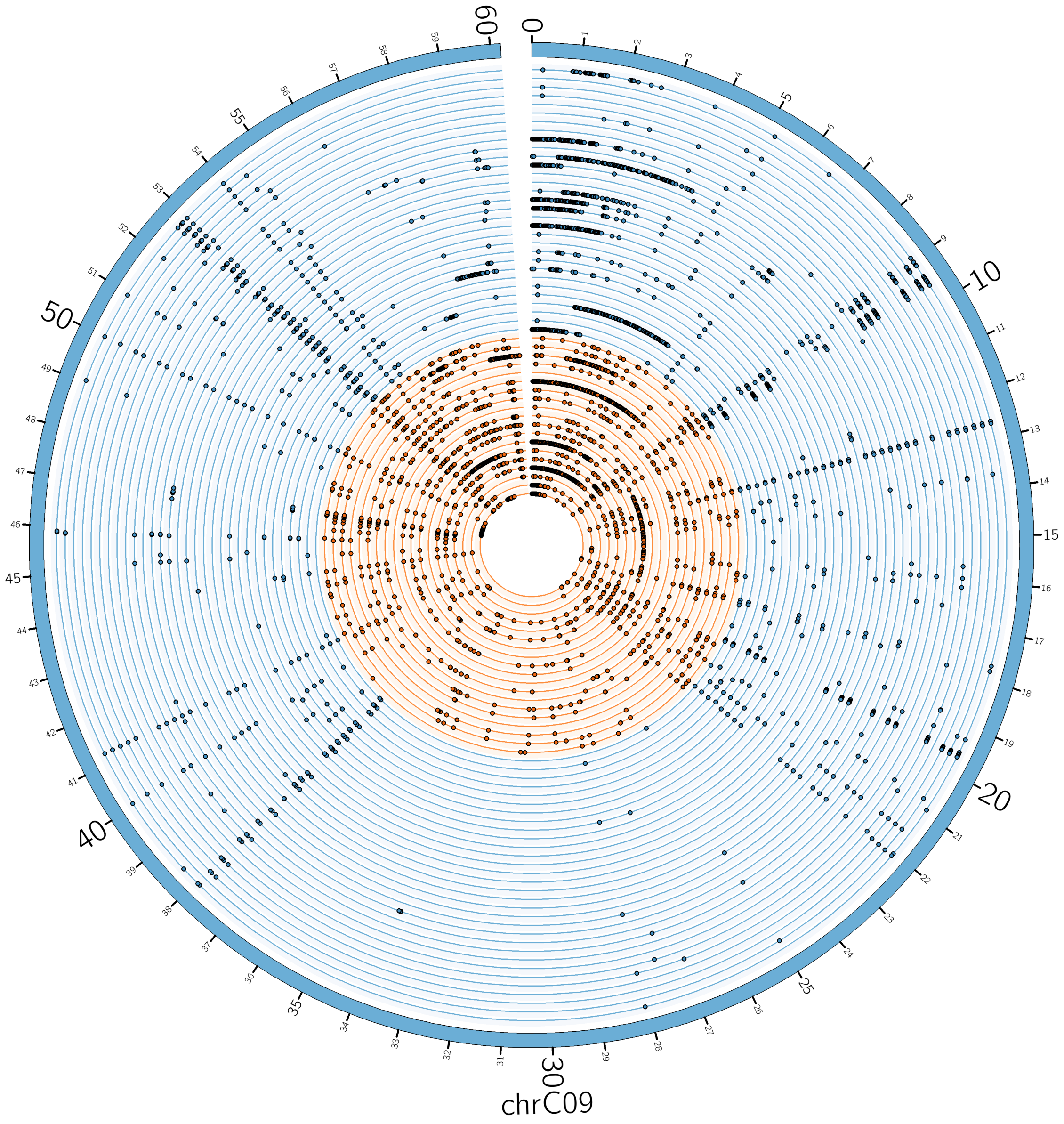

t

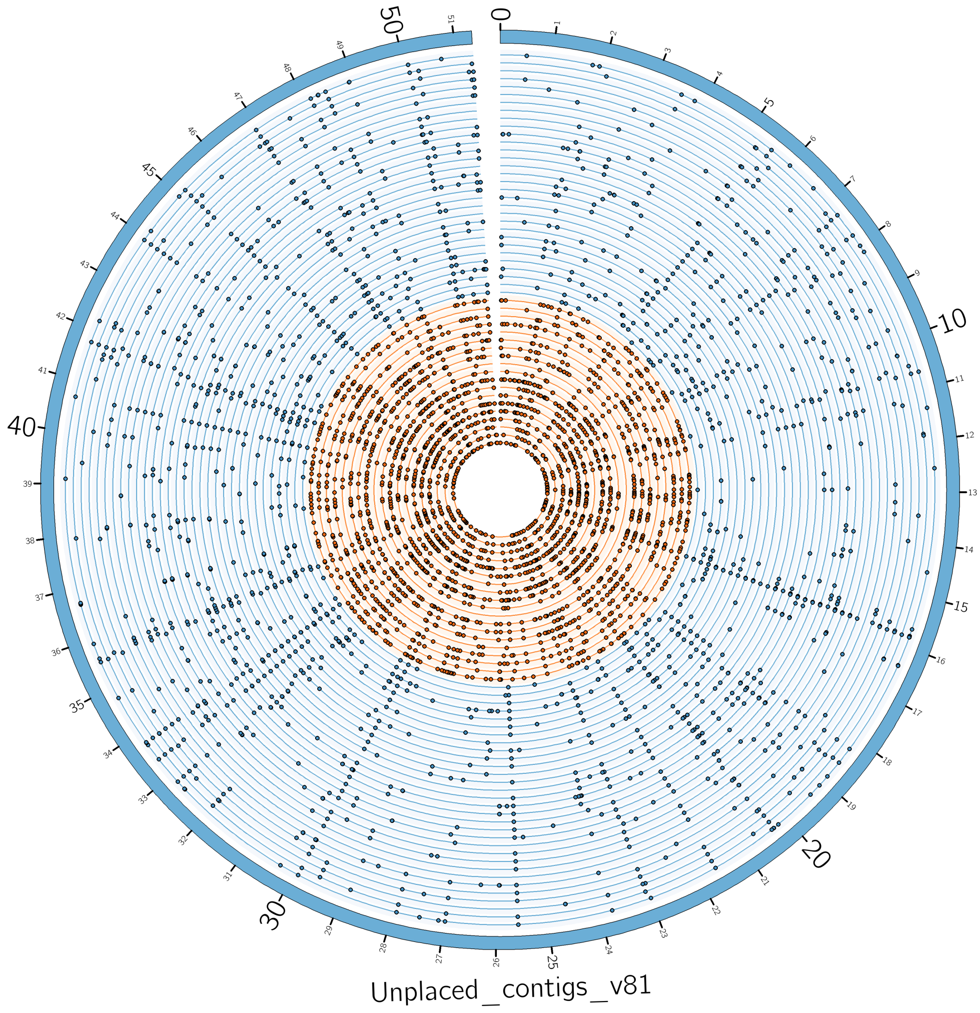

Supplementary Figure 1. Circos plots showing patterns of lost genes on the Darmor-*bzh* portion of the pangenome on the A genome (a-j), the C genome (k-s) and unplaced contigs (t). The dots represent lost genes in the non-synthetics (blue) and synthetics (orange) The order in which the accessions appear on the plots as indicated by the blue and orange rings is listed in Supplementary Table 19.

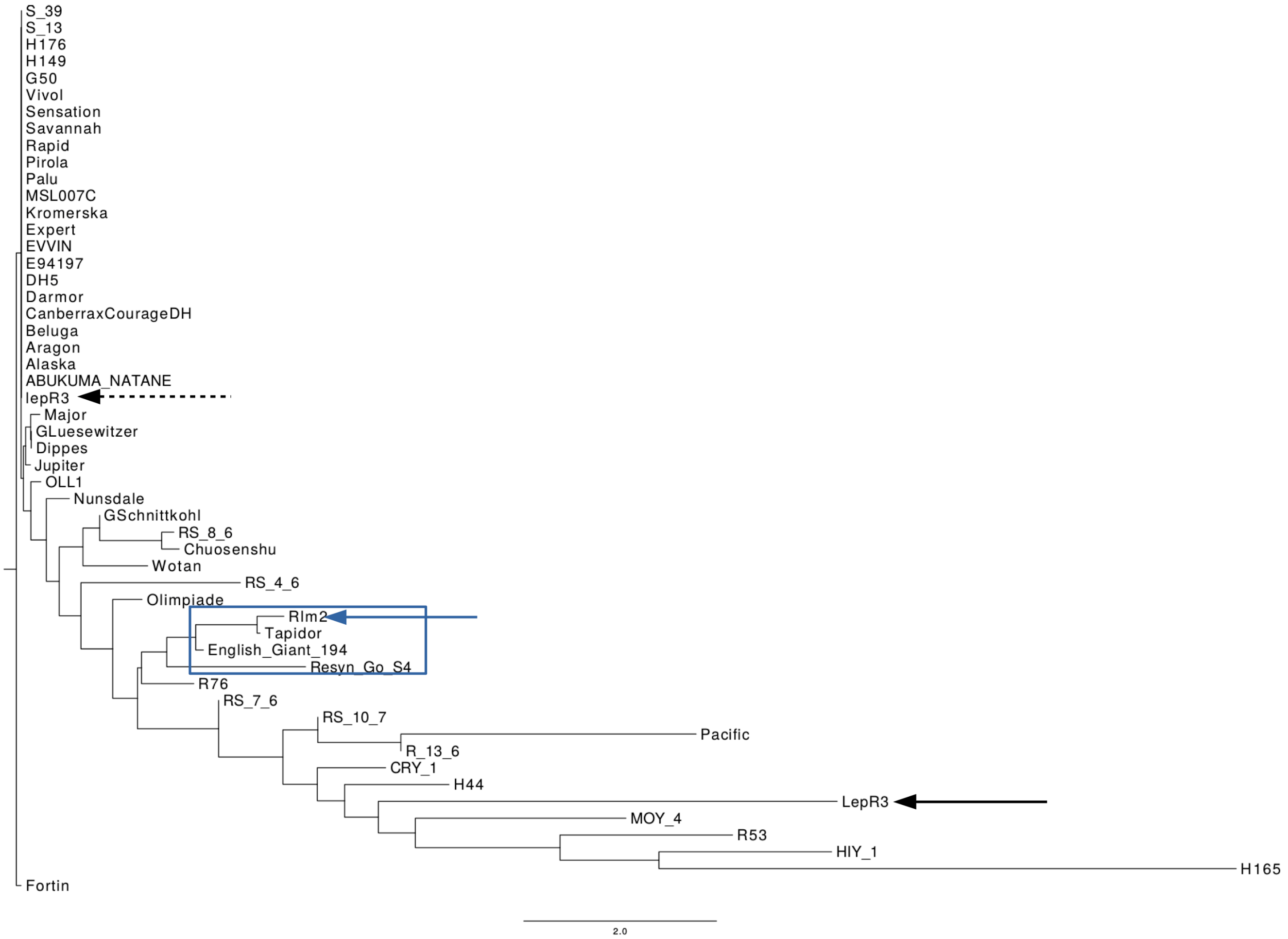

Supplementary Figure 2. Phylogenetic relationship of the allelic variants of the blackleg gene. The Neighbour Joining (NJ) tree was generated using the percentage identities of the amino acid sequences of the candidate blackleg genes in the non-synthetic and synthetic accessions. The coloured arrows point to the allelic variants (resistant and susceptible) of the genes: *lepR3/rlm2* (dashed black), *Rlm2* (blue) and *LepR3* (black).

a

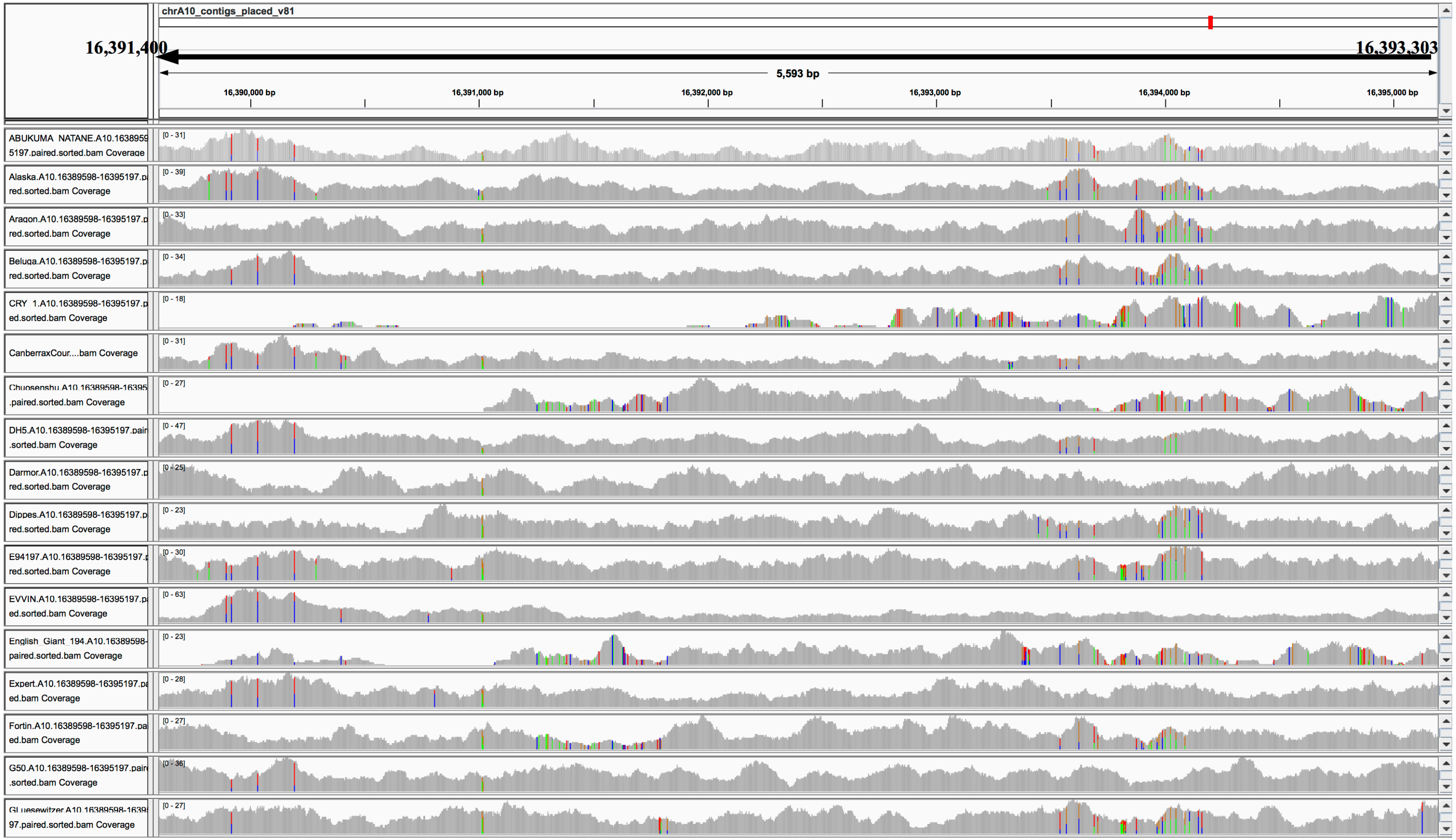
chrA10_contigs_placed_v81
16,391,400
16,393,303
5,593 bp
16,390,000 bp
16,391,000 bp
16,392,000 bp
16,393,000 bp
16,394,000 bp
16,395,000 bp
ABUKUMA NATANE.A10.16389598-16395197.paired.sorted.bam Coverage
Alaska.A10.16389598-16395197.paired.sorted.bam Coverage
Aragon.A10.16389598-16395197.paired.sorted.bam Coverage
Beluga.A10.16389598-16395197.paired.sorted.bam Coverage
CRY 1.A10.16389598-16395197.paired.sorted.bam Coverage
CanberraxCour....bam Coverage
Chuosenshu.A10.16389598-16395197.paired.sorted.bam Coverage
DH5.A10.16389598-16395197.paired.sorted.bam Coverage
Darmor.A10.16389598-16395197.paired.sorted.bam Coverage
Dippes.A10.16389598-16395197.paired.sorted.bam Coverage
E94197.A10.16389598-16395197.paired.sorted.bam Coverage
EVVIN.A10.16389598-16395197.paired.sorted.bam Coverage
English Giant 194.A10.16389598-16395197.paired.sorted.bam Coverage
Expert.A10.16389598-16395197.paired.sorted.bam Coverage
Fortin.A10.16389598-16395197.paired.sorted.bam Coverage
G50.A10.16389598-16395197.paired.sorted.bam Coverage
GLuesewitzer.A10.16389598-16395197.paired.sorted.bam Coverage
[0 - 31]
[0 - 39]
[0 - 33]
[0 - 34]
[0 - 18]
[0 - 31]
[0 - 27]
[0 - 47]
[0 - 25]
[0 - 23]
[0 - 30]
[0 - 63]
[0 - 23]
[0 - 28]
[0 - 27]
[0 - 36]
[0 - 27]

b

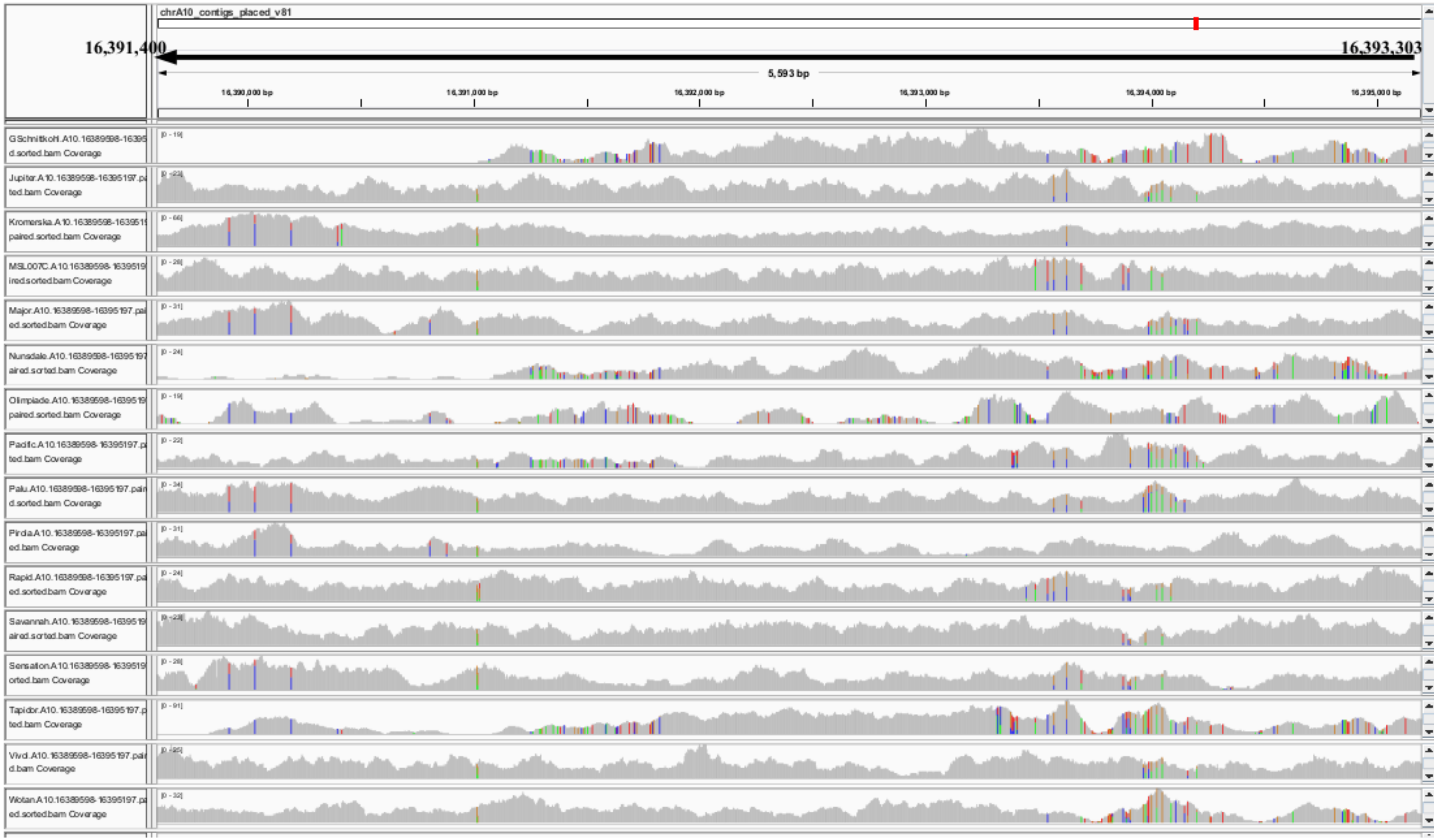

c

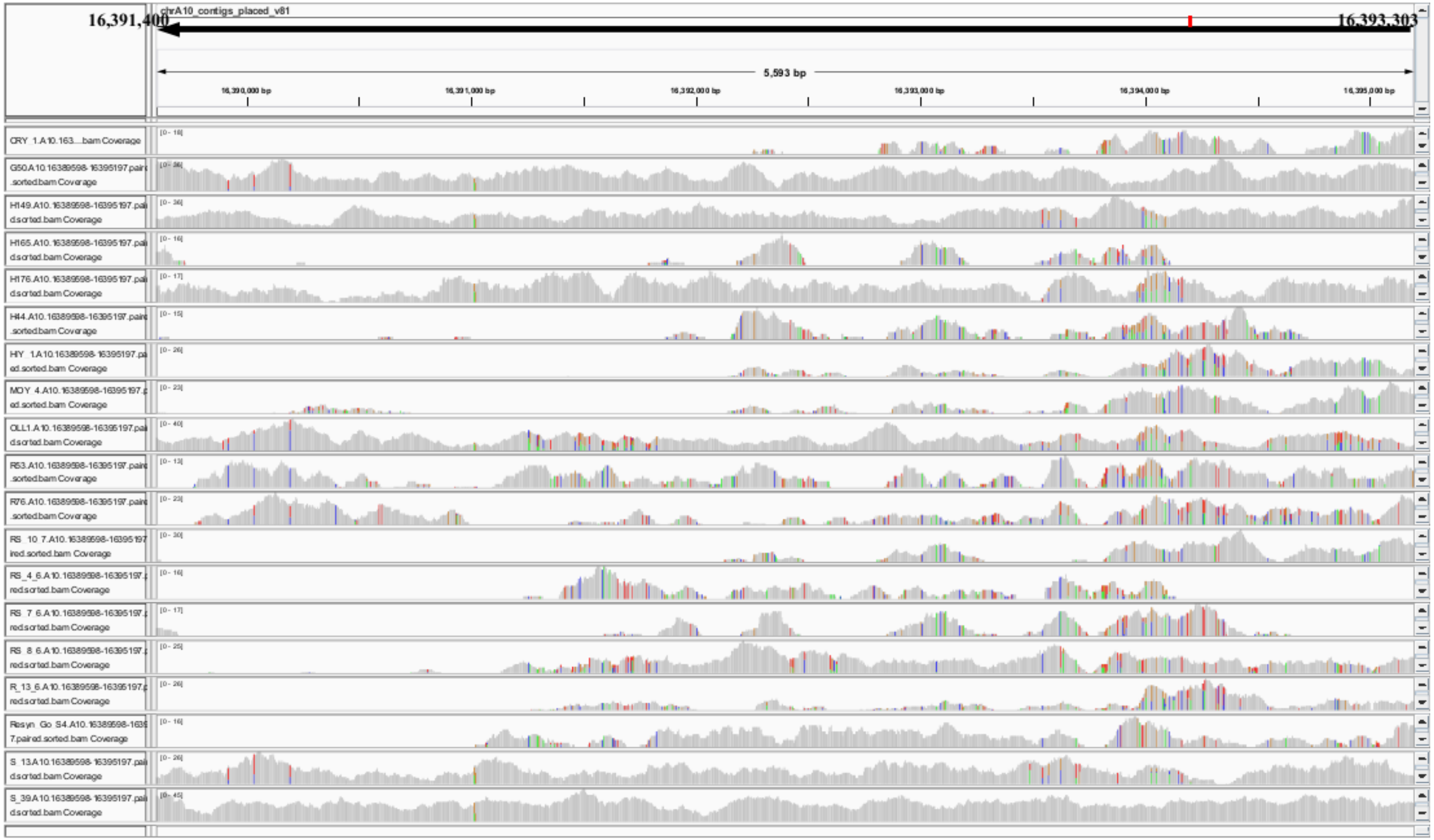
16,391,400
chrA10_contigs_placed_v81
16,393,303
5,593 bp
16,390,000 bp
16,391,000 bp
16,392,000 bp
16,393,000 bp
16,394,000 bp
16,395,000 bp

Supplementary Figure 3. Read mapping coverage in region harbouring *lepR3/rlm2* on chromosome A10 of the pangenome for the non-synthetics (a and b) and synthetics (c). The blackleg resistance gene alleles *Rlm2* and *LepR3* were not among the genes predicted in this study. However, when comparing the sequence of the susceptible allele (*lepR3/rlm2*) of the blackleg gene with the pangenome assembly, we found that it was present and occupied its expected region of 16,391,400 bp to 16,393,303 bp on chromosome A10. The sequence of the cloned gene (*lepR3*) from Darmor-*bzh* v4.0 (BnaA10g20720D) was used to delimit the exact location of the gene in the pangenome assembly (the black arrow indicates the orientation of BnaA10g20720D and where it aligns in the pangenome) as *lepR3/rlm2* was not annotated in this version of the assembly presumably due to the strict repeat masking employed during the gene annotation process to avoid the calling of false PAVs. According to read mapping coverage in the target region across all the accessions used in this study, this region was identified as being part of the core genome, but lower read mapping coverage was observed with respect to the non-synthetics suggesting that *lepR3/rlm2* may have diverged in these accessions, reflecting their diverse origins.

a

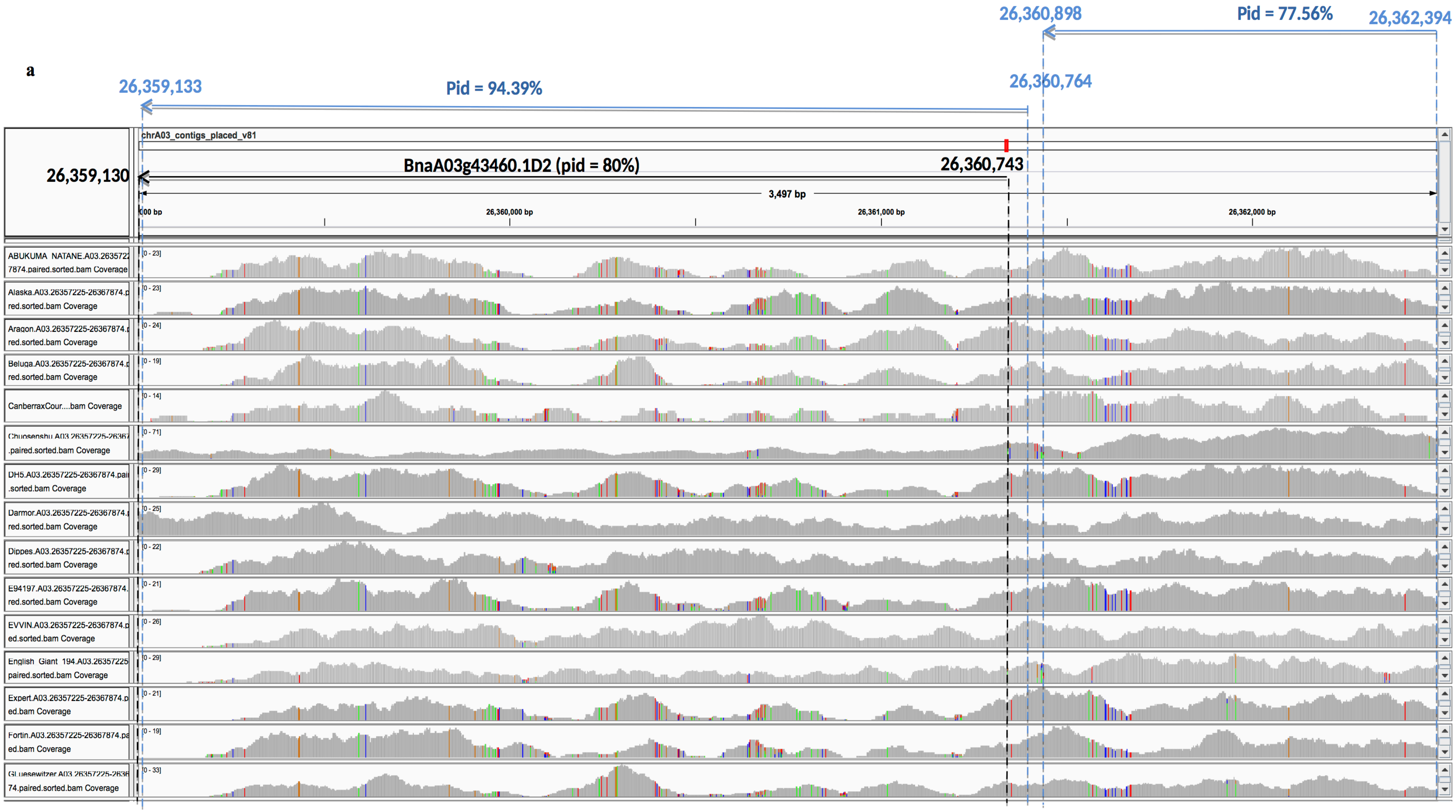

26,360,898
Pid = 77.56%
26,362,394
26,359,133
Pid = 94.39%
26,360,764
chrA03_contigs_placed_v81
26,359,130
BnaA03g43460.1D2 (pid = 80%)
26,360,743
3,497 bp
26,360,000 bp
26,361,000 bp
26,362,000 bp
ABUKUMA NATANE.A03.2635722 7874.paired.sorted.bam Coverage
Alaska.A03.26357225-26367874.p red.sorted.bam Coverage
Aragon.A03.26357225-26367874.p red.sorted.bam Coverage
Beluga.A03.26357225-26367874.p red.sorted.bam Coverage
CanberraxCour....bam Coverage
Chuosenshu.A03.26357225-26367 .paired.sorted.bam Coverage
DH5.A03.26357225-26367874.pai .sorted.bam Coverage
Darmor.A03.26357225-26367874.p red.sorted.bam Coverage
Dippes.A03.26357225-26367874.p red.sorted.bam Coverage
E94197.A03.26357225-26367874. red.sorted.bam Coverage
EVVIN.A03.26357225-26367874.p ed.sorted.bam Coverage
English Giant 194.A03.26357225 paired.sorted.bam Coverage
Expert.A03.26357225-26367874.p ed.bam Coverage
Fortin.A03.26357225-26367874.pa ed.bam Coverage
GLuesewitzer.A03.26357225-2636 74.paired.sorted.bam Coverage

b

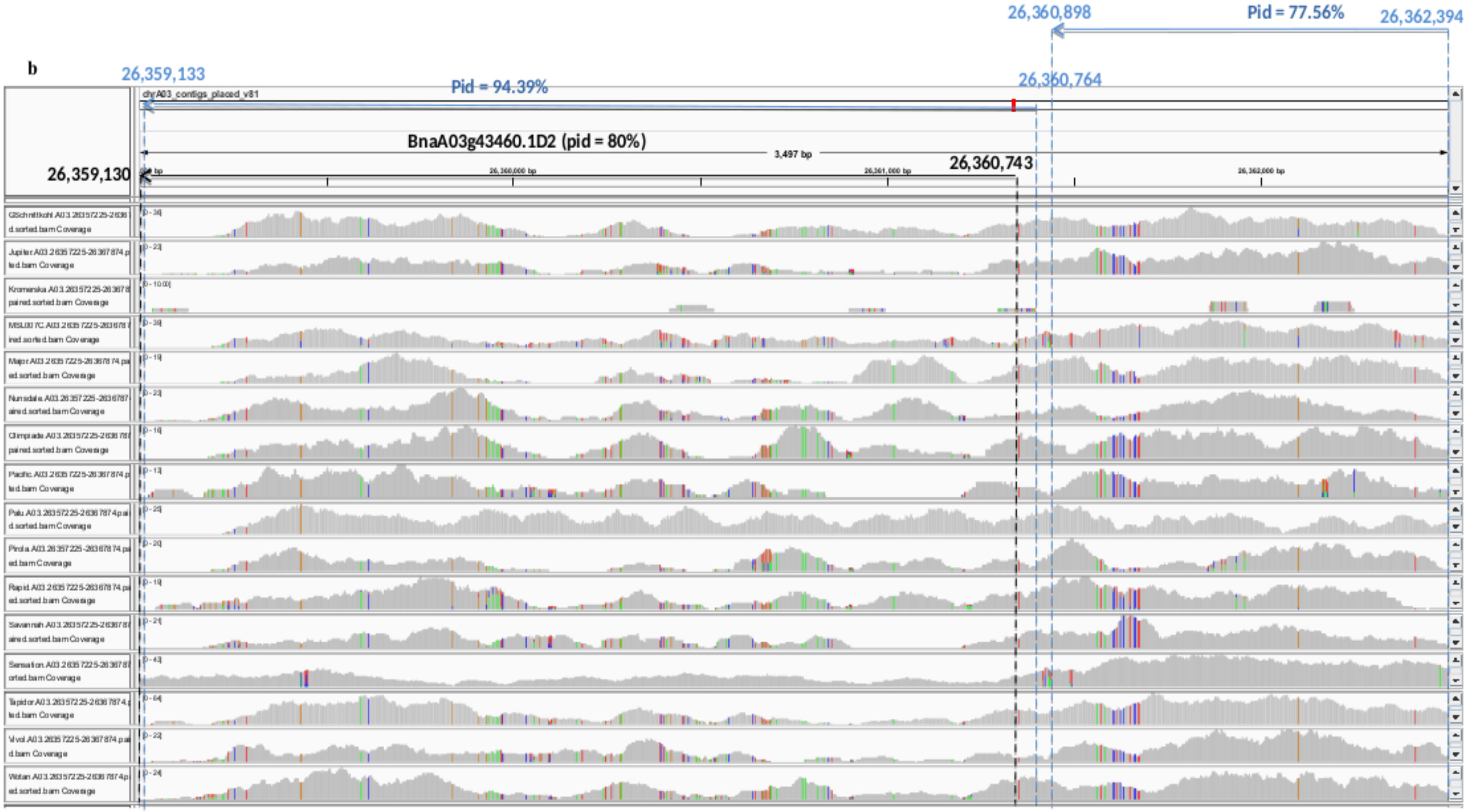
26,360,898
Pid = 77.56%
26,362,394
26,359,133
Pid = 94.39%
26,360,764
chrA03_contigs_placed_v81
BnaA03g43460.1D2 (pid = 80%)
3,497 bp
26,359,130
26,360,743

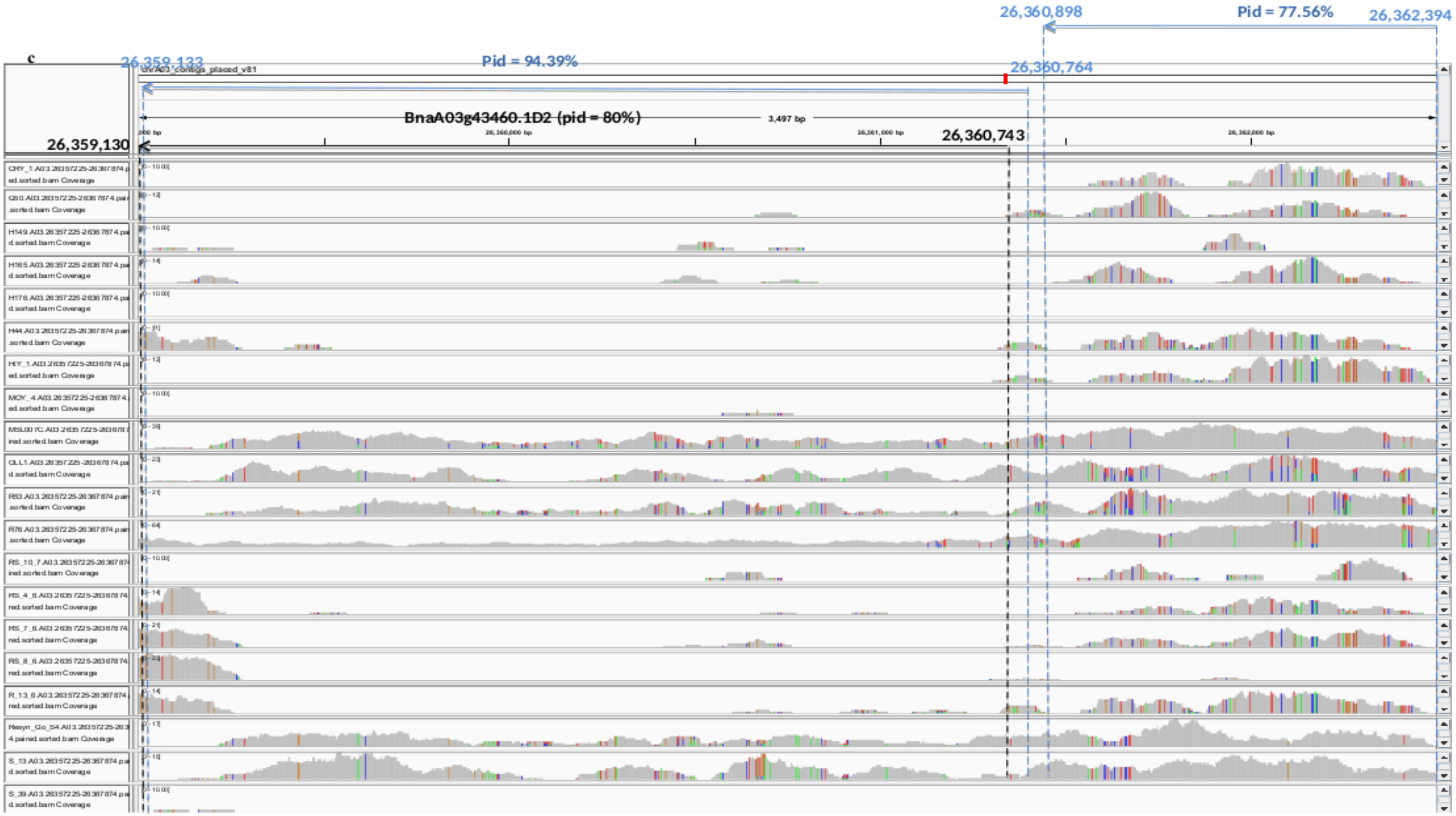
c
26,360,898
Pid = 77.56%
26,362,394
26,359,133
Pid = 94.39%
26,360,764
BnaA03g43460.1D2 (pid = 80%)
26,359,130
26,360,743

Supplementary Figure 4. Read mapping coverage in the region harbouring BnaA03g43460.1D2, which was identified as the potential candidate ortholog of the *B. rapa* gene, *CRa* on chromosome A03 of the pangenome in (a and b) non-synthetics and (c) synthetics. The black arrow indicates the position of BnaA03g43460.1D2, which was identified as a variable gene. The observations that BnaA03g43460.1D2 contained one exon while the *B. rapa CRa* gene contained four, and that BnaA03g43460.1D2 was missing two out of the three predicted domains of *CRa* could be due to an assembly artefact. Therefore, to determine whether BnaA03g43460.1D2 was truly variable, the CDS sequence of *CRa* was aligned to the reference using BLAST (the blue arrows delimit the positions of where the CDS sequence aligns) and read mapping coverage was inspected in the target region. The results from both steps concurred, confirming that BnaA03g43460.1D2, together with its flanking regions was variable. Lower read mapping coverage was observed in the synthetics compared to the non-synthetics suggesting that BnaA03g43460.1D2 may be highly diverged in the synthetic accessions. BnaA03g43460.1D2 was identified as being absent in the following accessions based on gene PAV results: CRY_1, G50, H176, HIY_1, MOY_4 and S_39.

a

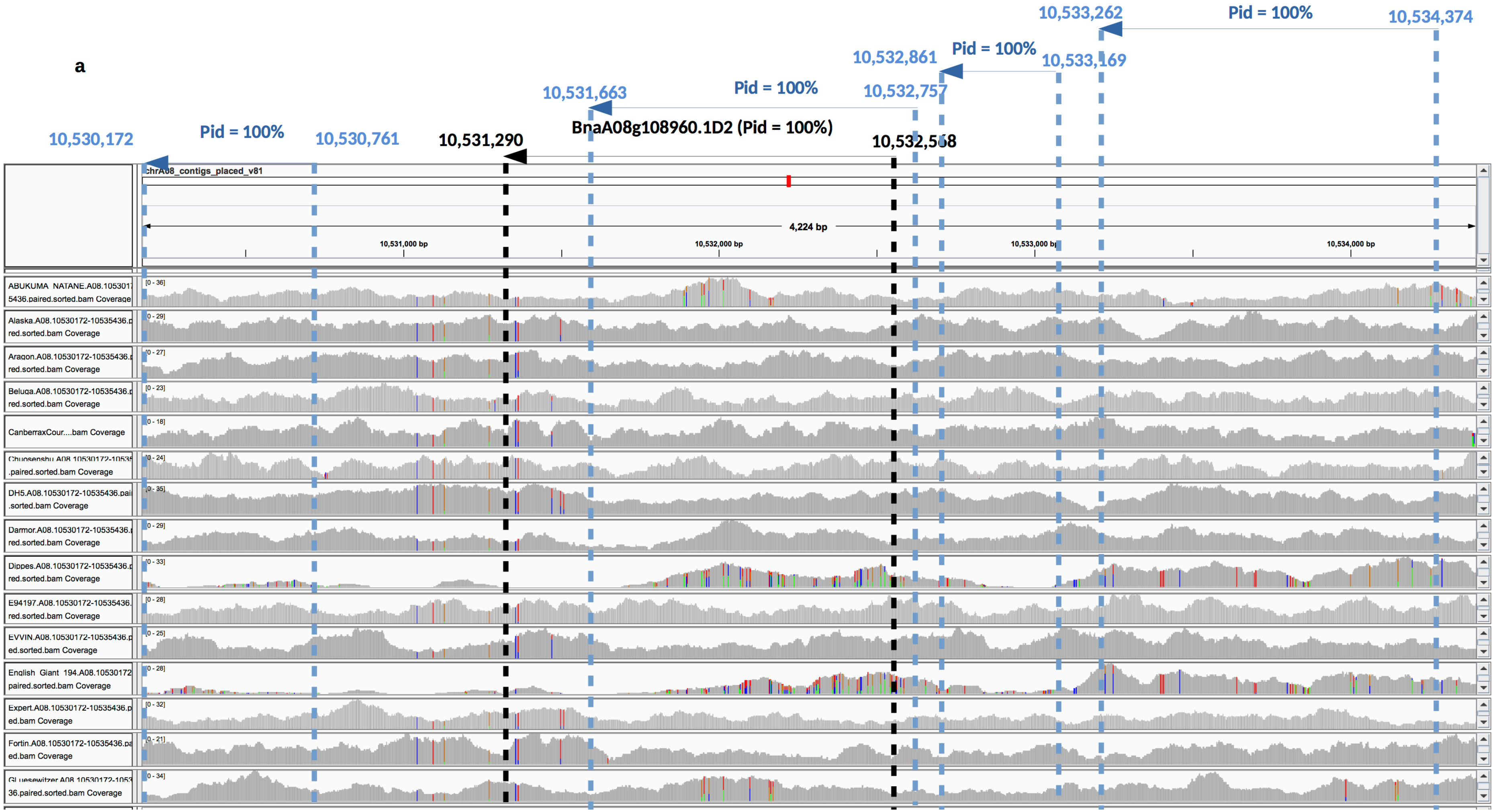
10,533,262
Pid = 100%
10,534,374
10,532,861
Pid = 100%
10,533,169
10,531,663
Pid = 100%
10,532,757
10,530,172
Pid = 100%
10,530,761
10,531,290
BnaA08g108960.1D2 (Pid = 100%)
10,532,568
chrA08_contigs_placed_v81
4,224 bp
10,531,000 bp
10,532,000 bp
10,534,000 bp
CanberraxCour....bam Coverage

b

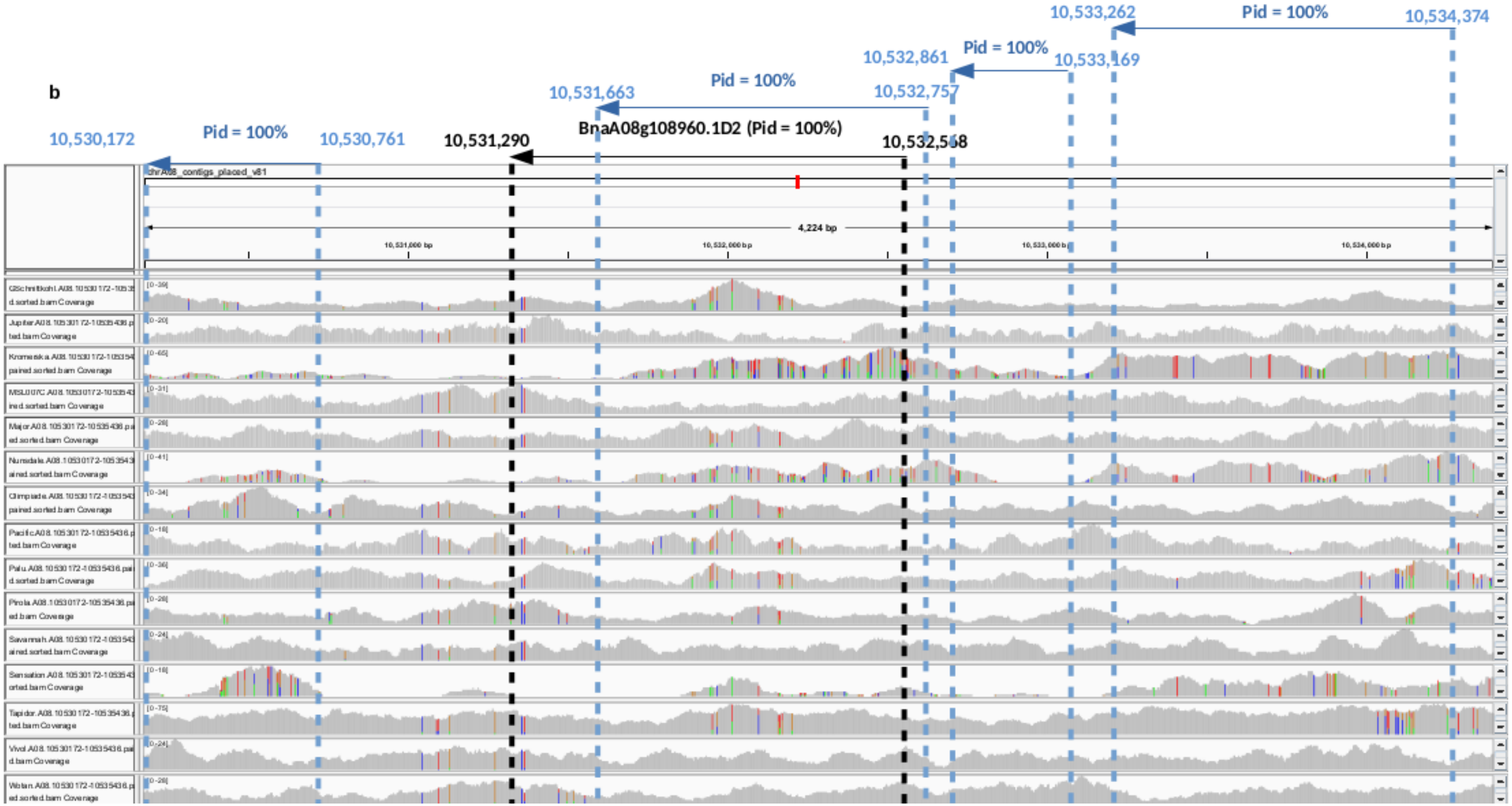
10,533,262
Pid = 100%
10,534,374
10,532,861
Pid = 100%
10,533,169
Pid = 100%
10,531,663
10,532,757
BnaA08g108960.1D2 (Pid = 100%)
10,530,172
Pid = 100%
10,530,761
10,531,290
10,532,568
4,224 bp

c

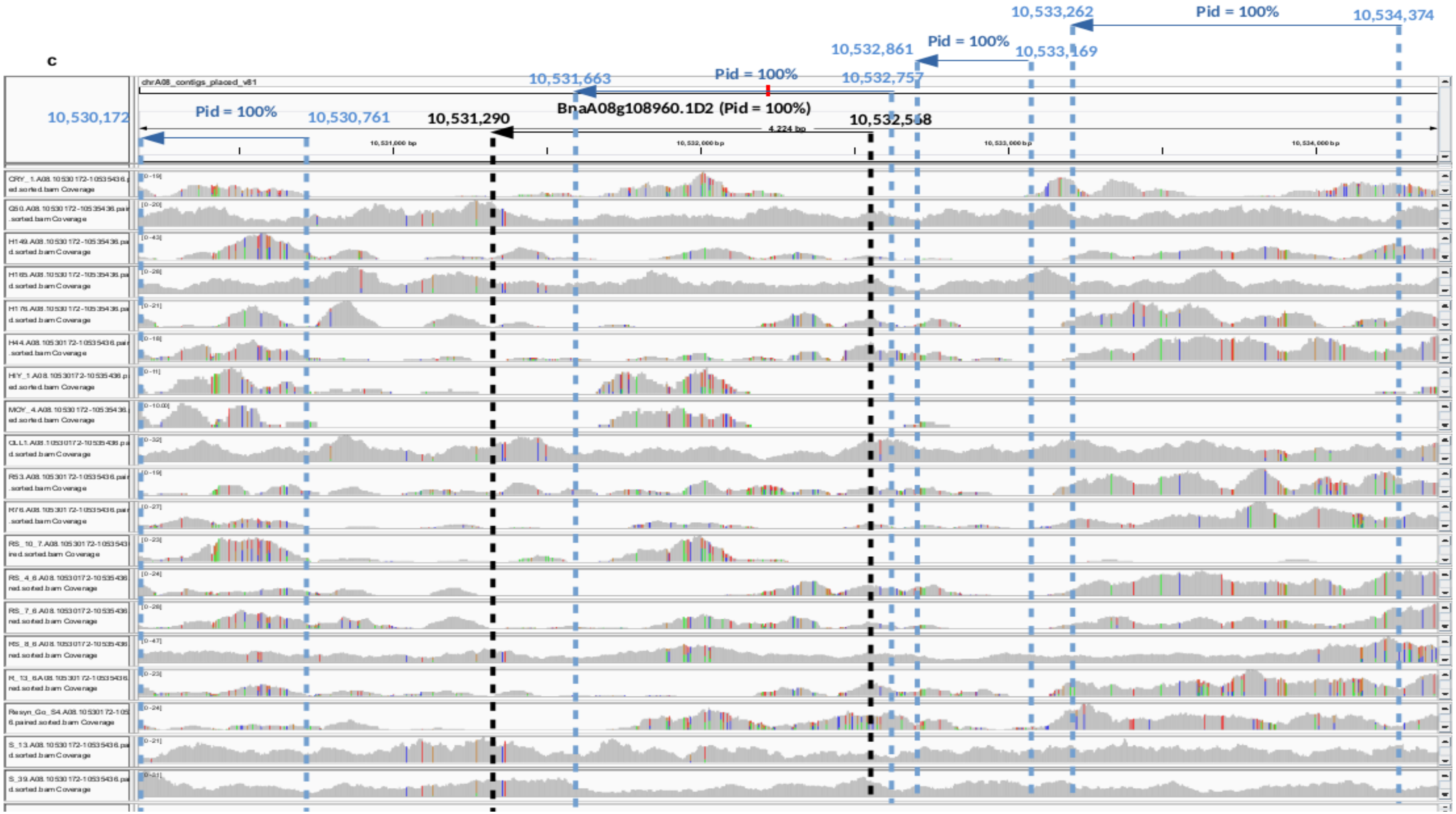
10,533,262
Pid = 100%
10,534,374
10,532,861
Pid = 100%
10,533,169
10,531,663
Pid = 100%
10,532,757
BnaA08g108960.1D2 (Pid = 100%)
10,530,172
Pid = 100%
10,530,761
10,531,290
10,532,568

Supplementary Figure 5. Read mapping coverage in the region harbouring BnA08g08960.1D2, which was identified as the potential candidate ortholog of the *B. rapa* gene, *crr1a* on chromosome A08 of the pangenome in (a and b) non-synthetics and (c) synthetics. The black arrow indicates the position of BnA08g08960.1D2, which was identified as a core gene. The observations that BnA08g08960.1D2 contained one exon while the *B. rapa crr1a* gene contained four, and that BnA08g08960.1D2 was missing two out of the four predicted domains of *crr1a* could be due to an assembly artefact. Therefore, to determine whether BnA08g08960.1D2 was truly variable, the CDS sequence of *crr1a* was aligned to the reference using BLAST (the blue arrows delimit the positions of where the CDS sequence aligns) and read mapping coverage was inspected in the target region. The results from both steps concurred, confirming that BnA08g08960.1D2, together with its flanking regions was core. Lower read mapping coverage was observed in the synthetics compared to the non-synthetics suggesting that BnA08g08960.1D2 may be diverged in the synthetic accessions.

It has been suggested that the genes for clubroot resistance in *B. napus* are located on the A genome[1], and clubroot resistance has been introgressed into *B. napus* from European fodder turnip[1,2]. However, the presence of the candidate orthologs of the clubroot resistance genes (*CRa* and *crr1a*) in the synthetics suggests that other sources of clubroot resistance may exist. The PAV behaviour of the clubroot orthologs is also consistent with that observed in Chinese cabbage[3] where gene presence and absence has been linked to resistance in a dosage-dependent manner, with some varieties exhibited partial resistance.

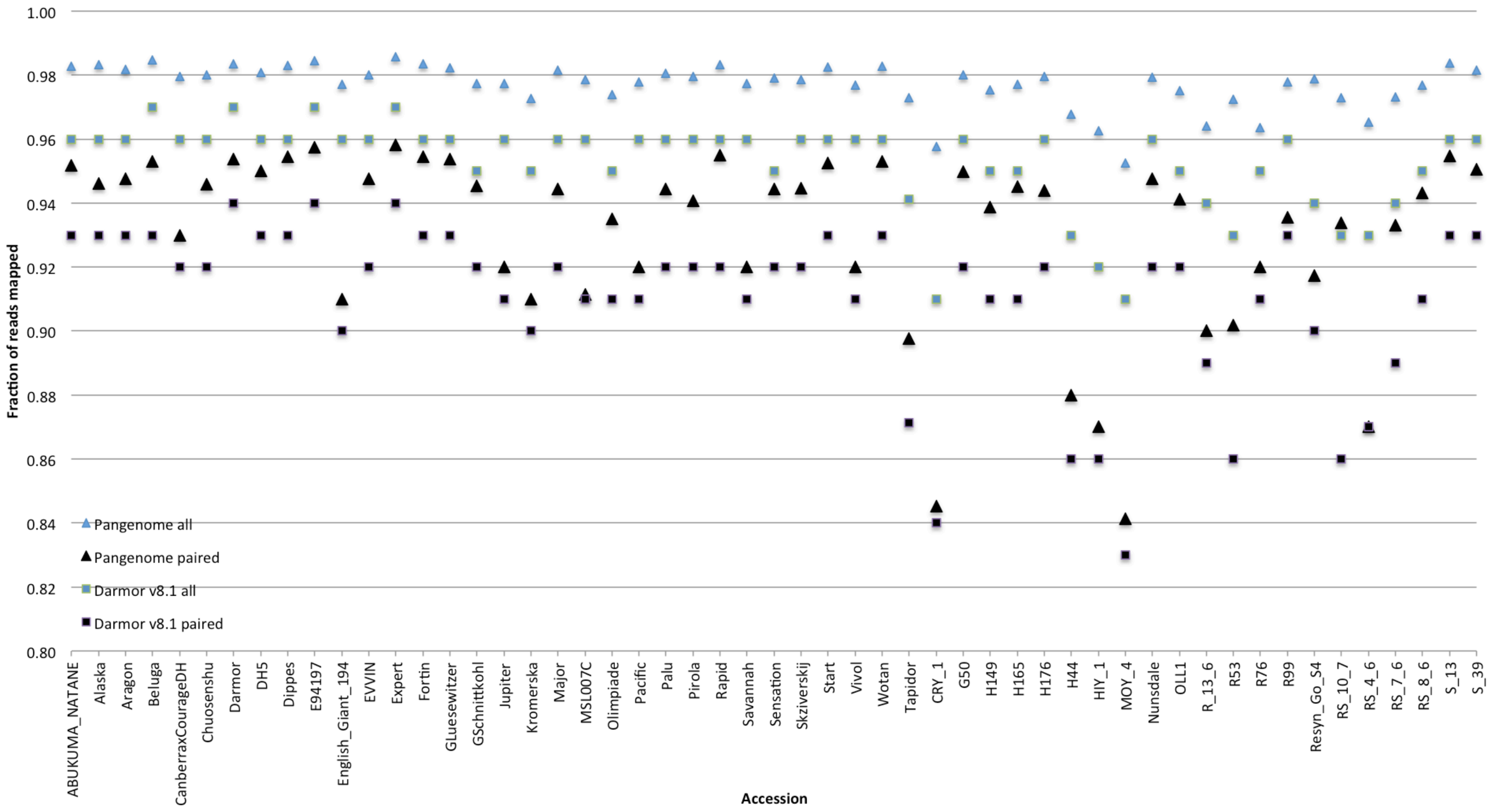

Supplementary Figure 6. For each of the accessions, the percentage of reads mapping to the Darmor-*bzh* v8.1 portion of the pangenome only and the entire pangenome was calculated. The results suggest that the newly assembled contigs provide mapping space for the previously unmapped reads. Reads mapping to mitochondrial, chloroplast and contamination sequences were excluded from the count.

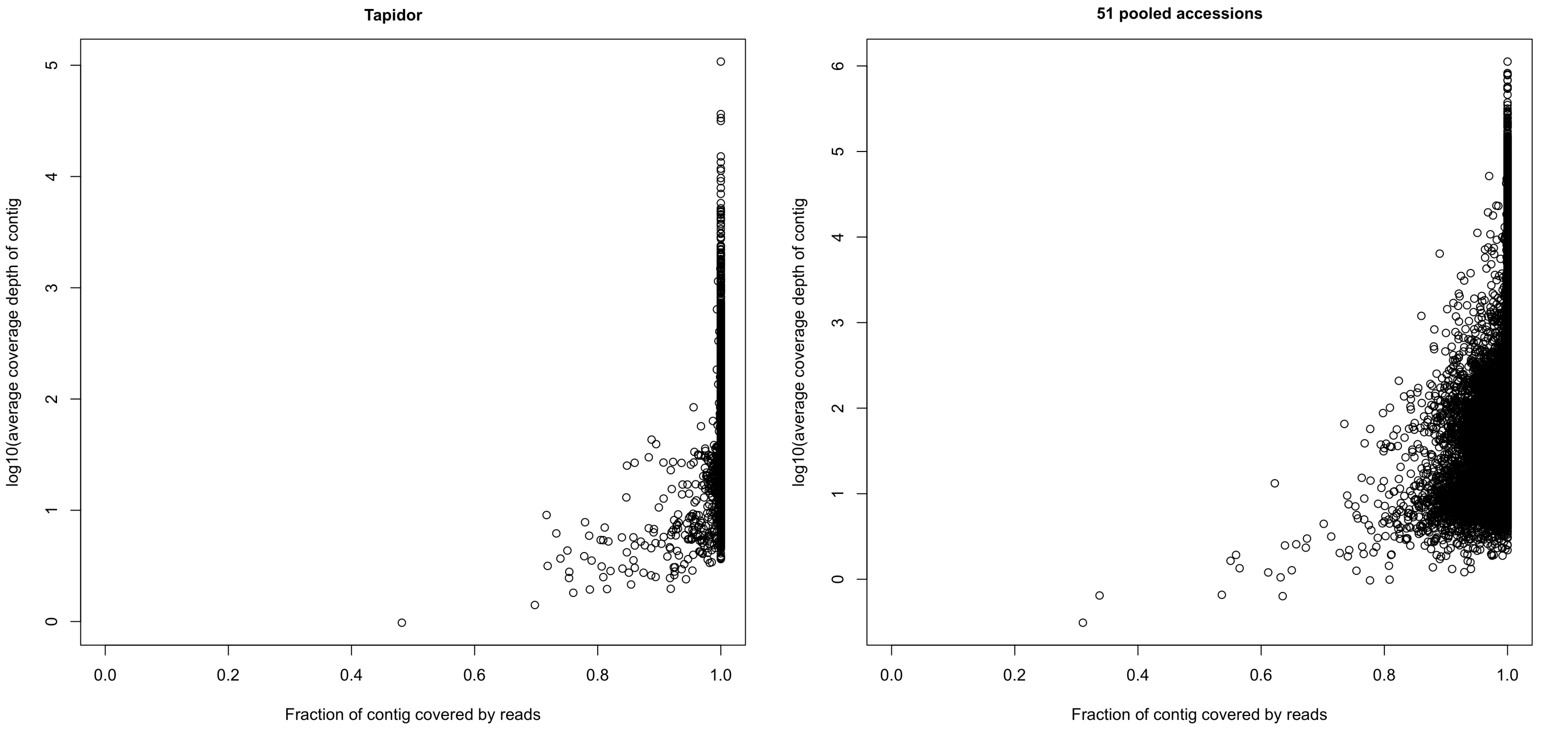

Supplementary Figure 7. Summary of coverage statistics when mapping reads from a given accession to the contigs contributed by this accession to the pangenome during iterative mapping and assembly. In this case, the Tapidor reads were mapped back to the newly assembled contigs stemming from the pangenome assembly. Similarly, reads from the 51 low coverage accessions were mapped to the contigs that originate from the assembly of their pooled unmapped reads. In both cases, the horizontal coverage, i.e., the fraction of contig that is covered by sequencing reads and the vertical coverage, i.e., the average coverage of reads mapped to the contig, were calculated for all the contigs.

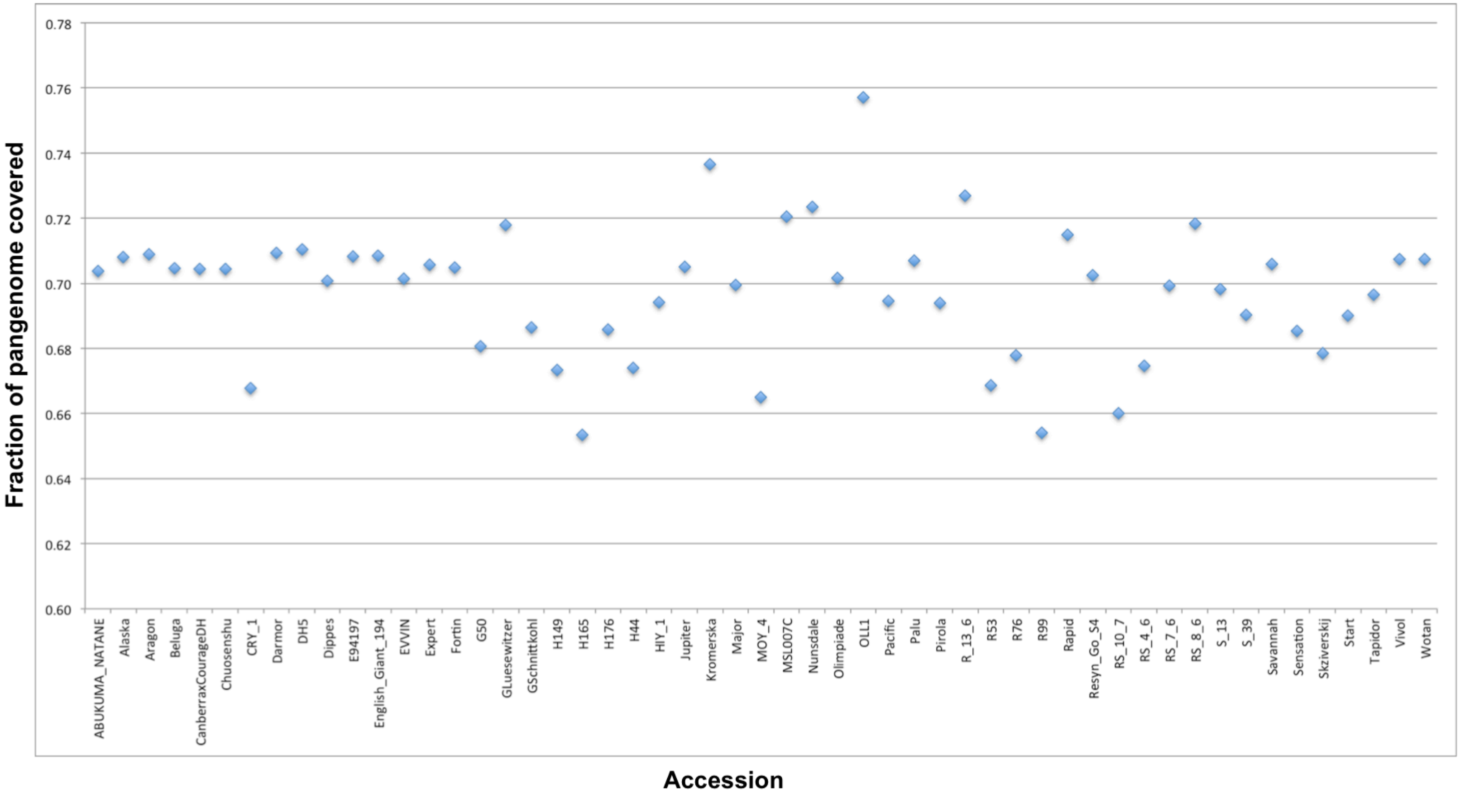

Supplementary Figure 8. The fraction of the pangenome which had reads mapping (coverage >=1) for each of the accessions.
