# Supplementary material for: Homoeologous exchange is a major cause of gene presence/absence variation in the amphidiploid Brassica napus
Source: Plant Biotechnol J. 2018 Jan 10;16(7):1265–74. doi: 10.1111/pbi.12867 (PMC5999312; doi:10.1111/pbi.12867)
Supplement: Supplementary file 2 — Table S1 Assembly statistics. Table S2 Number of genes annotated and used in the analysis. Table S3 BUSCO results to assess the completeness of the pangenome assembly. Table S5 Number of uniquely present and absent genes in (a) non‐synthetics and (b) synthetics. Table S6. Gene ontology (GO) enrichment of uniquely present genes. Table S7 GO enrichment of uniquely absent genes. Table S8 GO enrichment of variable genes for the biological process category. Table S9 Number of SNPs and SNP density across the pangenome. Table S10 Number of private SNPs in (a) non‐synthetics and (b) synthetics. Table S11 Properties of CDS SNPs in core and variable genomes. Table S12 GO enrichment of HE PAV genes. Table S13 Number of R‐genes absent in (a) non‐synthetics and (b) synthetics. Table S14 Number of acyl lipid metabolism genes absent (a) non‐synthetics and (b) synthetics. Table S15 Number of GSL metabolism genes absent in (a) non‐synthetics and (b) synthetics. Table S16 Reads used for pangenome assembly. Table S17 Pfam domains used to identify TE genes. Table S18 Domains used to identify R‐genes. The Coils database was used to identify CC domains. Table S19 Order of accession on Circos plot for (a) non‐synthetics and (b) synthetics. [file PBI-16-1265-s003.pdf]

Supplementary Table 1. Assembly statistics of the accessions used to build the pangenome.

**Accession**	**No. of contigs**	**Total length (bp)**	**Longest contig (bp)**	**N50**
Tapidor	20,382	35,208,066	122,068	5,866
51 lines pooled	173,375	158,682,692	41,759	1,162
Total newly assembled	193,757	193,890,758		
Darmor-*bzh* v8.1		850,288,203		
**Total**		1,044,178,961		

The accessions are listed in the order of addition to the pangenome. The reads from Tapidor (coverage ~ 80.1x) were used first. The reads from the 51 remaining accessions (excluding Darmor) were pooled prior to mapping and assembly due to low sequence coverage (<30x). Details about the reads used for assembly can be found in Supplementary Table 16.

Supplementary Table 2. Number of genes annotated and used in the analysis.

	**Number of genes used in the analysis**	**Number of complete genes**
Darmor-*bzh* v8.1	80,380	80,379
Newly assembled contigs	13,633	6,104
Pangenome	94,013	86,483

Two Darmor-*bzh* v8.1 genes, which had no Darmor-*bzh* reads aligned to them (BnaC03g33340.1D2 and BnaC06g05080.1D2), were removed from the final gene set. This resulted in 80,380 genes stemming from the Darmor-*bzh* portion of the pangenome assembly. The annotation of newly assembled contigs (>=1000 bp) resulted in 13,633 genes after removal of contaminants, TE genes, genes with an AED score of 1 and genes which were less than 100 bp long (in nucleotide space). The final gene set used in the analysis consisted of 94,013 genes.

Supplementary Table 3. BUSCO results to assess the completeness of the pangenome assembly.

	**Number of BUSCOS identified in the pangenome**	**Number of BUSCOS identified in Darmor-*bzh* v8.1**
Complete single-copy	202	201
Complete duplicated	1208	1206
Fragmented	10	9
Missing	20	24
**Total**	1440	1440

The number of BUSCOs identified in the pangenome assembly was compared with the results from the Darmor-*bzh* v8.1 assembly. 4 additional BUSCOs were identified in the newly assembled contigs. The total number of BUSCO groups searched was 1440.

Supplementary Table 4. Gene PAV with respect to the synthetic and non-synthetic accessions. The gene PAV matrix can be accessed from http://brassicagenome.net/downloads/BnapusPan/BnaPan.PAV.table.csv.gz. The accessions R99, Start and Skziverskij are included in the table but not used for further analysis due to low coverage (<10x). The Darmor-bzh reads used were from Bioproject PRJEB5841 (ERR457740, ERR457756 and ERR457797; total coverage ~16x). The Tapidor reads (paired-end only) were subsampled to ~16x.

Supplementary Table 5. Number of uniquely present and absent genes in (a) non-synthetics and (b) synthetics.

a

Accession name	No. of uniquely lost genes	No. of uniquely present genes
Abukuma Natane	59	0
Alaska	7	0
Aragon	12	0
Beluga	15	0
CanberraxCourageDH	13	0
Darmor-*bzh*	13	0
DH5	2	0
Dippes	17	0
E94197	7	0
EVVIN	59	11
Expert	8	1
GLuesewitzer	11	6
Jupiter	24	0
Kromerska	14	4
Major	30	0
MSL007C	5	0
Olimpiade	33	0
Pacific	104	0
Pirola	23	0
Rapid	11	0
Savannah	21	1
Tapidor	46	4
Vivol	9	0
Wotan	10	0
English_Giant_194	96	2
Nunsdale	25	2
Palu	68	1
Fortin	14	0
Sensation	333	16
Chuosenshu	120	3
Gruner Schnittkohl	318	0
**Average**	**49**	**2**

b

Accession name	No. of uniquely lost genes	No. of uniquely present genes
CRY_1	521	114
G50	579	2
H149	434	9
H165	772	10
H176	125	0
H44	491	0
HIY_1	93	9
MOY_4	394	183
OLL1	1	5
R_13_6	50	11
R53	806	6
R76	946	49
Resyn-Go S4	56	0
RS_10_7	905	4
RS_4_6	1052	5
RS_7_6	44	1
RS_8_6	89	4
S_13	25	0
S_39	877	1
**Average**	**435**	**22**

On average, each accession contained 84,192 genes. The synthetic accessions demonstrated the greatest variation, with an average of 22 uniquely present and 435 uniquely absent genes, compared to the non-synthetic accessions, which had an average of 2 uniquely present and 49 uniquely absent genes. Within the non-synthetic accessions, 1, 2, 2, and 8 uniquely present genes were identified on average, and 23, 219, 63 and 174 uniquely absent genes were identified on average in the oilseed, vegetable, fodder types, and swedes, respectively.

Supplementary Table 6. Gene ontology (GO) enrichment of uniquely present genes.

Type/Morphotype	GO.ID	Term	p-value
**Synthetics**	GO:0006468	protein phosphorylation	4.90E-05
	GO:0006952	defense response	0.00048
	GO:0009653	anatomical structure morphogenesis	0.00057
	GO:0035556	intracellular signal transduction	0.00126
	GO:0009626	plant-type hypersensitive response	0.00221
**Oilseeds**	GO:0044237	cellular metabolic process	2.50E-06
	GO:0008152	metabolic process	0.00013
	GO:1901564	organonitrogen compound metabolic proces	0.00055
	GO:0019752	carboxylic acid metabolic process	0.0038
	GO:0044710	single-organism metabolic process	0.00494
**Swedes**	GO:0070981	L-asparagine biosynthetic process	0.00086
	GO:0006529	asparagine biosynthetic process	0.00129
	GO:0006541	glutamine metabolic process	0.01305
	GO:0010951	negative regulation of endopeptidase activity	0.01577
**Fodder**	GO:0009094	L-phenylalanine biosynthetic process	0.00059
	GO:0010048	vernalization response	0.00595
	GO:0043481	anthocyanin accumulation in tissues	0.00852
	GO:0048440	carpel development	0.01493
**Vegetable**	NA	NA	NA

Top 5 most significant GO terms in the biological process category associated with uniquely present genes in each type/morphotype. No significant terms were found for the uniquely present genes in the vegetable types.

Supplementary Table 7. GO enrichment of uniquely absent genes.

Type/Morphotype	GO.ID	Term	p.value
**Synthetics**	GO:0006355	regulation of transcription	2.30E-06
	GO:0009873	ethylene-activated signaling pathway	6.60E-05
	GO:0009611	response to wounding	0.00013
	GO:0016567	protein ubiquitination	0.00022
	GO:0010200	response to chitin	0.00025
**Oilseeds**	GO:0042180	cellular ketone metabolic process	0.0045
	GO:0046719	regulation by virus of viral protein levels in host cell	0.0046
	GO:0030334	regulation of cell migration	0.0046
	GO:0006654	phosphatidic acid biosynthetic process	0.0046
	GO:0050764	regulation of phagocytosis	0.0046
**Swedes**	GO:0010044	response to aluminum ion	5.90E-07
	GO:0006011	UDP-glucose metabolic process	0.00018
	GO:0009910	negative regulation of flower development	0.0006
	GO:0010264	myo-inositol hexakisphosphate biosynthetic process	0.00094
	GO:0007062	sister chromatid cohesion	0.00162
**Fodder**	GO:0006436	tryptophanyl-tRNA aminoacylation	9.10E-05
	GO:0009739	response to gibberellin	0.00018
	GO:0046274	lignin catabolic process	0.0002
	GO:0010439	regulation of glucosinolate biosynthetic process	0.00113
	GO:0009911	positive regulation of flower development	0.00286
**Vegetable**	GO:0006655	phosphatidylglycerol biosynthetic process	0.0016
	GO:0032259	methylation	0.0034
	GO:1902047	polyamine transmembrane transport	0.0035
	GO:0048507	meristem development	0.0041
	GO:0043489	RNA stabilization	0.0063

Top 5 most significant GO terms in the biological process category associated with uniquely absent genes in each type/morphotype.

Supplementary Table 8. GO enrichment of variable genes for the biological process category.

GO.ID	Term	p.value
GO:0009626	plant-type hypersensitive response	3.4E-20
GO:0051607	defense response to virus	9.5E-13
GO:0090304	nucleic acid metabolic process	3.8E-10
GO:0045088	regulation of innate immune response	7.4E-10
GO:0002764	immune response-regulating signaling pathway	0.00000038
GO:0016106	sesquiterpenoid biosynthetic process	0.00000039
GO:0051762	sesquiterpene biosynthetic process	0.00000076
GO:0000966	RNA 5'-end processing	0.00000085
GO:0006412	translation	0.0000012
GO:0010204	defense response signaling pathway, resistance gene-independent	0.0000014
GO:0006171	cAMP biosynthetic process	0.000002
GO:0009816	defense response to bacterium, incompatible	0.0000025
GO:0044699	single-organism process	0.0000031
GO:0002230	positive regulation of defense response to virus by host	0.000017
GO:0006517	protein deglycosylation	0.000018
GO:0044260	cellular macromolecule metabolic process	0.00019
GO:2000071	regulation of defense response by callose deposition	0.00021
GO:0046741	transport of virus in host, tissue to tissue	0.00038
GO:0006085	acetyl-CoA biosynthetic process	0.00038
GO:0006265	DNA topological change	0.0006
GO:0009870	defense response signaling pathway, resistance gene-dependent	0.00074
GO:0051050	positive regulation of transport	0.00076
GO:0015718	monocarboxylic acid transport	0.00106
GO:0031323	regulation of cellular metabolic process	0.00124
GO:0009615	response to virus	0.00148
GO:0006182	cGMP biosynthetic process	0.0022
GO:0042138	meiotic DNA double-strand break formation	0.00235

Supplementary Table 9. Number of SNPs and SNP density across the pangenome.

**Chromosome**	**Chromosome size (bp)**	**No. of SNPs**	**SNP density (per kbp)**
chrA01	31,163,448	175,524	5.63
chrA02	31,341,975	159,976	5.10
chrA03	39,488,916	217,598	5.51
chrA04	23,309,376	126,400	5.42
chrA05	28,601,535	165,790	5.80
chrA06	31,899,768	173,569	5.44
chrA07	28,903,433	165,355	5.72
chrA08	21,742,160	112,067	5.15
chrA09	46,718,919	232,400	4.97
chrA10	19,955,110	102,619	5.14
chrC01	47,954,325	247,209	5.16
chrC02	58,662,066	325,324	5.55
chrC03	71,849,896	315,464	4.39
chrC04	61,044,540	273,593	4.48
chrC05	52,720,298	245,664	4.66
chrC06	44,607,072	244,755	5.49
chrC07	52,496,477	305,767	5.82
chrC08	46,289,800	245,513	5.30
chrC09	60,205,297	163,737	2.72
Unplaced	51,333,792	163,459	3.18
Tapidor_contigs	35,208,066	125,255	3.22
51_lines_contigs	158,682,692	588,691	3.71
**Total**	**1,044,178,961**	**4,875,729**	**4.67**

Supplementary Table 10. Number of private SNPs in (a) non-synthetics and (b) synthetics.

a

**Accession**	**Number of private SNPs**
Abukuma Natane	2716
Alaska	2687
Aragon	6248
Beluga	1847
CanberraxCourageDH	3855
Darmor-*bzh*	225
DH5	4015
Dippes	3102
E94197	2096
EVVIN	29009
Expert	2780
Gross Luesewitzer	3758
Jupiter	8353
Kromerska	33658
Major	3813
MSL007C	8694
Olimpiade	11262
Pacific	2343
Pirola	1589
Rapid	2801
Savannah	8211
Skziverskij	3649
Start	780
Tapidor	22818
Vivol	5579
Wotan	4462
Chuosenshu	16574
Gruner Schnittkohl	15442
Fortin	1648
Sensation	29315
English_Giant_194	13676
Palu	12729
Nunsdale	7373

b

**Accession**	**Number of private SNPs**
CRY_1	302252
G50	17936
H149	59877
H165	44493
H176	7296
H44	61461
HIY_1	67404
MOY_4	238989
OLL1	13491
R_13_6	61837
R53	57586
R76	159982
R99	3410
Resyn_Go_S4	2667
RS_10_7	91691
RS_4_6	61951
RS_7_6	56112
RS_8_6	46066
S_13	4593
S_39	24170

Supplementary Table 11. Properties of CDS SNPs in core and variable genomes.

	**core genome**	**variable genome**
Number of CDS SNPs	264,562	126,595
Number of synonymous SNPs	151,592	61,588
Number of non-synonymous SNPs	111,631	63,582
Number of nonsense SNPs	1,339	1,425
Average exon length (bp)	1130.55	790.40
Total exon length (bp)	66215345	29273035
SNP density (SNPs/Kbp)	3.99	4.51

Supplementary Table 12. GO enrichment of HE PAV genes. Significant GO terms in the biological process category are shown. for (a) A01 to C01, (b) A02 to C02, (c) A04 to C04, (d) A07 to C06, (e) A09 to C08, (f) A09 to C09, (g) A10 to C09, (h) C01 to A01, (i) C02 to A02, (k) C03 to A03, (l) C04 to A04, (m) C04 to A05, (n) C05 to A05, (o) C06 to A07, (p) C08 to C09, (q) C09 to A09 and (r) C09 to A10.

a

**GO.ID**	**Term**	**p.value**
GO:0015795	sorbitol transport	3.3E-05
GO:0015797	mannitol transport	3.3E-05
GO:0015752	D-ribose transport	3.3E-05
GO:0015753	D-xylose transport	3.3E-05
GO:0015793	glycerol transport	8.9E-05
GO:0015757	galactose transport	8.9E-05
GO:0015798	myo-inositol transport	0.00029
GO:1904659	glucose transmembrane transport	0.00067
GO:0015992	proton transport	0.00072
GO:0006979	response to oxidative stress	0.00118
GO:0016572	histone phosphorylation	0.00189
GO:0031348	negative regulation of defense response	0.00197
GO:0048467	gynoecium development	0.0038
GO:0042538	hyperosmotic salinity response	0.00387
GO:0008295	spermidine biosynthetic process	0.00499
GO:0090342	regulation of cell aging	0.00559
GO:0009052	pentose-phosphate shunt, non-oxidative b...	0.00559
GO:0010582	floral meristem determinacy	0.00575
GO:0019432	triglyceride biosynthetic process	0.00629
GO:0071492	cellular response to UV-A	0.00681
GO:0048235	pollen sperm cell differentiation	0.00687
GO:0018344	protein geranylgeranylation	0.00747
GO:0009446	putrescine biosynthetic process	0.00815
GO:0006527	arginine catabolic process	0.00885
GO:0016579	protein deubiquitination	0.0096
GO:0009759	indole glucosinolate biosynthetic proces...	0.01034
GO:0009625	response to insect	0.01079
GO:1901642	nucleoside transmembrane transport	0.01109
GO:0009260	ribonucleotide biosynthetic process	0.0119
GO:0008283	cell proliferation	0.01229

b

GO.ID	Term	p.value
GO:0002221	pattern recognition receptor signaling p...	0.0018
GO:0006289	nucleotide-excision repair	0.002
GO:0031407	oxylipin metabolic process	0.0021
GO:0009733	response to auxin	0.0029
GO:0006979	response to oxidative stress	0.0044
GO:0046686	response to cadmium ion	0.0049
GO:0060774	auxin mediated signaling pathway involve...	0.0053
GO:0006414	translational elongation	0.0057
GO:0060772	leaf phyllotactic patterning	0.0077
GO:0009664	plant-type cell wall organization	0.0079
GO:0043481	anthocyanin accumulation in tissues in r...	0.0082
GO:0010080	regulation of floral meristem growth	0.0091
GO:0015706	nitrate transport	0.0095
GO:0010167	response to nitrate	0.0098
GO:0000712	resolution of meiotic recombination inte...	0.0105
GO:0010492	maintenance of shoot apical meristem ide...	0.0112
GO:0007143	female meiotic division	0.0113
GO:0009932	cell tip growth	0.0115
GO:0009723	response to ethylene	0.0155
GO:0006094	gluconeogenesis	0.0164
GO:0080155	regulation of double fertilization formi...	0.0164
GO:0000462	maturation of SSU-rRNA from tricistronic...	0.0183
GO:2000904	regulation of starch metabolic process	0.0192
GO:0000271	polysaccharide biosynthetic process	0.0194
GO:0048511	rhythmic process	0.0212
GO:0071249	cellular response to nitrate	0.0212
GO:0046688	response to copper ion	0.0223
GO:0051187	cofactor catabolic process	0.0228
GO:0042545	cell wall modification	0.0271
GO:0015866	ADP transport	0.0277

c

**GO.ID**	**Term**	**p.value**
GO:0010422	regulation of brassinosteroid biosynthet...	0.0001
GO:0030639	polyketide biosynthetic process	0.00027
GO:0009298	GDP-mannose biosynthetic process	0.00149
GO:0080110	sporopollenin biosynthetic process	0.00166
GO:0006633	fatty acid biosynthetic process	0.00358
GO:0009888	tissue development	0.00399
GO:0010218	response to far red light	0.00471
GO:0006614	SRP-dependent cotranslational protein ta...	0.00523
GO:0006778	porphyrin-containing compound metabolic ...	0.00602
GO:0007623	circadian rhythm	0.00648
GO:0019853	L-ascorbic acid biosynthetic process	0.00694
GO:0006671	phytosphingosine metabolic process	0.00914
GO:0019877	diaminopimelate biosynthetic process	0.01066
GO:0048034	heme O biosynthetic process	0.01066
GO:0051457	maintenance of protein location in nucle...	0.01066
GO:0002679	respiratory burst involved in defense re...	0.01111
GO:0009887	organ morphogenesis	0.01117
GO:0032264	IMP salvage	0.01217
GO:0009585	red, far-red light phototransduction	0.01248
GO:0019307	mannose biosynthetic process	0.01369
GO:0040007	growth	0.01558
GO:0048653	anther development	0.01758
GO:0060359	response to ammonium ion	0.02121
GO:0019762	glucosinolate catabolic process	0.02127
GO:0009647	skotomorphogenesis	0.02271
GO:0046168	glycerol-3-phosphate catabolic process	0.02569
GO:0001736	establishment of planar polarity	0.02569
GO:0010311	lateral root formation	0.02669
GO:0006661	phosphatidylinositol biosynthetic proces...	0.02697
GO:0006626	protein targeting to mitochondrion	0.03268

d

GO.ID	Term	p.value
GO:0016540	protein autoprocessing	0.00017
GO:0019563	glycerol catabolic process	0.00039
GO:0009805	coumarin biosynthetic process	0.00104
GO:1901684	arsenate ion transmembrane transport	0.00116
GO:0035435	phosphate ion transmembrane transport	0.00215
GO:0009641	shade avoidance	0.00385
GO:0098655	cation transmembrane transport	0.00498
GO:0043456	regulation of pentose-phosphate shunt	0.00829
GO:0043068	positive regulation of programmed cell d...	0.0083
GO:0090057	root radial pattern formation	0.00967
GO:1901997	negative regulation of indoleacetic acid...	0.01104
GO:0090359	negative regulation of abscisic acid bio...	0.01104
GO:0006127	glycerophosphate shuttle	0.01104
GO:0010270	photosystem II oxygen evolving complex a...	0.01104
GO:0045117	azole transport	0.01241
GO:0009744	response to sucrose	0.01363
GO:0015822	ornithine transport	0.01378
GO:0010248	establishment or maintenance of transmem...	0.01378
GO:0015824	proline transport	0.01545
GO:0048767	root hair elongation	0.01568
GO:0046167	glycerol-3-phosphate biosynthetic proces...	0.01652
GO:0045723	positive regulation of fatty acid biosyn...	0.01652
GO:0071497	cellular response to freezing	0.01652
GO:0002237	response to molecule of bacterial origin	0.01725
GO:1901959	positive regulation of cutin biosyntheti...	0.01788
GO:0043091	L-arginine import	0.01788
GO:0042754	negative regulation of circadian rhythm	0.01788
GO:0010366	negative regulation of ethylene biosynth...	0.01924
GO:0010236	plastoquinone biosynthetic process	0.01924
GO:0006541	glutamine metabolic process	0.01933

e

GO.ID	Term	p.value
GO:0071249	cellular response to nitrate	0.000004
GO:0046256	2,4,6-trinitrotoluene catabolic process	0.000019
GO:0009854	oxidative photosynthetic carbon pathway	0.00066
GO:0006571	tyrosine biosynthetic process	0.00179
GO:0006623	protein targeting to vacuole	0.00244
GO:0019430	removal of superoxide radicals	0.00252
GO:0048527	lateral root development	0.00317
GO:0055114	oxidation-reduction process	0.00654
GO:0010152	pollen maturation	0.00732
GO:0036065	fucosylation	0.00794
GO:0010501	RNA secondary structure unwinding	0.01209
GO:0043153	entrainment of circadian clock by photop...	0.01351
GO:0009830	cell wall modification involved in absci...	0.01487
GO:0010260	organ senescence	0.01576
GO:0006474	N-terminal protein amino acid acetylatio...	0.01619
GO:0015770	sucrose transport	0.017
GO:0090057	root radial pattern formation	0.01886
GO:0018022	peptidyl-lysine methylation	0.01897
GO:0006109	regulation of carbohydrate metabolic pro...	0.02346
GO:1990570	GDP-mannose transmembrane transport	0.02419
GO:1901601	strigolactone biosynthetic process	0.02419
GO:0080037	negative regulation of cytokinin-activat...	0.02419
GO:0042276	error-prone translesion synthesis	0.02684
GO:0080178	5-carbamoylmethyluridine metabolic proce...	0.02684
GO:0010248	establishment or maintenance of transmem...	0.02684
GO:0010163	high-affinity potassium ion import	0.02948
GO:0031539	positive regulation of anthocyanin metab...	0.03212
GO:2000032	regulation of secondary shoot formation	0.03212
GO:0016311	dephosphorylation	0.03248
GO:0080187	floral organ senescence	0.03475

f

GO.ID	Term	p.value
GO:0010541	acropetal auxin transport	0.0000019
GO:0010315	auxin efflux	0.000016
GO:0010540	basipetal auxin transport	0.000028
GO:0043481	anthocyanin accumulation in tissues in r...	0.00044
GO:0046740	transport of virus in host, cell to cell	0.00102
GO:0010160	formation of organ boundary	0.00121
GO:0042964	thioredoxin biosynthetic process	0.00144
GO:0009932	cell tip growth	0.00173
GO:0010492	maintenance of shoot apical meristem ide...	0.00178
GO:0031146	SCF-dependent proteasomal ubiquitin-depe...	0.00182
GO:0009958	positive gravitropism	0.00227
GO:0009306	protein secretion	0.00248
GO:0009740	gibberellic acid mediated signaling path...	0.0028
GO:0010071	root meristem specification	0.00291
GO:0080086	stamen filament development	0.00336
GO:0048443	stamen development	0.00348
GO:0098655	cation transmembrane transport	0.00463
GO:0043086	negative regulation of catalytic activit...	0.00571
GO:0080167	response to karrikin	0.0059
GO:0009846	pollen germination	0.00636
GO:0045454	cell redox homeostasis	0.00642
GO:0010048	vernalization response	0.00825
GO:0009825	multidimensional cell growth	0.00834
GO:0006207	'de novo' pyrimidine nucleobase biosynth...	0.00973
GO:0010078	maintenance of root meristem identity	0.01088
GO:0000271	polysaccharide biosynthetic process	0.01364
GO:0019745	pentacyclic triterpenoid biosynthetic pr...	0.01388
GO:1903070	negative regulation of ER-associated ubi...	0.01471
GO:0015789	UDP-N-acetylgalactosamine transport	0.01471
GO:1990569	UDP-N-acetylglucosamine transmembrane tr...	0.01471

g

GO.ID	Term	p.value
GO:0010089	xylem development	0.000047
GO:0090435	protein localization to nuclear envelope	0.000083
GO:0046685	response to arsenic-containing substance	0.0019
GO:0016132	brassinosteroid biosynthetic process	0.0045
GO:0050983	deoxyhypusine biosynthetic process from ...	0.0056
GO:0045839	negative regulation of mitotic nuclear d...	0.0067
GO:2000603	regulation of secondary growth	0.0068
GO:0035235	ionotropic glutamate receptor signaling ...	0.0077
GO:0019424	sulfide oxidation, using siroheme sulfit...	0.009
GO:0051291	protein heterooligomerization	0.009
GO:0006617	SRP-dependent cotranslational protein ta...	0.009
GO:0048283	indeterminate inflorescence morphogenesi...	0.009
GO:0019419	sulfate reduction	0.0101
GO:1901430	positive regulation of syringal lignin b...	0.0124
GO:0071806	protein transmembrane transport	0.0132
GO:0032509	endosome transport via multivesicular bo...	0.0146
GO:0051604	protein maturation	0.0149
GO:0015012	heparan sulfate proteoglycan biosyntheti...	0.0168
GO:0019395	fatty acid oxidation	0.0188
GO:0009102	biotin biosynthetic process	0.019
GO:0006420	arginyl-tRNA aminoacylation	0.019
GO:0048657	anther wall tapetum cell differentiation	0.019
GO:0048868	pollen tube development	0.0198
GO:0019433	triglyceride catabolic process	0.0201
GO:0009684	indoleacetic acid biosynthetic process	0.0208
GO:0045038	protein import into chloroplast thylakoi...	0.0224
GO:1902047	polyamine transmembrane transport	0.0235
GO:0045324	late endosome to vacuole transport	0.0246
GO:0030418	nicotianamine biosynthetic process	0.0246
GO:0008333	endosome to lysosome transport	0.0246

h

GO.ID	Term	p.value
GO:0009698	phenylpropanoid metabolic process	0.00017
GO:0006545	glycine biosynthetic process	0.00268
GO:0009113	purine nucleobase biosynthetic process	0.00339
GO:0000302	response to reactive oxygen species	0.00447
GO:0009116	nucleoside metabolic process	0.00499
GO:0006527	arginine catabolic process	0.00533
GO:0045893	positive regulation of transcription, DN...	0.00622
GO:0007188	adenylate cyclase-modulating G-protein c...	0.00881
GO:0055114	oxidation-reduction process	0.00921
GO:0042744	hydrogen peroxide catabolic process	0.01059
GO:0009733	response to auxin	0.01245
GO:0006301	postreplication repair	0.01261
GO:0015689	molybdate ion transport	0.01303
GO:0045962	positive regulation of development, hete...	0.01303
GO:0006302	double-strand break repair	0.01321
GO:0009446	putrescine biosynthetic process	0.01419
GO:0009611	response to wounding	0.01584
GO:0042546	cell wall biogenesis	0.0162
GO:0048235	pollen sperm cell differentiation	0.01828
GO:2000067	regulation of root morphogenesis	0.01929
GO:0006598	polyamine catabolic process	0.0234
GO:0016192	vesicle-mediated transport	0.02397
GO:0009817	defense response to fungus, incompatible...	0.02473
GO:0030036	actin cytoskeleton organization	0.0281
GO:0016572	histone phosphorylation	0.02958
GO:0080125	multicellular structure septum developme...	0.03126
GO:0035670	plant-type ovary development	0.03128
GO:0042538	hyperosmotic salinity response	0.03152
GO:0098656	anion transmembrane transport	0.03233
GO:0006544	glycine metabolic process	0.03243

i

GO.ID	Term	p.value
GO:0009733	response to auxin	0.0002
GO:0034635	glutathione transport	0.00025
GO:0007143	female meiotic division	0.00035
GO:0080145	cysteine homeostasis	0.00127
GO:0002221	pattern recognition receptor signaling p...	0.00127
GO:1900384	regulation of flavonol biosynthetic proc...	0.00179
GO:0006694	steroid biosynthetic process	0.00233
GO:0060774	auxin mediated signaling pathway involve...	0.00386
GO:0060772	leaf phyllotactic patterning	0.00564
GO:0042761	very long-chain fatty acid biosynthetic ...	0.00603
GO:0006075	(1->3)-beta-D-glucan biosynthetic proces...	0.00697
GO:0000712	resolution of meiotic recombination inte...	0.00772
GO:0019048	modulation by virus of host morphology o...	0.00829
GO:0007140	male meiosis	0.00936
GO:0002229	defense response to oomycetes	0.01025
GO:0009723	response to ethylene	0.01182
GO:0002237	response to molecule of bacterial origin	0.01294
GO:0046686	response to cadmium ion	0.01312
GO:0016050	vesicle organization	0.01455
GO:0071333	cellular response to glucose stimulus	0.01568
GO:0010440	stomatal lineage progression	0.01866
GO:0090174	organelle membrane fusion	0.01937
GO:0010228	vegetative to reproductive phase transit...	0.02041
GO:0015866	ADP transport	0.02055
GO:0009718	anthocyanin-containing compound biosynth...	0.02145
GO:0009873	ethylene-activated signaling pathway	0.02227
GO:0010198	synergid death	0.02421
GO:0051085	chaperone mediated protein folding requi...	0.02421
GO:0071433	cell wall repair	0.02421
GO:0034976	response to endoplasmic reticulum stress	0.02636

j

GO.ID	Term	p.value
GO:0030091	protein repair	0.000016
GO:0006120	mitochondrial electron transport, NADH t...	0.000068
GO:0042138	meiotic DNA double-strand break formatio...	0.00028
GO:0034599	cellular response to oxidative stress	0.0013
GO:0043100	pyrimidine nucleobase salvage	0.0014
GO:0006557	S-adenosylmethioninamine biosynthetic pr...	0.00161
GO:0016049	cell growth	0.00178
GO:0010582	floral meristem determinacy	0.0019
GO:0007062	sister chromatid cohesion	0.00194
GO:0019433	triglyceride catabolic process	0.00233
GO:0048193	Golgi vesicle transport	0.00317
GO:0006734	NADH metabolic process	0.00347
GO:0009051	pentose-phosphate shunt, oxidative branc...	0.0038
GO:0006597	spermine biosynthetic process	0.00413
GO:0043086	negative regulation of catalytic activit...	0.0063
GO:0007131	reciprocal meiotic recombination	0.00671
GO:0030244	cellulose biosynthetic process	0.00697
GO:0007129	synapsis	0.00788
GO:0000902	cell morphogenesis	0.00836
GO:0034508	centromere complex assembly	0.00865
GO:0006598	polyamine catabolic process	0.00916
GO:0006739	NADP metabolic process	0.01028
GO:0009908	flower development	0.01134
GO:0010089	xylem development	0.0129
GO:0008215	spermine metabolic process	0.01575
GO:0010044	response to aluminum ion	0.01844
GO:0010335	response to non-ionic osmotic stress	0.01979
GO:0006982	response to lipid hydroperoxide	0.01979
GO:0008295	spermidine biosynthetic process	0.02263
GO:0015986	ATP synthesis coupled proton transport	0.02332

k

GO.ID	Term	p.value
GO:2000025	regulation of leaf formation	0.00014
GO:0048653	anther development	0.0013
GO:0010047	fruit dehiscence	0.00216
GO:0080026	response to indolebutyric acid	0.00226
GO:0009901	anther dehiscence	0.00363
GO:0006778	porphyrin-containing compound metabolic ...	0.00631
GO:0006032	chitin catabolic process	0.00781
GO:0018920	glyphosate metabolic process	0.00948
GO:0019877	diaminopimelate biosynthetic process	0.01106
GO:0048034	heme O biosynthetic process	0.01106
GO:0043968	histone H2A acetylation	0.01106
GO:0002679	respiratory burst involved in defense re...	0.01257
GO:0043987	histone H3-S10 phosphorylation	0.01263
GO:0032259	methylation	0.01396
GO:0016998	cell wall macromolecule catabolic proces...	0.01408
GO:0019307	mannose biosynthetic process	0.01419
GO:0071454	cellular response to anoxia	0.01888
GO:0080153	negative regulation of reductive pentose...	0.01888
GO:0010227	floral organ abscission	0.01996
GO:0032465	regulation of cytokinesis	0.02355
GO:0009647	skotomorphogenesis	0.02355
GO:0071806	protein transmembrane transport	0.02574
GO:0006537	glutamate biosynthetic process	0.02819
GO:0010254	nectary development	0.02973
GO:0006301	postreplication repair	0.03115
GO:0006909	phagocytosis	0.03127
GO:0015798	myo-inositol transport	0.03434
GO:0055114	oxidation-reduction process	0.0366
GO:0006811	ion transport	0.0384
GO:0010434	bract formation	0.03894

l

GO.ID	Term	p.value
GO:0043086	negative regulation of catalytic activit...	0.00069
GO:0006778	porphyrin-containing compound metabolic ...	0.00261
GO:0045490	pectin catabolic process	0.00274
GO:0043132	NAD transport	0.00334
GO:0010047	fruit dehiscence	0.00796
GO:0080026	response to indolebutyric acid	0.00815
GO:0009901	anther dehiscence	0.01036
GO:0006535	cysteine biosynthetic process from serin...	0.01036
GO:0000027	ribosomal large subunit assembly	0.01293
GO:0009965	leaf morphogenesis	0.01341
GO:0016925	protein sumoylation	0.01495
GO:0010214	seed coat development	0.0268
GO:0009106	lipoate metabolic process	0.03655
GO:0018108	peptidyl-tyrosine phosphorylation	0.03673
GO:0042545	cell wall modification	0.04214
GO:0009834	plant-type secondary cell wall biogenesi...	0.04549
GO:0006546	glycine catabolic process	0.04762
GO:0045893	positive regulation of transcription, DN...	0.04794
GO:0009809	lignin biosynthetic process	0.0494
GO:0048653	anther development	0.05032
GO:0006636	unsaturated fatty acid biosynthetic proc...	0.06332
GO:0009955	adaxial/abaxial pattern specification	0.07463
GO:0009073	aromatic amino acid family biosynthetic ...	0.07739
GO:0006766	vitamin metabolic process	0.07843
GO:0009684	indoleacetic acid biosynthetic process	0.09161
GO:0045454	cell redox homeostasis	0.09823
GO:0015995	chlorophyll biosynthetic process	0.10227
GO:0009695	jasmonic acid biosynthetic process	0.10982
GO:0009220	pyrimidine ribonucleotide biosynthetic p...	0.12456
GO:0009734	auxin-activated signaling pathway	0.12752

m

GO.ID	Term	p.value
GO:0072660	maintenance of protein location in plasm...	0.0000093
GO:0051245	negative regulation of cellular defense ...	0.000096
GO:0072661	protein targeting to plasma membrane	0.00012
GO:0010148	transpiration	0.00041
GO:0030036	actin cytoskeleton organization	0.00406
GO:0042981	regulation of apoptotic process	0.0048
GO:0001510	RNA methylation	0.00481
GO:0051091	positive regulation of sequence-specific...	0.0073
GO:0007346	regulation of mitotic cell cycle	0.0077
GO:1900057	positive regulation of leaf senescence	0.00773
GO:0016132	brassinosteroid biosynthetic process	0.00833
GO:0006457	protein folding	0.00948
GO:0051607	defense response to virus	0.01154
GO:0043574	peroxisomal transport	0.01157
GO:0061025	membrane fusion	0.01213
GO:0051403	stress-activated MAPK cascade	0.01271
GO:0042538	hyperosmotic salinity response	0.01511
GO:0010193	response to ozone	0.01524
GO:0000028	ribosomal small subunit assembly	0.01746
GO:0009723	response to ethylene	0.01917
GO:0050832	defense response to fungus	0.02137
GO:0006446	regulation of translational initiation	0.02163
GO:0045995	regulation of embryonic development	0.02214
GO:0046396	D-galacturonate metabolic process	0.02302
GO:0010213	non-photoreactive DNA repair	0.02302
GO:0001561	fatty acid alpha-oxidation	0.02302
GO:0043069	negative regulation of programmed cell d...	0.0244
GO:0000398	mRNA splicing, via spliceosome	0.02563
GO:0015801	aromatic amino acid transport	0.02681
GO:0071479	cellular response to ionizing radiation	0.02681

n

GO.ID	Term	p.value
GO:0035435	phosphate ion transmembrane transport	0.000011
GO:1901684	arsenate ion transmembrane transport	0.00045
GO:0006891	intra-Golgi vesicle-mediated transport	0.00312
GO:0009873	ethylene-activated signaling pathway	0.0057
GO:0010270	photosystem II oxygen evolving complex a...	0.00684
GO:0090359	negative regulation of abscisic acid bio...	0.00684
GO:0080037	negative regulation of cytokinin-activat...	0.00769
GO:0045723	positive regulation of fatty acid biosyn...	0.01025
GO:0046167	glycerol-3-phosphate biosynthetic proces...	0.01025
GO:1901959	positive regulation of cutin biosyntheti...	0.0111
GO:0009877	nodulation	0.0111
GO:0016540	protein autoprocessing	0.01194
GO:0061025	membrane fusion	0.01246
GO:0015757	galactose transport	0.01279
GO:0050691	regulation of defense response to virus ...	0.01449
GO:0010583	response to cyclopentenone	0.01471
GO:0015783	GDP-fucose transport	0.01533
GO:0006168	adenine salvage	0.01786
GO:0019563	glycerol catabolic process	0.01786
GO:0010271	regulation of chlorophyll catabolic proc...	0.01955
GO:0045910	negative regulation of DNA recombination	0.01955
GO:0010255	glucose mediated signaling pathway	0.02039
GO:0010188	response to microbial phytotoxin	0.02039
GO:0009833	plant-type primary cell wall biogenesis	0.02039
GO:0044209	AMP salvage	0.02207
GO:0009809	lignin biosynthetic process	0.023
GO:0006270	DNA replication initiation	0.02331
GO:0072334	UDP-galactose transmembrane transport	0.02459
GO:0015786	UDP-glucose transport	0.02542
GO:0010389	regulation of G2/M transition of mitotic...	0.02639

o

GO.ID	Term	p.value
GO:0031047	gene silencing by RNA	0.000073
GO:0046256	2,4,6-trinitrotoluene catabolic process	0.00013
GO:0006474	N-terminal protein amino acid acetylatio...	0.0004
GO:0015969	guanosine tetraphosphate metabolic proce...	0.00047
GO:0006000	fructose metabolic process	0.00056
GO:0006003	fructose 2,6-bisphosphate metabolic proc...	0.00056
GO:0031508	pericentric heterochromatin assembly	0.00074
GO:0090309	positive regulation of methylation-depen...	0.00074
GO:0019281	L-methionine biosynthetic process from h...	0.00074
GO:0016444	somatic cell DNA recombination	0.00103
GO:0043609	regulation of carbon utilization	0.00119
GO:1902039	negative regulation of seed dormancy pro...	0.00237
GO:0006623	protein targeting to vacuole	0.00291
GO:0042594	response to starvation	0.00302
GO:0009662	etioplast organization	0.00311
GO:0080188	RNA-directed DNA methylation	0.00352
GO:0010207	photosystem II assembly	0.00383
GO:0006002	fructose 6-phosphate metabolic process	0.00387
GO:0010424	DNA methylation on cytosine within a CG ...	0.00438
GO:0009657	plastid organization	0.00444
GO:0048838	release of seed from dormancy	0.00534
GO:0015749	monosaccharide transport	0.00584
GO:0031935	regulation of chromatin silencing	0.00688
GO:0010030	positive regulation of seed germination	0.00748
GO:0009939	positive regulation of gibberellic acid ...	0.00753
GO:0015691	cadmium ion transport	0.00934
GO:0080086	stamen filament development	0.01005
GO:0006878	cellular copper ion homeostasis	0.01365
GO:0003333	amino acid transmembrane transport	0.01372
GO:0006275	regulation of DNA replication	0.01381

p

GO.ID	Term	p.value
GO:0010160	formation of organ boundary	0.000079
GO:0071555	cell wall organization	0.00066
GO:0010541	acropetal auxin transport	0.00101
GO:0009306	protein secretion	0.00225
GO:0009740	gibberellic acid mediated signaling path...	0.00242
GO:0050829	defense response to Gram-negative bacter...	0.00293
GO:0080086	stamen filament development	0.00314
GO:0010315	auxin efflux	0.00339
GO:0010540	basipetal auxin transport	0.00472
GO:0080167	response to karrikin	0.005
GO:0010218	response to far red light	0.00883
GO:0000387	spliceosomal snRNP assembly	0.01019
GO:0006412	translation	0.01277
GO:0001510	RNA methylation	0.01327
GO:0090058	metaxylem development	0.01422
GO:0006407	rRNA export from nucleus	0.01422
GO:0010941	regulation of cell death	0.01422
GO:0009958	positive gravitropism	0.01612
GO:0048443	stamen development	0.01651
GO:0008380	RNA splicing	0.01673
GO:0000395	mRNA 5'-splice site recognition	0.01704
GO:0009686	gibberellin biosynthetic process	0.01781
GO:0018108	peptidyl-tyrosine phosphorylation	0.02032
GO:0006098	pentose-phosphate shunt	0.0208
GO:0000290	deadenylation-dependent decapping of nuc...	0.02265
GO:0051649	establishment of localization in cell	0.02284
GO:1902358	sulfate transmembrane transport	0.02326
GO:0044571	[2Fe-2S] cluster assembly	0.02544
GO:0010273	detoxification of copper ion	0.02544
GO:0010207	photosystem II assembly	0.02661

q

GO.ID	Term	p.value
GO:1900000	regulation of anthocyanin catabolic proc...	0.0000032
GO:1901527	abscisic acid-activated signaling pathwa...	0.0000032
GO:0010089	xylem development	0.00058
GO:2000603	regulation of secondary growth	0.00283
GO:0051291	protein heterooligomerization	0.00377
GO:0046685	response to arsenic-containing substance	0.00471
GO:1901430	positive regulation of syringal lignin b...	0.00518
GO:0090435	protein localization to nuclear envelope	0.00565
GO:0009102	biotin biosynthetic process	0.00799
GO:0006430	lysyl-tRNA aminoacylation	0.00799
GO:0048657	anther wall tapetum cell differentiation	0.00799
GO:0009690	cytokinin metabolic process	0.00826
GO:0042631	cellular response to water deprivation	0.01164
GO:0007094	mitotic spindle assembly checkpoint	0.01313
GO:0010268	brassinosteroid homeostasis	0.01406
GO:0010286	heat acclimation	0.01576
GO:0010224	response to UV-B	0.01818
GO:0045927	positive regulation of growth	0.02517
GO:0051259	protein oligomerization	0.02579
GO:0051260	protein homooligomerization	0.04116
GO:0048316	seed development	0.04446
GO:0044237	cellular metabolic process	0.05082
GO:0007049	cell cycle	0.06919
GO:0009911	positive regulation of flower developmen...	0.06929
GO:0048767	root hair elongation	0.08761
GO:0051645	Golgi localization	0.08847
GO:0060151	peroxisome localization	0.08847
GO:0051646	mitochondrion localization	0.09062
GO:0009718	anthocyanin-containing compound biosynth...	0.0962
GO:0006310	DNA recombination	0.09786

Supplementary Table 13. Number of *R*-genes absent in (a) non-synthetics and (b) synthetics.

a

Type/Morphotype	Accession	No. of genes absent
Oilseed	Abukuma Natane	124
	Alaska	129
	Aragon	131
	Beluga	128
	CanberraxCourageDH	141
	Darmor-*bzh*	141
	DH5	125
	Dippes	122
	E94197	124
	EVVIN	130
	Expert	133
	Gross Luesewitzer	121
	Jupiter	121
	Kromerska	117
	Major	125
	MSL007C	121
	Olimpiade	112
	Pacific	129
	Pirola	132
	Rapid	125
	Savannah	133
	Tapidor	130
	Vivol	138
	Wotan	131
	**Average**	**128**
Fodder	English_Giant_194	130
	Nunsdale	98
	Palu	119
	**Average**	**116**
Swede	Fortin	126
	Sensation	118
	**Average**	**122**
Vegetable	Chuosenshu	116
	Gruner Schnittkohl	125
	**Average**	**121**

b

Type/Morphotype	Accession	No. of genes absent
**Synthetic**	CRY_1	85
	G50	121
	H149	120
	H165	118
	H176	123
	H44	102
	HIY_1	94
	MOY_4	97
	OLL1	85
	R_13_6	81
	R53	122
	R76	110
	Resyn-Go S4	129
	RS_10_7	91
	RS_4_6	108
	RS_7_6	88
	RS_8_6	113
	S_13	130
	S_39	115
	**Average**	**107**

The domains used to identify the *R-genes* are listed in Supplementary Table 18.

On average, the oilseed types, the synthetics, swedes, vegetable types and fodder types were missing 128, 107, 122, 121 and 116 candidate resistance genes, respectively.

Supplementary Table 14. Number of acyl lipid metabolism genes absent (a) non-synthetics and (b) synthetics.

a

Type/Morphotype	Accession	No. of genes absent
**Oilseed**	Abukuma Natane	27
	Alaska	33
	Aragon	38
	Beluga	39
	CanberraxCourageDH	82
	Darmor-*bzh*	20
	DH5	34
	Dippes	41
	E94197	73
	EVVIN	67
	Expert	44
	Gross Luesewitzer	27
	Jupiter	24
	Kromerska	13
	Major	50
	MSL007C	34
	Olimpiade	37
	Pacific	44
	Pirola	34
	Rapid	31
	Savannah	24
	Tapidor	33
	Vivol	28
	Wotan	57
	**Average**	**39**
**Fodder**	English_Giant_194	58
	Nunsdale	27
	Palu	87
	**Average**	**57**
**Swede**	Fortin	26
	Sensation	55
	**Average**	**41**
**Vegetable**	Chuosenshu	35
	Gruner Schnittkohl	37
	**Average**	**36**

b

Type/Morphotype	Accession	No. of genes absent
Synthetic	CRY_1	32
	G50	34
	H149	35
	H165	31
	H176	24
	H44	62
	HIY_1	28
	MOY_4	28
	OLL1	69
	R_13_6	53
	R53	49
	R76	30
	Resyn-Go S4	16
	RS_10_7	38
	RS_4_6	36
	RS_7_6	77
	RS_8_6	50
	S_13	66
	S_39	45
	**Average**	**42**

On average, the oilseeds, synthetics, swedes, vegetable types and fodder types were missing 39, 42, 41, 36 and 57 acyl lipid metabolism genes. However, these numbers would be biased due to the low sample number for the fodder types, swedes and vegetable types. However, when considering the unique instance of each gene in each morphotype, the synthetics were found to be missing the greatest number of genes (388) while the oilseeds were missing the least (129). Similarly, the fodder types, swedes and vegetable types were missing 57, 68 and 76 genes, respectively.

Supplementary Table 15. Number of GSL metabolism genes absent in (a) non-synthetics and (b) synthetics.

a

Type/morphotype	Accession	Number of absent genes
**Oilseed**	Abukuma Natane	2
	Alaska	0
	Aragon	2
	Beluga	4
	CanberraxCourageDH	1
	Darmor-*bzh*	2
	DH5	4
	Dippes	2
	E94197	1
	EVVIN	3
	Expert	2
	Gross Luesewitzer	2
	Jupiter	4
	Kromerska	2
	Major	2
	MSL007C	1
	Olimpiade	2
	Pacific	6
	Pirola	3
	Rapid	1
	Savannah	2
	Tapidor	1
	Vivol	2
	Wotan	1
	**Average**	**2**
**Fodder**	English_Giant_194	3
	Palu	3
	Nunsdale	2
	**Average**	**3**
**Swede**	Sensation	7
	Fortin	1
	**Average**	**4**
**Vegetable**	Gruner Schnittkohl	6
	Chuosenshu	5
	**Average**	**6**

b

Type/morphotype	Accession	Number of absent genes
Synthetic	CRY_1	5
	G50	8
	H149	4
	H165	7
	H176	2
	H44	4
	HIY_1	2
	MOY_4	1
	OLL1	1
	R_13_6	0
	R53	12
	R76	8
	Resyn-Go S4	2
	RS_10_7	5
	RS_4_6	8
	RS_7_6	2
	RS_8_6	2
	S_13	3
	S_39	7
	**Average**	**4**

On average, the oilseeds, synthetics, swedes, vegetable types and fodder types were missing 2, 4, 4, 6 and 3 GSL metabolism genes. However, these numbers would be biased due to the low sample number for the fodder types, swedes and vegetable types. However, when considering the unique instance of each gene in each morphotype, the synthetics were found to be missing the greatest number of genes (46) while the oilseeds were missing the least (13). Similarly, the fodder types, swedes and vegetable types were missing 4, 7 and 7, respectively.

Supplementary Table 16. Reads used for pangenome assembly.

Name	Technology	Insert size	No. of reads	Coverage
Tapidor	Illumina MP	3000-8000	232856562	21.64
Tapidor	Illumina PE	200-500	591898425	58.87
Dippes	Illumina PE	190	153357542	13.57
Gross-Luesewitzer	Illumina PE	180	188295366	16.66
Jupiter	Illumina PE	289	165366150	14.63
Kromerska	Illumina PE	298	442905328	39.2
Major	Illumina PE	194	172830694	15.29
Skziverskij	Illumina PE	208	87711118	7.76
Start	Illumina PE	214	96472964	8.54
Aragon	Illumina PE	200	233093964	20.63
Beluga	Illumina PE	192	176014670	15.58
Olimpiade	Illumina PE	171	150272844	13.3
Wotan	Illumina PE	202	198746940	17.59
Canberra x Courage DH	Illumina PE	301	162726868	14.4
Resyn-Go S4	Illumina PE	227	117449460	10.39
Alaska	Illumina PE	196	219654046	19.44
Pirola	Illumina PE	160	133394340	11.8
Rapid	Illumina PE	207	175722650	15.55
Pacific	Illumina PE	311	120115248	10.63
Savannah	Illumina PE	312	179264778	15.86
Vivol	Illumina PE	319	168422320	14.9
Expert	Illumina PE	200	181589328	16.07
G 50	Illumina PE	178	138584190	12.26
H44	Illumina PE	303	162685050	14.4
H 149	Illumina PE	180	239431864	21.19
H 165	Illumina PE	172	168442916	14.91
H 176	Illumina PE	158	122837286	10.87
R53	Illumina PE	296	167093140	14.79
R76	Illumina PE	317	197589982	17.49
RS 4/6	Illumina PE	304	162966518	14.42
RS 10/7	Illumina PE	204	189715046	16.79
RS 13/6	Illumina PE	293	165220254	14.62
S 13	Illumina PE	212	141930992	12.56
OLL 1	Illumina PE	169	253552706	22.44
Chuosenshu	Illumina PE	191	200311236	17.73
Grüner Schnittkohl	Illumina PE	185	142128628	12.58
R 99	Illumina PE	158	100203212	8.87
RS 7/6	Illumina PE	199	178669242	15.81
RS 8/6	Illumina PE	197	224506148	19.87

S 39	Illumina PE	209	188940778	16.72
CRY 1	Illumina PE	307	181589328	16.07
HIY 1	Illumina PE	306	156257896	13.83
MOY 4	Illumina PE	306	158120246	13.99
English Giant 194	Illumina PE	303	167224372	14.8
Nunsdale	Illumina PE	179	182828324	16.18
Palu	Illumina PE	161	200369820	17.73
Abukuma Natane	Illumina PE	204	196171476	17.36
E94197	Illumina PE	199	222812810	19.72
Evvin	Illumina PE	199	184506352	16.33
Fortin Family	Illumina PE	198	161362924	14.28
Sensation NZ	Illumina PE	209	156426460	13.84
DH 5 (Oase*Nugget)	Illumina PE	204	297313104	26.31
MSL007C	Illumina PE	292	230282774	20.38

Supplementary Table 17. Pfam domains used to identify TE genes.

**Pfam domains used**	PF00075,PF00077,PF00078,PF00098,PF00424,PF00429,PF00469, PF00516,PF00517,PF00522,PF00539,PF00540,PF00552,PF00558, PF00559,PF00589,PF00607,PF00665,PF00692,PF00872,PF00906, PF00971,PF00979,PF01021,PF01045,PF01054,PF01140,PF01141, PF01359,PF01385,PF01498,PF01526,PF01527,PF01548,PF01609, PF01610,PF01695,PF01710,PF01797,PF02022,PF02093,PF02228, PF02281,PF02316,PF02337,PF02371,PF02411,PF02720,PF02813, PF02892,PF02914,PF02920,PF02959,PF02992,PF02994,PF02998, PF03004,PF03017,PF03050,PF03056,PF03078,PF03108,PF03184, PF03221,PF03274,PF03276,PF03400,PF03408,PF03539,PF03708, PF03716,PF03732,PF03811,PF04094,PF04160,PF04195,PF04218, PF04236,PF04582,PF04693,PF04740,PF04754,PF04827,PF04937, PF04986,PF05052,PF05344,PF05380,PF05399,PF05457,PF05485, PF05598,PF05599,PF05621,PF05699,PF05717,PF05754,PF05840, PF05851,PF05858,PF05928,PF06527,PF06815,PF06817,PF07253, PF07282,PF07567,PF07572,PF07592,PF07727,PF07999,PF08284, PF08333,PF08483,PF08705,PF08721,PF08722,PF08723,PF09035, PF09039,PF09077,PF09293,PF09299,PF09322,PF13966,PF10551, PF13456,PF14223,PF01107,PF13952,PF13963,PF13975,PF13976, PF14214,PF14227,PF14244,PF14372

Supplementary Table 18. Domains used to identify *R*-genes.

**Domain name**	**pfam**
TIR2	PF13676
TIR	PF01582
NB-ARC	PF00931
LRR_1	PF00560
LRR_2	PF07723
LRR_3	PF07725
LRR_4	PF12799
LRR_5	PF13306
LRR_6	PF13516
LRR_7	PF13855
LRR_8	PF14580

The Coils database was used to identify CC domains.

Supplementary Table 19. Order of accession on Circos plot for (a) non-synthetics and (b) synthetics.

a

Accession	Order
Abukuma Natane	1
Alaska	2
Aragon	3
Beluga	4
CanberraxCourageDH	5
Chuosenshu	6
Darmor-*bzh*	7
DH5	8
Dippes	9
E94197	10
English_Giant_194	11
EVVIN	12
Expert	13
Fortin	14
Gross Luesewitzer	15
Gruner Schnittkohl	16
Jupiter	17
Kromerska	18
Major	19
MSL007C	20
Nunsdale	21
Olimpiade	22
Pacific	23
Palu	24
Pirola	25
Rapid	26
Savannah	27
Sensation	28
Tapidor	29
Vivol	30
Wotan	31

b

Accession	Order
CRY_1	32
G50	33
H149	34
H165	35
H176	36
H44	37
HIY_1	38
MOY_4	39
OLL1	40
R_13_6	41
R53	42
R76	43
Resyn-Go S4	44
RS_10_7	45
RS_4_6	46
RS_7_6	47
RS_8_6	48
S_13	49
S_39	50

The order in which the accessions appear (outer to inner) on the circos plots (Supplementary Figure 1). The synthetic accessions are represented by orange dots (and orange circles) and the non-synthetic accessions are represented by blue dots (and blue circles).
