# Supplementary material for: Homoeologous exchange is a major cause of gene presence/absence variation in the amphidiploid Brassica napus
Source: Plant Biotechnol J. 2018 Jan 10;16(7):1265–74. doi: 10.1111/pbi.12867 (PMC5999312; doi:10.1111/pbi.12867)
Supplement: Supplementary file 4 — Data S1 Results of the HE analysis across the Darmor‐bzh portion of the pangenome. [file PBI-16-1265-s005.pdf]

Supplementary File 2: Results of the HE analysis across the Darmor-*bzh* portion of the pangenome. 'Total no. of lost genes' refers to the number of HE-related and non-HE-related PAV genes in the donor region on chromosome (Xn).

Accession	Direction of HE (Xn to Yn)	Total no. of lost genes on chromosome Xn	No. of HE-PAVs on chromosome Yn
H165	A01 to C01	637	277
RS_10_7	A01 to C01	487	178
RS_4_6	A01 to C01	1037	481
CRY_1	A02 to C02	527	182
H165	A02 to C02	41	2
S_39	A02 to C02	513	233
RS_10_7	A02 to C02	549	206
H165	A04 to C04	296	123
R76	A07 to C06	192	84
RS_4_6	A07 to C06	209	82
H149	A09 to C08	597	208
S_39	A09 to C08	319	110
Sensation	A09 to C09	336	81
Nunsdale	A09 to C09	131	47
G50	A09 to C09	354	144
H176	A09 to C09	242	66
R53	A09 to C09	300	77
Expert	A10 to C09	74	7
S_39	A10 to C09	284	94
Chuosenshu	C01 to A01	564	251
Gruner Schnittkohl	C01 to A01	696	280
Sensation	C01 to A01	247	79
CRY_1	C01 to A01	794	233
G50	C01 to A01	333	131
S_39	C01 to A01	296	115
Major	C01 to A01	280	79
H165	C01 to A01	250	64
G50	C02 to A02	432	113
Tapidor	C02 to A02	482	129
H165	C02 to A02	1272	446
H176	C02 to A02	273	85
R53	C02 to A02	1609	618
R76	C02 to A02	280	30
RS_10_7	C02 to A02	504	121
Abukuma Nantane	C03 to A03	527	223
Evvin	C03 to A03	303	86
Expert	C03 to A03	244	89
H149	C03 to A03	272	93
H44	C03 to A03	287	61
Pirola	C03 to A03	486	192
RS_4_6	C03 to A03	605	47
Sensation	C03 to A03	465	157
Tapidor	C03 to A03	272	72
Gruner Schnittkohl	C04 to A04	338	120
CRY_1	C04 to A04	288	64

Gruner Schnittkohl	C04 to A05	338	14
CRY_1	C04 to A05	288	5
Gruner Schnittkohl	C05 to A05	419	204
R53	C05 to A05	466	192
RS_10_7	C05 to A05	282	87
H149	C06 to A07	194	64
RS_7_6	C06 to A07	140	30
Chuosenshu	C08 to A09	195	80
DH5	C08 to A09	248	105
Pacific	C08 to A09	289	126
Sensation	C08 to A09	221	88
G50	C08 to A09	659	337
H165	C08 to A09	195	50
H44	C08 to A09	297	118
Dippes	C09 to A09	186	62
EVVIN	C09 to A09	83	20
Gross Luesewitzer	C09 to A09	103	29
Gruner Schnittkohl	C09 to A09	159	54
Jupiter	C09 to A09	155	51
Major	C09 to A09	176	63
Sensation	C09 to A09	255	69
Wotan	C09 to A09	200	61
H149	C09 to A09	247	23
H44	C09 to A09	361	98
Resyn_Go_S4	C09 to A09	273	101
RS_7_6	C09 to A09	306	82
English_Giant_194	C09 to A09	234	96
H149	C09 to A10	247	39

List of HE genes (A01 to C01)

**Accession**	**Lost gene**	**Duplicated HE gene**
H165	BnaA01g00010.1D2	BnaC01g00670.1D2
H165	BnaA01g00030.1D2	BnaC01g00690.1D2
H165	BnaA01g00040.1D2	BnaC01g00700.1D2
H165	BnaA01g00060.1D2	BnaC01g00730.1D2
H165	BnaA01g00080.1D2	BnaC01g00750.1D2
H165	BnaA01g00180.1D2	BnaC01g00860.1D2
H165	BnaA01g00260.1D2	BnaC01g00960.1D2
H165	BnaA01g00330.1D2	BnaC01g01030.1D2
H165	BnaA01g00360.1D2	BnaC01g01070.1D2
H165	BnaA01g00380.1D2	BnaC01g01090.1D2
H165	BnaA01g00480.1D2	BnaC01g01170.1D2
H165	BnaA01g00500.1D2	BnaC01g01190.1D2
H165	BnaA01g00530.1D2	BnaC01g01220.1D2
H165	BnaA01g00630.1D2	BnaC01g01310.1D2
H165	BnaA01g00640.1D2	BnaC01g01320.1D2
H165	BnaA01g00660.1D2	BnaC01g01340.1D2
H165	BnaA01g00670.1D2	BnaC01g01350.1D2
H165	BnaA01g00780.1D2	BnaC01g01470.1D2
H165	BnaA01g00850.1D2	BnaC01g01550.1D2
H165	BnaA01g00890.1D2	BnaC01g01600.1D2

H165	BnaA01g00920.1D2	BnaC01g01630.1D2
H165	BnaA01g00970.1D2	BnaC01g01670.1D2
H165	BnaA01g01050.1D2	BnaC01g01770.1D2
H165	BnaA01g01070.1D2	BnaC01g01790.1D2
H165	BnaA01g01160.1D2	BnaC01g01880.1D2
H165	BnaA01g01170.1D2	BnaC01g01880.1D2
H165	BnaA01g01180.1D2	BnaC01g01890.1D2
H165	BnaA01g01310.1D2	BnaC01g02000.1D2
H165	BnaA01g01510.1D2	BnaC01g02220.1D2
H165	BnaA01g01540.1D2	BnaC01g02250.1D2
H165	BnaA01g01720.1D2	BnaC01g02430.1D2
H165	BnaA01g01760.1D2	BnaC01g02470.1D2
H165	BnaA01g01770.1D2	BnaC01g02480.1D2
H165	BnaA01g01780.1D2	BnaC01g02490.1D2
H165	BnaA01g01830.1D2	BnaC01g02540.1D2
H165	BnaA01g01890.1D2	BnaC01g02600.1D2
H165	BnaA01g01900.1D2	BnaC01g02600.1D2
H165	BnaA01g01910.1D2	BnaC01g02600.1D2
H165	BnaA01g01970.1D2	BnaC01g02660.1D2
H165	BnaA01g01980.1D2	BnaC01g02670.1D2
H165	BnaA01g02030.1D2	BnaC01g02710.1D2
H165	BnaA01g02070.1D2	BnaC01g02740.1D2
H165	BnaA01g02280.1D2	BnaC01g03070.1D2
H165	BnaA01g02290.1D2	BnaC01g03080.1D2
H165	BnaA01g02310.1D2	BnaC01g03100.1D2
H165	BnaA01g02320.1D2	BnaC01g03110.1D2
H165	BnaA01g02380.1D2	BnaC01g03170.1D2
H165	BnaA01g02390.1D2	BnaC01g03180.1D2
H165	BnaA01g02430.1D2	BnaC01g03220.1D2
H165	BnaA01g02640.1D2	BnaC01g03500.1D2
H165	BnaA01g02710.1D2	BnaC01g03560.1D2
H165	BnaA01g02750.1D2	BnaC01g03590.1D2
H165	BnaA01g02780.1D2	BnaC01g03650.1D2
H165	BnaA01g02790.1D2	BnaC01g07600.1D2
H165	BnaA01g02810.1D2	BnaC01g03670.1D2
H165	BnaA01g02870.1D2	BnaC01g03730.1D2
H165	BnaA01g02950.1D2	BnaC01g03840.1D2
H165	BnaA01g02960.1D2	BnaC01g03850.1D2
H165	BnaA01g02980.1D2	BnaC01g03870.1D2
H165	BnaA01g03110.1D2	BnaC01g04030.1D2
H165	BnaA01g03130.1D2	BnaC01g04050.1D2
H165	BnaA01g03170.1D2	BnaC01g04110.1D2
H165	BnaA01g03210.1D2	BnaC01g04150.1D2
H165	BnaA01g03220.1D2	BnaC01g04160.1D2
H165	BnaA01g03290.1D2	BnaC01g04230.1D2
H165	BnaA01g03310.1D2	BnaC01g04250.1D2
H165	BnaA01g03330.1D2	BnaC01g04270.1D2
H165	BnaA01g03350.1D2	BnaC01g04290.1D2
H165	BnaA01g03410.1D2	BnaC01g04370.1D2
H165	BnaA01g03430.1D2	BnaC01g04390.1D2
H165	BnaA01g03440.1D2	BnaC01g04400.1D2

H165	BnaA01g03500.1D2	BnaC01g04460.1D2
H165	BnaA01g03550.1D2	BnaC01g04530.1D2
H165	BnaA01g03580.1D2	BnaC01g04570.1D2
H165	BnaA01g03590.1D2	BnaC01g04580.1D2
H165	BnaA01g03600.1D2	BnaC01g04590.1D2
H165	BnaA01g03620.1D2	BnaC01g04600.1D2
H165	BnaA01g03640.1D2	BnaC01g04620.1D2
H165	BnaA01g03730.1D2	BnaC01g04660.1D2
H165	BnaA01g03750.1D2	BnaC01g04670.1D2
H165	BnaA01g03770.1D2	BnaC01g04690.1D2
H165	BnaA01g03780.1D2	BnaC01g04700.1D2
H165	BnaA01g03810.1D2	BnaC01g04830.1D2
H165	BnaA01g03850.1D2	BnaC01g04870.1D2
H165	BnaA01g03980.1D2	BnaC01g05010.1D2
H165	BnaA01g04250.1D2	BnaC01g05280.1D2
H165	BnaA01g04370.1D2	BnaC01g05440.1D2
H165	BnaA01g04440.1D2	BnaC01g05510.1D2
H165	BnaA01g04640.1D2	BnaC01g05740.1D2
H165	BnaA01g04760.1D2	BnaC01g05860.1D2
H165	BnaA01g04930.1D2	BnaC01g01560.1D2
H165	BnaA01g05010.1D2	BnaC01g01410.1D2
H165	BnaA01g05120.1D2	BnaC01g06030.1D2
H165	BnaA01g05170.1D2	BnaC01g02820.1D2
H165	BnaA01g05200.1D2	BnaC01g02870.1D2
H165	BnaA01g05240.1D2	BnaC01g00070.1D2
H165	BnaA01g05260.1D2	BnaC01g00090.1D2
H165	BnaA01g05480.1D2	BnaC01g00330.1D2
H165	BnaA01g05550.1D2	BnaC01g00570.1D2
H165	BnaA01g05580.1D2	BnaC01g00430.1D2
H165	BnaA01g05650.1D2	BnaC01g00470.1D2
H165	BnaA01g05780.1D2	BnaC01g06070.1D2
H165	BnaA01g05850.1D2	BnaC01g06120.1D2
H165	BnaA01g05870.1D2	BnaC01g06140.1D2
H165	BnaA01g05900.1D2	BnaC01g06180.1D2
H165	BnaA01g05980.1D2	BnaC01g06270.1D2
H165	BnaA01g06080.1D2	BnaC01g06400.1D2
H165	BnaA01g06150.1D2	BnaC01g06460.1D2
H165	BnaA01g06180.1D2	BnaC01g06490.1D2
H165	BnaA01g06380.1D2	BnaC01g06600.1D2
H165	BnaA01g06400.1D2	BnaC01g06630.1D2
H165	BnaA01g06410.1D2	BnaC01g06640.1D2
H165	BnaA01g06420.1D2	BnaC01g06650.1D2
H165	BnaA01g06610.1D2	BnaC01g06970.1D2
H165	BnaA01g06620.1D2	BnaC01g06990.1D2
H165	BnaA01g06680.1D2	BnaC01g07070.1D2
H165	BnaA01g06760.1D2	BnaC01g07120.1D2
H165	BnaA01g06820.1D2	BnaC01g07190.1D2
H165	BnaA01g06830.1D2	BnaC01g07200.1D2
H165	BnaA01g06840.1D2	BnaC01g07210.1D2
H165	BnaA01g06870.1D2	BnaC01g07240.1D2
H165	BnaA01g06890.1D2	BnaC01g07260.1D2

H165	BnaA01g06900.1D2	BnaC01g07270.1D2
H165	BnaA01g06990.1D2	BnaC01g07390.1D2
H165	BnaA01g07000.1D2	BnaC01g07400.1D2
H165	BnaA01g07010.1D2	BnaC01g07410.1D2
H165	BnaA01g07020.1D2	BnaC01g07420.1D2
H165	BnaA01g07050.1D2	BnaC01g07450.1D2
H165	BnaA01g07070.1D2	BnaC01g07470.1D2
H165	BnaA01g07100.1D2	BnaC01g07880.1D2
H165	BnaA01g07140.1D2	BnaC01g07920.1D2
H165	BnaA01g07150.1D2	BnaC01g08000.1D2
H165	BnaA01g07160.1D2	BnaC01g07990.1D2
H165	BnaA01g07180.1D2	BnaC01g07970.1D2
H165	BnaA01g07300.1D2	BnaC01g08150.1D2
H165	BnaA01g07400.1D2	BnaC01g08250.1D2
H165	BnaA01g07410.1D2	BnaC01g08260.1D2
H165	BnaA01g07470.1D2	BnaC01g08310.1D2
H165	BnaA01g07480.1D2	BnaC01g07630.1D2
H165	BnaA01g07510.1D2	BnaC01g07630.1D2
H165	BnaA01g07520.1D2	BnaC01g08330.1D2
H165	BnaA01g07540.1D2	BnaC01g09100.1D2
H165	BnaA01g07550.1D2	BnaC01g09100.1D2
H165	BnaA01g07770.1D2	BnaC01g08540.1D2
H165	BnaA01g07800.1D2	BnaC01g08570.1D2
H165	BnaA01g07830.1D2	BnaC01g08580.1D2
H165	BnaA01g07840.1D2	BnaC01g08590.1D2
H165	BnaA01g07870.1D2	BnaC01g08620.1D2
H165	BnaA01g08030.1D2	BnaC01g08840.1D2
H165	BnaA01g08060.1D2	BnaC01g08870.1D2
H165	BnaA01g08070.1D2	BnaC01g08880.1D2
H165	BnaA01g08100.1D2	BnaC01g08900.1D2
H165	BnaA01g08260.1D2	BnaC01g09050.1D2
H165	BnaA01g08280.1D2	BnaC01g09160.1D2
H165	BnaA01g08300.1D2	BnaC01g09140.1D2
H165	BnaA01g08320.1D2	BnaC01g09120.1D2
H165	BnaA01g08330.1D2	BnaC01g09110.1D2
H165	BnaA01g08390.1D2	BnaC01g09200.1D2
H165	BnaA01g08430.1D2	BnaC01g09250.1D2
H165	BnaA01g08840.1D2	BnaC01g09760.1D2
H165	BnaA01g08990.1D2	BnaC01g09860.1D2
H165	BnaA01g09030.1D2	BnaC01g13150.1D2
H165	BnaA01g09140.1D2	BnaC01g10040.1D2
H165	BnaA01g09160.1D2	BnaC01g10060.1D2
H165	BnaA01g09200.1D2	BnaC01g10100.1D2
H165	BnaA01g09210.1D2	BnaC01g10110.1D2
H165	BnaA01g09320.1D2	BnaC01g10260.1D2
H165	BnaA01g09340.1D2	BnaC01g10280.1D2
H165	BnaA01g09350.1D2	BnaC01g10290.1D2
H165	BnaA01g09360.1D2	BnaC01g10300.1D2
H165	BnaA01g09520.1D2	BnaC01g10390.1D2
H165	BnaA01g09550.1D2	BnaC01g10420.1D2
H165	BnaA01g09560.1D2	BnaC01g10430.1D2

H165	BnaA01g09570.1D2	BnaC01g10440.1D2
H165	BnaA01g09580.1D2	BnaC01g10490.1D2
H165	BnaA01g09590.1D2	BnaC01g10500.1D2
H165	BnaA01g09610.1D2	BnaC01g10520.1D2
H165	BnaA01g09620.1D2	BnaC01g10530.1D2
H165	BnaA01g09630.1D2	BnaC01g10540.1D2
H165	BnaA01g09810.1D2	BnaC01g10760.1D2
H165	BnaA01g09820.1D2	BnaC01g10770.1D2
H165	BnaA01g09840.1D2	BnaC01g12900.1D2
H165	BnaA01g09880.1D2	BnaC01g12940.1D2
H165	BnaA01g10130.1D2	BnaC01g11310.1D2
H165	BnaA01g10220.1D2	BnaC01g11480.1D2
H165	BnaA01g10280.1D2	BnaC01g11570.1D2
H165	BnaA01g10310.1D2	BnaC01g11600.1D2
H165	BnaA01g10360.1D2	BnaC01g11630.1D2
H165	BnaA01g10400.1D2	BnaC01g11670.1D2
H165	BnaA01g10430.1D2	BnaC01g11710.1D2
H165	BnaA01g10510.1D2	BnaC01g11770.1D2
H165	BnaA01g10560.1D2	BnaC01g11800.1D2
H165	BnaA01g10570.1D2	BnaC01g11810.1D2
H165	BnaA01g10600.1D2	BnaC01g11840.1D2
H165	BnaA01g10610.1D2	BnaC01g11850.1D2
H165	BnaA01g10980.1D2	BnaC01g12230.1D2
H165	BnaA01g11050.1D2	BnaC01g12340.1D2
H165	BnaA01g11110.1D2	BnaC01g12390.1D2
H165	BnaA01g11180.1D2	BnaC01g12470.1D2
H165	BnaA01g11290.1D2	BnaC01g12550.1D2
H165	BnaA01g11300.1D2	BnaC01g12560.1D2
H165	BnaA01g11350.1D2	BnaC01g12630.1D2
H165	BnaA01g11370.1D2	BnaC01g12650.1D2
H165	BnaA01g11440.1D2	BnaC01g12750.1D2
H165	BnaA01g11530.1D2	BnaC01g15620.1D2
H165	BnaA01g11700.1D2	BnaC01g10460.1D2
H165	BnaA01g11720.1D2	BnaC01g11440.1D2
H165	BnaA01g11730.1D2	BnaC01g09080.1D2
H165	BnaA01g11740.1D2	BnaC01g09090.1D2
H165	BnaA01g11870.1D2	BnaC01g12850.1D2
H165	BnaA01g11900.1D2	BnaC01g13240.1D2
H165	BnaA01g11970.1D2	BnaC01g13300.1D2
H165	BnaA01g12120.1D2	BnaC01g13410.1D2
H165	BnaA01g12160.1D2	BnaC01g13440.1D2
H165	BnaA01g12200.1D2	BnaC01g15650.1D2
H165	BnaA01g12240.1D2	BnaC01g15690.1D2
H165	BnaA01g12330.1D2	BnaC01g13550.1D2
H165	BnaA01g12330.1D2	BnaC01g13560.1D2
H165	BnaA01g12430.1D2	BnaC01g13710.1D2
H165	BnaA01g12610.1D2	BnaC01g13740.1D2
H165	BnaA01g12650.1D2	BnaC01g14130.1D2
H165	BnaA01g12690.1D2	BnaC01g14200.1D2
H165	BnaA01g12710.1D2	BnaC01g14210.1D2
H165	BnaA01g12820.1D2	BnaC01g14310.1D2

H165	BnaA01g12840.1D2	BnaC01g14330.1D2
H165	BnaA01g12850.1D2	BnaC01g14340.1D2
H165	BnaA01g12930.1D2	BnaC01g14420.1D2
H165	BnaA01g12950.1D2	BnaC01g14190.1D2
H165	BnaA01g13030.1D2	BnaC01g14610.1D2
H165	BnaA01g13040.1D2	BnaC01g14620.1D2
H165	BnaA01g13120.1D2	BnaC01g14700.1D2
H165	BnaA01g13130.1D2	BnaC01g14710.1D2
H165	BnaA01g13220.1D2	BnaC01g14800.1D2
H165	BnaA01g13270.1D2	BnaC01g15580.1D2
H165	BnaA01g13360.1D2	BnaC01g14950.1D2
H165	BnaA01g13380.1D2	BnaC01g14970.1D2
H165	BnaA01g13410.1D2	BnaC01g15000.1D2
H165	BnaA01g13420.1D2	BnaC01g15010.1D2
H165	BnaA01g13470.1D2	BnaC01g15060.1D2
H165	BnaA01g13480.1D2	BnaC01g15070.1D2
H165	BnaA01g13650.1D2	BnaC01g15220.1D2
H165	BnaA01g13700.1D2	BnaC01g15270.1D2
H165	BnaA01g13720.1D2	BnaC01g15290.1D2
H165	BnaA01g13750.1D2	BnaC01g15320.1D2
H165	BnaA01g13910.1D2	BnaC01g14460.1D2
H165	BnaA01g13920.1D2	BnaC01g14450.1D2
H165	BnaA01g13970.1D2	BnaC01g15790.1D2
H165	BnaA01g14110.1D2	BnaC01g15910.1D2
H165	BnaA01g14260.1D2	BnaC01g16110.1D2
H165	BnaA01g14270.1D2	BnaC01g16120.1D2
H165	BnaA01g14340.1D2	BnaC01g16210.1D2
H165	BnaA01g14410.1D2	BnaC01g16330.1D2
H165	BnaA01g14420.1D2	BnaC01g16330.1D2
H165	BnaA01g14500.1D2	BnaC01g16410.1D2
H165	BnaA01g14530.1D2	BnaC01g16450.1D2
H165	BnaA01g14550.1D2	BnaC01g17290.1D2
H165	BnaA01g14620.1D2	BnaC01g16530.1D2
H165	BnaA01g14660.1D2	BnaC01g16600.1D2
H165	BnaA01g14670.1D2	BnaC01g16610.1D2
H165	BnaA01g14680.1D2	BnaC01g16620.1D2
H165	BnaA01g14730.1D2	BnaC01g16690.1D2
H165	BnaA01g14750.1D2	BnaC01g16700.1D2
H165	BnaA01g14780.1D2	BnaC01g16730.1D2
H165	BnaA01g14790.1D2	BnaC01g16740.1D2
H165	BnaA01g14800.1D2	BnaC01g16750.1D2
H165	BnaA01g14850.1D2	BnaC01g16800.1D2
H165	BnaA01g14920.1D2	BnaC01g16910.1D2
H165	BnaA01g14950.1D2	BnaC01g16940.1D2
H165	BnaA01g14960.1D2	BnaC01g17000.1D2
H165	BnaA01g14990.1D2	BnaC01g17030.1D2
H165	BnaA01g15120.1D2	BnaC01g17190.1D2
H165	BnaA01g15220.1D2	BnaC01g17420.1D2
H165	BnaA01g15360.1D2	BnaC01g17600.1D2
H165	BnaA01g15430.1D2	BnaC01g17650.1D2
H165	BnaA01g15430.1D2	BnaC01g17660.1D2

H165	BnaA01g15590.1D2	BnaC01g17770.1D2
H165	BnaA01g15660.1D2	BnaC01g17830.1D2
H165	BnaA01g15750.1D2	BnaC01g17930.1D2
H165	BnaA01g16390.1D2	BnaC01g16670.1D2
RS_10_7	BnaA01g01160.1D2	BnaC01g01880.1D2
RS_10_7	BnaA01g01170.1D2	BnaC01g01880.1D2
RS_10_7	BnaA01g01180.1D2	BnaC01g01890.1D2
RS_10_7	BnaA01g01240.1D2	BnaC01g01930.1D2
RS_10_7	BnaA01g01360.1D2	BnaC01g02040.1D2
RS_10_7	BnaA01g01420.1D2	BnaC01g02130.1D2
RS_10_7	BnaA01g01500.1D2	BnaC01g02210.1D2
RS_10_7	BnaA01g01520.1D2	BnaC01g02230.1D2
RS_10_7	BnaA01g01720.1D2	BnaC01g02430.1D2
RS_10_7	BnaA01g01810.1D2	BnaC01g02520.1D2
RS_10_7	BnaA01g01830.1D2	BnaC01g02540.1D2
RS_10_7	BnaA01g01850.1D2	BnaC01g02560.1D2
RS_10_7	BnaA01g01860.1D2	BnaC01g02570.1D2
RS_10_7	BnaA01g01890.1D2	BnaC01g02600.1D2
RS_10_7	BnaA01g01900.1D2	BnaC01g02600.1D2
RS_10_7	BnaA01g01910.1D2	BnaC01g02600.1D2
RS_10_7	BnaA01g11530.1D2	BnaC01g15620.1D2
RS_10_7	BnaA01g12790.1D2	BnaC01g14280.1D2
RS_10_7	BnaA01g12820.1D2	BnaC01g14310.1D2
RS_10_7	BnaA01g12840.1D2	BnaC01g14330.1D2
RS_10_7	BnaA01g12850.1D2	BnaC01g14340.1D2
RS_10_7	BnaA01g12930.1D2	BnaC01g14420.1D2
RS_10_7	BnaA01g12960.1D2	BnaC01g14520.1D2
RS_10_7	BnaA01g13000.1D2	BnaC01g14580.1D2
RS_10_7	BnaA01g13040.1D2	BnaC01g14620.1D2
RS_10_7	BnaA01g13120.1D2	BnaC01g14700.1D2
RS_10_7	BnaA01g13130.1D2	BnaC01g14710.1D2
RS_10_7	BnaA01g13170.1D2	BnaC01g14760.1D2
RS_10_7	BnaA01g13180.1D2	BnaC01g14770.1D2
RS_10_7	BnaA01g13190.1D2	BnaC01g14780.1D2
RS_10_7	BnaA01g13220.1D2	BnaC01g14800.1D2
RS_10_7	BnaA01g13270.1D2	BnaC01g15580.1D2
RS_10_7	BnaA01g13290.1D2	BnaC01g14850.1D2
RS_10_7	BnaA01g13310.1D2	BnaC01g14870.1D2
RS_10_7	BnaA01g13320.1D2	BnaC01g14900.1D2
RS_10_7	BnaA01g13330.1D2	BnaC01g14910.1D2
RS_10_7	BnaA01g13470.1D2	BnaC01g15060.1D2
RS_10_7	BnaA01g13480.1D2	BnaC01g15070.1D2
RS_10_7	BnaA01g13650.1D2	BnaC01g15220.1D2
RS_10_7	BnaA01g13720.1D2	BnaC01g15290.1D2
RS_10_7	BnaA01g13780.1D2	BnaC01g15350.1D2
RS_10_7	BnaA01g13910.1D2	BnaC01g14460.1D2
RS_10_7	BnaA01g13920.1D2	BnaC01g14450.1D2
RS_10_7	BnaA01g13930.1D2	BnaC01g15490.1D2
RS_10_7	BnaA01g13950.1D2	BnaC01g15520.1D2
RS_10_7	BnaA01g14220.1D2	BnaC01g16060.1D2
RS_10_7	BnaA01g14270.1D2	BnaC01g16120.1D2

RS_10_7	BnaA01g14340.1D2	BnaC01g16210.1D2
RS_10_7	BnaA01g14410.1D2	BnaC01g16330.1D2
RS_10_7	BnaA01g14420.1D2	BnaC01g16330.1D2
RS_10_7	BnaA01g14440.1D2	BnaC01g16350.1D2
RS_10_7	BnaA01g14500.1D2	BnaC01g16410.1D2
RS_10_7	BnaA01g14530.1D2	BnaC01g16450.1D2
RS_10_7	BnaA01g14610.1D2	BnaC01g16520.1D2
RS_10_7	BnaA01g14620.1D2	BnaC01g16530.1D2
RS_10_7	BnaA01g14670.1D2	BnaC01g16610.1D2
RS_10_7	BnaA01g14730.1D2	BnaC01g16690.1D2
RS_10_7	BnaA01g14850.1D2	BnaC01g16800.1D2
RS_10_7	BnaA01g14920.1D2	BnaC01g16910.1D2
RS_10_7	BnaA01g14990.1D2	BnaC01g17030.1D2
RS_10_7	BnaA01g15030.1D2	BnaC01g17080.1D2
RS_10_7	BnaA01g15170.1D2	BnaC01g17380.1D2
RS_10_7	BnaA01g15220.1D2	BnaC01g17420.1D2
RS_10_7	BnaA01g15380.1D2	BnaC01g17620.1D2
RS_10_7	BnaA01g15410.1D2	BnaC01g17630.1D2
RS_10_7	BnaA01g15410.1D2	BnaC01g17660.1D2
RS_10_7	BnaA01g15410.1D2	BnaC01g18040.1D2
RS_10_7	BnaA01g15420.1D2	BnaC01g17630.1D2
RS_10_7	BnaA01g15420.1D2	BnaC01g17660.1D2
RS_10_7	BnaA01g15420.1D2	BnaC01g18040.1D2
RS_10_7	BnaA01g15430.1D2	BnaC01g17630.1D2
RS_10_7	BnaA01g15430.1D2	BnaC01g17660.1D2
RS_10_7	BnaA01g15440.1D2	BnaC01g17630.1D2
RS_10_7	BnaA01g15440.1D2	BnaC01g17660.1D2
RS_10_7	BnaA01g15510.1D2	BnaC01g17690.1D2
RS_10_7	BnaA01g15600.1D2	BnaC01g17780.1D2
RS_10_7	BnaA01g15660.1D2	BnaC01g17830.1D2
RS_10_7	BnaA01g15750.1D2	BnaC01g17930.1D2
RS_10_7	BnaA01g15790.1D2	BnaC01g17950.1D2
RS_10_7	BnaA01g15800.1D2	BnaC01g17960.1D2
RS_10_7	BnaA01g16020.1D2	BnaC01g18720.1D2
RS_10_7	BnaA01g16070.1D2	BnaC01g18740.1D2
RS_10_7	BnaA01g16110.1D2	BnaC01g18790.1D2
RS_10_7	BnaA01g16120.1D2	BnaC01g18800.1D2
RS_10_7	BnaA01g16180.1D2	BnaC01g18850.1D2
RS_10_7	BnaA01g16190.1D2	BnaC01g18860.1D2
RS_10_7	BnaA01g16210.1D2	BnaC01g18880.1D2
RS_10_7	BnaA01g16220.1D2	BnaC01g18890.1D2
RS_10_7	BnaA01g16230.1D2	BnaC01g18900.1D2
RS_10_7	BnaA01g16250.1D2	BnaC01g18920.1D2
RS_10_7	BnaA01g16270.1D2	BnaC01g18930.1D2
RS_10_7	BnaA01g16400.1D2	BnaC01g17230.1D2
RS_10_7	BnaA01g16510.1D2	BnaC01g16220.1D2
RS_10_7	BnaA01g16540.1D2	BnaC01g19080.1D2
RS_10_7	BnaA01g16550.1D2	BnaC01g19570.1D2
RS_10_7	BnaA01g16600.1D2	BnaC01g19110.1D2
RS_10_7	BnaA01g16770.1D2	BnaC01g18410.1D2
RS_10_7	BnaA01g16810.1D2	BnaC01g18120.1D2

RS_10_7	BnaA01g16830.1D2	BnaC01g18150.1D2
RS_10_7	BnaA01g16900.1D2	BnaC01g18060.1D2
RS_10_7	BnaA01g17070.1D2	BnaC01g19690.1D2
RS_10_7	BnaA01g17170.1D2	BnaC01g19890.1D2
RS_10_7	BnaA01g17240.1D2	BnaC01g19960.1D2
RS_10_7	BnaA01g17320.1D2	BnaC01g20060.1D2
RS_10_7	BnaA01g17450.1D2	BnaC01g19230.1D2
RS_10_7	BnaA01g17610.1D2	BnaC01g19380.1D2
RS_10_7	BnaA01g17680.1D2	BnaC01g19440.1D2
RS_10_7	BnaA01g17690.1D2	BnaC01g19450.1D2
RS_10_7	BnaA01g17760.1D2	BnaC01g19550.1D2
RS_10_7	BnaA01g17810.1D2	BnaC01g20160.1D2
RS_10_7	BnaA01g17840.1D2	BnaC01g20200.1D2
RS_10_7	BnaA01g17860.1D2	BnaC01g20220.1D2
RS_10_7	BnaA01g17910.1D2	BnaC01g21740.1D2
RS_10_7	BnaA01g17930.1D2	BnaC01g21760.1D2
RS_10_7	BnaA01g17960.1D2	BnaC01g19620.1D2
RS_10_7	BnaA01g17970.1D2	BnaC01g19630.1D2
RS_10_7	BnaA01g17990.1D2	BnaC01g20340.1D2
RS_10_7	BnaA01g18010.1D2	BnaC01g20360.1D2
RS_10_7	BnaA01g18030.1D2	BnaC01g20370.1D2
RS_10_7	BnaA01g18210.1D2	BnaC01g20410.1D2
RS_10_7	BnaA01g18300.1D2	BnaC01g20610.1D2
RS_10_7	BnaA01g18310.1D2	BnaC01g20620.1D2
RS_10_7	BnaA01g18320.1D2	BnaC01g20630.1D2
RS_10_7	BnaA01g18340.1D2	BnaC01g20660.1D2
RS_10_7	BnaA01g18350.1D2	BnaC01g20660.1D2
RS_10_7	BnaA01g18360.1D2	BnaC01g20670.1D2
RS_10_7	BnaA01g18400.1D2	BnaC01g21370.1D2
RS_10_7	BnaA01g18500.1D2	BnaC01g20730.1D2
RS_10_7	BnaA01g18560.1D2	BnaC01g20890.1D2
RS_10_7	BnaA01g18580.1D2	BnaC01g20920.1D2
RS_10_7	BnaA01g18600.1D2	BnaC01g20970.1D2
RS_10_7	BnaA01g18600.1D2	BnaC01g20980.1D2
RS_10_7	BnaA01g18610.1D2	BnaC01g20990.1D2
RS_10_7	BnaA01g18640.1D2	BnaC01g21030.1D2
RS_10_7	BnaA01g18840.1D2	BnaC01g21080.1D2
RS_10_7	BnaA01g18850.1D2	BnaC01g21090.1D2
RS_10_7	BnaA01g18910.1D2	BnaC01g21140.1D2
RS_10_7	BnaA01g18930.1D2	BnaC01g21150.1D2
RS_10_7	BnaA01g18950.1D2	BnaC01g21170.1D2
RS_10_7	BnaA01g18960.1D2	BnaC01g21180.1D2
RS_10_7	BnaA01g18970.1D2	BnaC01g21190.1D2
RS_10_7	BnaA01g18980.1D2	BnaC01g21200.1D2
RS_10_7	BnaA01g19030.1D2	BnaC01g21240.1D2
RS_10_7	BnaA01g19040.1D2	BnaC01g21250.1D2
RS_10_7	BnaA01g19060.1D2	BnaC01g21270.1D2
RS_10_7	BnaA01g19070.1D2	BnaC01g02250.1D2
RS_10_7	BnaA01g19070.1D2	BnaC01g22340.1D2
RS_10_7	BnaA01g19080.1D2	BnaC01g22360.1D2
RS_10_7	BnaA01g19800.1D2	BnaC01g22310.1D2

RS_10_7	BnaA01g19850.1D2	BnaC01g24230.1D2
RS_10_7	BnaA01g19980.1D2	BnaC01g23520.1D2
RS_10_7	BnaA01g20120.1D2	BnaC01g25030.1D2
RS_10_7	BnaA01g20130.1D2	BnaC01g25020.1D2
RS_10_7	BnaA01g20140.1D2	BnaC01g25010.1D2
RS_10_7	BnaA01g20420.1D2	BnaC01g25060.1D2
RS_10_7	BnaA01g20480.1D2	BnaC01g24010.1D2
RS_10_7	BnaA01g20490.1D2	BnaC01g24020.1D2
RS_10_7	BnaA01g20510.1D2	BnaC01g24060.1D2
RS_10_7	BnaA01g20590.1D2	BnaC01g24130.1D2
RS_10_7	BnaA01g20690.1D2	BnaC01g21650.1D2
RS_10_7	BnaA01g20700.1D2	BnaC01g21640.1D2
RS_10_7	BnaA01g20810.1D2	BnaC01g25950.1D2
RS_10_7	BnaA01g20920.1D2	BnaC01g24940.1D2
RS_10_7	BnaA01g21570.1D2	BnaC01g23850.1D2
RS_10_7	BnaA01g21660.1D2	BnaC01g21550.1D2
RS_10_7	BnaA01g21670.1D2	BnaC01g21560.1D2
RS_10_7	BnaA01g21710.1D2	BnaC01g21580.1D2
RS_10_7	BnaA01g21750.1D2	BnaC01g26040.1D2
RS_10_7	BnaA01g21760.1D2	BnaC01g26050.1D2
RS_10_7	BnaA01g21780.1D2	BnaC01g26080.1D2
RS_10_7	BnaA01g21800.1D2	BnaC01g26110.1D2
RS_10_7	BnaA01g21920.1D2	BnaC01g22170.1D2
RS_10_7	BnaA01g21990.1D2	BnaC01g24160.1D2
RS_10_7	BnaA01g22430.1D2	BnaC01g21620.1D2
RS_10_7	BnaA01g24720.1D2	BnaC01g25140.1D2
RS_10_7	BnaA01g24940.1D2	BnaC01g22670.1D2
RS_10_7	BnaA01g35180.1D2	BnaC01g36760.1D2
RS_10_7	BnaA01g35200.1D2	BnaC01g36770.1D2
RS_10_7	BnaA01g35220.1D2	BnaC01g36790.1D2
RS_10_7	BnaA01g35260.1D2	BnaC01g36840.1D2
RS_10_7	BnaA01g35370.1D2	BnaC01g36980.1D2
RS_10_7	BnaA01g35410.1D2	BnaC01g37020.1D2
RS_10_7	BnaA01g35420.1D2	BnaC01g37030.1D2
RS_10_7	BnaA01g35460.1D2	BnaC01g37060.1D2
RS_10_7	BnaA01g35580.1D2	BnaC01g37180.1D2
RS_10_7	BnaA01g35750.1D2	BnaC01g37370.1D2
RS_4_6	BnaA01g05120.1D2	BnaC01g06030.1D2
RS_4_6	BnaA01g07010.1D2	BnaC01g07410.1D2
RS_4_6	BnaA01g07160.1D2	BnaC01g07990.1D2
RS_4_6	BnaA01g08990.1D2	BnaC01g09860.1D2
RS_4_6	BnaA01g09000.1D2	BnaC01g09870.1D2
RS_4_6	BnaA01g09010.1D2	BnaC01g09880.1D2
RS_4_6	BnaA01g09030.1D2	BnaC01g13150.1D2
RS_4_6	BnaA01g09040.1D2	BnaC01g13160.1D2
RS_4_6	BnaA01g09090.1D2	BnaC01g09960.1D2
RS_4_6	BnaA01g09120.1D2	BnaC01g10000.1D2
RS_4_6	BnaA01g09140.1D2	BnaC01g10040.1D2
RS_4_6	BnaA01g09150.1D2	BnaC01g10050.1D2
RS_4_6	BnaA01g09160.1D2	BnaC01g10060.1D2
RS_4_6	BnaA01g09200.1D2	BnaC01g10100.1D2

RS_4_6	BnaA01g09240.1D2	BnaC01g10170.1D2
RS_4_6	BnaA01g09260.1D2	BnaC01g10190.1D2
RS_4_6	BnaA01g09270.1D2	BnaC01g10210.1D2
RS_4_6	BnaA01g09320.1D2	BnaC01g10260.1D2
RS_4_6	BnaA01g09340.1D2	BnaC01g10280.1D2
RS_4_6	BnaA01g09360.1D2	BnaC01g10300.1D2
RS_4_6	BnaA01g09500.1D2	BnaC01g10370.1D2
RS_4_6	BnaA01g09520.1D2	BnaC01g10390.1D2
RS_4_6	BnaA01g09540.1D2	BnaC01g10410.1D2
RS_4_6	BnaA01g09550.1D2	BnaC01g10420.1D2
RS_4_6	BnaA01g09580.1D2	BnaC01g10490.1D2
RS_4_6	BnaA01g09610.1D2	BnaC01g10520.1D2
RS_4_6	BnaA01g09630.1D2	BnaC01g10540.1D2
RS_4_6	BnaA01g09670.1D2	BnaC01g10620.1D2
RS_4_6	BnaA01g09820.1D2	BnaC01g10770.1D2
RS_4_6	BnaA01g09840.1D2	BnaC01g12900.1D2
RS_4_6	BnaA01g09870.1D2	BnaC01g12920.1D2
RS_4_6	BnaA01g09880.1D2	BnaC01g12940.1D2
RS_4_6	BnaA01g09900.1D2	BnaC01g11060.1D2
RS_4_6	BnaA01g09910.1D2	BnaC01g11070.1D2
RS_4_6	BnaA01g09930.1D2	BnaC01g11090.1D2
RS_4_6	BnaA01g10110.1D2	BnaC01g11280.1D2
RS_4_6	BnaA01g10120.1D2	BnaC01g11290.1D2
RS_4_6	BnaA01g10130.1D2	BnaC01g11310.1D2
RS_4_6	BnaA01g10150.1D2	BnaC01g11330.1D2
RS_4_6	BnaA01g10230.1D2	BnaC01g11490.1D2
RS_4_6	BnaA01g10250.1D2	BnaC01g11530.1D2
RS_4_6	BnaA01g10280.1D2	BnaC01g11570.1D2
RS_4_6	BnaA01g10340.1D2	BnaC01g11620.1D2
RS_4_6	BnaA01g10360.1D2	BnaC01g11630.1D2
RS_4_6	BnaA01g10390.1D2	BnaC01g11660.1D2
RS_4_6	BnaA01g10400.1D2	BnaC01g11670.1D2
RS_4_6	BnaA01g10510.1D2	BnaC01g11770.1D2
RS_4_6	BnaA01g10520.1D2	BnaC01g11780.1D2
RS_4_6	BnaA01g10560.1D2	BnaC01g11800.1D2
RS_4_6	BnaA01g10570.1D2	BnaC01g11810.1D2
RS_4_6	BnaA01g10730.1D2	BnaC01g11980.1D2
RS_4_6	BnaA01g10790.1D2	BnaC01g12020.1D2
RS_4_6	BnaA01g10850.1D2	BnaC01g12120.1D2
RS_4_6	BnaA01g10870.1D2	BnaC01g12140.1D2
RS_4_6	BnaA01g10980.1D2	BnaC01g12230.1D2
RS_4_6	BnaA01g11000.1D2	BnaC01g12280.1D2
RS_4_6	BnaA01g11030.1D2	BnaC01g12330.1D2
RS_4_6	BnaA01g11070.1D2	BnaC01g12360.1D2
RS_4_6	BnaA01g11110.1D2	BnaC01g12390.1D2
RS_4_6	BnaA01g11180.1D2	BnaC01g12470.1D2
RS_4_6	BnaA01g11190.1D2	BnaC01g12480.1D2
RS_4_6	BnaA01g11250.1D2	BnaC01g12520.1D2
RS_4_6	BnaA01g11260.1D2	BnaC01g12530.1D2
RS_4_6	BnaA01g11320.1D2	BnaC01g12600.1D2
RS_4_6	BnaA01g11350.1D2	BnaC01g12630.1D2

RS_4_6	BnaA01g11360.1D2	BnaC01g12640.1D2
RS_4_6	BnaA01g11370.1D2	BnaC01g12650.1D2
RS_4_6	BnaA01g11400.1D2	BnaC01g12710.1D2
RS_4_6	BnaA01g11430.1D2	BnaC01g12730.1D2
RS_4_6	BnaA01g11440.1D2	BnaC01g12750.1D2
RS_4_6	BnaA01g11530.1D2	BnaC01g15620.1D2
RS_4_6	BnaA01g11580.1D2	BnaC01g12980.1D2
RS_4_6	BnaA01g11700.1D2	BnaC01g10460.1D2
RS_4_6	BnaA01g11770.1D2	BnaC01g11750.1D2
RS_4_6	BnaA01g11870.1D2	BnaC01g12850.1D2
RS_4_6	BnaA01g11900.1D2	BnaC01g13240.1D2
RS_4_6	BnaA01g11920.1D2	BnaC01g13260.1D2
RS_4_6	BnaA01g11930.1D2	BnaC01g13260.1D2
RS_4_6	BnaA01g11940.1D2	BnaC01g13280.1D2
RS_4_6	BnaA01g11970.1D2	BnaC01g13300.1D2
RS_4_6	BnaA01g11980.1D2	BnaC01g13310.1D2
RS_4_6	BnaA01g11990.1D2	BnaC01g13320.1D2
RS_4_6	BnaA01g12100.1D2	BnaC01g13390.1D2
RS_4_6	BnaA01g12140.1D2	BnaC01g13420.1D2
RS_4_6	BnaA01g12160.1D2	BnaC01g13440.1D2
RS_4_6	BnaA01g12190.1D2	BnaC01g15640.1D2
RS_4_6	BnaA01g12200.1D2	BnaC01g15650.1D2
RS_4_6	BnaA01g12420.1D2	BnaC01g13700.1D2
RS_4_6	BnaA01g12430.1D2	BnaC01g13710.1D2
RS_4_6	BnaA01g12440.1D2	BnaC01g13720.1D2
RS_4_6	BnaA01g12610.1D2	BnaC01g13740.1D2
RS_4_6	BnaA01g12630.1D2	BnaC01g14070.1D2
RS_4_6	BnaA01g12650.1D2	BnaC01g14130.1D2
RS_4_6	BnaA01g12670.1D2	BnaC01g14150.1D2
RS_4_6	BnaA01g12680.1D2	BnaC01g13730.1D2
RS_4_6	BnaA01g12690.1D2	BnaC01g14200.1D2
RS_4_6	BnaA01g12710.1D2	BnaC01g14210.1D2
RS_4_6	BnaA01g12740.1D2	BnaC01g14220.1D2
RS_4_6	BnaA01g12760.1D2	BnaC01g14250.1D2
RS_4_6	BnaA01g12780.1D2	BnaC01g14270.1D2
RS_4_6	BnaA01g12790.1D2	BnaC01g14280.1D2
RS_4_6	BnaA01g12810.1D2	BnaC01g14300.1D2
RS_4_6	BnaA01g12820.1D2	BnaC01g14310.1D2
RS_4_6	BnaA01g12840.1D2	BnaC01g14330.1D2
RS_4_6	BnaA01g12850.1D2	BnaC01g14340.1D2
RS_4_6	BnaA01g12930.1D2	BnaC01g14420.1D2
RS_4_6	BnaA01g12950.1D2	BnaC01g14190.1D2
RS_4_6	BnaA01g12960.1D2	BnaC01g14520.1D2
RS_4_6	BnaA01g13000.1D2	BnaC01g14580.1D2
RS_4_6	BnaA01g13010.1D2	BnaC01g14590.1D2
RS_4_6	BnaA01g13040.1D2	BnaC01g14620.1D2
RS_4_6	BnaA01g13120.1D2	BnaC01g14700.1D2
RS_4_6	BnaA01g13130.1D2	BnaC01g14710.1D2
RS_4_6	BnaA01g13170.1D2	BnaC01g14760.1D2
RS_4_6	BnaA01g13190.1D2	BnaC01g14780.1D2
RS_4_6	BnaA01g13220.1D2	BnaC01g14800.1D2

RS_4_6	BnaA01g13270.1D2	BnaC01g15580.1D2
RS_4_6	BnaA01g13310.1D2	BnaC01g14870.1D2
RS_4_6	BnaA01g13320.1D2	BnaC01g14900.1D2
RS_4_6	BnaA01g13330.1D2	BnaC01g14910.1D2
RS_4_6	BnaA01g13360.1D2	BnaC01g14950.1D2
RS_4_6	BnaA01g13470.1D2	BnaC01g15060.1D2
RS_4_6	BnaA01g13480.1D2	BnaC01g15070.1D2
RS_4_6	BnaA01g13490.1D2	BnaC01g15080.1D2
RS_4_6	BnaA01g13650.1D2	BnaC01g15220.1D2
RS_4_6	BnaA01g13720.1D2	BnaC01g15290.1D2
RS_4_6	BnaA01g13730.1D2	BnaC01g15300.1D2
RS_4_6	BnaA01g13750.1D2	BnaC01g15320.1D2
RS_4_6	BnaA01g13780.1D2	BnaC01g15350.1D2
RS_4_6	BnaA01g13830.1D2	BnaC01g15400.1D2
RS_4_6	BnaA01g13910.1D2	BnaC01g14460.1D2
RS_4_6	BnaA01g13920.1D2	BnaC01g14450.1D2
RS_4_6	BnaA01g13930.1D2	BnaC01g15490.1D2
RS_4_6	BnaA01g13940.1D2	BnaC01g15500.1D2
RS_4_6	BnaA01g13950.1D2	BnaC01g15520.1D2
RS_4_6	BnaA01g14110.1D2	BnaC01g15910.1D2
RS_4_6	BnaA01g14220.1D2	BnaC01g16060.1D2
RS_4_6	BnaA01g14240.1D2	BnaC01g16090.1D2
RS_4_6	BnaA01g14270.1D2	BnaC01g16120.1D2
RS_4_6	BnaA01g14310.1D2	BnaC01g16150.1D2
RS_4_6	BnaA01g14340.1D2	BnaC01g16210.1D2
RS_4_6	BnaA01g14410.1D2	BnaC01g16330.1D2
RS_4_6	BnaA01g14420.1D2	BnaC01g16330.1D2
RS_4_6	BnaA01g14440.1D2	BnaC01g16350.1D2
RS_4_6	BnaA01g14530.1D2	BnaC01g16450.1D2
RS_4_6	BnaA01g14550.1D2	BnaC01g17290.1D2
RS_4_6	BnaA01g14610.1D2	BnaC01g16520.1D2
RS_4_6	BnaA01g14620.1D2	BnaC01g16530.1D2
RS_4_6	BnaA01g14670.1D2	BnaC01g16610.1D2
RS_4_6	BnaA01g14730.1D2	BnaC01g16690.1D2
RS_4_6	BnaA01g14820.1D2	BnaC01g16770.1D2
RS_4_6	BnaA01g14830.1D2	BnaC01g16790.1D2
RS_4_6	BnaA01g14850.1D2	BnaC01g16800.1D2
RS_4_6	BnaA01g14920.1D2	BnaC01g16910.1D2
RS_4_6	BnaA01g14950.1D2	BnaC01g16940.1D2
RS_4_6	BnaA01g14990.1D2	BnaC01g17030.1D2
RS_4_6	BnaA01g15030.1D2	BnaC01g17080.1D2
RS_4_6	BnaA01g15120.1D2	BnaC01g17190.1D2
RS_4_6	BnaA01g15130.1D2	BnaC01g17200.1D2
RS_4_6	BnaA01g15220.1D2	BnaC01g17420.1D2
RS_4_6	BnaA01g15360.1D2	BnaC01g17600.1D2
RS_4_6	BnaA01g15380.1D2	BnaC01g17620.1D2
RS_4_6	BnaA01g15410.1D2	BnaC01g17630.1D2
RS_4_6	BnaA01g15410.1D2	BnaC01g17650.1D2
RS_4_6	BnaA01g15410.1D2	BnaC01g17660.1D2
RS_4_6	BnaA01g15420.1D2	BnaC01g17630.1D2
RS_4_6	BnaA01g15420.1D2	BnaC01g17650.1D2

RS_4_6	BnaA01g15420.1D2	BnaC01g17660.1D2
RS_4_6	BnaA01g15430.1D2	BnaC01g17630.1D2
RS_4_6	BnaA01g15430.1D2	BnaC01g17650.1D2
RS_4_6	BnaA01g15430.1D2	BnaC01g17660.1D2
RS_4_6	BnaA01g15430.1D2	BnaC01g22150.1D2
RS_4_6	BnaA01g15500.1D2	BnaC01g31050.1D2
RS_4_6	BnaA01g15600.1D2	BnaC01g17780.1D2
RS_4_6	BnaA01g15650.1D2	BnaC01g17820.1D2
RS_4_6	BnaA01g15660.1D2	BnaC01g17830.1D2
RS_4_6	BnaA01g15750.1D2	BnaC01g17930.1D2
RS_4_6	BnaA01g15790.1D2	BnaC01g17950.1D2
RS_4_6	BnaA01g15800.1D2	BnaC01g17960.1D2
RS_4_6	BnaA01g15830.1D2	BnaC01g18290.1D2
RS_4_6	BnaA01g16020.1D2	BnaC01g18720.1D2
RS_4_6	BnaA01g16050.1D2	BnaC01g25450.1D2
RS_4_6	BnaA01g16070.1D2	BnaC01g18740.1D2
RS_4_6	BnaA01g16080.1D2	BnaC01g18750.1D2
RS_4_6	BnaA01g16110.1D2	BnaC01g18790.1D2
RS_4_6	BnaA01g16120.1D2	BnaC01g18800.1D2
RS_4_6	BnaA01g16130.1D2	BnaC01g18810.1D2
RS_4_6	BnaA01g16180.1D2	BnaC01g18850.1D2
RS_4_6	BnaA01g16190.1D2	BnaC01g18860.1D2
RS_4_6	BnaA01g16210.1D2	BnaC01g18880.1D2
RS_4_6	BnaA01g16220.1D2	BnaC01g18890.1D2
RS_4_6	BnaA01g16230.1D2	BnaC01g18900.1D2
RS_4_6	BnaA01g16250.1D2	BnaC01g18920.1D2
RS_4_6	BnaA01g16270.1D2	BnaC01g18930.1D2
RS_4_6	BnaA01g16340.1D2	BnaC01g16550.1D2
RS_4_6	BnaA01g16400.1D2	BnaC01g17230.1D2
RS_4_6	BnaA01g16410.1D2	BnaC01g15980.1D2
RS_4_6	BnaA01g16510.1D2	BnaC01g16220.1D2
RS_4_6	BnaA01g16540.1D2	BnaC01g19080.1D2
RS_4_6	BnaA01g16600.1D2	BnaC01g19110.1D2
RS_4_6	BnaA01g16680.1D2	BnaC01g18530.1D2
RS_4_6	BnaA01g16710.1D2	BnaC01g18560.1D2
RS_4_6	BnaA01g16710.1D2	BnaC01g18570.1D2
RS_4_6	BnaA01g16750.1D2	BnaC01g18390.1D2
RS_4_6	BnaA01g16760.1D2	BnaC01g18400.1D2
RS_4_6	BnaA01g16770.1D2	BnaC01g18410.1D2
RS_4_6	BnaA01g16810.1D2	BnaC01g18120.1D2
RS_4_6	BnaA01g16830.1D2	BnaC01g18150.1D2
RS_4_6	BnaA01g16900.1D2	BnaC01g18060.1D2
RS_4_6	BnaA01g17070.1D2	BnaC01g19690.1D2
RS_4_6	BnaA01g17170.1D2	BnaC01g19890.1D2
RS_4_6	BnaA01g17240.1D2	BnaC01g19960.1D2
RS_4_6	BnaA01g17310.1D2	BnaC01g20020.1D2
RS_4_6	BnaA01g17320.1D2	BnaC01g20060.1D2
RS_4_6	BnaA01g17450.1D2	BnaC01g19230.1D2
RS_4_6	BnaA01g17580.1D2	BnaC01g19360.1D2
RS_4_6	BnaA01g17610.1D2	BnaC01g19380.1D2
RS_4_6	BnaA01g17620.1D2	BnaC01g19390.1D2

RS_4_6	BnaA01g17680.1D2	BnaC01g19440.1D2
RS_4_6	BnaA01g17690.1D2	BnaC01g19450.1D2
RS_4_6	BnaA01g17760.1D2	BnaC01g19550.1D2
RS_4_6	BnaA01g17810.1D2	BnaC01g20160.1D2
RS_4_6	BnaA01g17840.1D2	BnaC01g20200.1D2
RS_4_6	BnaA01g17860.1D2	BnaC01g20220.1D2
RS_4_6	BnaA01g17890.1D2	BnaC01g20260.1D2
RS_4_6	BnaA01g17910.1D2	BnaC01g21740.1D2
RS_4_6	BnaA01g17930.1D2	BnaC01g21760.1D2
RS_4_6	BnaA01g17960.1D2	BnaC01g19620.1D2
RS_4_6	BnaA01g17970.1D2	BnaC01g19630.1D2
RS_4_6	BnaA01g17990.1D2	BnaC01g20340.1D2
RS_4_6	BnaA01g18010.1D2	BnaC01g20360.1D2
RS_4_6	BnaA01g18090.1D2	BnaC01g28160.1D2
RS_4_6	BnaA01g18110.1D2	BnaC01g21730.1D2
RS_4_6	BnaA01g18220.1D2	BnaC01g20420.1D2
RS_4_6	BnaA01g18300.1D2	BnaC01g20610.1D2
RS_4_6	BnaA01g18310.1D2	BnaC01g20620.1D2
RS_4_6	BnaA01g18320.1D2	BnaC01g20630.1D2
RS_4_6	BnaA01g18340.1D2	BnaC01g20660.1D2
RS_4_6	BnaA01g18350.1D2	BnaC01g20660.1D2
RS_4_6	BnaA01g18360.1D2	BnaC01g20670.1D2
RS_4_6	BnaA01g18400.1D2	BnaC01g21370.1D2
RS_4_6	BnaA01g18450.1D2	BnaC01g20830.1D2
RS_4_6	BnaA01g18500.1D2	BnaC01g20730.1D2
RS_4_6	BnaA01g18560.1D2	BnaC01g20890.1D2
RS_4_6	BnaA01g18580.1D2	BnaC01g20920.1D2
RS_4_6	BnaA01g18590.1D2	BnaC01g20930.1D2
RS_4_6	BnaA01g18600.1D2	BnaC01g20970.1D2
RS_4_6	BnaA01g18600.1D2	BnaC01g20980.1D2
RS_4_6	BnaA01g18610.1D2	BnaC01g20990.1D2
RS_4_6	BnaA01g18640.1D2	BnaC01g21030.1D2
RS_4_6	BnaA01g18710.1D2	BnaC01g21800.1D2
RS_4_6	BnaA01g18810.1D2	BnaC01g25270.1D2
RS_4_6	BnaA01g18850.1D2	BnaC01g21090.1D2
RS_4_6	BnaA01g18900.1D2	BnaC01g21130.1D2
RS_4_6	BnaA01g18910.1D2	BnaC01g21140.1D2
RS_4_6	BnaA01g18930.1D2	BnaC01g21150.1D2
RS_4_6	BnaA01g18940.1D2	BnaC01g21160.1D2
RS_4_6	BnaA01g18950.1D2	BnaC01g21170.1D2
RS_4_6	BnaA01g18960.1D2	BnaC01g21180.1D2
RS_4_6	BnaA01g18970.1D2	BnaC01g21190.1D2
RS_4_6	BnaA01g18980.1D2	BnaC01g21200.1D2
RS_4_6	BnaA01g18990.1D2	BnaC01g21200.1D2
RS_4_6	BnaA01g19000.1D2	BnaC01g21210.1D2
RS_4_6	BnaA01g19030.1D2	BnaC01g21240.1D2
RS_4_6	BnaA01g19040.1D2	BnaC01g21250.1D2
RS_4_6	BnaA01g19060.1D2	BnaC01g21270.1D2
RS_4_6	BnaA01g19070.1D2	BnaC01g22340.1D2
RS_4_6	BnaA01g19080.1D2	BnaC01g22360.1D2
RS_4_6	BnaA01g19800.1D2	BnaC01g22310.1D2

RS_4_6	BnaA01g19850.1D2	BnaC01g24230.1D2
RS_4_6	BnaA01g19960.1D2	BnaC01g21790.1D2
RS_4_6	BnaA01g19980.1D2	BnaC01g23520.1D2
RS_4_6	BnaA01g20040.1D2	BnaC01g23580.1D2
RS_4_6	BnaA01g20070.1D2	BnaC01g23620.1D2
RS_4_6	BnaA01g20080.1D2	BnaC01g23630.1D2
RS_4_6	BnaA01g20110.1D2	BnaC01g25030.1D2
RS_4_6	BnaA01g20120.1D2	BnaC01g25030.1D2
RS_4_6	BnaA01g20130.1D2	BnaC01g25020.1D2
RS_4_6	BnaA01g20330.1D2	BnaC01g24550.1D2
RS_4_6	BnaA01g20340.1D2	BnaC01g23920.1D2
RS_4_6	BnaA01g20420.1D2	BnaC01g25060.1D2
RS_4_6	BnaA01g20480.1D2	BnaC01g24010.1D2
RS_4_6	BnaA01g20490.1D2	BnaC01g24020.1D2
RS_4_6	BnaA01g20510.1D2	BnaC01g24060.1D2
RS_4_6	BnaA01g20520.1D2	BnaC01g24070.1D2
RS_4_6	BnaA01g20670.1D2	BnaC01g21670.1D2
RS_4_6	BnaA01g20690.1D2	BnaC01g21650.1D2
RS_4_6	BnaA01g20700.1D2	BnaC01g21640.1D2
RS_4_6	BnaA01g20720.1D2	BnaC01g21820.1D2
RS_4_6	BnaA01g20810.1D2	BnaC01g25950.1D2
RS_4_6	BnaA01g20860.1D2	BnaC01g24910.1D2
RS_4_6	BnaA01g21120.1D2	BnaC01g25470.1D2
RS_4_6	BnaA01g21360.1D2	BnaC01g25970.1D2
RS_4_6	BnaA01g21440.1D2	BnaC01g25110.1D2
RS_4_6	BnaA01g21570.1D2	BnaC01g23850.1D2
RS_4_6	BnaA01g21660.1D2	BnaC01g21550.1D2
RS_4_6	BnaA01g21710.1D2	BnaC01g21580.1D2
RS_4_6	BnaA01g21750.1D2	BnaC01g26040.1D2
RS_4_6	BnaA01g21760.1D2	BnaC01g26050.1D2
RS_4_6	BnaA01g21770.1D2	BnaC01g26060.1D2
RS_4_6	BnaA01g21780.1D2	BnaC01g26080.1D2
RS_4_6	BnaA01g21800.1D2	BnaC01g26110.1D2
RS_4_6	BnaA01g21890.1D2	BnaC01g25800.1D2
RS_4_6	BnaA01g21920.1D2	BnaC01g22170.1D2
RS_4_6	BnaA01g21990.1D2	BnaC01g24160.1D2
RS_4_6	BnaA01g22090.1D2	BnaC01g26260.1D2
RS_4_6	BnaA01g22140.1D2	BnaC01g26330.1D2
RS_4_6	BnaA01g22190.1D2	BnaC01g31100.1D2
RS_4_6	BnaA01g22290.1D2	BnaC01g26830.1D2
RS_4_6	BnaA01g22350.1D2	BnaC01g26630.1D2
RS_4_6	BnaA01g22370.1D2	BnaC01g26610.1D2
RS_4_6	BnaA01g22430.1D2	BnaC01g21620.1D2
RS_4_6	BnaA01g22650.1D2	BnaC01g20430.1D2
RS_4_6	BnaA01g22820.1D2	BnaC01g26900.1D2
RS_4_6	BnaA01g22830.1D2	BnaC01g26910.1D2
RS_4_6	BnaA01g22890.1D2	BnaC01g26980.1D2
RS_4_6	BnaA01g22910.1D2	BnaC01g27020.1D2
RS_4_6	BnaA01g22940.1D2	BnaC01g25750.1D2
RS_4_6	BnaA01g22990.1D2	BnaC01g22600.1D2
RS_4_6	BnaA01g23040.1D2	BnaC01g22140.1D2

RS_4_6	BnaA01g23110.1D2	BnaC01g26480.1D2
RS_4_6	BnaA01g23120.1D2	BnaC01g26490.1D2
RS_4_6	BnaA01g23170.1D2	BnaC01g26540.1D2
RS_4_6	BnaA01g23190.1D2	BnaC01g26550.1D2
RS_4_6	BnaA01g23250.1D2	BnaC01g27190.1D2
RS_4_6	BnaA01g23500.1D2	BnaC01g22110.1D2
RS_4_6	BnaA01g23520.1D2	BnaC01g21480.1D2
RS_4_6	BnaA01g23650.1D2	BnaC01g28000.1D2
RS_4_6	BnaA01g23670.1D2	BnaC01g28020.1D2
RS_4_6	BnaA01g23720.1D2	BnaC01g21860.1D2
RS_4_6	BnaA01g23750.1D2	BnaC01g27490.1D2
RS_4_6	BnaA01g23800.1D2	BnaC01g27570.1D2
RS_4_6	BnaA01g23930.1D2	BnaC01g27710.1D2
RS_4_6	BnaA01g24110.1D2	BnaC01g28050.1D2
RS_4_6	BnaA01g24150.1D2	BnaC01g28100.1D2
RS_4_6	BnaA01g24180.1D2	BnaC01g29320.1D2
RS_4_6	BnaA01g24190.1D2	BnaC01g29340.1D2
RS_4_6	BnaA01g24240.1D2	BnaC01g18220.1D2
RS_4_6	BnaA01g24370.1D2	BnaC01g28130.1D2
RS_4_6	BnaA01g24420.1D2	BnaC01g28990.1D2
RS_4_6	BnaA01g24430.1D2	BnaC01g28980.1D2
RS_4_6	BnaA01g24440.1D2	BnaC01g28950.1D2
RS_4_6	BnaA01g24440.1D2	BnaC01g28960.1D2
RS_4_6	BnaA01g24490.1D2	BnaC01g31030.1D2
RS_4_6	BnaA01g24520.1D2	BnaC01g30990.1D2
RS_4_6	BnaA01g24550.1D2	BnaC01g30960.1D2
RS_4_6	BnaA01g24590.1D2	BnaC01g28240.1D2
RS_4_6	BnaA01g24610.1D2	BnaC01g28290.1D2
RS_4_6	BnaA01g24720.1D2	BnaC01g25140.1D2
RS_4_6	BnaA01g24750.1D2	BnaC01g28360.1D2
RS_4_6	BnaA01g24790.1D2	BnaC01g20480.1D2
RS_4_6	BnaA01g24870.1D2	BnaC01g28440.1D2
RS_4_6	BnaA01g24920.1D2	BnaC01g25440.1D2
RS_4_6	BnaA01g24940.1D2	BnaC01g22670.1D2
RS_4_6	BnaA01g25060.1D2	BnaC01g28560.1D2
RS_4_6	BnaA01g25220.1D2	BnaC01g29030.1D2
RS_4_6	BnaA01g25290.1D2	BnaC01g29100.1D2
RS_4_6	BnaA01g25350.1D2	BnaC01g29170.1D2
RS_4_6	BnaA01g25360.1D2	BnaC01g29180.1D2
RS_4_6	BnaA01g25560.1D2	BnaC01g29390.1D2
RS_4_6	BnaA01g25600.1D2	BnaC01g31040.1D2
RS_4_6	BnaA01g25610.1D2	BnaC01g31050.1D2
RS_4_6	BnaA01g25730.1D2	BnaC01g29630.1D2
RS_4_6	BnaA01g25790.1D2	BnaC01g29790.1D2
RS_4_6	BnaA01g25800.1D2	BnaC01g29780.1D2
RS_4_6	BnaA01g25850.1D2	BnaC01g29810.1D2
RS_4_6	BnaA01g25860.1D2	BnaC01g29820.1D2
RS_4_6	BnaA01g25890.1D2	BnaC01g29870.1D2
RS_4_6	BnaA01g25910.1D2	BnaC01g29890.1D2
RS_4_6	BnaA01g25960.1D2	BnaC01g29930.1D2
RS_4_6	BnaA01g26040.1D2	BnaC01g30010.1D2

RS_4_6	BnaA01g26110.1D2	BnaC01g30110.1D2
RS_4_6	BnaA01g26120.1D2	BnaC01g30120.1D2
RS_4_6	BnaA01g26130.1D2	BnaC01g30900.1D2
RS_4_6	BnaA01g26150.1D2	BnaC01g30920.1D2
RS_4_6	BnaA01g26160.1D2	BnaC01g30920.1D2
RS_4_6	BnaA01g26260.1D2	BnaC01g30270.1D2
RS_4_6	BnaA01g26320.1D2	BnaC01g30360.1D2
RS_4_6	BnaA01g26330.1D2	BnaC01g30360.1D2
RS_4_6	BnaA01g26340.1D2	BnaC01g30360.1D2
RS_4_6	BnaA01g26370.1D2	BnaC01g30380.1D2
RS_4_6	BnaA01g26400.1D2	BnaC01g30410.1D2
RS_4_6	BnaA01g26410.1D2	BnaC01g30430.1D2
RS_4_6	BnaA01g26430.1D2	BnaC01g32940.1D2
RS_4_6	BnaA01g26540.1D2	BnaC01g30480.1D2
RS_4_6	BnaA01g26550.1D2	BnaC01g30490.1D2
RS_4_6	BnaA01g26560.1D2	BnaC01g30500.1D2
RS_4_6	BnaA01g26590.1D2	BnaC01g30530.1D2
RS_4_6	BnaA01g26600.1D2	BnaC01g30540.1D2
RS_4_6	BnaA01g26610.1D2	BnaC01g30550.1D2
RS_4_6	BnaA01g26630.1D2	BnaC01g30570.1D2
RS_4_6	BnaA01g26640.1D2	BnaC01g30580.1D2
RS_4_6	BnaA01g26650.1D2	BnaC01g30590.1D2
RS_4_6	BnaA01g26660.1D2	BnaC01g30590.1D2
RS_4_6	BnaA01g26700.1D2	BnaC01g30670.1D2
RS_4_6	BnaA01g26710.1D2	BnaC01g30660.1D2
RS_4_6	BnaA01g26880.1D2	BnaC01g30800.1D2
RS_4_6	BnaA01g26890.1D2	BnaC01g30800.1D2
RS_4_6	BnaA01g26920.1D2	BnaC01g30820.1D2
RS_4_6	BnaA01g26990.1D2	BnaC01g32260.1D2
RS_4_6	BnaA01g27050.1D2	BnaC01g31270.1D2
RS_4_6	BnaA01g27100.1D2	BnaC01g31290.1D2
RS_4_6	BnaA01g27140.1D2	BnaC01g31330.1D2
RS_4_6	BnaA01g27180.1D2	BnaC01g31340.1D2
RS_4_6	BnaA01g27190.1D2	BnaC01g31350.1D2
RS_4_6	BnaA01g27210.1D2	BnaC01g32160.1D2
RS_4_6	BnaA01g27220.1D2	BnaC01g32150.1D2
RS_4_6	BnaA01g27260.1D2	BnaC01g32110.1D2
RS_4_6	BnaA01g27280.1D2	BnaC01g31530.1D2
RS_4_6	BnaA01g27440.1D2	BnaC01g35140.1D2
RS_4_6	BnaA01g27500.1D2	BnaC01g33590.1D2
RS_4_6	BnaA01g27570.1D2	BnaC01g31430.1D2
RS_4_6	BnaA01g27660.1D2	BnaC01g32010.1D2
RS_4_6	BnaA01g27730.1D2	BnaC01g33080.1D2
RS_4_6	BnaA01g27780.1D2	BnaC01g31820.1D2
RS_4_6	BnaA01g27870.1D2	BnaC01g31860.1D2
RS_4_6	BnaA01g27870.1D2	BnaC01g32180.1D2
RS_4_6	BnaA01g27880.1D2	BnaC01g31870.1D2
RS_4_6	BnaA01g27900.1D2	BnaC01g31920.1D2
RS_4_6	BnaA01g27960.1D2	BnaC01g32970.1D2
RS_4_6	BnaA01g28020.1D2	BnaC01g32220.1D2
RS_4_6	BnaA01g28060.1D2	BnaC01g32280.1D2

RS_4_6	BnaA01g28090.1D2	BnaC01g32300.1D2
RS_4_6	BnaA01g28100.1D2	BnaC01g32310.1D2
RS_4_6	BnaA01g28200.1D2	BnaC01g32410.1D2
RS_4_6	BnaA01g28210.1D2	BnaC01g32420.1D2
RS_4_6	BnaA01g28260.1D2	BnaC01g32480.1D2
RS_4_6	BnaA01g28280.1D2	BnaC01g32500.1D2
RS_4_6	BnaA01g28290.1D2	BnaC01g32510.1D2
RS_4_6	BnaA01g28400.1D2	BnaC01g33140.1D2
RS_4_6	BnaA01g28430.1D2	BnaC01g32770.1D2
RS_4_6	BnaA01g28440.1D2	BnaC01g32760.1D2
RS_4_6	BnaA01g28450.1D2	BnaC01g32750.1D2
RS_4_6	BnaA01g28470.1D2	BnaC01g32730.1D2
RS_4_6	BnaA01g28480.1D2	BnaC01g32720.1D2
RS_4_6	BnaA01g28540.1D2	BnaC01g32670.1D2
RS_4_6	BnaA01g28570.1D2	BnaC01g32640.1D2
RS_4_6	BnaA01g28650.1D2	BnaC01g33250.1D2
RS_4_6	BnaA01g28660.1D2	BnaC01g33270.1D2
RS_4_6	BnaA01g28720.1D2	BnaC01g33340.1D2
RS_4_6	BnaA01g28740.1D2	BnaC01g33370.1D2
RS_4_6	BnaA01g28750.1D2	BnaC01g33380.1D2
RS_4_6	BnaA01g28760.1D2	BnaC01g33390.1D2
RS_4_6	BnaA01g28790.1D2	BnaC01g33430.1D2
RS_4_6	BnaA01g28990.1D2	BnaC01g33010.1D2
RS_4_6	BnaA01g30680.1D2	BnaC01g33620.1D2
RS_4_6	BnaA01g30690.1D2	BnaC01g33630.1D2
RS_4_6	BnaA01g30700.1D2	BnaC01g33640.1D2
RS_4_6	BnaA01g30700.1D2	BnaC01g33650.1D2
RS_4_6	BnaA01g30720.1D2	BnaC01g33660.1D2
RS_4_6	BnaA01g30760.1D2	BnaC01g33690.1D2
RS_4_6	BnaA01g30800.1D2	BnaC01g32200.1D2
RS_4_6	BnaA01g30810.1D2	BnaC01g33800.1D2
RS_4_6	BnaA01g30890.1D2	BnaC01g33860.1D2
RS_4_6	BnaA01g30920.1D2	BnaC01g33880.1D2
RS_4_6	BnaA01g30970.1D2	BnaC01g33940.1D2
RS_4_6	BnaA01g30980.1D2	BnaC01g33950.1D2
RS_4_6	BnaA01g30990.1D2	BnaC01g33970.1D2
RS_4_6	BnaA01g31010.1D2	BnaC01g34180.1D2
RS_4_6	BnaA01g31030.1D2	BnaC01g35130.1D2
RS_4_6	BnaA01g31050.1D2	BnaC01g33780.1D2
RS_4_6	BnaA01g31060.1D2	BnaC01g33770.1D2
RS_4_6	BnaA01g31080.1D2	BnaC01g33730.1D2
RS_4_6	BnaA01g31090.1D2	BnaC01g33710.1D2
RS_4_6	BnaA01g31090.1D2	BnaC01g33720.1D2
RS_4_6	BnaA01g31130.1D2	BnaC01g34550.1D2
RS_4_6	BnaA01g31170.1D2	BnaC01g34620.1D2
RS_4_6	BnaA01g31240.1D2	BnaC01g34700.1D2
RS_4_6	BnaA01g31260.1D2	BnaC01g34720.1D2
RS_4_6	BnaA01g31440.1D2	BnaC01g34970.1D2
RS_4_6	BnaA01g31550.1D2	BnaC01g35060.1D2
RS_4_6	BnaA01g31680.1D2	BnaC01g34330.1D2
RS_4_6	BnaA01g31710.1D2	BnaC01g34270.1D2

RS_4_6	BnaA01g31720.1D2	BnaC01g34260.1D2
RS_4_6	BnaA01g32240.1D2	BnaC01g35400.1D2
RS_4_6	BnaA01g32280.1D2	BnaC01g35370.1D2
RS_4_6	BnaA01g32370.1D2	BnaC01g35290.1D2
RS_4_6	BnaA01g32380.1D2	BnaC01g35270.1D2
RS_4_6	BnaA01g32400.1D2	BnaC01g35250.1D2
RS_4_6	BnaA01g32610.1D2	BnaC01g34010.1D2
RS_4_6	BnaA01g32690.1D2	BnaC01g34110.1D2
RS_4_6	BnaA01g32700.1D2	BnaC01g34120.1D2
RS_4_6	BnaA01g33630.1D2	BnaC01g35490.1D2
RS_4_6	BnaA01g33670.1D2	BnaC01g35520.1D2
RS_4_6	BnaA01g33690.1D2	BnaC01g35540.1D2
RS_4_6	BnaA01g33730.1D2	BnaC01g35570.1D2
RS_4_6	BnaA01g33760.1D2	BnaC01g35590.1D2
RS_4_6	BnaA01g33780.1D2	BnaC01g35640.1D2
RS_4_6	BnaA01g33800.1D2	BnaC01g35670.1D2
RS_4_6	BnaA01g33840.1D2	BnaC01g35710.1D2
RS_4_6	BnaA01g33880.1D2	BnaC01g35750.1D2
RS_4_6	BnaA01g33960.1D2	BnaC01g36030.1D2
RS_4_6	BnaA01g33970.1D2	BnaC01g36040.1D2
RS_4_6	BnaA01g35470.1D2	BnaC01g37100.1D2
RS_4_6	BnaA01g35470.1D2	BnaC01g37120.1D2

List of HE genes (A02 to C02)

Accession	Lost gene	Duplicated HE gene
RS_10_7	BnaA02g04830.1D2	BnaC02g33250.1D2
RS_10_7	BnaA02g23430.1D2	BnaC02g29390.1D2
RS_10_7	BnaA02g24340.1D2	BnaC02g30180.1D2
RS_10_7	BnaA02g24560.1D2	BnaC02g30500.1D2
RS_10_7	BnaA02g24690.1D2	BnaC02g30610.1D2
RS_10_7	BnaA02g24740.1D2	BnaC02g30660.1D2
RS_10_7	BnaA02g24780.1D2	BnaC02g30670.1D2
RS_10_7	BnaA02g24900.1D2	BnaC02g31690.1D2
RS_10_7	BnaA02g24910.1D2	BnaC02g31710.1D2
RS_10_7	BnaA02g24940.1D2	BnaC02g31780.1D2
RS_10_7	BnaA02g25020.1D2	BnaC02g31380.1D2
RS_10_7	BnaA02g25070.1D2	BnaC02g30770.1D2
RS_10_7	BnaA02g25170.1D2	BnaC02g31930.1D2
RS_10_7	BnaA02g25190.1D2	BnaC02g31950.1D2
RS_10_7	BnaA02g25260.1D2	BnaC02g32020.1D2
RS_10_7	BnaA02g25440.1D2	BnaC02g31590.1D2
RS_10_7	BnaA02g25450.1D2	BnaC02g31580.1D2
RS_10_7	BnaA02g25460.1D2	BnaC02g31570.1D2
RS_10_7	BnaA02g25490.1D2	BnaC02g31560.1D2
RS_10_7	BnaA02g25510.1D2	BnaC02g31550.1D2
RS_10_7	BnaA02g25520.1D2	BnaC02g31540.1D2
RS_10_7	BnaA02g25550.1D2	BnaC02g32040.1D2
RS_10_7	BnaA02g25610.1D2	BnaC02g32100.1D2
RS_10_7	BnaA02g25640.1D2	BnaC02g32130.1D2
RS_10_7	BnaA02g25650.1D2	BnaC02g32140.1D2
RS_10_7	BnaA02g25750.1D2	BnaC02g31890.1D2

RS_10_7	BnaA02g25780.1D2	BnaC02g31900.1D2
RS_10_7	BnaA02g25880.1D2	BnaC02g32750.1D2
RS_10_7	BnaA02g25910.1D2	BnaC02g33340.1D2
RS_10_7	BnaA02g26060.1D2	BnaC02g33020.1D2
RS_10_7	BnaA02g26140.1D2	BnaC02g33120.1D2
RS_10_7	BnaA02g26360.1D2	BnaC02g33300.1D2
RS_10_7	BnaA02g26370.1D2	BnaC02g33310.1D2
RS_10_7	BnaA02g26420.1D2	BnaC02g33450.1D2
RS_10_7	BnaA02g26450.1D2	BnaC02g33490.1D2
RS_10_7	BnaA02g26470.1D2	BnaC02g33530.1D2
RS_10_7	BnaA02g26730.1D2	BnaC02g32840.1D2
RS_10_7	BnaA02g26740.1D2	BnaC02g32830.1D2
RS_10_7	BnaA02g26770.1D2	BnaC02g32600.1D2
RS_10_7	BnaA02g26780.1D2	BnaC02g32590.1D2
RS_10_7	BnaA02g26800.1D2	BnaC02g32560.1D2
RS_10_7	BnaA02g26820.1D2	BnaC02g32520.1D2
RS_10_7	BnaA02g26830.1D2	BnaC02g32510.1D2
RS_10_7	BnaA02g26850.1D2	BnaC02g32480.1D2
RS_10_7	BnaA02g26860.1D2	BnaC02g32470.1D2
RS_10_7	BnaA02g26880.1D2	BnaC02g32450.1D2
RS_10_7	BnaA02g26920.1D2	BnaC02g32380.1D2
RS_10_7	BnaA02g26940.1D2	BnaC02g32360.1D2
RS_10_7	BnaA02g26960.1D2	BnaC02g32330.1D2
RS_10_7	BnaA02g26980.1D2	BnaC02g32300.1D2
RS_10_7	BnaA02g27030.1D2	BnaC02g32240.1D2
RS_10_7	BnaA02g27090.1D2	BnaC02g33910.1D2
RS_10_7	BnaA02g27170.1D2	BnaC02g32390.1D2
RS_10_7	BnaA02g27210.1D2	BnaC02g33990.1D2
RS_10_7	BnaA02g27240.1D2	BnaC02g34050.1D2
RS_10_7	BnaA02g27270.1D2	BnaC02g34070.1D2
RS_10_7	BnaA02g27280.1D2	BnaC02g34080.1D2
RS_10_7	BnaA02g27290.1D2	BnaC02g34130.1D2
RS_10_7	BnaA02g27300.1D2	BnaC02g34140.1D2
RS_10_7	BnaA02g27370.1D2	BnaC02g33810.1D2
RS_10_7	BnaA02g27410.1D2	BnaC02g34260.1D2
RS_10_7	BnaA02g27420.1D2	BnaC02g34270.1D2
RS_10_7	BnaA02g27440.1D2	BnaC02g34290.1D2
RS_10_7	BnaA02g27450.1D2	BnaC02g34300.1D2
RS_10_7	BnaA02g27500.1D2	BnaC02g34350.1D2
RS_10_7	BnaA02g27550.1D2	BnaC02g35010.1D2
RS_10_7	BnaA02g27680.1D2	BnaC02g33740.1D2
RS_10_7	BnaA02g27730.1D2	BnaC02g33500.1D2
RS_10_7	BnaA02g27780.1D2	BnaC02g34400.1D2
RS_10_7	BnaA02g27850.1D2	BnaC02g34570.1D2
RS_10_7	BnaA02g27870.1D2	BnaC02g34590.1D2
RS_10_7	BnaA02g27880.1D2	BnaC02g34600.1D2
RS_10_7	BnaA02g27960.1D2	BnaC02g35200.1D2
RS_10_7	BnaA02g28040.1D2	BnaC02g34680.1D2
RS_10_7	BnaA02g28140.1D2	BnaC02g34760.1D2
RS_10_7	BnaA02g28150.1D2	BnaC02g34770.1D2
RS_10_7	BnaA02g28180.1D2	BnaC02g34810.1D2

RS_10_7	BnaA02g28200.1D2	BnaC02g34820.1D2
RS_10_7	BnaA02g28210.1D2	BnaC02g34840.1D2
RS_10_7	BnaA02g28220.1D2	BnaC02g34850.1D2
RS_10_7	BnaA02g28230.1D2	BnaC02g34860.1D2
RS_10_7	BnaA02g28280.1D2	BnaC02g34900.1D2
RS_10_7	BnaA02g28310.1D2	BnaC02g34950.1D2
RS_10_7	BnaA02g28400.1D2	BnaC02g36120.1D2
RS_10_7	BnaA02g28460.1D2	BnaC02g36170.1D2
RS_10_7	BnaA02g28470.1D2	BnaC02g36180.1D2
RS_10_7	BnaA02g28480.1D2	BnaC02g36190.1D2
RS_10_7	BnaA02g28550.1D2	BnaC02g35090.1D2
RS_10_7	BnaA02g28590.1D2	BnaC02g35220.1D2
RS_10_7	BnaA02g28600.1D2	BnaC02g35230.1D2
RS_10_7	BnaA02g28610.1D2	BnaC02g35240.1D2
RS_10_7	BnaA02g28890.1D2	BnaC02g35430.1D2
RS_10_7	BnaA02g28910.1D2	BnaC02g35460.1D2
RS_10_7	BnaA02g28950.1D2	BnaC02g35490.1D2
RS_10_7	BnaA02g29110.1D2	BnaC02g35650.1D2
RS_10_7	BnaA02g29200.1D2	BnaC02g35780.1D2
RS_10_7	BnaA02g29210.1D2	BnaC02g35790.1D2
RS_10_7	BnaA02g29220.1D2	BnaC02g35810.1D2
RS_10_7	BnaA02g29240.1D2	BnaC02g35850.1D2
RS_10_7	BnaA02g29310.1D2	BnaC02g35920.1D2
RS_10_7	BnaA02g29320.1D2	BnaC02g35930.1D2
RS_10_7	BnaA02g29350.1D2	BnaC02g35960.1D2
RS_10_7	BnaA02g29430.1D2	BnaC02g17330.1D2
RS_10_7	BnaA02g29520.1D2	BnaC02g36300.1D2
RS_10_7	BnaA02g29540.1D2	BnaC02g36340.1D2
RS_10_7	BnaA02g29560.1D2	BnaC02g36350.1D2
RS_10_7	BnaA02g29570.1D2	BnaC02g36360.1D2
RS_10_7	BnaA02g29580.1D2	BnaC02g36380.1D2
RS_10_7	BnaA02g29590.1D2	BnaC02g36390.1D2
RS_10_7	BnaA02g29640.1D2	BnaC02g36440.1D2
RS_10_7	BnaA02g29670.1D2	BnaC02g36470.1D2
RS_10_7	BnaA02g29730.1D2	BnaC02g36570.1D2
RS_10_7	BnaA02g29740.1D2	BnaC02g24530.1D2
RS_10_7	BnaA02g29740.1D2	BnaC02g36580.1D2
RS_10_7	BnaA02g29750.1D2	BnaC02g36600.1D2
RS_10_7	BnaA02g29760.1D2	BnaC02g36610.1D2
RS_10_7	BnaA02g29770.1D2	BnaC02g36620.1D2
RS_10_7	BnaA02g29840.1D2	BnaC02g36710.1D2
RS_10_7	BnaA02g29920.1D2	BnaC02g36770.1D2
RS_10_7	BnaA02g29940.1D2	BnaC02g36810.1D2
RS_10_7	BnaA02g29960.1D2	BnaC02g37700.1D2
RS_10_7	BnaA02g30010.1D2	BnaC02g36830.1D2
RS_10_7	BnaA02g30020.1D2	BnaC02g36830.1D2
RS_10_7	BnaA02g30050.1D2	BnaC02g36840.1D2
RS_10_7	BnaA02g30080.1D2	BnaC02g36870.1D2
RS_10_7	BnaA02g30110.1D2	BnaC02g36950.1D2
RS_10_7	BnaA02g30150.1D2	BnaC02g36990.1D2
RS_10_7	BnaA02g30220.1D2	BnaC02g36030.1D2

RS_10_7	BnaA02g30240.1D2	BnaC02g37020.1D2
RS_10_7	BnaA02g30280.1D2	BnaC02g37050.1D2
RS_10_7	BnaA02g30290.1D2	BnaC02g37060.1D2
RS_10_7	BnaA02g30370.1D2	BnaC02g37090.1D2
RS_10_7	BnaA02g30450.1D2	BnaC02g37220.1D2
RS_10_7	BnaA02g30610.1D2	BnaC02g37820.1D2
RS_10_7	BnaA02g30630.1D2	BnaC02g37840.1D2
RS_10_7	BnaA02g30680.1D2	BnaC02g37880.1D2
RS_10_7	BnaA02g30700.1D2	BnaC02g37900.1D2
RS_10_7	BnaA02g30710.1D2	BnaC02g37910.1D2
RS_10_7	BnaA02g30720.1D2	BnaC02g37920.1D2
RS_10_7	BnaA02g30750.1D2	BnaC02g37950.1D2
RS_10_7	BnaA02g30770.1D2	BnaC02g37970.1D2
RS_10_7	BnaA02g30790.1D2	BnaC02g38000.1D2
RS_10_7	BnaA02g30840.1D2	BnaC02g38040.1D2
RS_10_7	BnaA02g30940.1D2	BnaC02g38100.1D2
RS_10_7	BnaA02g30950.1D2	BnaC02g38140.1D2
RS_10_7	BnaA02g31220.1D2	BnaC02g38350.1D2
RS_10_7	BnaA02g31320.1D2	BnaC02g38990.1D2
RS_10_7	BnaA02g31360.1D2	BnaC02g39030.1D2
RS_10_7	BnaA02g31460.1D2	BnaC02g38490.1D2
RS_10_7	BnaA02g31480.1D2	BnaC02g38520.1D2
RS_10_7	BnaA02g31510.1D2	BnaC02g38550.1D2
RS_10_7	BnaA02g31620.1D2	BnaC02g38650.1D2
RS_10_7	BnaA02g31680.1D2	BnaC02g38760.1D2
RS_10_7	BnaA02g32100.1D2	BnaC02g38880.1D2
RS_10_7	BnaA02g32160.1D2	BnaC02g37710.1D2
RS_10_7	BnaA02g32180.1D2	BnaC02g37460.1D2
RS_10_7	BnaA02g32200.1D2	BnaC02g37420.1D2
RS_10_7	BnaA02g32240.1D2	BnaC02g37370.1D2
RS_10_7	BnaA02g32250.1D2	BnaC02g37360.1D2
RS_10_7	BnaA02g32300.1D2	BnaC02g39280.1D2
RS_10_7	BnaA02g32360.1D2	BnaC02g39350.1D2
RS_10_7	BnaA02g32370.1D2	BnaC02g39360.1D2
RS_10_7	BnaA02g32400.1D2	BnaC02g38940.1D2
RS_10_7	BnaA02g32420.1D2	BnaC02g39440.1D2
RS_10_7	BnaA02g32450.1D2	BnaC02g39480.1D2
RS_10_7	BnaA02g32480.1D2	BnaC02g39510.1D2
RS_10_7	BnaA02g32500.1D2	BnaC02g39530.1D2
RS_10_7	BnaA02g32660.1D2	BnaC02g39600.1D2
RS_10_7	BnaA02g32670.1D2	BnaC02g39590.1D2
RS_10_7	BnaA02g32740.1D2	BnaC02g39970.1D2
RS_10_7	BnaA02g32760.1D2	BnaC02g39750.1D2
RS_10_7	BnaA02g32840.1D2	BnaC02g39830.1D2
RS_10_7	BnaA02g32900.1D2	BnaC02g39920.1D2
RS_10_7	BnaA02g32920.1D2	BnaC02g39930.1D2
RS_10_7	BnaA02g32920.1D2	BnaC02g41070.1D2
RS_10_7	BnaA02g32950.1D2	BnaC02g39400.1D2
RS_10_7	BnaA02g33410.1D2	BnaC02g40170.1D2
RS_10_7	BnaA02g33440.1D2	BnaC02g40200.1D2
RS_10_7	BnaA02g33460.1D2	BnaC02g40230.1D2

RS_10_7	BnaA02g33540.1D2	BnaC02g40340.1D2
RS_10_7	BnaA02g33760.1D2	BnaC02g40520.1D2
RS_10_7	BnaA02g33780.1D2	BnaC02g40550.1D2
RS_10_7	BnaA02g33830.1D2	BnaC02g40630.1D2
RS_10_7	BnaA02g33860.1D2	BnaC02g40680.1D2
RS_10_7	BnaA02g33970.1D2	BnaC02g40820.1D2
RS_10_7	BnaA02g34040.1D2	BnaC02g40870.1D2
RS_10_7	BnaA02g34050.1D2	BnaC02g40870.1D2
RS_10_7	BnaA02g34130.1D2	BnaC02g40920.1D2
RS_10_7	BnaA02g34140.1D2	BnaC02g40930.1D2
RS_10_7	BnaA02g34150.1D2	BnaC02g40940.1D2
RS_10_7	BnaA02g34190.1D2	BnaC02g40990.1D2
RS_10_7	BnaA02g34220.1D2	BnaC02g41050.1D2
RS_10_7	BnaA02g34350.1D2	BnaC02g41510.1D2
RS_10_7	BnaA02g34350.1D2	BnaC02g41640.1D2
RS_10_7	BnaA02g34420.1D2	BnaC02g41240.1D2
RS_10_7	BnaA02g34520.1D2	BnaC02g41410.1D2
RS_10_7	BnaA02g34570.1D2	BnaC02g41770.1D2
RS_10_7	BnaA02g34600.1D2	BnaC02g41750.1D2
RS_10_7	BnaA02g34640.1D2	BnaC02g41650.1D2
RS_10_7	BnaA02g34760.1D2	BnaC02g17080.1D2
RS_10_7	BnaA02g34780.1D2	BnaC02g17120.1D2
RS_10_7	BnaA02g34790.1D2	BnaC02g17130.1D2
RS_10_7	BnaA02g34810.1D2	BnaC02g17150.1D2
RS_10_7	BnaA02g34850.1D2	BnaC02g41360.1D2
RS_10_7	BnaA02g34910.1D2	BnaC02g41470.1D2
RS_10_7	BnaA02g34980.1D2	BnaC02g41540.1D2
RS_10_7	BnaA02g35210.1D2	BnaC02g17450.1D2
RS_10_7	BnaA02g35220.1D2	BnaC02g17430.1D2
RS_10_7	BnaA02g35260.1D2	BnaC02g17380.1D2
S_39	BnaA02g00010.1D2	BnaC02g00740.1D2
S_39	BnaA02g00020.1D2	BnaC02g00730.1D2
S_39	BnaA02g00030.1D2	BnaC02g00690.1D2
S_39	BnaA02g00150.1D2	BnaC02g00630.1D2
S_39	BnaA02g00190.1D2	BnaC02g00590.1D2
S_39	BnaA02g00190.1D2	BnaC02g11160.1D2
S_39	BnaA02g00210.1D2	BnaC02g00570.1D2
S_39	BnaA02g00220.1D2	BnaC02g00560.1D2
S_39	BnaA02g00290.1D2	BnaC02g00500.1D2
S_39	BnaA02g00300.1D2	BnaC02g00490.1D2
S_39	BnaA02g00310.1D2	BnaC02g00480.1D2
S_39	BnaA02g00320.1D2	BnaC02g00470.1D2
S_39	BnaA02g00350.1D2	BnaC02g00450.1D2
S_39	BnaA02g00380.1D2	BnaC02g00410.1D2
S_39	BnaA02g00420.1D2	BnaC02g00370.1D2
S_39	BnaA02g00440.1D2	BnaC02g00350.1D2
S_39	BnaA02g00450.1D2	BnaC02g00340.1D2
S_39	BnaA02g00490.1D2	BnaC02g00300.1D2
S_39	BnaA02g00510.1D2	BnaC02g00280.1D2
S_39	BnaA02g00520.1D2	BnaC02g00270.1D2
S_39	BnaA02g00550.1D2	BnaC02g00240.1D2

S_39	BnaA02g00560.1D2	BnaC02g00230.1D2
S_39	BnaA02g00580.1D2	BnaC02g00210.1D2
S_39	BnaA02g00630.1D2	BnaC02g00110.1D2
S_39	BnaA02g00670.1D2	BnaC02g00070.1D2
S_39	BnaA02g00700.1D2	BnaC02g00040.1D2
S_39	BnaA02g00710.1D2	BnaC02g00030.1D2
S_39	BnaA02g00940.1D2	BnaC02g05980.1D2
S_39	BnaA02g01050.1D2	BnaC02g06370.1D2
S_39	BnaA02g01060.1D2	BnaC02g06380.1D2
S_39	BnaA02g01070.1D2	BnaC02g06390.1D2
S_39	BnaA02g01080.1D2	BnaC02g06400.1D2
S_39	BnaA02g01100.1D2	BnaC02g06420.1D2
S_39	BnaA02g01120.1D2	BnaC02g06440.1D2
S_39	BnaA02g01150.1D2	BnaC02g06450.1D2
S_39	BnaA02g01190.1D2	BnaC02g06470.1D2
S_39	BnaA02g01230.1D2	BnaC02g06500.1D2
S_39	BnaA02g01240.1D2	BnaC02g06520.1D2
S_39	BnaA02g01320.1D2	BnaC02g06610.1D2
S_39	BnaA02g01340.1D2	BnaC02g06630.1D2
S_39	BnaA02g01360.1D2	BnaC02g06650.1D2
S_39	BnaA02g01370.1D2	BnaC02g06660.1D2
S_39	BnaA02g01380.1D2	BnaC02g06670.1D2
S_39	BnaA02g01390.1D2	BnaC02g06680.1D2
S_39	BnaA02g01550.1D2	BnaC02g06850.1D2
S_39	BnaA02g01550.1D2	BnaC02g06860.1D2
S_39	BnaA02g01560.1D2	BnaC02g06850.1D2
S_39	BnaA02g01560.1D2	BnaC02g06860.1D2
S_39	BnaA02g01570.1D2	BnaC02g06870.1D2
S_39	BnaA02g01590.1D2	BnaC02g06890.1D2
S_39	BnaA02g01600.1D2	BnaC02g06900.1D2
S_39	BnaA02g01610.1D2	BnaC02g06910.1D2
S_39	BnaA02g01620.1D2	BnaC02g06920.1D2
S_39	BnaA02g01630.1D2	BnaC02g06930.1D2
S_39	BnaA02g01650.1D2	BnaC02g06950.1D2
S_39	BnaA02g01660.1D2	BnaC02g06960.1D2
S_39	BnaA02g01690.1D2	BnaC02g06990.1D2
S_39	BnaA02g01700.1D2	BnaC02g07000.1D2
S_39	BnaA02g01720.1D2	BnaC02g07030.1D2
S_39	BnaA02g01770.1D2	BnaC02g07090.1D2
S_39	BnaA02g01820.1D2	BnaC02g07140.1D2
S_39	BnaA02g01840.1D2	BnaC02g07150.1D2
S_39	BnaA02g01870.1D2	BnaC02g07180.1D2
S_39	BnaA02g01880.1D2	BnaC02g07190.1D2
S_39	BnaA02g01890.1D2	BnaC02g07200.1D2
S_39	BnaA02g01900.1D2	BnaC02g07210.1D2
S_39	BnaA02g01950.1D2	BnaC02g07270.1D2
S_39	BnaA02g01960.1D2	BnaC02g07280.1D2
S_39	BnaA02g01970.1D2	BnaC02g07290.1D2
S_39	BnaA02g01980.1D2	BnaC02g07300.1D2
S_39	BnaA02g01990.1D2	BnaC02g07310.1D2
S_39	BnaA02g02000.1D2	BnaC02g07320.1D2

S_39	BnaA02g02080.1D2	BnaC02g07420.1D2
S_39	BnaA02g02090.1D2	BnaC02g07440.1D2
S_39	BnaA02g02100.1D2	BnaC02g07520.1D2
S_39	BnaA02g02110.1D2	BnaC02g07530.1D2
S_39	BnaA02g02120.1D2	BnaC02g07540.1D2
S_39	BnaA02g02140.1D2	BnaC02g07590.1D2
S_39	BnaA02g02150.1D2	BnaC02g07600.1D2
S_39	BnaA02g02190.1D2	BnaC02g07660.1D2
S_39	BnaA02g02250.1D2	BnaC02g07720.1D2
S_39	BnaA02g02270.1D2	BnaC02g07740.1D2
S_39	BnaA02g02280.1D2	BnaC02g07750.1D2
S_39	BnaA02g02310.1D2	BnaC02g07780.1D2
S_39	BnaA02g02320.1D2	BnaC02g07790.1D2
S_39	BnaA02g02340.1D2	BnaC02g07800.1D2
S_39	BnaA02g02350.1D2	BnaC02g07810.1D2
S_39	BnaA02g02390.1D2	BnaC02g07850.1D2
S_39	BnaA02g02400.1D2	BnaC02g07860.1D2
S_39	BnaA02g02410.1D2	BnaC02g07870.1D2
S_39	BnaA02g02500.1D2	BnaC02g07920.1D2
S_39	BnaA02g02520.1D2	BnaC02g07940.1D2
S_39	BnaA02g02680.1D2	BnaC02g08150.1D2
S_39	BnaA02g02730.1D2	BnaC02g08200.1D2
S_39	BnaA02g02740.1D2	BnaC02g08210.1D2
S_39	BnaA02g02820.1D2	BnaC02g08290.1D2
S_39	BnaA02g02870.1D2	BnaC02g08340.1D2
S_39	BnaA02g02880.1D2	BnaC02g08350.1D2
S_39	BnaA02g02910.1D2	BnaC02g08380.1D2
S_39	BnaA02g02990.1D2	BnaC02g08580.1D2
S_39	BnaA02g03020.1D2	BnaC02g08600.1D2
S_39	BnaA02g03050.1D2	BnaC02g08630.1D2
S_39	BnaA02g03080.1D2	BnaC02g08660.1D2
S_39	BnaA02g03090.1D2	BnaC02g08680.1D2
S_39	BnaA02g03100.1D2	BnaC02g08690.1D2
S_39	BnaA02g03110.1D2	BnaC02g08700.1D2
S_39	BnaA02g03150.1D2	BnaC02g08720.1D2
S_39	BnaA02g03150.1D2	BnaC02g08740.1D2
S_39	BnaA02g03160.1D2	BnaC02g08720.1D2
S_39	BnaA02g03160.1D2	BnaC02g08740.1D2
S_39	BnaA02g03170.1D2	BnaC02g08750.1D2
S_39	BnaA02g03210.1D2	BnaC02g08800.1D2
S_39	BnaA02g03260.1D2	BnaC02g08850.1D2
S_39	BnaA02g03270.1D2	BnaC02g08860.1D2
S_39	BnaA02g03280.1D2	BnaC02g08870.1D2
S_39	BnaA02g03300.1D2	BnaC02g08890.1D2
S_39	BnaA02g03480.1D2	BnaC02g09150.1D2
S_39	BnaA02g03490.1D2	BnaC02g09160.1D2
S_39	BnaA02g03500.1D2	BnaC02g09170.1D2
S_39	BnaA02g03690.1D2	BnaC02g09420.1D2
S_39	BnaA02g03700.1D2	BnaC02g09430.1D2
S_39	BnaA02g03810.1D2	BnaC02g09830.1D2
S_39	BnaA02g03920.1D2	BnaC02g08530.1D2

S_39	BnaA02g03950.1D2	BnaC02g06190.1D2
S_39	BnaA02g04000.1D2	BnaC02g06150.1D2
S_39	BnaA02g04030.1D2	BnaC02g06120.1D2
S_39	BnaA02g04040.1D2	BnaC02g06110.1D2
S_39	BnaA02g04110.1D2	BnaC02g00150.1D2
S_39	BnaA02g04120.1D2	BnaC02g00140.1D2
S_39	BnaA02g04310.1D2	BnaC02g06280.1D2
S_39	BnaA02g04470.1D2	BnaC02g09270.1D2
S_39	BnaA02g04780.1D2	BnaC02g09490.1D2
S_39	BnaA02g04790.1D2	BnaC02g09470.1D2
S_39	BnaA02g04790.1D2	BnaC02g09510.1D2
S_39	BnaA02g04820.1D2	BnaC02g09470.1D2
S_39	BnaA02g04820.1D2	BnaC02g09510.1D2
S_39	BnaA02g04820.1D2	BnaC02g09520.1D2
S_39	BnaA02g04820.1D2	BnaC02g09550.1D2
S_39	BnaA02g04870.1D2	BnaC02g09610.1D2
S_39	BnaA02g05160.1D2	BnaC02g10130.1D2
S_39	BnaA02g05170.1D2	BnaC02g10140.1D2
S_39	BnaA02g05210.1D2	BnaC02g10170.1D2
S_39	BnaA02g05220.1D2	BnaC02g10180.1D2
S_39	BnaA02g05230.1D2	BnaC02g10190.1D2
S_39	BnaA02g05240.1D2	BnaC02g10200.1D2
S_39	BnaA02g05260.1D2	BnaC02g10190.1D2
S_39	BnaA02g05370.1D2	BnaC02g10270.1D2
S_39	BnaA02g05380.1D2	BnaC02g10280.1D2
S_39	BnaA02g05420.1D2	BnaC02g10360.1D2
S_39	BnaA02g05450.1D2	BnaC02g10400.1D2
S_39	BnaA02g05470.1D2	BnaC02g10410.1D2
S_39	BnaA02g05520.1D2	BnaC02g10470.1D2
S_39	BnaA02g05570.1D2	BnaC02g10500.1D2
S_39	BnaA02g05640.1D2	BnaC02g10600.1D2
S_39	BnaA02g05730.1D2	BnaC02g10700.1D2
S_39	BnaA02g05750.1D2	BnaC02g10720.1D2
S_39	BnaA02g06690.1D2	BnaC02g10790.1D2
S_39	BnaA02g06700.1D2	BnaC02g10800.1D2
S_39	BnaA02g06710.1D2	BnaC02g10810.1D2
S_39	BnaA02g06740.1D2	BnaC02g10860.1D2
S_39	BnaA02g06800.1D2	BnaC02g10900.1D2
S_39	BnaA02g06820.1D2	BnaC02g10920.1D2
S_39	BnaA02g06840.1D2	BnaC02g10940.1D2
S_39	BnaA02g06890.1D2	BnaC02g15180.1D2
S_39	BnaA02g06910.1D2	BnaC02g15160.1D2
S_39	BnaA02g07850.1D2	BnaC02g15480.1D2
S_39	BnaA02g08000.1D2	BnaC02g11110.1D2
S_39	BnaA02g08010.1D2	BnaC02g11110.1D2
S_39	BnaA02g08040.1D2	BnaC02g11140.1D2
S_39	BnaA02g08050.1D2	BnaC02g11150.1D2
S_39	BnaA02g08060.1D2	BnaC02g00590.1D2
S_39	BnaA02g08060.1D2	BnaC02g11160.1D2
S_39	BnaA02g08100.1D2	BnaC02g11210.1D2
S_39	BnaA02g08140.1D2	BnaC02g11290.1D2

S_39	BnaA02g08160.1D2	BnaC02g11310.1D2
S_39	BnaA02g08230.1D2	BnaC02g11420.1D2
S_39	BnaA02g08330.1D2	BnaC02g11520.1D2
S_39	BnaA02g08360.1D2	BnaC02g11550.1D2
S_39	BnaA02g08460.1D2	BnaC02g11670.1D2
S_39	BnaA02g08470.1D2	BnaC02g12400.1D2
S_39	BnaA02g08530.1D2	BnaC02g11700.1D2
S_39	BnaA02g08560.1D2	BnaC02g11750.1D2
S_39	BnaA02g08630.1D2	BnaC02g11810.1D2
S_39	BnaA02g08650.1D2	BnaC02g11820.1D2
S_39	BnaA02g08670.1D2	BnaC02g11840.1D2
S_39	BnaA02g08710.1D2	BnaC02g11870.1D2
S_39	BnaA02g16940.1D2	BnaC02g06790.1D2
S_39	BnaA02g33390.1D2	BnaC02g40120.1D2
S_39	BnaA02g33410.1D2	BnaC02g40170.1D2
S_39	BnaA02g33440.1D2	BnaC02g40200.1D2
S_39	BnaA02g33450.1D2	BnaC02g40210.1D2
S_39	BnaA02g33460.1D2	BnaC02g40230.1D2
S_39	BnaA02g33750.1D2	BnaC02g40900.1D2
S_39	BnaA02g33760.1D2	BnaC02g40520.1D2
S_39	BnaA02g33780.1D2	BnaC02g40550.1D2
S_39	BnaA02g33830.1D2	BnaC02g40630.1D2
S_39	BnaA02g33840.1D2	BnaC02g40640.1D2
S_39	BnaA02g33850.1D2	BnaC02g40650.1D2
S_39	BnaA02g33860.1D2	BnaC02g40680.1D2
S_39	BnaA02g33870.1D2	BnaC02g40690.1D2
S_39	BnaA02g33950.1D2	BnaC02g40800.1D2
S_39	BnaA02g33970.1D2	BnaC02g40820.1D2
S_39	BnaA02g33990.1D2	BnaC02g40840.1D2
S_39	BnaA02g34000.1D2	BnaC02g40850.1D2
S_39	BnaA02g34040.1D2	BnaC02g40870.1D2
S_39	BnaA02g34050.1D2	BnaC02g40870.1D2
S_39	BnaA02g34130.1D2	BnaC02g40920.1D2
S_39	BnaA02g34140.1D2	BnaC02g40930.1D2
S_39	BnaA02g34150.1D2	BnaC02g40940.1D2
S_39	BnaA02g34190.1D2	BnaC02g40990.1D2
S_39	BnaA02g34220.1D2	BnaC02g41050.1D2
S_39	BnaA02g34240.1D2	BnaC02g41060.1D2
S_39	BnaA02g34250.1D2	BnaC02g41080.1D2
S_39	BnaA02g34300.1D2	BnaC02g41130.1D2
S_39	BnaA02g34330.1D2	BnaC02g41150.1D2
S_39	BnaA02g34380.1D2	BnaC02g41180.1D2
S_39	BnaA02g34400.1D2	BnaC02g41220.1D2
S_39	BnaA02g34420.1D2	BnaC02g41240.1D2
S_39	BnaA02g34570.1D2	BnaC02g41770.1D2
S_39	BnaA02g34600.1D2	BnaC02g41750.1D2
S_39	BnaA02g34790.1D2	BnaC02g17130.1D2
S_39	BnaA02g34810.1D2	BnaC02g17150.1D2
S_39	BnaA02g34900.1D2	BnaC02g41460.1D2
S_39	BnaA02g34910.1D2	BnaC02g41470.1D2
S_39	BnaA02g34980.1D2	BnaC02g41540.1D2

S_39	BnaA02g35210.1D2	BnaC02g17450.1D2
S_39	BnaA02g35220.1D2	BnaC02g17430.1D2
H165	BnaA02g04850.1D2	BnaC02g09590.1D2
H165	BnaA02g27240.1D2	BnaC02g34050.1D2
CRY_1	BnaA02g04830.1D2	BnaC02g33250.1D2
CRY_1	BnaA02g17090.1D2	BnaC02g23100.1D2
CRY_1	BnaA02g17170.1D2	BnaC02g22000.1D2
CRY_1	BnaA02g17210.1D2	BnaC02g22050.1D2
CRY_1	BnaA02g17260.1D2	BnaC02g22130.1D2
CRY_1	BnaA02g17300.1D2	BnaC02g22510.1D2
CRY_1	BnaA02g17370.1D2	BnaC02g22720.1D2
CRY_1	BnaA02g17480.1D2	BnaC02g22300.1D2
CRY_1	BnaA02g17520.1D2	BnaC02g22370.1D2
CRY_1	BnaA02g17540.1D2	BnaC02g22400.1D2
CRY_1	BnaA02g17830.1D2	BnaC02g23390.1D2
CRY_1	BnaA02g17990.1D2	BnaC02g22870.1D2
CRY_1	BnaA02g18140.1D2	BnaC02g25080.1D2
CRY_1	BnaA02g18240.1D2	BnaC02g24570.1D2
CRY_1	BnaA02g18310.1D2	BnaC02g24460.1D2
CRY_1	BnaA02g18390.1D2	BnaC02g24380.1D2
CRY_1	BnaA02g18480.1D2	BnaC02g24910.1D2
CRY_1	BnaA02g18490.1D2	BnaC02g24930.1D2
CRY_1	BnaA02g18500.1D2	BnaC02g24940.1D2
CRY_1	BnaA02g18520.1D2	BnaC02g24960.1D2
CRY_1	BnaA02g18590.1D2	BnaC02g24660.1D2
CRY_1	BnaA02g18870.1D2	BnaC02g24890.1D2
CRY_1	BnaA02g19080.1D2	BnaC02g26380.1D2
CRY_1	BnaA02g19310.1D2	BnaC02g22770.1D2
CRY_1	BnaA02g19390.1D2	BnaC02g23620.1D2
CRY_1	BnaA02g19470.1D2	BnaC02g25240.1D2
CRY_1	BnaA02g19620.1D2	BnaC02g25430.1D2
CRY_1	BnaA02g19660.1D2	BnaC02g25490.1D2
CRY_1	BnaA02g19810.1D2	BnaC02g25680.1D2
CRY_1	BnaA02g19820.1D2	BnaC02g25690.1D2
CRY_1	BnaA02g19900.1D2	BnaC02g25820.1D2
CRY_1	BnaA02g20060.1D2	BnaC02g24210.1D2
CRY_1	BnaA02g20270.1D2	BnaC02g22230.1D2
CRY_1	BnaA02g20340.1D2	BnaC02g24000.1D2
CRY_1	BnaA02g20990.1D2	BnaC02g23940.1D2
CRY_1	BnaA02g21430.1D2	BnaC02g27640.1D2
CRY_1	BnaA02g21570.1D2	BnaC02g29600.1D2
CRY_1	BnaA02g22040.1D2	BnaC02g24140.1D2
CRY_1	BnaA02g22170.1D2	BnaC02g28790.1D2
CRY_1	BnaA02g22230.1D2	BnaC02g30250.1D2
CRY_1	BnaA02g22240.1D2	BnaC02g27720.1D2
CRY_1	BnaA02g22570.1D2	BnaC02g31350.1D2
CRY_1	BnaA02g22600.1D2	BnaC02g31300.1D2
CRY_1	BnaA02g22610.1D2	BnaC02g31290.1D2
CRY_1	BnaA02g22680.1D2	BnaC02g31210.1D2
CRY_1	BnaA02g22700.1D2	BnaC02g29740.1D2
CRY_1	BnaA02g22790.1D2	BnaC02g28120.1D2

CRY_1	BnaA02g22800.1D2	BnaC02g28130.1D2
CRY_1	BnaA02g22810.1D2	BnaC02g28150.1D2
CRY_1	BnaA02g22860.1D2	BnaC02g28240.1D2
CRY_1	BnaA02g23020.1D2	BnaC02g29620.1D2
CRY_1	BnaA02g23030.1D2	BnaC02g30140.1D2
CRY_1	BnaA02g23180.1D2	BnaC02g29000.1D2
CRY_1	BnaA02g23290.1D2	BnaC02g29140.1D2
CRY_1	BnaA02g23380.1D2	BnaC02g29330.1D2
CRY_1	BnaA02g23430.1D2	BnaC02g29390.1D2
CRY_1	BnaA02g23810.1D2	BnaC02g28530.1D2
CRY_1	BnaA02g24400.1D2	BnaC02g30340.1D2
CRY_1	BnaA02g24500.1D2	BnaC02g30420.1D2
CRY_1	BnaA02g24520.1D2	BnaC02g30470.1D2
CRY_1	BnaA02g24570.1D2	BnaC02g30560.1D2
CRY_1	BnaA02g24660.1D2	BnaC02g30580.1D2
CRY_1	BnaA02g24690.1D2	BnaC02g30610.1D2
CRY_1	BnaA02g24740.1D2	BnaC02g30660.1D2
CRY_1	BnaA02g24780.1D2	BnaC02g30670.1D2
CRY_1	BnaA02g24910.1D2	BnaC02g31710.1D2
CRY_1	BnaA02g25020.1D2	BnaC02g31380.1D2
CRY_1	BnaA02g25040.1D2	BnaC02g30790.1D2
CRY_1	BnaA02g25070.1D2	BnaC02g30770.1D2
CRY_1	BnaA02g25210.1D2	BnaC02g30950.1D2
CRY_1	BnaA02g25240.1D2	BnaC02g32010.1D2
CRY_1	BnaA02g25270.1D2	BnaC02g32030.1D2
CRY_1	BnaA02g25460.1D2	BnaC02g31570.1D2
CRY_1	BnaA02g25550.1D2	BnaC02g32040.1D2
CRY_1	BnaA02g25610.1D2	BnaC02g32100.1D2
CRY_1	BnaA02g25630.1D2	BnaC02g32120.1D2
CRY_1	BnaA02g25640.1D2	BnaC02g32130.1D2
CRY_1	BnaA02g25650.1D2	BnaC02g32140.1D2
CRY_1	BnaA02g25780.1D2	BnaC02g31900.1D2
CRY_1	BnaA02g25800.1D2	BnaC02g22690.1D2
CRY_1	BnaA02g25910.1D2	BnaC02g33340.1D2
CRY_1	BnaA02g26060.1D2	BnaC02g33020.1D2
CRY_1	BnaA02g26470.1D2	BnaC02g33530.1D2
CRY_1	BnaA02g26730.1D2	BnaC02g32840.1D2
CRY_1	BnaA02g26780.1D2	BnaC02g32590.1D2
CRY_1	BnaA02g26830.1D2	BnaC02g32510.1D2
CRY_1	BnaA02g26850.1D2	BnaC02g32480.1D2
CRY_1	BnaA02g27030.1D2	BnaC02g32240.1D2
CRY_1	BnaA02g27050.1D2	BnaC02g33860.1D2
CRY_1	BnaA02g27090.1D2	BnaC02g33910.1D2
CRY_1	BnaA02g27110.1D2	BnaC02g33950.1D2
CRY_1	BnaA02g27210.1D2	BnaC02g33990.1D2
CRY_1	BnaA02g27240.1D2	BnaC02g34050.1D2
CRY_1	BnaA02g27280.1D2	BnaC02g34080.1D2
CRY_1	BnaA02g27290.1D2	BnaC02g34130.1D2
CRY_1	BnaA02g27300.1D2	BnaC02g34140.1D2
CRY_1	BnaA02g27400.1D2	BnaC02g34250.1D2
CRY_1	BnaA02g27410.1D2	BnaC02g34260.1D2

CRY_1	BnaA02g27420.1D2	BnaC02g34270.1D2
CRY_1	BnaA02g27430.1D2	BnaC02g34280.1D2
CRY_1	BnaA02g27450.1D2	BnaC02g34300.1D2
CRY_1	BnaA02g27490.1D2	BnaC02g34340.1D2
CRY_1	BnaA02g27500.1D2	BnaC02g34350.1D2
CRY_1	BnaA02g27550.1D2	BnaC02g35010.1D2
CRY_1	BnaA02g27680.1D2	BnaC02g33740.1D2
CRY_1	BnaA02g27790.1D2	BnaC02g34410.1D2
CRY_1	BnaA02g27850.1D2	BnaC02g34570.1D2
CRY_1	BnaA02g27870.1D2	BnaC02g34590.1D2
CRY_1	BnaA02g27900.1D2	BnaC02g34620.1D2
CRY_1	BnaA02g28040.1D2	BnaC02g34680.1D2
CRY_1	BnaA02g28080.1D2	BnaC02g34730.1D2
CRY_1	BnaA02g28160.1D2	BnaC02g34780.1D2
CRY_1	BnaA02g28400.1D2	BnaC02g36120.1D2
CRY_1	BnaA02g28460.1D2	BnaC02g36170.1D2
CRY_1	BnaA02g28530.1D2	BnaC02g35070.1D2
CRY_1	BnaA02g28550.1D2	BnaC02g35090.1D2
CRY_1	BnaA02g28590.1D2	BnaC02g35220.1D2
CRY_1	BnaA02g28600.1D2	BnaC02g35230.1D2
CRY_1	BnaA02g28890.1D2	BnaC02g35430.1D2
CRY_1	BnaA02g28910.1D2	BnaC02g35460.1D2
CRY_1	BnaA02g28950.1D2	BnaC02g35490.1D2
CRY_1	BnaA02g29090.1D2	BnaC02g35640.1D2
CRY_1	BnaA02g29110.1D2	BnaC02g35650.1D2
CRY_1	BnaA02g29120.1D2	BnaC02g35660.1D2
CRY_1	BnaA02g29210.1D2	BnaC02g35790.1D2
CRY_1	BnaA02g29320.1D2	BnaC02g35930.1D2
CRY_1	BnaA02g29520.1D2	BnaC02g36300.1D2
CRY_1	BnaA02g29530.1D2	BnaC02g36330.1D2
CRY_1	BnaA02g29640.1D2	BnaC02g36440.1D2
CRY_1	BnaA02g29650.1D2	BnaC02g36450.1D2
CRY_1	BnaA02g29670.1D2	BnaC02g36470.1D2
CRY_1	BnaA02g29690.1D2	BnaC02g36510.1D2
CRY_1	BnaA02g29700.1D2	BnaC02g36520.1D2
CRY_1	BnaA02g29720.1D2	BnaC02g36560.1D2
CRY_1	BnaA02g29730.1D2	BnaC02g36570.1D2
CRY_1	BnaA02g29920.1D2	BnaC02g36770.1D2
CRY_1	BnaA02g30010.1D2	BnaC02g36830.1D2
CRY_1	BnaA02g30080.1D2	BnaC02g36870.1D2
CRY_1	BnaA02g30220.1D2	BnaC02g36030.1D2
CRY_1	BnaA02g30240.1D2	BnaC02g37020.1D2
CRY_1	BnaA02g30290.1D2	BnaC02g37060.1D2
CRY_1	BnaA02g30370.1D2	BnaC02g37090.1D2
CRY_1	BnaA02g30600.1D2	BnaC02g37810.1D2
CRY_1	BnaA02g30680.1D2	BnaC02g37880.1D2
CRY_1	BnaA02g30700.1D2	BnaC02g37900.1D2
CRY_1	BnaA02g30720.1D2	BnaC02g37920.1D2
CRY_1	BnaA02g30750.1D2	BnaC02g37950.1D2
CRY_1	BnaA02g30770.1D2	BnaC02g37970.1D2
CRY_1	BnaA02g30790.1D2	BnaC02g38000.1D2

CRY_1	BnaA02g30840.1D2	BnaC02g38040.1D2
CRY_1	BnaA02g30940.1D2	BnaC02g38100.1D2
CRY_1	BnaA02g31140.1D2	BnaC02g38300.1D2
CRY_1	BnaA02g31510.1D2	BnaC02g38550.1D2
CRY_1	BnaA02g32100.1D2	BnaC02g38880.1D2
CRY_1	BnaA02g32240.1D2	BnaC02g37370.1D2
CRY_1	BnaA02g32250.1D2	BnaC02g37360.1D2
CRY_1	BnaA02g32480.1D2	BnaC02g39510.1D2
CRY_1	BnaA02g32670.1D2	BnaC02g39590.1D2
CRY_1	BnaA02g32710.1D2	BnaC02g39700.1D2
CRY_1	BnaA02g33760.1D2	BnaC02g40520.1D2
CRY_1	BnaA02g33780.1D2	BnaC02g40550.1D2
CRY_1	BnaA02g34040.1D2	BnaC02g40870.1D2
CRY_1	BnaA02g34130.1D2	BnaC02g40920.1D2
CRY_1	BnaA02g34300.1D2	BnaC02g41130.1D2
CRY_1	BnaA02g34410.1D2	BnaC02g41230.1D2
CRY_1	BnaA02g34520.1D2	BnaC02g41410.1D2
CRY_1	BnaA02g34570.1D2	BnaC02g41770.1D2
CRY_1	BnaA02g34600.1D2	BnaC02g41750.1D2
CRY_1	BnaA02g34640.1D2	BnaC02g41650.1D2
CRY_1	BnaA02g34760.1D2	BnaC02g17080.1D2
CRY_1	BnaA02g34790.1D2	BnaC02g17130.1D2
CRY_1	BnaA02g34810.1D2	BnaC02g17150.1D2
CRY_1	BnaA02g34850.1D2	BnaC02g41360.1D2
CRY_1	BnaA02g34890.1D2	BnaC02g41450.1D2
CRY_1	BnaA02g34910.1D2	BnaC02g41470.1D2
CRY_1	BnaA02g34980.1D2	BnaC02g41540.1D2
CRY_1	BnaA02g35010.1D2	BnaC02g41560.1D2
CRY_1	BnaA02g35150.1D2	BnaC02g41700.1D2

List of HE genes (A04 to C04)

Accession	Lost gene	Duplicated HE gene
H165	BnaA04g20610.1D2	BnaC04g40490.1D2
H165	BnaA04g20660.1D2	BnaC04g40540.1D2
H165	BnaA04g20700.1D2	BnaC04g40580.1D2
H165	BnaA04g20720.1D2	BnaC04g40600.1D2
H165	BnaA04g20830.1D2	BnaC04g40850.1D2
H165	BnaA04g20860.1D2	BnaC04g40880.1D2
H165	BnaA04g20920.1D2	BnaC04g40940.1D2
H165	BnaA04g20930.1D2	BnaC04g40950.1D2
H165	BnaA04g20970.1D2	BnaC04g41000.1D2
H165	BnaA04g21040.1D2	BnaC04g41100.1D2
H165	BnaA04g21080.1D2	BnaC04g41140.1D2
H165	BnaA04g21090.1D2	BnaC04g41150.1D2
H165	BnaA04g21170.1D2	BnaC04g42620.1D2
H165	BnaA04g21180.1D2	BnaC04g42630.1D2
H165	BnaA04g21250.1D2	BnaC04g42730.1D2
H165	BnaA04g21270.1D2	BnaC04g41190.1D2
H165	BnaA04g21280.1D2	BnaC04g41200.1D2
H165	BnaA04g21330.1D2	BnaC04g41260.1D2
H165	BnaA04g21380.1D2	BnaC04g41310.1D2

H165	BnaA04g21730.1D2	BnaC04g40740.1D2
H165	BnaA04g21740.1D2	BnaC04g40770.1D2
H165	BnaA04g21750.1D2	BnaC04g40780.1D2
H165	BnaA04g21760.1D2	BnaC04g40800.1D2
H165	BnaA04g21780.1D2	BnaC04g40800.1D2
H165	BnaA04g21790.1D2	BnaC04g40800.1D2
H165	BnaA04g21810.1D2	BnaC04g40710.1D2
H165	BnaA04g21820.1D2	BnaC04g40700.1D2
H165	BnaA04g21840.1D2	BnaC04g40690.1D2
H165	BnaA04g21850.1D2	BnaC04g40680.1D2
H165	BnaA04g21980.1D2	BnaC04g41980.1D2
H165	BnaA04g22000.1D2	BnaC04g41960.1D2
H165	BnaA04g22080.1D2	BnaC04g42520.1D2
H165	BnaA04g22110.1D2	BnaC04g42550.1D2
H165	BnaA04g22170.1D2	BnaC04g41880.1D2
H165	BnaA04g22190.1D2	BnaC04g41870.1D2
H165	BnaA04g22230.1D2	BnaC04g41830.1D2
H165	BnaA04g22260.1D2	BnaC04g41800.1D2
H165	BnaA04g22270.1D2	BnaC04g41780.1D2
H165	BnaA04g22280.1D2	BnaC04g41780.1D2
H165	BnaA04g22290.1D2	BnaC04g41760.1D2
H165	BnaA04g22350.1D2	BnaC04g42560.1D2
H165	BnaA04g22370.1D2	BnaC04g41680.1D2
H165	BnaA04g22490.1D2	BnaC04g41570.1D2
H165	BnaA04g22690.1D2	BnaC04g42250.1D2
H165	BnaA04g22720.1D2	BnaC04g42280.1D2
H165	BnaA04g22760.1D2	BnaC04g42330.1D2
H165	BnaA04g23000.1D2	BnaC04g42440.1D2
H165	BnaA04g23110.1D2	BnaC04g43010.1D2
H165	BnaA04g23250.1D2	BnaC04g43200.1D2
H165	BnaA04g23320.1D2	BnaC04g43270.1D2
H165	BnaA04g23390.1D2	BnaC04g43340.1D2
H165	BnaA04g23400.1D2	BnaC04g43350.1D2
H165	BnaA04g23660.1D2	BnaC04g42090.1D2
H165	BnaA04g23680.1D2	BnaC04g42110.1D2
H165	BnaA04g23710.1D2	BnaC04g42760.1D2
H165	BnaA04g23720.1D2	BnaC04g42770.1D2
H165	BnaA04g23730.1D2	BnaC04g42770.1D2
H165	BnaA04g23800.1D2	BnaC04g43640.1D2
H165	BnaA04g23840.1D2	BnaC04g43680.1D2
H165	BnaA04g23850.1D2	BnaC04g43690.1D2
H165	BnaA04g23880.1D2	BnaC04g43720.1D2
H165	BnaA04g23950.1D2	BnaC04g02750.1D2
H165	BnaA04g23950.1D2	BnaC04g43820.1D2
H165	BnaA04g24130.1D2	BnaC04g46010.1D2
H165	BnaA04g24180.1D2	BnaC04g46040.1D2
H165	BnaA04g24200.1D2	BnaC04g46060.1D2
H165	BnaA04g24280.1D2	BnaC04g43990.1D2
H165	BnaA04g24410.1D2	BnaC04g44060.1D2
H165	BnaA04g24420.1D2	BnaC04g44070.1D2
H165	BnaA04g24440.1D2	BnaC04g44100.1D2

H165	BnaA04g24450.1D2	BnaC04g44110.1D2
H165	BnaA04g24470.1D2	BnaC04g44140.1D2
H165	BnaA04g24480.1D2	BnaC04g44150.1D2
H165	BnaA04g24680.1D2	BnaC04g44370.1D2
H165	BnaA04g24770.1D2	BnaC04g44460.1D2
H165	BnaA04g24780.1D2	BnaC04g44470.1D2
H165	BnaA04g24830.1D2	BnaC04g44520.1D2
H165	BnaA04g24850.1D2	BnaC04g44540.1D2
H165	BnaA04g24860.1D2	BnaC04g44550.1D2
H165	BnaA04g24880.1D2	BnaC04g44570.1D2
H165	BnaA04g24890.1D2	BnaC04g44580.1D2
H165	BnaA04g24950.1D2	BnaC04g44640.1D2
H165	BnaA04g25000.1D2	BnaC04g44680.1D2
H165	BnaA04g25050.1D2	BnaC04g44730.1D2
H165	BnaA04g25130.1D2	BnaC04g44810.1D2
H165	BnaA04g25290.1D2	BnaC04g45010.1D2
H165	BnaA04g25360.1D2	BnaC04g45120.1D2
H165	BnaA04g25380.1D2	BnaC04g45140.1D2
H165	BnaA04g25580.1D2	BnaC04g45920.1D2
H165	BnaA04g25600.1D2	BnaC04g45930.1D2
H165	BnaA04g25610.1D2	BnaC04g45930.1D2
H165	BnaA04g25650.1D2	BnaC04g45310.1D2
H165	BnaA04g25680.1D2	BnaC04g45350.1D2
H165	BnaA04g25710.1D2	BnaC04g45400.1D2
H165	BnaA04g25740.1D2	BnaC04g45430.1D2
H165	BnaA04g25800.1D2	BnaC04g45490.1D2
H165	BnaA04g25820.1D2	BnaC04g45510.1D2
H165	BnaA04g25860.1D2	BnaC04g45540.1D2
H165	BnaA04g25870.1D2	BnaC04g45550.1D2
H165	BnaA04g25880.1D2	BnaC04g45560.1D2
H165	BnaA04g25940.1D2	BnaC04g45620.1D2
H165	BnaA04g25990.1D2	BnaC04g45680.1D2
H165	BnaA04g26020.1D2	BnaC04g45720.1D2
H165	BnaA04g26040.1D2	BnaC04g45750.1D2
H165	BnaA04g26070.1D2	BnaC04g45770.1D2
H165	BnaA04g26090.1D2	BnaC04g45790.1D2
H165	BnaA04g26200.1D2	BnaC04g45840.1D2
H165	BnaA04g26210.1D2	BnaC04g45830.1D2
H165	BnaA04g26250.1D2	BnaC04g46570.1D2
H165	BnaA04g26270.1D2	BnaC04g46550.1D2
H165	BnaA04g26300.1D2	BnaC04g46500.1D2
H165	BnaA04g26360.1D2	BnaC04g46420.1D2
H165	BnaA04g26390.1D2	BnaC04g46390.1D2
H165	BnaA04g26470.1D2	BnaC04g46310.1D2
H165	BnaA04g26570.1D2	BnaC04g46130.1D2
H165	BnaA04g26690.1D2	BnaC04g45860.1D2

List of HE genes (A07 to C06)

Accession	Lost gene	Duplicated HE gene
R76	BnaA07g31690.1D2	BnaC06g31140.1D2
R76	BnaA07g31750.1D2	BnaC06g31190.1D2

R76	BnaA07g31930.1D2	BnaC06g31420.1D2
R76	BnaA07g31940.1D2	BnaC06g31430.1D2
R76	BnaA07g31950.1D2	BnaC06g31440.1D2
R76	BnaA07g31960.1D2	BnaC06g31450.1D2
R76	BnaA07g31970.1D2	BnaC06g31460.1D2
R76	BnaA07g31990.1D2	BnaC06g31480.1D2
R76	BnaA07g32010.1D2	BnaC06g31500.1D2
R76	BnaA07g32060.1D2	BnaC06g31870.1D2
R76	BnaA07g32070.1D2	BnaC06g31880.1D2
R76	BnaA07g32100.1D2	BnaC06g31920.1D2
R76	BnaA07g32130.1D2	BnaC06g31950.1D2
R76	BnaA07g32140.1D2	BnaC06g31960.1D2
R76	BnaA07g32200.1D2	BnaC06g32040.1D2
R76	BnaA07g32220.1D2	BnaC06g32020.1D2
R76	BnaA07g32400.1D2	BnaC06g32240.1D2
R76	BnaA07g32680.1D2	BnaC06g32520.1D2
R76	BnaA07g32750.1D2	BnaC06g32580.1D2
R76	BnaA07g32790.1D2	BnaC06g32620.1D2
R76	BnaA07g32810.1D2	BnaC06g32640.1D2
R76	BnaA07g33030.1D2	BnaC06g32900.1D2
R76	BnaA07g33060.1D2	BnaC06g32940.1D2
R76	BnaA07g33210.1D2	BnaC06g33130.1D2
R76	BnaA07g33250.1D2	BnaC06g33150.1D2
R76	BnaA07g33260.1D2	BnaC06g33160.1D2
R76	BnaA07g33270.1D2	BnaC06g33170.1D2
R76	BnaA07g33280.1D2	BnaC06g33180.1D2
R76	BnaA07g33330.1D2	BnaC06g33230.1D2
R76	BnaA07g33340.1D2	BnaC06g33240.1D2
R76	BnaA07g33350.1D2	BnaC06g33250.1D2
R76	BnaA07g33400.1D2	BnaC06g33320.1D2
R76	BnaA07g33410.1D2	BnaC06g33330.1D2
R76	BnaA07g33460.1D2	BnaC06g33390.1D2
R76	BnaA07g33540.1D2	BnaC06g33480.1D2
R76	BnaA07g33580.1D2	BnaC06g33660.1D2
R76	BnaA07g33590.1D2	BnaC06g33670.1D2
R76	BnaA07g33630.1D2	BnaC06g33700.1D2
R76	BnaA07g33640.1D2	BnaC06g33710.1D2
R76	BnaA07g33660.1D2	BnaC06g33730.1D2
R76	BnaA07g33700.1D2	BnaC06g33790.1D2
R76	BnaA07g33710.1D2	BnaC06g33800.1D2
R76	BnaA07g33750.1D2	BnaC06g33840.1D2
R76	BnaA07g33890.1D2	BnaC06g33970.1D2
R76	BnaA07g33910.1D2	BnaC06g33990.1D2
R76	BnaA07g34000.1D2	BnaC06g34120.1D2
R76	BnaA07g34030.1D2	BnaC06g34150.1D2
R76	BnaA07g34060.1D2	BnaC06g34180.1D2
R76	BnaA07g34070.1D2	BnaC06g34190.1D2
R76	BnaA07g34120.1D2	BnaC06g34240.1D2
R76	BnaA07g34140.1D2	BnaC06g34260.1D2
R76	BnaA07g34210.1D2	BnaC06g34320.1D2
R76	BnaA07g34230.1D2	BnaC06g34340.1D2

R76	BnaA07g34250.1D2	BnaC06g34360.1D2
R76	BnaA07g34280.1D2	BnaC06g34380.1D2
R76	BnaA07g34310.1D2	BnaC06g34410.1D2
R76	BnaA07g34420.1D2	BnaC06g34510.1D2
R76	BnaA07g34440.1D2	BnaC06g34530.1D2
R76	BnaA07g34460.1D2	BnaC06g34550.1D2
R76	BnaA07g34560.1D2	BnaC06g34650.1D2
R76	BnaA07g34830.1D2	BnaC06g34960.1D2
R76	BnaA07g34840.1D2	BnaC06g34970.1D2
R76	BnaA07g34850.1D2	BnaC06g34980.1D2
R76	BnaA07g34860.1D2	BnaC06g34990.1D2
R76	BnaA07g34920.1D2	BnaC06g35060.1D2
R76	BnaA07g34960.1D2	BnaC06g35090.1D2
R76	BnaA07g34970.1D2	BnaC06g35100.1D2
R76	BnaA07g34980.1D2	BnaC06g35110.1D2
R76	BnaA07g35070.1D2	BnaC06g35350.1D2
R76	BnaA07g35080.1D2	BnaC06g35360.1D2
R76	BnaA07g35120.1D2	BnaC06g33580.1D2
R76	BnaA07g35130.1D2	BnaC06g33570.1D2
R76	BnaA07g35150.1D2	BnaC06g33550.1D2
R76	BnaA07g35250.1D2	BnaC06g34000.1D2
R76	BnaA07g35260.1D2	BnaC06g33620.1D2
R76	BnaA07g35350.1D2	BnaC06g31040.1D2
R76	BnaA07g35370.1D2	BnaC06g31010.1D2
R76	BnaA07g35420.1D2	BnaC06g30950.1D2
R76	BnaA07g35450.1D2	BnaC06g30920.1D2
R76	BnaA07g35460.1D2	BnaC06g31670.1D2
R76	BnaA07g35470.1D2	BnaC06g31670.1D2
R76	BnaA07g35530.1D2	BnaC06g30880.1D2
RS_4_6	BnaA07g31570.1D2	BnaC06g31070.1D2
RS_4_6	BnaA07g31640.1D2	BnaC06g31100.1D2
RS_4_6	BnaA07g31690.1D2	BnaC06g31140.1D2
RS_4_6	BnaA07g31740.1D2	BnaC06g31170.1D2
RS_4_6	BnaA07g31790.1D2	BnaC06g31280.1D2
RS_4_6	BnaA07g31810.1D2	BnaC06g31300.1D2
RS_4_6	BnaA07g31840.1D2	BnaC06g31330.1D2
RS_4_6	BnaA07g31970.1D2	BnaC06g31460.1D2
RS_4_6	BnaA07g32070.1D2	BnaC06g31880.1D2
RS_4_6	BnaA07g32200.1D2	BnaC06g32040.1D2
RS_4_6	BnaA07g32220.1D2	BnaC06g32020.1D2
RS_4_6	BnaA07g32240.1D2	BnaC06g32070.1D2
RS_4_6	BnaA07g32320.1D2	BnaC06g32170.1D2
RS_4_6	BnaA07g32330.1D2	BnaC06g32180.1D2
RS_4_6	BnaA07g32400.1D2	BnaC06g32240.1D2
RS_4_6	BnaA07g32410.1D2	BnaC06g32250.1D2
RS_4_6	BnaA07g32680.1D2	BnaC06g32520.1D2
RS_4_6	BnaA07g32740.1D2	BnaC06g32570.1D2
RS_4_6	BnaA07g32750.1D2	BnaC06g32580.1D2
RS_4_6	BnaA07g32790.1D2	BnaC06g32620.1D2
RS_4_6	BnaA07g32810.1D2	BnaC06g32640.1D2
RS_4_6	BnaA07g33030.1D2	BnaC06g32900.1D2

RS_4_6	BnaA07g33060.1D2	BnaC06g32940.1D2
RS_4_6	BnaA07g33070.1D2	BnaC06g32950.1D2
RS_4_6	BnaA07g33170.1D2	BnaC06g33100.1D2
RS_4_6	BnaA07g33260.1D2	BnaC06g33160.1D2
RS_4_6	BnaA07g33270.1D2	BnaC06g33170.1D2
RS_4_6	BnaA07g33280.1D2	BnaC06g33180.1D2
RS_4_6	BnaA07g33350.1D2	BnaC06g33250.1D2
RS_4_6	BnaA07g33540.1D2	BnaC06g33480.1D2
RS_4_6	BnaA07g33580.1D2	BnaC06g33660.1D2
RS_4_6	BnaA07g33590.1D2	BnaC06g33670.1D2
RS_4_6	BnaA07g33630.1D2	BnaC06g33700.1D2
RS_4_6	BnaA07g33640.1D2	BnaC06g33710.1D2
RS_4_6	BnaA07g33670.1D2	BnaC06g33740.1D2
RS_4_6	BnaA07g33710.1D2	BnaC06g33800.1D2
RS_4_6	BnaA07g33860.1D2	BnaC06g33940.1D2
RS_4_6	BnaA07g33880.1D2	BnaC06g33960.1D2
RS_4_6	BnaA07g33970.1D2	BnaC06g34060.1D2
RS_4_6	BnaA07g33980.1D2	BnaC06g34070.1D2
RS_4_6	BnaA07g34000.1D2	BnaC06g34120.1D2
RS_4_6	BnaA07g34010.1D2	BnaC06g34130.1D2
RS_4_6	BnaA07g34020.1D2	BnaC06g34140.1D2
RS_4_6	BnaA07g34030.1D2	BnaC06g34150.1D2
RS_4_6	BnaA07g34090.1D2	BnaC06g34210.1D2
RS_4_6	BnaA07g34170.1D2	BnaC06g34280.1D2
RS_4_6	BnaA07g34210.1D2	BnaC06g34320.1D2
RS_4_6	BnaA07g34410.1D2	BnaC06g34500.1D2
RS_4_6	BnaA07g34420.1D2	BnaC06g34510.1D2
RS_4_6	BnaA07g34440.1D2	BnaC06g34530.1D2
RS_4_6	BnaA07g34520.1D2	BnaC06g34610.1D2
RS_4_6	BnaA07g34540.1D2	BnaC06g34630.1D2
RS_4_6	BnaA07g34830.1D2	BnaC06g34960.1D2
RS_4_6	BnaA07g34920.1D2	BnaC06g35060.1D2
RS_4_6	BnaA07g34960.1D2	BnaC06g35090.1D2
RS_4_6	BnaA07g34980.1D2	BnaC06g35110.1D2
RS_4_6	BnaA07g35000.1D2	BnaC06g35130.1D2
RS_4_6	BnaA07g35010.1D2	BnaC06g35280.1D2
RS_4_6	BnaA07g35070.1D2	BnaC06g35350.1D2
RS_4_6	BnaA07g35130.1D2	BnaC06g33570.1D2
RS_4_6	BnaA07g35150.1D2	BnaC06g33550.1D2
RS_4_6	BnaA07g35270.1D2	BnaC06g32140.1D2
RS_4_6	BnaA07g35350.1D2	BnaC06g31040.1D2
RS_4_6	BnaA07g35370.1D2	BnaC06g31010.1D2

List of HE genes (A09 to C08)

**Accession**	**Lost gene**	**Duplicated HE gene**
H149	BnaA09g45690.1D2	BnaC08g34770.1D2
H149	BnaA09g45750.1D2	BnaC08g34860.1D2
H149	BnaA09g45760.1D2	BnaC08g34870.1D2
H149	BnaA09g45910.1D2	BnaC08g34220.1D2
H149	BnaA09g45970.1D2	BnaC08g34140.1D2
H149	BnaA09g46000.1D2	BnaC08g34130.1D2

H149	BnaA09g46040.1D2	BnaC08g33830.1D2
H149	BnaA09g46050.1D2	BnaC08g33830.1D2
H149	BnaA09g46070.1D2	BnaC08g33840.1D2
H149	BnaA09g46080.1D2	BnaC08g33880.1D2
H149	BnaA09g46090.1D2	BnaC08g33890.1D2
H149	BnaA09g46140.1D2	BnaC08g33950.1D2
H149	BnaA09g46210.1D2	BnaC08g34020.1D2
H149	BnaA09g46480.1D2	BnaC08g33790.1D2
H149	BnaA09g46710.1D2	BnaC08g34390.1D2
H149	BnaA09g46740.1D2	BnaC08g34420.1D2
H149	BnaA09g46770.1D2	BnaC08g34450.1D2
H149	BnaA09g46790.1D2	BnaC08g34470.1D2
H149	BnaA09g46850.1D2	BnaC08g34540.1D2
H149	BnaA09g46930.1D2	BnaC08g34650.1D2
H149	BnaA09g46940.1D2	BnaC08g34660.1D2
H149	BnaA09g46980.1D2	BnaC08g34910.1D2
H149	BnaA09g46990.1D2	BnaC08g34910.1D2
H149	BnaA09g47000.1D2	BnaC08g34920.1D2
H149	BnaA09g47010.1D2	BnaC08g34930.1D2
H149	BnaA09g47040.1D2	BnaC08g34970.1D2
H149	BnaA09g47090.1D2	BnaC08g35030.1D2
H149	BnaA09g47190.1D2	BnaC08g35120.1D2
H149	BnaA09g47300.1D2	BnaC08g35560.1D2
H149	BnaA09g47320.1D2	BnaC08g35580.1D2
H149	BnaA09g47340.1D2	BnaC08g35600.1D2
H149	BnaA09g47360.1D2	BnaC08g35620.1D2
H149	BnaA09g47420.1D2	BnaC08g35680.1D2
H149	BnaA09g47510.1D2	BnaC08g35770.1D2
H149	BnaA09g47530.1D2	BnaC08g35790.1D2
H149	BnaA09g47620.1D2	BnaC08g35890.1D2
H149	BnaA09g47710.1D2	BnaC08g35950.1D2
H149	BnaA09g47720.1D2	BnaC08g35960.1D2
H149	BnaA09g47810.1D2	BnaC08g36020.1D2
H149	BnaA09g47840.1D2	BnaC08g36060.1D2
H149	BnaA09g47900.1D2	BnaC08g36170.1D2
H149	BnaA09g47930.1D2	BnaC08g36200.1D2
H149	BnaA09g47940.1D2	BnaC08g36210.1D2
H149	BnaA09g47980.1D2	BnaC08g36240.1D2
H149	BnaA09g48000.1D2	BnaC08g36280.1D2
H149	BnaA09g48020.1D2	BnaC08g36300.1D2
H149	BnaA09g48070.1D2	BnaC08g36340.1D2
H149	BnaA09g48080.1D2	BnaC08g36350.1D2
H149	BnaA09g48140.1D2	BnaC08g36400.1D2
H149	BnaA09g48160.1D2	BnaC08g36420.1D2
H149	BnaA09g48180.1D2	BnaC08g36440.1D2
H149	BnaA09g48220.1D2	BnaC08g36470.1D2
H149	BnaA09g48230.1D2	BnaC08g36480.1D2
H149	BnaA09g48240.1D2	BnaC08g36490.1D2
H149	BnaA09g48250.1D2	BnaC08g36490.1D2
H149	BnaA09g48260.1D2	BnaC08g36500.1D2
H149	BnaA09g48270.1D2	BnaC08g36510.1D2

H149	BnaA09g48490.1D2	BnaC08g36810.1D2
H149	BnaA09g48510.1D2	BnaC08g36840.1D2
H149	BnaA09g48560.1D2	BnaC08g36900.1D2
H149	BnaA09g48580.1D2	BnaC08g36920.1D2
H149	BnaA09g48630.1D2	BnaC08g37000.1D2
H149	BnaA09g48670.1D2	BnaC08g37120.1D2
H149	BnaA09g48750.1D2	BnaC08g37230.1D2
H149	BnaA09g48860.1D2	BnaC08g37410.1D2
H149	BnaA09g48940.1D2	BnaC08g37330.1D2
H149	BnaA09g48950.1D2	BnaC08g37320.1D2
H149	BnaA09g49370.1D2	BnaC08g37870.1D2
H149	BnaA09g49430.1D2	BnaC08g37860.1D2
H149	BnaA09g49450.1D2	BnaC08g37870.1D2
H149	BnaA09g49470.1D2	BnaC08g37890.1D2
H149	BnaA09g49480.1D2	BnaC08g37900.1D2
H149	BnaA09g49490.1D2	BnaC08g37910.1D2
H149	BnaA09g49500.1D2	BnaC08g37920.1D2
H149	BnaA09g49640.1D2	BnaC08g38080.1D2
H149	BnaA09g49660.1D2	BnaC08g38100.1D2
H149	BnaA09g49670.1D2	BnaC08g38110.1D2
H149	BnaA09g49690.1D2	BnaC08g38130.1D2
H149	BnaA09g49710.1D2	BnaC08g38150.1D2
H149	BnaA09g49850.1D2	BnaC08g38320.1D2
H149	BnaA09g49860.1D2	BnaC08g38330.1D2
H149	BnaA09g49890.1D2	BnaC08g38360.1D2
H149	BnaA09g49930.1D2	BnaC08g38430.1D2
H149	BnaA09g49990.1D2	BnaC08g38510.1D2
H149	BnaA09g50030.1D2	BnaC08g38540.1D2
H149	BnaA09g50050.1D2	BnaC08g38560.1D2
H149	BnaA09g50110.1D2	BnaC08g38620.1D2
H149	BnaA09g50130.1D2	BnaC08g38640.1D2
H149	BnaA09g50150.1D2	BnaC08g38660.1D2
H149	BnaA09g50180.1D2	BnaC08g40850.1D2
H149	BnaA09g50460.1D2	BnaC08g40030.1D2
H149	BnaA09g50620.1D2	BnaC08g39900.1D2
H149	BnaA09g50650.1D2	BnaC08g39860.1D2
H149	BnaA09g50670.1D2	BnaC08g39820.1D2
H149	BnaA09g50680.1D2	BnaC08g39810.1D2
H149	BnaA09g50710.1D2	BnaC08g39780.1D2
H149	BnaA09g50760.1D2	BnaC08g39710.1D2
H149	BnaA09g50780.1D2	BnaC08g39690.1D2
H149	BnaA09g50820.1D2	BnaC08g39620.1D2
H149	BnaA09g50830.1D2	BnaC08g39630.1D2
H149	BnaA09g50900.1D2	BnaC08g39570.1D2
H149	BnaA09g51040.1D2	BnaC08g39410.1D2
H149	BnaA09g51070.1D2	BnaC08g39380.1D2
H149	BnaA09g51110.1D2	BnaC08g39340.1D2
H149	BnaA09g51130.1D2	BnaC08g39300.1D2
H149	BnaA09g51270.1D2	BnaC08g37740.1D2
H149	BnaA09g51310.1D2	BnaC08g37700.1D2
H149	BnaA09g51420.1D2	BnaC08g38380.1D2

H149	BnaA09g51430.1D2	BnaC08g38520.1D2
H149	BnaA09g51440.1D2	BnaC08g40780.1D2
H149	BnaA09g51480.1D2	BnaC08g37930.1D2
H149	BnaA09g51510.1D2	BnaC08g39270.1D2
H149	BnaA09g51520.1D2	BnaC08g39260.1D2
H149	BnaA09g51620.1D2	BnaC08g40070.1D2
H149	BnaA09g51900.1D2	BnaC08g39000.1D2
H149	BnaA09g51950.1D2	BnaC08g38910.1D2
H149	BnaA09g51990.1D2	BnaC08g38870.1D2
H149	BnaA09g52060.1D2	BnaC08g38800.1D2
H149	BnaA09g52230.1D2	BnaC08g40920.1D2
H149	BnaA09g52270.1D2	BnaC08g40960.1D2
H149	BnaA09g52330.1D2	BnaC08g41070.1D2
H149	BnaA09g52340.1D2	BnaC08g41070.1D2
S_39	BnaA09g47690.1D2	BnaC08g35930.1D2
S_39	BnaA09g47840.1D2	BnaC08g36060.1D2
S_39	BnaA09g47930.1D2	BnaC08g36200.1D2
S_39	BnaA09g47940.1D2	BnaC08g36210.1D2
S_39	BnaA09g47950.1D2	BnaC08g36220.1D2
S_39	BnaA09g47970.1D2	BnaC08g36240.1D2
S_39	BnaA09g47980.1D2	BnaC08g36240.1D2
S_39	BnaA09g48000.1D2	BnaC08g36280.1D2
S_39	BnaA09g48060.1D2	BnaC08g36320.1D2
S_39	BnaA09g48090.1D2	BnaC08g36360.1D2
S_39	BnaA09g48100.1D2	BnaC08g36370.1D2
S_39	BnaA09g48120.1D2	BnaC08g36390.1D2
S_39	BnaA09g48160.1D2	BnaC08g36420.1D2
S_39	BnaA09g48180.1D2	BnaC08g36440.1D2
S_39	BnaA09g48240.1D2	BnaC08g36490.1D2
S_39	BnaA09g48250.1D2	BnaC08g36490.1D2
S_39	BnaA09g48480.1D2	BnaC08g36800.1D2
S_39	BnaA09g48560.1D2	BnaC08g36900.1D2
S_39	BnaA09g48630.1D2	BnaC08g37000.1D2
S_39	BnaA09g48660.1D2	BnaC08g37050.1D2
S_39	BnaA09g48750.1D2	BnaC08g37230.1D2
S_39	BnaA09g48950.1D2	BnaC08g37320.1D2
S_39	BnaA09g49090.1D2	BnaC08g37450.1D2
S_39	BnaA09g49100.1D2	BnaC08g35390.1D2
S_39	BnaA09g49340.1D2	BnaC08g37770.1D2
S_39	BnaA09g49410.1D2	BnaC08g37840.1D2
S_39	BnaA09g49430.1D2	BnaC08g37860.1D2
S_39	BnaA09g49450.1D2	BnaC08g37870.1D2
S_39	BnaA09g49470.1D2	BnaC08g37890.1D2
S_39	BnaA09g49480.1D2	BnaC08g37900.1D2
S_39	BnaA09g49490.1D2	BnaC08g37910.1D2
S_39	BnaA09g49500.1D2	BnaC08g37920.1D2
S_39	BnaA09g49640.1D2	BnaC08g38080.1D2
S_39	BnaA09g49660.1D2	BnaC08g38100.1D2
S_39	BnaA09g49690.1D2	BnaC08g38130.1D2
S_39	BnaA09g49700.1D2	BnaC08g38140.1D2
S_39	BnaA09g49710.1D2	BnaC08g38150.1D2

S_39	BnaA09g49770.1D2	BnaC08g38210.1D2
S_39	BnaA09g49830.1D2	BnaC08g38300.1D2
S_39	BnaA09g49860.1D2	BnaC08g38330.1D2
S_39	BnaA09g49930.1D2	BnaC08g38430.1D2
S_39	BnaA09g49990.1D2	BnaC08g38510.1D2
S_39	BnaA09g50030.1D2	BnaC08g38540.1D2
S_39	BnaA09g50100.1D2	BnaC08g38610.1D2
S_39	BnaA09g50130.1D2	BnaC08g38640.1D2
S_39	BnaA09g50180.1D2	BnaC08g40850.1D2
S_39	BnaA09g50260.1D2	BnaC08g40410.1D2
S_39	BnaA09g50330.1D2	BnaC08g40340.1D2
S_39	BnaA09g50340.1D2	BnaC08g40330.1D2
S_39	BnaA09g50410.1D2	BnaC08g40230.1D2
S_39	BnaA09g50460.1D2	BnaC08g40030.1D2
S_39	BnaA09g50540.1D2	BnaC08g39990.1D2
S_39	BnaA09g50550.1D2	BnaC08g39980.1D2
S_39	BnaA09g50580.1D2	BnaC08g39950.1D2
S_39	BnaA09g50620.1D2	BnaC08g39900.1D2
S_39	BnaA09g50670.1D2	BnaC08g39820.1D2
S_39	BnaA09g50680.1D2	BnaC08g39810.1D2
S_39	BnaA09g50710.1D2	BnaC08g39780.1D2
S_39	BnaA09g50760.1D2	BnaC08g39710.1D2
S_39	BnaA09g50770.1D2	BnaC08g39700.1D2
S_39	BnaA09g50790.1D2	BnaC08g39680.1D2
S_39	BnaA09g50830.1D2	BnaC08g39630.1D2
S_39	BnaA09g50900.1D2	BnaC08g39570.1D2
S_39	BnaA09g50930.1D2	BnaC08g39520.1D2
S_39	BnaA09g50940.1D2	BnaC08g39510.1D2
S_39	BnaA09g51030.1D2	BnaC08g39420.1D2
S_39	BnaA09g51040.1D2	BnaC08g39410.1D2
S_39	BnaA09g51070.1D2	BnaC08g39380.1D2
S_39	BnaA09g51080.1D2	BnaC08g39370.1D2
S_39	BnaA09g51290.1D2	BnaC08g37720.1D2
S_39	BnaA09g51300.1D2	BnaC08g37710.1D2
S_39	BnaA09g51320.1D2	BnaC08g37690.1D2
S_39	BnaA09g51430.1D2	BnaC08g38520.1D2
S_39	BnaA09g51470.1D2	BnaC08g38260.1D2
S_39	BnaA09g51480.1D2	BnaC08g37930.1D2
S_39	BnaA09g51630.1D2	BnaC08g40080.1D2
S_39	BnaA09g51690.1D2	BnaC08g40180.1D2
S_39	BnaA09g51700.1D2	BnaC08g40190.1D2

List of HE genes (A09 to C09)

**Accession**	**Lost gene**	**Duplicated HE gene**
Sensation	BnaA09g00940.1D2	BnaC09g01180.1D2
Sensation	BnaA09g01460.1D2	BnaC09g01130.1D2
Sensation	BnaA09g01520.1D2	BnaC09g01080.1D2
Sensation	BnaA09g01570.1D2	BnaC09g00020.1D2
Sensation	BnaA09g01580.1D2	BnaC09g00030.1D2
Sensation	BnaA09g01590.1D2	BnaC09g00040.1D2
Sensation	BnaA09g01630.1D2	BnaC09g00060.1D2

Sensation	BnaA09g01690.1D2	BnaC09g00130.1D2
Sensation	BnaA09g01700.1D2	BnaC09g00140.1D2
Sensation	BnaA09g01720.1D2	BnaC09g00170.1D2
Sensation	BnaA09g01740.1D2	BnaC09g00180.1D2
Sensation	BnaA09g01810.1D2	BnaC09g00270.1D2
Sensation	BnaA09g01820.1D2	BnaC09g00280.1D2
Sensation	BnaA09g01940.1D2	BnaC09g00450.1D2
Sensation	BnaA09g02010.1D2	BnaC09g00540.1D2
Sensation	BnaA09g02080.1D2	BnaC09g00590.1D2
Sensation	BnaA09g02090.1D2	BnaC09g00600.1D2
Sensation	BnaA09g02120.1D2	BnaC09g00630.1D2
Sensation	BnaA09g02140.1D2	BnaC09g00650.1D2
Sensation	BnaA09g02150.1D2	BnaC09g00660.1D2
Sensation	BnaA09g02280.1D2	BnaC09g00800.1D2
Sensation	BnaA09g02330.1D2	BnaC09g00860.1D2
Sensation	BnaA09g02640.1D2	BnaC09g01410.1D2
Sensation	BnaA09g02740.1D2	BnaC09g01470.1D2
Sensation	BnaA09g02780.1D2	BnaC09g01490.1D2
Sensation	BnaA09g02800.1D2	BnaC09g01510.1D2
Sensation	BnaA09g02810.1D2	BnaC09g01520.1D2
Sensation	BnaA09g02820.1D2	BnaC09g01530.1D2
Sensation	BnaA09g02890.1D2	BnaC09g01560.1D2
Sensation	BnaA09g02970.1D2	BnaC09g01710.1D2
Sensation	BnaA09g03150.1D2	BnaC09g01850.1D2
Sensation	BnaA09g03210.1D2	BnaC09g01910.1D2
Sensation	BnaA09g03410.1D2	BnaC09g02020.1D2
Sensation	BnaA09g03420.1D2	BnaC09g02030.1D2
Sensation	BnaA09g03530.1D2	BnaC09g02130.1D2
Sensation	BnaA09g03750.1D2	BnaC09g02310.1D2
Sensation	BnaA09g03770.1D2	BnaC09g02360.1D2
Sensation	BnaA09g03850.1D2	BnaC09g02410.1D2
Sensation	BnaA09g03900.1D2	BnaC09g02450.1D2
Sensation	BnaA09g04180.1D2	BnaC09g01230.1D2
Sensation	BnaA09g24780.1D2	BnaC09g01280.1D2
Nunsdale	BnaA09g02970.1D2	BnaC09g01710.1D2
Nunsdale	BnaA09g02980.1D2	BnaC09g01720.1D2
Nunsdale	BnaA09g02990.1D2	BnaC09g01730.1D2
Nunsdale	BnaA09g03000.1D2	BnaC09g01740.1D2
Nunsdale	BnaA09g03010.1D2	BnaC09g01750.1D2
Nunsdale	BnaA09g03020.1D2	BnaC09g01770.1D2
Nunsdale	BnaA09g03030.1D2	BnaC09g01780.1D2
Nunsdale	BnaA09g03150.1D2	BnaC09g01850.1D2
Nunsdale	BnaA09g03210.1D2	BnaC09g01910.1D2
Nunsdale	BnaA09g03310.1D2	BnaC09g03130.1D2
Nunsdale	BnaA09g03410.1D2	BnaC09g02020.1D2
Nunsdale	BnaA09g03480.1D2	BnaC09g02090.1D2
Nunsdale	BnaA09g03670.1D2	BnaC09g02240.1D2
Nunsdale	BnaA09g03720.1D2	BnaC09g02280.1D2
Nunsdale	BnaA09g03910.1D2	BnaC09g02460.1D2
Nunsdale	BnaA09g04460.1D2	BnaC09g02850.1D2
Nunsdale	BnaA09g04530.1D2	BnaC09g02910.1D2

Nunsdale	BnaA09g04540.1D2	BnaC09g02920.1D2
Nunsdale	BnaA09g04590.1D2	BnaC09g02960.1D2
Nunsdale	BnaA09g04600.1D2	BnaC09g02970.1D2
Nunsdale	BnaA09g04610.1D2	BnaC09g02980.1D2
Nunsdale	BnaA09g04640.1D2	BnaC09g03000.1D2
Nunsdale	BnaA09g04790.1D2	BnaC09g03410.1D2
Nunsdale	BnaA09g04890.1D2	BnaC09g03500.1D2
Nunsdale	BnaA09g04920.1D2	BnaC09g03540.1D2
Nunsdale	BnaA09g05350.1D2	BnaC09g03940.1D2
Nunsdale	BnaA09g05380.1D2	BnaC09g03960.1D2
Nunsdale	BnaA09g05390.1D2	BnaC09g03970.1D2
Nunsdale	BnaA09g06310.1D2	BnaC09g04930.1D2
Nunsdale	BnaA09g06940.1D2	BnaC09g05790.1D2
Nunsdale	BnaA09g07710.1D2	BnaC09g06790.1D2
Nunsdale	BnaA09g07970.1D2	BnaC09g07100.1D2
Nunsdale	BnaA09g24770.1D2	BnaC09g01270.1D2
G50	BnaA09g02270.1D2	BnaC09g00790.1D2
G50	BnaA09g02280.1D2	BnaC09g00800.1D2
G50	BnaA09g02510.1D2	BnaC09g01340.1D2
G50	BnaA09g02570.1D2	BnaC09g01370.1D2
G50	BnaA09g02740.1D2	BnaC09g01470.1D2
G50	BnaA09g02770.1D2	BnaC09g01480.1D2
G50	BnaA09g02780.1D2	BnaC09g01490.1D2
G50	BnaA09g02880.1D2	BnaC09g01570.1D2
G50	BnaA09g02910.1D2	BnaC09g01610.1D2
G50	BnaA09g02920.1D2	BnaC09g01640.1D2
G50	BnaA09g02930.1D2	BnaC09g01660.1D2
G50	BnaA09g02940.1D2	BnaC09g01670.1D2
G50	BnaA09g02960.1D2	BnaC09g01700.1D2
G50	BnaA09g02980.1D2	BnaC09g01720.1D2
G50	BnaA09g03000.1D2	BnaC09g01740.1D2
G50	BnaA09g03150.1D2	BnaC09g01850.1D2
G50	BnaA09g03160.1D2	BnaC09g01860.1D2
G50	BnaA09g03210.1D2	BnaC09g01910.1D2
G50	BnaA09g03420.1D2	BnaC09g02030.1D2
G50	BnaA09g03610.1D2	BnaC09g02180.1D2
G50	BnaA09g03670.1D2	BnaC09g02240.1D2
G50	BnaA09g03690.1D2	BnaC09g02260.1D2
G50	BnaA09g03700.1D2	BnaC09g02270.1D2
G50	BnaA09g03750.1D2	BnaC09g02310.1D2
G50	BnaA09g03770.1D2	BnaC09g02360.1D2
G50	BnaA09g03900.1D2	BnaC09g02450.1D2
G50	BnaA09g03910.1D2	BnaC09g02460.1D2
G50	BnaA09g04110.1D2	BnaC09g02570.1D2
G50	BnaA09g04220.1D2	BnaC09g01040.1D2
G50	BnaA09g04280.1D2	BnaC09g02650.1D2
G50	BnaA09g04430.1D2	BnaC09g02830.1D2
G50	BnaA09g04460.1D2	BnaC09g02850.1D2
G50	BnaA09g04530.1D2	BnaC09g02910.1D2
G50	BnaA09g04540.1D2	BnaC09g02920.1D2
G50	BnaA09g04590.1D2	BnaC09g02960.1D2

G50	BnaA09g04600.1D2	BnaC09g02970.1D2
G50	BnaA09g04610.1D2	BnaC09g02980.1D2
G50	BnaA09g04690.1D2	BnaC09g05720.1D2
G50	BnaA09g04790.1D2	BnaC09g03410.1D2
G50	BnaA09g04850.1D2	BnaC09g03470.1D2
G50	BnaA09g04890.1D2	BnaC09g03500.1D2
G50	BnaA09g05120.1D2	BnaC09g03710.1D2
G50	BnaA09g05130.1D2	BnaC09g03730.1D2
G50	BnaA09g05150.1D2	BnaC09g03760.1D2
G50	BnaA09g05190.1D2	BnaC09g05660.1D2
G50	BnaA09g05200.1D2	BnaC09g05670.1D2
G50	BnaA09g05350.1D2	BnaC09g03940.1D2
G50	BnaA09g05380.1D2	BnaC09g03960.1D2
G50	BnaA09g05390.1D2	BnaC09g03970.1D2
G50	BnaA09g05510.1D2	BnaC09g04140.1D2
G50	BnaA09g05530.1D2	BnaC09g04150.1D2
G50	BnaA09g05550.1D2	BnaC09g04180.1D2
G50	BnaA09g05660.1D2	BnaC09g04320.1D2
G50	BnaA09g06010.1D2	BnaC09g05700.1D2
G50	BnaA09g06030.1D2	BnaC09g04650.1D2
G50	BnaA09g06050.1D2	BnaC09g04670.1D2
G50	BnaA09g06060.1D2	BnaC09g04680.1D2
G50	BnaA09g06100.1D2	BnaC09g04730.1D2
G50	BnaA09g06150.1D2	BnaC09g05620.1D2
G50	BnaA09g06280.1D2	BnaC09g04890.1D2
G50	BnaA09g06300.1D2	BnaC09g04920.1D2
G50	BnaA09g06330.1D2	BnaC09g04950.1D2
G50	BnaA09g06340.1D2	BnaC09g05020.1D2
G50	BnaA09g06350.1D2	BnaC09g05030.1D2
G50	BnaA09g06360.1D2	BnaC09g05050.1D2
G50	BnaA09g06450.1D2	BnaC09g05080.1D2
G50	BnaA09g06460.1D2	BnaC09g05090.1D2
G50	BnaA09g06530.1D2	BnaC09g05160.1D2
G50	BnaA09g06540.1D2	BnaC09g05170.1D2
G50	BnaA09g06550.1D2	BnaC09g05180.1D2
G50	BnaA09g06640.1D2	BnaC09g05280.1D2
G50	BnaA09g06660.1D2	BnaC09g05300.1D2
G50	BnaA09g06770.1D2	BnaC09g05580.1D2
G50	BnaA09g06850.1D2	BnaC09g05470.1D2
G50	BnaA09g07280.1D2	BnaC09g06290.1D2
G50	BnaA09g07290.1D2	BnaC09g06310.1D2
G50	BnaA09g07330.1D2	BnaC09g06350.1D2
G50	BnaA09g07390.1D2	BnaC09g06400.1D2
G50	BnaA09g07410.1D2	BnaC09g06420.1D2
G50	BnaA09g07420.1D2	BnaC09g06430.1D2
G50	BnaA09g07440.1D2	BnaC09g06450.1D2
G50	BnaA09g07450.1D2	BnaC09g06460.1D2
G50	BnaA09g07470.1D2	BnaC09g06500.1D2
G50	BnaA09g07540.1D2	BnaC09g06590.1D2
G50	BnaA09g07620.1D2	BnaC09g06690.1D2
G50	BnaA09g07630.1D2	BnaC09g06700.1D2

G50	BnaA09g07660.1D2	BnaC09g06740.1D2
G50	BnaA09g07710.1D2	BnaC09g06790.1D2
G50	BnaA09g07790.1D2	BnaC09g08510.1D2
G50	BnaA09g07830.1D2	BnaC09g06970.1D2
G50	BnaA09g07840.1D2	BnaC09g06980.1D2
G50	BnaA09g07850.1D2	BnaC09g06990.1D2
G50	BnaA09g07860.1D2	BnaC09g08550.1D2
G50	BnaA09g07890.1D2	BnaC09g07030.1D2
G50	BnaA09g07910.1D2	BnaC09g07050.1D2
G50	BnaA09g07950.1D2	BnaC09g07090.1D2
G50	BnaA09g07970.1D2	BnaC09g07100.1D2
G50	BnaA09g07980.1D2	BnaC09g07130.1D2
G50	BnaA09g07990.1D2	BnaC09g07140.1D2
G50	BnaA09g08000.1D2	BnaC09g07150.1D2
G50	BnaA09g08140.1D2	BnaC09g07330.1D2
G50	BnaA09g08200.1D2	BnaC09g07380.1D2
G50	BnaA09g08250.1D2	BnaC09g07430.1D2
G50	BnaA09g08330.1D2	BnaC09g07680.1D2
G50	BnaA09g08360.1D2	BnaC09g07710.1D2
G50	BnaA09g08420.1D2	BnaC09g07780.1D2
G50	BnaA09g08440.1D2	BnaC09g07810.1D2
G50	BnaA09g08450.1D2	BnaC09g07820.1D2
G50	BnaA09g08480.1D2	BnaC09g07870.1D2
G50	BnaA09g08510.1D2	BnaC09g07920.1D2
G50	BnaA09g08530.1D2	BnaC09g07940.1D2
G50	BnaA09g08540.1D2	BnaC09g07950.1D2
G50	BnaA09g08570.1D2	BnaC09g07980.1D2
G50	BnaA09g08580.1D2	BnaC09g07990.1D2
G50	BnaA09g08600.1D2	BnaC09g08010.1D2
G50	BnaA09g08610.1D2	BnaC09g08020.1D2
G50	BnaA09g08620.1D2	BnaC09g08030.1D2
G50	BnaA09g08670.1D2	BnaC09g08060.1D2
G50	BnaA09g08810.1D2	BnaC09g07560.1D2
G50	BnaA09g08900.1D2	BnaC09g06890.1D2
G50	BnaA09g08940.1D2	BnaC09g06090.1D2
G50	BnaA09g08970.1D2	BnaC09g06140.1D2
G50	BnaA09g08990.1D2	BnaC09g06200.1D2
G50	BnaA09g09410.1D2	BnaC09g08810.1D2
G50	BnaA09g09440.1D2	BnaC09g08860.1D2
G50	BnaA09g09460.1D2	BnaC09g08880.1D2
G50	BnaA09g09510.1D2	BnaC09g08940.1D2
G50	BnaA09g09530.1D2	BnaC09g08960.1D2
G50	BnaA09g09580.1D2	BnaC09g09010.1D2
G50	BnaA09g09610.1D2	BnaC09g09040.1D2
G50	BnaA09g09760.1D2	BnaC09g09120.1D2
G50	BnaA09g09760.1D2	BnaC09g09130.1D2
G50	BnaA09g09770.1D2	BnaC09g09120.1D2
G50	BnaA09g09770.1D2	BnaC09g09130.1D2
G50	BnaA09g09810.1D2	BnaC09g09170.1D2
G50	BnaA09g09870.1D2	BnaC09g09230.1D2
G50	BnaA09g09880.1D2	BnaC09g09240.1D2

G50	BnaA09g10210.1D2	BnaC09g08470.1D2
G50	BnaA09g10220.1D2	BnaC09g08460.1D2
G50	BnaA09g10450.1D2	BnaC09g08650.1D2
G50	BnaA09g10470.1D2	BnaC09g08610.1D2
G50	BnaA09g24770.1D2	BnaC09g01270.1D2
H176	BnaA09g00920.1D2	BnaC09g01160.1D2
H176	BnaA09g01410.1D2	BnaC09g03180.1D2
H176	BnaA09g01510.1D2	BnaC09g01090.1D2
H176	BnaA09g01550.1D2	BnaC09g01050.1D2
H176	BnaA09g01580.1D2	BnaC09g00030.1D2
H176	BnaA09g01590.1D2	BnaC09g00040.1D2
H176	BnaA09g01630.1D2	BnaC09g00060.1D2
H176	BnaA09g01700.1D2	BnaC09g00140.1D2
H176	BnaA09g01720.1D2	BnaC09g00170.1D2
H176	BnaA09g01780.1D2	BnaC09g00220.1D2
H176	BnaA09g01790.1D2	BnaC09g00250.1D2
H176	BnaA09g01810.1D2	BnaC09g00270.1D2
H176	BnaA09g01820.1D2	BnaC09g00280.1D2
H176	BnaA09g02120.1D2	BnaC09g00630.1D2
H176	BnaA09g02280.1D2	BnaC09g00800.1D2
H176	BnaA09g02390.1D2	BnaC09g00960.1D2
H176	BnaA09g02570.1D2	BnaC09g01370.1D2
H176	BnaA09g02770.1D2	BnaC09g01480.1D2
H176	BnaA09g02910.1D2	BnaC09g01610.1D2
H176	BnaA09g02970.1D2	BnaC09g01710.1D2
H176	BnaA09g03010.1D2	BnaC09g01750.1D2
H176	BnaA09g03020.1D2	BnaC09g01770.1D2
H176	BnaA09g03210.1D2	BnaC09g01910.1D2
H176	BnaA09g03530.1D2	BnaC09g02130.1D2
H176	BnaA09g03610.1D2	BnaC09g02180.1D2
H176	BnaA09g03670.1D2	BnaC09g02240.1D2
H176	BnaA09g03690.1D2	BnaC09g02260.1D2
H176	BnaA09g03750.1D2	BnaC09g02310.1D2
H176	BnaA09g03770.1D2	BnaC09g02360.1D2
H176	BnaA09g03900.1D2	BnaC09g02450.1D2
H176	BnaA09g03930.1D2	BnaC09g02480.1D2
H176	BnaA09g04070.1D2	BnaC09g02520.1D2
H176	BnaA09g04210.1D2	BnaC09g01040.1D2
H176	BnaA09g04220.1D2	BnaC09g01040.1D2
H176	BnaA09g04330.1D2	BnaC09g02690.1D2
H176	BnaA09g04410.1D2	BnaC09g02800.1D2
H176	BnaA09g04460.1D2	BnaC09g02850.1D2
H176	BnaA09g04530.1D2	BnaC09g02910.1D2
H176	BnaA09g04540.1D2	BnaC09g02920.1D2
H176	BnaA09g04590.1D2	BnaC09g02960.1D2
H176	BnaA09g04600.1D2	BnaC09g02970.1D2
H176	BnaA09g04610.1D2	BnaC09g02980.1D2
H176	BnaA09g04630.1D2	BnaC09g02990.1D2
H176	BnaA09g04640.1D2	BnaC09g03000.1D2
H176	BnaA09g04690.1D2	BnaC09g05720.1D2
H176	BnaA09g04850.1D2	BnaC09g03470.1D2

H176	BnaA09g04870.1D2	BnaC09g03480.1D2
H176	BnaA09g04940.1D2	BnaC09g03550.1D2
H176	BnaA09g05020.1D2	BnaC09g03620.1D2
H176	BnaA09g05120.1D2	BnaC09g03710.1D2
H176	BnaA09g05130.1D2	BnaC09g03730.1D2
H176	BnaA09g05380.1D2	BnaC09g03960.1D2
H176	BnaA09g05390.1D2	BnaC09g03970.1D2
H176	BnaA09g05510.1D2	BnaC09g04140.1D2
H176	BnaA09g24770.1D2	BnaC09g01270.1D2
R53	BnaA09g00940.1D2	BnaC09g01180.1D2
R53	BnaA09g01340.1D2	BnaC09g03250.1D2
R53	BnaA09g01700.1D2	BnaC09g00140.1D2
R53	BnaA09g01780.1D2	BnaC09g00220.1D2
R53	BnaA09g01890.1D2	BnaC09g00360.1D2
R53	BnaA09g01990.1D2	BnaC09g00510.1D2
R53	BnaA09g02010.1D2	BnaC09g00540.1D2
R53	BnaA09g02030.1D2	BnaC09g00560.1D2
R53	BnaA09g02080.1D2	BnaC09g00590.1D2
R53	BnaA09g02090.1D2	BnaC09g00600.1D2
R53	BnaA09g02120.1D2	BnaC09g00630.1D2
R53	BnaA09g02150.1D2	BnaC09g00660.1D2
R53	BnaA09g02280.1D2	BnaC09g00800.1D2
R53	BnaA09g02330.1D2	BnaC09g00860.1D2
R53	BnaA09g05130.1D2	BnaC09g03730.1D2
R53	BnaA09g05300.1D2	BnaC09g03880.1D2
R53	BnaA09g05350.1D2	BnaC09g03940.1D2
R53	BnaA09g05380.1D2	BnaC09g03960.1D2
R53	BnaA09g05390.1D2	BnaC09g03970.1D2
R53	BnaA09g05510.1D2	BnaC09g04140.1D2
R53	BnaA09g05530.1D2	BnaC09g04150.1D2
R53	BnaA09g05660.1D2	BnaC09g04320.1D2
R53	BnaA09g05880.1D2	BnaC09g04520.1D2
R53	BnaA09g06030.1D2	BnaC09g04650.1D2
R53	BnaA09g06050.1D2	BnaC09g04670.1D2
R53	BnaA09g06060.1D2	BnaC09g04680.1D2
R53	BnaA09g06150.1D2	BnaC09g05620.1D2
R53	BnaA09g06190.1D2	BnaC09g04800.1D2
R53	BnaA09g06300.1D2	BnaC09g04920.1D2
R53	BnaA09g06310.1D2	BnaC09g04930.1D2
R53	BnaA09g06330.1D2	BnaC09g04950.1D2
R53	BnaA09g06350.1D2	BnaC09g05030.1D2
R53	BnaA09g06390.1D2	BnaC09g05070.1D2
R53	BnaA09g06450.1D2	BnaC09g05080.1D2
R53	BnaA09g06460.1D2	BnaC09g05090.1D2
R53	BnaA09g06480.1D2	BnaC09g05110.1D2
R53	BnaA09g06530.1D2	BnaC09g05160.1D2
R53	BnaA09g06540.1D2	BnaC09g05170.1D2
R53	BnaA09g06550.1D2	BnaC09g05180.1D2
R53	BnaA09g06560.1D2	BnaC09g05200.1D2
R53	BnaA09g06570.1D2	BnaC09g05210.1D2
R53	BnaA09g06580.1D2	BnaC09g05220.1D2

R53	BnaA09g06590.1D2	BnaC09g05230.1D2
R53	BnaA09g06610.1D2	BnaC09g05240.1D2
R53	BnaA09g06620.1D2	BnaC09g05250.1D2
R53	BnaA09g06640.1D2	BnaC09g05280.1D2
R53	BnaA09g06650.1D2	BnaC09g05290.1D2
R53	BnaA09g06720.1D2	BnaC09g05390.1D2
R53	BnaA09g06770.1D2	BnaC09g05580.1D2
R53	BnaA09g06850.1D2	BnaC09g05470.1D2
R53	BnaA09g06860.1D2	BnaC09g05480.1D2
R53	BnaA09g07110.1D2	BnaC09g09360.1D2
R53	BnaA09g07310.1D2	BnaC09g06330.1D2
R53	BnaA09g07330.1D2	BnaC09g06350.1D2
R53	BnaA09g07380.1D2	BnaC09g06390.1D2
R53	BnaA09g07390.1D2	BnaC09g06400.1D2
R53	BnaA09g07410.1D2	BnaC09g06420.1D2
R53	BnaA09g07420.1D2	BnaC09g06430.1D2
R53	BnaA09g07440.1D2	BnaC09g06450.1D2
R53	BnaA09g07450.1D2	BnaC09g06460.1D2
R53	BnaA09g07460.1D2	BnaC09g06480.1D2
R53	BnaA09g07480.1D2	BnaC09g06510.1D2
R53	BnaA09g07490.1D2	BnaC09g06520.1D2
R53	BnaA09g07540.1D2	BnaC09g06590.1D2
R53	BnaA09g07650.1D2	BnaC09g06720.1D2
R53	BnaA09g07710.1D2	BnaC09g06790.1D2
R53	BnaA09g07740.1D2	BnaC09g06810.1D2
R53	BnaA09g08890.1D2	BnaC09g06910.1D2
R53	BnaA09g08910.1D2	BnaC09g06060.1D2
R53	BnaA09g08940.1D2	BnaC09g06090.1D2
R53	BnaA09g08950.1D2	BnaC09g06120.1D2
R53	BnaA09g08970.1D2	BnaC09g06140.1D2
R53	BnaA09g08990.1D2	BnaC09g06200.1D2
R53	BnaA09g16050.1D2	BnaC09g16030.1D2
R53	BnaA09g16050.1D2	BnaC09g16060.1D2

List of HE genes (C01 to A01)

**Accession**	**Lost gene**	**Duplicated HE gene**
Chuosenshu	BnaC01g00010.1D2	BnaA01g05210.1D2
Chuosenshu	BnaC01g00070.1D2	BnaA01g05240.1D2
Chuosenshu	BnaC01g00080.1D2	BnaA01g05250.1D2
Chuosenshu	BnaC01g00160.1D2	BnaA01g05300.1D2
Chuosenshu	BnaC01g00220.1D2	BnaA01g05340.1D2
Chuosenshu	BnaC01g00250.1D2	BnaA01g05370.1D2
Chuosenshu	BnaC01g00280.1D2	BnaA01g05410.1D2
Chuosenshu	BnaC01g00330.1D2	BnaA01g05480.1D2
Chuosenshu	BnaC01g00370.1D2	BnaA01g05530.1D2
Chuosenshu	BnaC01g00540.1D2	BnaA01g05610.1D2
Chuosenshu	BnaC01g00560.1D2	BnaA01g05600.1D2
Chuosenshu	BnaC01g00700.1D2	BnaA01g00040.1D2
Chuosenshu	BnaC01g00730.1D2	BnaA01g00060.1D2
Chuosenshu	BnaC01g00750.1D2	BnaA01g00080.1D2
Chuosenshu	BnaC01g00800.1D2	BnaA01g00130.1D2

Chuosenshu	BnaC01g00810.1D2	BnaA01g00140.1D2
Chuosenshu	BnaC01g00870.1D2	BnaA01g00190.1D2
Chuosenshu	BnaC01g00890.1D2	BnaA01g00210.1D2
Chuosenshu	BnaC01g00960.1D2	BnaA01g00260.1D2
Chuosenshu	BnaC01g00990.1D2	BnaA01g00290.1D2
Chuosenshu	BnaC01g01030.1D2	BnaA01g00330.1D2
Chuosenshu	BnaC01g01070.1D2	BnaA01g00360.1D2
Chuosenshu	BnaC01g01210.1D2	BnaA01g00560.1D2
Chuosenshu	BnaC01g01220.1D2	BnaA01g00530.1D2
Chuosenshu	BnaC01g01270.1D2	BnaA01g00590.1D2
Chuosenshu	BnaC01g01290.1D2	BnaA01g00610.1D2
Chuosenshu	BnaC01g01340.1D2	BnaA01g00660.1D2
Chuosenshu	BnaC01g01440.1D2	BnaA01g00740.1D2
Chuosenshu	BnaC01g01460.1D2	BnaA01g00760.1D2
Chuosenshu	BnaC01g01490.1D2	BnaA01g00800.1D2
Chuosenshu	BnaC01g01540.1D2	BnaA01g00840.1D2
Chuosenshu	BnaC01g01550.1D2	BnaA01g00850.1D2
Chuosenshu	BnaC01g01560.1D2	BnaA01g04930.1D2
Chuosenshu	BnaC01g01610.1D2	BnaA01g00900.1D2
Chuosenshu	BnaC01g01630.1D2	BnaA01g00920.1D2
Chuosenshu	BnaC01g01650.1D2	BnaA01g00950.1D2
Chuosenshu	BnaC01g01660.1D2	BnaA01g00960.1D2
Chuosenshu	BnaC01g01760.1D2	BnaA01g01040.1D2
Chuosenshu	BnaC01g01880.1D2	BnaA01g01170.1D2
Chuosenshu	BnaC01g01890.1D2	BnaA01g01180.1D2
Chuosenshu	BnaC01g01920.1D2	BnaA01g01230.1D2
Chuosenshu	BnaC01g01990.1D2	BnaA01g01300.1D2
Chuosenshu	BnaC01g02230.1D2	BnaA01g01520.1D2
Chuosenshu	BnaC01g02250.1D2	BnaA01g01540.1D2
Chuosenshu	BnaC01g02300.1D2	BnaA01g01590.1D2
Chuosenshu	BnaC01g02310.1D2	BnaA01g01600.1D2
Chuosenshu	BnaC01g02390.1D2	BnaA01g01670.1D2
Chuosenshu	BnaC01g02420.1D2	BnaA01g01710.1D2
Chuosenshu	BnaC01g02470.1D2	BnaA01g01760.1D2
Chuosenshu	BnaC01g02490.1D2	BnaA01g01780.1D2
Chuosenshu	BnaC01g02540.1D2	BnaA01g01830.1D2
Chuosenshu	BnaC01g02570.1D2	BnaA01g01860.1D2
Chuosenshu	BnaC01g02590.1D2	BnaA01g01880.1D2
Chuosenshu	BnaC01g02610.1D2	BnaA01g01920.1D2
Chuosenshu	BnaC01g02640.1D2	BnaA01g01950.1D2
Chuosenshu	BnaC01g02670.1D2	BnaA01g01980.1D2
Chuosenshu	BnaC01g02710.1D2	BnaA01g02030.1D2
Chuosenshu	BnaC01g02820.1D2	BnaA01g05170.1D2
Chuosenshu	BnaC01g02870.1D2	BnaA01g05200.1D2
Chuosenshu	BnaC01g02910.1D2	BnaA01g02130.1D2
Chuosenshu	BnaC01g02920.1D2	BnaA01g02140.1D2
Chuosenshu	BnaC01g02980.1D2	BnaA01g02190.1D2
Chuosenshu	BnaC01g02990.1D2	BnaA01g02200.1D2
Chuosenshu	BnaC01g03000.1D2	BnaA01g02210.1D2
Chuosenshu	BnaC01g03070.1D2	BnaA01g02280.1D2
Chuosenshu	BnaC01g03080.1D2	BnaA01g02290.1D2

Chuosenshu	BnaC01g03100.1D2	BnaA01g02310.1D2
Chuosenshu	BnaC01g03110.1D2	BnaA01g02320.1D2
Chuosenshu	BnaC01g03130.1D2	BnaA01g02340.1D2
Chuosenshu	BnaC01g03150.1D2	BnaA01g02360.1D2
Chuosenshu	BnaC01g03170.1D2	BnaA01g02380.1D2
Chuosenshu	BnaC01g03200.1D2	BnaA01g02410.1D2
Chuosenshu	BnaC01g03260.1D2	BnaA01g02470.1D2
Chuosenshu	BnaC01g03270.1D2	BnaA01g02480.1D2
Chuosenshu	BnaC01g03300.1D2	BnaA01g02500.1D2
Chuosenshu	BnaC01g03360.1D2	BnaA01g02540.1D2
Chuosenshu	BnaC01g03420.1D2	BnaA01g02580.1D2
Chuosenshu	BnaC01g03480.1D2	BnaA01g02630.1D2
Chuosenshu	BnaC01g03490.1D2	BnaA01g02630.1D2
Chuosenshu	BnaC01g03500.1D2	BnaA01g02640.1D2
Chuosenshu	BnaC01g03510.1D2	BnaA01g02650.1D2
Chuosenshu	BnaC01g03520.1D2	BnaA01g02650.1D2
Chuosenshu	BnaC01g03550.1D2	BnaA01g02700.1D2
Chuosenshu	BnaC01g03560.1D2	BnaA01g02710.1D2
Chuosenshu	BnaC01g03600.1D2	BnaA01g02760.1D2
Chuosenshu	BnaC01g03630.1D2	BnaA01g02770.1D2
Chuosenshu	BnaC01g03640.1D2	BnaA01g02770.1D2
Chuosenshu	BnaC01g03670.1D2	BnaA01g02810.1D2
Chuosenshu	BnaC01g03710.1D2	BnaA01g02850.1D2
Chuosenshu	BnaC01g03720.1D2	BnaA01g02860.1D2
Chuosenshu	BnaC01g03730.1D2	BnaA01g02870.1D2
Chuosenshu	BnaC01g03740.1D2	BnaA01g02880.1D2
Chuosenshu	BnaC01g03770.1D2	BnaA01g02900.1D2
Chuosenshu	BnaC01g03820.1D2	BnaA01g02930.1D2
Chuosenshu	BnaC01g03850.1D2	BnaA01g02960.1D2
Chuosenshu	BnaC01g04000.1D2	BnaA01g03080.1D2
Chuosenshu	BnaC01g04130.1D2	BnaA01g03190.1D2
Chuosenshu	BnaC01g04160.1D2	BnaA01g03220.1D2
Chuosenshu	BnaC01g04190.1D2	BnaA01g03250.1D2
Chuosenshu	BnaC01g04200.1D2	BnaA01g03260.1D2
Chuosenshu	BnaC01g04210.1D2	BnaA01g03270.1D2
Chuosenshu	BnaC01g04230.1D2	BnaA01g03290.1D2
Chuosenshu	BnaC01g04250.1D2	BnaA01g03310.1D2
Chuosenshu	BnaC01g04270.1D2	BnaA01g03330.1D2
Chuosenshu	BnaC01g04340.1D2	BnaA01g03390.1D2
Chuosenshu	BnaC01g04370.1D2	BnaA01g03410.1D2
Chuosenshu	BnaC01g04380.1D2	BnaA01g03420.1D2
Chuosenshu	BnaC01g04460.1D2	BnaA01g03500.1D2
Chuosenshu	BnaC01g04490.1D2	BnaA01g03530.1D2
Chuosenshu	BnaC01g04530.1D2	BnaA01g03550.1D2
Chuosenshu	BnaC01g04540.1D2	BnaA01g03560.1D2
Chuosenshu	BnaC01g04570.1D2	BnaA01g03580.1D2
Chuosenshu	BnaC01g04580.1D2	BnaA01g03590.1D2
Chuosenshu	BnaC01g04590.1D2	BnaA01g03600.1D2
Chuosenshu	BnaC01g04610.1D2	BnaA01g03630.1D2
Chuosenshu	BnaC01g04660.1D2	BnaA01g03730.1D2
Chuosenshu	BnaC01g04750.1D2	BnaA01g03790.1D2

Chuosenshu	BnaC01g04800.1D2	BnaA01g03790.1D2
Chuosenshu	BnaC01g04870.1D2	BnaA01g03850.1D2
Chuosenshu	BnaC01g04890.1D2	BnaA01g03880.1D2
Chuosenshu	BnaC01g05010.1D2	BnaA01g03980.1D2
Chuosenshu	BnaC01g05070.1D2	BnaA01g04060.1D2
Chuosenshu	BnaC01g05140.1D2	BnaA01g04130.1D2
Chuosenshu	BnaC01g05150.1D2	BnaA01g04130.1D2
Chuosenshu	BnaC01g05190.1D2	BnaA01g04160.1D2
Chuosenshu	BnaC01g05210.1D2	BnaA01g04180.1D2
Chuosenshu	BnaC01g05220.1D2	BnaA01g04190.1D2
Chuosenshu	BnaC01g05240.1D2	BnaA01g04210.1D2
Chuosenshu	BnaC01g05280.1D2	BnaA01g04250.1D2
Chuosenshu	BnaC01g05300.1D2	BnaA01g04270.1D2
Chuosenshu	BnaC01g05310.1D2	BnaA01g04280.1D2
Chuosenshu	BnaC01g05320.1D2	BnaA01g04280.1D2
Chuosenshu	BnaC01g05410.1D2	BnaA01g04340.1D2
Chuosenshu	BnaC01g05450.1D2	BnaA01g04380.1D2
Chuosenshu	BnaC01g05470.1D2	BnaA01g04400.1D2
Chuosenshu	BnaC01g05510.1D2	BnaA01g04440.1D2
Chuosenshu	BnaC01g05530.1D2	BnaA01g04460.1D2
Chuosenshu	BnaC01g05560.1D2	BnaA01g04490.1D2
Chuosenshu	BnaC01g05600.1D2	BnaA01g04530.1D2
Chuosenshu	BnaC01g05730.1D2	BnaA01g04630.1D2
Chuosenshu	BnaC01g05740.1D2	BnaA01g04640.1D2
Chuosenshu	BnaC01g05810.1D2	BnaA01g04700.1D2
Chuosenshu	BnaC01g05840.1D2	BnaA01g04730.1D2
Chuosenshu	BnaC01g05860.1D2	BnaA01g04760.1D2
Chuosenshu	BnaC01g06010.1D2	BnaA01g04810.1D2
Chuosenshu	BnaC01g06070.1D2	BnaA01g05780.1D2
Chuosenshu	BnaC01g06100.1D2	BnaA01g05820.1D2
Chuosenshu	BnaC01g06140.1D2	BnaA01g05870.1D2
Chuosenshu	BnaC01g06160.1D2	BnaA01g05890.1D2
Chuosenshu	BnaC01g06180.1D2	BnaA01g05900.1D2
Chuosenshu	BnaC01g06220.1D2	BnaA01g05930.1D2
Chuosenshu	BnaC01g06260.1D2	BnaA01g05970.1D2
Chuosenshu	BnaC01g06270.1D2	BnaA01g05980.1D2
Chuosenshu	BnaC01g06280.1D2	BnaA01g05990.1D2
Chuosenshu	BnaC01g06370.1D2	BnaA01g06060.1D2
Chuosenshu	BnaC01g06380.1D2	BnaA01g06060.1D2
Chuosenshu	BnaC01g06390.1D2	BnaA01g06070.1D2
Chuosenshu	BnaC01g06490.1D2	BnaA01g06180.1D2
Chuosenshu	BnaC01g06630.1D2	BnaA01g06400.1D2
Chuosenshu	BnaC01g06640.1D2	BnaA01g06410.1D2
Chuosenshu	BnaC01g06650.1D2	BnaA01g06420.1D2
Chuosenshu	BnaC01g06880.1D2	BnaA01g06550.1D2
Chuosenshu	BnaC01g06970.1D2	BnaA01g06610.1D2
Chuosenshu	BnaC01g07010.1D2	BnaA01g06640.1D2
Chuosenshu	BnaC01g07100.1D2	BnaA01g06740.1D2
Chuosenshu	BnaC01g07570.1D2	BnaA01g03470.1D2
Chuosenshu	BnaC01g07590.1D2	BnaA01g05950.1D2
Chuosenshu	BnaC01g07610.1D2	BnaA01g02780.1D2

Chuosenshu	BnaC01g07710.1D2	BnaA01g05070.1D2
Chuosenshu	BnaC01g32640.1D2	BnaA01g28570.1D2
Chuosenshu	BnaC01g33270.1D2	BnaA01g28660.1D2
GSchnittkohl	BnaC01g00070.1D2	BnaA01g05240.1D2
GSchnittkohl	BnaC01g00120.1D2	BnaA01g05270.1D2
GSchnittkohl	BnaC01g00160.1D2	BnaA01g05300.1D2
GSchnittkohl	BnaC01g00260.1D2	BnaA01g05380.1D2
GSchnittkohl	BnaC01g00280.1D2	BnaA01g05410.1D2
GSchnittkohl	BnaC01g00320.1D2	BnaA01g05480.1D2
GSchnittkohl	BnaC01g00330.1D2	BnaA01g05480.1D2
GSchnittkohl	BnaC01g00340.1D2	BnaA01g05490.1D2
GSchnittkohl	BnaC01g00370.1D2	BnaA01g05530.1D2
GSchnittkohl	BnaC01g00570.1D2	BnaA01g05550.1D2
GSchnittkohl	BnaC01g00630.1D2	BnaA01g05670.1D2
GSchnittkohl	BnaC01g00700.1D2	BnaA01g00040.1D2
GSchnittkohl	BnaC01g00710.1D2	BnaA01g00050.1D2
GSchnittkohl	BnaC01g00730.1D2	BnaA01g00060.1D2
GSchnittkohl	BnaC01g00770.1D2	BnaA01g00100.1D2
GSchnittkohl	BnaC01g00800.1D2	BnaA01g00130.1D2
GSchnittkohl	BnaC01g00810.1D2	BnaA01g00140.1D2
GSchnittkohl	BnaC01g00850.1D2	BnaA01g00170.1D2
GSchnittkohl	BnaC01g00860.1D2	BnaA01g00180.1D2
GSchnittkohl	BnaC01g00870.1D2	BnaA01g00190.1D2
GSchnittkohl	BnaC01g00890.1D2	BnaA01g00210.1D2
GSchnittkohl	BnaC01g00960.1D2	BnaA01g00260.1D2
GSchnittkohl	BnaC01g00980.1D2	BnaA01g00280.1D2
GSchnittkohl	BnaC01g01030.1D2	BnaA01g00330.1D2
GSchnittkohl	BnaC01g01070.1D2	BnaA01g00360.1D2
GSchnittkohl	BnaC01g01120.1D2	BnaA01g00410.1D2
GSchnittkohl	BnaC01g01210.1D2	BnaA01g00560.1D2
GSchnittkohl	BnaC01g01220.1D2	BnaA01g00530.1D2
GSchnittkohl	BnaC01g01270.1D2	BnaA01g00590.1D2
GSchnittkohl	BnaC01g01350.1D2	BnaA01g00670.1D2
GSchnittkohl	BnaC01g01400.1D2	BnaA01g00710.1D2
GSchnittkohl	BnaC01g01440.1D2	BnaA01g00740.1D2
GSchnittkohl	BnaC01g01460.1D2	BnaA01g00760.1D2
GSchnittkohl	BnaC01g01480.1D2	BnaA01g00790.1D2
GSchnittkohl	BnaC01g01490.1D2	BnaA01g00800.1D2
GSchnittkohl	BnaC01g01530.1D2	BnaA01g00830.1D2
GSchnittkohl	BnaC01g01540.1D2	BnaA01g00840.1D2
GSchnittkohl	BnaC01g01550.1D2	BnaA01g00850.1D2
GSchnittkohl	BnaC01g01560.1D2	BnaA01g04930.1D2
GSchnittkohl	BnaC01g01600.1D2	BnaA01g00890.1D2
GSchnittkohl	BnaC01g01610.1D2	BnaA01g00900.1D2
GSchnittkohl	BnaC01g01630.1D2	BnaA01g00920.1D2
GSchnittkohl	BnaC01g01650.1D2	BnaA01g00950.1D2
GSchnittkohl	BnaC01g01690.1D2	BnaA01g00990.1D2
GSchnittkohl	BnaC01g01700.1D2	BnaA01g01000.1D2
GSchnittkohl	BnaC01g01880.1D2	BnaA01g01160.1D2
GSchnittkohl	BnaC01g01880.1D2	BnaA01g01170.1D2
GSchnittkohl	BnaC01g01890.1D2	BnaA01g01180.1D2

GSchnittkohl	BnaC01g01920.1D2	BnaA01g01230.1D2
GSchnittkohl	BnaC01g01930.1D2	BnaA01g01240.1D2
GSchnittkohl	BnaC01g01940.1D2	BnaA01g01250.1D2
GSchnittkohl	BnaC01g01990.1D2	BnaA01g01300.1D2
GSchnittkohl	BnaC01g02000.1D2	BnaA01g01310.1D2
GSchnittkohl	BnaC01g02130.1D2	BnaA01g01420.1D2
GSchnittkohl	BnaC01g02210.1D2	BnaA01g01500.1D2
GSchnittkohl	BnaC01g02230.1D2	BnaA01g01520.1D2
GSchnittkohl	BnaC01g02250.1D2	BnaA01g01540.1D2
GSchnittkohl	BnaC01g02300.1D2	BnaA01g01590.1D2
GSchnittkohl	BnaC01g02330.1D2	BnaA01g01620.1D2
GSchnittkohl	BnaC01g02390.1D2	BnaA01g01670.1D2
GSchnittkohl	BnaC01g02470.1D2	BnaA01g01760.1D2
GSchnittkohl	BnaC01g02490.1D2	BnaA01g01780.1D2
GSchnittkohl	BnaC01g02520.1D2	BnaA01g01810.1D2
GSchnittkohl	BnaC01g02540.1D2	BnaA01g01830.1D2
GSchnittkohl	BnaC01g02560.1D2	BnaA01g01850.1D2
GSchnittkohl	BnaC01g02570.1D2	BnaA01g01860.1D2
GSchnittkohl	BnaC01g02610.1D2	BnaA01g01920.1D2
GSchnittkohl	BnaC01g02640.1D2	BnaA01g01950.1D2
GSchnittkohl	BnaC01g02830.1D2	BnaA01g05180.1D2
GSchnittkohl	BnaC01g02870.1D2	BnaA01g05200.1D2
GSchnittkohl	BnaC01g02960.1D2	BnaA01g02170.1D2
GSchnittkohl	BnaC01g03070.1D2	BnaA01g02280.1D2
GSchnittkohl	BnaC01g03230.1D2	BnaA01g02440.1D2
GSchnittkohl	BnaC01g03260.1D2	BnaA01g02470.1D2
GSchnittkohl	BnaC01g03270.1D2	BnaA01g02480.1D2
GSchnittkohl	BnaC01g03480.1D2	BnaA01g02630.1D2
GSchnittkohl	BnaC01g03490.1D2	BnaA01g02630.1D2
GSchnittkohl	BnaC01g03600.1D2	BnaA01g02760.1D2
GSchnittkohl	BnaC01g03730.1D2	BnaA01g02870.1D2
GSchnittkohl	BnaC01g03740.1D2	BnaA01g02880.1D2
GSchnittkohl	BnaC01g03820.1D2	BnaA01g02930.1D2
GSchnittkohl	BnaC01g04000.1D2	BnaA01g03080.1D2
GSchnittkohl	BnaC01g04050.1D2	BnaA01g03130.1D2
GSchnittkohl	BnaC01g04160.1D2	BnaA01g03220.1D2
GSchnittkohl	BnaC01g04210.1D2	BnaA01g03270.1D2
GSchnittkohl	BnaC01g04220.1D2	BnaA01g03280.1D2
GSchnittkohl	BnaC01g04320.1D2	BnaA01g03380.1D2
GSchnittkohl	BnaC01g04440.1D2	BnaA01g03480.1D2
GSchnittkohl	BnaC01g04460.1D2	BnaA01g03500.1D2
GSchnittkohl	BnaC01g04580.1D2	BnaA01g03590.1D2
GSchnittkohl	BnaC01g04590.1D2	BnaA01g03600.1D2
GSchnittkohl	BnaC01g04700.1D2	BnaA01g03780.1D2
GSchnittkohl	BnaC01g04830.1D2	BnaA01g03810.1D2
GSchnittkohl	BnaC01g04840.1D2	BnaA01g03820.1D2
GSchnittkohl	BnaC01g04980.1D2	BnaA01g04920.1D2
GSchnittkohl	BnaC01g05010.1D2	BnaA01g03980.1D2
GSchnittkohl	BnaC01g05040.1D2	BnaA01g04030.1D2
GSchnittkohl	BnaC01g05070.1D2	BnaA01g04060.1D2
GSchnittkohl	BnaC01g05100.1D2	BnaA01g04100.1D2

GSchnittkohl	BnaC01g05150.1D2	BnaA01g04130.1D2
GSchnittkohl	BnaC01g05210.1D2	BnaA01g04180.1D2
GSchnittkohl	BnaC01g05300.1D2	BnaA01g04270.1D2
GSchnittkohl	BnaC01g05310.1D2	BnaA01g04280.1D2
GSchnittkohl	BnaC01g05320.1D2	BnaA01g04280.1D2
GSchnittkohl	BnaC01g05340.1D2	BnaA01g04300.1D2
GSchnittkohl	BnaC01g05360.1D2	BnaA01g04310.1D2
GSchnittkohl	BnaC01g05410.1D2	BnaA01g04340.1D2
GSchnittkohl	BnaC01g05470.1D2	BnaA01g04400.1D2
GSchnittkohl	BnaC01g05480.1D2	BnaA01g04410.1D2
GSchnittkohl	BnaC01g05500.1D2	BnaA01g04430.1D2
GSchnittkohl	BnaC01g05600.1D2	BnaA01g04530.1D2
GSchnittkohl	BnaC01g05690.1D2	BnaA01g04600.1D2
GSchnittkohl	BnaC01g05740.1D2	BnaA01g04640.1D2
GSchnittkohl	BnaC01g05790.1D2	BnaA01g04680.1D2
GSchnittkohl	BnaC01g05810.1D2	BnaA01g04700.1D2
GSchnittkohl	BnaC01g05840.1D2	BnaA01g04730.1D2
GSchnittkohl	BnaC01g05970.1D2	BnaA01g04850.1D2
GSchnittkohl	BnaC01g07550.1D2	BnaA01g05690.1D2
GSchnittkohl	BnaC01g07570.1D2	BnaA01g03470.1D2
GSchnittkohl	BnaC01g07640.1D2	BnaA01g04060.1D2
GSchnittkohl	BnaC01g07650.1D2	BnaA01g04070.1D2
GSchnittkohl	BnaC01g07670.1D2	BnaA01g04070.1D2
GSchnittkohl	BnaC01g07710.1D2	BnaA01g05070.1D2
GSchnittkohl	BnaC01g14850.1D2	BnaA01g13290.1D2
GSchnittkohl	BnaC01g14870.1D2	BnaA01g13310.1D2
GSchnittkohl	BnaC01g14900.1D2	BnaA01g13320.1D2
GSchnittkohl	BnaC01g14920.1D2	BnaA01g13340.1D2
GSchnittkohl	BnaC01g14950.1D2	BnaA01g13360.1D2
GSchnittkohl	BnaC01g14970.1D2	BnaA01g13380.1D2
GSchnittkohl	BnaC01g14990.1D2	BnaA01g13400.1D2
GSchnittkohl	BnaC01g15000.1D2	BnaA01g13410.1D2
GSchnittkohl	BnaC01g15580.1D2	BnaA01g13270.1D2
GSchnittkohl	BnaC01g29960.1D2	BnaA01g25990.1D2
GSchnittkohl	BnaC01g29970.1D2	BnaA01g29010.1D2
GSchnittkohl	BnaC01g30040.1D2	BnaA01g26070.1D2
GSchnittkohl	BnaC01g30120.1D2	BnaA01g26120.1D2
GSchnittkohl	BnaC01g30250.1D2	BnaA01g26250.1D2
GSchnittkohl	BnaC01g30270.1D2	BnaA01g26260.1D2
GSchnittkohl	BnaC01g30380.1D2	BnaA01g26370.1D2
GSchnittkohl	BnaC01g30390.1D2	BnaA01g26380.1D2
GSchnittkohl	BnaC01g30410.1D2	BnaA01g26400.1D2
GSchnittkohl	BnaC01g30480.1D2	BnaA01g26540.1D2
GSchnittkohl	BnaC01g30490.1D2	BnaA01g26550.1D2
GSchnittkohl	BnaC01g30570.1D2	BnaA01g26630.1D2
GSchnittkohl	BnaC01g30580.1D2	BnaA01g26640.1D2
GSchnittkohl	BnaC01g30670.1D2	BnaA01g26700.1D2
GSchnittkohl	BnaC01g30780.1D2	BnaA01g26790.1D2
GSchnittkohl	BnaC01g30840.1D2	BnaA01g26930.1D2
GSchnittkohl	BnaC01g30860.1D2	BnaA01g26950.1D2
GSchnittkohl	BnaC01g31310.1D2	BnaA01g27120.1D2

GSchnittkohl	BnaC01g31430.1D2	BnaA01g27570.1D2
GSchnittkohl	BnaC01g31570.1D2	BnaA01g27310.1D2
GSchnittkohl	BnaC01g31600.1D2	BnaA01g27350.1D2
GSchnittkohl	BnaC01g31620.1D2	BnaA01g27350.1D2
GSchnittkohl	BnaC01g31640.1D2	BnaA01g27370.1D2
GSchnittkohl	BnaC01g31680.1D2	BnaA01g27420.1D2
GSchnittkohl	BnaC01g31780.1D2	BnaA01g27760.1D2
GSchnittkohl	BnaC01g31920.1D2	BnaA01g27900.1D2
GSchnittkohl	BnaC01g31930.1D2	BnaA01g27910.1D2
GSchnittkohl	BnaC01g31970.1D2	BnaA01g27930.1D2
GSchnittkohl	BnaC01g32010.1D2	BnaA01g27660.1D2
GSchnittkohl	BnaC01g32160.1D2	BnaA01g27210.1D2
GSchnittkohl	BnaC01g32280.1D2	BnaA01g28060.1D2
GSchnittkohl	BnaC01g32300.1D2	BnaA01g28090.1D2
GSchnittkohl	BnaC01g32410.1D2	BnaA01g28200.1D2
GSchnittkohl	BnaC01g32420.1D2	BnaA01g28210.1D2
GSchnittkohl	BnaC01g32490.1D2	BnaA01g28270.1D2
GSchnittkohl	BnaC01g32500.1D2	BnaA01g28280.1D2
GSchnittkohl	BnaC01g32530.1D2	BnaA01g28900.1D2
GSchnittkohl	BnaC01g32710.1D2	BnaA01g28490.1D2
GSchnittkohl	BnaC01g32870.1D2	BnaA01g27980.1D2
GSchnittkohl	BnaC01g32880.1D2	BnaA01g27990.1D2
GSchnittkohl	BnaC01g32890.1D2	BnaA01g28000.1D2
GSchnittkohl	BnaC01g32940.1D2	BnaA01g26430.1D2
GSchnittkohl	BnaC01g32980.1D2	BnaA01g28370.1D2
GSchnittkohl	BnaC01g33010.1D2	BnaA01g28990.1D2
GSchnittkohl	BnaC01g33240.1D2	BnaA01g28640.1D2
GSchnittkohl	BnaC01g33270.1D2	BnaA01g28660.1D2
GSchnittkohl	BnaC01g33310.1D2	BnaA01g28700.1D2
GSchnittkohl	BnaC01g33380.1D2	BnaA01g28750.1D2
GSchnittkohl	BnaC01g33590.1D2	BnaA01g27500.1D2
GSchnittkohl	BnaC01g33660.1D2	BnaA01g30720.1D2
GSchnittkohl	BnaC01g33680.1D2	BnaA01g30750.1D2
GSchnittkohl	BnaC01g33720.1D2	BnaA01g30780.1D2
GSchnittkohl	BnaC01g33720.1D2	BnaA01g31090.1D2
GSchnittkohl	BnaC01g33730.1D2	BnaA01g30780.1D2
GSchnittkohl	BnaC01g33730.1D2	BnaA01g31080.1D2
GSchnittkohl	BnaC01g33780.1D2	BnaA01g31050.1D2
GSchnittkohl	BnaC01g33900.1D2	BnaA01g30940.1D2
GSchnittkohl	BnaC01g33940.1D2	BnaA01g30970.1D2
GSchnittkohl	BnaC01g33950.1D2	BnaA01g30980.1D2
GSchnittkohl	BnaC01g33970.1D2	BnaA01g30990.1D2
GSchnittkohl	BnaC01g34040.1D2	BnaA01g32620.1D2
GSchnittkohl	BnaC01g34070.1D2	BnaA01g32660.1D2
GSchnittkohl	BnaC01g34160.1D2	BnaA01g31000.1D2
GSchnittkohl	BnaC01g34180.1D2	BnaA01g31010.1D2
GSchnittkohl	BnaC01g34230.1D2	BnaA01g31760.1D2
GSchnittkohl	BnaC01g34240.1D2	BnaA01g31740.1D2
GSchnittkohl	BnaC01g34270.1D2	BnaA01g31710.1D2
GSchnittkohl	BnaC01g34300.1D2	BnaA01g31650.1D2
GSchnittkohl	BnaC01g34350.1D2	BnaA01g31670.1D2

GSchnittkohl	BnaC01g34510.1D2	BnaA01g29210.1D2
GSchnittkohl	BnaC01g34520.1D2	BnaA01g29220.1D2
GSchnittkohl	BnaC01g34550.1D2	BnaA01g31130.1D2
GSchnittkohl	BnaC01g34560.1D2	BnaA01g31140.1D2
GSchnittkohl	BnaC01g34620.1D2	BnaA01g31170.1D2
GSchnittkohl	BnaC01g34770.1D2	BnaA01g31300.1D2
GSchnittkohl	BnaC01g34850.1D2	BnaA01g31340.1D2
GSchnittkohl	BnaC01g34860.1D2	BnaA01g31340.1D2
GSchnittkohl	BnaC01g34870.1D2	BnaA01g31350.1D2
GSchnittkohl	BnaC01g34880.1D2	BnaA01g31360.1D2
GSchnittkohl	BnaC01g35010.1D2	BnaA01g31480.1D2
GSchnittkohl	BnaC01g35030.1D2	BnaA01g31490.1D2
GSchnittkohl	BnaC01g35060.1D2	BnaA01g31550.1D2
GSchnittkohl	BnaC01g35070.1D2	BnaA01g31550.1D2
GSchnittkohl	BnaC01g35100.1D2	BnaA01g31580.1D2
GSchnittkohl	BnaC01g35110.1D2	BnaA01g31590.1D2
GSchnittkohl	BnaC01g35200.1D2	BnaA01g32600.1D2
GSchnittkohl	BnaC01g35360.1D2	BnaA01g32290.1D2
GSchnittkohl	BnaC01g35380.1D2	BnaA01g32260.1D2
GSchnittkohl	BnaC01g35470.1D2	BnaA01g33590.1D2
GSchnittkohl	BnaC01g35520.1D2	BnaA01g33670.1D2
GSchnittkohl	BnaC01g35530.1D2	BnaA01g33680.1D2
GSchnittkohl	BnaC01g35550.1D2	BnaA01g33710.1D2
GSchnittkohl	BnaC01g35590.1D2	BnaA01g33760.1D2
GSchnittkohl	BnaC01g35640.1D2	BnaA01g33780.1D2
GSchnittkohl	BnaC01g35680.1D2	BnaA01g33810.1D2
GSchnittkohl	BnaC01g35700.1D2	BnaA01g33830.1D2
GSchnittkohl	BnaC01g35710.1D2	BnaA01g33840.1D2
GSchnittkohl	BnaC01g35730.1D2	BnaA01g33860.1D2
GSchnittkohl	BnaC01g36800.1D2	BnaA01g35230.1D2
GSchnittkohl	BnaC01g36840.1D2	BnaA01g35260.1D2
GSchnittkohl	BnaC01g36980.1D2	BnaA01g35370.1D2
GSchnittkohl	BnaC01g37390.1D2	BnaA01g35770.1D2
Sensation	BnaC01g00250.1D2	BnaA01g05370.1D2
Sensation	BnaC01g06750.1D2	BnaA01g31260.1D2
Sensation	BnaC01g31290.1D2	BnaA01g27100.1D2
Sensation	BnaC01g31300.1D2	BnaA01g27110.1D2
Sensation	BnaC01g31310.1D2	BnaA01g27120.1D2
Sensation	BnaC01g31330.1D2	BnaA01g27140.1D2
Sensation	BnaC01g31340.1D2	BnaA01g27180.1D2
Sensation	BnaC01g31430.1D2	BnaA01g27570.1D2
Sensation	BnaC01g31480.1D2	BnaA01g27610.1D2
Sensation	BnaC01g31780.1D2	BnaA01g27760.1D2
Sensation	BnaC01g32010.1D2	BnaA01g27660.1D2
Sensation	BnaC01g32110.1D2	BnaA01g27260.1D2
Sensation	BnaC01g32140.1D2	BnaA01g27230.1D2
Sensation	BnaC01g32500.1D2	BnaA01g28280.1D2
Sensation	BnaC01g32640.1D2	BnaA01g28570.1D2
Sensation	BnaC01g32870.1D2	BnaA01g27980.1D2
Sensation	BnaC01g32980.1D2	BnaA01g28370.1D2
Sensation	BnaC01g33010.1D2	BnaA01g28990.1D2

Sensation	BnaC01g33380.1D2	BnaA01g28750.1D2
Sensation	BnaC01g33430.1D2	BnaA01g28790.1D2
Sensation	BnaC01g33630.1D2	BnaA01g30690.1D2
Sensation	BnaC01g33690.1D2	BnaA01g30760.1D2
Sensation	BnaC01g33720.1D2	BnaA01g31090.1D2
Sensation	BnaC01g33730.1D2	BnaA01g31080.1D2
Sensation	BnaC01g33850.1D2	BnaA01g30880.1D2
Sensation	BnaC01g33890.1D2	BnaA01g30930.1D2
Sensation	BnaC01g33900.1D2	BnaA01g30940.1D2
Sensation	BnaC01g33910.1D2	BnaA01g30950.1D2
Sensation	BnaC01g33940.1D2	BnaA01g30970.1D2
Sensation	BnaC01g33950.1D2	BnaA01g30980.1D2
Sensation	BnaC01g33970.1D2	BnaA01g30990.1D2
Sensation	BnaC01g34090.1D2	BnaA01g32680.1D2
Sensation	BnaC01g34110.1D2	BnaA01g32690.1D2
Sensation	BnaC01g34180.1D2	BnaA01g31010.1D2
Sensation	BnaC01g34200.1D2	BnaA01g31770.1D2
Sensation	BnaC01g34230.1D2	BnaA01g31760.1D2
Sensation	BnaC01g34270.1D2	BnaA01g31710.1D2
Sensation	BnaC01g34310.1D2	BnaA01g31700.1D2
Sensation	BnaC01g34320.1D2	BnaA01g31690.1D2
Sensation	BnaC01g34330.1D2	BnaA01g31680.1D2
Sensation	BnaC01g34350.1D2	BnaA01g31670.1D2
Sensation	BnaC01g34690.1D2	BnaA01g31230.1D2
Sensation	BnaC01g34700.1D2	BnaA01g31240.1D2
Sensation	BnaC01g34720.1D2	BnaA01g31260.1D2
Sensation	BnaC01g34750.1D2	BnaA01g31280.1D2
Sensation	BnaC01g34760.1D2	BnaA01g31290.1D2
Sensation	BnaC01g34770.1D2	BnaA01g31300.1D2
Sensation	BnaC01g34850.1D2	BnaA01g31340.1D2
Sensation	BnaC01g34860.1D2	BnaA01g31340.1D2
Sensation	BnaC01g34890.1D2	BnaA01g31380.1D2
Sensation	BnaC01g34910.1D2	BnaA01g31400.1D2
Sensation	BnaC01g34920.1D2	BnaA01g31400.1D2
Sensation	BnaC01g34960.1D2	BnaA01g31430.1D2
Sensation	BnaC01g34970.1D2	BnaA01g31440.1D2
Sensation	BnaC01g35010.1D2	BnaA01g31480.1D2
Sensation	BnaC01g35020.1D2	BnaA01g31490.1D2
Sensation	BnaC01g35030.1D2	BnaA01g31490.1D2
Sensation	BnaC01g35060.1D2	BnaA01g31550.1D2
Sensation	BnaC01g35100.1D2	BnaA01g31580.1D2
Sensation	BnaC01g35110.1D2	BnaA01g31590.1D2
Sensation	BnaC01g35370.1D2	BnaA01g32280.1D2
Sensation	BnaC01g35390.1D2	BnaA01g32250.1D2
Sensation	BnaC01g35550.1D2	BnaA01g33710.1D2
Sensation	BnaC01g35730.1D2	BnaA01g33860.1D2
Sensation	BnaC01g36530.1D2	BnaA01g29060.1D2
CRY_1	BnaC01g00070.1D2	BnaA01g05240.1D2
CRY_1	BnaC01g00080.1D2	BnaA01g05250.1D2
CRY_1	BnaC01g00090.1D2	BnaA01g05260.1D2
CRY_1	BnaC01g00120.1D2	BnaA01g05270.1D2

CRY_1	BnaC01g00320.1D2	BnaA01g05480.1D2
CRY_1	BnaC01g00810.1D2	BnaA01g00140.1D2
CRY_1	BnaC01g00920.1D2	BnaA01g04950.1D2
CRY_1	BnaC01g00960.1D2	BnaA01g00260.1D2
CRY_1	BnaC01g01180.1D2	BnaA01g00490.1D2
CRY_1	BnaC01g01220.1D2	BnaA01g00530.1D2
CRY_1	BnaC01g01230.1D2	BnaA01g00540.1D2
CRY_1	BnaC01g01550.1D2	BnaA01g00850.1D2
CRY_1	BnaC01g01760.1D2	BnaA01g01040.1D2
CRY_1	BnaC01g01940.1D2	BnaA01g01250.1D2
CRY_1	BnaC01g02250.1D2	BnaA01g19070.1D2
CRY_1	BnaC01g02710.1D2	BnaA01g02030.1D2
CRY_1	BnaC01g02870.1D2	BnaA01g05200.1D2
CRY_1	BnaC01g02970.1D2	BnaA01g02180.1D2
CRY_1	BnaC01g03090.1D2	BnaA01g02300.1D2
CRY_1	BnaC01g03100.1D2	BnaA01g02310.1D2
CRY_1	BnaC01g03310.1D2	BnaA01g02510.1D2
CRY_1	BnaC01g03820.1D2	BnaA01g02930.1D2
CRY_1	BnaC01g04190.1D2	BnaA01g03250.1D2
CRY_1	BnaC01g04220.1D2	BnaA01g03280.1D2
CRY_1	BnaC01g04440.1D2	BnaA01g03480.1D2
CRY_1	BnaC01g04490.1D2	BnaA01g03530.1D2
CRY_1	BnaC01g04720.1D2	BnaA01g03430.1D2
CRY_1	BnaC01g04750.1D2	BnaA01g03790.1D2
CRY_1	BnaC01g04830.1D2	BnaA01g03810.1D2
CRY_1	BnaC01g04840.1D2	BnaA01g03820.1D2
CRY_1	BnaC01g04930.1D2	BnaA01g03890.1D2
CRY_1	BnaC01g05360.1D2	BnaA01g04310.1D2
CRY_1	BnaC01g05840.1D2	BnaA01g04730.1D2
CRY_1	BnaC01g06220.1D2	BnaA01g05930.1D2
CRY_1	BnaC01g06370.1D2	BnaA01g06060.1D2
CRY_1	BnaC01g06380.1D2	BnaA01g06060.1D2
CRY_1	BnaC01g06650.1D2	BnaA01g06420.1D2
CRY_1	BnaC01g06880.1D2	BnaA01g06550.1D2
CRY_1	BnaC01g07650.1D2	BnaA01g04070.1D2
CRY_1	BnaC01g07710.1D2	BnaA01g05070.1D2
CRY_1	BnaC01g08010.1D2	BnaA01g07190.1D2
CRY_1	BnaC01g08690.1D2	BnaA01g07960.1D2
CRY_1	BnaC01g09070.1D2	BnaA01g11730.1D2
CRY_1	BnaC01g09080.1D2	BnaA01g11730.1D2
CRY_1	BnaC01g09670.1D2	BnaA01g08780.1D2
CRY_1	BnaC01g09870.1D2	BnaA01g09000.1D2
CRY_1	BnaC01g09990.1D2	BnaA01g02350.1D2
CRY_1	BnaC01g10400.1D2	BnaA01g09530.1D2
CRY_1	BnaC01g10440.1D2	BnaA01g09570.1D2
CRY_1	BnaC01g10830.1D2	BnaA01g08880.1D2
CRY_1	BnaC01g11070.1D2	BnaA01g09910.1D2
CRY_1	BnaC01g11090.1D2	BnaA01g09930.1D2
CRY_1	BnaC01g11330.1D2	BnaA01g10150.1D2
CRY_1	BnaC01g12080.1D2	BnaA01g10820.1D2
CRY_1	BnaC01g12710.1D2	BnaA01g11400.1D2

CRY_1	BnaC01g13740.1D2	BnaA01g12610.1D2
CRY_1	BnaC01g13980.1D2	BnaA01g12480.1D2
CRY_1	BnaC01g14190.1D2	BnaA01g12950.1D2
CRY_1	BnaC01g14580.1D2	BnaA01g13000.1D2
CRY_1	BnaC01g14620.1D2	BnaA01g13040.1D2
CRY_1	BnaC01g14700.1D2	BnaA01g13120.1D2
CRY_1	BnaC01g15040.1D2	BnaA01g13450.1D2
CRY_1	BnaC01g15140.1D2	BnaA01g13560.1D2
CRY_1	BnaC01g15340.1D2	BnaA01g13770.1D2
CRY_1	BnaC01g15380.1D2	BnaA01g13810.1D2
CRY_1	BnaC01g15730.1D2	BnaA01g12170.1D2
CRY_1	BnaC01g16210.1D2	BnaA01g14340.1D2
CRY_1	BnaC01g16320.1D2	BnaA01g14400.1D2
CRY_1	BnaC01g16370.1D2	BnaA01g14460.1D2
CRY_1	BnaC01g16640.1D2	BnaA01g14700.1D2
CRY_1	BnaC01g16910.1D2	BnaA01g14920.1D2
CRY_1	BnaC01g17190.1D2	BnaA01g15120.1D2
CRY_1	BnaC01g17250.1D2	BnaA01g14120.1D2
CRY_1	BnaC01g17290.1D2	BnaA01g14550.1D2
CRY_1	BnaC01g17630.1D2	BnaA01g15410.1D2
CRY_1	BnaC01g17650.1D2	BnaA01g15410.1D2
CRY_1	BnaC01g17650.1D2	BnaA01g15440.1D2
CRY_1	BnaC01g17660.1D2	BnaA01g15410.1D2
CRY_1	BnaC01g17660.1D2	BnaA01g15440.1D2
CRY_1	BnaC01g17810.1D2	BnaA01g15630.1D2
CRY_1	BnaC01g17910.1D2	BnaA01g15740.1D2
CRY_1	BnaC01g18080.1D2	BnaA01g24250.1D2
CRY_1	BnaC01g18290.1D2	BnaA01g15830.1D2
CRY_1	BnaC01g18330.1D2	BnaA01g15880.1D2
CRY_1	BnaC01g18930.1D2	BnaA01g16270.1D2
CRY_1	BnaC01g19240.1D2	BnaA01g17460.1D2
CRY_1	BnaC01g19890.1D2	BnaA01g17170.1D2
CRY_1	BnaC01g20160.1D2	BnaA01g17810.1D2
CRY_1	BnaC01g20340.1D2	BnaA01g17990.1D2
CRY_1	BnaC01g20480.1D2	BnaA01g24790.1D2
CRY_1	BnaC01g20610.1D2	BnaA01g18300.1D2
CRY_1	BnaC01g20740.1D2	BnaA01g18490.1D2
CRY_1	BnaC01g21030.1D2	BnaA01g18640.1D2
CRY_1	BnaC01g21240.1D2	BnaA01g19030.1D2
CRY_1	BnaC01g21250.1D2	BnaA01g19040.1D2
CRY_1	BnaC01g21280.1D2	BnaA01g19120.1D2
CRY_1	BnaC01g21550.1D2	BnaA01g21660.1D2
CRY_1	BnaC01g21560.1D2	BnaA01g21670.1D2
CRY_1	BnaC01g21620.1D2	BnaA01g22430.1D2
CRY_1	BnaC01g21800.1D2	BnaA01g18710.1D2
CRY_1	BnaC01g21820.1D2	BnaA01g20720.1D2
CRY_1	BnaC01g22200.1D2	BnaA01g21950.1D2
CRY_1	BnaC01g22360.1D2	BnaA01g19080.1D2
CRY_1	BnaC01g23810.1D2	BnaA01g20290.1D2
CRY_1	BnaC01g23840.1D2	BnaA01g21560.1D2
CRY_1	BnaC01g23850.1D2	BnaA01g21570.1D2

CRY_1	BnaC01g24130.1D2	BnaA01g20590.1D2
CRY_1	BnaC01g24140.1D2	BnaA01g21950.1D2
CRY_1	BnaC01g24830.1D2	BnaA01g24210.1D2
CRY_1	BnaC01g25110.1D2	BnaA01g21440.1D2
CRY_1	BnaC01g25850.1D2	BnaA01g21280.1D2
CRY_1	BnaC01g25950.1D2	BnaA01g20810.1D2
CRY_1	BnaC01g26050.1D2	BnaA01g21760.1D2
CRY_1	BnaC01g26450.1D2	BnaA01g23080.1D2
CRY_1	BnaC01g26490.1D2	BnaA01g23120.1D2
CRY_1	BnaC01g26840.1D2	BnaA01g22300.1D2
CRY_1	BnaC01g26920.1D2	BnaA01g22840.1D2
CRY_1	BnaC01g27480.1D2	BnaA01g23740.1D2
CRY_1	BnaC01g27710.1D2	BnaA01g23930.1D2
CRY_1	BnaC01g28000.1D2	BnaA01g23650.1D2
CRY_1	BnaC01g28380.1D2	BnaA01g24850.1D2
CRY_1	BnaC01g28470.1D2	BnaA01g24890.1D2
CRY_1	BnaC01g29050.1D2	BnaA01g25240.1D2
CRY_1	BnaC01g29260.1D2	BnaA01g21400.1D2
CRY_1	BnaC01g29320.1D2	BnaA01g24180.1D2
CRY_1	BnaC01g29780.1D2	BnaA01g25800.1D2
CRY_1	BnaC01g29970.1D2	BnaA01g29010.1D2
CRY_1	BnaC01g30690.1D2	BnaA01g26690.1D2
CRY_1	BnaC01g30690.1D2	BnaA01g26690.1D2
CRY_1	BnaC01g30690.1D2	BnaA01g26690.1D2
G50	BnaC01g00070.1D2	BnaA01g05240.1D2
G50	BnaC01g00080.1D2	BnaA01g05250.1D2
G50	BnaC01g00160.1D2	BnaA01g05300.1D2
G50	BnaC01g00260.1D2	BnaA01g05380.1D2
G50	BnaC01g00280.1D2	BnaA01g05410.1D2
G50	BnaC01g00330.1D2	BnaA01g05480.1D2
G50	BnaC01g00340.1D2	BnaA01g05490.1D2
G50	BnaC01g00440.1D2	BnaA01g05570.1D2
G50	BnaC01g00540.1D2	BnaA01g05610.1D2
G50	BnaC01g00560.1D2	BnaA01g05600.1D2
G50	BnaC01g00630.1D2	BnaA01g05670.1D2
G50	BnaC01g00690.1D2	BnaA01g00030.1D2
G50	BnaC01g00710.1D2	BnaA01g00050.1D2
G50	BnaC01g00750.1D2	BnaA01g00080.1D2
G50	BnaC01g00770.1D2	BnaA01g00100.1D2
G50	BnaC01g00800.1D2	BnaA01g00130.1D2
G50	BnaC01g00810.1D2	BnaA01g00140.1D2
G50	BnaC01g00860.1D2	BnaA01g00180.1D2
G50	BnaC01g00870.1D2	BnaA01g00190.1D2
G50	BnaC01g00890.1D2	BnaA01g00210.1D2
G50	BnaC01g00960.1D2	BnaA01g00260.1D2
G50	BnaC01g00980.1D2	BnaA01g00280.1D2
G50	BnaC01g00990.1D2	BnaA01g00290.1D2
G50	BnaC01g01030.1D2	BnaA01g00330.1D2
G50	BnaC01g01070.1D2	BnaA01g00360.1D2
G50	BnaC01g01080.1D2	BnaA01g00370.1D2
G50	BnaC01g01090.1D2	BnaA01g00380.1D2

G50	BnaC01g01120.1D2	BnaA01g00410.1D2
G50	BnaC01g01190.1D2	BnaA01g00500.1D2
G50	BnaC01g01200.1D2	BnaA01g00510.1D2
G50	BnaC01g01220.1D2	BnaA01g00530.1D2
G50	BnaC01g01240.1D2	BnaA01g00550.1D2
G50	BnaC01g01280.1D2	BnaA01g00600.1D2
G50	BnaC01g01290.1D2	BnaA01g00610.1D2
G50	BnaC01g01320.1D2	BnaA01g00640.1D2
G50	BnaC01g01350.1D2	BnaA01g00670.1D2
G50	BnaC01g01400.1D2	BnaA01g00710.1D2
G50	BnaC01g01410.1D2	BnaA01g05010.1D2
G50	BnaC01g01440.1D2	BnaA01g00740.1D2
G50	BnaC01g01460.1D2	BnaA01g00760.1D2
G50	BnaC01g01480.1D2	BnaA01g00790.1D2
G50	BnaC01g01490.1D2	BnaA01g00800.1D2
G50	BnaC01g01510.1D2	BnaA01g00820.1D2
G50	BnaC01g01530.1D2	BnaA01g00830.1D2
G50	BnaC01g01540.1D2	BnaA01g00840.1D2
G50	BnaC01g01550.1D2	BnaA01g00850.1D2
G50	BnaC01g01560.1D2	BnaA01g04930.1D2
G50	BnaC01g01600.1D2	BnaA01g00890.1D2
G50	BnaC01g01610.1D2	BnaA01g00900.1D2
G50	BnaC01g01650.1D2	BnaA01g00950.1D2
G50	BnaC01g01660.1D2	BnaA01g00960.1D2
G50	BnaC01g01680.1D2	BnaA01g00980.1D2
G50	BnaC01g01700.1D2	BnaA01g01000.1D2
G50	BnaC01g01920.1D2	BnaA01g01230.1D2
G50	BnaC01g01990.1D2	BnaA01g01300.1D2
G50	BnaC01g02000.1D2	BnaA01g01310.1D2
G50	BnaC01g02210.1D2	BnaA01g01500.1D2
G50	BnaC01g02230.1D2	BnaA01g01520.1D2
G50	BnaC01g02300.1D2	BnaA01g01590.1D2
G50	BnaC01g02310.1D2	BnaA01g01600.1D2
G50	BnaC01g02390.1D2	BnaA01g01670.1D2
G50	BnaC01g02410.1D2	BnaA01g01700.1D2
G50	BnaC01g02470.1D2	BnaA01g01760.1D2
G50	BnaC01g02480.1D2	BnaA01g01770.1D2
G50	BnaC01g02490.1D2	BnaA01g01780.1D2
G50	BnaC01g02520.1D2	BnaA01g01810.1D2
G50	BnaC01g02610.1D2	BnaA01g01920.1D2
G50	BnaC01g02660.1D2	BnaA01g01970.1D2
G50	BnaC01g02670.1D2	BnaA01g01980.1D2
G50	BnaC01g02820.1D2	BnaA01g05170.1D2
G50	BnaC01g02870.1D2	BnaA01g05200.1D2
G50	BnaC01g02970.1D2	BnaA01g02180.1D2
G50	BnaC01g03080.1D2	BnaA01g02290.1D2
G50	BnaC01g03090.1D2	BnaA01g02300.1D2
G50	BnaC01g03150.1D2	BnaA01g02360.1D2
G50	BnaC01g03170.1D2	BnaA01g02380.1D2
G50	BnaC01g03300.1D2	BnaA01g02500.1D2
G50	BnaC01g03480.1D2	BnaA01g02630.1D2

G50	BnaC01g03500.1D2	BnaA01g02640.1D2
G50	BnaC01g03640.1D2	BnaA01g02770.1D2
G50	BnaC01g03710.1D2	BnaA01g02850.1D2
G50	BnaC01g03720.1D2	BnaA01g02860.1D2
G50	BnaC01g04050.1D2	BnaA01g03130.1D2
G50	BnaC01g04190.1D2	BnaA01g03250.1D2
G50	BnaC01g04210.1D2	BnaA01g03270.1D2
G50	BnaC01g04230.1D2	BnaA01g03290.1D2
G50	BnaC01g04320.1D2	BnaA01g03380.1D2
G50	BnaC01g04340.1D2	BnaA01g03390.1D2
G50	BnaC01g04370.1D2	BnaA01g03410.1D2
G50	BnaC01g04400.1D2	BnaA01g03440.1D2
G50	BnaC01g04460.1D2	BnaA01g03500.1D2
G50	BnaC01g04720.1D2	BnaA01g03430.1D2
G50	BnaC01g04830.1D2	BnaA01g03810.1D2
G50	BnaC01g04930.1D2	BnaA01g03890.1D2
G50	BnaC01g05100.1D2	BnaA01g04100.1D2
G50	BnaC01g05190.1D2	BnaA01g04160.1D2
G50	BnaC01g05210.1D2	BnaA01g04180.1D2
G50	BnaC01g05310.1D2	BnaA01g04280.1D2
G50	BnaC01g05320.1D2	BnaA01g04280.1D2
G50	BnaC01g07640.1D2	BnaA01g04060.1D2
G50	BnaC01g07660.1D2	BnaA01g00950.1D2
G50	BnaC01g07670.1D2	BnaA01g04070.1D2
G50	BnaC01g07710.1D2	BnaA01g05070.1D2
G50	BnaC01g19590.1D2	BnaA01g17940.1D2
S_39	BnaC01g06750.1D2	BnaA01g31260.1D2
S_39	BnaC01g27090.1D2	BnaA01g24330.1D2
S_39	BnaC01g30060.1D2	BnaA01g26080.1D2
S_39	BnaC01g30110.1D2	BnaA01g26110.1D2
S_39	BnaC01g30470.1D2	BnaA01g26520.1D2
S_39	BnaC01g30480.1D2	BnaA01g26540.1D2
S_39	BnaC01g30490.1D2	BnaA01g26550.1D2
S_39	BnaC01g30530.1D2	BnaA01g26590.1D2
S_39	BnaC01g30540.1D2	BnaA01g26600.1D2
S_39	BnaC01g30550.1D2	BnaA01g26610.1D2
S_39	BnaC01g30570.1D2	BnaA01g26630.1D2
S_39	BnaC01g30580.1D2	BnaA01g26640.1D2
S_39	BnaC01g30660.1D2	BnaA01g26710.1D2
S_39	BnaC01g30670.1D2	BnaA01g26700.1D2
S_39	BnaC01g30690.1D2	BnaA01g26690.1D2
S_39	BnaC01g30690.1D2	BnaA01g26690.1D2
S_39	BnaC01g30690.1D2	BnaA01g26690.1D2
S_39	BnaC01g30780.1D2	BnaA01g26790.1D2
S_39	BnaC01g30810.1D2	BnaA01g26900.1D2
S_39	BnaC01g30840.1D2	BnaA01g26930.1D2
S_39	BnaC01g30850.1D2	BnaA01g26940.1D2
S_39	BnaC01g30860.1D2	BnaA01g26950.1D2
S_39	BnaC01g30930.1D2	BnaA01g26170.1D2
S_39	BnaC01g31220.1D2	BnaA01g27510.1D2
S_39	BnaC01g31290.1D2	BnaA01g27100.1D2

S_39	BnaC01g31300.1D2	BnaA01g27110.1D2
S_39	BnaC01g31310.1D2	BnaA01g27120.1D2
S_39	BnaC01g31330.1D2	BnaA01g27140.1D2
S_39	BnaC01g31340.1D2	BnaA01g27180.1D2
S_39	BnaC01g31350.1D2	BnaA01g27190.1D2
S_39	BnaC01g31430.1D2	BnaA01g27570.1D2
S_39	BnaC01g31530.1D2	BnaA01g27280.1D2
S_39	BnaC01g31780.1D2	BnaA01g27760.1D2
S_39	BnaC01g31920.1D2	BnaA01g27900.1D2
S_39	BnaC01g31970.1D2	BnaA01g27930.1D2
S_39	BnaC01g32110.1D2	BnaA01g27260.1D2
S_39	BnaC01g32120.1D2	BnaA01g27250.1D2
S_39	BnaC01g32140.1D2	BnaA01g27230.1D2
S_39	BnaC01g32160.1D2	BnaA01g27210.1D2
S_39	BnaC01g32280.1D2	BnaA01g28060.1D2
S_39	BnaC01g32320.1D2	BnaA01g28110.1D2
S_39	BnaC01g32330.1D2	BnaA01g28120.1D2
S_39	BnaC01g32340.1D2	BnaA01g27980.1D2
S_39	BnaC01g32410.1D2	BnaA01g28200.1D2
S_39	BnaC01g32420.1D2	BnaA01g28210.1D2
S_39	BnaC01g32450.1D2	BnaA01g28230.1D2
S_39	BnaC01g32480.1D2	BnaA01g28260.1D2
S_39	BnaC01g32500.1D2	BnaA01g28280.1D2
S_39	BnaC01g32530.1D2	BnaA01g28900.1D2
S_39	BnaC01g32580.1D2	BnaA01g28310.1D2
S_39	BnaC01g32640.1D2	BnaA01g28570.1D2
S_39	BnaC01g32710.1D2	BnaA01g28490.1D2
S_39	BnaC01g32730.1D2	BnaA01g28470.1D2
S_39	BnaC01g32740.1D2	BnaA01g28460.1D2
S_39	BnaC01g32760.1D2	BnaA01g28440.1D2
S_39	BnaC01g32870.1D2	BnaA01g27980.1D2
S_39	BnaC01g32880.1D2	BnaA01g27990.1D2
S_39	BnaC01g32890.1D2	BnaA01g28000.1D2
S_39	BnaC01g32900.1D2	BnaA01g27090.1D2
S_39	BnaC01g32960.1D2	BnaA01g26450.1D2
S_39	BnaC01g32980.1D2	BnaA01g28370.1D2
S_39	BnaC01g33010.1D2	BnaA01g28990.1D2
S_39	BnaC01g33080.1D2	BnaA01g27730.1D2
S_39	BnaC01g33240.1D2	BnaA01g28640.1D2
S_39	BnaC01g33270.1D2	BnaA01g28660.1D2
S_39	BnaC01g33310.1D2	BnaA01g28700.1D2
S_39	BnaC01g33340.1D2	BnaA01g28720.1D2
S_39	BnaC01g33370.1D2	BnaA01g28740.1D2
S_39	BnaC01g33380.1D2	BnaA01g28750.1D2
S_39	BnaC01g33420.1D2	BnaA01g28780.1D2
S_39	BnaC01g33430.1D2	BnaA01g28790.1D2
S_39	BnaC01g33630.1D2	BnaA01g30690.1D2
S_39	BnaC01g33690.1D2	BnaA01g30760.1D2
S_39	BnaC01g33720.1D2	BnaA01g30780.1D2
S_39	BnaC01g33720.1D2	BnaA01g31090.1D2
S_39	BnaC01g33730.1D2	BnaA01g30780.1D2

S_39	BnaC01g33730.1D2	BnaA01g31080.1D2
S_39	BnaC01g33780.1D2	BnaA01g31050.1D2
S_39	BnaC01g33940.1D2	BnaA01g30970.1D2
S_39	BnaC01g33950.1D2	BnaA01g30980.1D2
S_39	BnaC01g33970.1D2	BnaA01g30990.1D2
S_39	BnaC01g34060.1D2	BnaA01g32640.1D2
S_39	BnaC01g34090.1D2	BnaA01g32680.1D2
S_39	BnaC01g34160.1D2	BnaA01g31000.1D2
S_39	BnaC01g34230.1D2	BnaA01g31760.1D2
S_39	BnaC01g34440.1D2	BnaA01g32580.1D2
S_39	BnaC01g34550.1D2	BnaA01g31130.1D2
S_39	BnaC01g34620.1D2	BnaA01g31170.1D2
S_39	BnaC01g34690.1D2	BnaA01g31230.1D2
S_39	BnaC01g34700.1D2	BnaA01g31240.1D2
S_39	BnaC01g34720.1D2	BnaA01g31260.1D2
S_39	BnaC01g34750.1D2	BnaA01g31280.1D2
S_39	BnaC01g34770.1D2	BnaA01g31300.1D2
S_39	BnaC01g34850.1D2	BnaA01g31340.1D2
S_39	BnaC01g34860.1D2	BnaA01g31340.1D2
S_39	BnaC01g34880.1D2	BnaA01g31360.1D2
S_39	BnaC01g34910.1D2	BnaA01g31400.1D2
S_39	BnaC01g34920.1D2	BnaA01g31400.1D2
S_39	BnaC01g34960.1D2	BnaA01g31430.1D2
S_39	BnaC01g35030.1D2	BnaA01g31490.1D2
S_39	BnaC01g35060.1D2	BnaA01g31550.1D2
S_39	BnaC01g35070.1D2	BnaA01g31550.1D2
S_39	BnaC01g35110.1D2	BnaA01g31590.1D2
S_39	BnaC01g36100.1D2	BnaA01g34040.1D2
S_39	BnaC01g36750.1D2	BnaA01g35170.1D2
S_39	BnaC01g36780.1D2	BnaA01g35210.1D2
S_39	BnaC01g36790.1D2	BnaA01g35220.1D2
S_39	BnaC01g36800.1D2	BnaA01g35230.1D2
S_39	BnaC01g36810.1D2	BnaA01g35240.1D2
S_39	BnaC01g36840.1D2	BnaA01g35260.1D2
S_39	BnaC01g36860.1D2	BnaA01g35270.1D2
S_39	BnaC01g36870.1D2	BnaA01g35280.1D2
S_39	BnaC01g36980.1D2	BnaA01g35370.1D2
S_39	BnaC01g37030.1D2	BnaA01g35420.1D2
S_39	BnaC01g37120.1D2	BnaA01g35520.1D2
S_39	BnaC01g37330.1D2	BnaA01g35720.1D2
S_39	BnaC01g37390.1D2	BnaA01g35770.1D2
Major	BnaC01g18150.1D2	BnaA01g16830.1D2
Major	BnaC01g19260.1D2	BnaA01g17480.1D2
Major	BnaC01g19280.1D2	BnaA01g17500.1D2
Major	BnaC01g19550.1D2	BnaA01g17760.1D2
Major	BnaC01g19980.1D2	BnaA01g17280.1D2
Major	BnaC01g20220.1D2	BnaA01g17860.1D2
Major	BnaC01g20240.1D2	BnaA01g17880.1D2
Major	BnaC01g20250.1D2	BnaA01g17880.1D2
Major	BnaC01g20260.1D2	BnaA01g17890.1D2
Major	BnaC01g32420.1D2	BnaA01g28210.1D2

Major	BnaC01g32530.1D2	BnaA01g28900.1D2
Major	BnaC01g32640.1D2	BnaA01g28570.1D2
Major	BnaC01g32760.1D2	BnaA01g28440.1D2
Major	BnaC01g32880.1D2	BnaA01g27990.1D2
Major	BnaC01g32890.1D2	BnaA01g28000.1D2
Major	BnaC01g32980.1D2	BnaA01g28370.1D2
Major	BnaC01g33240.1D2	BnaA01g28640.1D2
Major	BnaC01g33310.1D2	BnaA01g28700.1D2
Major	BnaC01g33380.1D2	BnaA01g28750.1D2
Major	BnaC01g33420.1D2	BnaA01g28780.1D2
Major	BnaC01g33660.1D2	BnaA01g30720.1D2
Major	BnaC01g33720.1D2	BnaA01g31090.1D2
Major	BnaC01g33850.1D2	BnaA01g30880.1D2
Major	BnaC01g33940.1D2	BnaA01g30970.1D2
Major	BnaC01g33950.1D2	BnaA01g30980.1D2
Major	BnaC01g34120.1D2	BnaA01g32700.1D2
Major	BnaC01g34240.1D2	BnaA01g31740.1D2
Major	BnaC01g34270.1D2	BnaA01g31710.1D2
Major	BnaC01g34300.1D2	BnaA01g31650.1D2
Major	BnaC01g34350.1D2	BnaA01g31670.1D2
Major	BnaC01g34480.1D2	BnaA01g29180.1D2
Major	BnaC01g34880.1D2	BnaA01g31360.1D2
Major	BnaC01g34960.1D2	BnaA01g31430.1D2
Major	BnaC01g34970.1D2	BnaA01g31440.1D2
Major	BnaC01g35060.1D2	BnaA01g31550.1D2
Major	BnaC01g35390.1D2	BnaA01g32250.1D2
Major	BnaC01g35490.1D2	BnaA01g33630.1D2
Major	BnaC01g35500.1D2	BnaA01g33640.1D2
Major	BnaC01g35510.1D2	BnaA01g33650.1D2
Major	BnaC01g35520.1D2	BnaA01g33670.1D2
Major	BnaC01g35540.1D2	BnaA01g33690.1D2
Major	BnaC01g35550.1D2	BnaA01g33710.1D2
Major	BnaC01g35710.1D2	BnaA01g33840.1D2
Major	BnaC01g36840.1D2	BnaA01g35260.1D2
Major	BnaC01g36980.1D2	BnaA01g35370.1D2
Major	BnaC01g37030.1D2	BnaA01g35420.1D2
Major	BnaC01g37120.1D2	BnaA01g35520.1D2
Major	BnaC01g37390.1D2	BnaA01g35770.1D2
H165	BnaC01g06750.1D2	BnaA01g31260.1D2
H165	BnaC01g18500.1D2	BnaA01g16660.1D2
H165	BnaC01g18520.1D2	BnaA01g16670.1D2
H165	BnaC01g18610.1D2	BnaA01g15930.1D2
H165	BnaC01g18880.1D2	BnaA01g16210.1D2
H165	BnaC01g18890.1D2	BnaA01g16220.1D2
H165	BnaC01g18930.1D2	BnaA01g16270.1D2
H165	BnaC01g19230.1D2	BnaA01g17450.1D2
H165	BnaC01g19300.1D2	BnaA01g17520.1D2
H165	BnaC01g19550.1D2	BnaA01g17760.1D2
H165	BnaC01g19570.1D2	BnaA01g16550.1D2
H165	BnaC01g22770.1D2	BnaA01g19500.1D2
H165	BnaC01g27090.1D2	BnaA01g24330.1D2

H165	BnaC01g29970.1D2	BnaA01g29010.1D2
H165	BnaC01g30010.1D2	BnaA01g26040.1D2
H165	BnaC01g30100.1D2	BnaA01g26100.1D2
H165	BnaC01g30460.1D2	BnaA01g26510.1D2
H165	BnaC01g30480.1D2	BnaA01g26540.1D2
H165	BnaC01g30490.1D2	BnaA01g26550.1D2
H165	BnaC01g30550.1D2	BnaA01g26610.1D2
H165	BnaC01g30570.1D2	BnaA01g26630.1D2
H165	BnaC01g30670.1D2	BnaA01g26700.1D2
H165	BnaC01g30690.1D2	BnaA01g26690.1D2
H165	BnaC01g30690.1D2	BnaA01g26690.1D2
H165	BnaC01g30690.1D2	BnaA01g26690.1D2
H165	BnaC01g30860.1D2	BnaA01g26950.1D2
H165	BnaC01g31300.1D2	BnaA01g27110.1D2
H165	BnaC01g31310.1D2	BnaA01g27120.1D2
H165	BnaC01g31430.1D2	BnaA01g27570.1D2
H165	BnaC01g31480.1D2	BnaA01g27610.1D2
H165	BnaC01g31590.1D2	BnaA01g27330.1D2
H165	BnaC01g31780.1D2	BnaA01g27760.1D2
H165	BnaC01g31800.1D2	BnaA01g27770.1D2
H165	BnaC01g32110.1D2	BnaA01g27260.1D2
H165	BnaC01g32490.1D2	BnaA01g28270.1D2
H165	BnaC01g32500.1D2	BnaA01g28280.1D2
H165	BnaC01g32530.1D2	BnaA01g28900.1D2
H165	BnaC01g32640.1D2	BnaA01g28570.1D2
H165	BnaC01g32980.1D2	BnaA01g28370.1D2
H165	BnaC01g33380.1D2	BnaA01g28750.1D2
H165	BnaC01g33420.1D2	BnaA01g28780.1D2
H165	BnaC01g33590.1D2	BnaA01g27500.1D2
H165	BnaC01g33630.1D2	BnaA01g30690.1D2
H165	BnaC01g33680.1D2	BnaA01g30750.1D2
H165	BnaC01g33730.1D2	BnaA01g30780.1D2
H165	BnaC01g33730.1D2	BnaA01g31080.1D2
H165	BnaC01g33780.1D2	BnaA01g31050.1D2
H165	BnaC01g34120.1D2	BnaA01g32700.1D2
H165	BnaC01g34130.1D2	BnaA01g32710.1D2
H165	BnaC01g34160.1D2	BnaA01g31000.1D2
H165	BnaC01g34620.1D2	BnaA01g31170.1D2
H165	BnaC01g34720.1D2	BnaA01g31260.1D2
H165	BnaC01g34770.1D2	BnaA01g31300.1D2
H165	BnaC01g34850.1D2	BnaA01g31340.1D2
H165	BnaC01g34960.1D2	BnaA01g31430.1D2
H165	BnaC01g35060.1D2	BnaA01g31550.1D2
H165	BnaC01g35070.1D2	BnaA01g31550.1D2
H165	BnaC01g35110.1D2	BnaA01g31590.1D2
H165	BnaC01g35540.1D2	BnaA01g33690.1D2
H165	BnaC01g35710.1D2	BnaA01g33840.1D2
H165	BnaC01g36170.1D2	BnaA01g32930.1D2
H165	BnaC01g36710.1D2	BnaA01g35130.1D2
H165	BnaC01g36790.1D2	BnaA01g35220.1D2
H165	BnaC01g36800.1D2	BnaA01g35230.1D2

H165	BnaC01g36840.1D2	BnaA01g35260.1D2
H165	BnaC01g37020.1D2	BnaA01g35410.1D2
H165	BnaC01g37390.1D2	BnaA01g35770.1D2

List of HE genes (C02 to A02)

**Accession**	**Lost gene**	**Duplicated HE gene**
G50	BnaC02g00230.1D2	BnaA02g00560.1D2
G50	BnaC02g00280.1D2	BnaA02g00510.1D2
G50	BnaC02g00300.1D2	BnaA02g00490.1D2
G50	BnaC02g00310.1D2	BnaA02g00480.1D2
G50	BnaC02g00480.1D2	BnaA02g00310.1D2
G50	BnaC02g00530.1D2	BnaA02g00260.1D2
G50	BnaC02g00550.1D2	BnaA02g00240.1D2
G50	BnaC02g00680.1D2	BnaA02g00040.1D2
G50	BnaC02g00730.1D2	BnaA02g00020.1D2
G50	BnaC02g11060.1D2	BnaA02g07950.1D2
G50	BnaC02g11130.1D2	BnaA02g08030.1D2
G50	BnaC02g11550.1D2	BnaA02g08360.1D2
G50	BnaC02g11570.1D2	BnaA02g08370.1D2
G50	BnaC02g11660.1D2	BnaA02g08430.1D2
G50	BnaC02g11730.1D2	BnaA02g08530.1D2
G50	BnaC02g11840.1D2	BnaA02g08670.1D2
G50	BnaC02g11930.1D2	BnaA02g08770.1D2
G50	BnaC02g11960.1D2	BnaA02g08780.1D2
G50	BnaC02g11970.1D2	BnaA02g08790.1D2
G50	BnaC02g12080.1D2	BnaA02g08900.1D2
G50	BnaC02g12120.1D2	BnaA02g08940.1D2
G50	BnaC02g12210.1D2	BnaA02g09000.1D2
G50	BnaC02g12230.1D2	BnaA02g08980.1D2
G50	BnaC02g12250.1D2	BnaA02g09110.1D2
G50	BnaC02g12290.1D2	BnaA02g09120.1D2
G50	BnaC02g12400.1D2	BnaA02g08470.1D2
G50	BnaC02g12440.1D2	BnaA02g09300.1D2
G50	BnaC02g12530.1D2	BnaA02g09380.1D2
G50	BnaC02g12590.1D2	BnaA02g09420.1D2
G50	BnaC02g12750.1D2	BnaA02g09520.1D2
G50	BnaC02g12930.1D2	BnaA02g09750.1D2
G50	BnaC02g12980.1D2	BnaA02g09780.1D2
G50	BnaC02g13100.1D2	BnaA02g11230.1D2
G50	BnaC02g13560.1D2	BnaA02g10090.1D2
G50	BnaC02g13570.1D2	BnaA02g10100.1D2
G50	BnaC02g13590.1D2	BnaA02g10120.1D2
G50	BnaC02g13610.1D2	BnaA02g10140.1D2
G50	BnaC02g13630.1D2	BnaA02g10160.1D2
G50	BnaC02g13650.1D2	BnaA02g10180.1D2
G50	BnaC02g13720.1D2	BnaA02g10260.1D2
G50	BnaC02g13740.1D2	BnaA02g10280.1D2
G50	BnaC02g13790.1D2	BnaA02g10410.1D2
G50	BnaC02g13910.1D2	BnaA02g10590.1D2
G50	BnaC02g14050.1D2	BnaA02g13340.1D2
G50	BnaC02g14200.1D2	BnaA02g11310.1D2

G50	BnaC02g14290.1D2	BnaA02g10890.1D2
G50	BnaC02g14620.1D2	BnaA02g11380.1D2
G50	BnaC02g14640.1D2	BnaA02g11400.1D2
G50	BnaC02g14700.1D2	BnaA02g11440.1D2
G50	BnaC02g14810.1D2	BnaA02g11500.1D2
G50	BnaC02g14840.1D2	BnaA02g11530.1D2
G50	BnaC02g14860.1D2	BnaA02g11540.1D2
G50	BnaC02g14870.1D2	BnaA02g11560.1D2
G50	BnaC02g15040.1D2	BnaA02g11300.1D2
G50	BnaC02g15250.1D2	BnaA02g11660.1D2
G50	BnaC02g15280.1D2	BnaA02g11680.1D2
G50	BnaC02g15300.1D2	BnaA02g13650.1D2
G50	BnaC02g15580.1D2	BnaA02g11720.1D2
G50	BnaC02g15760.1D2	BnaA02g11910.1D2
G50	BnaC02g15770.1D2	BnaA02g11920.1D2
G50	BnaC02g15860.1D2	BnaA02g12030.1D2
G50	BnaC02g15880.1D2	BnaA02g12040.1D2
G50	BnaC02g15930.1D2	BnaA02g12480.1D2
G50	BnaC02g15940.1D2	BnaA02g12490.1D2
G50	BnaC02g15990.1D2	BnaA02g12160.1D2
G50	BnaC02g16130.1D2	BnaA02g12620.1D2
G50	BnaC02g16190.1D2	BnaA02g12690.1D2
G50	BnaC02g16200.1D2	BnaA02g12690.1D2
G50	BnaC02g16220.1D2	BnaA02g12710.1D2
G50	BnaC02g16250.1D2	BnaA02g12760.1D2
G50	BnaC02g16260.1D2	BnaA02g12770.1D2
G50	BnaC02g16280.1D2	BnaA02g12800.1D2
G50	BnaC02g16330.1D2	BnaA02g12920.1D2
G50	BnaC02g16350.1D2	BnaA02g12940.1D2
G50	BnaC02g16390.1D2	BnaA02g12980.1D2
G50	BnaC02g16400.1D2	BnaA02g12990.1D2
G50	BnaC02g16440.1D2	BnaA02g13040.1D2
G50	BnaC02g16500.1D2	BnaA02g13070.1D2
G50	BnaC02g16520.1D2	BnaA02g13100.1D2
G50	BnaC02g16560.1D2	BnaA02g13150.1D2
G50	BnaC02g16580.1D2	BnaA02g13160.1D2
G50	BnaC02g16590.1D2	BnaA02g13170.1D2
G50	BnaC02g16600.1D2	BnaA02g13180.1D2
G50	BnaC02g16610.1D2	BnaA02g13190.1D2
G50	BnaC02g16670.1D2	BnaA02g13250.1D2
G50	BnaC02g17180.1D2	BnaA02g11860.1D2
G50	BnaC02g17550.1D2	BnaA02g13710.1D2
G50	BnaC02g17570.1D2	BnaA02g13680.1D2
G50	BnaC02g17810.1D2	BnaA02g13460.1D2
G50	BnaC02g17820.1D2	BnaA02g13450.1D2
G50	BnaC02g17830.1D2	BnaA02g13440.1D2
G50	BnaC02g17930.1D2	BnaA02g13620.1D2
G50	BnaC02g17980.1D2	BnaA02g13410.1D2
G50	BnaC02g17990.1D2	BnaA02g13400.1D2
G50	BnaC02g18170.1D2	BnaA02g13280.1D2
G50	BnaC02g18200.1D2	BnaA02g13310.1D2

G50	BnaC02g31440.1D2	BnaA02g24810.1D2
G50	BnaC02g31550.1D2	BnaA02g25510.1D2
G50	BnaC02g31690.1D2	BnaA02g24900.1D2
G50	BnaC02g31710.1D2	BnaA02g24910.1D2
G50	BnaC02g31740.1D2	BnaA02g25070.1D2
G50	BnaC02g31820.1D2	BnaA02g25000.1D2
G50	BnaC02g31910.1D2	BnaA02g25140.1D2
G50	BnaC02g32070.1D2	BnaA02g25580.1D2
G50	BnaC02g32110.1D2	BnaA02g25620.1D2
G50	BnaC02g32140.1D2	BnaA02g25650.1D2
G50	BnaC02g32310.1D2	BnaA02g26970.1D2
G50	BnaC02g32320.1D2	BnaA02g26970.1D2
G50	BnaC02g32380.1D2	BnaA02g26920.1D2
G50	BnaC02g32390.1D2	BnaA02g27170.1D2
G50	BnaC02g32490.1D2	BnaA02g26840.1D2
G50	BnaC02g32680.1D2	BnaA02g25820.1D2
G50	BnaC02g32880.1D2	BnaA02g26700.1D2
H176	BnaC02g12980.1D2	BnaA02g09780.1D2
H176	BnaC02g14050.1D2	BnaA02g13340.1D2
H176	BnaC02g16280.1D2	BnaA02g12800.1D2
H176	BnaC02g16310.1D2	BnaA02g12850.1D2
H176	BnaC02g16330.1D2	BnaA02g12920.1D2
H176	BnaC02g16350.1D2	BnaA02g12940.1D2
H176	BnaC02g16380.1D2	BnaA02g12970.1D2
H176	BnaC02g16390.1D2	BnaA02g12980.1D2
H176	BnaC02g16400.1D2	BnaA02g12990.1D2
H176	BnaC02g21060.1D2	BnaA02g16930.1D2
H176	BnaC02g26400.1D2	BnaA02g19060.1D2
H176	BnaC02g35650.1D2	BnaA02g29110.1D2
H176	BnaC02g35660.1D2	BnaA02g29120.1D2
H176	BnaC02g35810.1D2	BnaA02g29220.1D2
H176	BnaC02g35840.1D2	BnaA02g29230.1D2
H176	BnaC02g35850.1D2	BnaA02g29240.1D2
H176	BnaC02g35870.1D2	BnaA02g29270.1D2
H176	BnaC02g35910.1D2	BnaA02g29300.1D2
H176	BnaC02g35920.1D2	BnaA02g29310.1D2
H176	BnaC02g35930.1D2	BnaA02g29320.1D2
H176	BnaC02g35950.1D2	BnaA02g29340.1D2
H176	BnaC02g35960.1D2	BnaA02g29350.1D2
H176	BnaC02g36300.1D2	BnaA02g29520.1D2
H176	BnaC02g36340.1D2	BnaA02g29540.1D2
H176	BnaC02g36380.1D2	BnaA02g29580.1D2
H176	BnaC02g36390.1D2	BnaA02g29590.1D2
H176	BnaC02g36440.1D2	BnaA02g29640.1D2
H176	BnaC02g36460.1D2	BnaA02g29660.1D2
H176	BnaC02g36470.1D2	BnaA02g29670.1D2
H176	BnaC02g36500.1D2	BnaA02g29880.1D2
H176	BnaC02g36510.1D2	BnaA02g29690.1D2
H176	BnaC02g36530.1D2	BnaA02g29710.1D2
H176	BnaC02g36560.1D2	BnaA02g29720.1D2
H176	BnaC02g36570.1D2	BnaA02g29730.1D2

H176	BnaC02g36580.1D2	BnaA02g29740.1D2
H176	BnaC02g36600.1D2	BnaA02g29750.1D2
H176	BnaC02g36610.1D2	BnaA02g29760.1D2
H176	BnaC02g36620.1D2	BnaA02g29770.1D2
H176	BnaC02g36710.1D2	BnaA02g29840.1D2
H176	BnaC02g36760.1D2	BnaA02g29910.1D2
H176	BnaC02g36770.1D2	BnaA02g29920.1D2
H176	BnaC02g36810.1D2	BnaA02g29940.1D2
H176	BnaC02g36870.1D2	BnaA02g30080.1D2
H176	BnaC02g36880.1D2	BnaA02g30090.1D2
H176	BnaC02g36960.1D2	BnaA02g30120.1D2
H176	BnaC02g36990.1D2	BnaA02g30150.1D2
H176	BnaC02g37000.1D2	BnaA02g30230.1D2
H176	BnaC02g37010.1D2	BnaA02g30230.1D2
H176	BnaC02g37060.1D2	BnaA02g30290.1D2
H176	BnaC02g37090.1D2	BnaA02g30370.1D2
H176	BnaC02g37160.1D2	BnaA02g30350.1D2
H176	BnaC02g37210.1D2	BnaA02g30440.1D2
H176	BnaC02g37230.1D2	BnaA02g30470.1D2
H176	BnaC02g37270.1D2	BnaA02g30500.1D2
H176	BnaC02g37310.1D2	BnaA02g30530.1D2
H176	BnaC02g37440.1D2	BnaA02g32190.1D2
H176	BnaC02g37460.1D2	BnaA02g32180.1D2
H176	BnaC02g37470.1D2	BnaA02g32170.1D2
H176	BnaC02g37480.1D2	BnaA02g32170.1D2
H176	BnaC02g37500.1D2	BnaA02g29390.1D2
H176	BnaC02g37580.1D2	BnaA02g29990.1D2
H176	BnaC02g37700.1D2	BnaA02g29960.1D2
H176	BnaC02g37710.1D2	BnaA02g32160.1D2
H176	BnaC02g37720.1D2	BnaA02g32150.1D2
H176	BnaC02g37750.1D2	BnaA02g32140.1D2
H176	BnaC02g37810.1D2	BnaA02g30600.1D2
H176	BnaC02g37820.1D2	BnaA02g30610.1D2
H176	BnaC02g37840.1D2	BnaA02g30630.1D2
H176	BnaC02g37860.1D2	BnaA02g30660.1D2
H176	BnaC02g37880.1D2	BnaA02g30680.1D2
H176	BnaC02g37890.1D2	BnaA02g30690.1D2
H176	BnaC02g37950.1D2	BnaA02g30750.1D2
H176	BnaC02g38000.1D2	BnaA02g30790.1D2
H176	BnaC02g38040.1D2	BnaA02g30840.1D2
H176	BnaC02g38100.1D2	BnaA02g30940.1D2
H176	BnaC02g38180.1D2	BnaA02g31000.1D2
H176	BnaC02g38280.1D2	BnaA02g31120.1D2
H176	BnaC02g38300.1D2	BnaA02g31140.1D2
H176	BnaC02g38320.1D2	BnaA02g31160.1D2
H176	BnaC02g38350.1D2	BnaA02g31220.1D2
H176	BnaC02g38960.1D2	BnaA02g31290.1D2
H176	BnaC02g38970.1D2	BnaA02g31310.1D2
H176	BnaC02g38990.1D2	BnaA02g31320.1D2
H176	BnaC02g39150.1D2	BnaA02g31050.1D2
H176	BnaC02g39370.1D2	BnaA02g32110.1D2

R76	BnaC02g07530.1D2	BnaA02g02110.1D2
R76	BnaC02g07650.1D2	BnaA02g02180.1D2
R76	BnaC02g07660.1D2	BnaA02g02190.1D2
R76	BnaC02g07700.1D2	BnaA02g02230.1D2
R76	BnaC02g07790.1D2	BnaA02g02320.1D2
R76	BnaC02g07800.1D2	BnaA02g02340.1D2
R76	BnaC02g07850.1D2	BnaA02g02390.1D2
R76	BnaC02g08250.1D2	BnaA02g02780.1D2
R76	BnaC02g08490.1D2	BnaA02g04250.1D2
R76	BnaC02g08580.1D2	BnaA02g02990.1D2
R76	BnaC02g08620.1D2	BnaA02g03040.1D2
R76	BnaC02g08630.1D2	BnaA02g03050.1D2
R76	BnaC02g08830.1D2	BnaA02g03240.1D2
R76	BnaC02g08950.1D2	BnaA02g03350.1D2
R76	BnaC02g09010.1D2	BnaA02g03380.1D2
R76	BnaC02g09040.1D2	BnaA02g03380.1D2
R76	BnaC02g09170.1D2	BnaA02g03500.1D2
R76	BnaC02g09220.1D2	BnaA02g04420.1D2
R76	BnaC02g09260.1D2	BnaA02g04460.1D2
R76	BnaC02g09530.1D2	BnaA02g04810.1D2
R76	BnaC02g09580.1D2	BnaA02g04840.1D2
R76	BnaC02g10050.1D2	BnaA02g05100.1D2
R76	BnaC02g10070.1D2	BnaA02g05130.1D2
R76	BnaC02g10500.1D2	BnaA02g05570.1D2
R76	BnaC02g10560.1D2	BnaA02g05590.1D2
R76	BnaC02g10560.1D2	BnaA02g05600.1D2
R76	BnaC02g10710.1D2	BnaA02g05740.1D2
R76	BnaC02g12270.1D2	BnaA02g05320.1D2
R76	BnaC02g33660.1D2	BnaA02g26650.1D2
R76	BnaC02g37160.1D2	BnaA02g30350.1D2
R76	BnaC02g39630.1D2	BnaA02g32570.1D2
RS_10_7	BnaC02g00110.1D2	BnaA02g00630.1D2
RS_10_7	BnaC02g00230.1D2	BnaA02g00560.1D2
RS_10_7	BnaC02g00300.1D2	BnaA02g00490.1D2
RS_10_7	BnaC02g00410.1D2	BnaA02g00380.1D2
RS_10_7	BnaC02g00730.1D2	BnaA02g00020.1D2
RS_10_7	BnaC02g05990.1D2	BnaA02g00950.1D2
RS_10_7	BnaC02g06080.1D2	BnaA02g01010.1D2
RS_10_7	BnaC02g06220.1D2	BnaA02g04220.1D2
RS_10_7	BnaC02g06410.1D2	BnaA02g01090.1D2
RS_10_7	BnaC02g06420.1D2	BnaA02g01100.1D2
RS_10_7	BnaC02g06440.1D2	BnaA02g01120.1D2
RS_10_7	BnaC02g06440.1D2	BnaA02g01140.1D2
RS_10_7	BnaC02g06450.1D2	BnaA02g01150.1D2
RS_10_7	BnaC02g06810.1D2	BnaA02g01510.1D2
RS_10_7	BnaC02g06870.1D2	BnaA02g01570.1D2
RS_10_7	BnaC02g06910.1D2	BnaA02g01610.1D2
RS_10_7	BnaC02g06990.1D2	BnaA02g01690.1D2
RS_10_7	BnaC02g07200.1D2	BnaA02g01890.1D2
RS_10_7	BnaC02g07290.1D2	BnaA02g01970.1D2
RS_10_7	BnaC02g07790.1D2	BnaA02g02320.1D2

RS_10_7	BnaC02g07850.1D2	BnaA02g02390.1D2
RS_10_7	BnaC02g07900.1D2	BnaA02g02480.1D2
RS_10_7	BnaC02g08100.1D2	BnaA02g02630.1D2
RS_10_7	BnaC02g08150.1D2	BnaA02g02680.1D2
RS_10_7	BnaC02g08490.1D2	BnaA02g04250.1D2
RS_10_7	BnaC02g08580.1D2	BnaA02g02990.1D2
RS_10_7	BnaC02g08630.1D2	BnaA02g03050.1D2
RS_10_7	BnaC02g09040.1D2	BnaA02g03380.1D2
RS_10_7	BnaC02g09150.1D2	BnaA02g03480.1D2
RS_10_7	BnaC02g09220.1D2	BnaA02g04420.1D2
RS_10_7	BnaC02g09560.1D2	BnaA02g04790.1D2
RS_10_7	BnaC02g09580.1D2	BnaA02g04840.1D2
RS_10_7	BnaC02g09710.1D2	BnaA02g04940.1D2
RS_10_7	BnaC02g09750.1D2	BnaA02g03890.1D2
RS_10_7	BnaC02g10070.1D2	BnaA02g05130.1D2
RS_10_7	BnaC02g10150.1D2	BnaA02g05180.1D2
RS_10_7	BnaC02g10190.1D2	BnaA02g05230.1D2
RS_10_7	BnaC02g10190.1D2	BnaA02g05260.1D2
RS_10_7	BnaC02g10280.1D2	BnaA02g05380.1D2
RS_10_7	BnaC02g10570.1D2	BnaA02g05610.1D2
RS_10_7	BnaC02g10780.1D2	BnaA02g06680.1D2
RS_10_7	BnaC02g10810.1D2	BnaA02g06710.1D2
RS_10_7	BnaC02g10930.1D2	BnaA02g06830.1D2
RS_10_7	BnaC02g10940.1D2	BnaA02g06840.1D2
RS_10_7	BnaC02g11120.1D2	BnaA02g08020.1D2
RS_10_7	BnaC02g11230.1D2	BnaA02g08200.1D2
RS_10_7	BnaC02g11290.1D2	BnaA02g08140.1D2
RS_10_7	BnaC02g11320.1D2	BnaA02g08170.1D2
RS_10_7	BnaC02g11550.1D2	BnaA02g08360.1D2
RS_10_7	BnaC02g11570.1D2	BnaA02g08370.1D2
RS_10_7	BnaC02g11590.1D2	BnaA02g08380.1D2
RS_10_7	BnaC02g11730.1D2	BnaA02g08530.1D2
RS_10_7	BnaC02g11800.1D2	BnaA02g08620.1D2
RS_10_7	BnaC02g11960.1D2	BnaA02g08780.1D2
RS_10_7	BnaC02g11970.1D2	BnaA02g08790.1D2
RS_10_7	BnaC02g12110.1D2	BnaA02g08930.1D2
RS_10_7	BnaC02g12250.1D2	BnaA02g09110.1D2
RS_10_7	BnaC02g12290.1D2	BnaA02g09120.1D2
RS_10_7	BnaC02g12440.1D2	BnaA02g09300.1D2
RS_10_7	BnaC02g12950.1D2	BnaA02g09770.1D2
RS_10_7	BnaC02g12980.1D2	BnaA02g09780.1D2
RS_10_7	BnaC02g13100.1D2	BnaA02g11230.1D2
RS_10_7	BnaC02g13910.1D2	BnaA02g10590.1D2
RS_10_7	BnaC02g14050.1D2	BnaA02g13340.1D2
RS_10_7	BnaC02g14130.1D2	BnaA02g10790.1D2
RS_10_7	BnaC02g14220.1D2	BnaA02g11090.1D2
RS_10_7	BnaC02g14310.1D2	BnaA02g10910.1D2
RS_10_7	BnaC02g14620.1D2	BnaA02g11380.1D2
RS_10_7	BnaC02g14640.1D2	BnaA02g11400.1D2
RS_10_7	BnaC02g14840.1D2	BnaA02g11530.1D2
RS_10_7	BnaC02g14860.1D2	BnaA02g11540.1D2

RS_10_7	BnaC02g14870.1D2	BnaA02g11560.1D2
RS_10_7	BnaC02g15240.1D2	BnaA02g11650.1D2
RS_10_7	BnaC02g15250.1D2	BnaA02g11660.1D2
RS_10_7	BnaC02g15280.1D2	BnaA02g11680.1D2
RS_10_7	BnaC02g15290.1D2	BnaA02g11670.1D2
RS_10_7	BnaC02g15290.1D2	BnaA02g11680.1D2
RS_10_7	BnaC02g15360.1D2	BnaA02g10710.1D2
RS_10_7	BnaC02g15490.1D2	BnaA02g07860.1D2
RS_10_7	BnaC02g15890.1D2	BnaA02g12070.1D2
RS_10_7	BnaC02g16020.1D2	BnaA02g12530.1D2
RS_10_7	BnaC02g16140.1D2	BnaA02g12640.1D2
RS_10_7	BnaC02g16190.1D2	BnaA02g12690.1D2
RS_10_7	BnaC02g16280.1D2	BnaA02g12800.1D2
RS_10_7	BnaC02g16390.1D2	BnaA02g12980.1D2
RS_10_7	BnaC02g16500.1D2	BnaA02g13070.1D2
RS_10_7	BnaC02g16580.1D2	BnaA02g13160.1D2
RS_10_7	BnaC02g17550.1D2	BnaA02g13710.1D2
RS_10_7	BnaC02g17720.1D2	BnaA02g13940.1D2
RS_10_7	BnaC02g17730.1D2	BnaA02g13930.1D2
RS_10_7	BnaC02g17730.1D2	BnaA02g13950.1D2
RS_10_7	BnaC02g17760.1D2	BnaA02g13980.1D2
RS_10_7	BnaC02g17870.1D2	BnaA02g13480.1D2
RS_10_7	BnaC02g17930.1D2	BnaA02g13620.1D2
RS_10_7	BnaC02g18290.1D2	BnaA02g14090.1D2
RS_10_7	BnaC02g18540.1D2	BnaA02g14380.1D2
RS_10_7	BnaC02g18610.1D2	BnaA02g14450.1D2
RS_10_7	BnaC02g18640.1D2	BnaA02g14480.1D2
RS_10_7	BnaC02g18680.1D2	BnaA02g14580.1D2
RS_10_7	BnaC02g19040.1D2	BnaA02g15660.1D2
RS_10_7	BnaC02g19210.1D2	BnaA02g14990.1D2
RS_10_7	BnaC02g19240.1D2	BnaA02g15010.1D2
RS_10_7	BnaC02g19380.1D2	BnaA02g15510.1D2
RS_10_7	BnaC02g19430.1D2	BnaA02g15530.1D2
RS_10_7	BnaC02g20340.1D2	BnaA02g15820.1D2
RS_10_7	BnaC02g20420.1D2	BnaA02g15900.1D2
RS_10_7	BnaC02g20530.1D2	BnaA02g15960.1D2
RS_10_7	BnaC02g20560.1D2	BnaA02g15970.1D2
RS_10_7	BnaC02g20580.1D2	BnaA02g15990.1D2
RS_10_7	BnaC02g20890.1D2	BnaA02g16180.1D2
RS_10_7	BnaC02g20950.1D2	BnaA02g16220.1D2
RS_10_7	BnaC02g20970.1D2	BnaA02g16880.1D2
RS_10_7	BnaC02g21060.1D2	BnaA02g16930.1D2
RS_10_7	BnaC02g21120.1D2	BnaA02g16280.1D2
RS_10_7	BnaC02g21130.1D2	BnaA02g16290.1D2
RS_10_7	BnaC02g21240.1D2	BnaA02g16410.1D2
RS_10_7	BnaC02g21300.1D2	BnaA02g16320.1D2
RS_10_7	BnaC02g21310.1D2	BnaA02g15500.1D2
RS_10_7	BnaC02g21490.1D2	BnaA02g16530.1D2
RS_10_7	BnaC02g21670.1D2	BnaA02g17120.1D2
RS_10_7	BnaC02g21680.1D2	BnaA02g17110.1D2
RS_10_7	BnaC02g21900.1D2	BnaA02g17140.1D2

RS_10_7	BnaC02g23070.1D2	BnaA02g17060.1D2
RS_10_7	BnaC02g23100.1D2	BnaA02g17090.1D2
RS_10_7	BnaC02g23310.1D2	BnaA02g17790.1D2
Tapidor	BnaC02g00030.1D2	BnaA02g00710.1D2
Tapidor	BnaC02g00040.1D2	BnaA02g00700.1D2
Tapidor	BnaC02g00050.1D2	BnaA02g00690.1D2
Tapidor	BnaC02g00110.1D2	BnaA02g00630.1D2
Tapidor	BnaC02g00210.1D2	BnaA02g00580.1D2
Tapidor	BnaC02g00230.1D2	BnaA02g00560.1D2
Tapidor	BnaC02g00280.1D2	BnaA02g00510.1D2
Tapidor	BnaC02g00290.1D2	BnaA02g00500.1D2
Tapidor	BnaC02g00330.1D2	BnaA02g00460.1D2
Tapidor	BnaC02g00340.1D2	BnaA02g00750.1D2
Tapidor	BnaC02g00380.1D2	BnaA02g00410.1D2
Tapidor	BnaC02g00560.1D2	BnaA02g00220.1D2
Tapidor	BnaC02g00580.1D2	BnaA02g00200.1D2
Tapidor	BnaC02g00730.1D2	BnaA02g00020.1D2
Tapidor	BnaC02g05970.1D2	BnaA02g00910.1D2
Tapidor	BnaC02g05990.1D2	BnaA02g00950.1D2
Tapidor	BnaC02g06080.1D2	BnaA02g01010.1D2
Tapidor	BnaC02g06090.1D2	BnaA02g01010.1D2
Tapidor	BnaC02g06100.1D2	BnaA02g01020.1D2
Tapidor	BnaC02g06110.1D2	BnaA02g04040.1D2
Tapidor	BnaC02g06190.1D2	BnaA02g03950.1D2
Tapidor	BnaC02g06200.1D2	BnaA02g03940.1D2
Tapidor	BnaC02g06260.1D2	BnaA02g04290.1D2
Tapidor	BnaC02g06280.1D2	BnaA02g04310.1D2
Tapidor	BnaC02g06300.1D2	BnaA02g04170.1D2
Tapidor	BnaC02g06330.1D2	BnaA02g04060.1D2
Tapidor	BnaC02g06380.1D2	BnaA02g01060.1D2
Tapidor	BnaC02g06390.1D2	BnaA02g01070.1D2
Tapidor	BnaC02g06400.1D2	BnaA02g01080.1D2
Tapidor	BnaC02g06440.1D2	BnaA02g01120.1D2
Tapidor	BnaC02g06440.1D2	BnaA02g01140.1D2
Tapidor	BnaC02g06450.1D2	BnaA02g01150.1D2
Tapidor	BnaC02g06470.1D2	BnaA02g01190.1D2
Tapidor	BnaC02g06640.1D2	BnaA02g01350.1D2
Tapidor	BnaC02g06650.1D2	BnaA02g01360.1D2
Tapidor	BnaC02g06660.1D2	BnaA02g01370.1D2
Tapidor	BnaC02g06670.1D2	BnaA02g01380.1D2
Tapidor	BnaC02g06700.1D2	BnaA02g01440.1D2
Tapidor	BnaC02g06790.1D2	BnaA02g16940.1D2
Tapidor	BnaC02g06810.1D2	BnaA02g01510.1D2
Tapidor	BnaC02g06850.1D2	BnaA02g01550.1D2
Tapidor	BnaC02g06850.1D2	BnaA02g01560.1D2
Tapidor	BnaC02g06870.1D2	BnaA02g01570.1D2
Tapidor	BnaC02g06890.1D2	BnaA02g01590.1D2
Tapidor	BnaC02g06900.1D2	BnaA02g01600.1D2
Tapidor	BnaC02g06910.1D2	BnaA02g01610.1D2
Tapidor	BnaC02g06920.1D2	BnaA02g01620.1D2
Tapidor	BnaC02g06930.1D2	BnaA02g01630.1D2

Tapidor	BnaC02g06960.1D2	BnaA02g01660.1D2
Tapidor	BnaC02g06990.1D2	BnaA02g01690.1D2
Tapidor	BnaC02g07010.1D2	BnaA02g01710.1D2
Tapidor	BnaC02g07020.1D2	BnaA02g01710.1D2
Tapidor	BnaC02g07070.1D2	BnaA02g01750.1D2
Tapidor	BnaC02g07090.1D2	BnaA02g01770.1D2
Tapidor	BnaC02g07140.1D2	BnaA02g01820.1D2
Tapidor	BnaC02g07170.1D2	BnaA02g01860.1D2
Tapidor	BnaC02g07200.1D2	BnaA02g01890.1D2
Tapidor	BnaC02g07270.1D2	BnaA02g01950.1D2
Tapidor	BnaC02g07280.1D2	BnaA02g01960.1D2
Tapidor	BnaC02g07310.1D2	BnaA02g01990.1D2
Tapidor	BnaC02g07320.1D2	BnaA02g02000.1D2
Tapidor	BnaC02g07370.1D2	BnaA02g02020.1D2
Tapidor	BnaC02g07410.1D2	BnaA02g02070.1D2
Tapidor	BnaC02g07440.1D2	BnaA02g02090.1D2
Tapidor	BnaC02g07520.1D2	BnaA02g02100.1D2
Tapidor	BnaC02g07530.1D2	BnaA02g02110.1D2
Tapidor	BnaC02g07660.1D2	BnaA02g02190.1D2
Tapidor	BnaC02g07700.1D2	BnaA02g02230.1D2
Tapidor	BnaC02g07790.1D2	BnaA02g02320.1D2
Tapidor	BnaC02g07800.1D2	BnaA02g02340.1D2
Tapidor	BnaC02g07810.1D2	BnaA02g02350.1D2
Tapidor	BnaC02g07830.1D2	BnaA02g02370.1D2
Tapidor	BnaC02g07850.1D2	BnaA02g02390.1D2
Tapidor	BnaC02g07860.1D2	BnaA02g02400.1D2
Tapidor	BnaC02g07890.1D2	BnaA02g02470.1D2
Tapidor	BnaC02g07900.1D2	BnaA02g02480.1D2
Tapidor	BnaC02g07940.1D2	BnaA02g02520.1D2
Tapidor	BnaC02g08000.1D2	BnaA02g02550.1D2
Tapidor	BnaC02g08030.1D2	BnaA02g02560.1D2
Tapidor	BnaC02g08050.1D2	BnaA02g02580.1D2
Tapidor	BnaC02g08090.1D2	BnaA02g02620.1D2
Tapidor	BnaC02g08100.1D2	BnaA02g02630.1D2
Tapidor	BnaC02g08120.1D2	BnaA02g02650.1D2
Tapidor	BnaC02g08150.1D2	BnaA02g02680.1D2
Tapidor	BnaC02g08190.1D2	BnaA02g02720.1D2
Tapidor	BnaC02g08200.1D2	BnaA02g02730.1D2
Tapidor	BnaC02g08210.1D2	BnaA02g02740.1D2
Tapidor	BnaC02g08290.1D2	BnaA02g02820.1D2
Tapidor	BnaC02g08300.1D2	BnaA02g02830.1D2
Tapidor	BnaC02g08340.1D2	BnaA02g02870.1D2
Tapidor	BnaC02g08370.1D2	BnaA02g02900.1D2
Tapidor	BnaC02g08380.1D2	BnaA02g02910.1D2
Tapidor	BnaC02g08390.1D2	BnaA02g02920.1D2
Tapidor	BnaC02g08490.1D2	BnaA02g04250.1D2
Tapidor	BnaC02g08620.1D2	BnaA02g03040.1D2
Tapidor	BnaC02g08630.1D2	BnaA02g03050.1D2
Tapidor	BnaC02g08680.1D2	BnaA02g03090.1D2
Tapidor	BnaC02g08800.1D2	BnaA02g03210.1D2
Tapidor	BnaC02g08810.1D2	BnaA02g03220.1D2

Tapidor	BnaC02g08850.1D2	BnaA02g03260.1D2
Tapidor	BnaC02g08860.1D2	BnaA02g03270.1D2
Tapidor	BnaC02g08890.1D2	BnaA02g03300.1D2
Tapidor	BnaC02g08900.1D2	BnaA02g03310.1D2
Tapidor	BnaC02g08950.1D2	BnaA02g03350.1D2
Tapidor	BnaC02g08960.1D2	BnaA02g03360.1D2
Tapidor	BnaC02g09010.1D2	BnaA02g03380.1D2
Tapidor	BnaC02g09040.1D2	BnaA02g03380.1D2
Tapidor	BnaC02g09090.1D2	BnaA02g03430.1D2
Tapidor	BnaC02g09200.1D2	BnaA02g04400.1D2
Tapidor	BnaC02g09220.1D2	BnaA02g04420.1D2
Tapidor	BnaC02g09450.1D2	BnaA02g03720.1D2
Tapidor	BnaC02g09470.1D2	BnaA02g04790.1D2
Tapidor	BnaC02g09480.1D2	BnaA02g04800.1D2
Tapidor	BnaC02g09490.1D2	BnaA02g04780.1D2
Tapidor	BnaC02g09550.1D2	BnaA02g04800.1D2
Tapidor	BnaC02g09710.1D2	BnaA02g04940.1D2
Tapidor	BnaC02g09730.1D2	BnaA02g03910.1D2
Tapidor	BnaC02g09860.1D2	BnaA02g03780.1D2
Tapidor	BnaC02g09900.1D2	BnaA02g04970.1D2
Tapidor	BnaC02g09960.1D2	BnaA02g05020.1D2
Tapidor	BnaC02g10050.1D2	BnaA02g05100.1D2
Tapidor	BnaC02g10070.1D2	BnaA02g05130.1D2
Tapidor	BnaC02g10130.1D2	BnaA02g05160.1D2
Tapidor	BnaC02g10150.1D2	BnaA02g05180.1D2
Tapidor	BnaC02g10360.1D2	BnaA02g05420.1D2
Tapidor	BnaC02g10460.1D2	BnaA02g05510.1D2
Tapidor	BnaC02g10600.1D2	BnaA02g05640.1D2
Tapidor	BnaC02g10660.1D2	BnaA02g05690.1D2
Tapidor	BnaC02g10710.1D2	BnaA02g05740.1D2
Tapidor	BnaC02g12270.1D2	BnaA02g05320.1D2
Tapidor	BnaC02g13720.1D2	BnaA02g10260.1D2
H165	BnaC02g11090.1D2	BnaA02g07980.1D2
H165	BnaC02g11130.1D2	BnaA02g08030.1D2
H165	BnaC02g11160.1D2	BnaA02g08060.1D2
H165	BnaC02g11200.1D2	BnaA02g08090.1D2
H165	BnaC02g11460.1D2	BnaA02g08280.1D2
H165	BnaC02g11550.1D2	BnaA02g08360.1D2
H165	BnaC02g11560.1D2	BnaA02g08370.1D2
H165	BnaC02g11590.1D2	BnaA02g08380.1D2
H165	BnaC02g11730.1D2	BnaA02g08530.1D2
H165	BnaC02g11800.1D2	BnaA02g08620.1D2
H165	BnaC02g11930.1D2	BnaA02g08770.1D2
H165	BnaC02g11970.1D2	BnaA02g08790.1D2
H165	BnaC02g11990.1D2	BnaA02g08810.1D2
H165	BnaC02g12070.1D2	BnaA02g08890.1D2
H165	BnaC02g12080.1D2	BnaA02g08900.1D2
H165	BnaC02g12100.1D2	BnaA02g08920.1D2
H165	BnaC02g12110.1D2	BnaA02g08930.1D2
H165	BnaC02g12120.1D2	BnaA02g08940.1D2
H165	BnaC02g12250.1D2	BnaA02g09110.1D2

H165	BnaC02g12290.1D2	BnaA02g09120.1D2
H165	BnaC02g12440.1D2	BnaA02g09300.1D2
H165	BnaC02g12530.1D2	BnaA02g09380.1D2
H165	BnaC02g12590.1D2	BnaA02g09420.1D2
H165	BnaC02g12740.1D2	BnaA02g09520.1D2
H165	BnaC02g12750.1D2	BnaA02g09520.1D2
H165	BnaC02g12930.1D2	BnaA02g09750.1D2
H165	BnaC02g13090.1D2	BnaA02g10310.1D2
H165	BnaC02g13100.1D2	BnaA02g11230.1D2
H165	BnaC02g13280.1D2	BnaA02g09890.1D2
H165	BnaC02g13300.1D2	BnaA02g09910.1D2
H165	BnaC02g13590.1D2	BnaA02g10120.1D2
H165	BnaC02g13730.1D2	BnaA02g10270.1D2
H165	BnaC02g13790.1D2	BnaA02g10410.1D2
H165	BnaC02g13820.1D2	BnaA02g10520.1D2
H165	BnaC02g13830.1D2	BnaA02g10530.1D2
H165	BnaC02g13980.1D2	BnaA02g09280.1D2
H165	BnaC02g14050.1D2	BnaA02g13340.1D2
H165	BnaC02g14090.1D2	BnaA02g10750.1D2
H165	BnaC02g14100.1D2	BnaA02g10760.1D2
H165	BnaC02g14150.1D2	BnaA02g10810.1D2
H165	BnaC02g14210.1D2	BnaA02g11060.1D2
H165	BnaC02g14250.1D2	BnaA02g10650.1D2
H165	BnaC02g14260.1D2	BnaA02g10640.1D2
H165	BnaC02g14290.1D2	BnaA02g10890.1D2
H165	BnaC02g14310.1D2	BnaA02g10910.1D2
H165	BnaC02g14330.1D2	BnaA02g10930.1D2
H165	BnaC02g14400.1D2	BnaA02g10990.1D2
H165	BnaC02g14610.1D2	BnaA02g11370.1D2
H165	BnaC02g14620.1D2	BnaA02g11380.1D2
H165	BnaC02g14740.1D2	BnaA02g11460.1D2
H165	BnaC02g14770.1D2	BnaA02g11480.1D2
H165	BnaC02g14810.1D2	BnaA02g11500.1D2
H165	BnaC02g14830.1D2	BnaA02g11520.1D2
H165	BnaC02g14840.1D2	BnaA02g11530.1D2
H165	BnaC02g14870.1D2	BnaA02g11560.1D2
H165	BnaC02g14980.1D2	BnaA02g11590.1D2
H165	BnaC02g14990.1D2	BnaA02g11610.1D2
H165	BnaC02g15010.1D2	BnaA02g11630.1D2
H165	BnaC02g15240.1D2	BnaA02g11650.1D2
H165	BnaC02g15250.1D2	BnaA02g11660.1D2
H165	BnaC02g15290.1D2	BnaA02g11670.1D2
H165	BnaC02g15290.1D2	BnaA02g11680.1D2
H165	BnaC02g15570.1D2	BnaA02g11710.1D2
H165	BnaC02g15580.1D2	BnaA02g11720.1D2
H165	BnaC02g15610.1D2	BnaA02g11760.1D2
H165	BnaC02g15630.1D2	BnaA02g11790.1D2
H165	BnaC02g15840.1D2	BnaA02g12010.1D2
H165	BnaC02g15860.1D2	BnaA02g12030.1D2
H165	BnaC02g15880.1D2	BnaA02g12040.1D2
H165	BnaC02g15970.1D2	BnaA02g12510.1D2

H165	BnaC02g15990.1D2	BnaA02g12160.1D2
H165	BnaC02g16020.1D2	BnaA02g12530.1D2
H165	BnaC02g16070.1D2	BnaA02g12570.1D2
H165	BnaC02g16140.1D2	BnaA02g12640.1D2
H165	BnaC02g16200.1D2	BnaA02g12690.1D2
H165	BnaC02g16260.1D2	BnaA02g12770.1D2
H165	BnaC02g16280.1D2	BnaA02g12800.1D2
H165	BnaC02g16390.1D2	BnaA02g12980.1D2
H165	BnaC02g16440.1D2	BnaA02g13040.1D2
H165	BnaC02g16500.1D2	BnaA02g13070.1D2
H165	BnaC02g16560.1D2	BnaA02g13150.1D2
H165	BnaC02g16580.1D2	BnaA02g13160.1D2
H165	BnaC02g16590.1D2	BnaA02g13170.1D2
H165	BnaC02g16600.1D2	BnaA02g13180.1D2
H165	BnaC02g16610.1D2	BnaA02g13190.1D2
H165	BnaC02g16650.1D2	BnaA02g13230.1D2
H165	BnaC02g16680.1D2	BnaA02g13260.1D2
H165	BnaC02g17100.1D2	BnaA02g34780.1D2
H165	BnaC02g17120.1D2	BnaA02g34780.1D2
H165	BnaC02g17210.1D2	BnaA02g11690.1D2
H165	BnaC02g17320.1D2	BnaA02g35280.1D2
H165	BnaC02g17380.1D2	BnaA02g35260.1D2
H165	BnaC02g17550.1D2	BnaA02g13710.1D2
H165	BnaC02g17710.1D2	BnaA02g13930.1D2
H165	BnaC02g17720.1D2	BnaA02g13940.1D2
H165	BnaC02g17730.1D2	BnaA02g13930.1D2
H165	BnaC02g17730.1D2	BnaA02g13950.1D2
H165	BnaC02g17760.1D2	BnaA02g13980.1D2
H165	BnaC02g17810.1D2	BnaA02g13460.1D2
H165	BnaC02g17820.1D2	BnaA02g13450.1D2
H165	BnaC02g17830.1D2	BnaA02g13440.1D2
H165	BnaC02g17870.1D2	BnaA02g13480.1D2
H165	BnaC02g18200.1D2	BnaA02g13310.1D2
H165	BnaC02g18260.1D2	BnaA02g14040.1D2
H165	BnaC02g18300.1D2	BnaA02g14100.1D2
H165	BnaC02g18420.1D2	BnaA02g14330.1D2
H165	BnaC02g18430.1D2	BnaA02g14350.1D2
H165	BnaC02g18540.1D2	BnaA02g14380.1D2
H165	BnaC02g18550.1D2	BnaA02g14380.1D2
H165	BnaC02g18610.1D2	BnaA02g14450.1D2
H165	BnaC02g18640.1D2	BnaA02g14480.1D2
H165	BnaC02g18690.1D2	BnaA02g14590.1D2
H165	BnaC02g18830.1D2	BnaA02g14760.1D2
H165	BnaC02g18960.1D2	BnaA02g14910.1D2
H165	BnaC02g18980.1D2	BnaA02g15720.1D2
H165	BnaC02g19040.1D2	BnaA02g15660.1D2
H165	BnaC02g19080.1D2	BnaA02g14920.1D2
H165	BnaC02g19110.1D2	BnaA02g14930.1D2
H165	BnaC02g19190.1D2	BnaA02g14960.1D2
H165	BnaC02g19210.1D2	BnaA02g14990.1D2
H165	BnaC02g19240.1D2	BnaA02g15010.1D2

H165	BnaC02g19260.1D2	BnaA02g15030.1D2
H165	BnaC02g19280.1D2	BnaA02g15060.1D2
H165	BnaC02g19310.1D2	BnaA02g15080.1D2
H165	BnaC02g19320.1D2	BnaA02g15090.1D2
H165	BnaC02g19330.1D2	BnaA02g15100.1D2
H165	BnaC02g19340.1D2	BnaA02g15460.1D2
H165	BnaC02g19380.1D2	BnaA02g15510.1D2
H165	BnaC02g19420.1D2	BnaA02g15520.1D2
H165	BnaC02g19460.1D2	BnaA02g14560.1D2
H165	BnaC02g19560.1D2	BnaA02g15640.1D2
H165	BnaC02g19590.1D2	BnaA02g15620.1D2
H165	BnaC02g19600.1D2	BnaA02g15610.1D2
H165	BnaC02g19620.1D2	BnaA02g15590.1D2
H165	BnaC02g19630.1D2	BnaA02g15580.1D2
H165	BnaC02g19710.1D2	BnaA02g14670.1D2
H165	BnaC02g19740.1D2	BnaA02g14240.1D2
H165	BnaC02g19780.1D2	BnaA02g14290.1D2
H165	BnaC02g19850.1D2	BnaA02g14150.1D2
H165	BnaC02g19880.1D2	BnaA02g14170.1D2
H165	BnaC02g19890.1D2	BnaA02g14180.1D2
H165	BnaC02g19900.1D2	BnaA02g14190.1D2
H165	BnaC02g19910.1D2	BnaA02g14210.1D2
H165	BnaC02g19950.1D2	BnaA02g15200.1D2
H165	BnaC02g19960.1D2	BnaA02g15210.1D2
H165	BnaC02g19980.1D2	BnaA02g15230.1D2
H165	BnaC02g19990.1D2	BnaA02g15230.1D2
H165	BnaC02g20040.1D2	BnaA02g15280.1D2
H165	BnaC02g20120.1D2	BnaA02g15310.1D2
H165	BnaC02g20290.1D2	BnaA02g15760.1D2
H165	BnaC02g20310.1D2	BnaA02g15790.1D2
H165	BnaC02g20400.1D2	BnaA02g15890.1D2
H165	BnaC02g20420.1D2	BnaA02g15900.1D2
H165	BnaC02g20430.1D2	BnaA02g15910.1D2
H165	BnaC02g20490.1D2	BnaA02g15940.1D2
H165	BnaC02g20530.1D2	BnaA02g15960.1D2
H165	BnaC02g20560.1D2	BnaA02g15970.1D2
H165	BnaC02g20580.1D2	BnaA02g15990.1D2
H165	BnaC02g20590.1D2	BnaA02g16000.1D2
H165	BnaC02g20620.1D2	BnaA02g16020.1D2
H165	BnaC02g20630.1D2	BnaA02g16030.1D2
H165	BnaC02g20680.1D2	BnaA02g16920.1D2
H165	BnaC02g20770.1D2	BnaA02g16120.1D2
H165	BnaC02g20830.1D2	BnaA02g16160.1D2
H165	BnaC02g20890.1D2	BnaA02g16180.1D2
H165	BnaC02g20930.1D2	BnaA02g16200.1D2
H165	BnaC02g20950.1D2	BnaA02g16220.1D2
H165	BnaC02g20960.1D2	BnaA02g16230.1D2
H165	BnaC02g20970.1D2	BnaA02g16880.1D2
H165	BnaC02g21060.1D2	BnaA02g16930.1D2
H165	BnaC02g21100.1D2	BnaA02g16260.1D2
H165	BnaC02g21120.1D2	BnaA02g16280.1D2

H165	BnaC02g21130.1D2	BnaA02g16290.1D2
H165	BnaC02g21150.1D2	BnaA02g16340.1D2
H165	BnaC02g21180.1D2	BnaA02g16360.1D2
H165	BnaC02g21240.1D2	BnaA02g16410.1D2
H165	BnaC02g21300.1D2	BnaA02g16320.1D2
H165	BnaC02g21310.1D2	BnaA02g15500.1D2
H165	BnaC02g21360.1D2	BnaA02g15850.1D2
H165	BnaC02g21370.1D2	BnaA02g15840.1D2
H165	BnaC02g21400.1D2	BnaA02g15490.1D2
H165	BnaC02g21490.1D2	BnaA02g16530.1D2
H165	BnaC02g21570.1D2	BnaA02g16600.1D2
H165	BnaC02g21580.1D2	BnaA02g16610.1D2
H165	BnaC02g21590.1D2	BnaA02g16620.1D2
H165	BnaC02g21600.1D2	BnaA02g16630.1D2
H165	BnaC02g21670.1D2	BnaA02g17120.1D2
H165	BnaC02g21680.1D2	BnaA02g17110.1D2
H165	BnaC02g21730.1D2	BnaA02g16700.1D2
H165	BnaC02g21740.1D2	BnaA02g16710.1D2
H165	BnaC02g21750.1D2	BnaA02g16720.1D2
H165	BnaC02g21910.1D2	BnaA02g17150.1D2
H165	BnaC02g22020.1D2	BnaA02g17190.1D2
H165	BnaC02g22050.1D2	BnaA02g17210.1D2
H165	BnaC02g22220.1D2	BnaA02g20280.1D2
H165	BnaC02g22230.1D2	BnaA02g20270.1D2
H165	BnaC02g22240.1D2	BnaA02g20260.1D2
H165	BnaC02g22290.1D2	BnaA02g17470.1D2
H165	BnaC02g22340.1D2	BnaA02g17500.1D2
H165	BnaC02g22370.1D2	BnaA02g17520.1D2
H165	BnaC02g22470.1D2	BnaA02g15870.1D2
H165	BnaC02g22490.1D2	BnaA02g17330.1D2
H165	BnaC02g22510.1D2	BnaA02g17300.1D2
H165	BnaC02g22720.1D2	BnaA02g17370.1D2
H165	BnaC02g22750.1D2	BnaA02g17340.1D2
H165	BnaC02g22820.1D2	BnaA02g18030.1D2
H165	BnaC02g22830.1D2	BnaA02g18020.1D2
H165	BnaC02g23030.1D2	BnaA02g16780.1D2
H165	BnaC02g23040.1D2	BnaA02g16770.1D2
H165	BnaC02g23060.1D2	BnaA02g17050.1D2
H165	BnaC02g23070.1D2	BnaA02g17060.1D2
H165	BnaC02g23080.1D2	BnaA02g17070.1D2
H165	BnaC02g23100.1D2	BnaA02g17090.1D2
H165	BnaC02g23180.1D2	BnaA02g17660.1D2
H165	BnaC02g23190.1D2	BnaA02g17670.1D2
H165	BnaC02g23260.1D2	BnaA02g17750.1D2
H165	BnaC02g23270.1D2	BnaA02g17760.1D2
H165	BnaC02g23600.1D2	BnaA02g19420.1D2
H165	BnaC02g23610.1D2	BnaA02g19400.1D2
H165	BnaC02g23620.1D2	BnaA02g19390.1D2
H165	BnaC02g23630.1D2	BnaA02g21180.1D2
H165	BnaC02g23750.1D2	BnaA02g20780.1D2
H165	BnaC02g23760.1D2	BnaA02g20790.1D2

H165	BnaC02g23790.1D2	BnaA02g20820.1D2
H165	BnaC02g23800.1D2	BnaA02g20830.1D2
H165	BnaC02g23820.1D2	BnaA02g20840.1D2
H165	BnaC02g23850.1D2	BnaA02g20870.1D2
H165	BnaC02g23860.1D2	BnaA02g20880.1D2
H165	BnaC02g23870.1D2	BnaA02g20890.1D2
H165	BnaC02g23930.1D2	BnaA02g20980.1D2
H165	BnaC02g23940.1D2	BnaA02g20990.1D2
H165	BnaC02g23960.1D2	BnaA02g21010.1D2
H165	BnaC02g24130.1D2	BnaA02g22020.1D2
H165	BnaC02g24140.1D2	BnaA02g22040.1D2
H165	BnaC02g24150.1D2	BnaA02g20320.1D2
H165	BnaC02g24170.1D2	BnaA02g19240.1D2
H165	BnaC02g24210.1D2	BnaA02g20060.1D2
H165	BnaC02g24250.1D2	BnaA02g20620.1D2
H165	BnaC02g24260.1D2	BnaA02g20610.1D2
H165	BnaC02g24270.1D2	BnaA02g20600.1D2
H165	BnaC02g24370.1D2	BnaA02g18400.1D2
H165	BnaC02g24380.1D2	BnaA02g18390.1D2
H165	BnaC02g24410.1D2	BnaA02g18370.1D2
H165	BnaC02g24430.1D2	BnaA02g18340.1D2
H165	BnaC02g24440.1D2	BnaA02g18340.1D2
H165	BnaC02g24480.1D2	BnaA02g18280.1D2
H165	BnaC02g24520.1D2	BnaA02g18260.1D2
H165	BnaC02g24530.1D2	BnaA02g18250.1D2
H165	BnaC02g24900.1D2	BnaA02g18470.1D2
H165	BnaC02g24940.1D2	BnaA02g18500.1D2
H165	BnaC02g24950.1D2	BnaA02g18510.1D2
H165	BnaC02g24960.1D2	BnaA02g18520.1D2
H165	BnaC02g25020.1D2	BnaA02g18190.1D2
H165	BnaC02g25040.1D2	BnaA02g18170.1D2
H165	BnaC02g25330.1D2	BnaA02g19520.1D2
H165	BnaC02g25350.1D2	BnaA02g19540.1D2
H165	BnaC02g25370.1D2	BnaA02g19560.1D2
H165	BnaC02g25420.1D2	BnaA02g19610.1D2
H165	BnaC02g25460.1D2	BnaA02g19640.1D2
H165	BnaC02g25490.1D2	BnaA02g19660.1D2
H165	BnaC02g25560.1D2	BnaA02g19750.1D2
H165	BnaC02g25650.1D2	BnaA02g19770.1D2
H165	BnaC02g25660.1D2	BnaA02g19780.1D2
H165	BnaC02g25680.1D2	BnaA02g19810.1D2
H165	BnaC02g25690.1D2	BnaA02g19820.1D2
H165	BnaC02g25710.1D2	BnaA02g19830.1D2
H165	BnaC02g25720.1D2	BnaA02g19840.1D2
H165	BnaC02g25770.1D2	BnaA02g20460.1D2
H165	BnaC02g25800.1D2	BnaA02g19870.1D2
H165	BnaC02g25820.1D2	BnaA02g19900.1D2
H165	BnaC02g25920.1D2	BnaA02g19260.1D2
H165	BnaC02g26020.1D2	BnaA02g20370.1D2
H165	BnaC02g26110.1D2	BnaA02g17450.1D2
H165	BnaC02g26330.1D2	BnaA02g19120.1D2

H165	BnaC02g26500.1D2	BnaA02g20630.1D2
H165	BnaC02g26550.1D2	BnaA02g21940.1D2
H165	BnaC02g26570.1D2	BnaA02g21950.1D2
H165	BnaC02g26620.1D2	BnaA02g21890.1D2
H165	BnaC02g26810.1D2	BnaA02g21740.1D2
H165	BnaC02g26860.1D2	BnaA02g21690.1D2
H165	BnaC02g27270.1D2	BnaA02g19950.1D2
H165	BnaC02g27340.1D2	BnaA02g22320.1D2
H165	BnaC02g27350.1D2	BnaA02g22340.1D2
H165	BnaC02g27410.1D2	BnaA02g22360.1D2
H165	BnaC02g27590.1D2	BnaA02g21390.1D2
H165	BnaC02g27610.1D2	BnaA02g21400.1D2
H165	BnaC02g27780.1D2	BnaA02g22050.1D2
H165	BnaC02g27960.1D2	BnaA02g23830.1D2
H165	BnaC02g27970.1D2	BnaA02g23840.1D2
H165	BnaC02g28050.1D2	BnaA02g23900.1D2
H165	BnaC02g28080.1D2	BnaA02g22750.1D2
H165	BnaC02g28230.1D2	BnaA02g22850.1D2
H165	BnaC02g28240.1D2	BnaA02g22860.1D2
H165	BnaC02g28250.1D2	BnaA02g22890.1D2
H165	BnaC02g28300.1D2	BnaA02g22920.1D2
H165	BnaC02g28320.1D2	BnaA02g22960.1D2
H165	BnaC02g28530.1D2	BnaA02g23810.1D2
H165	BnaC02g28570.1D2	BnaA02g23710.1D2
H165	BnaC02g28640.1D2	BnaA02g23680.1D2
H165	BnaC02g28710.1D2	BnaA02g23940.1D2
H165	BnaC02g28910.1D2	BnaA02g23130.1D2
H165	BnaC02g28930.1D2	BnaA02g23150.1D2
H165	BnaC02g29140.1D2	BnaA02g23290.1D2
H165	BnaC02g29370.1D2	BnaA02g23410.1D2
H165	BnaC02g29410.1D2	BnaA02g23440.1D2
H165	BnaC02g29550.1D2	BnaA02g23520.1D2
H165	BnaC02g29610.1D2	BnaA02g21560.1D2
H165	BnaC02g29620.1D2	BnaA02g23020.1D2
H165	BnaC02g29740.1D2	BnaA02g22700.1D2
H165	BnaC02g29790.1D2	BnaA02g24120.1D2
H165	BnaC02g29810.1D2	BnaA02g24120.1D2
H165	BnaC02g29830.1D2	BnaA02g24100.1D2
H165	BnaC02g29890.1D2	BnaA02g20450.1D2
H165	BnaC02g30030.1D2	BnaA02g24190.1D2
H165	BnaC02g30180.1D2	BnaA02g24340.1D2
H165	BnaC02g30250.1D2	BnaA02g22230.1D2
H165	BnaC02g30400.1D2	BnaA02g24460.1D2
H165	BnaC02g30480.1D2	BnaA02g24540.1D2
H165	BnaC02g30490.1D2	BnaA02g24550.1D2
H165	BnaC02g30580.1D2	BnaA02g24660.1D2
H165	BnaC02g30610.1D2	BnaA02g24690.1D2
H165	BnaC02g30640.1D2	BnaA02g24700.1D2
H165	BnaC02g30670.1D2	BnaA02g24780.1D2
H165	BnaC02g30950.1D2	BnaA02g25210.1D2
H165	BnaC02g31090.1D2	BnaA02g21220.1D2

H165	BnaC02g31290.1D2	BnaA02g22610.1D2
H165	BnaC02g31300.1D2	BnaA02g22600.1D2
H165	BnaC02g31350.1D2	BnaA02g22570.1D2
H165	BnaC02g31440.1D2	BnaA02g24810.1D2
H165	BnaC02g31530.1D2	BnaA02g25530.1D2
H165	BnaC02g31540.1D2	BnaA02g25520.1D2
H165	BnaC02g31550.1D2	BnaA02g25510.1D2
H165	BnaC02g31690.1D2	BnaA02g24900.1D2
H165	BnaC02g31710.1D2	BnaA02g24910.1D2
H165	BnaC02g31740.1D2	BnaA02g25070.1D2
H165	BnaC02g31820.1D2	BnaA02g25000.1D2
H165	BnaC02g31860.1D2	BnaA02g24640.1D2
H165	BnaC02g31910.1D2	BnaA02g25140.1D2
H165	BnaC02g31930.1D2	BnaA02g25170.1D2
H165	BnaC02g31940.1D2	BnaA02g25180.1D2
H165	BnaC02g31950.1D2	BnaA02g25190.1D2
H165	BnaC02g31970.1D2	BnaA02g25200.1D2
H165	BnaC02g31990.1D2	BnaA02g25220.1D2
H165	BnaC02g32020.1D2	BnaA02g25260.1D2
H165	BnaC02g32030.1D2	BnaA02g25270.1D2
H165	BnaC02g32060.1D2	BnaA02g25570.1D2
H165	BnaC02g32070.1D2	BnaA02g25580.1D2
H165	BnaC02g32090.1D2	BnaA02g25600.1D2
H165	BnaC02g32100.1D2	BnaA02g25610.1D2
H165	BnaC02g32120.1D2	BnaA02g25630.1D2
H165	BnaC02g32130.1D2	BnaA02g25640.1D2
H165	BnaC02g32150.1D2	BnaA02g25660.1D2
H165	BnaC02g32310.1D2	BnaA02g26970.1D2
H165	BnaC02g32380.1D2	BnaA02g26920.1D2
H165	BnaC02g32390.1D2	BnaA02g27170.1D2
H165	BnaC02g32430.1D2	BnaA02g26900.1D2
H165	BnaC02g32450.1D2	BnaA02g26880.1D2
H165	BnaC02g32460.1D2	BnaA02g26870.1D2
H165	BnaC02g32470.1D2	BnaA02g26860.1D2
H165	BnaC02g32490.1D2	BnaA02g26840.1D2
H165	BnaC02g32510.1D2	BnaA02g26830.1D2
H165	BnaC02g32550.1D2	BnaA02g26810.1D2
H165	BnaC02g32560.1D2	BnaA02g26800.1D2
H165	BnaC02g32590.1D2	BnaA02g26780.1D2
H165	BnaC02g32600.1D2	BnaA02g26770.1D2
H165	BnaC02g32680.1D2	BnaA02g25820.1D2
H165	BnaC02g32690.1D2	BnaA02g25830.1D2
H165	BnaC02g32830.1D2	BnaA02g26740.1D2
H165	BnaC02g32840.1D2	BnaA02g26730.1D2
H165	BnaC02g32850.1D2	BnaA02g26710.1D2
H165	BnaC02g34510.1D2	BnaA02g28130.1D2
H165	BnaC02g34850.1D2	BnaA02g28220.1D2
H165	BnaC02g34860.1D2	BnaA02g28230.1D2
H165	BnaC02g34870.1D2	BnaA02g28240.1D2
H165	BnaC02g35240.1D2	BnaA02g28610.1D2
H165	BnaC02g35250.1D2	BnaA02g28620.1D2

H165	BnaC02g35260.1D2	BnaA02g28630.1D2
H165	BnaC02g35390.1D2	BnaA02g28820.1D2
H165	BnaC02g35490.1D2	BnaA02g28950.1D2
H165	BnaC02g35520.1D2	BnaA02g28980.1D2
H165	BnaC02g35640.1D2	BnaA02g29090.1D2
H165	BnaC02g35650.1D2	BnaA02g29110.1D2
H165	BnaC02g35660.1D2	BnaA02g29120.1D2
H165	BnaC02g35710.1D2	BnaA02g28000.1D2
H165	BnaC02g35810.1D2	BnaA02g29220.1D2
H165	BnaC02g35840.1D2	BnaA02g29230.1D2
H165	BnaC02g35850.1D2	BnaA02g29240.1D2
H165	BnaC02g35910.1D2	BnaA02g29300.1D2
H165	BnaC02g35920.1D2	BnaA02g29310.1D2
H165	BnaC02g35930.1D2	BnaA02g29320.1D2
H165	BnaC02g35950.1D2	BnaA02g29340.1D2
H165	BnaC02g36100.1D2	BnaA02g28380.1D2
H165	BnaC02g36440.1D2	BnaA02g29640.1D2
H165	BnaC02g36470.1D2	BnaA02g29670.1D2
H165	BnaC02g36510.1D2	BnaA02g29690.1D2
H165	BnaC02g36610.1D2	BnaA02g29760.1D2
H165	BnaC02g36620.1D2	BnaA02g29770.1D2
H165	BnaC02g36870.1D2	BnaA02g30080.1D2
H165	BnaC02g37000.1D2	BnaA02g30230.1D2
H165	BnaC02g37010.1D2	BnaA02g30230.1D2
H165	BnaC02g37020.1D2	BnaA02g30240.1D2
H165	BnaC02g37060.1D2	BnaA02g30290.1D2
H165	BnaC02g37160.1D2	BnaA02g30350.1D2
H165	BnaC02g37210.1D2	BnaA02g30440.1D2
H165	BnaC02g37270.1D2	BnaA02g30500.1D2
H165	BnaC02g37300.1D2	BnaA02g30520.1D2
H165	BnaC02g37310.1D2	BnaA02g30530.1D2
H165	BnaC02g37360.1D2	BnaA02g32250.1D2
H165	BnaC02g37420.1D2	BnaA02g32200.1D2
H165	BnaC02g37440.1D2	BnaA02g32190.1D2
H165	BnaC02g37460.1D2	BnaA02g32180.1D2
H165	BnaC02g37470.1D2	BnaA02g32170.1D2
H165	BnaC02g37500.1D2	BnaA02g29390.1D2
H165	BnaC02g37710.1D2	BnaA02g32160.1D2
H165	BnaC02g37720.1D2	BnaA02g32150.1D2
H165	BnaC02g37750.1D2	BnaA02g32140.1D2
H165	BnaC02g37880.1D2	BnaA02g30680.1D2
H165	BnaC02g37900.1D2	BnaA02g30700.1D2
H165	BnaC02g37950.1D2	BnaA02g30750.1D2
H165	BnaC02g37960.1D2	BnaA02g30760.1D2
H165	BnaC02g37980.1D2	BnaA02g30780.1D2
H165	BnaC02g38000.1D2	BnaA02g30790.1D2
H165	BnaC02g38040.1D2	BnaA02g30840.1D2
H165	BnaC02g38100.1D2	BnaA02g30940.1D2
H165	BnaC02g38180.1D2	BnaA02g31000.1D2
H165	BnaC02g38320.1D2	BnaA02g31160.1D2
H165	BnaC02g38690.1D2	BnaA02g31630.1D2

H165	BnaC02g39280.1D2	BnaA02g32300.1D2
H165	BnaC02g39370.1D2	BnaA02g32110.1D2
H165	BnaC02g39580.1D2	BnaA02g32680.1D2
H165	BnaC02g39590.1D2	BnaA02g32670.1D2
H165	BnaC02g40200.1D2	BnaA02g33440.1D2
H165	BnaC02g40230.1D2	BnaA02g33460.1D2
H165	BnaC02g40510.1D2	BnaA02g33740.1D2
H165	BnaC02g40550.1D2	BnaA02g33770.1D2
H165	BnaC02g40550.1D2	BnaA02g33780.1D2
H165	BnaC02g40560.1D2	BnaA02g33790.1D2
H165	BnaC02g40810.1D2	BnaA02g33960.1D2
H165	BnaC02g40820.1D2	BnaA02g33970.1D2
H165	BnaC02g40910.1D2	BnaA02g34120.1D2
H165	BnaC02g40920.1D2	BnaA02g34130.1D2
H165	BnaC02g40940.1D2	BnaA02g34150.1D2
H165	BnaC02g40970.1D2	BnaA02g34180.1D2
H165	BnaC02g40990.1D2	BnaA02g34190.1D2
H165	BnaC02g41120.1D2	BnaA02g34290.1D2
H165	BnaC02g41220.1D2	BnaA02g34400.1D2
H165	BnaC02g41400.1D2	BnaA02g34510.1D2
H165	BnaC02g41440.1D2	BnaA02g34880.1D2
H165	BnaC02g41770.1D2	BnaA02g34570.1D2
R53	BnaC02g06110.1D2	BnaA02g04040.1D2
R53	BnaC02g06150.1D2	BnaA02g04000.1D2
R53	BnaC02g06190.1D2	BnaA02g03950.1D2
R53	BnaC02g06200.1D2	BnaA02g03940.1D2
R53	BnaC02g06220.1D2	BnaA02g04220.1D2
R53	BnaC02g06260.1D2	BnaA02g04290.1D2
R53	BnaC02g06280.1D2	BnaA02g04310.1D2
R53	BnaC02g06300.1D2	BnaA02g04170.1D2
R53	BnaC02g06330.1D2	BnaA02g04060.1D2
R53	BnaC02g06400.1D2	BnaA02g01080.1D2
R53	BnaC02g06440.1D2	BnaA02g01120.1D2
R53	BnaC02g06440.1D2	BnaA02g01140.1D2
R53	BnaC02g06450.1D2	BnaA02g01150.1D2
R53	BnaC02g06470.1D2	BnaA02g01190.1D2
R53	BnaC02g06530.1D2	BnaA02g01250.1D2
R53	BnaC02g06550.1D2	BnaA02g01270.1D2
R53	BnaC02g06560.1D2	BnaA02g01290.1D2
R53	BnaC02g06570.1D2	BnaA02g01300.1D2
R53	BnaC02g06580.1D2	BnaA02g01300.1D2
R53	BnaC02g06590.1D2	BnaA02g01300.1D2
R53	BnaC02g06640.1D2	BnaA02g01350.1D2
R53	BnaC02g06650.1D2	BnaA02g01360.1D2
R53	BnaC02g06780.1D2	BnaA02g01490.1D2
R53	BnaC02g06790.1D2	BnaA02g16940.1D2
R53	BnaC02g06810.1D2	BnaA02g01510.1D2
R53	BnaC02g06830.1D2	BnaA02g01530.1D2
R53	BnaC02g06850.1D2	BnaA02g01550.1D2
R53	BnaC02g06850.1D2	BnaA02g01560.1D2
R53	BnaC02g06870.1D2	BnaA02g01570.1D2

R53	BnaC02g06890.1D2	BnaA02g01590.1D2
R53	BnaC02g06900.1D2	BnaA02g01600.1D2
R53	BnaC02g06910.1D2	BnaA02g01610.1D2
R53	BnaC02g06920.1D2	BnaA02g01620.1D2
R53	BnaC02g06930.1D2	BnaA02g01630.1D2
R53	BnaC02g06960.1D2	BnaA02g01660.1D2
R53	BnaC02g06990.1D2	BnaA02g01690.1D2
R53	BnaC02g07020.1D2	BnaA02g01710.1D2
R53	BnaC02g07070.1D2	BnaA02g01750.1D2
R53	BnaC02g07090.1D2	BnaA02g01770.1D2
R53	BnaC02g07140.1D2	BnaA02g01820.1D2
R53	BnaC02g07170.1D2	BnaA02g01860.1D2
R53	BnaC02g07180.1D2	BnaA02g01870.1D2
R53	BnaC02g07200.1D2	BnaA02g01890.1D2
R53	BnaC02g07270.1D2	BnaA02g01950.1D2
R53	BnaC02g07280.1D2	BnaA02g01960.1D2
R53	BnaC02g07290.1D2	BnaA02g01970.1D2
R53	BnaC02g07310.1D2	BnaA02g01990.1D2
R53	BnaC02g07320.1D2	BnaA02g02000.1D2
R53	BnaC02g07370.1D2	BnaA02g02020.1D2
R53	BnaC02g07410.1D2	BnaA02g02070.1D2
R53	BnaC02g07440.1D2	BnaA02g02090.1D2
R53	BnaC02g07520.1D2	BnaA02g02100.1D2
R53	BnaC02g07660.1D2	BnaA02g02190.1D2
R53	BnaC02g07700.1D2	BnaA02g02230.1D2
R53	BnaC02g07780.1D2	BnaA02g02310.1D2
R53	BnaC02g07790.1D2	BnaA02g02320.1D2
R53	BnaC02g07800.1D2	BnaA02g02340.1D2
R53	BnaC02g07810.1D2	BnaA02g02350.1D2
R53	BnaC02g07830.1D2	BnaA02g02370.1D2
R53	BnaC02g07850.1D2	BnaA02g02390.1D2
R53	BnaC02g07860.1D2	BnaA02g02400.1D2
R53	BnaC02g07890.1D2	BnaA02g02470.1D2
R53	BnaC02g07900.1D2	BnaA02g02480.1D2
R53	BnaC02g07940.1D2	BnaA02g02520.1D2
R53	BnaC02g07980.1D2	BnaA02g02530.1D2
R53	BnaC02g08000.1D2	BnaA02g02550.1D2
R53	BnaC02g08020.1D2	BnaA02g02560.1D2
R53	BnaC02g08030.1D2	BnaA02g02560.1D2
R53	BnaC02g08050.1D2	BnaA02g02580.1D2
R53	BnaC02g08090.1D2	BnaA02g02620.1D2
R53	BnaC02g08100.1D2	BnaA02g02630.1D2
R53	BnaC02g08120.1D2	BnaA02g02650.1D2
R53	BnaC02g08140.1D2	BnaA02g02670.1D2
R53	BnaC02g08150.1D2	BnaA02g02680.1D2
R53	BnaC02g08190.1D2	BnaA02g02720.1D2
R53	BnaC02g08210.1D2	BnaA02g02740.1D2
R53	BnaC02g08250.1D2	BnaA02g02780.1D2
R53	BnaC02g08290.1D2	BnaA02g02820.1D2
R53	BnaC02g08300.1D2	BnaA02g02830.1D2
R53	BnaC02g08340.1D2	BnaA02g02870.1D2

R53	BnaC02g08370.1D2	BnaA02g02900.1D2
R53	BnaC02g08380.1D2	BnaA02g02910.1D2
R53	BnaC02g08390.1D2	BnaA02g02920.1D2
R53	BnaC02g08490.1D2	BnaA02g04250.1D2
R53	BnaC02g08530.1D2	BnaA02g03920.1D2
R53	BnaC02g08560.1D2	BnaA02g03930.1D2
R53	BnaC02g08580.1D2	BnaA02g02990.1D2
R53	BnaC02g08600.1D2	BnaA02g03020.1D2
R53	BnaC02g08620.1D2	BnaA02g03040.1D2
R53	BnaC02g08630.1D2	BnaA02g03050.1D2
R53	BnaC02g08650.1D2	BnaA02g03070.1D2
R53	BnaC02g08680.1D2	BnaA02g03090.1D2
R53	BnaC02g08810.1D2	BnaA02g03220.1D2
R53	BnaC02g08830.1D2	BnaA02g03240.1D2
R53	BnaC02g08850.1D2	BnaA02g03260.1D2
R53	BnaC02g08860.1D2	BnaA02g03270.1D2
R53	BnaC02g08880.1D2	BnaA02g03290.1D2
R53	BnaC02g08890.1D2	BnaA02g03300.1D2
R53	BnaC02g08900.1D2	BnaA02g03310.1D2
R53	BnaC02g08950.1D2	BnaA02g03350.1D2
R53	BnaC02g08960.1D2	BnaA02g03360.1D2
R53	BnaC02g09010.1D2	BnaA02g03380.1D2
R53	BnaC02g09040.1D2	BnaA02g03380.1D2
R53	BnaC02g09050.1D2	BnaA02g03390.1D2
R53	BnaC02g09090.1D2	BnaA02g03430.1D2
R53	BnaC02g09110.1D2	BnaA02g03450.1D2
R53	BnaC02g09130.1D2	BnaA02g03460.1D2
R53	BnaC02g09150.1D2	BnaA02g03480.1D2
R53	BnaC02g09160.1D2	BnaA02g03490.1D2
R53	BnaC02g09170.1D2	BnaA02g03500.1D2
R53	BnaC02g09220.1D2	BnaA02g04420.1D2
R53	BnaC02g09260.1D2	BnaA02g04460.1D2
R53	BnaC02g09310.1D2	BnaA02g03630.1D2
R53	BnaC02g09420.1D2	BnaA02g03690.1D2
R53	BnaC02g09430.1D2	BnaA02g03700.1D2
R53	BnaC02g09450.1D2	BnaA02g03720.1D2
R53	BnaC02g09470.1D2	BnaA02g04790.1D2
R53	BnaC02g09480.1D2	BnaA02g04800.1D2
R53	BnaC02g09500.1D2	BnaA02g04790.1D2
R53	BnaC02g09510.1D2	BnaA02g04790.1D2
R53	BnaC02g09530.1D2	BnaA02g04810.1D2
R53	BnaC02g09550.1D2	BnaA02g04800.1D2
R53	BnaC02g09570.1D2	BnaA02g04830.1D2
R53	BnaC02g09650.1D2	BnaA02g04910.1D2
R53	BnaC02g09710.1D2	BnaA02g04940.1D2
R53	BnaC02g09730.1D2	BnaA02g03910.1D2
R53	BnaC02g09860.1D2	BnaA02g03780.1D2
R53	BnaC02g09900.1D2	BnaA02g04970.1D2
R53	BnaC02g09960.1D2	BnaA02g05020.1D2
R53	BnaC02g10010.1D2	BnaA02g05060.1D2
R53	BnaC02g10050.1D2	BnaA02g05100.1D2

R53	BnaC02g10070.1D2	BnaA02g05130.1D2
R53	BnaC02g10130.1D2	BnaA02g05160.1D2
R53	BnaC02g10150.1D2	BnaA02g05180.1D2
R53	BnaC02g10170.1D2	BnaA02g05210.1D2
R53	BnaC02g10260.1D2	BnaA02g05360.1D2
R53	BnaC02g10270.1D2	BnaA02g05370.1D2
R53	BnaC02g10310.1D2	BnaA02g05400.1D2
R53	BnaC02g10320.1D2	BnaA02g05410.1D2
R53	BnaC02g10360.1D2	BnaA02g05420.1D2
R53	BnaC02g10460.1D2	BnaA02g05510.1D2
R53	BnaC02g10470.1D2	BnaA02g05520.1D2
R53	BnaC02g10490.1D2	BnaA02g05540.1D2
R53	BnaC02g10500.1D2	BnaA02g05570.1D2
R53	BnaC02g10560.1D2	BnaA02g05590.1D2
R53	BnaC02g10560.1D2	BnaA02g05600.1D2
R53	BnaC02g10570.1D2	BnaA02g05610.1D2
R53	BnaC02g10580.1D2	BnaA02g05620.1D2
R53	BnaC02g10600.1D2	BnaA02g05640.1D2
R53	BnaC02g10630.1D2	BnaA02g05670.1D2
R53	BnaC02g10660.1D2	BnaA02g05690.1D2
R53	BnaC02g10690.1D2	BnaA02g05720.1D2
R53	BnaC02g10700.1D2	BnaA02g05730.1D2
R53	BnaC02g10710.1D2	BnaA02g05740.1D2
R53	BnaC02g10770.1D2	BnaA02g06670.1D2
R53	BnaC02g10780.1D2	BnaA02g06680.1D2
R53	BnaC02g10800.1D2	BnaA02g06700.1D2
R53	BnaC02g10810.1D2	BnaA02g06710.1D2
R53	BnaC02g10820.1D2	BnaA02g06720.1D2
R53	BnaC02g10850.1D2	BnaA02g06730.1D2
R53	BnaC02g10860.1D2	BnaA02g06740.1D2
R53	BnaC02g10870.1D2	BnaA02g06750.1D2
R53	BnaC02g10920.1D2	BnaA02g06820.1D2
R53	BnaC02g10930.1D2	BnaA02g06830.1D2
R53	BnaC02g10960.1D2	BnaA02g04330.1D2
R53	BnaC02g11060.1D2	BnaA02g07950.1D2
R53	BnaC02g11070.1D2	BnaA02g07960.1D2
R53	BnaC02g11090.1D2	BnaA02g07980.1D2
R53	BnaC02g11130.1D2	BnaA02g08030.1D2
R53	BnaC02g11160.1D2	BnaA02g08060.1D2
R53	BnaC02g11200.1D2	BnaA02g08090.1D2
R53	BnaC02g11460.1D2	BnaA02g08280.1D2
R53	BnaC02g11490.1D2	BnaA02g08310.1D2
R53	BnaC02g11540.1D2	BnaA02g08350.1D2
R53	BnaC02g11560.1D2	BnaA02g08370.1D2
R53	BnaC02g11590.1D2	BnaA02g08380.1D2
R53	BnaC02g11730.1D2	BnaA02g08530.1D2
R53	BnaC02g11800.1D2	BnaA02g08620.1D2
R53	BnaC02g11820.1D2	BnaA02g08650.1D2
R53	BnaC02g11840.1D2	BnaA02g08670.1D2
R53	BnaC02g11870.1D2	BnaA02g08710.1D2
R53	BnaC02g11880.1D2	BnaA02g08720.1D2

R53	BnaC02g11930.1D2	BnaA02g08770.1D2
R53	BnaC02g11970.1D2	BnaA02g08790.1D2
R53	BnaC02g11990.1D2	BnaA02g08810.1D2
R53	BnaC02g12070.1D2	BnaA02g08890.1D2
R53	BnaC02g12080.1D2	BnaA02g08900.1D2
R53	BnaC02g12100.1D2	BnaA02g08920.1D2
R53	BnaC02g12120.1D2	BnaA02g08940.1D2
R53	BnaC02g12250.1D2	BnaA02g09110.1D2
R53	BnaC02g12270.1D2	BnaA02g05320.1D2
R53	BnaC02g12290.1D2	BnaA02g09120.1D2
R53	BnaC02g12440.1D2	BnaA02g09300.1D2
R53	BnaC02g12450.1D2	BnaA02g09310.1D2
R53	BnaC02g12480.1D2	BnaA02g09340.1D2
R53	BnaC02g12530.1D2	BnaA02g09380.1D2
R53	BnaC02g12590.1D2	BnaA02g09420.1D2
R53	BnaC02g12750.1D2	BnaA02g09520.1D2
R53	BnaC02g13100.1D2	BnaA02g11230.1D2
R53	BnaC02g13280.1D2	BnaA02g09890.1D2
R53	BnaC02g13300.1D2	BnaA02g09910.1D2
R53	BnaC02g13580.1D2	BnaA02g10110.1D2
R53	BnaC02g13610.1D2	BnaA02g10140.1D2
R53	BnaC02g13630.1D2	BnaA02g10160.1D2
R53	BnaC02g13650.1D2	BnaA02g10180.1D2
R53	BnaC02g13810.1D2	BnaA02g10510.1D2
R53	BnaC02g13820.1D2	BnaA02g10520.1D2
R53	BnaC02g13870.1D2	BnaA02g10560.1D2
R53	BnaC02g14260.1D2	BnaA02g10640.1D2
R53	BnaC02g15160.1D2	BnaA02g06910.1D2
R53	BnaC02g15180.1D2	BnaA02g06890.1D2
R53	BnaC02g15330.1D2	BnaA02g07010.1D2
R53	BnaC02g15360.1D2	BnaA02g10710.1D2
R53	BnaC02g15460.1D2	BnaA02g09670.1D2
R53	BnaC02g17080.1D2	BnaA02g34760.1D2
R53	BnaC02g17100.1D2	BnaA02g34780.1D2
R53	BnaC02g17120.1D2	BnaA02g34780.1D2
R53	BnaC02g17290.1D2	BnaA02g34650.1D2
R53	BnaC02g17320.1D2	BnaA02g35280.1D2
R53	BnaC02g17380.1D2	BnaA02g35260.1D2
R53	BnaC02g17390.1D2	BnaA02g35250.1D2
R53	BnaC02g17450.1D2	BnaA02g35210.1D2
R53	BnaC02g18540.1D2	BnaA02g14380.1D2
R53	BnaC02g18550.1D2	BnaA02g14380.1D2
R53	BnaC02g18610.1D2	BnaA02g14450.1D2
R53	BnaC02g18640.1D2	BnaA02g14480.1D2
R53	BnaC02g18690.1D2	BnaA02g14590.1D2
R53	BnaC02g18870.1D2	BnaA02g14820.1D2
R53	BnaC02g19030.1D2	BnaA02g15670.1D2
R53	BnaC02g19040.1D2	BnaA02g15660.1D2
R53	BnaC02g19200.1D2	BnaA02g14980.1D2
R53	BnaC02g19210.1D2	BnaA02g14990.1D2
R53	BnaC02g19220.1D2	BnaA02g14990.1D2

R53	BnaC02g19240.1D2	BnaA02g15010.1D2
R53	BnaC02g19430.1D2	BnaA02g15530.1D2
R53	BnaC02g19450.1D2	BnaA02g14830.1D2
R53	BnaC02g19460.1D2	BnaA02g14560.1D2
R53	BnaC02g19620.1D2	BnaA02g15590.1D2
R53	BnaC02g19630.1D2	BnaA02g15580.1D2
R53	BnaC02g19710.1D2	BnaA02g14670.1D2
R53	BnaC02g19820.1D2	BnaA02g14270.1D2
R53	BnaC02g19990.1D2	BnaA02g15230.1D2
R53	BnaC02g20040.1D2	BnaA02g15280.1D2
R53	BnaC02g20120.1D2	BnaA02g15310.1D2
R53	BnaC02g20310.1D2	BnaA02g15790.1D2
R53	BnaC02g20430.1D2	BnaA02g15910.1D2
R53	BnaC02g20490.1D2	BnaA02g15940.1D2
R53	BnaC02g20530.1D2	BnaA02g15960.1D2
R53	BnaC02g20560.1D2	BnaA02g15970.1D2
R53	BnaC02g20580.1D2	BnaA02g15990.1D2
R53	BnaC02g20620.1D2	BnaA02g16020.1D2
R53	BnaC02g20830.1D2	BnaA02g16160.1D2
R53	BnaC02g20890.1D2	BnaA02g16180.1D2
R53	BnaC02g20960.1D2	BnaA02g16230.1D2
R53	BnaC02g20990.1D2	BnaA02g16830.1D2
R53	BnaC02g21120.1D2	BnaA02g16280.1D2
R53	BnaC02g21130.1D2	BnaA02g16290.1D2
R53	BnaC02g21150.1D2	BnaA02g16340.1D2
R53	BnaC02g21180.1D2	BnaA02g16360.1D2
R53	BnaC02g21280.1D2	BnaA02g16300.1D2
R53	BnaC02g21300.1D2	BnaA02g16320.1D2
R53	BnaC02g21310.1D2	BnaA02g15500.1D2
R53	BnaC02g21320.1D2	BnaA02g15500.1D2
R53	BnaC02g21350.1D2	BnaA02g15860.1D2
R53	BnaC02g21360.1D2	BnaA02g15850.1D2
R53	BnaC02g21400.1D2	BnaA02g15490.1D2
R53	BnaC02g21490.1D2	BnaA02g16530.1D2
R53	BnaC02g21510.1D2	BnaA02g16540.1D2
R53	BnaC02g21550.1D2	BnaA02g16580.1D2
R53	BnaC02g21570.1D2	BnaA02g16600.1D2
R53	BnaC02g21600.1D2	BnaA02g16630.1D2
R53	BnaC02g21680.1D2	BnaA02g17110.1D2
R53	BnaC02g21730.1D2	BnaA02g16700.1D2
R53	BnaC02g21740.1D2	BnaA02g16710.1D2
R53	BnaC02g21790.1D2	BnaA02g16750.1D2
R53	BnaC02g21900.1D2	BnaA02g17140.1D2
R53	BnaC02g22000.1D2	BnaA02g17170.1D2
R53	BnaC02g22020.1D2	BnaA02g17190.1D2
R53	BnaC02g22220.1D2	BnaA02g20280.1D2
R53	BnaC02g22230.1D2	BnaA02g20270.1D2
R53	BnaC02g22240.1D2	BnaA02g20260.1D2
R53	BnaC02g22300.1D2	BnaA02g17480.1D2
R53	BnaC02g22370.1D2	BnaA02g17520.1D2
R53	BnaC02g22400.1D2	BnaA02g17540.1D2

R53	BnaC02g22470.1D2	BnaA02g15870.1D2
R53	BnaC02g22490.1D2	BnaA02g17330.1D2
R53	BnaC02g22540.1D2	BnaA02g16800.1D2
R53	BnaC02g22670.1D2	BnaA02g25790.1D2
R53	BnaC02g22690.1D2	BnaA02g25800.1D2
R53	BnaC02g22720.1D2	BnaA02g17370.1D2
R53	BnaC02g22750.1D2	BnaA02g17340.1D2
R53	BnaC02g22820.1D2	BnaA02g18030.1D2
R53	BnaC02g22830.1D2	BnaA02g18020.1D2
R53	BnaC02g22840.1D2	BnaA02g18010.1D2
R53	BnaC02g22870.1D2	BnaA02g17990.1D2
R53	BnaC02g23030.1D2	BnaA02g16780.1D2
R53	BnaC02g23080.1D2	BnaA02g17070.1D2
R53	BnaC02g23110.1D2	BnaA02g17100.1D2
R53	BnaC02g23200.1D2	BnaA02g17680.1D2
R53	BnaC02g23250.1D2	BnaA02g21200.1D2
R53	BnaC02g23260.1D2	BnaA02g17750.1D2
R53	BnaC02g23270.1D2	BnaA02g17760.1D2
R53	BnaC02g23310.1D2	BnaA02g17790.1D2
R53	BnaC02g23340.1D2	BnaA02g17810.1D2
R53	BnaC02g23390.1D2	BnaA02g17830.1D2
R53	BnaC02g23440.1D2	BnaA02g17870.1D2
R53	BnaC02g23600.1D2	BnaA02g19420.1D2
R53	BnaC02g23610.1D2	BnaA02g19400.1D2
R53	BnaC02g23620.1D2	BnaA02g19390.1D2
R53	BnaC02g23750.1D2	BnaA02g20780.1D2
R53	BnaC02g23820.1D2	BnaA02g20840.1D2
R53	BnaC02g23840.1D2	BnaA02g20860.1D2
R53	BnaC02g23850.1D2	BnaA02g20870.1D2
R53	BnaC02g23860.1D2	BnaA02g20880.1D2
R53	BnaC02g23870.1D2	BnaA02g20890.1D2
R53	BnaC02g23930.1D2	BnaA02g20980.1D2
R53	BnaC02g23940.1D2	BnaA02g20990.1D2
R53	BnaC02g23960.1D2	BnaA02g21010.1D2
R53	BnaC02g23980.1D2	BnaA02g21030.1D2
R53	BnaC02g24000.1D2	BnaA02g20340.1D2
R53	BnaC02g24080.1D2	BnaA02g18670.1D2
R53	BnaC02g24140.1D2	BnaA02g22040.1D2
R53	BnaC02g24150.1D2	BnaA02g20320.1D2
R53	BnaC02g24170.1D2	BnaA02g19240.1D2
R53	BnaC02g24180.1D2	BnaA02g19230.1D2
R53	BnaC02g24210.1D2	BnaA02g20060.1D2
R53	BnaC02g24290.1D2	BnaA02g20570.1D2
R53	BnaC02g24320.1D2	BnaA02g18460.1D2
R53	BnaC02g24340.1D2	BnaA02g18430.1D2
R53	BnaC02g24360.1D2	BnaA02g18410.1D2
R53	BnaC02g24370.1D2	BnaA02g18400.1D2
R53	BnaC02g24390.1D2	BnaA02g18380.1D2
R53	BnaC02g24410.1D2	BnaA02g18370.1D2
R53	BnaC02g24430.1D2	BnaA02g18340.1D2
R53	BnaC02g24440.1D2	BnaA02g18340.1D2

R53	BnaC02g24480.1D2	BnaA02g18280.1D2
R53	BnaC02g24520.1D2	BnaA02g18260.1D2
R53	BnaC02g24530.1D2	BnaA02g18250.1D2
R53	BnaC02g24570.1D2	BnaA02g18240.1D2
R53	BnaC02g24610.1D2	BnaA02g18230.1D2
R53	BnaC02g24660.1D2	BnaA02g18590.1D2
R53	BnaC02g24830.1D2	BnaA02g18800.1D2
R53	BnaC02g24860.1D2	BnaA02g18850.1D2
R53	BnaC02g24870.1D2	BnaA02g18850.1D2
R53	BnaC02g24890.1D2	BnaA02g18870.1D2
R53	BnaC02g24900.1D2	BnaA02g18470.1D2
R53	BnaC02g24910.1D2	BnaA02g18480.1D2
R53	BnaC02g24940.1D2	BnaA02g18500.1D2
R53	BnaC02g24950.1D2	BnaA02g18510.1D2
R53	BnaC02g24960.1D2	BnaA02g18520.1D2
R53	BnaC02g25010.1D2	BnaA02g18200.1D2
R53	BnaC02g25020.1D2	BnaA02g18190.1D2
R53	BnaC02g25040.1D2	BnaA02g18170.1D2
R53	BnaC02g25070.1D2	BnaA02g18160.1D2
R53	BnaC02g25080.1D2	BnaA02g18150.1D2
R53	BnaC02g25180.1D2	BnaA02g20500.1D2
R53	BnaC02g25240.1D2	BnaA02g19470.1D2
R53	BnaC02g25260.1D2	BnaA02g19480.1D2
R53	BnaC02g25280.1D2	BnaA02g19490.1D2
R53	BnaC02g25330.1D2	BnaA02g19520.1D2
R53	BnaC02g25370.1D2	BnaA02g19560.1D2
R53	BnaC02g25380.1D2	BnaA02g19580.1D2
R53	BnaC02g25420.1D2	BnaA02g19610.1D2
R53	BnaC02g25460.1D2	BnaA02g19640.1D2
R53	BnaC02g25490.1D2	BnaA02g19660.1D2
R53	BnaC02g25550.1D2	BnaA02g19740.1D2
R53	BnaC02g25560.1D2	BnaA02g19750.1D2
R53	BnaC02g25650.1D2	BnaA02g19770.1D2
R53	BnaC02g25660.1D2	BnaA02g19780.1D2
R53	BnaC02g25670.1D2	BnaA02g19790.1D2
R53	BnaC02g25680.1D2	BnaA02g19810.1D2
R53	BnaC02g25690.1D2	BnaA02g19820.1D2
R53	BnaC02g25710.1D2	BnaA02g19830.1D2
R53	BnaC02g25720.1D2	BnaA02g19840.1D2
R53	BnaC02g25730.1D2	BnaA02g19850.1D2
R53	BnaC02g25770.1D2	BnaA02g20460.1D2
R53	BnaC02g25800.1D2	BnaA02g19870.1D2
R53	BnaC02g25820.1D2	BnaA02g19900.1D2
R53	BnaC02g25830.1D2	BnaA02g19910.1D2
R53	BnaC02g25880.1D2	BnaA02g21190.1D2
R53	BnaC02g25920.1D2	BnaA02g19260.1D2
R53	BnaC02g26020.1D2	BnaA02g20370.1D2
R53	BnaC02g26040.1D2	BnaA02g20390.1D2
R53	BnaC02g26080.1D2	BnaA02g20420.1D2
R53	BnaC02g26100.1D2	BnaA02g20440.1D2
R53	BnaC02g26110.1D2	BnaA02g17450.1D2

R53	BnaC02g26250.1D2	BnaA02g18930.1D2
R53	BnaC02g26260.1D2	BnaA02g18940.1D2
R53	BnaC02g26320.1D2	BnaA02g19130.1D2
R53	BnaC02g26330.1D2	BnaA02g19120.1D2
R53	BnaC02g26340.1D2	BnaA02g19110.1D2
R53	BnaC02g26350.1D2	BnaA02g19090.1D2
R53	BnaC02g26380.1D2	BnaA02g19080.1D2
R53	BnaC02g26400.1D2	BnaA02g19060.1D2
R53	BnaC02g26410.1D2	BnaA02g19050.1D2
R53	BnaC02g26470.1D2	BnaA02g19030.1D2
R53	BnaC02g26520.1D2	BnaA02g20640.1D2
R53	BnaC02g26540.1D2	BnaA02g21910.1D2
R53	BnaC02g26550.1D2	BnaA02g21940.1D2
R53	BnaC02g26570.1D2	BnaA02g21950.1D2
R53	BnaC02g26620.1D2	BnaA02g21890.1D2
R53	BnaC02g26630.1D2	BnaA02g21880.1D2
R53	BnaC02g26810.1D2	BnaA02g21740.1D2
R53	BnaC02g26860.1D2	BnaA02g21690.1D2
R53	BnaC02g27070.1D2	BnaA02g21640.1D2
R53	BnaC02g27100.1D2	BnaA02g21660.1D2
R53	BnaC02g27170.1D2	BnaA02g21480.1D2
R53	BnaC02g27220.1D2	BnaA02g22260.1D2
R53	BnaC02g27270.1D2	BnaA02g19950.1D2
R53	BnaC02g27340.1D2	BnaA02g22320.1D2
R53	BnaC02g27460.1D2	BnaA02g21320.1D2
R53	BnaC02g27490.1D2	BnaA02g21330.1D2
R53	BnaC02g27610.1D2	BnaA02g21400.1D2
R53	BnaC02g27620.1D2	BnaA02g21410.1D2
R53	BnaC02g27630.1D2	BnaA02g21420.1D2
R53	BnaC02g27640.1D2	BnaA02g21430.1D2
R53	BnaC02g27670.1D2	BnaA02g25770.1D2
R53	BnaC02g27690.1D2	BnaA02g21460.1D2
R53	BnaC02g27720.1D2	BnaA02g22240.1D2
R53	BnaC02g27730.1D2	BnaA02g22250.1D2
R53	BnaC02g27780.1D2	BnaA02g22050.1D2
R53	BnaC02g27880.1D2	BnaA02g22150.1D2
R53	BnaC02g27960.1D2	BnaA02g23830.1D2
R53	BnaC02g27970.1D2	BnaA02g23840.1D2
R53	BnaC02g27980.1D2	BnaA02g23850.1D2
R53	BnaC02g28010.1D2	BnaA02g23860.1D2
R53	BnaC02g28010.1D2	BnaA02g23870.1D2
R53	BnaC02g28020.1D2	BnaA02g23880.1D2
R53	BnaC02g28050.1D2	BnaA02g23900.1D2
R53	BnaC02g28080.1D2	BnaA02g22750.1D2
R53	BnaC02g28130.1D2	BnaA02g22800.1D2
R53	BnaC02g28150.1D2	BnaA02g22810.1D2
R53	BnaC02g28230.1D2	BnaA02g22850.1D2
R53	BnaC02g28240.1D2	BnaA02g22860.1D2
R53	BnaC02g28250.1D2	BnaA02g22890.1D2
R53	BnaC02g28280.1D2	BnaA02g22910.1D2
R53	BnaC02g28290.1D2	BnaA02g22920.1D2

R53	BnaC02g28300.1D2	BnaA02g22920.1D2
R53	BnaC02g28320.1D2	BnaA02g22960.1D2
R53	BnaC02g28340.1D2	BnaA02g23040.1D2
R53	BnaC02g28530.1D2	BnaA02g23810.1D2
R53	BnaC02g28560.1D2	BnaA02g23720.1D2
R53	BnaC02g28570.1D2	BnaA02g23710.1D2
R53	BnaC02g28640.1D2	BnaA02g23680.1D2
R53	BnaC02g28710.1D2	BnaA02g23940.1D2
R53	BnaC02g28920.1D2	BnaA02g23140.1D2
R53	BnaC02g28930.1D2	BnaA02g23150.1D2
R53	BnaC02g29140.1D2	BnaA02g23290.1D2
R53	BnaC02g29320.1D2	BnaA02g23370.1D2
R53	BnaC02g29370.1D2	BnaA02g23410.1D2
R53	BnaC02g29410.1D2	BnaA02g23440.1D2
R53	BnaC02g29430.1D2	BnaA02g23490.1D2
R53	BnaC02g29550.1D2	BnaA02g23520.1D2
R53	BnaC02g29620.1D2	BnaA02g23020.1D2
R53	BnaC02g29650.1D2	BnaA02g23070.1D2
R53	BnaC02g29730.1D2	BnaA02g24210.1D2
R53	BnaC02g29740.1D2	BnaA02g22700.1D2
R53	BnaC02g29810.1D2	BnaA02g24120.1D2
R53	BnaC02g29830.1D2	BnaA02g24100.1D2
R53	BnaC02g29860.1D2	BnaA02g24090.1D2
R53	BnaC02g29890.1D2	BnaA02g20450.1D2
R53	BnaC02g30030.1D2	BnaA02g24190.1D2
R53	BnaC02g30140.1D2	BnaA02g23030.1D2
R53	BnaC02g30180.1D2	BnaA02g24340.1D2
R53	BnaC02g30250.1D2	BnaA02g22230.1D2
R53	BnaC02g30350.1D2	BnaA02g24400.1D2
R53	BnaC02g30400.1D2	BnaA02g24460.1D2
R53	BnaC02g30410.1D2	BnaA02g24480.1D2
R53	BnaC02g30420.1D2	BnaA02g24500.1D2
R53	BnaC02g30480.1D2	BnaA02g24540.1D2
R53	BnaC02g30490.1D2	BnaA02g24550.1D2
R53	BnaC02g30560.1D2	BnaA02g24570.1D2
R53	BnaC02g30580.1D2	BnaA02g24660.1D2
R53	BnaC02g30610.1D2	BnaA02g24690.1D2
R53	BnaC02g30640.1D2	BnaA02g24700.1D2
R53	BnaC02g30670.1D2	BnaA02g24780.1D2
R53	BnaC02g30950.1D2	BnaA02g25210.1D2
R53	BnaC02g31090.1D2	BnaA02g21220.1D2
R53	BnaC02g31110.1D2	BnaA02g21230.1D2
R53	BnaC02g31130.1D2	BnaA02g21250.1D2
R53	BnaC02g31290.1D2	BnaA02g22610.1D2
R53	BnaC02g31300.1D2	BnaA02g22600.1D2
R53	BnaC02g31350.1D2	BnaA02g22570.1D2
R53	BnaC02g31370.1D2	BnaA02g26570.1D2
R53	BnaC02g31380.1D2	BnaA02g25020.1D2
R53	BnaC02g31440.1D2	BnaA02g24810.1D2
R53	BnaC02g31690.1D2	BnaA02g24900.1D2
R53	BnaC02g31710.1D2	BnaA02g24910.1D2

R53	BnaC02g31820.1D2	BnaA02g25000.1D2
R53	BnaC02g31860.1D2	BnaA02g24640.1D2
R53	BnaC02g31910.1D2	BnaA02g25140.1D2
R53	BnaC02g31930.1D2	BnaA02g25170.1D2
R53	BnaC02g31940.1D2	BnaA02g25180.1D2
R53	BnaC02g31950.1D2	BnaA02g25190.1D2
R53	BnaC02g31970.1D2	BnaA02g25200.1D2
R53	BnaC02g31990.1D2	BnaA02g25220.1D2
R53	BnaC02g32010.1D2	BnaA02g25240.1D2
R53	BnaC02g32020.1D2	BnaA02g25260.1D2
R53	BnaC02g32030.1D2	BnaA02g25270.1D2
R53	BnaC02g32070.1D2	BnaA02g25580.1D2
R53	BnaC02g32100.1D2	BnaA02g25610.1D2
R53	BnaC02g32110.1D2	BnaA02g25620.1D2
R53	BnaC02g32140.1D2	BnaA02g25650.1D2
R53	BnaC02g32150.1D2	BnaA02g25660.1D2
R53	BnaC02g32240.1D2	BnaA02g27030.1D2
R53	BnaC02g32280.1D2	BnaA02g27000.1D2
R53	BnaC02g32310.1D2	BnaA02g26970.1D2
R53	BnaC02g32320.1D2	BnaA02g26970.1D2
R53	BnaC02g32330.1D2	BnaA02g26960.1D2
R53	BnaC02g32360.1D2	BnaA02g26940.1D2
R53	BnaC02g32380.1D2	BnaA02g26920.1D2
R53	BnaC02g32390.1D2	BnaA02g27170.1D2
R53	BnaC02g32400.1D2	BnaA02g26910.1D2
R53	BnaC02g32430.1D2	BnaA02g26900.1D2
R53	BnaC02g32450.1D2	BnaA02g26880.1D2
R53	BnaC02g32460.1D2	BnaA02g26870.1D2
R53	BnaC02g32470.1D2	BnaA02g26860.1D2
R53	BnaC02g32480.1D2	BnaA02g26850.1D2
R53	BnaC02g32490.1D2	BnaA02g26840.1D2
R53	BnaC02g32510.1D2	BnaA02g26830.1D2
R53	BnaC02g32550.1D2	BnaA02g26810.1D2
R53	BnaC02g32560.1D2	BnaA02g26800.1D2
R53	BnaC02g32590.1D2	BnaA02g26780.1D2
R53	BnaC02g32600.1D2	BnaA02g26770.1D2
R53	BnaC02g32610.1D2	BnaA02g26790.1D2
R53	BnaC02g32680.1D2	BnaA02g25820.1D2
R53	BnaC02g32690.1D2	BnaA02g25830.1D2
R53	BnaC02g32730.1D2	BnaA02g25860.1D2
R53	BnaC02g32750.1D2	BnaA02g25880.1D2
R53	BnaC02g32830.1D2	BnaA02g26740.1D2
R53	BnaC02g32840.1D2	BnaA02g26730.1D2
R53	BnaC02g32850.1D2	BnaA02g26710.1D2
R53	BnaC02g32880.1D2	BnaA02g26700.1D2
R53	BnaC02g32930.1D2	BnaA02g27810.1D2
R53	BnaC02g32970.1D2	BnaA02g26010.1D2
R53	BnaC02g32990.1D2	BnaA02g26050.1D2
R53	BnaC02g33180.1D2	BnaA02g26180.1D2
R53	BnaC02g33190.1D2	BnaA02g26190.1D2
R53	BnaC02g33200.1D2	BnaA02g26200.1D2

R53	BnaC02g33210.1D2	BnaA02g26290.1D2
R53	BnaC02g33240.1D2	BnaA02g26310.1D2
R53	BnaC02g33250.1D2	BnaA02g26320.1D2
R53	BnaC02g33310.1D2	BnaA02g26370.1D2
R53	BnaC02g33340.1D2	BnaA02g25910.1D2
R53	BnaC02g33410.1D2	BnaA02g26390.1D2
R53	BnaC02g33450.1D2	BnaA02g26420.1D2
R53	BnaC02g33490.1D2	BnaA02g26450.1D2
R53	BnaC02g33500.1D2	BnaA02g27730.1D2
R53	BnaC02g33540.1D2	BnaA02g26480.1D2
R53	BnaC02g33550.1D2	BnaA02g26490.1D2
R53	BnaC02g33660.1D2	BnaA02g26650.1D2
R53	BnaC02g33940.1D2	BnaA02g27100.1D2
R53	BnaC02g33950.1D2	BnaA02g27110.1D2
R53	BnaC02g34080.1D2	BnaA02g27280.1D2
R53	BnaC02g34130.1D2	BnaA02g27290.1D2
R53	BnaC02g34140.1D2	BnaA02g27300.1D2
R53	BnaC02g34250.1D2	BnaA02g27400.1D2
R53	BnaC02g34340.1D2	BnaA02g27490.1D2
R53	BnaC02g34400.1D2	BnaA02g27780.1D2
R53	BnaC02g34570.1D2	BnaA02g27850.1D2
R53	BnaC02g34620.1D2	BnaA02g27900.1D2
R53	BnaC02g34670.1D2	BnaA02g28030.1D2
R53	BnaC02g34810.1D2	BnaA02g28180.1D2
R53	BnaC02g34840.1D2	BnaA02g28210.1D2
R53	BnaC02g34850.1D2	BnaA02g28220.1D2
R53	BnaC02g34910.1D2	BnaA02g28290.1D2
R53	BnaC02g35010.1D2	BnaA02g27550.1D2
R53	BnaC02g35070.1D2	BnaA02g28530.1D2
R53	BnaC02g35100.1D2	BnaA02g28560.1D2
R53	BnaC02g35190.1D2	BnaA02g27970.1D2
R53	BnaC02g35240.1D2	BnaA02g28610.1D2
R53	BnaC02g35260.1D2	BnaA02g28630.1D2
R53	BnaC02g35340.1D2	BnaA02g28790.1D2
R53	BnaC02g35390.1D2	BnaA02g28820.1D2
R53	BnaC02g35490.1D2	BnaA02g28950.1D2
R53	BnaC02g35520.1D2	BnaA02g28980.1D2
R53	BnaC02g35530.1D2	BnaA02g28990.1D2
R53	BnaC02g35640.1D2	BnaA02g29090.1D2
R53	BnaC02g35650.1D2	BnaA02g29110.1D2
R53	BnaC02g35660.1D2	BnaA02g29120.1D2
R53	BnaC02g35710.1D2	BnaA02g28000.1D2
R53	BnaC02g35840.1D2	BnaA02g29230.1D2
R53	BnaC02g35850.1D2	BnaA02g29240.1D2
R53	BnaC02g35870.1D2	BnaA02g29270.1D2
R53	BnaC02g35920.1D2	BnaA02g29310.1D2
R53	BnaC02g35930.1D2	BnaA02g29320.1D2
R53	BnaC02g35950.1D2	BnaA02g29340.1D2
R53	BnaC02g35960.1D2	BnaA02g29350.1D2
R53	BnaC02g35990.1D2	BnaA02g29360.1D2
R53	BnaC02g36010.1D2	BnaA02g29380.1D2

R53	BnaC02g36070.1D2	BnaA02g28350.1D2
R53	BnaC02g36120.1D2	BnaA02g28400.1D2
R53	BnaC02g36210.1D2	BnaA02g28500.1D2
R53	BnaC02g36450.1D2	BnaA02g29650.1D2
R53	BnaC02g36470.1D2	BnaA02g29670.1D2
R53	BnaC02g36510.1D2	BnaA02g29690.1D2
R53	BnaC02g36570.1D2	BnaA02g29730.1D2
R53	BnaC02g36580.1D2	BnaA02g29740.1D2
R53	BnaC02g36840.1D2	BnaA02g30050.1D2
R53	BnaC02g37500.1D2	BnaA02g29390.1D2
R53	BnaC02g40230.1D2	BnaA02g33460.1D2
R53	BnaC02g40560.1D2	BnaA02g33790.1D2
R53	BnaC02g40630.1D2	BnaA02g33830.1D2
R53	BnaC02g40800.1D2	BnaA02g33950.1D2
R53	BnaC02g40810.1D2	BnaA02g33960.1D2
R53	BnaC02g40820.1D2	BnaA02g33970.1D2
R53	BnaC02g40910.1D2	BnaA02g34120.1D2
R53	BnaC02g40940.1D2	BnaA02g34150.1D2
R53	BnaC02g41060.1D2	BnaA02g34240.1D2
R53	BnaC02g41270.1D2	BnaA02g34110.1D2
R53	BnaC02g41400.1D2	BnaA02g34510.1D2
R53	BnaC02g41450.1D2	BnaA02g34890.1D2
R53	BnaC02g41470.1D2	BnaA02g34910.1D2
R53	BnaC02g41550.1D2	BnaA02g35000.1D2
R53	BnaC02g41560.1D2	BnaA02g35010.1D2
R53	BnaC02g41580.1D2	BnaA02g35040.1D2
R53	BnaC02g41600.1D2	BnaA02g35060.1D2
R53	BnaC02g41620.1D2	BnaA02g35080.1D2
R53	BnaC02g41630.1D2	BnaA02g35090.1D2
R53	BnaC02g41750.1D2	BnaA02g34600.1D2
R53	BnaC02g41770.1D2	BnaA02g34570.1D2
R53	BnaC02g41790.1D2	BnaA02g34550.1D2

List of HE genes (C03 to A03)

**Accession**	**Lost gene**	**Duplicated HE gene**
Abukuma Natane	BnaC03g00050.1D2	BnaA03g00130.1D2
Abukuma Natane	BnaC03g00080.1D2	BnaA03g00170.1D2
Abukuma Natane	BnaC03g00120.1D2	BnaA03g00210.1D2
Abukuma Natane	BnaC03g00140.1D2	BnaA03g00230.1D2
Abukuma Natane	BnaC03g00150.1D2	BnaA03g00240.1D2
Abukuma Natane	BnaC03g00180.1D2	BnaA03g00270.1D2
Abukuma Natane	BnaC03g00250.1D2	BnaA03g00330.1D2
Abukuma Natane	BnaC03g00260.1D2	BnaA03g00340.1D2
Abukuma Natane	BnaC03g00270.1D2	BnaA03g00350.1D2
Abukuma Natane	BnaC03g00320.1D2	BnaA03g00400.1D2
Abukuma Natane	BnaC03g00340.1D2	BnaA03g00420.1D2
Abukuma Natane	BnaC03g00360.1D2	BnaA03g00440.1D2
Abukuma Natane	BnaC03g00420.1D2	BnaA03g00490.1D2
Abukuma Natane	BnaC03g00460.1D2	BnaA03g02020.1D2
Abukuma Natane	BnaC03g00470.1D2	BnaA03g02030.1D2
Abukuma Natane	BnaC03g00500.1D2	BnaA03g02050.1D2

Abukuma Natane	BnaC03g00520.1D2	BnaA03g02070.1D2
Abukuma Natane	BnaC03g00530.1D2	BnaA03g02080.1D2
Abukuma Natane	BnaC03g00540.1D2	BnaA03g02090.1D2
Abukuma Natane	BnaC03g00560.1D2	BnaA03g02110.1D2
Abukuma Natane	BnaC03g00580.1D2	BnaA03g02250.1D2
Abukuma Natane	BnaC03g00590.1D2	BnaA03g02240.1D2
Abukuma Natane	BnaC03g00610.1D2	BnaA03g02220.1D2
Abukuma Natane	BnaC03g00670.1D2	BnaA03g02170.1D2
Abukuma Natane	BnaC03g00710.1D2	BnaA03g02140.1D2
Abukuma Natane	BnaC03g00720.1D2	BnaA03g02130.1D2
Abukuma Natane	BnaC03g00740.1D2	BnaA03g00540.1D2
Abukuma Natane	BnaC03g00820.1D2	BnaA03g00620.1D2
Abukuma Natane	BnaC03g00830.1D2	BnaA03g00630.1D2
Abukuma Natane	BnaC03g00850.1D2	BnaA03g00650.1D2
Abukuma Natane	BnaC03g00890.1D2	BnaA03g00680.1D2
Abukuma Natane	BnaC03g00910.1D2	BnaA03g00700.1D2
Abukuma Natane	BnaC03g00920.1D2	BnaA03g00710.1D2
Abukuma Natane	BnaC03g00940.1D2	BnaA03g00730.1D2
Abukuma Natane	BnaC03g00960.1D2	BnaA03g00750.1D2
Abukuma Natane	BnaC03g00970.1D2	BnaA03g00760.1D2
Abukuma Natane	BnaC03g00990.1D2	BnaA03g00780.1D2
Abukuma Natane	BnaC03g01030.1D2	BnaA03g00820.1D2
Abukuma Natane	BnaC03g01040.1D2	BnaA03g00830.1D2
Abukuma Natane	BnaC03g01160.1D2	BnaA03g00980.1D2
Abukuma Natane	BnaC03g01230.1D2	BnaA03g01050.1D2
Abukuma Natane	BnaC03g01240.1D2	BnaA03g01060.1D2
Abukuma Natane	BnaC03g01250.1D2	BnaA03g01070.1D2
Abukuma Natane	BnaC03g01260.1D2	BnaA03g01080.1D2
Abukuma Natane	BnaC03g01270.1D2	BnaA03g01090.1D2
Abukuma Natane	BnaC03g01320.1D2	BnaA03g01190.1D2
Abukuma Natane	BnaC03g01340.1D2	BnaA03g01200.1D2
Abukuma Natane	BnaC03g01360.1D2	BnaA03g01220.1D2
Abukuma Natane	BnaC03g01410.1D2	BnaA03g01290.1D2
Abukuma Natane	BnaC03g01420.1D2	BnaA03g01300.1D2
Abukuma Natane	BnaC03g01430.1D2	BnaA03g01310.1D2
Abukuma Natane	BnaC03g01450.1D2	BnaA03g01330.1D2
Abukuma Natane	BnaC03g01460.1D2	BnaA03g01340.1D2
Abukuma Natane	BnaC03g01470.1D2	BnaA03g01350.1D2
Abukuma Natane	BnaC03g01480.1D2	BnaA03g01360.1D2
Abukuma Natane	BnaC03g01490.1D2	BnaA03g01370.1D2
Abukuma Natane	BnaC03g01530.1D2	BnaA03g01410.1D2
Abukuma Natane	BnaC03g01540.1D2	BnaA03g01420.1D2
Abukuma Natane	BnaC03g01570.1D2	BnaA03g01440.1D2
Abukuma Natane	BnaC03g01610.1D2	BnaA03g01470.1D2
Abukuma Natane	BnaC03g01630.1D2	BnaA03g07590.1D2
Abukuma Natane	BnaC03g01640.1D2	BnaA03g07600.1D2
Abukuma Natane	BnaC03g01670.1D2	BnaA03g07630.1D2
Abukuma Natane	BnaC03g01790.1D2	BnaA03g07730.1D2
Abukuma Natane	BnaC03g01820.1D2	BnaA03g02290.1D2
Abukuma Natane	BnaC03g01840.1D2	BnaA03g01490.1D2
Abukuma Natane	BnaC03g01920.1D2	BnaA03g01570.1D2

Abukuma Natane	BnaC03g01950.1D2	BnaA03g01600.1D2
Abukuma Natane	BnaC03g02010.1D2	BnaA03g01640.1D2
Abukuma Natane	BnaC03g02030.1D2	BnaA03g01670.1D2
Abukuma Natane	BnaC03g02030.1D2	BnaA03g01690.1D2
Abukuma Natane	BnaC03g02040.1D2	BnaA03g01670.1D2
Abukuma Natane	BnaC03g02040.1D2	BnaA03g01690.1D2
Abukuma Natane	BnaC03g02050.1D2	BnaA03g01710.1D2
Abukuma Natane	BnaC03g02070.1D2	BnaA03g01710.1D2
Abukuma Natane	BnaC03g02090.1D2	BnaA03g01730.1D2
Abukuma Natane	BnaC03g02180.1D2	BnaA03g01780.1D2
Abukuma Natane	BnaC03g02190.1D2	BnaA03g01790.1D2
Abukuma Natane	BnaC03g02200.1D2	BnaA03g01800.1D2
Abukuma Natane	BnaC03g02220.1D2	BnaA03g01810.1D2
Abukuma Natane	BnaC03g02240.1D2	BnaA03g01840.1D2
Abukuma Natane	BnaC03g02270.1D2	BnaA03g01860.1D2
Abukuma Natane	BnaC03g02300.1D2	BnaA03g01890.1D2
Abukuma Natane	BnaC03g02300.1D2	BnaA03g01910.1D2
Abukuma Natane	BnaC03g02380.1D2	BnaA03g07570.1D2
Abukuma Natane	BnaC03g02400.1D2	BnaA03g07560.1D2
Abukuma Natane	BnaC03g02430.1D2	BnaA03g07480.1D2
Abukuma Natane	BnaC03g02510.1D2	BnaA03g02530.1D2
Abukuma Natane	BnaC03g02520.1D2	BnaA03g02540.1D2
Abukuma Natane	BnaC03g02530.1D2	BnaA03g02550.1D2
Abukuma Natane	BnaC03g02540.1D2	BnaA03g02560.1D2
Abukuma Natane	BnaC03g02550.1D2	BnaA03g02570.1D2
Abukuma Natane	BnaC03g02590.1D2	BnaA03g02590.1D2
Abukuma Natane	BnaC03g02600.1D2	BnaA03g02600.1D2
Abukuma Natane	BnaC03g02610.1D2	BnaA03g02600.1D2
Abukuma Natane	BnaC03g02650.1D2	BnaA03g02610.1D2
Abukuma Natane	BnaC03g02650.1D2	BnaA03g02620.1D2
Abukuma Natane	BnaC03g02680.1D2	BnaA03g02660.1D2
Abukuma Natane	BnaC03g02700.1D2	BnaA03g02690.1D2
Abukuma Natane	BnaC03g02780.1D2	BnaA03g02780.1D2
Abukuma Natane	BnaC03g02840.1D2	BnaA03g02840.1D2
Abukuma Natane	BnaC03g02850.1D2	BnaA03g02850.1D2
Abukuma Natane	BnaC03g02860.1D2	BnaA03g02860.1D2
Abukuma Natane	BnaC03g02880.1D2	BnaA03g02880.1D2
Abukuma Natane	BnaC03g02890.1D2	BnaA03g02890.1D2
Abukuma Natane	BnaC03g02920.1D2	BnaA03g02940.1D2
Abukuma Natane	BnaC03g02930.1D2	BnaA03g02950.1D2
Abukuma Natane	BnaC03g02990.1D2	BnaA03g03020.1D2
Abukuma Natane	BnaC03g03010.1D2	BnaA03g03040.1D2
Abukuma Natane	BnaC03g03020.1D2	BnaA03g03050.1D2
Abukuma Natane	BnaC03g03030.1D2	BnaA03g03060.1D2
Abukuma Natane	BnaC03g03050.1D2	BnaA03g07820.1D2
Abukuma Natane	BnaC03g03080.1D2	BnaA03g07290.1D2
Abukuma Natane	BnaC03g03120.1D2	BnaA03g07330.1D2
Abukuma Natane	BnaC03g03130.1D2	BnaA03g07340.1D2
Abukuma Natane	BnaC03g03150.1D2	BnaA03g07360.1D2
Abukuma Natane	BnaC03g03180.1D2	BnaA03g03120.1D2
Abukuma Natane	BnaC03g03210.1D2	BnaA03g03150.1D2

Abukuma Natane	BnaC03g03230.1D2	BnaA03g03160.1D2
Abukuma Natane	BnaC03g03250.1D2	BnaA03g03180.1D2
Abukuma Natane	BnaC03g03270.1D2	BnaA03g03220.1D2
Abukuma Natane	BnaC03g03270.1D2	BnaA03g03230.1D2
Abukuma Natane	BnaC03g03280.1D2	BnaA03g03220.1D2
Abukuma Natane	BnaC03g03280.1D2	BnaA03g03230.1D2
Abukuma Natane	BnaC03g03290.1D2	BnaA03g03240.1D2
Abukuma Natane	BnaC03g03300.1D2	BnaA03g03250.1D2
Abukuma Natane	BnaC03g03320.1D2	BnaA03g03270.1D2
Abukuma Natane	BnaC03g03330.1D2	BnaA03g03280.1D2
Abukuma Natane	BnaC03g03350.1D2	BnaA03g03300.1D2
Abukuma Natane	BnaC03g03400.1D2	BnaA03g07260.1D2
Abukuma Natane	BnaC03g03410.1D2	BnaA03g07250.1D2
Abukuma Natane	BnaC03g03420.1D2	BnaA03g07240.1D2
Abukuma Natane	BnaC03g03440.1D2	BnaA03g07220.1D2
Abukuma Natane	BnaC03g03470.1D2	BnaA03g07200.1D2
Abukuma Natane	BnaC03g03500.1D2	BnaA03g07180.1D2
Abukuma Natane	BnaC03g03510.1D2	BnaA03g07170.1D2
Abukuma Natane	BnaC03g03570.1D2	BnaA03g03320.1D2
Abukuma Natane	BnaC03g03590.1D2	BnaA03g03370.1D2
Abukuma Natane	BnaC03g03620.1D2	BnaA03g03390.1D2
Abukuma Natane	BnaC03g03630.1D2	BnaA03g03400.1D2
Abukuma Natane	BnaC03g03690.1D2	BnaA03g03430.1D2
Abukuma Natane	BnaC03g03710.1D2	BnaA03g03450.1D2
Abukuma Natane	BnaC03g03730.1D2	BnaA03g03470.1D2
Abukuma Natane	BnaC03g03740.1D2	BnaA03g03480.1D2
Abukuma Natane	BnaC03g03750.1D2	BnaA03g03490.1D2
Abukuma Natane	BnaC03g03760.1D2	BnaA03g03500.1D2
Abukuma Natane	BnaC03g03800.1D2	BnaA03g03530.1D2
Abukuma Natane	BnaC03g03820.1D2	BnaA03g03550.1D2
Abukuma Natane	BnaC03g03890.1D2	BnaA03g01140.1D2
Abukuma Natane	BnaC03g03900.1D2	BnaA03g01150.1D2
Abukuma Natane	BnaC03g03920.1D2	BnaA03g01170.1D2
Abukuma Natane	BnaC03g03970.1D2	BnaA03g03620.1D2
Abukuma Natane	BnaC03g04050.1D2	BnaA03g03670.1D2
Abukuma Natane	BnaC03g04060.1D2	BnaA03g03680.1D2
Abukuma Natane	BnaC03g04080.1D2	BnaA03g03700.1D2
Abukuma Natane	BnaC03g04100.1D2	BnaA03g03720.1D2
Abukuma Natane	BnaC03g04130.1D2	BnaA03g03740.1D2
Abukuma Natane	BnaC03g04130.1D2	BnaA03g03750.1D2
Abukuma Natane	BnaC03g04140.1D2	BnaA03g03760.1D2
Abukuma Natane	BnaC03g04160.1D2	BnaA03g03800.1D2
Abukuma Natane	BnaC03g04170.1D2	BnaA03g03490.1D2
Abukuma Natane	BnaC03g04170.1D2	BnaA03g03790.1D2
Abukuma Natane	BnaC03g04180.1D2	BnaA03g03780.1D2
Abukuma Natane	BnaC03g04210.1D2	BnaA03g03830.1D2
Abukuma Natane	BnaC03g04220.1D2	BnaA03g03840.1D2
Abukuma Natane	BnaC03g04240.1D2	BnaA03g03870.1D2
Abukuma Natane	BnaC03g04390.1D2	BnaA03g03970.1D2
Abukuma Natane	BnaC03g04420.1D2	BnaA03g04000.1D2
Abukuma Natane	BnaC03g04460.1D2	BnaA03g04020.1D2

Abukuma Natane	BnaC03g04500.1D2	BnaA03g04050.1D2
Abukuma Natane	BnaC03g04530.1D2	BnaA03g04080.1D2
Abukuma Natane	BnaC03g04560.1D2	BnaA03g04100.1D2
Abukuma Natane	BnaC03g04570.1D2	BnaA03g04110.1D2
Abukuma Natane	BnaC03g04580.1D2	BnaA03g04120.1D2
Abukuma Natane	BnaC03g04600.1D2	BnaA03g04130.1D2
Abukuma Natane	BnaC03g04610.1D2	BnaA03g04140.1D2
Abukuma Natane	BnaC03g04620.1D2	BnaA03g04150.1D2
Abukuma Natane	BnaC03g04630.1D2	BnaA03g04160.1D2
Abukuma Natane	BnaC03g04660.1D2	BnaA03g04200.1D2
Abukuma Natane	BnaC03g04670.1D2	BnaA03g04210.1D2
Abukuma Natane	BnaC03g04710.1D2	BnaA03g04250.1D2
Abukuma Natane	BnaC03g04730.1D2	BnaA03g04270.1D2
Abukuma Natane	BnaC03g04780.1D2	BnaA03g04320.1D2
Abukuma Natane	BnaC03g04800.1D2	BnaA03g04330.1D2
Abukuma Natane	BnaC03g04820.1D2	BnaA03g04350.1D2
Abukuma Natane	BnaC03g04830.1D2	BnaA03g04360.1D2
Abukuma Natane	BnaC03g04900.1D2	BnaA03g04400.1D2
Abukuma Natane	BnaC03g04950.1D2	BnaA03g04450.1D2
Abukuma Natane	BnaC03g04990.1D2	BnaA03g04450.1D2
Abukuma Natane	BnaC03g05070.1D2	BnaA03g04540.1D2
Abukuma Natane	BnaC03g05100.1D2	BnaA03g04580.1D2
Abukuma Natane	BnaC03g05150.1D2	BnaA03g04620.1D2
Abukuma Natane	BnaC03g05180.1D2	BnaA03g04650.1D2
Abukuma Natane	BnaC03g05190.1D2	BnaA03g04660.1D2
Abukuma Natane	BnaC03g05210.1D2	BnaA03g04680.1D2
Abukuma Natane	BnaC03g05220.1D2	BnaA03g04690.1D2
Abukuma Natane	BnaC03g05260.1D2	BnaA03g04790.1D2
Abukuma Natane	BnaC03g05290.1D2	BnaA03g04820.1D2
Abukuma Natane	BnaC03g05320.1D2	BnaA03g04850.1D2
Abukuma Natane	BnaC03g05420.1D2	BnaA03g04920.1D2
Abukuma Natane	BnaC03g05430.1D2	BnaA03g04930.1D2
Abukuma Natane	BnaC03g05440.1D2	BnaA03g04940.1D2
Abukuma Natane	BnaC03g05470.1D2	BnaA03g04970.1D2
Abukuma Natane	BnaC03g05510.1D2	BnaA03g05010.1D2
Abukuma Natane	BnaC03g05520.1D2	BnaA03g05020.1D2
Abukuma Natane	BnaC03g05530.1D2	BnaA03g05030.1D2
Abukuma Natane	BnaC03g05600.1D2	BnaA03g05090.1D2
Abukuma Natane	BnaC03g05620.1D2	BnaA03g05110.1D2
Abukuma Natane	BnaC03g05640.1D2	BnaA03g05120.1D2
Abukuma Natane	BnaC03g05650.1D2	BnaA03g05130.1D2
Abukuma Natane	BnaC03g05660.1D2	BnaA03g05140.1D2
Abukuma Natane	BnaC03g05680.1D2	BnaA03g05160.1D2
Abukuma Natane	BnaC03g05700.1D2	BnaA03g05190.1D2
Abukuma Natane	BnaC03g05840.1D2	BnaA03g05300.1D2
Abukuma Natane	BnaC03g05890.1D2	BnaA03g05340.1D2
Abukuma Natane	BnaC03g05990.1D2	BnaA03g05450.1D2
Abukuma Natane	BnaC03g06050.1D2	BnaA03g05550.1D2
Abukuma Natane	BnaC03g06130.1D2	BnaA03g05610.1D2
Abukuma Natane	BnaC03g06210.1D2	BnaA03g05680.1D2
Abukuma Natane	BnaC03g10890.1D2	BnaA03g04760.1D2

Abukuma Natane	BnaC03g10900.1D2	BnaA03g04750.1D2
Abukuma Natane	BnaC03g10930.1D2	BnaA03g04730.1D2
Abukuma Natane	BnaC03g10940.1D2	BnaA03g04720.1D2
Abukuma Natane	BnaC03g10970.1D2	BnaA03g04700.1D2
Abukuma Natane	BnaC03g11290.1D2	BnaA03g10670.1D2
Abukuma Natane	BnaC03g11320.1D2	BnaA03g10540.1D2
Abukuma Natane	BnaC03g11350.1D2	BnaA03g10570.1D2
Abukuma Natane	BnaC03g11360.1D2	BnaA03g10580.1D2
Abukuma Natane	BnaC03g11370.1D2	BnaA03g10590.1D2
Abukuma Natane	BnaC03g11490.1D2	BnaA03g10720.1D2
Abukuma Natane	BnaC03g11520.1D2	BnaA03g10750.1D2
Evvin	BnaC03g00120.1D2	BnaA03g00210.1D2
Evvin	BnaC03g00140.1D2	BnaA03g00230.1D2
Evvin	BnaC03g00150.1D2	BnaA03g00240.1D2
Evvin	BnaC03g00160.1D2	BnaA03g00250.1D2
Evvin	BnaC03g00270.1D2	BnaA03g00350.1D2
Evvin	BnaC03g00280.1D2	BnaA03g00360.1D2
Evvin	BnaC03g00320.1D2	BnaA03g00400.1D2
Evvin	BnaC03g00330.1D2	BnaA03g00410.1D2
Evvin	BnaC03g00360.1D2	BnaA03g00440.1D2
Evvin	BnaC03g00420.1D2	BnaA03g00490.1D2
Evvin	BnaC03g00450.1D2	BnaA03g02010.1D2
Evvin	BnaC03g00470.1D2	BnaA03g02030.1D2
Evvin	BnaC03g00500.1D2	BnaA03g02050.1D2
Evvin	BnaC03g00520.1D2	BnaA03g02070.1D2
Evvin	BnaC03g00530.1D2	BnaA03g02080.1D2
Evvin	BnaC03g00540.1D2	BnaA03g02090.1D2
Evvin	BnaC03g00560.1D2	BnaA03g02110.1D2
Evvin	BnaC03g00580.1D2	BnaA03g02250.1D2
Evvin	BnaC03g00610.1D2	BnaA03g02220.1D2
Evvin	BnaC03g00670.1D2	BnaA03g02170.1D2
Evvin	BnaC03g00710.1D2	BnaA03g02140.1D2
Evvin	BnaC03g00720.1D2	BnaA03g02130.1D2
Evvin	BnaC03g00740.1D2	BnaA03g00540.1D2
Evvin	BnaC03g00820.1D2	BnaA03g00620.1D2
Evvin	BnaC03g00850.1D2	BnaA03g00650.1D2
Evvin	BnaC03g00960.1D2	BnaA03g00750.1D2
Evvin	BnaC03g01060.1D2	BnaA03g00850.1D2
Evvin	BnaC03g01160.1D2	BnaA03g00980.1D2
Evvin	BnaC03g01170.1D2	BnaA03g00990.1D2
Evvin	BnaC03g01220.1D2	BnaA03g01040.1D2
Evvin	BnaC03g01230.1D2	BnaA03g01050.1D2
Evvin	BnaC03g01240.1D2	BnaA03g01060.1D2
Evvin	BnaC03g01260.1D2	BnaA03g01080.1D2
Evvin	BnaC03g01360.1D2	BnaA03g01220.1D2
Evvin	BnaC03g01410.1D2	BnaA03g01290.1D2
Evvin	BnaC03g01420.1D2	BnaA03g01300.1D2
Evvin	BnaC03g01430.1D2	BnaA03g01310.1D2
Evvin	BnaC03g01450.1D2	BnaA03g01330.1D2
Evvin	BnaC03g01460.1D2	BnaA03g01340.1D2
Evvin	BnaC03g01470.1D2	BnaA03g01350.1D2

Evvin	BnaC03g01480.1D2	BnaA03g01360.1D2
Evvin	BnaC03g01490.1D2	BnaA03g01370.1D2
Evvin	BnaC03g01540.1D2	BnaA03g01420.1D2
Evvin	BnaC03g01820.1D2	BnaA03g02290.1D2
Evvin	BnaC03g02010.1D2	BnaA03g01640.1D2
Evvin	BnaC03g02190.1D2	BnaA03g01790.1D2
Evvin	BnaC03g02240.1D2	BnaA03g01840.1D2
Evvin	BnaC03g02300.1D2	BnaA03g01890.1D2
Evvin	BnaC03g02300.1D2	BnaA03g01910.1D2
Evvin	BnaC03g02400.1D2	BnaA03g07560.1D2
Evvin	BnaC03g02430.1D2	BnaA03g07480.1D2
Evvin	BnaC03g02600.1D2	BnaA03g02600.1D2
Evvin	BnaC03g02610.1D2	BnaA03g02600.1D2
Evvin	BnaC03g02860.1D2	BnaA03g02860.1D2
Evvin	BnaC03g02880.1D2	BnaA03g02880.1D2
Evvin	BnaC03g02920.1D2	BnaA03g02940.1D2
Evvin	BnaC03g03080.1D2	BnaA03g07290.1D2
Evvin	BnaC03g03110.1D2	BnaA03g07320.1D2
Evvin	BnaC03g03300.1D2	BnaA03g03250.1D2
Evvin	BnaC03g03320.1D2	BnaA03g03270.1D2
Evvin	BnaC03g03330.1D2	BnaA03g03280.1D2
Evvin	BnaC03g03340.1D2	BnaA03g03290.1D2
Evvin	BnaC03g03350.1D2	BnaA03g03300.1D2
Evvin	BnaC03g03420.1D2	BnaA03g07240.1D2
Evvin	BnaC03g03440.1D2	BnaA03g07220.1D2
Evvin	BnaC03g03510.1D2	BnaA03g07170.1D2
Evvin	BnaC03g03630.1D2	BnaA03g03400.1D2
Evvin	BnaC03g03690.1D2	BnaA03g03430.1D2
Evvin	BnaC03g03900.1D2	BnaA03g01150.1D2
Evvin	BnaC03g03920.1D2	BnaA03g01170.1D2
Evvin	BnaC03g04170.1D2	BnaA03g03490.1D2
Evvin	BnaC03g04170.1D2	BnaA03g03790.1D2
Evvin	BnaC03g04210.1D2	BnaA03g03830.1D2
Evvin	BnaC03g04240.1D2	BnaA03g03870.1D2
Evvin	BnaC03g04460.1D2	BnaA03g04020.1D2
Evvin	BnaC03g04530.1D2	BnaA03g04080.1D2
Evvin	BnaC03g04560.1D2	BnaA03g04100.1D2
Evvin	BnaC03g04570.1D2	BnaA03g04110.1D2
Evvin	BnaC03g04580.1D2	BnaA03g04120.1D2
Evvin	BnaC03g04610.1D2	BnaA03g04140.1D2
Evvin	BnaC03g04660.1D2	BnaA03g04200.1D2
Evvin	BnaC03g04710.1D2	BnaA03g04250.1D2
Evvin	BnaC03g04730.1D2	BnaA03g04270.1D2
Evvin	BnaC03g04780.1D2	BnaA03g04320.1D2
Evvin	BnaC03g04990.1D2	BnaA03g04450.1D2
Evvin	BnaC03g05100.1D2	BnaA03g04580.1D2
Evvin	BnaC03g05180.1D2	BnaA03g04650.1D2
Evvin	BnaC03g20320.1D2	BnaA03g18360.1D2
Expert	BnaC03g00050.1D2	BnaA03g00130.1D2
Expert	BnaC03g00080.1D2	BnaA03g00170.1D2
Expert	BnaC03g00120.1D2	BnaA03g00210.1D2

Expert	BnaC03g00140.1D2	BnaA03g00230.1D2
Expert	BnaC03g00150.1D2	BnaA03g00240.1D2
Expert	BnaC03g00160.1D2	BnaA03g00250.1D2
Expert	BnaC03g00180.1D2	BnaA03g00270.1D2
Expert	BnaC03g00250.1D2	BnaA03g00330.1D2
Expert	BnaC03g00260.1D2	BnaA03g00340.1D2
Expert	BnaC03g00270.1D2	BnaA03g00350.1D2
Expert	BnaC03g00280.1D2	BnaA03g00360.1D2
Expert	BnaC03g00320.1D2	BnaA03g00400.1D2
Expert	BnaC03g00330.1D2	BnaA03g00410.1D2
Expert	BnaC03g00340.1D2	BnaA03g00420.1D2
Expert	BnaC03g00360.1D2	BnaA03g00440.1D2
Expert	BnaC03g00420.1D2	BnaA03g00490.1D2
Expert	BnaC03g00450.1D2	BnaA03g02010.1D2
Expert	BnaC03g00460.1D2	BnaA03g02020.1D2
Expert	BnaC03g00470.1D2	BnaA03g02030.1D2
Expert	BnaC03g00500.1D2	BnaA03g02050.1D2
Expert	BnaC03g00520.1D2	BnaA03g02070.1D2
Expert	BnaC03g00530.1D2	BnaA03g02080.1D2
Expert	BnaC03g00540.1D2	BnaA03g02090.1D2
Expert	BnaC03g00560.1D2	BnaA03g02110.1D2
Expert	BnaC03g00580.1D2	BnaA03g02250.1D2
Expert	BnaC03g00610.1D2	BnaA03g02220.1D2
Expert	BnaC03g00670.1D2	BnaA03g02170.1D2
Expert	BnaC03g00710.1D2	BnaA03g02140.1D2
Expert	BnaC03g00720.1D2	BnaA03g02130.1D2
Expert	BnaC03g00740.1D2	BnaA03g00540.1D2
Expert	BnaC03g00820.1D2	BnaA03g00620.1D2
Expert	BnaC03g00850.1D2	BnaA03g00650.1D2
Expert	BnaC03g00890.1D2	BnaA03g00680.1D2
Expert	BnaC03g00910.1D2	BnaA03g00700.1D2
Expert	BnaC03g00920.1D2	BnaA03g00710.1D2
Expert	BnaC03g00940.1D2	BnaA03g00730.1D2
Expert	BnaC03g00960.1D2	BnaA03g00750.1D2
Expert	BnaC03g00970.1D2	BnaA03g00760.1D2
Expert	BnaC03g00990.1D2	BnaA03g00780.1D2
Expert	BnaC03g01020.1D2	BnaA03g00810.1D2
Expert	BnaC03g01030.1D2	BnaA03g00820.1D2
Expert	BnaC03g01040.1D2	BnaA03g00830.1D2
Expert	BnaC03g01050.1D2	BnaA03g00840.1D2
Expert	BnaC03g01070.1D2	BnaA03g00860.1D2
Expert	BnaC03g01160.1D2	BnaA03g00980.1D2
Expert	BnaC03g01220.1D2	BnaA03g01040.1D2
Expert	BnaC03g01230.1D2	BnaA03g01050.1D2
Expert	BnaC03g01240.1D2	BnaA03g01060.1D2
Expert	BnaC03g01260.1D2	BnaA03g01080.1D2
Expert	BnaC03g01270.1D2	BnaA03g01090.1D2
Expert	BnaC03g01320.1D2	BnaA03g01190.1D2
Expert	BnaC03g01340.1D2	BnaA03g01200.1D2
Expert	BnaC03g01360.1D2	BnaA03g01220.1D2
Expert	BnaC03g01410.1D2	BnaA03g01290.1D2

Expert	BnaC03g01420.1D2	BnaA03g01300.1D2
Expert	BnaC03g01430.1D2	BnaA03g01310.1D2
Expert	BnaC03g01450.1D2	BnaA03g01330.1D2
Expert	BnaC03g01460.1D2	BnaA03g01340.1D2
Expert	BnaC03g01470.1D2	BnaA03g01350.1D2
Expert	BnaC03g01480.1D2	BnaA03g01360.1D2
Expert	BnaC03g01490.1D2	BnaA03g01370.1D2
Expert	BnaC03g01530.1D2	BnaA03g01410.1D2
Expert	BnaC03g01540.1D2	BnaA03g01420.1D2
Expert	BnaC03g01570.1D2	BnaA03g01440.1D2
Expert	BnaC03g01580.1D2	BnaA03g01450.1D2
Expert	BnaC03g01610.1D2	BnaA03g01470.1D2
Expert	BnaC03g01640.1D2	BnaA03g07600.1D2
Expert	BnaC03g01670.1D2	BnaA03g07630.1D2
Expert	BnaC03g01680.1D2	BnaA03g07650.1D2
Expert	BnaC03g01790.1D2	BnaA03g07730.1D2
Expert	BnaC03g01820.1D2	BnaA03g02290.1D2
Expert	BnaC03g01840.1D2	BnaA03g01490.1D2
Expert	BnaC03g01860.1D2	BnaA03g01530.1D2
Expert	BnaC03g01900.1D2	BnaA03g01550.1D2
Expert	BnaC03g01920.1D2	BnaA03g01570.1D2
Expert	BnaC03g01950.1D2	BnaA03g01600.1D2
Expert	BnaC03g02010.1D2	BnaA03g01640.1D2
Expert	BnaC03g02030.1D2	BnaA03g01670.1D2
Expert	BnaC03g02030.1D2	BnaA03g01690.1D2
Expert	BnaC03g02040.1D2	BnaA03g01670.1D2
Expert	BnaC03g02040.1D2	BnaA03g01690.1D2
Expert	BnaC03g02050.1D2	BnaA03g01710.1D2
Expert	BnaC03g02070.1D2	BnaA03g01710.1D2
Expert	BnaC03g02090.1D2	BnaA03g01730.1D2
Expert	BnaC03g02180.1D2	BnaA03g01780.1D2
Expert	BnaC03g02200.1D2	BnaA03g01800.1D2
Expert	BnaC03g02220.1D2	BnaA03g01810.1D2
Expert	BnaC03g02240.1D2	BnaA03g01840.1D2
Expert	BnaC03g02270.1D2	BnaA03g01860.1D2
Expert	BnaC03g02300.1D2	BnaA03g01890.1D2
Expert	BnaC03g02300.1D2	BnaA03g01910.1D2
Expert	BnaC03g03890.1D2	BnaA03g01140.1D2
H149	BnaC03g00050.1D2	BnaA03g00130.1D2
H149	BnaC03g00080.1D2	BnaA03g00170.1D2
H149	BnaC03g00120.1D2	BnaA03g00210.1D2
H149	BnaC03g00140.1D2	BnaA03g00230.1D2
H149	BnaC03g00150.1D2	BnaA03g00240.1D2
H149	BnaC03g00160.1D2	BnaA03g00250.1D2
H149	BnaC03g00180.1D2	BnaA03g00270.1D2
H149	BnaC03g00250.1D2	BnaA03g00330.1D2
H149	BnaC03g00260.1D2	BnaA03g00340.1D2
H149	BnaC03g00270.1D2	BnaA03g00350.1D2
H149	BnaC03g00320.1D2	BnaA03g00400.1D2
H149	BnaC03g00360.1D2	BnaA03g00440.1D2
H149	BnaC03g00450.1D2	BnaA03g02010.1D2

H149	BnaC03g00460.1D2	BnaA03g02020.1D2
H149	BnaC03g00470.1D2	BnaA03g02030.1D2
H149	BnaC03g00500.1D2	BnaA03g02050.1D2
H149	BnaC03g00540.1D2	BnaA03g02090.1D2
H149	BnaC03g00560.1D2	BnaA03g02110.1D2
H149	BnaC03g00580.1D2	BnaA03g02250.1D2
H149	BnaC03g00610.1D2	BnaA03g02220.1D2
H149	BnaC03g00710.1D2	BnaA03g02140.1D2
H149	BnaC03g00720.1D2	BnaA03g02130.1D2
H149	BnaC03g00740.1D2	BnaA03g00540.1D2
H149	BnaC03g00820.1D2	BnaA03g00620.1D2
H149	BnaC03g00890.1D2	BnaA03g00680.1D2
H149	BnaC03g00910.1D2	BnaA03g00700.1D2
H149	BnaC03g00920.1D2	BnaA03g00710.1D2
H149	BnaC03g00940.1D2	BnaA03g00730.1D2
H149	BnaC03g00970.1D2	BnaA03g00760.1D2
H149	BnaC03g00990.1D2	BnaA03g00780.1D2
H149	BnaC03g01030.1D2	BnaA03g00820.1D2
H149	BnaC03g01050.1D2	BnaA03g00840.1D2
H149	BnaC03g01160.1D2	BnaA03g00980.1D2
H149	BnaC03g01230.1D2	BnaA03g01050.1D2
H149	BnaC03g01240.1D2	BnaA03g01060.1D2
H149	BnaC03g01250.1D2	BnaA03g01070.1D2
H149	BnaC03g01260.1D2	BnaA03g01080.1D2
H149	BnaC03g01320.1D2	BnaA03g01190.1D2
H149	BnaC03g01340.1D2	BnaA03g01200.1D2
H149	BnaC03g01360.1D2	BnaA03g01220.1D2
H149	BnaC03g01410.1D2	BnaA03g01290.1D2
H149	BnaC03g01420.1D2	BnaA03g01300.1D2
H149	BnaC03g01430.1D2	BnaA03g01310.1D2
H149	BnaC03g01450.1D2	BnaA03g01330.1D2
H149	BnaC03g01460.1D2	BnaA03g01340.1D2
H149	BnaC03g01470.1D2	BnaA03g01350.1D2
H149	BnaC03g01480.1D2	BnaA03g01360.1D2
H149	BnaC03g01490.1D2	BnaA03g01370.1D2
H149	BnaC03g01540.1D2	BnaA03g01420.1D2
H149	BnaC03g01580.1D2	BnaA03g01450.1D2
H149	BnaC03g01590.1D2	BnaA03g01450.1D2
H149	BnaC03g01610.1D2	BnaA03g01470.1D2
H149	BnaC03g01640.1D2	BnaA03g07600.1D2
H149	BnaC03g01670.1D2	BnaA03g07630.1D2
H149	BnaC03g01680.1D2	BnaA03g07650.1D2
H149	BnaC03g01790.1D2	BnaA03g07730.1D2
H149	BnaC03g01820.1D2	BnaA03g02290.1D2
H149	BnaC03g01840.1D2	BnaA03g01490.1D2
H149	BnaC03g01900.1D2	BnaA03g01550.1D2
H149	BnaC03g01950.1D2	BnaA03g01600.1D2
H149	BnaC03g02010.1D2	BnaA03g01640.1D2
H149	BnaC03g02030.1D2	BnaA03g01670.1D2
H149	BnaC03g02030.1D2	BnaA03g01690.1D2
H149	BnaC03g02040.1D2	BnaA03g01670.1D2

H149	BnaC03g02040.1D2	BnaA03g01690.1D2
H149	BnaC03g02070.1D2	BnaA03g01710.1D2
H149	BnaC03g02090.1D2	BnaA03g01730.1D2
H149	BnaC03g02180.1D2	BnaA03g01780.1D2
H149	BnaC03g02190.1D2	BnaA03g01790.1D2
H149	BnaC03g02200.1D2	BnaA03g01800.1D2
H149	BnaC03g02240.1D2	BnaA03g01840.1D2
H149	BnaC03g02270.1D2	BnaA03g01860.1D2
H149	BnaC03g02290.1D2	BnaA03g01880.1D2
H149	BnaC03g02300.1D2	BnaA03g01890.1D2
H149	BnaC03g02300.1D2	BnaA03g01910.1D2
H149	BnaC03g02380.1D2	BnaA03g07570.1D2
H149	BnaC03g02400.1D2	BnaA03g07560.1D2
H149	BnaC03g02430.1D2	BnaA03g07480.1D2
H149	BnaC03g02520.1D2	BnaA03g02540.1D2
H149	BnaC03g02530.1D2	BnaA03g02550.1D2
H149	BnaC03g02540.1D2	BnaA03g02560.1D2
H149	BnaC03g02550.1D2	BnaA03g02570.1D2
H149	BnaC03g02590.1D2	BnaA03g02590.1D2
H149	BnaC03g02610.1D2	BnaA03g02600.1D2
H149	BnaC03g02650.1D2	BnaA03g02610.1D2
H149	BnaC03g02650.1D2	BnaA03g02620.1D2
H149	BnaC03g02680.1D2	BnaA03g02660.1D2
H149	BnaC03g02700.1D2	BnaA03g02690.1D2
H149	BnaC03g02840.1D2	BnaA03g02840.1D2
H149	BnaC03g02850.1D2	BnaA03g02850.1D2
H149	BnaC03g02860.1D2	BnaA03g02860.1D2
H149	BnaC03g02890.1D2	BnaA03g02890.1D2
H149	BnaC03g02920.1D2	BnaA03g02940.1D2
H149	BnaC03g02990.1D2	BnaA03g03020.1D2
H149	BnaC03g03890.1D2	BnaA03g01140.1D2
H149	BnaC03g03900.1D2	BnaA03g01150.1D2
H149	BnaC03g03920.1D2	BnaA03g01170.1D2
H44	BnaC03g04080.1D2	BnaA03g03700.1D2
H44	BnaC03g04140.1D2	BnaA03g03760.1D2
H44	BnaC03g04160.1D2	BnaA03g03800.1D2
H44	BnaC03g04170.1D2	BnaA03g03790.1D2
H44	BnaC03g04180.1D2	BnaA03g03780.1D2
H44	BnaC03g04210.1D2	BnaA03g03830.1D2
H44	BnaC03g04240.1D2	BnaA03g03870.1D2
H44	BnaC03g04530.1D2	BnaA03g04080.1D2
H44	BnaC03g04610.1D2	BnaA03g04140.1D2
H44	BnaC03g04710.1D2	BnaA03g04250.1D2
H44	BnaC03g04730.1D2	BnaA03g04270.1D2
H44	BnaC03g04760.1D2	BnaA03g04300.1D2
H44	BnaC03g04900.1D2	BnaA03g04400.1D2
H44	BnaC03g04950.1D2	BnaA03g04450.1D2
H44	BnaC03g04990.1D2	BnaA03g04450.1D2
H44	BnaC03g05100.1D2	BnaA03g04580.1D2
H44	BnaC03g05430.1D2	BnaA03g04930.1D2
H44	BnaC03g05520.1D2	BnaA03g05020.1D2

H44	BnaC03g05700.1D2	BnaA03g05190.1D2
H44	BnaC03g05760.1D2	BnaA03g05240.1D2
H44	BnaC03g05840.1D2	BnaA03g05300.1D2
H44	BnaC03g05950.1D2	BnaA03g05410.1D2
H44	BnaC03g05960.1D2	BnaA03g05420.1D2
H44	BnaC03g06130.1D2	BnaA03g05610.1D2
H44	BnaC03g06180.1D2	BnaA03g05660.1D2
H44	BnaC03g06200.1D2	BnaA03g05670.1D2
H44	BnaC03g06380.1D2	BnaA03g05780.1D2
H44	BnaC03g06500.1D2	BnaA03g05880.1D2
H44	BnaC03g06560.1D2	BnaA03g05910.1D2
H44	BnaC03g06600.1D2	BnaA03g06030.1D2
H44	BnaC03g06620.1D2	BnaA03g05920.1D2
H44	BnaC03g06630.1D2	BnaA03g05920.1D2
H44	BnaC03g06640.1D2	BnaA03g05940.1D2
H44	BnaC03g06650.1D2	BnaA03g05950.1D2
H44	BnaC03g06700.1D2	BnaA03g06050.1D2
H44	BnaC03g06720.1D2	BnaA03g06070.1D2
H44	BnaC03g06790.1D2	BnaA03g06130.1D2
H44	BnaC03g06800.1D2	BnaA03g06130.1D2
H44	BnaC03g06880.1D2	BnaA03g06210.1D2
H44	BnaC03g06900.1D2	BnaA03g06230.1D2
H44	BnaC03g06960.1D2	BnaA03g06300.1D2
H44	BnaC03g07010.1D2	BnaA03g06370.1D2
H44	BnaC03g07120.1D2	BnaA03g06460.1D2
H44	BnaC03g07170.1D2	BnaA03g06520.1D2
H44	BnaC03g07520.1D2	BnaA03g06800.1D2
H44	BnaC03g07530.1D2	BnaA03g06810.1D2
H44	BnaC03g07550.1D2	BnaA03g06830.1D2
H44	BnaC03g07610.1D2	BnaA03g06890.1D2
H44	BnaC03g07720.1D2	BnaA03g06970.1D2
H44	BnaC03g07780.1D2	BnaA03g07050.1D2
H44	BnaC03g07820.1D2	BnaA03g08030.1D2
H44	BnaC03g07870.1D2	BnaA03g08080.1D2
H44	BnaC03g08020.1D2	BnaA03g08230.1D2
H44	BnaC03g08050.1D2	BnaA03g08250.1D2
H44	BnaC03g10900.1D2	BnaA03g04750.1D2
H44	BnaC03g10930.1D2	BnaA03g04730.1D2
H44	BnaC03g10940.1D2	BnaA03g04720.1D2
H44	BnaC03g19230.1D2	BnaA03g18320.1D2
H44	BnaC03g20320.1D2	BnaA03g18360.1D2
H44	BnaC03g23460.1D2	BnaA03g20970.1D2
H44	BnaC03g46790.1D2	BnaA03g20750.1D2
Pirola	BnaC03g00050.1D2	BnaA03g00130.1D2
Pirola	BnaC03g00080.1D2	BnaA03g00170.1D2
Pirola	BnaC03g00120.1D2	BnaA03g00210.1D2
Pirola	BnaC03g00140.1D2	BnaA03g00230.1D2
Pirola	BnaC03g00150.1D2	BnaA03g00240.1D2
Pirola	BnaC03g00160.1D2	BnaA03g00250.1D2
Pirola	BnaC03g00180.1D2	BnaA03g00270.1D2
Pirola	BnaC03g00250.1D2	BnaA03g00330.1D2

Pirola	BnaC03g00260.1D2	BnaA03g00340.1D2
Pirola	BnaC03g00270.1D2	BnaA03g00350.1D2
Pirola	BnaC03g00280.1D2	BnaA03g00360.1D2
Pirola	BnaC03g00320.1D2	BnaA03g00400.1D2
Pirola	BnaC03g00330.1D2	BnaA03g00410.1D2
Pirola	BnaC03g00360.1D2	BnaA03g00440.1D2
Pirola	BnaC03g00420.1D2	BnaA03g00490.1D2
Pirola	BnaC03g00450.1D2	BnaA03g02010.1D2
Pirola	BnaC03g00460.1D2	BnaA03g02020.1D2
Pirola	BnaC03g00470.1D2	BnaA03g02030.1D2
Pirola	BnaC03g00500.1D2	BnaA03g02050.1D2
Pirola	BnaC03g00520.1D2	BnaA03g02070.1D2
Pirola	BnaC03g00560.1D2	BnaA03g02110.1D2
Pirola	BnaC03g00580.1D2	BnaA03g02250.1D2
Pirola	BnaC03g00610.1D2	BnaA03g02220.1D2
Pirola	BnaC03g00670.1D2	BnaA03g02170.1D2
Pirola	BnaC03g00710.1D2	BnaA03g02140.1D2
Pirola	BnaC03g00720.1D2	BnaA03g02130.1D2
Pirola	BnaC03g00740.1D2	BnaA03g00540.1D2
Pirola	BnaC03g00820.1D2	BnaA03g00620.1D2
Pirola	BnaC03g00830.1D2	BnaA03g00630.1D2
Pirola	BnaC03g00840.1D2	BnaA03g00640.1D2
Pirola	BnaC03g00850.1D2	BnaA03g00650.1D2
Pirola	BnaC03g00910.1D2	BnaA03g00700.1D2
Pirola	BnaC03g00920.1D2	BnaA03g00710.1D2
Pirola	BnaC03g00940.1D2	BnaA03g00730.1D2
Pirola	BnaC03g00960.1D2	BnaA03g00750.1D2
Pirola	BnaC03g00970.1D2	BnaA03g00760.1D2
Pirola	BnaC03g00990.1D2	BnaA03g00780.1D2
Pirola	BnaC03g01030.1D2	BnaA03g00820.1D2
Pirola	BnaC03g01050.1D2	BnaA03g00840.1D2
Pirola	BnaC03g01060.1D2	BnaA03g00850.1D2
Pirola	BnaC03g01160.1D2	BnaA03g00980.1D2
Pirola	BnaC03g01230.1D2	BnaA03g01050.1D2
Pirola	BnaC03g01240.1D2	BnaA03g01060.1D2
Pirola	BnaC03g01250.1D2	BnaA03g01070.1D2
Pirola	BnaC03g01260.1D2	BnaA03g01080.1D2
Pirola	BnaC03g01360.1D2	BnaA03g01220.1D2
Pirola	BnaC03g01420.1D2	BnaA03g01300.1D2
Pirola	BnaC03g01450.1D2	BnaA03g01330.1D2
Pirola	BnaC03g01460.1D2	BnaA03g01340.1D2
Pirola	BnaC03g01470.1D2	BnaA03g01350.1D2
Pirola	BnaC03g01480.1D2	BnaA03g01360.1D2
Pirola	BnaC03g01490.1D2	BnaA03g01370.1D2
Pirola	BnaC03g01530.1D2	BnaA03g01410.1D2
Pirola	BnaC03g01570.1D2	BnaA03g01440.1D2
Pirola	BnaC03g01580.1D2	BnaA03g01450.1D2
Pirola	BnaC03g01640.1D2	BnaA03g07600.1D2
Pirola	BnaC03g01670.1D2	BnaA03g07630.1D2
Pirola	BnaC03g01680.1D2	BnaA03g07650.1D2
Pirola	BnaC03g01790.1D2	BnaA03g07730.1D2

Pirola	BnaC03g01820.1D2	BnaA03g02290.1D2
Pirola	BnaC03g01840.1D2	BnaA03g01490.1D2
Pirola	BnaC03g01900.1D2	BnaA03g01550.1D2
Pirola	BnaC03g01920.1D2	BnaA03g01570.1D2
Pirola	BnaC03g01950.1D2	BnaA03g01600.1D2
Pirola	BnaC03g02010.1D2	BnaA03g01640.1D2
Pirola	BnaC03g02030.1D2	BnaA03g01670.1D2
Pirola	BnaC03g02030.1D2	BnaA03g01690.1D2
Pirola	BnaC03g02040.1D2	BnaA03g01670.1D2
Pirola	BnaC03g02040.1D2	BnaA03g01690.1D2
Pirola	BnaC03g02070.1D2	BnaA03g01710.1D2
Pirola	BnaC03g02090.1D2	BnaA03g01730.1D2
Pirola	BnaC03g02180.1D2	BnaA03g01780.1D2
Pirola	BnaC03g02190.1D2	BnaA03g01790.1D2
Pirola	BnaC03g02200.1D2	BnaA03g01800.1D2
Pirola	BnaC03g02220.1D2	BnaA03g01810.1D2
Pirola	BnaC03g02240.1D2	BnaA03g01840.1D2
Pirola	BnaC03g02270.1D2	BnaA03g01860.1D2
Pirola	BnaC03g02290.1D2	BnaA03g01880.1D2
Pirola	BnaC03g02300.1D2	BnaA03g01890.1D2
Pirola	BnaC03g02300.1D2	BnaA03g01910.1D2
Pirola	BnaC03g02380.1D2	BnaA03g07570.1D2
Pirola	BnaC03g02400.1D2	BnaA03g07560.1D2
Pirola	BnaC03g02430.1D2	BnaA03g07480.1D2
Pirola	BnaC03g02510.1D2	BnaA03g02530.1D2
Pirola	BnaC03g02520.1D2	BnaA03g02540.1D2
Pirola	BnaC03g02530.1D2	BnaA03g02550.1D2
Pirola	BnaC03g02540.1D2	BnaA03g02560.1D2
Pirola	BnaC03g02610.1D2	BnaA03g02600.1D2
Pirola	BnaC03g02650.1D2	BnaA03g02610.1D2
Pirola	BnaC03g02650.1D2	BnaA03g02620.1D2
Pirola	BnaC03g02660.1D2	BnaA03g02610.1D2
Pirola	BnaC03g02660.1D2	BnaA03g02620.1D2
Pirola	BnaC03g02660.1D2	BnaA03g08180.1D2
Pirola	BnaC03g02680.1D2	BnaA03g02660.1D2
Pirola	BnaC03g02700.1D2	BnaA03g02690.1D2
Pirola	BnaC03g02780.1D2	BnaA03g02780.1D2
Pirola	BnaC03g02840.1D2	BnaA03g02840.1D2
Pirola	BnaC03g02850.1D2	BnaA03g02850.1D2
Pirola	BnaC03g02860.1D2	BnaA03g02860.1D2
Pirola	BnaC03g02880.1D2	BnaA03g02880.1D2
Pirola	BnaC03g02890.1D2	BnaA03g02890.1D2
Pirola	BnaC03g02920.1D2	BnaA03g02940.1D2
Pirola	BnaC03g02990.1D2	BnaA03g03020.1D2
Pirola	BnaC03g03000.1D2	BnaA03g03030.1D2
Pirola	BnaC03g03020.1D2	BnaA03g03050.1D2
Pirola	BnaC03g03030.1D2	BnaA03g03060.1D2
Pirola	BnaC03g03050.1D2	BnaA03g07820.1D2
Pirola	BnaC03g03120.1D2	BnaA03g07330.1D2
Pirola	BnaC03g03130.1D2	BnaA03g07340.1D2
Pirola	BnaC03g03150.1D2	BnaA03g07360.1D2

Pirola	BnaC03g03230.1D2	BnaA03g03160.1D2
Pirola	BnaC03g03250.1D2	BnaA03g03180.1D2
Pirola	BnaC03g03280.1D2	BnaA03g03220.1D2
Pirola	BnaC03g03280.1D2	BnaA03g03230.1D2
Pirola	BnaC03g03290.1D2	BnaA03g03240.1D2
Pirola	BnaC03g03300.1D2	BnaA03g03250.1D2
Pirola	BnaC03g03320.1D2	BnaA03g03270.1D2
Pirola	BnaC03g03330.1D2	BnaA03g03280.1D2
Pirola	BnaC03g03350.1D2	BnaA03g03300.1D2
Pirola	BnaC03g03400.1D2	BnaA03g07260.1D2
Pirola	BnaC03g03410.1D2	BnaA03g07250.1D2
Pirola	BnaC03g03420.1D2	BnaA03g07240.1D2
Pirola	BnaC03g03440.1D2	BnaA03g07220.1D2
Pirola	BnaC03g03470.1D2	BnaA03g07200.1D2
Pirola	BnaC03g03500.1D2	BnaA03g07180.1D2
Pirola	BnaC03g03510.1D2	BnaA03g07170.1D2
Pirola	BnaC03g03570.1D2	BnaA03g03320.1D2
Pirola	BnaC03g03590.1D2	BnaA03g03370.1D2
Pirola	BnaC03g03620.1D2	BnaA03g03390.1D2
Pirola	BnaC03g03630.1D2	BnaA03g03400.1D2
Pirola	BnaC03g03700.1D2	BnaA03g03440.1D2
Pirola	BnaC03g03710.1D2	BnaA03g03450.1D2
Pirola	BnaC03g03730.1D2	BnaA03g03470.1D2
Pirola	BnaC03g03740.1D2	BnaA03g03480.1D2
Pirola	BnaC03g03750.1D2	BnaA03g03490.1D2
Pirola	BnaC03g03750.1D2	BnaA03g03790.1D2
Pirola	BnaC03g03760.1D2	BnaA03g03500.1D2
Pirola	BnaC03g03800.1D2	BnaA03g03530.1D2
Pirola	BnaC03g03820.1D2	BnaA03g03550.1D2
Pirola	BnaC03g03920.1D2	BnaA03g01170.1D2
Pirola	BnaC03g03970.1D2	BnaA03g03620.1D2
Pirola	BnaC03g04050.1D2	BnaA03g03670.1D2
Pirola	BnaC03g04060.1D2	BnaA03g03680.1D2
Pirola	BnaC03g04080.1D2	BnaA03g03700.1D2
Pirola	BnaC03g04160.1D2	BnaA03g03800.1D2
Pirola	BnaC03g04170.1D2	BnaA03g03490.1D2
Pirola	BnaC03g04170.1D2	BnaA03g03790.1D2
Pirola	BnaC03g04180.1D2	BnaA03g03780.1D2
Pirola	BnaC03g04210.1D2	BnaA03g03830.1D2
Pirola	BnaC03g04220.1D2	BnaA03g03840.1D2
Pirola	BnaC03g04240.1D2	BnaA03g03870.1D2
Pirola	BnaC03g04390.1D2	BnaA03g03970.1D2
Pirola	BnaC03g04450.1D2	BnaA03g04010.1D2
Pirola	BnaC03g04460.1D2	BnaA03g04020.1D2
Pirola	BnaC03g04530.1D2	BnaA03g04080.1D2
Pirola	BnaC03g04560.1D2	BnaA03g04100.1D2
Pirola	BnaC03g04570.1D2	BnaA03g04110.1D2
Pirola	BnaC03g04580.1D2	BnaA03g04120.1D2
Pirola	BnaC03g04600.1D2	BnaA03g04130.1D2
Pirola	BnaC03g04610.1D2	BnaA03g04140.1D2
Pirola	BnaC03g04620.1D2	BnaA03g04150.1D2

Pirola	BnaC03g04630.1D2	BnaA03g04160.1D2
Pirola	BnaC03g04660.1D2	BnaA03g04200.1D2
Pirola	BnaC03g04670.1D2	BnaA03g04210.1D2
Pirola	BnaC03g04730.1D2	BnaA03g04270.1D2
Pirola	BnaC03g04760.1D2	BnaA03g04300.1D2
Pirola	BnaC03g04800.1D2	BnaA03g04330.1D2
Pirola	BnaC03g04820.1D2	BnaA03g04350.1D2
Pirola	BnaC03g04830.1D2	BnaA03g04360.1D2
Pirola	BnaC03g04890.1D2	BnaA03g04390.1D2
Pirola	BnaC03g04900.1D2	BnaA03g04400.1D2
Pirola	BnaC03g04990.1D2	BnaA03g04450.1D2
Pirola	BnaC03g05070.1D2	BnaA03g04540.1D2
Pirola	BnaC03g05100.1D2	BnaA03g04580.1D2
Pirola	BnaC03g05180.1D2	BnaA03g04650.1D2
Pirola	BnaC03g05190.1D2	BnaA03g04660.1D2
Pirola	BnaC03g05210.1D2	BnaA03g04680.1D2
Pirola	BnaC03g05220.1D2	BnaA03g04690.1D2
Pirola	BnaC03g05260.1D2	BnaA03g04790.1D2
Pirola	BnaC03g05360.1D2	BnaA03g04880.1D2
Pirola	BnaC03g05430.1D2	BnaA03g04930.1D2
Pirola	BnaC03g05440.1D2	BnaA03g04940.1D2
Pirola	BnaC03g05460.1D2	BnaA03g04960.1D2
Pirola	BnaC03g05510.1D2	BnaA03g05010.1D2
Pirola	BnaC03g05520.1D2	BnaA03g05020.1D2
Pirola	BnaC03g05530.1D2	BnaA03g05030.1D2
Pirola	BnaC03g05640.1D2	BnaA03g05120.1D2
Pirola	BnaC03g05680.1D2	BnaA03g05160.1D2
Pirola	BnaC03g05700.1D2	BnaA03g05190.1D2
Pirola	BnaC03g05840.1D2	BnaA03g05300.1D2
Pirola	BnaC03g05890.1D2	BnaA03g05340.1D2
Pirola	BnaC03g05950.1D2	BnaA03g05410.1D2
Pirola	BnaC03g05960.1D2	BnaA03g05420.1D2
Pirola	BnaC03g06050.1D2	BnaA03g05550.1D2
Pirola	BnaC03g06130.1D2	BnaA03g05610.1D2
Pirola	BnaC03g06200.1D2	BnaA03g05670.1D2
Pirola	BnaC03g06210.1D2	BnaA03g05680.1D2
Pirola	BnaC03g10900.1D2	BnaA03g04750.1D2
Pirola	BnaC03g10930.1D2	BnaA03g04730.1D2
Pirola	BnaC03g10940.1D2	BnaA03g04720.1D2
Pirola	BnaC03g10970.1D2	BnaA03g04700.1D2
RS_4_6	BnaC03g00320.1D2	BnaA03g00400.1D2
RS_4_6	BnaC03g00360.1D2	BnaA03g00440.1D2
RS_4_6	BnaC03g00450.1D2	BnaA03g02010.1D2
RS_4_6	BnaC03g00470.1D2	BnaA03g02030.1D2
RS_4_6	BnaC03g00520.1D2	BnaA03g02070.1D2
RS_4_6	BnaC03g00530.1D2	BnaA03g02080.1D2
RS_4_6	BnaC03g00560.1D2	BnaA03g02110.1D2
RS_4_6	BnaC03g00740.1D2	BnaA03g00540.1D2
RS_4_6	BnaC03g00850.1D2	BnaA03g00650.1D2
RS_4_6	BnaC03g00890.1D2	BnaA03g00680.1D2
RS_4_6	BnaC03g01030.1D2	BnaA03g00820.1D2

RS_4_6	BnaC03g01230.1D2	BnaA03g01050.1D2
RS_4_6	BnaC03g01240.1D2	BnaA03g01060.1D2
RS_4_6	BnaC03g01360.1D2	BnaA03g01220.1D2
RS_4_6	BnaC03g01410.1D2	BnaA03g01290.1D2
RS_4_6	BnaC03g01420.1D2	BnaA03g01300.1D2
RS_4_6	BnaC03g01450.1D2	BnaA03g01330.1D2
RS_4_6	BnaC03g01490.1D2	BnaA03g01370.1D2
RS_4_6	BnaC03g01540.1D2	BnaA03g01420.1D2
RS_4_6	BnaC03g01570.1D2	BnaA03g01440.1D2
RS_4_6	BnaC03g01790.1D2	BnaA03g07730.1D2
RS_4_6	BnaC03g01820.1D2	BnaA03g02290.1D2
RS_4_6	BnaC03g01860.1D2	BnaA03g01530.1D2
RS_4_6	BnaC03g01900.1D2	BnaA03g01550.1D2
RS_4_6	BnaC03g02090.1D2	BnaA03g01730.1D2
RS_4_6	BnaC03g02180.1D2	BnaA03g01780.1D2
RS_4_6	BnaC03g02240.1D2	BnaA03g01840.1D2
RS_4_6	BnaC03g02520.1D2	BnaA03g02540.1D2
RS_4_6	BnaC03g02600.1D2	BnaA03g02600.1D2
RS_4_6	BnaC03g02610.1D2	BnaA03g02600.1D2
RS_4_6	BnaC03g02650.1D2	BnaA03g02610.1D2
RS_4_6	BnaC03g02650.1D2	BnaA03g02620.1D2
RS_4_6	BnaC03g02680.1D2	BnaA03g02660.1D2
RS_4_6	BnaC03g03110.1D2	BnaA03g07320.1D2
RS_4_6	BnaC03g03130.1D2	BnaA03g07340.1D2
RS_4_6	BnaC03g03270.1D2	BnaA03g03220.1D2
RS_4_6	BnaC03g03270.1D2	BnaA03g03230.1D2
RS_4_6	BnaC03g03280.1D2	BnaA03g03220.1D2
RS_4_6	BnaC03g03280.1D2	BnaA03g03230.1D2
RS_4_6	BnaC03g03410.1D2	BnaA03g07250.1D2
RS_4_6	BnaC03g03470.1D2	BnaA03g07200.1D2
RS_4_6	BnaC03g03630.1D2	BnaA03g03400.1D2
RS_4_6	BnaC03g03690.1D2	BnaA03g03430.1D2
RS_4_6	BnaC03g03750.1D2	BnaA03g03490.1D2
RS_4_6	BnaC03g03750.1D2	BnaA03g03790.1D2
RS_4_6	BnaC03g03890.1D2	BnaA03g01140.1D2
RS_4_6	BnaC03g03900.1D2	BnaA03g01150.1D2
RS_4_6	BnaC03g03920.1D2	BnaA03g01170.1D2
RS_4_6	BnaC03g04170.1D2	BnaA03g03490.1D2
RS_4_6	BnaC03g04170.1D2	BnaA03g03790.1D2
RS_4_6	BnaC03g04210.1D2	BnaA03g03830.1D2
RS_4_6	BnaC03g04220.1D2	BnaA03g03840.1D2
Sensation	BnaC03g00080.1D2	BnaA03g00170.1D2
Sensation	BnaC03g00100.1D2	BnaA03g00190.1D2
Sensation	BnaC03g00120.1D2	BnaA03g00210.1D2
Sensation	BnaC03g00140.1D2	BnaA03g00230.1D2
Sensation	BnaC03g00150.1D2	BnaA03g00240.1D2
Sensation	BnaC03g00160.1D2	BnaA03g00250.1D2
Sensation	BnaC03g00180.1D2	BnaA03g00270.1D2
Sensation	BnaC03g00320.1D2	BnaA03g00400.1D2
Sensation	BnaC03g00340.1D2	BnaA03g00420.1D2
Sensation	BnaC03g00360.1D2	BnaA03g00440.1D2

Sensation	BnaC03g00420.1D2	BnaA03g00490.1D2
Sensation	BnaC03g00450.1D2	BnaA03g02010.1D2
Sensation	BnaC03g00460.1D2	BnaA03g02020.1D2
Sensation	BnaC03g00470.1D2	BnaA03g02030.1D2
Sensation	BnaC03g00520.1D2	BnaA03g02070.1D2
Sensation	BnaC03g00530.1D2	BnaA03g02080.1D2
Sensation	BnaC03g00560.1D2	BnaA03g02110.1D2
Sensation	BnaC03g00610.1D2	BnaA03g02220.1D2
Sensation	BnaC03g00720.1D2	BnaA03g02130.1D2
Sensation	BnaC03g00890.1D2	BnaA03g00680.1D2
Sensation	BnaC03g00920.1D2	BnaA03g00710.1D2
Sensation	BnaC03g00970.1D2	BnaA03g00760.1D2
Sensation	BnaC03g01160.1D2	BnaA03g00980.1D2
Sensation	BnaC03g01170.1D2	BnaA03g00990.1D2
Sensation	BnaC03g01220.1D2	BnaA03g01040.1D2
Sensation	BnaC03g01260.1D2	BnaA03g01080.1D2
Sensation	BnaC03g01270.1D2	BnaA03g01090.1D2
Sensation	BnaC03g01420.1D2	BnaA03g01300.1D2
Sensation	BnaC03g01450.1D2	BnaA03g01330.1D2
Sensation	BnaC03g01480.1D2	BnaA03g01360.1D2
Sensation	BnaC03g01490.1D2	BnaA03g01370.1D2
Sensation	BnaC03g01530.1D2	BnaA03g01410.1D2
Sensation	BnaC03g01540.1D2	BnaA03g01420.1D2
Sensation	BnaC03g01570.1D2	BnaA03g01440.1D2
Sensation	BnaC03g01580.1D2	BnaA03g01450.1D2
Sensation	BnaC03g01790.1D2	BnaA03g07730.1D2
Sensation	BnaC03g01820.1D2	BnaA03g02290.1D2
Sensation	BnaC03g01900.1D2	BnaA03g01550.1D2
Sensation	BnaC03g01920.1D2	BnaA03g01570.1D2
Sensation	BnaC03g01950.1D2	BnaA03g01600.1D2
Sensation	BnaC03g02010.1D2	BnaA03g01640.1D2
Sensation	BnaC03g02190.1D2	BnaA03g01790.1D2
Sensation	BnaC03g02200.1D2	BnaA03g01800.1D2
Sensation	BnaC03g02240.1D2	BnaA03g01840.1D2
Sensation	BnaC03g02290.1D2	BnaA03g01880.1D2
Sensation	BnaC03g02300.1D2	BnaA03g01890.1D2
Sensation	BnaC03g02300.1D2	BnaA03g01910.1D2
Sensation	BnaC03g02380.1D2	BnaA03g07570.1D2
Sensation	BnaC03g02400.1D2	BnaA03g07560.1D2
Sensation	BnaC03g02430.1D2	BnaA03g07480.1D2
Sensation	BnaC03g02610.1D2	BnaA03g02600.1D2
Sensation	BnaC03g02680.1D2	BnaA03g02660.1D2
Sensation	BnaC03g02890.1D2	BnaA03g02890.1D2
Sensation	BnaC03g02920.1D2	BnaA03g02940.1D2
Sensation	BnaC03g03280.1D2	BnaA03g03220.1D2
Sensation	BnaC03g03280.1D2	BnaA03g03230.1D2
Sensation	BnaC03g03330.1D2	BnaA03g03280.1D2
Sensation	BnaC03g03350.1D2	BnaA03g03300.1D2
Sensation	BnaC03g03420.1D2	BnaA03g07240.1D2
Sensation	BnaC03g03440.1D2	BnaA03g07220.1D2
Sensation	BnaC03g03590.1D2	BnaA03g03370.1D2

Sensation	BnaC03g03630.1D2	BnaA03g03400.1D2
Sensation	BnaC03g03690.1D2	BnaA03g03430.1D2
Sensation	BnaC03g03750.1D2	BnaA03g03490.1D2
Sensation	BnaC03g03750.1D2	BnaA03g03790.1D2
Sensation	BnaC03g04170.1D2	BnaA03g03490.1D2
Sensation	BnaC03g04170.1D2	BnaA03g03790.1D2
Sensation	BnaC03g04180.1D2	BnaA03g03780.1D2
Sensation	BnaC03g04210.1D2	BnaA03g03830.1D2
Sensation	BnaC03g04220.1D2	BnaA03g03840.1D2
Sensation	BnaC03g04330.1D2	BnaA03g03920.1D2
Sensation	BnaC03g04390.1D2	BnaA03g03970.1D2
Sensation	BnaC03g04420.1D2	BnaA03g04000.1D2
Sensation	BnaC03g04450.1D2	BnaA03g04010.1D2
Sensation	BnaC03g04460.1D2	BnaA03g04020.1D2
Sensation	BnaC03g04500.1D2	BnaA03g04050.1D2
Sensation	BnaC03g04530.1D2	BnaA03g04080.1D2
Sensation	BnaC03g04560.1D2	BnaA03g04100.1D2
Sensation	BnaC03g04570.1D2	BnaA03g04110.1D2
Sensation	BnaC03g04580.1D2	BnaA03g04120.1D2
Sensation	BnaC03g04600.1D2	BnaA03g04130.1D2
Sensation	BnaC03g04610.1D2	BnaA03g04140.1D2
Sensation	BnaC03g04630.1D2	BnaA03g04160.1D2
Sensation	BnaC03g04710.1D2	BnaA03g04250.1D2
Sensation	BnaC03g04730.1D2	BnaA03g04270.1D2
Sensation	BnaC03g04760.1D2	BnaA03g04300.1D2
Sensation	BnaC03g04780.1D2	BnaA03g04320.1D2
Sensation	BnaC03g04900.1D2	BnaA03g04400.1D2
Sensation	BnaC03g04990.1D2	BnaA03g04450.1D2
Sensation	BnaC03g05070.1D2	BnaA03g04540.1D2
Sensation	BnaC03g05100.1D2	BnaA03g04580.1D2
Sensation	BnaC03g05180.1D2	BnaA03g04650.1D2
Sensation	BnaC03g05190.1D2	BnaA03g04660.1D2
Sensation	BnaC03g05430.1D2	BnaA03g04930.1D2
Sensation	BnaC03g05440.1D2	BnaA03g04940.1D2
Sensation	BnaC03g05460.1D2	BnaA03g04960.1D2
Sensation	BnaC03g05470.1D2	BnaA03g04970.1D2
Sensation	BnaC03g05510.1D2	BnaA03g05010.1D2
Sensation	BnaC03g05520.1D2	BnaA03g05020.1D2
Sensation	BnaC03g05530.1D2	BnaA03g05030.1D2
Sensation	BnaC03g05640.1D2	BnaA03g05120.1D2
Sensation	BnaC03g05650.1D2	BnaA03g05130.1D2
Sensation	BnaC03g05660.1D2	BnaA03g05140.1D2
Sensation	BnaC03g05680.1D2	BnaA03g05160.1D2
Sensation	BnaC03g05830.1D2	BnaA03g05290.1D2
Sensation	BnaC03g05840.1D2	BnaA03g05300.1D2
Sensation	BnaC03g05860.1D2	BnaA03g05320.1D2
Sensation	BnaC03g05960.1D2	BnaA03g05420.1D2
Sensation	BnaC03g05980.1D2	BnaA03g05440.1D2
Sensation	BnaC03g05990.1D2	BnaA03g05450.1D2
Sensation	BnaC03g06010.1D2	BnaA03g05510.1D2
Sensation	BnaC03g06120.1D2	BnaA03g05610.1D2

Sensation	BnaC03g06130.1D2	BnaA03g05610.1D2
Sensation	BnaC03g06150.1D2	BnaA03g05630.1D2
Sensation	BnaC03g06180.1D2	BnaA03g05660.1D2
Sensation	BnaC03g06200.1D2	BnaA03g05670.1D2
Sensation	BnaC03g06380.1D2	BnaA03g05780.1D2
Sensation	BnaC03g06390.1D2	BnaA03g05790.1D2
Sensation	BnaC03g06470.1D2	BnaA03g05850.1D2
Sensation	BnaC03g06500.1D2	BnaA03g05880.1D2
Sensation	BnaC03g06520.1D2	BnaA03g05900.1D2
Sensation	BnaC03g06560.1D2	BnaA03g05910.1D2
Sensation	BnaC03g06600.1D2	BnaA03g06030.1D2
Sensation	BnaC03g06610.1D2	BnaA03g05910.1D2
Sensation	BnaC03g06620.1D2	BnaA03g05920.1D2
Sensation	BnaC03g06630.1D2	BnaA03g05920.1D2
Sensation	BnaC03g06640.1D2	BnaA03g05940.1D2
Sensation	BnaC03g06650.1D2	BnaA03g05950.1D2
Sensation	BnaC03g06670.1D2	BnaA03g06020.1D2
Sensation	BnaC03g06680.1D2	BnaA03g06030.1D2
Sensation	BnaC03g06690.1D2	BnaA03g06040.1D2
Sensation	BnaC03g06700.1D2	BnaA03g06050.1D2
Sensation	BnaC03g06720.1D2	BnaA03g06070.1D2
Sensation	BnaC03g06770.1D2	BnaA03g06110.1D2
Sensation	BnaC03g06780.1D2	BnaA03g06120.1D2
Sensation	BnaC03g06790.1D2	BnaA03g06130.1D2
Sensation	BnaC03g06800.1D2	BnaA03g06130.1D2
Sensation	BnaC03g06880.1D2	BnaA03g06210.1D2
Sensation	BnaC03g06900.1D2	BnaA03g06230.1D2
Sensation	BnaC03g06930.1D2	BnaA03g06270.1D2
Sensation	BnaC03g06940.1D2	BnaA03g06280.1D2
Sensation	BnaC03g07120.1D2	BnaA03g06460.1D2
Sensation	BnaC03g07430.1D2	BnaA03g06680.1D2
Sensation	BnaC03g07480.1D2	BnaA03g06760.1D2
Sensation	BnaC03g07520.1D2	BnaA03g06800.1D2
Sensation	BnaC03g07530.1D2	BnaA03g06810.1D2
Sensation	BnaC03g07630.1D2	BnaA03g06910.1D2
Sensation	BnaC03g07980.1D2	BnaA03g08190.1D2
Sensation	BnaC03g08050.1D2	BnaA03g08250.1D2
Sensation	BnaC03g08180.1D2	BnaA03g08350.1D2
Sensation	BnaC03g08270.1D2	BnaA03g08420.1D2
Sensation	BnaC03g08300.1D2	BnaA03g08450.1D2
Sensation	BnaC03g08380.1D2	BnaA03g08510.1D2
Sensation	BnaC03g08440.1D2	BnaA03g08560.1D2
Sensation	BnaC03g08460.1D2	BnaA03g08580.1D2
Sensation	BnaC03g08550.1D2	BnaA03g08680.1D2
Sensation	BnaC03g08560.1D2	BnaA03g08690.1D2
Sensation	BnaC03g10940.1D2	BnaA03g04720.1D2
Sensation	BnaC03g11050.1D2	BnaA03g08510.1D2
Sensation	BnaC03g11210.1D2	BnaA03g08500.1D2
Sensation	BnaC03g11270.1D2	BnaA03g08500.1D2
Tapidor	BnaC03g00100.1D2	BnaA03g00190.1D2
Tapidor	BnaC03g00120.1D2	BnaA03g00210.1D2

Tapidor	BnaC03g00140.1D2	BnaA03g00230.1D2
Tapidor	BnaC03g00150.1D2	BnaA03g00240.1D2
Tapidor	BnaC03g00160.1D2	BnaA03g00250.1D2
Tapidor	BnaC03g00270.1D2	BnaA03g00350.1D2
Tapidor	BnaC03g00280.1D2	BnaA03g00360.1D2
Tapidor	BnaC03g00320.1D2	BnaA03g00400.1D2
Tapidor	BnaC03g00360.1D2	BnaA03g00440.1D2
Tapidor	BnaC03g00420.1D2	BnaA03g00490.1D2
Tapidor	BnaC03g00450.1D2	BnaA03g02010.1D2
Tapidor	BnaC03g00460.1D2	BnaA03g02020.1D2
Tapidor	BnaC03g00470.1D2	BnaA03g02030.1D2
Tapidor	BnaC03g00500.1D2	BnaA03g02050.1D2
Tapidor	BnaC03g00520.1D2	BnaA03g02070.1D2
Tapidor	BnaC03g00530.1D2	BnaA03g02080.1D2
Tapidor	BnaC03g00540.1D2	BnaA03g02090.1D2
Tapidor	BnaC03g00550.1D2	BnaA03g02100.1D2
Tapidor	BnaC03g00560.1D2	BnaA03g02110.1D2
Tapidor	BnaC03g00580.1D2	BnaA03g02250.1D2
Tapidor	BnaC03g00610.1D2	BnaA03g02220.1D2
Tapidor	BnaC03g00710.1D2	BnaA03g02140.1D2
Tapidor	BnaC03g00740.1D2	BnaA03g00540.1D2
Tapidor	BnaC03g00820.1D2	BnaA03g00620.1D2
Tapidor	BnaC03g00830.1D2	BnaA03g00630.1D2
Tapidor	BnaC03g00850.1D2	BnaA03g00650.1D2
Tapidor	BnaC03g00920.1D2	BnaA03g00710.1D2
Tapidor	BnaC03g01160.1D2	BnaA03g00980.1D2
Tapidor	BnaC03g01230.1D2	BnaA03g01050.1D2
Tapidor	BnaC03g01240.1D2	BnaA03g01060.1D2
Tapidor	BnaC03g01260.1D2	BnaA03g01080.1D2
Tapidor	BnaC03g01360.1D2	BnaA03g01220.1D2
Tapidor	BnaC03g01410.1D2	BnaA03g01290.1D2
Tapidor	BnaC03g01420.1D2	BnaA03g01300.1D2
Tapidor	BnaC03g01430.1D2	BnaA03g01310.1D2
Tapidor	BnaC03g01450.1D2	BnaA03g01330.1D2
Tapidor	BnaC03g01460.1D2	BnaA03g01340.1D2
Tapidor	BnaC03g01470.1D2	BnaA03g01350.1D2
Tapidor	BnaC03g01480.1D2	BnaA03g01360.1D2
Tapidor	BnaC03g01490.1D2	BnaA03g01370.1D2
Tapidor	BnaC03g01540.1D2	BnaA03g01420.1D2
Tapidor	BnaC03g01670.1D2	BnaA03g07630.1D2
Tapidor	BnaC03g01820.1D2	BnaA03g02290.1D2
Tapidor	BnaC03g02190.1D2	BnaA03g01790.1D2
Tapidor	BnaC03g02240.1D2	BnaA03g01840.1D2
Tapidor	BnaC03g02300.1D2	BnaA03g01890.1D2
Tapidor	BnaC03g02300.1D2	BnaA03g01910.1D2
Tapidor	BnaC03g02330.1D2	BnaA03g01930.1D2
Tapidor	BnaC03g02380.1D2	BnaA03g07570.1D2
Tapidor	BnaC03g02400.1D2	BnaA03g07560.1D2
Tapidor	BnaC03g02430.1D2	BnaA03g07480.1D2
Tapidor	BnaC03g02600.1D2	BnaA03g02600.1D2
Tapidor	BnaC03g02610.1D2	BnaA03g02600.1D2

Tapidor	BnaC03g02680.1D2	BnaA03g02660.1D2
Tapidor	BnaC03g02890.1D2	BnaA03g02890.1D2
Tapidor	BnaC03g02920.1D2	BnaA03g02940.1D2
Tapidor	BnaC03g03110.1D2	BnaA03g07320.1D2
Tapidor	BnaC03g03320.1D2	BnaA03g03270.1D2
Tapidor	BnaC03g03590.1D2	BnaA03g03370.1D2
Tapidor	BnaC03g03750.1D2	BnaA03g03490.1D2
Tapidor	BnaC03g03750.1D2	BnaA03g03790.1D2
Tapidor	BnaC03g04170.1D2	BnaA03g03490.1D2
Tapidor	BnaC03g04170.1D2	BnaA03g03790.1D2
Tapidor	BnaC03g04210.1D2	BnaA03g03830.1D2
Tapidor	BnaC03g04460.1D2	BnaA03g04020.1D2
Tapidor	BnaC03g04530.1D2	BnaA03g04080.1D2
Tapidor	BnaC03g04580.1D2	BnaA03g04120.1D2
Tapidor	BnaC03g04610.1D2	BnaA03g04140.1D2
Tapidor	BnaC03g04710.1D2	BnaA03g04250.1D2
Tapidor	BnaC03g04730.1D2	BnaA03g04270.1D2
Tapidor	BnaC03g04760.1D2	BnaA03g04300.1D2
Tapidor	BnaC03g04780.1D2	BnaA03g04320.1D2
Tapidor	BnaC03g04990.1D2	BnaA03g04450.1D2
Tapidor	BnaC03g05100.1D2	BnaA03g04580.1D2
Tapidor	BnaC03g05180.1D2	BnaA03g04650.1D2

List of HE genes (C04 to A04)

**Accession**	**Lost gene**	**Duplicated HE gene**
GSchnittkohl	BnaC04g00950.1D2	BnaA04g26610.1D2
GSchnittkohl	BnaC04g42090.1D2	BnaA04g23660.1D2
GSchnittkohl	BnaC04g42140.1D2	BnaA04g23690.1D2
GSchnittkohl	BnaC04g42230.1D2	BnaA04g22660.1D2
GSchnittkohl	BnaC04g42290.1D2	BnaA04g22730.1D2
GSchnittkohl	BnaC04g42370.1D2	BnaA04g22930.1D2
GSchnittkohl	BnaC04g42400.1D2	BnaA04g22960.1D2
GSchnittkohl	BnaC04g42410.1D2	BnaA04g22970.1D2
GSchnittkohl	BnaC04g42430.1D2	BnaA04g22990.1D2
GSchnittkohl	BnaC04g42440.1D2	BnaA04g23000.1D2
GSchnittkohl	BnaC04g42920.1D2	BnaA04g23630.1D2
GSchnittkohl	BnaC04g42950.1D2	BnaA04g23040.1D2
GSchnittkohl	BnaC04g42960.1D2	BnaA04g23050.1D2
GSchnittkohl	BnaC04g42990.1D2	BnaA04g23090.1D2
GSchnittkohl	BnaC04g43010.1D2	BnaA04g23110.1D2
GSchnittkohl	BnaC04g43050.1D2	BnaA04g23140.1D2
GSchnittkohl	BnaC04g43070.1D2	BnaA04g23160.1D2
GSchnittkohl	BnaC04g43110.1D2	BnaA04g23190.1D2
GSchnittkohl	BnaC04g43270.1D2	BnaA04g23320.1D2
GSchnittkohl	BnaC04g43290.1D2	BnaA04g23340.1D2
GSchnittkohl	BnaC04g43340.1D2	BnaA04g23390.1D2
GSchnittkohl	BnaC04g43350.1D2	BnaA04g23400.1D2
GSchnittkohl	BnaC04g43470.1D2	BnaA04g23490.1D2
GSchnittkohl	BnaC04g43500.1D2	BnaA04g23520.1D2
GSchnittkohl	BnaC04g43650.1D2	BnaA04g23810.1D2
GSchnittkohl	BnaC04g43660.1D2	BnaA04g23820.1D2

GSchnittkohl	BnaC04g43670.1D2	BnaA04g23830.1D2
GSchnittkohl	BnaC04g43680.1D2	BnaA04g23840.1D2
GSchnittkohl	BnaC04g43690.1D2	BnaA04g23850.1D2
GSchnittkohl	BnaC04g43700.1D2	BnaA04g23860.1D2
GSchnittkohl	BnaC04g43740.1D2	BnaA04g23900.1D2
GSchnittkohl	BnaC04g43970.1D2	BnaA04g24260.1D2
GSchnittkohl	BnaC04g43990.1D2	BnaA04g24280.1D2
GSchnittkohl	BnaC04g44080.1D2	BnaA04g24420.1D2
GSchnittkohl	BnaC04g44090.1D2	BnaA04g24430.1D2
GSchnittkohl	BnaC04g44130.1D2	BnaA04g24460.1D2
GSchnittkohl	BnaC04g44140.1D2	BnaA04g24470.1D2
GSchnittkohl	BnaC04g44170.1D2	BnaA04g24490.1D2
GSchnittkohl	BnaC04g44170.1D2	BnaA04g24500.1D2
GSchnittkohl	BnaC04g44210.1D2	BnaA04g24520.1D2
GSchnittkohl	BnaC04g44220.1D2	BnaA04g24530.1D2
GSchnittkohl	BnaC04g44230.1D2	BnaA04g24540.1D2
GSchnittkohl	BnaC04g44280.1D2	BnaA04g24610.1D2
GSchnittkohl	BnaC04g44340.1D2	BnaA04g24650.1D2
GSchnittkohl	BnaC04g44350.1D2	BnaA04g24660.1D2
GSchnittkohl	BnaC04g44370.1D2	BnaA04g24680.1D2
GSchnittkohl	BnaC04g44420.1D2	BnaA04g24750.1D2
GSchnittkohl	BnaC04g44460.1D2	BnaA04g24770.1D2
GSchnittkohl	BnaC04g44480.1D2	BnaA04g25310.1D2
GSchnittkohl	BnaC04g44500.1D2	BnaA04g24810.1D2
GSchnittkohl	BnaC04g44520.1D2	BnaA04g24830.1D2
GSchnittkohl	BnaC04g44540.1D2	BnaA04g24850.1D2
GSchnittkohl	BnaC04g44550.1D2	BnaA04g24860.1D2
GSchnittkohl	BnaC04g44560.1D2	BnaA04g24870.1D2
GSchnittkohl	BnaC04g44570.1D2	BnaA04g24880.1D2
GSchnittkohl	BnaC04g44580.1D2	BnaA04g24890.1D2
GSchnittkohl	BnaC04g44620.1D2	BnaA04g24930.1D2
GSchnittkohl	BnaC04g44640.1D2	BnaA04g24950.1D2
GSchnittkohl	BnaC04g44670.1D2	BnaA04g24990.1D2
GSchnittkohl	BnaC04g44680.1D2	BnaA04g25000.1D2
GSchnittkohl	BnaC04g44690.1D2	BnaA04g25010.1D2
GSchnittkohl	BnaC04g44710.1D2	BnaA04g25030.1D2
GSchnittkohl	BnaC04g44720.1D2	BnaA04g25040.1D2
GSchnittkohl	BnaC04g44730.1D2	BnaA04g25050.1D2
GSchnittkohl	BnaC04g44810.1D2	BnaA04g25130.1D2
GSchnittkohl	BnaC04g44860.1D2	BnaA04g25150.1D2
GSchnittkohl	BnaC04g44870.1D2	BnaA04g25160.1D2
GSchnittkohl	BnaC04g44910.1D2	BnaA04g26070.1D2
GSchnittkohl	BnaC04g44970.1D2	BnaA04g25220.1D2
GSchnittkohl	BnaC04g45010.1D2	BnaA04g25290.1D2
GSchnittkohl	BnaC04g45120.1D2	BnaA04g25360.1D2
GSchnittkohl	BnaC04g45210.1D2	BnaA04g25480.1D2
GSchnittkohl	BnaC04g45230.1D2	BnaA04g25490.1D2
GSchnittkohl	BnaC04g45240.1D2	BnaA04g25500.1D2
GSchnittkohl	BnaC04g45260.1D2	BnaA04g25530.1D2
GSchnittkohl	BnaC04g45310.1D2	BnaA04g25650.1D2
GSchnittkohl	BnaC04g45320.1D2	BnaA04g25660.1D2

GSchnittkohl	BnaC04g45350.1D2	BnaA04g25680.1D2
GSchnittkohl	BnaC04g45380.1D2	BnaA04g25690.1D2
GSchnittkohl	BnaC04g45480.1D2	BnaA04g25790.1D2
GSchnittkohl	BnaC04g45490.1D2	BnaA04g25800.1D2
GSchnittkohl	BnaC04g45500.1D2	BnaA04g25810.1D2
GSchnittkohl	BnaC04g45540.1D2	BnaA04g25860.1D2
GSchnittkohl	BnaC04g45550.1D2	BnaA04g25870.1D2
GSchnittkohl	BnaC04g45560.1D2	BnaA04g25880.1D2
GSchnittkohl	BnaC04g45600.1D2	BnaA04g25920.1D2
GSchnittkohl	BnaC04g45610.1D2	BnaA04g25930.1D2
GSchnittkohl	BnaC04g45620.1D2	BnaA04g25940.1D2
GSchnittkohl	BnaC04g45630.1D2	BnaA04g25950.1D2
GSchnittkohl	BnaC04g45650.1D2	BnaA04g25960.1D2
GSchnittkohl	BnaC04g45660.1D2	BnaA04g25970.1D2
GSchnittkohl	BnaC04g45680.1D2	BnaA04g25990.1D2
GSchnittkohl	BnaC04g45720.1D2	BnaA04g26020.1D2
GSchnittkohl	BnaC04g45750.1D2	BnaA04g26040.1D2
GSchnittkohl	BnaC04g45770.1D2	BnaA04g26070.1D2
GSchnittkohl	BnaC04g45780.1D2	BnaA04g26080.1D2
GSchnittkohl	BnaC04g45800.1D2	BnaA04g26100.1D2
GSchnittkohl	BnaC04g45860.1D2	BnaA04g26690.1D2
GSchnittkohl	BnaC04g45870.1D2	BnaA04g26680.1D2
GSchnittkohl	BnaC04g45890.1D2	BnaA04g25540.1D2
GSchnittkohl	BnaC04g45910.1D2	BnaA04g25560.1D2
GSchnittkohl	BnaC04g45990.1D2	BnaA04g24110.1D2
GSchnittkohl	BnaC04g46010.1D2	BnaA04g24130.1D2
GSchnittkohl	BnaC04g46040.1D2	BnaA04g24180.1D2
GSchnittkohl	BnaC04g46060.1D2	BnaA04g24200.1D2
GSchnittkohl	BnaC04g46110.1D2	BnaA04g26580.1D2
GSchnittkohl	BnaC04g46120.1D2	BnaA04g26580.1D2
GSchnittkohl	BnaC04g46130.1D2	BnaA04g26570.1D2
GSchnittkohl	BnaC04g46220.1D2	BnaA04g26510.1D2
GSchnittkohl	BnaC04g46320.1D2	BnaA04g26460.1D2
GSchnittkohl	BnaC04g46350.1D2	BnaA04g26440.1D2
GSchnittkohl	BnaC04g46420.1D2	BnaA04g26360.1D2
GSchnittkohl	BnaC04g46450.1D2	BnaA04g26340.1D2
GSchnittkohl	BnaC04g46490.1D2	BnaA04g26310.1D2
GSchnittkohl	BnaC04g46500.1D2	BnaA04g26300.1D2
GSchnittkohl	BnaC04g46510.1D2	BnaA04g26290.1D2
GSchnittkohl	BnaC04g46520.1D2	BnaA04g26110.1D2
GSchnittkohl	BnaC04g46550.1D2	BnaA04g26270.1D2
GSchnittkohl	BnaC04g46560.1D2	BnaA04g26260.1D2
GSchnittkohl	BnaC04g46570.1D2	BnaA04g26250.1D2
GSchnittkohl	BnaC04g46580.1D2	BnaA04g26240.1D2
CRY_1	BnaC04g11680.1D2	BnaA04g23580.1D2
CRY_1	BnaC04g11690.1D2	BnaA04g23580.1D2
CRY_1	BnaC04g41680.1D2	BnaA04g22370.1D2
CRY_1	BnaC04g42140.1D2	BnaA04g23690.1D2
CRY_1	BnaC04g42170.1D2	BnaA04g22590.1D2
CRY_1	BnaC04g42200.1D2	BnaA04g22630.1D2
CRY_1	BnaC04g42230.1D2	BnaA04g22660.1D2

CRY_1	BnaC04g42530.1D2	BnaA04g22100.1D2
CRY_1	BnaC04g42920.1D2	BnaA04g23630.1D2
CRY_1	BnaC04g42990.1D2	BnaA04g23090.1D2
CRY_1	BnaC04g43070.1D2	BnaA04g23160.1D2
CRY_1	BnaC04g43280.1D2	BnaA04g23330.1D2
CRY_1	BnaC04g43290.1D2	BnaA04g23340.1D2
CRY_1	BnaC04g43340.1D2	BnaA04g23390.1D2
CRY_1	BnaC04g43350.1D2	BnaA04g23400.1D2
CRY_1	BnaC04g43540.1D2	BnaA04g23560.1D2
CRY_1	BnaC04g43550.1D2	BnaA04g23570.1D2
CRY_1	BnaC04g43570.1D2	BnaA04g23590.1D2
CRY_1	BnaC04g43650.1D2	BnaA04g23810.1D2
CRY_1	BnaC04g43660.1D2	BnaA04g23820.1D2
CRY_1	BnaC04g43670.1D2	BnaA04g23830.1D2
CRY_1	BnaC04g43680.1D2	BnaA04g23840.1D2
CRY_1	BnaC04g43690.1D2	BnaA04g23850.1D2
CRY_1	BnaC04g43700.1D2	BnaA04g23860.1D2
CRY_1	BnaC04g43800.1D2	BnaA04g23930.1D2
CRY_1	BnaC04g43880.1D2	BnaA04g24020.1D2
CRY_1	BnaC04g44080.1D2	BnaA04g24420.1D2
CRY_1	BnaC04g44090.1D2	BnaA04g24430.1D2
CRY_1	BnaC04g44170.1D2	BnaA04g24490.1D2
CRY_1	BnaC04g44210.1D2	BnaA04g24520.1D2
CRY_1	BnaC04g44220.1D2	BnaA04g24530.1D2
CRY_1	BnaC04g44230.1D2	BnaA04g24540.1D2
CRY_1	BnaC04g44340.1D2	BnaA04g24650.1D2
CRY_1	BnaC04g44350.1D2	BnaA04g24660.1D2
CRY_1	BnaC04g44400.1D2	BnaA04g24720.1D2
CRY_1	BnaC04g44460.1D2	BnaA04g24770.1D2
CRY_1	BnaC04g44580.1D2	BnaA04g24890.1D2
CRY_1	BnaC04g44730.1D2	BnaA04g25050.1D2
CRY_1	BnaC04g44810.1D2	BnaA04g25130.1D2
CRY_1	BnaC04g44910.1D2	BnaA04g26070.1D2
CRY_1	BnaC04g44950.1D2	BnaA04g25200.1D2
CRY_1	BnaC04g45010.1D2	BnaA04g25290.1D2
CRY_1	BnaC04g45120.1D2	BnaA04g25360.1D2
CRY_1	BnaC04g45150.1D2	BnaA04g25410.1D2
CRY_1	BnaC04g45210.1D2	BnaA04g25480.1D2
CRY_1	BnaC04g45240.1D2	BnaA04g25500.1D2
CRY_1	BnaC04g45410.1D2	BnaA04g25720.1D2
CRY_1	BnaC04g45480.1D2	BnaA04g25790.1D2
CRY_1	BnaC04g45720.1D2	BnaA04g26020.1D2
CRY_1	BnaC04g45800.1D2	BnaA04g26100.1D2
CRY_1	BnaC04g45860.1D2	BnaA04g26690.1D2
CRY_1	BnaC04g45950.1D2	BnaA04g25060.1D2
CRY_1	BnaC04g45990.1D2	BnaA04g24110.1D2
CRY_1	BnaC04g46010.1D2	BnaA04g24130.1D2
CRY_1	BnaC04g46040.1D2	BnaA04g24180.1D2
CRY_1	BnaC04g46060.1D2	BnaA04g24200.1D2
CRY_1	BnaC04g46110.1D2	BnaA04g26580.1D2
CRY_1	BnaC04g46130.1D2	BnaA04g26570.1D2

CRY_1	BnaC04g46220.1D2	BnaA04g26510.1D2
CRY_1	BnaC04g46350.1D2	BnaA04g26440.1D2
CRY_1	BnaC04g46420.1D2	BnaA04g26360.1D2
CRY_1	BnaC04g46510.1D2	BnaA04g26290.1D2
CRY_1	BnaC04g46520.1D2	BnaA04g26110.1D2
CRY_1	BnaC04g46550.1D2	BnaA04g26270.1D2

List of HE genes (C04 to A05)

**Accession**	**Lost gene**	**Duplicated HE gene**
GSchnittkohl	BnaC04g00020.1D2	BnaA05g00160.1D2
GSchnittkohl	BnaC04g00030.1D2	BnaA05g00160.1D2
GSchnittkohl	BnaC04g00100.1D2	BnaA05g00230.1D2
GSchnittkohl	BnaC04g00140.1D2	BnaA05g00270.1D2
GSchnittkohl	BnaC04g00180.1D2	BnaA05g00320.1D2
GSchnittkohl	BnaC04g00820.1D2	BnaA05g00460.1D2
GSchnittkohl	BnaC04g00830.1D2	BnaA05g00480.1D2
GSchnittkohl	BnaC04g00900.1D2	BnaA05g00400.1D2
GSchnittkohl	BnaC04g00910.1D2	BnaA05g00390.1D2
GSchnittkohl	BnaC04g18680.1D2	BnaA05g31470.1D2
GSchnittkohl	BnaC04g35580.1D2	BnaA05g35050.1D2
GSchnittkohl	BnaC04g44470.1D2	BnaA05g03360.1D2
GSchnittkohl	BnaC04g46040.1D2	BnaA05g00180.1D2
GSchnittkohl	BnaC04g46110.1D2	BnaA05g00400.1D2
CRY_1	BnaC04g13740.1D2	BnaA05g11840.1D2
CRY_1	BnaC04g35500.1D2	BnaA05g34480.1D2
CRY_1	BnaC04g44910.1D2	BnaA05g05330.1D2
CRY_1	BnaC04g46040.1D2	BnaA05g00180.1D2
CRY_1	BnaC04g46520.1D2	BnaA05g01060.1D2

List of HE genes (C05 to A05)

**Accession**	**Lost gene**	**Duplicated HE gene**
GSchnittkohl	BnaC05g36320.1D2	BnaA05g27610.1D2
GSchnittkohl	BnaC05g36360.1D2	BnaA05g27660.1D2
GSchnittkohl	BnaC05g36380.1D2	BnaA05g27680.1D2
GSchnittkohl	BnaC05g36400.1D2	BnaA05g27700.1D2
GSchnittkohl	BnaC05g36480.1D2	BnaA05g27740.1D2
GSchnittkohl	BnaC05g36520.1D2	BnaA05g27800.1D2
GSchnittkohl	BnaC05g36530.1D2	BnaA05g27830.1D2
GSchnittkohl	BnaC05g36560.1D2	BnaA05g27870.1D2
GSchnittkohl	BnaC05g37490.1D2	BnaA05g28770.1D2
GSchnittkohl	BnaC05g37540.1D2	BnaA05g28820.1D2
GSchnittkohl	BnaC05g37550.1D2	BnaA05g28830.1D2
GSchnittkohl	BnaC05g37570.1D2	BnaA05g28850.1D2
GSchnittkohl	BnaC05g37640.1D2	BnaA05g28900.1D2
GSchnittkohl	BnaC05g37840.1D2	BnaA05g29010.1D2
GSchnittkohl	BnaC05g37880.1D2	BnaA05g29040.1D2
GSchnittkohl	BnaC05g37910.1D2	BnaA05g29070.1D2
GSchnittkohl	BnaC05g37920.1D2	BnaA05g29080.1D2
GSchnittkohl	BnaC05g37930.1D2	BnaA05g29090.1D2
GSchnittkohl	BnaC05g37940.1D2	BnaA05g29100.1D2
GSchnittkohl	BnaC05g37950.1D2	BnaA05g29110.1D2

GSchnittkohl	BnaC05g37960.1D2	BnaA05g29300.1D2
GSchnittkohl	BnaC05g38000.1D2	BnaA05g29260.1D2
GSchnittkohl	BnaC05g38010.1D2	BnaA05g29250.1D2
GSchnittkohl	BnaC05g38040.1D2	BnaA05g29220.1D2
GSchnittkohl	BnaC05g38050.1D2	BnaA05g29210.1D2
GSchnittkohl	BnaC05g38070.1D2	BnaA05g29190.1D2
GSchnittkohl	BnaC05g38200.1D2	BnaA05g29340.1D2
GSchnittkohl	BnaC05g38210.1D2	BnaA05g29350.1D2
GSchnittkohl	BnaC05g38220.1D2	BnaA05g29360.1D2
GSchnittkohl	BnaC05g38250.1D2	BnaA05g29400.1D2
GSchnittkohl	BnaC05g38280.1D2	BnaA05g29430.1D2
GSchnittkohl	BnaC05g38300.1D2	BnaA05g29440.1D2
GSchnittkohl	BnaC05g38390.1D2	BnaA05g29510.1D2
GSchnittkohl	BnaC05g38500.1D2	BnaA05g29630.1D2
GSchnittkohl	BnaC05g38530.1D2	BnaA05g29650.1D2
GSchnittkohl	BnaC05g38680.1D2	BnaA05g29780.1D2
GSchnittkohl	BnaC05g38730.1D2	BnaA05g29820.1D2
GSchnittkohl	BnaC05g38730.1D2	BnaA05g29830.1D2
GSchnittkohl	BnaC05g38750.1D2	BnaA05g30830.1D2
GSchnittkohl	BnaC05g38760.1D2	BnaA05g30840.1D2
GSchnittkohl	BnaC05g38770.1D2	BnaA05g30850.1D2
GSchnittkohl	BnaC05g38790.1D2	BnaA05g30870.1D2
GSchnittkohl	BnaC05g38820.1D2	BnaA05g30080.1D2
GSchnittkohl	BnaC05g38840.1D2	BnaA05g30100.1D2
GSchnittkohl	BnaC05g38850.1D2	BnaA05g30110.1D2
GSchnittkohl	BnaC05g38880.1D2	BnaA05g30290.1D2
GSchnittkohl	BnaC05g38900.1D2	BnaA05g30130.1D2
GSchnittkohl	BnaC05g38930.1D2	BnaA05g30160.1D2
GSchnittkohl	BnaC05g38940.1D2	BnaA05g30170.1D2
GSchnittkohl	BnaC05g38950.1D2	BnaA05g30180.1D2
GSchnittkohl	BnaC05g38980.1D2	BnaA05g30200.1D2
GSchnittkohl	BnaC05g38990.1D2	BnaA05g30210.1D2
GSchnittkohl	BnaC05g39000.1D2	BnaA05g30220.1D2
GSchnittkohl	BnaC05g39090.1D2	BnaA05g30300.1D2
GSchnittkohl	BnaC05g39110.1D2	BnaA05g30320.1D2
GSchnittkohl	BnaC05g39140.1D2	BnaA05g30350.1D2
GSchnittkohl	BnaC05g39160.1D2	BnaA05g30370.1D2
GSchnittkohl	BnaC05g39170.1D2	BnaA05g30380.1D2
GSchnittkohl	BnaC05g39250.1D2	BnaA05g30500.1D2
GSchnittkohl	BnaC05g39270.1D2	BnaA05g30520.1D2
GSchnittkohl	BnaC05g39410.1D2	BnaA05g30640.1D2
GSchnittkohl	BnaC05g39470.1D2	BnaA05g30700.1D2
GSchnittkohl	BnaC05g39640.1D2	BnaA05g30940.1D2
GSchnittkohl	BnaC05g39710.1D2	BnaA05g30410.1D2
GSchnittkohl	BnaC05g39720.1D2	BnaA05g30430.1D2
GSchnittkohl	BnaC05g39730.1D2	BnaA05g30070.1D2
GSchnittkohl	BnaC05g39770.1D2	BnaA05g30030.1D2
GSchnittkohl	BnaC05g39780.1D2	BnaA05g30020.1D2
GSchnittkohl	BnaC05g39790.1D2	BnaA05g30010.1D2
GSchnittkohl	BnaC05g39810.1D2	BnaA05g29950.1D2
GSchnittkohl	BnaC05g39820.1D2	BnaA05g29930.1D2

GSchnittkohl	BnaC05g39840.1D2	BnaA05g29910.1D2
GSchnittkohl	BnaC05g39860.1D2	BnaA05g29880.1D2
GSchnittkohl	BnaC05g39870.1D2	BnaA05g29870.1D2
GSchnittkohl	BnaC05g39880.1D2	BnaA05g29860.1D2
GSchnittkohl	BnaC05g40140.1D2	BnaA05g31130.1D2
GSchnittkohl	BnaC05g40260.1D2	BnaA05g31240.1D2
GSchnittkohl	BnaC05g40320.1D2	BnaA05g31300.1D2
GSchnittkohl	BnaC05g40330.1D2	BnaA05g31310.1D2
GSchnittkohl	BnaC05g40380.1D2	BnaA05g31370.1D2
GSchnittkohl	BnaC05g40410.1D2	BnaA05g31410.1D2
GSchnittkohl	BnaC05g40430.1D2	BnaA05g31420.1D2
GSchnittkohl	BnaC05g40460.1D2	BnaA05g31450.1D2
GSchnittkohl	BnaC05g40470.1D2	BnaA05g31460.1D2
GSchnittkohl	BnaC05g40510.1D2	BnaA05g31510.1D2
GSchnittkohl	BnaC05g40520.1D2	BnaA05g31520.1D2
GSchnittkohl	BnaC05g40530.1D2	BnaA05g31530.1D2
GSchnittkohl	BnaC05g40570.1D2	BnaA05g31560.1D2
GSchnittkohl	BnaC05g40580.1D2	BnaA05g31580.1D2
GSchnittkohl	BnaC05g40590.1D2	BnaA05g31590.1D2
GSchnittkohl	BnaC05g40730.1D2	BnaA05g31710.1D2
GSchnittkohl	BnaC05g40740.1D2	BnaA05g31730.1D2
GSchnittkohl	BnaC05g40810.1D2	BnaA05g31780.1D2
GSchnittkohl	BnaC05g40820.1D2	BnaA05g31790.1D2
GSchnittkohl	BnaC05g40830.1D2	BnaA05g31800.1D2
GSchnittkohl	BnaC05g40840.1D2	BnaA05g31810.1D2
GSchnittkohl	BnaC05g40850.1D2	BnaA05g31820.1D2
GSchnittkohl	BnaC05g40870.1D2	BnaA05g31830.1D2
GSchnittkohl	BnaC05g40880.1D2	BnaA05g31840.1D2
GSchnittkohl	BnaC05g40890.1D2	BnaA05g31880.1D2
GSchnittkohl	BnaC05g40900.1D2	BnaA05g31890.1D2
GSchnittkohl	BnaC05g40930.1D2	BnaA05g31920.1D2
GSchnittkohl	BnaC05g40940.1D2	BnaA05g31930.1D2
GSchnittkohl	BnaC05g40950.1D2	BnaA05g31940.1D2
GSchnittkohl	BnaC05g40960.1D2	BnaA05g31950.1D2
GSchnittkohl	BnaC05g40970.1D2	BnaA05g31960.1D2
GSchnittkohl	BnaC05g41040.1D2	BnaA05g32050.1D2
GSchnittkohl	BnaC05g41050.1D2	BnaA05g32080.1D2
GSchnittkohl	BnaC05g41060.1D2	BnaA05g32090.1D2
GSchnittkohl	BnaC05g41070.1D2	BnaA05g32100.1D2
GSchnittkohl	BnaC05g41110.1D2	BnaA05g32140.1D2
GSchnittkohl	BnaC05g41130.1D2	BnaA05g32150.1D2
GSchnittkohl	BnaC05g41150.1D2	BnaA05g32160.1D2
GSchnittkohl	BnaC05g41160.1D2	BnaA05g32170.1D2
GSchnittkohl	BnaC05g41170.1D2	BnaA05g32180.1D2
GSchnittkohl	BnaC05g41220.1D2	BnaA05g32240.1D2
GSchnittkohl	BnaC05g41250.1D2	BnaA05g32260.1D2
GSchnittkohl	BnaC05g41280.1D2	BnaA05g32290.1D2
GSchnittkohl	BnaC05g41300.1D2	BnaA05g32300.1D2
GSchnittkohl	BnaC05g41330.1D2	BnaA05g32330.1D2
GSchnittkohl	BnaC05g41350.1D2	BnaA05g32350.1D2
GSchnittkohl	BnaC05g41370.1D2	BnaA05g32370.1D2

GSchnittkohl	BnaC05g41380.1D2	BnaA05g32380.1D2
GSchnittkohl	BnaC05g41410.1D2	BnaA05g32410.1D2
GSchnittkohl	BnaC05g41430.1D2	BnaA05g32420.1D2
GSchnittkohl	BnaC05g41470.1D2	BnaA05g32460.1D2
GSchnittkohl	BnaC05g41490.1D2	BnaA05g32480.1D2
GSchnittkohl	BnaC05g41500.1D2	BnaA05g32490.1D2
GSchnittkohl	BnaC05g41540.1D2	BnaA05g32530.1D2
GSchnittkohl	BnaC05g41550.1D2	BnaA05g32540.1D2
GSchnittkohl	BnaC05g41570.1D2	BnaA05g32560.1D2
GSchnittkohl	BnaC05g41580.1D2	BnaA05g32570.1D2
GSchnittkohl	BnaC05g41640.1D2	BnaA05g32620.1D2
GSchnittkohl	BnaC05g41660.1D2	BnaA05g32640.1D2
GSchnittkohl	BnaC05g41680.1D2	BnaA05g32660.1D2
GSchnittkohl	BnaC05g41690.1D2	BnaA05g32670.1D2
GSchnittkohl	BnaC05g41740.1D2	BnaA05g32710.1D2
GSchnittkohl	BnaC05g41760.1D2	BnaA05g32730.1D2
GSchnittkohl	BnaC05g41780.1D2	BnaA05g32740.1D2
GSchnittkohl	BnaC05g41890.1D2	BnaA05g33530.1D2
GSchnittkohl	BnaC05g41920.1D2	BnaA05g33500.1D2
GSchnittkohl	BnaC05g41940.1D2	BnaA05g33490.1D2
GSchnittkohl	BnaC05g42110.1D2	BnaA05g33380.1D2
GSchnittkohl	BnaC05g42120.1D2	BnaA05g33370.1D2
GSchnittkohl	BnaC05g42130.1D2	BnaA05g33720.1D2
GSchnittkohl	BnaC05g42190.1D2	BnaA05g33780.1D2
GSchnittkohl	BnaC05g42200.1D2	BnaA05g33790.1D2
GSchnittkohl	BnaC05g42260.1D2	BnaA05g33860.1D2
GSchnittkohl	BnaC05g42280.1D2	BnaA05g33880.1D2
GSchnittkohl	BnaC05g42340.1D2	BnaA05g33970.1D2
GSchnittkohl	BnaC05g42360.1D2	BnaA05g33360.1D2
GSchnittkohl	BnaC05g42380.1D2	BnaA05g33350.1D2
GSchnittkohl	BnaC05g42390.1D2	BnaA05g33330.1D2
GSchnittkohl	BnaC05g42420.1D2	BnaA05g33310.1D2
GSchnittkohl	BnaC05g42850.1D2	BnaA05g34190.1D2
GSchnittkohl	BnaC05g42860.1D2	BnaA05g34180.1D2
GSchnittkohl	BnaC05g42880.1D2	BnaA05g34170.1D2
GSchnittkohl	BnaC05g42930.1D2	BnaA05g34120.1D2
GSchnittkohl	BnaC05g42990.1D2	BnaA05g34060.1D2
GSchnittkohl	BnaC05g43000.1D2	BnaA05g34050.1D2
GSchnittkohl	BnaC05g43020.1D2	BnaA05g34030.1D2
GSchnittkohl	BnaC05g43040.1D2	BnaA05g34020.1D2
GSchnittkohl	BnaC05g43150.1D2	BnaA05g32870.1D2
GSchnittkohl	BnaC05g43160.1D2	BnaA05g32870.1D2
GSchnittkohl	BnaC05g43200.1D2	BnaA05g32910.1D2
GSchnittkohl	BnaC05g43230.1D2	BnaA05g32930.1D2
GSchnittkohl	BnaC05g43250.1D2	BnaA05g32950.1D2
GSchnittkohl	BnaC05g43270.1D2	BnaA05g32970.1D2
GSchnittkohl	BnaC05g43290.1D2	BnaA05g33220.1D2
GSchnittkohl	BnaC05g43310.1D2	BnaA05g33200.1D2
GSchnittkohl	BnaC05g43430.1D2	BnaA05g33130.1D2
GSchnittkohl	BnaC05g43500.1D2	BnaA05g33070.1D2
GSchnittkohl	BnaC05g43510.1D2	BnaA05g33060.1D2

GSchnittkohl	BnaC05g43520.1D2	BnaA05g33050.1D2
GSchnittkohl	BnaC05g43530.1D2	BnaA05g33050.1D2
GSchnittkohl	BnaC05g43570.1D2	BnaA05g33020.1D2
GSchnittkohl	BnaC05g43760.1D2	BnaA05g34300.1D2
GSchnittkohl	BnaC05g43770.1D2	BnaA05g34310.1D2
GSchnittkohl	BnaC05g43780.1D2	BnaA05g34320.1D2
GSchnittkohl	BnaC05g43790.1D2	BnaA05g34330.1D2
GSchnittkohl	BnaC05g43830.1D2	BnaA05g34360.1D2
GSchnittkohl	BnaC05g43850.1D2	BnaA05g34390.1D2
GSchnittkohl	BnaC05g43870.1D2	BnaA05g34410.1D2
GSchnittkohl	BnaC05g43920.1D2	BnaA05g34440.1D2
GSchnittkohl	BnaC05g43950.1D2	BnaA05g34230.1D2
GSchnittkohl	BnaC05g43960.1D2	BnaA05g34240.1D2
GSchnittkohl	BnaC05g43970.1D2	BnaA05g34240.1D2
GSchnittkohl	BnaC05g44090.1D2	BnaA05g34960.1D2
GSchnittkohl	BnaC05g44110.1D2	BnaA05g34940.1D2
GSchnittkohl	BnaC05g44160.1D2	BnaA05g34880.1D2
GSchnittkohl	BnaC05g44170.1D2	BnaA05g34870.1D2
GSchnittkohl	BnaC05g44210.1D2	BnaA05g34830.1D2
GSchnittkohl	BnaC05g44230.1D2	BnaA05g35030.1D2
GSchnittkohl	BnaC05g44250.1D2	BnaA05g35010.1D2
GSchnittkohl	BnaC05g44260.1D2	BnaA05g34800.1D2
GSchnittkohl	BnaC05g44280.1D2	BnaA05g34780.1D2
GSchnittkohl	BnaC05g44290.1D2	BnaA05g34770.1D2
GSchnittkohl	BnaC05g44300.1D2	BnaA05g34760.1D2
GSchnittkohl	BnaC05g44320.1D2	BnaA05g34740.1D2
GSchnittkohl	BnaC05g44360.1D2	BnaA05g34700.1D2
GSchnittkohl	BnaC05g44410.1D2	BnaA05g34640.1D2
GSchnittkohl	BnaC05g44460.1D2	BnaA05g34590.1D2
GSchnittkohl	BnaC05g44470.1D2	BnaA05g34580.1D2
GSchnittkohl	BnaC05g44490.1D2	BnaA05g34570.1D2
GSchnittkohl	BnaC05g44500.1D2	BnaA05g34560.1D2
R53	BnaC05g35880.1D2	BnaA05g27150.1D2
R53	BnaC05g35890.1D2	BnaA05g27140.1D2
R53	BnaC05g35910.1D2	BnaA05g27120.1D2
R53	BnaC05g35960.1D2	BnaA05g27160.1D2
R53	BnaC05g35970.1D2	BnaA05g27170.1D2
R53	BnaC05g36010.1D2	BnaA05g27220.1D2
R53	BnaC05g36060.1D2	BnaA05g27310.1D2
R53	BnaC05g36100.1D2	BnaA05g27390.1D2
R53	BnaC05g36110.1D2	BnaA05g27410.1D2
R53	BnaC05g36130.1D2	BnaA05g27440.1D2
R53	BnaC05g36150.1D2	BnaA05g27470.1D2
R53	BnaC05g36170.1D2	BnaA05g27490.1D2
R53	BnaC05g36200.1D2	BnaA05g27520.1D2
R53	BnaC05g36320.1D2	BnaA05g27610.1D2
R53	BnaC05g36330.1D2	BnaA05g27620.1D2
R53	BnaC05g36380.1D2	BnaA05g27680.1D2
R53	BnaC05g36400.1D2	BnaA05g27700.1D2
R53	BnaC05g36480.1D2	BnaA05g27740.1D2
R53	BnaC05g36520.1D2	BnaA05g27800.1D2

R53	BnaC05g36530.1D2	BnaA05g27830.1D2
R53	BnaC05g36560.1D2	BnaA05g27870.1D2
R53	BnaC05g36590.1D2	BnaA05g27900.1D2
R53	BnaC05g36600.1D2	BnaA05g27910.1D2
R53	BnaC05g36620.1D2	BnaA05g27930.1D2
R53	BnaC05g36630.1D2	BnaA05g27930.1D2
R53	BnaC05g36650.1D2	BnaA05g27950.1D2
R53	BnaC05g36660.1D2	BnaA05g27970.1D2
R53	BnaC05g36670.1D2	BnaA05g27980.1D2
R53	BnaC05g36700.1D2	BnaA05g28020.1D2
R53	BnaC05g36710.1D2	BnaA05g28030.1D2
R53	BnaC05g36730.1D2	BnaA05g28050.1D2
R53	BnaC05g36770.1D2	BnaA05g28080.1D2
R53	BnaC05g36780.1D2	BnaA05g28090.1D2
R53	BnaC05g36790.1D2	BnaA05g28100.1D2
R53	BnaC05g36800.1D2	BnaA05g28110.1D2
R53	BnaC05g36830.1D2	BnaA05g28140.1D2
R53	BnaC05g36850.1D2	BnaA05g28170.1D2
R53	BnaC05g36900.1D2	BnaA05g28190.1D2
R53	BnaC05g36930.1D2	BnaA05g28300.1D2
R53	BnaC05g36940.1D2	BnaA05g28300.1D2
R53	BnaC05g37010.1D2	BnaA05g28380.1D2
R53	BnaC05g37040.1D2	BnaA05g28390.1D2
R53	BnaC05g37050.1D2	BnaA05g28400.1D2
R53	BnaC05g37160.1D2	BnaA05g28480.1D2
R53	BnaC05g37180.1D2	BnaA05g28500.1D2
R53	BnaC05g37220.1D2	BnaA05g28550.1D2
R53	BnaC05g37230.1D2	BnaA05g28560.1D2
R53	BnaC05g37240.1D2	BnaA05g28570.1D2
R53	BnaC05g37260.1D2	BnaA05g28590.1D2
R53	BnaC05g37270.1D2	BnaA05g29130.1D2
R53	BnaC05g37290.1D2	BnaA05g28600.1D2
R53	BnaC05g37380.1D2	BnaA05g29120.1D2
R53	BnaC05g37410.1D2	BnaA05g28720.1D2
R53	BnaC05g37440.1D2	BnaA05g28740.1D2
R53	BnaC05g37550.1D2	BnaA05g28830.1D2
R53	BnaC05g37640.1D2	BnaA05g28900.1D2
R53	BnaC05g37650.1D2	BnaA05g28910.1D2
R53	BnaC05g37730.1D2	BnaA05g28920.1D2
R53	BnaC05g37740.1D2	BnaA05g28930.1D2
R53	BnaC05g37900.1D2	BnaA05g29060.1D2
R53	BnaC05g37940.1D2	BnaA05g29100.1D2
R53	BnaC05g38010.1D2	BnaA05g29250.1D2
R53	BnaC05g38050.1D2	BnaA05g29210.1D2
R53	BnaC05g38070.1D2	BnaA05g29190.1D2
R53	BnaC05g38170.1D2	BnaA05g28230.1D2
R53	BnaC05g38180.1D2	BnaA05g28220.1D2
R53	BnaC05g38200.1D2	BnaA05g29340.1D2
R53	BnaC05g38210.1D2	BnaA05g29350.1D2
R53	BnaC05g38220.1D2	BnaA05g29360.1D2
R53	BnaC05g38230.1D2	BnaA05g29370.1D2

R53	BnaC05g38250.1D2	BnaA05g29400.1D2
R53	BnaC05g38370.1D2	BnaA05g29500.1D2
R53	BnaC05g38390.1D2	BnaA05g29510.1D2
R53	BnaC05g38440.1D2	BnaA05g29560.1D2
R53	BnaC05g38470.1D2	BnaA05g29600.1D2
R53	BnaC05g38480.1D2	BnaA05g29610.1D2
R53	BnaC05g38530.1D2	BnaA05g29650.1D2
R53	BnaC05g38610.1D2	BnaA05g29700.1D2
R53	BnaC05g38630.1D2	BnaA05g29710.1D2
R53	BnaC05g38680.1D2	BnaA05g29780.1D2
R53	BnaC05g38750.1D2	BnaA05g30830.1D2
R53	BnaC05g38760.1D2	BnaA05g30840.1D2
R53	BnaC05g38790.1D2	BnaA05g30870.1D2
R53	BnaC05g38810.1D2	BnaA05g30890.1D2
R53	BnaC05g38820.1D2	BnaA05g30080.1D2
R53	BnaC05g38850.1D2	BnaA05g30110.1D2
R53	BnaC05g38900.1D2	BnaA05g30130.1D2
R53	BnaC05g38930.1D2	BnaA05g30160.1D2
R53	BnaC05g38950.1D2	BnaA05g30180.1D2
R53	BnaC05g39120.1D2	BnaA05g30330.1D2
R53	BnaC05g39170.1D2	BnaA05g30380.1D2
R53	BnaC05g39180.1D2	BnaA05g30390.1D2
R53	BnaC05g39250.1D2	BnaA05g30500.1D2
R53	BnaC05g39360.1D2	BnaA05g30600.1D2
R53	BnaC05g39380.1D2	BnaA05g30620.1D2
R53	BnaC05g39390.1D2	BnaA05g30620.1D2
R53	BnaC05g39470.1D2	BnaA05g30700.1D2
R53	BnaC05g39560.1D2	BnaA05g30780.1D2
R53	BnaC05g39570.1D2	BnaA05g30790.1D2
R53	BnaC05g39640.1D2	BnaA05g30940.1D2
R53	BnaC05g39730.1D2	BnaA05g30070.1D2
R53	BnaC05g39810.1D2	BnaA05g29950.1D2
R53	BnaC05g39820.1D2	BnaA05g29930.1D2
R53	BnaC05g39900.1D2	BnaA05g31050.1D2
R53	BnaC05g40140.1D2	BnaA05g31130.1D2
R53	BnaC05g40190.1D2	BnaA05g31170.1D2
R53	BnaC05g40200.1D2	BnaA05g31180.1D2
R53	BnaC05g40380.1D2	BnaA05g31370.1D2
R53	BnaC05g40410.1D2	BnaA05g31410.1D2
R53	BnaC05g40430.1D2	BnaA05g31420.1D2
R53	BnaC05g40460.1D2	BnaA05g31450.1D2
R53	BnaC05g40470.1D2	BnaA05g31460.1D2
R53	BnaC05g40580.1D2	BnaA05g31580.1D2
R53	BnaC05g40730.1D2	BnaA05g31710.1D2
R53	BnaC05g40800.1D2	BnaA05g31780.1D2
R53	BnaC05g40810.1D2	BnaA05g31780.1D2
R53	BnaC05g40830.1D2	BnaA05g31800.1D2
R53	BnaC05g40840.1D2	BnaA05g31810.1D2
R53	BnaC05g40850.1D2	BnaA05g31820.1D2
R53	BnaC05g40940.1D2	BnaA05g31930.1D2
R53	BnaC05g40970.1D2	BnaA05g31960.1D2

R53	BnaC05g41050.1D2	BnaA05g32080.1D2
R53	BnaC05g41060.1D2	BnaA05g32090.1D2
R53	BnaC05g41070.1D2	BnaA05g32100.1D2
R53	BnaC05g41090.1D2	BnaA05g32110.1D2
R53	BnaC05g41110.1D2	BnaA05g32140.1D2
R53	BnaC05g41130.1D2	BnaA05g32150.1D2
R53	BnaC05g41150.1D2	BnaA05g32160.1D2
R53	BnaC05g41160.1D2	BnaA05g32170.1D2
R53	BnaC05g41170.1D2	BnaA05g32180.1D2
R53	BnaC05g41220.1D2	BnaA05g32240.1D2
R53	BnaC05g41250.1D2	BnaA05g32260.1D2
R53	BnaC05g41370.1D2	BnaA05g32370.1D2
R53	BnaC05g41380.1D2	BnaA05g32380.1D2
R53	BnaC05g41550.1D2	BnaA05g32540.1D2
R53	BnaC05g41590.1D2	BnaA05g34690.1D2
R53	BnaC05g41660.1D2	BnaA05g32640.1D2
R53	BnaC05g41680.1D2	BnaA05g32660.1D2
R53	BnaC05g41690.1D2	BnaA05g32670.1D2
R53	BnaC05g41740.1D2	BnaA05g32710.1D2
R53	BnaC05g41760.1D2	BnaA05g32730.1D2
R53	BnaC05g41890.1D2	BnaA05g33530.1D2
R53	BnaC05g41940.1D2	BnaA05g33490.1D2
R53	BnaC05g41950.1D2	BnaA05g33480.1D2
R53	BnaC05g41960.1D2	BnaA05g33470.1D2
R53	BnaC05g42130.1D2	BnaA05g33720.1D2
R53	BnaC05g42260.1D2	BnaA05g33860.1D2
R53	BnaC05g42280.1D2	BnaA05g33880.1D2
R53	BnaC05g42360.1D2	BnaA05g33360.1D2
R53	BnaC05g42380.1D2	BnaA05g33350.1D2
R53	BnaC05g42390.1D2	BnaA05g33330.1D2
R53	BnaC05g42490.1D2	BnaA05g33270.1D2
R53	BnaC05g42510.1D2	BnaA05g33250.1D2
R53	BnaC05g43060.1D2	BnaA05g34000.1D2
R53	BnaC05g43140.1D2	BnaA05g32870.1D2
R53	BnaC05g43150.1D2	BnaA05g32870.1D2
R53	BnaC05g43160.1D2	BnaA05g32870.1D2
R53	BnaC05g43180.1D2	BnaA05g32890.1D2
R53	BnaC05g43230.1D2	BnaA05g32930.1D2
R53	BnaC05g43250.1D2	BnaA05g32950.1D2
R53	BnaC05g43270.1D2	BnaA05g32970.1D2
R53	BnaC05g43280.1D2	BnaA05g33240.1D2
R53	BnaC05g43310.1D2	BnaA05g33200.1D2
R53	BnaC05g43340.1D2	BnaA05g33190.1D2
R53	BnaC05g43350.1D2	BnaA05g33180.1D2
R53	BnaC05g43360.1D2	BnaA05g33170.1D2
R53	BnaC05g43430.1D2	BnaA05g33130.1D2
R53	BnaC05g43490.1D2	BnaA05g33080.1D2
R53	BnaC05g43570.1D2	BnaA05g33020.1D2
R53	BnaC05g43700.1D2	BnaA05g33630.1D2
R53	BnaC05g43760.1D2	BnaA05g34300.1D2
R53	BnaC05g43770.1D2	BnaA05g34310.1D2

R53	BnaC05g43780.1D2	BnaA05g34320.1D2
R53	BnaC05g43850.1D2	BnaA05g34390.1D2
R53	BnaC05g44080.1D2	BnaA05g34970.1D2
R53	BnaC05g44100.1D2	BnaA05g34950.1D2
R53	BnaC05g44110.1D2	BnaA05g34940.1D2
R53	BnaC05g44140.1D2	BnaA05g34910.1D2
R53	BnaC05g44160.1D2	BnaA05g34880.1D2
R53	BnaC05g44220.1D2	BnaA05g34820.1D2
R53	BnaC05g44230.1D2	BnaA05g35030.1D2
R53	BnaC05g44250.1D2	BnaA05g35010.1D2
R53	BnaC05g44260.1D2	BnaA05g34800.1D2
R53	BnaC05g44280.1D2	BnaA05g34780.1D2
R53	BnaC05g44290.1D2	BnaA05g34770.1D2
R53	BnaC05g44320.1D2	BnaA05g34740.1D2
R53	BnaC05g44360.1D2	BnaA05g34700.1D2
R53	BnaC05g44390.1D2	BnaA05g34660.1D2
R53	BnaC05g44410.1D2	BnaA05g34640.1D2
R53	BnaC05g44460.1D2	BnaA05g34590.1D2
R53	BnaC05g44470.1D2	BnaA05g34580.1D2
R53	BnaC05g44500.1D2	BnaA05g34560.1D2
RS_10_7	BnaC05g29290.1D2	BnaA05g19910.1D2
RS_10_7	BnaC05g32270.1D2	BnaA05g23930.1D2
RS_10_7	BnaC05g32610.1D2	BnaA05g24050.1D2
RS_10_7	BnaC05g32690.1D2	BnaA05g24090.1D2
RS_10_7	BnaC05g32870.1D2	BnaA05g26460.1D2
RS_10_7	BnaC05g32930.1D2	BnaA05g24240.1D2
RS_10_7	BnaC05g32990.1D2	BnaA05g24280.1D2
RS_10_7	BnaC05g33020.1D2	BnaA05g24310.1D2
RS_10_7	BnaC05g33130.1D2	BnaA05g24420.1D2
RS_10_7	BnaC05g33520.1D2	BnaA05g25120.1D2
RS_10_7	BnaC05g33530.1D2	BnaA05g25350.1D2
RS_10_7	BnaC05g33830.1D2	BnaA05g24920.1D2
RS_10_7	BnaC05g33840.1D2	BnaA05g24930.1D2
RS_10_7	BnaC05g34110.1D2	BnaA05g25240.1D2
RS_10_7	BnaC05g34180.1D2	BnaA05g25300.1D2
RS_10_7	BnaC05g34210.1D2	BnaA05g25330.1D2
RS_10_7	BnaC05g34340.1D2	BnaA05g25490.1D2
RS_10_7	BnaC05g34340.1D2	BnaA05g25520.1D2
RS_10_7	BnaC05g34470.1D2	BnaA05g25610.1D2
RS_10_7	BnaC05g34640.1D2	BnaA05g25720.1D2
RS_10_7	BnaC05g34740.1D2	BnaA05g25790.1D2
RS_10_7	BnaC05g34820.1D2	BnaA05g25870.1D2
RS_10_7	BnaC05g34960.1D2	BnaA05g26110.1D2
RS_10_7	BnaC05g35110.1D2	BnaA05g26210.1D2
RS_10_7	BnaC05g35260.1D2	BnaA05g26360.1D2
RS_10_7	BnaC05g35940.1D2	BnaA05g27100.1D2
RS_10_7	BnaC05g36010.1D2	BnaA05g27220.1D2
RS_10_7	BnaC05g36280.1D2	BnaA05g27580.1D2
RS_10_7	BnaC05g36590.1D2	BnaA05g27900.1D2
RS_10_7	BnaC05g36710.1D2	BnaA05g28030.1D2
RS_10_7	BnaC05g36780.1D2	BnaA05g28090.1D2

RS_10_7	BnaC05g36830.1D2	BnaA05g28140.1D2
RS_10_7	BnaC05g36900.1D2	BnaA05g28190.1D2
RS_10_7	BnaC05g36940.1D2	BnaA05g28300.1D2
RS_10_7	BnaC05g36970.1D2	BnaA05g28330.1D2
RS_10_7	BnaC05g37080.1D2	BnaA05g28430.1D2
RS_10_7	BnaC05g37640.1D2	BnaA05g28900.1D2
RS_10_7	BnaC05g37940.1D2	BnaA05g29100.1D2
RS_10_7	BnaC05g38000.1D2	BnaA05g29260.1D2
RS_10_7	BnaC05g38050.1D2	BnaA05g29210.1D2
RS_10_7	BnaC05g38390.1D2	BnaA05g29510.1D2
RS_10_7	BnaC05g38410.1D2	BnaA05g29530.1D2
RS_10_7	BnaC05g38610.1D2	BnaA05g29700.1D2
RS_10_7	BnaC05g38750.1D2	BnaA05g30830.1D2
RS_10_7	BnaC05g38760.1D2	BnaA05g30840.1D2
RS_10_7	BnaC05g38980.1D2	BnaA05g30200.1D2
RS_10_7	BnaC05g39270.1D2	BnaA05g30520.1D2
RS_10_7	BnaC05g39290.1D2	BnaA05g30530.1D2
RS_10_7	BnaC05g39380.1D2	BnaA05g30620.1D2
RS_10_7	BnaC05g39390.1D2	BnaA05g30620.1D2
RS_10_7	BnaC05g39410.1D2	BnaA05g30640.1D2
RS_10_7	BnaC05g39600.1D2	BnaA05g30800.1D2
RS_10_7	BnaC05g39640.1D2	BnaA05g30940.1D2
RS_10_7	BnaC05g39730.1D2	BnaA05g30070.1D2
RS_10_7	BnaC05g39900.1D2	BnaA05g31050.1D2
RS_10_7	BnaC05g40190.1D2	BnaA05g31170.1D2
RS_10_7	BnaC05g40520.1D2	BnaA05g31520.1D2
RS_10_7	BnaC05g40800.1D2	BnaA05g31780.1D2
RS_10_7	BnaC05g40840.1D2	BnaA05g31810.1D2
RS_10_7	BnaC05g40870.1D2	BnaA05g31830.1D2
RS_10_7	BnaC05g40880.1D2	BnaA05g31840.1D2
RS_10_7	BnaC05g41090.1D2	BnaA05g32110.1D2
RS_10_7	BnaC05g41110.1D2	BnaA05g32140.1D2
RS_10_7	BnaC05g41550.1D2	BnaA05g32540.1D2
RS_10_7	BnaC05g41760.1D2	BnaA05g32730.1D2
RS_10_7	BnaC05g41950.1D2	BnaA05g33480.1D2
RS_10_7	BnaC05g42120.1D2	BnaA05g33370.1D2
RS_10_7	BnaC05g42200.1D2	BnaA05g33790.1D2
RS_10_7	BnaC05g42260.1D2	BnaA05g33860.1D2
RS_10_7	BnaC05g42360.1D2	BnaA05g33360.1D2
RS_10_7	BnaC05g42930.1D2	BnaA05g34120.1D2
RS_10_7	BnaC05g43060.1D2	BnaA05g34000.1D2
RS_10_7	BnaC05g43140.1D2	BnaA05g32870.1D2
RS_10_7	BnaC05g43150.1D2	BnaA05g32870.1D2
RS_10_7	BnaC05g43270.1D2	BnaA05g32970.1D2
RS_10_7	BnaC05g43310.1D2	BnaA05g33200.1D2
RS_10_7	BnaC05g43340.1D2	BnaA05g33190.1D2
RS_10_7	BnaC05g43520.1D2	BnaA05g33050.1D2
RS_10_7	BnaC05g43780.1D2	BnaA05g34320.1D2
RS_10_7	BnaC05g44090.1D2	BnaA05g34960.1D2
RS_10_7	BnaC05g44160.1D2	BnaA05g34880.1D2
RS_10_7	BnaC05g44230.1D2	BnaA05g35030.1D2

RS_10_7	BnaC05g44260.1D2	BnaA05g34800.1D2
RS_10_7	BnaC05g44320.1D2	BnaA05g34740.1D2
RS_10_7	BnaC05g44360.1D2	BnaA05g34700.1D2
RS_10_7	BnaC05g44410.1D2	BnaA05g34640.1D2
RS_10_7	BnaC05g44460.1D2	BnaA05g34590.1D2
RS_10_7	BnaC05g44470.1D2	BnaA05g34580.1D2

List of HE genes (C06 to A07)

**Accession**	**Lost gene**	**Duplicated HE gene**
H149	BnaC06g26380.1D2	BnaA07g33570.1D2
H149	BnaC06g30890.1D2	BnaA07g35520.1D2
H149	BnaC06g30930.1D2	BnaA07g35440.1D2
H149	BnaC06g31070.1D2	BnaA07g31570.1D2
H149	BnaC06g31140.1D2	BnaA07g31690.1D2
H149	BnaC06g31170.1D2	BnaA07g31740.1D2
H149	BnaC06g31190.1D2	BnaA07g31750.1D2
H149	BnaC06g31210.1D2	BnaA07g31770.1D2
H149	BnaC06g31230.1D2	BnaA07g31780.1D2
H149	BnaC06g31720.1D2	BnaA07g29860.1D2
H149	BnaC06g31900.1D2	BnaA07g32080.1D2
H149	BnaC06g31950.1D2	BnaA07g32130.1D2
H149	BnaC06g31960.1D2	BnaA07g32140.1D2
H149	BnaC06g32010.1D2	BnaA07g32200.1D2
H149	BnaC06g32070.1D2	BnaA07g32240.1D2
H149	BnaC06g32270.1D2	BnaA07g32440.1D2
H149	BnaC06g32430.1D2	BnaA07g32600.1D2
H149	BnaC06g32440.1D2	BnaA07g32600.1D2
H149	BnaC06g32490.1D2	BnaA07g32650.1D2
H149	BnaC06g32590.1D2	BnaA07g32770.1D2
H149	BnaC06g32620.1D2	BnaA07g32790.1D2
H149	BnaC06g32680.1D2	BnaA07g32850.1D2
H149	BnaC06g32840.1D2	BnaA07g32980.1D2
H149	BnaC06g32850.1D2	BnaA07g32990.1D2
H149	BnaC06g33010.1D2	BnaA07g33110.1D2
H149	BnaC06g33330.1D2	BnaA07g33410.1D2
H149	BnaC06g33390.1D2	BnaA07g33460.1D2
H149	BnaC06g33430.1D2	BnaA07g33490.1D2
H149	BnaC06g33440.1D2	BnaA07g33500.1D2
H149	BnaC06g33480.1D2	BnaA07g33540.1D2
H149	BnaC06g33500.1D2	BnaA07g35230.1D2
H149	BnaC06g33510.1D2	BnaA07g35220.1D2
H149	BnaC06g33540.1D2	BnaA07g35170.1D2
H149	BnaC06g33550.1D2	BnaA07g35150.1D2
H149	BnaC06g33570.1D2	BnaA07g35130.1D2
H149	BnaC06g33650.1D2	BnaA07g33570.1D2
H149	BnaC06g33720.1D2	BnaA07g33650.1D2
H149	BnaC06g33740.1D2	BnaA07g33670.1D2
H149	BnaC06g33790.1D2	BnaA07g33700.1D2
H149	BnaC06g33940.1D2	BnaA07g33860.1D2
H149	BnaC06g33950.1D2	BnaA07g33870.1D2
H149	BnaC06g34000.1D2	BnaA07g35250.1D2

H149	BnaC06g34010.1D2	BnaA07g33920.1D2
H149	BnaC06g34020.1D2	BnaA07g33930.1D2
H149	BnaC06g34040.1D2	BnaA07g33950.1D2
H149	BnaC06g34060.1D2	BnaA07g33970.1D2
H149	BnaC06g34120.1D2	BnaA07g34000.1D2
H149	BnaC06g34240.1D2	BnaA07g34120.1D2
H149	BnaC06g34310.1D2	BnaA07g34200.1D2
H149	BnaC06g34390.1D2	BnaA07g34290.1D2
H149	BnaC06g34430.1D2	BnaA07g34330.1D2
H149	BnaC06g34440.1D2	BnaA07g34340.1D2
H149	BnaC06g34510.1D2	BnaA07g34420.1D2
H149	BnaC06g34540.1D2	BnaA07g34450.1D2
H149	BnaC06g34630.1D2	BnaA07g34540.1D2
H149	BnaC06g34830.1D2	BnaA07g34700.1D2
H149	BnaC06g34900.1D2	BnaA07g34790.1D2
H149	BnaC06g34980.1D2	BnaA07g34850.1D2
H149	BnaC06g34990.1D2	BnaA07g34860.1D2
H149	BnaC06g35000.1D2	BnaA07g34870.1D2
H149	BnaC06g35040.1D2	BnaA07g34900.1D2
H149	BnaC06g35290.1D2	BnaA07g35020.1D2
H149	BnaC06g35340.1D2	BnaA07g35050.1D2
H149	BnaC06g35360.1D2	BnaA07g35080.1D2
RS_7_6	BnaC06g31430.1D2	BnaA07g31940.1D2
RS_7_6	BnaC06g31450.1D2	BnaA07g31960.1D2
RS_7_6	BnaC06g31460.1D2	BnaA07g31970.1D2
RS_7_6	BnaC06g31470.1D2	BnaA07g31980.1D2
RS_7_6	BnaC06g31720.1D2	BnaA07g29860.1D2
RS_7_6	BnaC06g31980.1D2	BnaA07g32160.1D2
RS_7_6	BnaC06g32010.1D2	BnaA07g32200.1D2
RS_7_6	BnaC06g32040.1D2	BnaA07g32200.1D2
RS_7_6	BnaC06g32260.1D2	BnaA07g32420.1D2
RS_7_6	BnaC06g32270.1D2	BnaA07g32440.1D2
RS_7_6	BnaC06g32430.1D2	BnaA07g32600.1D2
RS_7_6	BnaC06g32440.1D2	BnaA07g32600.1D2
RS_7_6	BnaC06g32490.1D2	BnaA07g32650.1D2
RS_7_6	BnaC06g32560.1D2	BnaA07g32730.1D2
RS_7_6	BnaC06g32590.1D2	BnaA07g32770.1D2
RS_7_6	BnaC06g32620.1D2	BnaA07g32790.1D2
RS_7_6	BnaC06g32680.1D2	BnaA07g32850.1D2
RS_7_6	BnaC06g32770.1D2	BnaA07g32920.1D2
RS_7_6	BnaC06g32840.1D2	BnaA07g32980.1D2
RS_7_6	BnaC06g32850.1D2	BnaA07g32990.1D2
RS_7_6	BnaC06g33010.1D2	BnaA07g33110.1D2
RS_7_6	BnaC06g33260.1D2	BnaA07g33360.1D2
RS_7_6	BnaC06g33330.1D2	BnaA07g33410.1D2
RS_7_6	BnaC06g33340.1D2	BnaA07g33420.1D2
RS_7_6	BnaC06g33480.1D2	BnaA07g33540.1D2
RS_7_6	BnaC06g33500.1D2	BnaA07g35230.1D2
RS_7_6	BnaC06g33510.1D2	BnaA07g35220.1D2
RS_7_6	BnaC06g33540.1D2	BnaA07g35170.1D2
RS_7_6	BnaC06g33550.1D2	BnaA07g35150.1D2

RS_7_6	BnaC06g33570.1D2	BnaA07g35130.1D2

List of HE genes (C08 to A09)

**Accession**	**Lost gene**	**Duplicated HE gene**
Chuosenshu	BnaC08g28950.1D2	BnaA09g40970.1D2
Chuosenshu	BnaC08g38700.1D2	BnaA09g52150.1D2
Chuosenshu	BnaC08g38780.1D2	BnaA09g52080.1D2
Chuosenshu	BnaC08g38820.1D2	BnaA09g52040.1D2
Chuosenshu	BnaC08g38860.1D2	BnaA09g52000.1D2
Chuosenshu	BnaC08g38870.1D2	BnaA09g51990.1D2
Chuosenshu	BnaC08g38880.1D2	BnaA09g51980.1D2
Chuosenshu	BnaC08g38890.1D2	BnaA09g51970.1D2
Chuosenshu	BnaC08g38910.1D2	BnaA09g51950.1D2
Chuosenshu	BnaC08g39040.1D2	BnaA09g51240.1D2
Chuosenshu	BnaC08g39050.1D2	BnaA09g51230.1D2
Chuosenshu	BnaC08g39090.1D2	BnaA09g51170.1D2
Chuosenshu	BnaC08g39240.1D2	BnaA09g51540.1D2
Chuosenshu	BnaC08g39260.1D2	BnaA09g51520.1D2
Chuosenshu	BnaC08g39270.1D2	BnaA09g51510.1D2
Chuosenshu	BnaC08g39300.1D2	BnaA09g51130.1D2
Chuosenshu	BnaC08g39320.1D2	BnaA09g51120.1D2
Chuosenshu	BnaC08g39340.1D2	BnaA09g51110.1D2
Chuosenshu	BnaC08g39380.1D2	BnaA09g51070.1D2
Chuosenshu	BnaC08g39410.1D2	BnaA09g51040.1D2
Chuosenshu	BnaC08g39420.1D2	BnaA09g51030.1D2
Chuosenshu	BnaC08g39480.1D2	BnaA09g50970.1D2
Chuosenshu	BnaC08g39510.1D2	BnaA09g50940.1D2
Chuosenshu	BnaC08g39540.1D2	BnaA09g50920.1D2
Chuosenshu	BnaC08g39570.1D2	BnaA09g50900.1D2
Chuosenshu	BnaC08g39580.1D2	BnaA09g50870.1D2
Chuosenshu	BnaC08g39590.1D2	BnaA09g50870.1D2
Chuosenshu	BnaC08g39620.1D2	BnaA09g50820.1D2
Chuosenshu	BnaC08g39680.1D2	BnaA09g50790.1D2
Chuosenshu	BnaC08g39690.1D2	BnaA09g50780.1D2
Chuosenshu	BnaC08g39700.1D2	BnaA09g50770.1D2
Chuosenshu	BnaC08g39710.1D2	BnaA09g50760.1D2
Chuosenshu	BnaC08g39720.1D2	BnaA09g50750.1D2
Chuosenshu	BnaC08g39770.1D2	BnaA09g50720.1D2
Chuosenshu	BnaC08g39810.1D2	BnaA09g50680.1D2
Chuosenshu	BnaC08g39820.1D2	BnaA09g50670.1D2
Chuosenshu	BnaC08g39890.1D2	BnaA09g50630.1D2
Chuosenshu	BnaC08g39900.1D2	BnaA09g50620.1D2
Chuosenshu	BnaC08g39930.1D2	BnaA09g50590.1D2
Chuosenshu	BnaC08g39970.1D2	BnaA09g50560.1D2
Chuosenshu	BnaC08g39980.1D2	BnaA09g50550.1D2
Chuosenshu	BnaC08g39990.1D2	BnaA09g50540.1D2
Chuosenshu	BnaC08g40080.1D2	BnaA09g51630.1D2
Chuosenshu	BnaC08g40100.1D2	BnaA09g51640.1D2
Chuosenshu	BnaC08g40110.1D2	BnaA09g51640.1D2
Chuosenshu	BnaC08g40130.1D2	BnaA09g51650.1D2
Chuosenshu	BnaC08g40140.1D2	BnaA09g51650.1D2

Chuosenshu	BnaC08g40180.1D2	BnaA09g51690.1D2
Chuosenshu	BnaC08g40190.1D2	BnaA09g51700.1D2
Chuosenshu	BnaC08g40260.1D2	BnaA09g50390.1D2
Chuosenshu	BnaC08g40280.1D2	BnaA09g50370.1D2
Chuosenshu	BnaC08g40300.1D2	BnaA09g50360.1D2
Chuosenshu	BnaC08g40320.1D2	BnaA09g50360.1D2
Chuosenshu	BnaC08g40330.1D2	BnaA09g50340.1D2
Chuosenshu	BnaC08g40340.1D2	BnaA09g50330.1D2
Chuosenshu	BnaC08g40360.1D2	BnaA09g50320.1D2
Chuosenshu	BnaC08g40370.1D2	BnaA09g50310.1D2
Chuosenshu	BnaC08g40470.1D2	BnaA09g51770.1D2
Chuosenshu	BnaC08g40490.1D2	BnaA09g51800.1D2
Chuosenshu	BnaC08g40500.1D2	BnaA09g51810.1D2
Chuosenshu	BnaC08g40520.1D2	BnaA09g51820.1D2
Chuosenshu	BnaC08g40560.1D2	BnaA09g51860.1D2
Chuosenshu	BnaC08g40710.1D2	BnaA09g51140.1D2
Chuosenshu	BnaC08g40720.1D2	BnaA09g52420.1D2
Chuosenshu	BnaC08g40760.1D2	BnaA09g51450.1D2
Chuosenshu	BnaC08g40880.1D2	BnaA09g52190.1D2
Chuosenshu	BnaC08g40900.1D2	BnaA09g52210.1D2
Chuosenshu	BnaC08g40910.1D2	BnaA09g52220.1D2
Chuosenshu	BnaC08g40920.1D2	BnaA09g52230.1D2
Chuosenshu	BnaC08g40940.1D2	BnaA09g52250.1D2
Chuosenshu	BnaC08g41050.1D2	BnaA09g52310.1D2
Chuosenshu	BnaC08g41080.1D2	BnaA09g52350.1D2
Chuosenshu	BnaC08g41090.1D2	BnaA09g52360.1D2
Chuosenshu	BnaC08g41100.1D2	BnaA09g52370.1D2
Chuosenshu	BnaC08g41120.1D2	BnaA09g52390.1D2
Chuosenshu	BnaC08g41190.1D2	BnaA09g52460.1D2
Chuosenshu	BnaC08g41210.1D2	BnaA09g52470.1D2
Chuosenshu	BnaC08g41240.1D2	BnaA09g52520.1D2
Chuosenshu	BnaC08g41260.1D2	BnaA09g52540.1D2
Chuosenshu	BnaC08g41400.1D2	BnaA09g51750.1D2
DH5	BnaC08g35730.1D2	BnaA09g47460.1D2
DH5	BnaC08g35780.1D2	BnaA09g47520.1D2
DH5	BnaC08g35800.1D2	BnaA09g47540.1D2
DH5	BnaC08g35830.1D2	BnaA09g47570.1D2
DH5	BnaC08g35840.1D2	BnaA09g47570.1D2
DH5	BnaC08g35930.1D2	BnaA09g47690.1D2
DH5	BnaC08g35960.1D2	BnaA09g47720.1D2
DH5	BnaC08g35970.1D2	BnaA09g47730.1D2
DH5	BnaC08g36140.1D2	BnaA09g47890.1D2
DH5	BnaC08g36150.1D2	BnaA09g47890.1D2
DH5	BnaC08g36170.1D2	BnaA09g47900.1D2
DH5	BnaC08g36190.1D2	BnaA09g47920.1D2
DH5	BnaC08g36210.1D2	BnaA09g47940.1D2
DH5	BnaC08g36280.1D2	BnaA09g48000.1D2
DH5	BnaC08g36300.1D2	BnaA09g48020.1D2
DH5	BnaC08g36340.1D2	BnaA09g48070.1D2
DH5	BnaC08g36400.1D2	BnaA09g48140.1D2
DH5	BnaC08g36420.1D2	BnaA09g48160.1D2

DH5	BnaC08g36440.1D2	BnaA09g48180.1D2
DH5	BnaC08g36480.1D2	BnaA09g48230.1D2
DH5	BnaC08g36490.1D2	BnaA09g48240.1D2
DH5	BnaC08g36500.1D2	BnaA09g48260.1D2
DH5	BnaC08g36510.1D2	BnaA09g48270.1D2
DH5	BnaC08g36520.1D2	BnaA09g48280.1D2
DH5	BnaC08g36540.1D2	BnaA09g48300.1D2
DH5	BnaC08g36600.1D2	BnaA09g48370.1D2
DH5	BnaC08g36620.1D2	BnaA09g48390.1D2
DH5	BnaC08g36690.1D2	BnaA09g48470.1D2
DH5	BnaC08g36800.1D2	BnaA09g48480.1D2
DH5	BnaC08g36830.1D2	BnaA09g48500.1D2
DH5	BnaC08g36860.1D2	BnaA09g48530.1D2
DH5	BnaC08g36870.1D2	BnaA09g48540.1D2
DH5	BnaC08g36920.1D2	BnaA09g48580.1D2
DH5	BnaC08g36930.1D2	BnaA09g48590.1D2
DH5	BnaC08g36980.1D2	BnaA09g49120.1D2
DH5	BnaC08g37000.1D2	BnaA09g48630.1D2
DH5	BnaC08g37030.1D2	BnaA09g48640.1D2
DH5	BnaC08g37170.1D2	BnaA09g48700.1D2
DH5	BnaC08g37220.1D2	BnaA09g48740.1D2
DH5	BnaC08g37230.1D2	BnaA09g48750.1D2
DH5	BnaC08g37300.1D2	BnaA09g48980.1D2
DH5	BnaC08g37330.1D2	BnaA09g48940.1D2
DH5	BnaC08g37340.1D2	BnaA09g48930.1D2
DH5	BnaC08g37350.1D2	BnaA09g48920.1D2
DH5	BnaC08g37360.1D2	BnaA09g48910.1D2
DH5	BnaC08g37400.1D2	BnaA09g48870.1D2
DH5	BnaC08g37410.1D2	BnaA09g48860.1D2
DH5	BnaC08g37680.1D2	BnaA09g51330.1D2
DH5	BnaC08g37720.1D2	BnaA09g51290.1D2
DH5	BnaC08g39040.1D2	BnaA09g51240.1D2
DH5	BnaC08g39050.1D2	BnaA09g51230.1D2
DH5	BnaC08g39060.1D2	BnaA09g51220.1D2
DH5	BnaC08g39090.1D2	BnaA09g51170.1D2
DH5	BnaC08g39220.1D2	BnaA09g51550.1D2
DH5	BnaC08g39240.1D2	BnaA09g51540.1D2
DH5	BnaC08g39260.1D2	BnaA09g51520.1D2
DH5	BnaC08g39270.1D2	BnaA09g51510.1D2
DH5	BnaC08g39300.1D2	BnaA09g51130.1D2
DH5	BnaC08g39320.1D2	BnaA09g51120.1D2
DH5	BnaC08g39340.1D2	BnaA09g51110.1D2
DH5	BnaC08g39380.1D2	BnaA09g51070.1D2
DH5	BnaC08g39400.1D2	BnaA09g51050.1D2
DH5	BnaC08g39410.1D2	BnaA09g51040.1D2
DH5	BnaC08g39420.1D2	BnaA09g51030.1D2
DH5	BnaC08g39460.1D2	BnaA09g50990.1D2
DH5	BnaC08g39480.1D2	BnaA09g50970.1D2
DH5	BnaC08g39510.1D2	BnaA09g50940.1D2
DH5	BnaC08g39520.1D2	BnaA09g50930.1D2
DH5	BnaC08g39540.1D2	BnaA09g50920.1D2

DH5	BnaC08g39570.1D2	BnaA09g50900.1D2
DH5	BnaC08g39590.1D2	BnaA09g50870.1D2
DH5	BnaC08g39620.1D2	BnaA09g50820.1D2
DH5	BnaC08g39680.1D2	BnaA09g50790.1D2
DH5	BnaC08g39690.1D2	BnaA09g50780.1D2
DH5	BnaC08g39700.1D2	BnaA09g50770.1D2
DH5	BnaC08g39710.1D2	BnaA09g50760.1D2
DH5	BnaC08g39770.1D2	BnaA09g50720.1D2
DH5	BnaC08g39810.1D2	BnaA09g50680.1D2
DH5	BnaC08g39820.1D2	BnaA09g50670.1D2
DH5	BnaC08g39890.1D2	BnaA09g50630.1D2
DH5	BnaC08g39900.1D2	BnaA09g50620.1D2
DH5	BnaC08g39930.1D2	BnaA09g50590.1D2
DH5	BnaC08g39970.1D2	BnaA09g50560.1D2
DH5	BnaC08g39980.1D2	BnaA09g50550.1D2
DH5	BnaC08g39990.1D2	BnaA09g50540.1D2
DH5	BnaC08g40080.1D2	BnaA09g51630.1D2
DH5	BnaC08g40100.1D2	BnaA09g51640.1D2
DH5	BnaC08g40110.1D2	BnaA09g51640.1D2
DH5	BnaC08g40130.1D2	BnaA09g51650.1D2
DH5	BnaC08g40140.1D2	BnaA09g51650.1D2
DH5	BnaC08g40180.1D2	BnaA09g51690.1D2
DH5	BnaC08g40190.1D2	BnaA09g51700.1D2
DH5	BnaC08g40260.1D2	BnaA09g50390.1D2
DH5	BnaC08g40280.1D2	BnaA09g50370.1D2
DH5	BnaC08g40320.1D2	BnaA09g50360.1D2
DH5	BnaC08g40330.1D2	BnaA09g50340.1D2
DH5	BnaC08g40340.1D2	BnaA09g50330.1D2
DH5	BnaC08g40360.1D2	BnaA09g50320.1D2
DH5	BnaC08g40490.1D2	BnaA09g51800.1D2
DH5	BnaC08g40500.1D2	BnaA09g51810.1D2
DH5	BnaC08g40520.1D2	BnaA09g51820.1D2
DH5	BnaC08g40540.1D2	BnaA09g51840.1D2
DH5	BnaC08g40560.1D2	BnaA09g51860.1D2
DH5	BnaC08g40710.1D2	BnaA09g51140.1D2
DH5	BnaC08g40760.1D2	BnaA09g51450.1D2
Pacific	BnaC08g31250.1D2	BnaA09g39770.1D2
Pacific	BnaC08g35730.1D2	BnaA09g47460.1D2
Pacific	BnaC08g35780.1D2	BnaA09g47520.1D2
Pacific	BnaC08g35790.1D2	BnaA09g47530.1D2
Pacific	BnaC08g35800.1D2	BnaA09g47540.1D2
Pacific	BnaC08g35830.1D2	BnaA09g47570.1D2
Pacific	BnaC08g35840.1D2	BnaA09g47570.1D2
Pacific	BnaC08g35870.1D2	BnaA09g47590.1D2
Pacific	BnaC08g35930.1D2	BnaA09g47690.1D2
Pacific	BnaC08g35960.1D2	BnaA09g47720.1D2
Pacific	BnaC08g35970.1D2	BnaA09g47730.1D2
Pacific	BnaC08g36020.1D2	BnaA09g47810.1D2
Pacific	BnaC08g36050.1D2	BnaA09g47830.1D2
Pacific	BnaC08g36090.1D2	BnaA09g47870.1D2
Pacific	BnaC08g36130.1D2	BnaA09g47880.1D2

Pacific	BnaC08g36140.1D2	BnaA09g47890.1D2
Pacific	BnaC08g36150.1D2	BnaA09g47890.1D2
Pacific	BnaC08g36170.1D2	BnaA09g47900.1D2
Pacific	BnaC08g36190.1D2	BnaA09g47920.1D2
Pacific	BnaC08g36200.1D2	BnaA09g47930.1D2
Pacific	BnaC08g36210.1D2	BnaA09g47940.1D2
Pacific	BnaC08g36280.1D2	BnaA09g48000.1D2
Pacific	BnaC08g36300.1D2	BnaA09g48020.1D2
Pacific	BnaC08g36340.1D2	BnaA09g48070.1D2
Pacific	BnaC08g36350.1D2	BnaA09g48080.1D2
Pacific	BnaC08g36360.1D2	BnaA09g48090.1D2
Pacific	BnaC08g36400.1D2	BnaA09g48140.1D2
Pacific	BnaC08g36440.1D2	BnaA09g48180.1D2
Pacific	BnaC08g36480.1D2	BnaA09g48230.1D2
Pacific	BnaC08g36490.1D2	BnaA09g48240.1D2
Pacific	BnaC08g36500.1D2	BnaA09g48260.1D2
Pacific	BnaC08g36510.1D2	BnaA09g48270.1D2
Pacific	BnaC08g36520.1D2	BnaA09g48280.1D2
Pacific	BnaC08g36540.1D2	BnaA09g48300.1D2
Pacific	BnaC08g36600.1D2	BnaA09g48370.1D2
Pacific	BnaC08g36620.1D2	BnaA09g48390.1D2
Pacific	BnaC08g36650.1D2	BnaA09g48420.1D2
Pacific	BnaC08g36670.1D2	BnaA09g48440.1D2
Pacific	BnaC08g36690.1D2	BnaA09g48470.1D2
Pacific	BnaC08g36800.1D2	BnaA09g48480.1D2
Pacific	BnaC08g36830.1D2	BnaA09g48500.1D2
Pacific	BnaC08g36860.1D2	BnaA09g48530.1D2
Pacific	BnaC08g36870.1D2	BnaA09g48540.1D2
Pacific	BnaC08g36920.1D2	BnaA09g48580.1D2
Pacific	BnaC08g36930.1D2	BnaA09g48590.1D2
Pacific	BnaC08g36940.1D2	BnaA09g48600.1D2
Pacific	BnaC08g36970.1D2	BnaA09g49130.1D2
Pacific	BnaC08g36980.1D2	BnaA09g49120.1D2
Pacific	BnaC08g37000.1D2	BnaA09g48630.1D2
Pacific	BnaC08g37030.1D2	BnaA09g48640.1D2
Pacific	BnaC08g37170.1D2	BnaA09g48700.1D2
Pacific	BnaC08g37220.1D2	BnaA09g48740.1D2
Pacific	BnaC08g37230.1D2	BnaA09g48750.1D2
Pacific	BnaC08g37240.1D2	BnaA09g48760.1D2
Pacific	BnaC08g37300.1D2	BnaA09g48980.1D2
Pacific	BnaC08g37330.1D2	BnaA09g48940.1D2
Pacific	BnaC08g37340.1D2	BnaA09g48930.1D2
Pacific	BnaC08g37350.1D2	BnaA09g48920.1D2
Pacific	BnaC08g37360.1D2	BnaA09g48910.1D2
Pacific	BnaC08g37400.1D2	BnaA09g48870.1D2
Pacific	BnaC08g37410.1D2	BnaA09g48860.1D2
Pacific	BnaC08g37650.1D2	BnaA09g51360.1D2
Pacific	BnaC08g37680.1D2	BnaA09g51330.1D2
Pacific	BnaC08g37720.1D2	BnaA09g51290.1D2
Pacific	BnaC08g39000.1D2	BnaA09g51900.1D2
Pacific	BnaC08g39090.1D2	BnaA09g51170.1D2

Pacific	BnaC08g39180.1D2	BnaA09g51590.1D2
Pacific	BnaC08g39240.1D2	BnaA09g51540.1D2
Pacific	BnaC08g39260.1D2	BnaA09g51520.1D2
Pacific	BnaC08g39300.1D2	BnaA09g51130.1D2
Pacific	BnaC08g39320.1D2	BnaA09g51120.1D2
Pacific	BnaC08g39340.1D2	BnaA09g51110.1D2
Pacific	BnaC08g39380.1D2	BnaA09g51070.1D2
Pacific	BnaC08g39400.1D2	BnaA09g51050.1D2
Pacific	BnaC08g39410.1D2	BnaA09g51040.1D2
Pacific	BnaC08g39420.1D2	BnaA09g51030.1D2
Pacific	BnaC08g39440.1D2	BnaA09g51010.1D2
Pacific	BnaC08g39460.1D2	BnaA09g50990.1D2
Pacific	BnaC08g39480.1D2	BnaA09g50970.1D2
Pacific	BnaC08g39510.1D2	BnaA09g50940.1D2
Pacific	BnaC08g39520.1D2	BnaA09g50930.1D2
Pacific	BnaC08g39530.1D2	BnaA09g50920.1D2
Pacific	BnaC08g39540.1D2	BnaA09g50920.1D2
Pacific	BnaC08g39570.1D2	BnaA09g50900.1D2
Pacific	BnaC08g39580.1D2	BnaA09g50870.1D2
Pacific	BnaC08g39590.1D2	BnaA09g50870.1D2
Pacific	BnaC08g39620.1D2	BnaA09g50820.1D2
Pacific	BnaC08g39680.1D2	BnaA09g50790.1D2
Pacific	BnaC08g39690.1D2	BnaA09g50780.1D2
Pacific	BnaC08g39700.1D2	BnaA09g50770.1D2
Pacific	BnaC08g39710.1D2	BnaA09g50760.1D2
Pacific	BnaC08g39720.1D2	BnaA09g50750.1D2
Pacific	BnaC08g39760.1D2	BnaA09g50720.1D2
Pacific	BnaC08g39770.1D2	BnaA09g50720.1D2
Pacific	BnaC08g39810.1D2	BnaA09g50680.1D2
Pacific	BnaC08g39820.1D2	BnaA09g50670.1D2
Pacific	BnaC08g39890.1D2	BnaA09g50630.1D2
Pacific	BnaC08g39970.1D2	BnaA09g50560.1D2
Pacific	BnaC08g39980.1D2	BnaA09g50550.1D2
Pacific	BnaC08g39990.1D2	BnaA09g50540.1D2
Pacific	BnaC08g40080.1D2	BnaA09g51630.1D2
Pacific	BnaC08g40100.1D2	BnaA09g51640.1D2
Pacific	BnaC08g40110.1D2	BnaA09g51640.1D2
Pacific	BnaC08g40130.1D2	BnaA09g51650.1D2
Pacific	BnaC08g40140.1D2	BnaA09g51650.1D2
Pacific	BnaC08g40180.1D2	BnaA09g51690.1D2
Pacific	BnaC08g40190.1D2	BnaA09g51700.1D2
Pacific	BnaC08g40200.1D2	BnaA09g51720.1D2
Pacific	BnaC08g40260.1D2	BnaA09g50390.1D2
Pacific	BnaC08g40280.1D2	BnaA09g50370.1D2
Pacific	BnaC08g40300.1D2	BnaA09g50360.1D2
Pacific	BnaC08g40320.1D2	BnaA09g50360.1D2
Pacific	BnaC08g40340.1D2	BnaA09g50330.1D2
Pacific	BnaC08g40360.1D2	BnaA09g50320.1D2
Pacific	BnaC08g40500.1D2	BnaA09g51810.1D2
Pacific	BnaC08g40520.1D2	BnaA09g51820.1D2
Pacific	BnaC08g40610.1D2	BnaA09g52680.1D2

Pacific	BnaC08g40630.1D2	BnaA09g52710.1D2
Pacific	BnaC08g40640.1D2	BnaA09g52720.1D2
Pacific	BnaC08g40650.1D2	BnaA09g52730.1D2
Pacific	BnaC08g40690.1D2	BnaA09g52770.1D2
Pacific	BnaC08g40710.1D2	BnaA09g51140.1D2
Pacific	BnaC08g40730.1D2	BnaA09g51170.1D2
Pacific	BnaC08g40760.1D2	BnaA09g51450.1D2
Pacific	BnaC08g41370.1D2	BnaA09g52800.1D2
Pacific	BnaC08g41380.1D2	BnaA09g52810.1D2
Sensation	BnaC08g36440.1D2	BnaA09g48180.1D2
Sensation	BnaC08g36520.1D2	BnaA09g48280.1D2
Sensation	BnaC08g36540.1D2	BnaA09g48300.1D2
Sensation	BnaC08g36670.1D2	BnaA09g48440.1D2
Sensation	BnaC08g36690.1D2	BnaA09g48470.1D2
Sensation	BnaC08g36830.1D2	BnaA09g48500.1D2
Sensation	BnaC08g36860.1D2	BnaA09g48530.1D2
Sensation	BnaC08g36870.1D2	BnaA09g48540.1D2
Sensation	BnaC08g36930.1D2	BnaA09g48590.1D2
Sensation	BnaC08g37000.1D2	BnaA09g48630.1D2
Sensation	BnaC08g37030.1D2	BnaA09g48640.1D2
Sensation	BnaC08g37230.1D2	BnaA09g48750.1D2
Sensation	BnaC08g37340.1D2	BnaA09g48930.1D2
Sensation	BnaC08g37400.1D2	BnaA09g48870.1D2
Sensation	BnaC08g37650.1D2	BnaA09g51360.1D2
Sensation	BnaC08g37660.1D2	BnaA09g51350.1D2
Sensation	BnaC08g37720.1D2	BnaA09g51290.1D2
Sensation	BnaC08g38030.1D2	BnaA09g49590.1D2
Sensation	BnaC08g38050.1D2	BnaA09g49610.1D2
Sensation	BnaC08g38060.1D2	BnaA09g49620.1D2
Sensation	BnaC08g38130.1D2	BnaA09g49690.1D2
Sensation	BnaC08g38140.1D2	BnaA09g49700.1D2
Sensation	BnaC08g38230.1D2	BnaA09g49780.1D2
Sensation	BnaC08g38320.1D2	BnaA09g49850.1D2
Sensation	BnaC08g38330.1D2	BnaA09g49860.1D2
Sensation	BnaC08g38370.1D2	BnaA09g49900.1D2
Sensation	BnaC08g38400.1D2	BnaA09g49910.1D2
Sensation	BnaC08g38430.1D2	BnaA09g49930.1D2
Sensation	BnaC08g38490.1D2	BnaA09g49970.1D2
Sensation	BnaC08g38510.1D2	BnaA09g49990.1D2
Sensation	BnaC08g38600.1D2	BnaA09g50090.1D2
Sensation	BnaC08g38610.1D2	BnaA09g50100.1D2
Sensation	BnaC08g38640.1D2	BnaA09g50130.1D2
Sensation	BnaC08g38660.1D2	BnaA09g50150.1D2
Sensation	BnaC08g38670.1D2	BnaA09g50160.1D2
Sensation	BnaC08g38700.1D2	BnaA09g52150.1D2
Sensation	BnaC08g38730.1D2	BnaA09g52130.1D2
Sensation	BnaC08g38780.1D2	BnaA09g52080.1D2
Sensation	BnaC08g38810.1D2	BnaA09g52050.1D2
Sensation	BnaC08g38820.1D2	BnaA09g52040.1D2
Sensation	BnaC08g38970.1D2	BnaA09g51730.1D2
Sensation	BnaC08g39000.1D2	BnaA09g51900.1D2

Sensation	BnaC08g39040.1D2	BnaA09g51240.1D2
Sensation	BnaC08g39050.1D2	BnaA09g51230.1D2
Sensation	BnaC08g39060.1D2	BnaA09g51220.1D2
Sensation	BnaC08g39090.1D2	BnaA09g51170.1D2
Sensation	BnaC08g39180.1D2	BnaA09g51590.1D2
Sensation	BnaC08g39240.1D2	BnaA09g51540.1D2
Sensation	BnaC08g39260.1D2	BnaA09g51520.1D2
Sensation	BnaC08g39300.1D2	BnaA09g51130.1D2
Sensation	BnaC08g39320.1D2	BnaA09g51120.1D2
Sensation	BnaC08g39340.1D2	BnaA09g51110.1D2
Sensation	BnaC08g39380.1D2	BnaA09g51070.1D2
Sensation	BnaC08g39460.1D2	BnaA09g50990.1D2
Sensation	BnaC08g39510.1D2	BnaA09g50940.1D2
Sensation	BnaC08g39520.1D2	BnaA09g50930.1D2
Sensation	BnaC08g39680.1D2	BnaA09g50790.1D2
Sensation	BnaC08g39690.1D2	BnaA09g50780.1D2
Sensation	BnaC08g39700.1D2	BnaA09g50770.1D2
Sensation	BnaC08g39980.1D2	BnaA09g50550.1D2
Sensation	BnaC08g39990.1D2	BnaA09g50540.1D2
Sensation	BnaC08g40030.1D2	BnaA09g50460.1D2
Sensation	BnaC08g40100.1D2	BnaA09g51640.1D2
Sensation	BnaC08g40110.1D2	BnaA09g51640.1D2
Sensation	BnaC08g40130.1D2	BnaA09g51650.1D2
Sensation	BnaC08g40180.1D2	BnaA09g51690.1D2
Sensation	BnaC08g40320.1D2	BnaA09g50360.1D2
Sensation	BnaC08g40330.1D2	BnaA09g50340.1D2
Sensation	BnaC08g40340.1D2	BnaA09g50330.1D2
Sensation	BnaC08g40360.1D2	BnaA09g50320.1D2
Sensation	BnaC08g40500.1D2	BnaA09g51810.1D2
Sensation	BnaC08g40520.1D2	BnaA09g51820.1D2
Sensation	BnaC08g40540.1D2	BnaA09g51840.1D2
Sensation	BnaC08g40560.1D2	BnaA09g51860.1D2
Sensation	BnaC08g40610.1D2	BnaA09g52680.1D2
Sensation	BnaC08g40640.1D2	BnaA09g52720.1D2
Sensation	BnaC08g40710.1D2	BnaA09g51140.1D2
Sensation	BnaC08g40720.1D2	BnaA09g52420.1D2
Sensation	BnaC08g40730.1D2	BnaA09g51170.1D2
Sensation	BnaC08g40760.1D2	BnaA09g51450.1D2
Sensation	BnaC08g40850.1D2	BnaA09g50180.1D2
Sensation	BnaC08g40950.1D2	BnaA09g52260.1D2
Sensation	BnaC08g41010.1D2	BnaA09g52260.1D2
Sensation	BnaC08g41050.1D2	BnaA09g52310.1D2
Sensation	BnaC08g41190.1D2	BnaA09g52460.1D2
Sensation	BnaC08g41260.1D2	BnaA09g52540.1D2
Sensation	BnaC08g41370.1D2	BnaA09g52800.1D2
Sensation	BnaC08g41380.1D2	BnaA09g52810.1D2
G50	BnaC08g29850.1D2	BnaA09g42140.1D2
G50	BnaC08g29860.1D2	BnaA09g42150.1D2
G50	BnaC08g29880.1D2	BnaA09g42160.1D2
G50	BnaC08g29900.1D2	BnaA09g42190.1D2
G50	BnaC08g29930.1D2	BnaA09g42230.1D2

G50	BnaC08g29970.1D2	BnaA09g42280.1D2
G50	BnaC08g30050.1D2	BnaA09g42500.1D2
G50	BnaC08g30070.1D2	BnaA09g42510.1D2
G50	BnaC08g30100.1D2	BnaA09g42590.1D2
G50	BnaC08g30150.1D2	BnaA09g42730.1D2
G50	BnaC08g30170.1D2	BnaA09g42750.1D2
G50	BnaC08g30220.1D2	BnaA09g42840.1D2
G50	BnaC08g30230.1D2	BnaA09g42850.1D2
G50	BnaC08g30250.1D2	BnaA09g42910.1D2
G50	BnaC08g30270.1D2	BnaA09g42930.1D2
G50	BnaC08g30280.1D2	BnaA09g42940.1D2
G50	BnaC08g30290.1D2	BnaA09g42950.1D2
G50	BnaC08g30340.1D2	BnaA09g43000.1D2
G50	BnaC08g30360.1D2	BnaA09g43020.1D2
G50	BnaC08g30420.1D2	BnaA09g43100.1D2
G50	BnaC08g30430.1D2	BnaA09g43110.1D2
G50	BnaC08g30440.1D2	BnaA09g43120.1D2
G50	BnaC08g30450.1D2	BnaA09g43130.1D2
G50	BnaC08g30460.1D2	BnaA09g43150.1D2
G50	BnaC08g30490.1D2	BnaA09g43170.1D2
G50	BnaC08g30520.1D2	BnaA09g43190.1D2
G50	BnaC08g30550.1D2	BnaA09g43220.1D2
G50	BnaC08g30600.1D2	BnaA09g43260.1D2
G50	BnaC08g30630.1D2	BnaA09g43280.1D2
G50	BnaC08g30650.1D2	BnaA09g43300.1D2
G50	BnaC08g30660.1D2	BnaA09g43320.1D2
G50	BnaC08g30740.1D2	BnaA09g43390.1D2
G50	BnaC08g30810.1D2	BnaA09g43460.1D2
G50	BnaC08g30930.1D2	BnaA09g43520.1D2
G50	BnaC08g30960.1D2	BnaA09g43610.1D2
G50	BnaC08g30980.1D2	BnaA09g43610.1D2
G50	BnaC08g31000.1D2	BnaA09g43590.1D2
G50	BnaC08g31020.1D2	BnaA09g44470.1D2
G50	BnaC08g31050.1D2	BnaA09g42730.1D2
G50	BnaC08g31080.1D2	BnaA09g43060.1D2
G50	BnaC08g31330.1D2	BnaA09g42390.1D2
G50	BnaC08g31340.1D2	BnaA09g42400.1D2
G50	BnaC08g31440.1D2	BnaA09g42080.1D2
G50	BnaC08g31450.1D2	BnaA09g42090.1D2
G50	BnaC08g31460.1D2	BnaA09g42100.1D2
G50	BnaC08g31500.1D2	BnaA09g43620.1D2
G50	BnaC08g31510.1D2	BnaA09g43630.1D2
G50	BnaC08g31520.1D2	BnaA09g43650.1D2
G50	BnaC08g31540.1D2	BnaA09g43670.1D2
G50	BnaC08g31550.1D2	BnaA09g43680.1D2
G50	BnaC08g31560.1D2	BnaA09g43690.1D2
G50	BnaC08g31570.1D2	BnaA09g43700.1D2
G50	BnaC08g31620.1D2	BnaA09g43750.1D2
G50	BnaC08g31780.1D2	BnaA09g43850.1D2
G50	BnaC08g31820.1D2	BnaA09g43900.1D2
G50	BnaC08g31830.1D2	BnaA09g43910.1D2

G50	BnaC08g31840.1D2	BnaA09g43920.1D2
G50	BnaC08g31850.1D2	BnaA09g43930.1D2
G50	BnaC08g31880.1D2	BnaA09g43960.1D2
G50	BnaC08g31900.1D2	BnaA09g43980.1D2
G50	BnaC08g31910.1D2	BnaA09g43990.1D2
G50	BnaC08g31930.1D2	BnaA09g44010.1D2
G50	BnaC08g31990.1D2	BnaA09g44070.1D2
G50	BnaC08g32130.1D2	BnaA09g44170.1D2
G50	BnaC08g32150.1D2	BnaA09g44190.1D2
G50	BnaC08g32180.1D2	BnaA09g44240.1D2
G50	BnaC08g32240.1D2	BnaA09g44290.1D2
G50	BnaC08g32250.1D2	BnaA09g44300.1D2
G50	BnaC08g32310.1D2	BnaA09g44330.1D2
G50	BnaC08g32340.1D2	BnaA09g44370.1D2
G50	BnaC08g32360.1D2	BnaA09g44390.1D2
G50	BnaC08g32410.1D2	BnaA09g44510.1D2
G50	BnaC08g32420.1D2	BnaA09g44520.1D2
G50	BnaC08g32430.1D2	BnaA09g44530.1D2
G50	BnaC08g32450.1D2	BnaA09g44550.1D2
G50	BnaC08g32470.1D2	BnaA09g44570.1D2
G50	BnaC08g32480.1D2	BnaA09g44590.1D2
G50	BnaC08g32490.1D2	BnaA09g44600.1D2
G50	BnaC08g32510.1D2	BnaA09g44620.1D2
G50	BnaC08g32540.1D2	BnaA09g44680.1D2
G50	BnaC08g32550.1D2	BnaA09g44700.1D2
G50	BnaC08g32600.1D2	BnaA09g44790.1D2
G50	BnaC08g32610.1D2	BnaA09g44810.1D2
G50	BnaC08g32620.1D2	BnaA09g44830.1D2
G50	BnaC08g32640.1D2	BnaA09g44840.1D2
G50	BnaC08g32660.1D2	BnaA09g44880.1D2
G50	BnaC08g32670.1D2	BnaA09g44890.1D2
G50	BnaC08g32700.1D2	BnaA09g44910.1D2
G50	BnaC08g32730.1D2	BnaA09g44940.1D2
G50	BnaC08g32750.1D2	BnaA09g46540.1D2
G50	BnaC08g32810.1D2	BnaA09g45000.1D2
G50	BnaC08g32820.1D2	BnaA09g45010.1D2
G50	BnaC08g32830.1D2	BnaA09g45020.1D2
G50	BnaC08g32840.1D2	BnaA09g45030.1D2
G50	BnaC08g32850.1D2	BnaA09g45040.1D2
G50	BnaC08g32860.1D2	BnaA09g45050.1D2
G50	BnaC08g32900.1D2	BnaA09g45090.1D2
G50	BnaC08g32910.1D2	BnaA09g45100.1D2
G50	BnaC08g32940.1D2	BnaA09g45120.1D2
G50	BnaC08g32960.1D2	BnaA09g45160.1D2
G50	BnaC08g32980.1D2	BnaA09g45190.1D2
G50	BnaC08g33000.1D2	BnaA09g45210.1D2
G50	BnaC08g33020.1D2	BnaA09g45230.1D2
G50	BnaC08g33030.1D2	BnaA09g45240.1D2
G50	BnaC08g33040.1D2	BnaA09g45250.1D2
G50	BnaC08g33150.1D2	BnaA09g45330.1D2
G50	BnaC08g33280.1D2	BnaA09g45430.1D2

G50	BnaC08g33300.1D2	BnaA09g45450.1D2
G50	BnaC08g33410.1D2	BnaA09g45490.1D2
G50	BnaC08g33430.1D2	BnaA09g45500.1D2
G50	BnaC08g33440.1D2	BnaA09g45520.1D2
G50	BnaC08g33450.1D2	BnaA09g45530.1D2
G50	BnaC08g33470.1D2	BnaA09g45560.1D2
G50	BnaC08g33480.1D2	BnaA09g45570.1D2
G50	BnaC08g33490.1D2	BnaA09g45580.1D2
G50	BnaC08g33500.1D2	BnaA09g45590.1D2
G50	BnaC08g33510.1D2	BnaA09g45600.1D2
G50	BnaC08g33730.1D2	BnaA09g46420.1D2
G50	BnaC08g33760.1D2	BnaA09g46450.1D2
G50	BnaC08g33790.1D2	BnaA09g46480.1D2
G50	BnaC08g33800.1D2	BnaA09g46490.1D2
G50	BnaC08g33810.1D2	BnaA09g46500.1D2
G50	BnaC08g33890.1D2	BnaA09g46090.1D2
G50	BnaC08g33900.1D2	BnaA09g46100.1D2
G50	BnaC08g34000.1D2	BnaA09g46190.1D2
G50	BnaC08g34070.1D2	BnaA09g46260.1D2
G50	BnaC08g34190.1D2	BnaA09g45940.1D2
G50	BnaC08g34220.1D2	BnaA09g45910.1D2
G50	BnaC08g34230.1D2	BnaA09g45900.1D2
G50	BnaC08g34250.1D2	BnaA09g45860.1D2
G50	BnaC08g34270.1D2	BnaA09g45840.1D2
G50	BnaC08g34380.1D2	BnaA09g46700.1D2
G50	BnaC08g34390.1D2	BnaA09g46710.1D2
G50	BnaC08g34400.1D2	BnaA09g46720.1D2
G50	BnaC08g34420.1D2	BnaA09g46740.1D2
G50	BnaC08g34430.1D2	BnaA09g46750.1D2
G50	BnaC08g34440.1D2	BnaA09g46760.1D2
G50	BnaC08g34450.1D2	BnaA09g46770.1D2
G50	BnaC08g34500.1D2	BnaA09g46800.1D2
G50	BnaC08g34500.1D2	BnaA09g46810.1D2
G50	BnaC08g34530.1D2	BnaA09g46840.1D2
G50	BnaC08g34540.1D2	BnaA09g46850.1D2
G50	BnaC08g34570.1D2	BnaA09g46870.1D2
G50	BnaC08g34610.1D2	BnaA09g46890.1D2
G50	BnaC08g34630.1D2	BnaA09g46910.1D2
G50	BnaC08g34650.1D2	BnaA09g46930.1D2
G50	BnaC08g34660.1D2	BnaA09g46940.1D2
G50	BnaC08g34670.1D2	BnaA09g46950.1D2
G50	BnaC08g34700.1D2	BnaA09g45800.1D2
G50	BnaC08g34710.1D2	BnaA09g45780.1D2
G50	BnaC08g34770.1D2	BnaA09g45690.1D2
G50	BnaC08g34830.1D2	BnaA09g45730.1D2
G50	BnaC08g34840.1D2	BnaA09g45740.1D2
G50	BnaC08g34850.1D2	BnaA09g45740.1D2
G50	BnaC08g34860.1D2	BnaA09g45750.1D2
G50	BnaC08g34870.1D2	BnaA09g45760.1D2
G50	BnaC08g34880.1D2	BnaA09g46960.1D2
G50	BnaC08g34920.1D2	BnaA09g47000.1D2

G50	BnaC08g34930.1D2	BnaA09g47010.1D2
G50	BnaC08g35000.1D2	BnaA09g47060.1D2
G50	BnaC08g35010.1D2	BnaA09g47070.1D2
G50	BnaC08g35020.1D2	BnaA09g47080.1D2
G50	BnaC08g35030.1D2	BnaA09g47090.1D2
G50	BnaC08g35110.1D2	BnaA09g47180.1D2
G50	BnaC08g35120.1D2	BnaA09g47190.1D2
G50	BnaC08g35140.1D2	BnaA09g44200.1D2
G50	BnaC08g35230.1D2	BnaA09g46620.1D2
G50	BnaC08g35250.1D2	BnaA09g46600.1D2
G50	BnaC08g35370.1D2	BnaA09g45990.1D2
G50	BnaC08g35490.1D2	BnaA09g47240.1D2
G50	BnaC08g35550.1D2	BnaA09g47290.1D2
G50	BnaC08g35560.1D2	BnaA09g47300.1D2
G50	BnaC08g35600.1D2	BnaA09g47340.1D2
G50	BnaC08g35610.1D2	BnaA09g47350.1D2
G50	BnaC08g35650.1D2	BnaA09g47390.1D2
G50	BnaC08g35670.1D2	BnaA09g47410.1D2
G50	BnaC08g35680.1D2	BnaA09g47420.1D2
G50	BnaC08g35730.1D2	BnaA09g47460.1D2
G50	BnaC08g35780.1D2	BnaA09g47520.1D2
G50	BnaC08g35800.1D2	BnaA09g47540.1D2
G50	BnaC08g35830.1D2	BnaA09g47570.1D2
G50	BnaC08g35840.1D2	BnaA09g47570.1D2
G50	BnaC08g35870.1D2	BnaA09g47590.1D2
G50	BnaC08g35930.1D2	BnaA09g47690.1D2
G50	BnaC08g35960.1D2	BnaA09g47720.1D2
G50	BnaC08g35970.1D2	BnaA09g47730.1D2
G50	BnaC08g36020.1D2	BnaA09g47810.1D2
G50	BnaC08g36040.1D2	BnaA09g47440.1D2
G50	BnaC08g36150.1D2	BnaA09g47890.1D2
G50	BnaC08g36170.1D2	BnaA09g47900.1D2
G50	BnaC08g36200.1D2	BnaA09g47930.1D2
G50	BnaC08g36210.1D2	BnaA09g47940.1D2
G50	BnaC08g36220.1D2	BnaA09g47950.1D2
G50	BnaC08g36280.1D2	BnaA09g48000.1D2
G50	BnaC08g36300.1D2	BnaA09g48020.1D2
G50	BnaC08g36340.1D2	BnaA09g48070.1D2
G50	BnaC08g36350.1D2	BnaA09g48080.1D2
G50	BnaC08g36360.1D2	BnaA09g48090.1D2
G50	BnaC08g36400.1D2	BnaA09g48140.1D2
G50	BnaC08g36420.1D2	BnaA09g48160.1D2
G50	BnaC08g36440.1D2	BnaA09g48180.1D2
G50	BnaC08g36480.1D2	BnaA09g48230.1D2
G50	BnaC08g36490.1D2	BnaA09g48240.1D2
G50	BnaC08g36500.1D2	BnaA09g48260.1D2
G50	BnaC08g36510.1D2	BnaA09g48270.1D2
G50	BnaC08g36520.1D2	BnaA09g48280.1D2
G50	BnaC08g36540.1D2	BnaA09g48300.1D2
G50	BnaC08g36600.1D2	BnaA09g48370.1D2
G50	BnaC08g36620.1D2	BnaA09g48390.1D2

G50	BnaC08g36650.1D2	BnaA09g48420.1D2
G50	BnaC08g36690.1D2	BnaA09g48470.1D2
G50	BnaC08g36800.1D2	BnaA09g48480.1D2
G50	BnaC08g36830.1D2	BnaA09g48500.1D2
G50	BnaC08g36860.1D2	BnaA09g48530.1D2
G50	BnaC08g36920.1D2	BnaA09g48580.1D2
G50	BnaC08g36930.1D2	BnaA09g48590.1D2
G50	BnaC08g36980.1D2	BnaA09g49120.1D2
G50	BnaC08g37000.1D2	BnaA09g48630.1D2
G50	BnaC08g37030.1D2	BnaA09g48640.1D2
G50	BnaC08g37170.1D2	BnaA09g48700.1D2
G50	BnaC08g37220.1D2	BnaA09g48740.1D2
G50	BnaC08g37230.1D2	BnaA09g48750.1D2
G50	BnaC08g37300.1D2	BnaA09g48980.1D2
G50	BnaC08g37330.1D2	BnaA09g48940.1D2
G50	BnaC08g37340.1D2	BnaA09g48930.1D2
G50	BnaC08g37350.1D2	BnaA09g48920.1D2
G50	BnaC08g37360.1D2	BnaA09g48910.1D2
G50	BnaC08g37400.1D2	BnaA09g48870.1D2
G50	BnaC08g37410.1D2	BnaA09g48860.1D2
G50	BnaC08g37450.1D2	BnaA09g49090.1D2
G50	BnaC08g37460.1D2	BnaA09g49090.1D2
G50	BnaC08g37680.1D2	BnaA09g51330.1D2
G50	BnaC08g37720.1D2	BnaA09g51290.1D2
G50	BnaC08g37790.1D2	BnaA09g49350.1D2
G50	BnaC08g37910.1D2	BnaA09g49490.1D2
G50	BnaC08g37920.1D2	BnaA09g49500.1D2
G50	BnaC08g38000.1D2	BnaA09g49570.1D2
G50	BnaC08g38010.1D2	BnaA09g49580.1D2
G50	BnaC08g38060.1D2	BnaA09g49620.1D2
G50	BnaC08g38080.1D2	BnaA09g49640.1D2
G50	BnaC08g38130.1D2	BnaA09g49690.1D2
G50	BnaC08g38140.1D2	BnaA09g49700.1D2
G50	BnaC08g38150.1D2	BnaA09g49710.1D2
G50	BnaC08g38320.1D2	BnaA09g49850.1D2
G50	BnaC08g38330.1D2	BnaA09g49860.1D2
G50	BnaC08g38370.1D2	BnaA09g49900.1D2
G50	BnaC08g38420.1D2	BnaA09g49920.1D2
G50	BnaC08g38580.1D2	BnaA09g50070.1D2
G50	BnaC08g38580.1D2	BnaA09g50080.1D2
G50	BnaC08g38590.1D2	BnaA09g50070.1D2
G50	BnaC08g38590.1D2	BnaA09g50080.1D2
G50	BnaC08g38600.1D2	BnaA09g50090.1D2
G50	BnaC08g38610.1D2	BnaA09g50100.1D2
G50	BnaC08g38640.1D2	BnaA09g50130.1D2
G50	BnaC08g38660.1D2	BnaA09g50150.1D2
G50	BnaC08g38670.1D2	BnaA09g50160.1D2
G50	BnaC08g38700.1D2	BnaA09g52150.1D2
G50	BnaC08g38730.1D2	BnaA09g52130.1D2
G50	BnaC08g38780.1D2	BnaA09g52080.1D2
G50	BnaC08g38790.1D2	BnaA09g52070.1D2

G50	BnaC08g38810.1D2	BnaA09g52050.1D2
G50	BnaC08g38820.1D2	BnaA09g52040.1D2
G50	BnaC08g38850.1D2	BnaA09g52010.1D2
G50	BnaC08g38860.1D2	BnaA09g52000.1D2
G50	BnaC08g39000.1D2	BnaA09g51900.1D2
G50	BnaC08g39090.1D2	BnaA09g51170.1D2
G50	BnaC08g39100.1D2	BnaA09g51160.1D2
G50	BnaC08g39180.1D2	BnaA09g51590.1D2
G50	BnaC08g39240.1D2	BnaA09g51540.1D2
G50	BnaC08g39260.1D2	BnaA09g51520.1D2
G50	BnaC08g39300.1D2	BnaA09g51130.1D2
G50	BnaC08g39320.1D2	BnaA09g51120.1D2
G50	BnaC08g39340.1D2	BnaA09g51110.1D2
G50	BnaC08g39380.1D2	BnaA09g51070.1D2
G50	BnaC08g39400.1D2	BnaA09g51050.1D2
G50	BnaC08g39410.1D2	BnaA09g51040.1D2
G50	BnaC08g39420.1D2	BnaA09g51030.1D2
G50	BnaC08g39440.1D2	BnaA09g51010.1D2
G50	BnaC08g39460.1D2	BnaA09g50990.1D2
G50	BnaC08g39480.1D2	BnaA09g50970.1D2
G50	BnaC08g39510.1D2	BnaA09g50940.1D2
G50	BnaC08g39520.1D2	BnaA09g50930.1D2
G50	BnaC08g39570.1D2	BnaA09g50900.1D2
G50	BnaC08g39580.1D2	BnaA09g50870.1D2
G50	BnaC08g39590.1D2	BnaA09g50870.1D2
G50	BnaC08g39620.1D2	BnaA09g50820.1D2
G50	BnaC08g39680.1D2	BnaA09g50790.1D2
G50	BnaC08g39690.1D2	BnaA09g50780.1D2
G50	BnaC08g39700.1D2	BnaA09g50770.1D2
G50	BnaC08g39710.1D2	BnaA09g50760.1D2
G50	BnaC08g39720.1D2	BnaA09g50750.1D2
G50	BnaC08g39770.1D2	BnaA09g50720.1D2
G50	BnaC08g39820.1D2	BnaA09g50670.1D2
G50	BnaC08g39890.1D2	BnaA09g50630.1D2
G50	BnaC08g39930.1D2	BnaA09g50590.1D2
G50	BnaC08g39970.1D2	BnaA09g50560.1D2
G50	BnaC08g39980.1D2	BnaA09g50550.1D2
G50	BnaC08g40000.1D2	BnaA09g50490.1D2
G50	BnaC08g40030.1D2	BnaA09g50460.1D2
G50	BnaC08g40070.1D2	BnaA09g51620.1D2
G50	BnaC08g40110.1D2	BnaA09g51640.1D2
G50	BnaC08g40130.1D2	BnaA09g51650.1D2
G50	BnaC08g40140.1D2	BnaA09g51650.1D2
G50	BnaC08g40180.1D2	BnaA09g51690.1D2
G50	BnaC08g40280.1D2	BnaA09g50370.1D2
G50	BnaC08g40300.1D2	BnaA09g50360.1D2
G50	BnaC08g40320.1D2	BnaA09g50360.1D2
G50	BnaC08g40330.1D2	BnaA09g50340.1D2
G50	BnaC08g40340.1D2	BnaA09g50330.1D2
G50	BnaC08g40360.1D2	BnaA09g50320.1D2
G50	BnaC08g40370.1D2	BnaA09g50310.1D2

G50	BnaC08g40410.1D2	BnaA09g50260.1D2
G50	BnaC08g40420.1D2	BnaA09g50250.1D2
G50	BnaC08g40500.1D2	BnaA09g51810.1D2
G50	BnaC08g40610.1D2	BnaA09g52680.1D2
G50	BnaC08g40630.1D2	BnaA09g52710.1D2
G50	BnaC08g40640.1D2	BnaA09g52720.1D2
G50	BnaC08g40650.1D2	BnaA09g52730.1D2
G50	BnaC08g40710.1D2	BnaA09g51140.1D2
G50	BnaC08g40720.1D2	BnaA09g52420.1D2
G50	BnaC08g40730.1D2	BnaA09g51170.1D2
G50	BnaC08g40760.1D2	BnaA09g51450.1D2
G50	BnaC08g40820.1D2	BnaA09g50230.1D2
G50	BnaC08g40850.1D2	BnaA09g50180.1D2
G50	BnaC08g40880.1D2	BnaA09g52190.1D2
G50	BnaC08g40950.1D2	BnaA09g52260.1D2
G50	BnaC08g41010.1D2	BnaA09g52260.1D2
G50	BnaC08g41050.1D2	BnaA09g52310.1D2
G50	BnaC08g41120.1D2	BnaA09g52390.1D2
G50	BnaC08g41190.1D2	BnaA09g52460.1D2
G50	BnaC08g41210.1D2	BnaA09g52470.1D2
G50	BnaC08g41240.1D2	BnaA09g52520.1D2
G50	BnaC08g41280.1D2	BnaA09g52560.1D2
G50	BnaC08g41290.1D2	BnaA09g52570.1D2
G50	BnaC08g41300.1D2	BnaA09g52580.1D2
G50	BnaC08g41310.1D2	BnaA09g52590.1D2
G50	BnaC08g41320.1D2	BnaA09g52600.1D2
G50	BnaC08g41370.1D2	BnaA09g52800.1D2
G50	BnaC08g41380.1D2	BnaA09g52810.1D2
G50	BnaC08g41390.1D2	BnaA09g52820.1D2
H165	BnaC08g34400.1D2	BnaA09g46720.1D2
H165	BnaC08g34570.1D2	BnaA09g46870.1D2
H165	BnaC08g34670.1D2	BnaA09g46950.1D2
H165	BnaC08g34710.1D2	BnaA09g45780.1D2
H165	BnaC08g34850.1D2	BnaA09g45740.1D2
H165	BnaC08g35610.1D2	BnaA09g47350.1D2
H165	BnaC08g35650.1D2	BnaA09g47390.1D2
H165	BnaC08g35800.1D2	BnaA09g47540.1D2
H165	BnaC08g35830.1D2	BnaA09g47570.1D2
H165	BnaC08g35930.1D2	BnaA09g47690.1D2
H165	BnaC08g35960.1D2	BnaA09g47720.1D2
H165	BnaC08g36190.1D2	BnaA09g47920.1D2
H165	BnaC08g36210.1D2	BnaA09g47940.1D2
H165	BnaC08g36420.1D2	BnaA09g48160.1D2
H165	BnaC08g36440.1D2	BnaA09g48180.1D2
H165	BnaC08g36520.1D2	BnaA09g48280.1D2
H165	BnaC08g36540.1D2	BnaA09g48300.1D2
H165	BnaC08g36980.1D2	BnaA09g49120.1D2
H165	BnaC08g37000.1D2	BnaA09g48630.1D2
H165	BnaC08g37030.1D2	BnaA09g48640.1D2
H165	BnaC08g37230.1D2	BnaA09g48750.1D2
H165	BnaC08g37240.1D2	BnaA09g48760.1D2

H165	BnaC08g37300.1D2	BnaA09g48980.1D2
H165	BnaC08g37360.1D2	BnaA09g48910.1D2
H165	BnaC08g37400.1D2	BnaA09g48870.1D2
H165	BnaC08g37460.1D2	BnaA09g49090.1D2
H165	BnaC08g37650.1D2	BnaA09g51360.1D2
H165	BnaC08g37840.1D2	BnaA09g49410.1D2
H165	BnaC08g37890.1D2	BnaA09g49470.1D2
H165	BnaC08g38130.1D2	BnaA09g49690.1D2
H165	BnaC08g38140.1D2	BnaA09g49700.1D2
H165	BnaC08g38230.1D2	BnaA09g49780.1D2
H165	BnaC08g38320.1D2	BnaA09g49850.1D2
H165	BnaC08g38430.1D2	BnaA09g49930.1D2
H165	BnaC08g38450.1D2	BnaA09g49940.1D2
H165	BnaC08g39460.1D2	BnaA09g50990.1D2
H165	BnaC08g39540.1D2	BnaA09g50920.1D2
H165	BnaC08g39680.1D2	BnaA09g50790.1D2
H165	BnaC08g39690.1D2	BnaA09g50780.1D2
H165	BnaC08g39700.1D2	BnaA09g50770.1D2
H165	BnaC08g39710.1D2	BnaA09g50760.1D2
H165	BnaC08g39980.1D2	BnaA09g50550.1D2
H165	BnaC08g40030.1D2	BnaA09g50460.1D2
H165	BnaC08g40100.1D2	BnaA09g51640.1D2
H165	BnaC08g40110.1D2	BnaA09g51640.1D2
H165	BnaC08g40130.1D2	BnaA09g51650.1D2
H165	BnaC08g40180.1D2	BnaA09g51690.1D2
H165	BnaC08g40300.1D2	BnaA09g50360.1D2
H165	BnaC08g40330.1D2	BnaA09g50340.1D2
H165	BnaC08g40340.1D2	BnaA09g50330.1D2
H44	BnaC08g18840.1D2	BnaA09g52170.1D2
H44	BnaC08g29180.1D2	BnaA09g41220.1D2
H44	BnaC08g29210.1D2	BnaA09g41250.1D2
H44	BnaC08g29270.1D2	BnaA09g41290.1D2
H44	BnaC08g29350.1D2	BnaA09g41460.1D2
H44	BnaC08g29380.1D2	BnaA09g41480.1D2
H44	BnaC08g29390.1D2	BnaA09g41490.1D2
H44	BnaC08g29480.1D2	BnaA09g41770.1D2
H44	BnaC08g29730.1D2	BnaA09g41960.1D2
H44	BnaC08g29790.1D2	BnaA09g42010.1D2
H44	BnaC08g29850.1D2	BnaA09g42140.1D2
H44	BnaC08g29860.1D2	BnaA09g42150.1D2
H44	BnaC08g29880.1D2	BnaA09g42160.1D2
H44	BnaC08g29890.1D2	BnaA09g42180.1D2
H44	BnaC08g29890.1D2	BnaA09g42330.1D2
H44	BnaC08g29900.1D2	BnaA09g42190.1D2
H44	BnaC08g30020.1D2	BnaA09g42340.1D2
H44	BnaC08g30050.1D2	BnaA09g42500.1D2
H44	BnaC08g30120.1D2	BnaA09g42600.1D2
H44	BnaC08g30290.1D2	BnaA09g42950.1D2
H44	BnaC08g30360.1D2	BnaA09g43020.1D2
H44	BnaC08g30460.1D2	BnaA09g43150.1D2
H44	BnaC08g30540.1D2	BnaA09g43210.1D2

H44	BnaC08g30550.1D2	BnaA09g43220.1D2
H44	BnaC08g30630.1D2	BnaA09g43280.1D2
H44	BnaC08g30810.1D2	BnaA09g43460.1D2
H44	BnaC08g30960.1D2	BnaA09g43610.1D2
H44	BnaC08g30980.1D2	BnaA09g43610.1D2
H44	BnaC08g31000.1D2	BnaA09g43590.1D2
H44	BnaC08g31170.1D2	BnaA09g41450.1D2
H44	BnaC08g31290.1D2	BnaA09g42050.1D2
H44	BnaC08g31340.1D2	BnaA09g42400.1D2
H44	BnaC08g31440.1D2	BnaA09g42080.1D2
H44	BnaC08g31470.1D2	BnaA09g42110.1D2
H44	BnaC08g31510.1D2	BnaA09g43630.1D2
H44	BnaC08g31540.1D2	BnaA09g43670.1D2
H44	BnaC08g31550.1D2	BnaA09g43680.1D2
H44	BnaC08g31620.1D2	BnaA09g43750.1D2
H44	BnaC08g31760.1D2	BnaA09g43830.1D2
H44	BnaC08g31780.1D2	BnaA09g43850.1D2
H44	BnaC08g31820.1D2	BnaA09g43900.1D2
H44	BnaC08g31860.1D2	BnaA09g43940.1D2
H44	BnaC08g31920.1D2	BnaA09g44000.1D2
H44	BnaC08g31930.1D2	BnaA09g44010.1D2
H44	BnaC08g31990.1D2	BnaA09g44070.1D2
H44	BnaC08g32040.1D2	BnaA09g44100.1D2
H44	BnaC08g32060.1D2	BnaA09g44120.1D2
H44	BnaC08g32110.1D2	BnaA09g44150.1D2
H44	BnaC08g32130.1D2	BnaA09g44170.1D2
H44	BnaC08g32140.1D2	BnaA09g44180.1D2
H44	BnaC08g32180.1D2	BnaA09g44240.1D2
H44	BnaC08g32250.1D2	BnaA09g44300.1D2
H44	BnaC08g32310.1D2	BnaA09g44330.1D2
H44	BnaC08g32340.1D2	BnaA09g44370.1D2
H44	BnaC08g32410.1D2	BnaA09g44510.1D2
H44	BnaC08g32450.1D2	BnaA09g44550.1D2
H44	BnaC08g32470.1D2	BnaA09g44570.1D2
H44	BnaC08g32510.1D2	BnaA09g44620.1D2
H44	BnaC08g32550.1D2	BnaA09g44700.1D2
H44	BnaC08g32600.1D2	BnaA09g44790.1D2
H44	BnaC08g32620.1D2	BnaA09g44830.1D2
H44	BnaC08g32630.1D2	BnaA09g44840.1D2
H44	BnaC08g32640.1D2	BnaA09g44840.1D2
H44	BnaC08g32660.1D2	BnaA09g44880.1D2
H44	BnaC08g32670.1D2	BnaA09g44890.1D2
H44	BnaC08g32750.1D2	BnaA09g46540.1D2
H44	BnaC08g32800.1D2	BnaA09g44980.1D2
H44	BnaC08g32810.1D2	BnaA09g45000.1D2
H44	BnaC08g32820.1D2	BnaA09g45010.1D2
H44	BnaC08g32830.1D2	BnaA09g45020.1D2
H44	BnaC08g32940.1D2	BnaA09g45120.1D2
H44	BnaC08g33150.1D2	BnaA09g45330.1D2
H44	BnaC08g33230.1D2	BnaA09g45390.1D2
H44	BnaC08g33280.1D2	BnaA09g45430.1D2

H44	BnaC08g33300.1D2	BnaA09g45450.1D2
H44	BnaC08g33370.1D2	BnaA09g45460.1D2
H44	BnaC08g33440.1D2	BnaA09g45520.1D2
H44	BnaC08g33450.1D2	BnaA09g45530.1D2
H44	BnaC08g35200.1D2	BnaA09g44960.1D2
H44	BnaC08g35350.1D2	BnaA09g42360.1D2
H44	BnaC08g35360.1D2	BnaA09g42380.1D2
H44	BnaC08g38690.1D2	BnaA09g52170.1D2
H44	BnaC08g38870.1D2	BnaA09g51990.1D2
H44	BnaC08g38920.1D2	BnaA09g51940.1D2
H44	BnaC08g38970.1D2	BnaA09g51730.1D2
H44	BnaC08g39000.1D2	BnaA09g51900.1D2
H44	BnaC08g39090.1D2	BnaA09g51170.1D2
H44	BnaC08g39220.1D2	BnaA09g51550.1D2
H44	BnaC08g39240.1D2	BnaA09g51540.1D2
H44	BnaC08g39260.1D2	BnaA09g51520.1D2
H44	BnaC08g39270.1D2	BnaA09g51510.1D2
H44	BnaC08g39320.1D2	BnaA09g51120.1D2
H44	BnaC08g39340.1D2	BnaA09g51110.1D2
H44	BnaC08g39460.1D2	BnaA09g50990.1D2
H44	BnaC08g39510.1D2	BnaA09g50940.1D2
H44	BnaC08g39540.1D2	BnaA09g50920.1D2
H44	BnaC08g39590.1D2	BnaA09g50870.1D2
H44	BnaC08g40500.1D2	BnaA09g51810.1D2
H44	BnaC08g40520.1D2	BnaA09g51820.1D2
H44	BnaC08g40610.1D2	BnaA09g52680.1D2
H44	BnaC08g40630.1D2	BnaA09g52710.1D2
H44	BnaC08g40640.1D2	BnaA09g52720.1D2
H44	BnaC08g40690.1D2	BnaA09g52770.1D2
H44	BnaC08g40710.1D2	BnaA09g51140.1D2
H44	BnaC08g40720.1D2	BnaA09g52420.1D2
H44	BnaC08g40900.1D2	BnaA09g52210.1D2
H44	BnaC08g40910.1D2	BnaA09g52220.1D2
H44	BnaC08g40920.1D2	BnaA09g52230.1D2
H44	BnaC08g40930.1D2	BnaA09g52240.1D2
H44	BnaC08g40950.1D2	BnaA09g52260.1D2
H44	BnaC08g41010.1D2	BnaA09g52260.1D2
H44	BnaC08g41050.1D2	BnaA09g52310.1D2
H44	BnaC08g41080.1D2	BnaA09g52350.1D2
H44	BnaC08g41090.1D2	BnaA09g52360.1D2
H44	BnaC08g41100.1D2	BnaA09g52370.1D2
H44	BnaC08g41210.1D2	BnaA09g52470.1D2
H44	BnaC08g41260.1D2	BnaA09g52540.1D2
H44	BnaC08g41370.1D2	BnaA09g52800.1D2
H44	BnaC08g41380.1D2	BnaA09g52810.1D2

List of HE genes (C09 to A09)

**Accession**	**Lost gene**	**Duplicated HE gene**
Dippes	BnaC09g00040.1D2	BnaA09g01590.1D2
Dippes	BnaC09g00060.1D2	BnaA09g01630.1D2
Dippes	BnaC09g00070.1D2	BnaA09g01640.1D2

Dippes	BnaC09g00080.1D2	BnaA09g01650.1D2
Dippes	BnaC09g00170.1D2	BnaA09g01720.1D2
Dippes	BnaC09g00180.1D2	BnaA09g01740.1D2
Dippes	BnaC09g00190.1D2	BnaA09g01750.1D2
Dippes	BnaC09g00200.1D2	BnaA09g01760.1D2
Dippes	BnaC09g00220.1D2	BnaA09g01780.1D2
Dippes	BnaC09g00270.1D2	BnaA09g01810.1D2
Dippes	BnaC09g00280.1D2	BnaA09g01820.1D2
Dippes	BnaC09g00320.1D2	BnaA09g01850.1D2
Dippes	BnaC09g00330.1D2	BnaA09g01860.1D2
Dippes	BnaC09g00340.1D2	BnaA09g01870.1D2
Dippes	BnaC09g00360.1D2	BnaA09g01890.1D2
Dippes	BnaC09g00370.1D2	BnaA09g01900.1D2
Dippes	BnaC09g00420.1D2	BnaA09g01910.1D2
Dippes	BnaC09g00440.1D2	BnaA09g01920.1D2
Dippes	BnaC09g00460.1D2	BnaA09g01950.1D2
Dippes	BnaC09g00480.1D2	BnaA09g01960.1D2
Dippes	BnaC09g00510.1D2	BnaA09g01990.1D2
Dippes	BnaC09g00520.1D2	BnaA09g02000.1D2
Dippes	BnaC09g00540.1D2	BnaA09g02010.1D2
Dippes	BnaC09g00600.1D2	BnaA09g02090.1D2
Dippes	BnaC09g00800.1D2	BnaA09g02280.1D2
Dippes	BnaC09g00900.1D2	BnaA09g02050.1D2
Dippes	BnaC09g01050.1D2	BnaA09g01550.1D2
Dippes	BnaC09g01080.1D2	BnaA09g01520.1D2
Dippes	BnaC09g01090.1D2	BnaA09g01510.1D2
Dippes	BnaC09g01100.1D2	BnaA09g01500.1D2
Dippes	BnaC09g01130.1D2	BnaA09g01460.1D2
Dippes	BnaC09g01140.1D2	BnaA09g01440.1D2
Dippes	BnaC09g01180.1D2	BnaA09g00940.1D2
Dippes	BnaC09g01190.1D2	BnaA09g00950.1D2
Dippes	BnaC09g01190.1D2	BnaA09g00960.1D2
Dippes	BnaC09g01470.1D2	BnaA09g02740.1D2
Dippes	BnaC09g01480.1D2	BnaA09g02770.1D2
Dippes	BnaC09g01490.1D2	BnaA09g02780.1D2
Dippes	BnaC09g01500.1D2	BnaA09g02790.1D2
Dippes	BnaC09g01660.1D2	BnaA09g02930.1D2
Dippes	BnaC09g01720.1D2	BnaA09g02980.1D2
Dippes	BnaC09g01730.1D2	BnaA09g02990.1D2
Dippes	BnaC09g01740.1D2	BnaA09g03000.1D2
Dippes	BnaC09g01750.1D2	BnaA09g03010.1D2
Dippes	BnaC09g01770.1D2	BnaA09g03020.1D2
Dippes	BnaC09g01780.1D2	BnaA09g03030.1D2
Dippes	BnaC09g01850.1D2	BnaA09g03150.1D2
Dippes	BnaC09g01910.1D2	BnaA09g03210.1D2
Dippes	BnaC09g01930.1D2	BnaA09g03230.1D2
Dippes	BnaC09g02110.1D2	BnaA09g03550.1D2
Dippes	BnaC09g02180.1D2	BnaA09g03610.1D2
Dippes	BnaC09g02200.1D2	BnaA09g03630.1D2
Dippes	BnaC09g02260.1D2	BnaA09g03690.1D2
Dippes	BnaC09g02290.1D2	BnaA09g03730.1D2

Dippes	BnaC09g02310.1D2	BnaA09g03750.1D2
Dippes	BnaC09g02360.1D2	BnaA09g03770.1D2
Dippes	BnaC09g03040.1D2	BnaA09g03300.1D2
Dippes	BnaC09g03130.1D2	BnaA09g03310.1D2
Dippes	BnaC09g03180.1D2	BnaA09g01410.1D2
Dippes	BnaC09g03220.1D2	BnaA09g01370.1D2
Dippes	BnaC09g10800.1D2	BnaA09g11280.1D2
Dippes	BnaC09g11010.1D2	BnaA09g11370.1D2
Dippes	BnaC09g11060.1D2	BnaA09g11390.1D2
EVVIN	BnaC09g00040.1D2	BnaA09g01590.1D2
EVVIN	BnaC09g00060.1D2	BnaA09g01630.1D2
EVVIN	BnaC09g00080.1D2	BnaA09g01650.1D2
EVVIN	BnaC09g00180.1D2	BnaA09g01740.1D2
EVVIN	BnaC09g00190.1D2	BnaA09g01750.1D2
EVVIN	BnaC09g00200.1D2	BnaA09g01760.1D2
EVVIN	BnaC09g00220.1D2	BnaA09g01780.1D2
EVVIN	BnaC09g00280.1D2	BnaA09g01820.1D2
EVVIN	BnaC09g00330.1D2	BnaA09g01860.1D2
EVVIN	BnaC09g00480.1D2	BnaA09g01960.1D2
EVVIN	BnaC09g00520.1D2	BnaA09g02000.1D2
EVVIN	BnaC09g00640.1D2	BnaA09g02130.1D2
EVVIN	BnaC09g00790.1D2	BnaA09g02270.1D2
EVVIN	BnaC09g00800.1D2	BnaA09g02280.1D2
EVVIN	BnaC09g01040.1D2	BnaA09g04210.1D2
EVVIN	BnaC09g01130.1D2	BnaA09g01460.1D2
EVVIN	BnaC09g01140.1D2	BnaA09g01440.1D2
EVVIN	BnaC09g01160.1D2	BnaA09g00920.1D2
EVVIN	BnaC09g01190.1D2	BnaA09g00950.1D2
EVVIN	BnaC09g01190.1D2	BnaA09g00960.1D2
EVVIN	BnaC09g03180.1D2	BnaA09g01410.1D2
GLuesewitzer	BnaC09g01470.1D2	BnaA09g02740.1D2
GLuesewitzer	BnaC09g01480.1D2	BnaA09g02770.1D2
GLuesewitzer	BnaC09g01490.1D2	BnaA09g02780.1D2
GLuesewitzer	BnaC09g01500.1D2	BnaA09g02790.1D2
GLuesewitzer	BnaC09g01660.1D2	BnaA09g02930.1D2
GLuesewitzer	BnaC09g01740.1D2	BnaA09g03000.1D2
GLuesewitzer	BnaC09g01750.1D2	BnaA09g03010.1D2
GLuesewitzer	BnaC09g01770.1D2	BnaA09g03020.1D2
GLuesewitzer	BnaC09g01780.1D2	BnaA09g03030.1D2
GLuesewitzer	BnaC09g01850.1D2	BnaA09g03150.1D2
GLuesewitzer	BnaC09g01910.1D2	BnaA09g03210.1D2
GLuesewitzer	BnaC09g01930.1D2	BnaA09g03230.1D2
GLuesewitzer	BnaC09g02180.1D2	BnaA09g03610.1D2
GLuesewitzer	BnaC09g02200.1D2	BnaA09g03630.1D2
GLuesewitzer	BnaC09g02290.1D2	BnaA09g03730.1D2
GLuesewitzer	BnaC09g02310.1D2	BnaA09g03750.1D2
GLuesewitzer	BnaC09g02360.1D2	BnaA09g03770.1D2
GLuesewitzer	BnaC09g02440.1D2	BnaA09g03890.1D2
GLuesewitzer	BnaC09g02480.1D2	BnaA09g03930.1D2
GLuesewitzer	BnaC09g02580.1D2	BnaA09g04130.1D2
GLuesewitzer	BnaC09g02670.1D2	BnaA09g04300.1D2

GLuesewitzer	BnaC09g02920.1D2	BnaA09g04540.1D2
GLuesewitzer	BnaC09g03090.1D2	BnaA09g03380.1D2
GLuesewitzer	BnaC09g03130.1D2	BnaA09g03310.1D2
GLuesewitzer	BnaC09g03340.1D2	BnaA09g04750.1D2
GLuesewitzer	BnaC09g03730.1D2	BnaA09g05130.1D2
GLuesewitzer	BnaC09g04270.1D2	BnaA09g05630.1D2
GLuesewitzer	BnaC09g08490.1D2	BnaA09g10190.1D2
GLuesewitzer	BnaC09g11010.1D2	BnaA09g11370.1D2
GSchnittkohl	BnaC09g00040.1D2	BnaA09g01590.1D2
GSchnittkohl	BnaC09g00060.1D2	BnaA09g01630.1D2
GSchnittkohl	BnaC09g00070.1D2	BnaA09g01640.1D2
GSchnittkohl	BnaC09g00180.1D2	BnaA09g01740.1D2
GSchnittkohl	BnaC09g00220.1D2	BnaA09g01780.1D2
GSchnittkohl	BnaC09g00250.1D2	BnaA09g01790.1D2
GSchnittkohl	BnaC09g00280.1D2	BnaA09g01820.1D2
GSchnittkohl	BnaC09g00320.1D2	BnaA09g01850.1D2
GSchnittkohl	BnaC09g00330.1D2	BnaA09g01860.1D2
GSchnittkohl	BnaC09g00440.1D2	BnaA09g01920.1D2
GSchnittkohl	BnaC09g00460.1D2	BnaA09g01950.1D2
GSchnittkohl	BnaC09g00480.1D2	BnaA09g01960.1D2
GSchnittkohl	BnaC09g00520.1D2	BnaA09g02000.1D2
GSchnittkohl	BnaC09g00730.1D2	BnaA09g02220.1D2
GSchnittkohl	BnaC09g00800.1D2	BnaA09g02280.1D2
GSchnittkohl	BnaC09g00900.1D2	BnaA09g02050.1D2
GSchnittkohl	BnaC09g00990.1D2	BnaA09g02430.1D2
GSchnittkohl	BnaC09g01010.1D2	BnaA09g04200.1D2
GSchnittkohl	BnaC09g01090.1D2	BnaA09g01510.1D2
GSchnittkohl	BnaC09g01130.1D2	BnaA09g01460.1D2
GSchnittkohl	BnaC09g01190.1D2	BnaA09g00950.1D2
GSchnittkohl	BnaC09g01190.1D2	BnaA09g00960.1D2
GSchnittkohl	BnaC09g01290.1D2	BnaA09g02450.1D2
GSchnittkohl	BnaC09g01440.1D2	BnaA09g02680.1D2
GSchnittkohl	BnaC09g01470.1D2	BnaA09g02740.1D2
GSchnittkohl	BnaC09g01480.1D2	BnaA09g02770.1D2
GSchnittkohl	BnaC09g01490.1D2	BnaA09g02780.1D2
GSchnittkohl	BnaC09g01500.1D2	BnaA09g02790.1D2
GSchnittkohl	BnaC09g01530.1D2	BnaA09g02820.1D2
GSchnittkohl	BnaC09g01540.1D2	BnaA09g02840.1D2
GSchnittkohl	BnaC09g01610.1D2	BnaA09g02910.1D2
GSchnittkohl	BnaC09g01660.1D2	BnaA09g02930.1D2
GSchnittkohl	BnaC09g01720.1D2	BnaA09g02980.1D2
GSchnittkohl	BnaC09g01730.1D2	BnaA09g02990.1D2
GSchnittkohl	BnaC09g01740.1D2	BnaA09g03000.1D2
GSchnittkohl	BnaC09g01750.1D2	BnaA09g03010.1D2
GSchnittkohl	BnaC09g01770.1D2	BnaA09g03020.1D2
GSchnittkohl	BnaC09g01780.1D2	BnaA09g03030.1D2
GSchnittkohl	BnaC09g01830.1D2	BnaA09g03120.1D2
GSchnittkohl	BnaC09g01850.1D2	BnaA09g03150.1D2
GSchnittkohl	BnaC09g01860.1D2	BnaA09g03160.1D2
GSchnittkohl	BnaC09g01880.1D2	BnaA09g03180.1D2
GSchnittkohl	BnaC09g01910.1D2	BnaA09g03210.1D2

GSchnittkohl	BnaC09g01930.1D2	BnaA09g03230.1D2
GSchnittkohl	BnaC09g01960.1D2	BnaA09g03260.1D2
GSchnittkohl	BnaC09g02060.1D2	BnaA09g03450.1D2
GSchnittkohl	BnaC09g02070.1D2	BnaA09g03460.1D2
GSchnittkohl	BnaC09g02180.1D2	BnaA09g03610.1D2
GSchnittkohl	BnaC09g02200.1D2	BnaA09g03630.1D2
GSchnittkohl	BnaC09g02290.1D2	BnaA09g03730.1D2
GSchnittkohl	BnaC09g02360.1D2	BnaA09g03770.1D2
GSchnittkohl	BnaC09g03040.1D2	BnaA09g03300.1D2
GSchnittkohl	BnaC09g03090.1D2	BnaA09g03380.1D2
GSchnittkohl	BnaC09g03130.1D2	BnaA09g03310.1D2
GSchnittkohl	BnaC09g03180.1D2	BnaA09g01410.1D2
Jupiter	BnaC09g00060.1D2	BnaA09g01630.1D2
Jupiter	BnaC09g00070.1D2	BnaA09g01640.1D2
Jupiter	BnaC09g00080.1D2	BnaA09g01650.1D2
Jupiter	BnaC09g00110.1D2	BnaA09g01670.1D2
Jupiter	BnaC09g00120.1D2	BnaA09g01680.1D2
Jupiter	BnaC09g00170.1D2	BnaA09g01720.1D2
Jupiter	BnaC09g00180.1D2	BnaA09g01740.1D2
Jupiter	BnaC09g00190.1D2	BnaA09g01750.1D2
Jupiter	BnaC09g00220.1D2	BnaA09g01780.1D2
Jupiter	BnaC09g00250.1D2	BnaA09g01790.1D2
Jupiter	BnaC09g00270.1D2	BnaA09g01810.1D2
Jupiter	BnaC09g00280.1D2	BnaA09g01820.1D2
Jupiter	BnaC09g00320.1D2	BnaA09g01850.1D2
Jupiter	BnaC09g00440.1D2	BnaA09g01920.1D2
Jupiter	BnaC09g00480.1D2	BnaA09g01960.1D2
Jupiter	BnaC09g00510.1D2	BnaA09g01990.1D2
Jupiter	BnaC09g00520.1D2	BnaA09g02000.1D2
Jupiter	BnaC09g00540.1D2	BnaA09g02010.1D2
Jupiter	BnaC09g00810.1D2	BnaA09g02290.1D2
Jupiter	BnaC09g00900.1D2	BnaA09g02050.1D2
Jupiter	BnaC09g00930.1D2	BnaA09g02370.1D2
Jupiter	BnaC09g01010.1D2	BnaA09g04190.1D2
Jupiter	BnaC09g01010.1D2	BnaA09g04200.1D2
Jupiter	BnaC09g01030.1D2	BnaA09g04240.1D2
Jupiter	BnaC09g01040.1D2	BnaA09g04210.1D2
Jupiter	BnaC09g01050.1D2	BnaA09g01550.1D2
Jupiter	BnaC09g01140.1D2	BnaA09g01440.1D2
Jupiter	BnaC09g01190.1D2	BnaA09g00950.1D2
Jupiter	BnaC09g01190.1D2	BnaA09g00960.1D2
Jupiter	BnaC09g01290.1D2	BnaA09g02450.1D2
Jupiter	BnaC09g01310.1D2	BnaA09g02480.1D2
Jupiter	BnaC09g01340.1D2	BnaA09g02510.1D2
Jupiter	BnaC09g01360.1D2	BnaA09g02550.1D2
Jupiter	BnaC09g01380.1D2	BnaA09g02580.1D2
Jupiter	BnaC09g01440.1D2	BnaA09g02680.1D2
Jupiter	BnaC09g01470.1D2	BnaA09g02740.1D2
Jupiter	BnaC09g01490.1D2	BnaA09g02780.1D2
Jupiter	BnaC09g01500.1D2	BnaA09g02790.1D2
Jupiter	BnaC09g01530.1D2	BnaA09g02820.1D2

Jupiter	BnaC09g01540.1D2	BnaA09g02840.1D2
Jupiter	BnaC09g01610.1D2	BnaA09g02910.1D2
Jupiter	BnaC09g01660.1D2	BnaA09g02930.1D2
Jupiter	BnaC09g01720.1D2	BnaA09g02980.1D2
Jupiter	BnaC09g01740.1D2	BnaA09g03000.1D2
Jupiter	BnaC09g01770.1D2	BnaA09g03020.1D2
Jupiter	BnaC09g01780.1D2	BnaA09g03030.1D2
Jupiter	BnaC09g01830.1D2	BnaA09g03120.1D2
Jupiter	BnaC09g01910.1D2	BnaA09g03210.1D2
Jupiter	BnaC09g01930.1D2	BnaA09g03230.1D2
Jupiter	BnaC09g03090.1D2	BnaA09g03380.1D2
Jupiter	BnaC09g03130.1D2	BnaA09g03310.1D2
Jupiter	BnaC09g03190.1D2	BnaA09g01400.1D2
Jupiter	BnaC09g03210.1D2	BnaA09g01370.1D2
Major	BnaC09g00040.1D2	BnaA09g01590.1D2
Major	BnaC09g00060.1D2	BnaA09g01630.1D2
Major	BnaC09g00070.1D2	BnaA09g01640.1D2
Major	BnaC09g00080.1D2	BnaA09g01650.1D2
Major	BnaC09g00180.1D2	BnaA09g01740.1D2
Major	BnaC09g00190.1D2	BnaA09g01750.1D2
Major	BnaC09g00200.1D2	BnaA09g01760.1D2
Major	BnaC09g00220.1D2	BnaA09g01780.1D2
Major	BnaC09g00250.1D2	BnaA09g01790.1D2
Major	BnaC09g00280.1D2	BnaA09g01820.1D2
Major	BnaC09g00320.1D2	BnaA09g01850.1D2
Major	BnaC09g00460.1D2	BnaA09g01950.1D2
Major	BnaC09g00480.1D2	BnaA09g01960.1D2
Major	BnaC09g00520.1D2	BnaA09g02000.1D2
Major	BnaC09g00800.1D2	BnaA09g02280.1D2
Major	BnaC09g00810.1D2	BnaA09g02290.1D2
Major	BnaC09g00860.1D2	BnaA09g02330.1D2
Major	BnaC09g01190.1D2	BnaA09g00950.1D2
Major	BnaC09g01190.1D2	BnaA09g00960.1D2
Major	BnaC09g01290.1D2	BnaA09g02450.1D2
Major	BnaC09g01310.1D2	BnaA09g02480.1D2
Major	BnaC09g01440.1D2	BnaA09g02680.1D2
Major	BnaC09g01480.1D2	BnaA09g02770.1D2
Major	BnaC09g01490.1D2	BnaA09g02780.1D2
Major	BnaC09g01500.1D2	BnaA09g02790.1D2
Major	BnaC09g01530.1D2	BnaA09g02820.1D2
Major	BnaC09g01540.1D2	BnaA09g02840.1D2
Major	BnaC09g01610.1D2	BnaA09g02910.1D2
Major	BnaC09g01660.1D2	BnaA09g02930.1D2
Major	BnaC09g01740.1D2	BnaA09g03000.1D2
Major	BnaC09g01750.1D2	BnaA09g03010.1D2
Major	BnaC09g01770.1D2	BnaA09g03020.1D2
Major	BnaC09g01780.1D2	BnaA09g03030.1D2
Major	BnaC09g01790.1D2	BnaA09g03090.1D2
Major	BnaC09g01830.1D2	BnaA09g03120.1D2
Major	BnaC09g01850.1D2	BnaA09g03150.1D2
Major	BnaC09g01860.1D2	BnaA09g03160.1D2

Major	BnaC09g01880.1D2	BnaA09g03180.1D2
Major	BnaC09g01910.1D2	BnaA09g03210.1D2
Major	BnaC09g02070.1D2	BnaA09g03460.1D2
Major	BnaC09g02110.1D2	BnaA09g03550.1D2
Major	BnaC09g02180.1D2	BnaA09g03610.1D2
Major	BnaC09g02200.1D2	BnaA09g03630.1D2
Major	BnaC09g02290.1D2	BnaA09g03730.1D2
Major	BnaC09g02310.1D2	BnaA09g03750.1D2
Major	BnaC09g02360.1D2	BnaA09g03770.1D2
Major	BnaC09g02480.1D2	BnaA09g03930.1D2
Major	BnaC09g02520.1D2	BnaA09g04070.1D2
Major	BnaC09g02540.1D2	BnaA09g04080.1D2
Major	BnaC09g02580.1D2	BnaA09g04130.1D2
Major	BnaC09g02600.1D2	BnaA09g00870.1D2
Major	BnaC09g02610.1D2	BnaA09g00860.1D2
Major	BnaC09g02650.1D2	BnaA09g04280.1D2
Major	BnaC09g02670.1D2	BnaA09g04300.1D2
Major	BnaC09g02680.1D2	BnaA09g04320.1D2
Major	BnaC09g02910.1D2	BnaA09g04530.1D2
Major	BnaC09g02920.1D2	BnaA09g04540.1D2
Major	BnaC09g02960.1D2	BnaA09g04590.1D2
Major	BnaC09g02970.1D2	BnaA09g04600.1D2
Major	BnaC09g02980.1D2	BnaA09g04610.1D2
Major	BnaC09g03000.1D2	BnaA09g04640.1D2
Major	BnaC09g03040.1D2	BnaA09g03300.1D2
Major	BnaC09g03170.1D2	BnaA09g03350.1D2
Major	BnaC09g03180.1D2	BnaA09g01410.1D2
Sensation	BnaC09g02520.1D2	BnaA09g04070.1D2
Sensation	BnaC09g02540.1D2	BnaA09g04080.1D2
Sensation	BnaC09g02580.1D2	BnaA09g04130.1D2
Sensation	BnaC09g02600.1D2	BnaA09g00870.1D2
Sensation	BnaC09g02610.1D2	BnaA09g00860.1D2
Sensation	BnaC09g02650.1D2	BnaA09g04280.1D2
Sensation	BnaC09g02670.1D2	BnaA09g04300.1D2
Sensation	BnaC09g02680.1D2	BnaA09g04320.1D2
Sensation	BnaC09g02690.1D2	BnaA09g04330.1D2
Sensation	BnaC09g02760.1D2	BnaA09g04380.1D2
Sensation	BnaC09g02890.1D2	BnaA09g04520.1D2
Sensation	BnaC09g02900.1D2	BnaA09g05730.1D2
Sensation	BnaC09g02910.1D2	BnaA09g04530.1D2
Sensation	BnaC09g02920.1D2	BnaA09g04540.1D2
Sensation	BnaC09g03000.1D2	BnaA09g04640.1D2
Sensation	BnaC09g03180.1D2	BnaA09g01410.1D2
Sensation	BnaC09g03250.1D2	BnaA09g01340.1D2
Sensation	BnaC09g03500.1D2	BnaA09g04890.1D2
Sensation	BnaC09g03690.1D2	BnaA09g05100.1D2
Sensation	BnaC09g03710.1D2	BnaA09g05120.1D2
Sensation	BnaC09g03730.1D2	BnaA09g05130.1D2
Sensation	BnaC09g03750.1D2	BnaA09g05140.1D2
Sensation	BnaC09g03960.1D2	BnaA09g05380.1D2
Sensation	BnaC09g04140.1D2	BnaA09g05510.1D2

Sensation	BnaC09g04160.1D2	BnaA09g05540.1D2
Sensation	BnaC09g04270.1D2	BnaA09g05630.1D2
Sensation	BnaC09g04280.1D2	BnaA09g05630.1D2
Sensation	BnaC09g04340.1D2	BnaA09g05680.1D2
Sensation	BnaC09g04590.1D2	BnaA09g05950.1D2
Sensation	BnaC09g04870.1D2	BnaA09g06260.1D2
Sensation	BnaC09g05210.1D2	BnaA09g06570.1D2
Sensation	BnaC09g05290.1D2	BnaA09g06650.1D2
Sensation	BnaC09g05410.1D2	BnaA09g06800.1D2
Sensation	BnaC09g05430.1D2	BnaA09g06810.1D2
Sensation	BnaC09g05470.1D2	BnaA09g06850.1D2
Sensation	BnaC09g05600.1D2	BnaA09g06750.1D2
Sensation	BnaC09g05630.1D2	BnaA09g06170.1D2
Sensation	BnaC09g05730.1D2	BnaA09g04700.1D2
Sensation	BnaC09g05760.1D2	BnaA09g06900.1D2
Sensation	BnaC09g05760.1D2	BnaA09g06910.1D2
Sensation	BnaC09g05840.1D2	BnaA09g06980.1D2
Sensation	BnaC09g05960.1D2	BnaA09g07070.1D2
Sensation	BnaC09g06000.1D2	BnaA09g07150.1D2
Sensation	BnaC09g06020.1D2	BnaA09g07160.1D2
Sensation	BnaC09g06240.1D2	BnaA09g07230.1D2
Sensation	BnaC09g06250.1D2	BnaA09g07250.1D2
Sensation	BnaC09g06260.1D2	BnaA09g07260.1D2
Sensation	BnaC09g06520.1D2	BnaA09g07490.1D2
Sensation	BnaC09g06540.1D2	BnaA09g07500.1D2
Sensation	BnaC09g06590.1D2	BnaA09g07540.1D2
Sensation	BnaC09g06780.1D2	BnaA09g07700.1D2
Sensation	BnaC09g06790.1D2	BnaA09g07710.1D2
Sensation	BnaC09g06830.1D2	BnaA09g07760.1D2
Sensation	BnaC09g07050.1D2	BnaA09g07910.1D2
Sensation	BnaC09g07090.1D2	BnaA09g07950.1D2
Sensation	BnaC09g07130.1D2	BnaA09g07980.1D2
Sensation	BnaC09g07140.1D2	BnaA09g07990.1D2
Sensation	BnaC09g07150.1D2	BnaA09g08000.1D2
Sensation	BnaC09g07170.1D2	BnaA09g08010.1D2
Sensation	BnaC09g07350.1D2	BnaA09g08170.1D2
Sensation	BnaC09g07360.1D2	BnaA09g08180.1D2
Sensation	BnaC09g07430.1D2	BnaA09g08250.1D2
Sensation	BnaC09g07470.1D2	BnaA09g08790.1D2
Sensation	BnaC09g07640.1D2	BnaA09g08310.1D2
Sensation	BnaC09g07700.1D2	BnaA09g08350.1D2
Sensation	BnaC09g07710.1D2	BnaA09g08360.1D2
Sensation	BnaC09g07720.1D2	BnaA09g08370.1D2
Sensation	BnaC09g08510.1D2	BnaA09g07790.1D2
Sensation	BnaC09g09350.1D2	BnaA09g07100.1D2
Sensation	BnaC09g39700.1D2	BnaA09g08860.1D2
Wotan	BnaC09g00040.1D2	BnaA09g01590.1D2
Wotan	BnaC09g00060.1D2	BnaA09g01630.1D2
Wotan	BnaC09g00170.1D2	BnaA09g01720.1D2
Wotan	BnaC09g00180.1D2	BnaA09g01740.1D2
Wotan	BnaC09g00190.1D2	BnaA09g01750.1D2

Wotan	BnaC09g00200.1D2	BnaA09g01760.1D2
Wotan	BnaC09g00220.1D2	BnaA09g01780.1D2
Wotan	BnaC09g00250.1D2	BnaA09g01790.1D2
Wotan	BnaC09g00270.1D2	BnaA09g01810.1D2
Wotan	BnaC09g00280.1D2	BnaA09g01820.1D2
Wotan	BnaC09g00320.1D2	BnaA09g01850.1D2
Wotan	BnaC09g00440.1D2	BnaA09g01920.1D2
Wotan	BnaC09g00460.1D2	BnaA09g01950.1D2
Wotan	BnaC09g00510.1D2	BnaA09g01990.1D2
Wotan	BnaC09g00520.1D2	BnaA09g02000.1D2
Wotan	BnaC09g00540.1D2	BnaA09g02010.1D2
Wotan	BnaC09g00620.1D2	BnaA09g02110.1D2
Wotan	BnaC09g00800.1D2	BnaA09g02280.1D2
Wotan	BnaC09g01010.1D2	BnaA09g04200.1D2
Wotan	BnaC09g01030.1D2	BnaA09g04240.1D2
Wotan	BnaC09g01040.1D2	BnaA09g04210.1D2
Wotan	BnaC09g01130.1D2	BnaA09g01460.1D2
Wotan	BnaC09g01140.1D2	BnaA09g01440.1D2
Wotan	BnaC09g01180.1D2	BnaA09g00940.1D2
Wotan	BnaC09g01290.1D2	BnaA09g02450.1D2
Wotan	BnaC09g01310.1D2	BnaA09g02480.1D2
Wotan	BnaC09g01340.1D2	BnaA09g02510.1D2
Wotan	BnaC09g01470.1D2	BnaA09g02740.1D2
Wotan	BnaC09g01480.1D2	BnaA09g02770.1D2
Wotan	BnaC09g01490.1D2	BnaA09g02780.1D2
Wotan	BnaC09g01500.1D2	BnaA09g02790.1D2
Wotan	BnaC09g01530.1D2	BnaA09g02820.1D2
Wotan	BnaC09g01540.1D2	BnaA09g02840.1D2
Wotan	BnaC09g01660.1D2	BnaA09g02930.1D2
Wotan	BnaC09g01730.1D2	BnaA09g02990.1D2
Wotan	BnaC09g01740.1D2	BnaA09g03000.1D2
Wotan	BnaC09g01750.1D2	BnaA09g03010.1D2
Wotan	BnaC09g01770.1D2	BnaA09g03020.1D2
Wotan	BnaC09g01780.1D2	BnaA09g03030.1D2
Wotan	BnaC09g01850.1D2	BnaA09g03150.1D2
Wotan	BnaC09g01910.1D2	BnaA09g03210.1D2
Wotan	BnaC09g01930.1D2	BnaA09g03230.1D2
Wotan	BnaC09g02030.1D2	BnaA09g03420.1D2
Wotan	BnaC09g02070.1D2	BnaA09g03460.1D2
Wotan	BnaC09g02110.1D2	BnaA09g03550.1D2
Wotan	BnaC09g02180.1D2	BnaA09g03610.1D2
Wotan	BnaC09g02200.1D2	BnaA09g03630.1D2
Wotan	BnaC09g02290.1D2	BnaA09g03730.1D2
Wotan	BnaC09g02310.1D2	BnaA09g03750.1D2
Wotan	BnaC09g02360.1D2	BnaA09g03770.1D2
Wotan	BnaC09g03040.1D2	BnaA09g03300.1D2
Wotan	BnaC09g03090.1D2	BnaA09g03380.1D2
Wotan	BnaC09g03130.1D2	BnaA09g03310.1D2
Wotan	BnaC09g03170.1D2	BnaA09g03350.1D2
Wotan	BnaC09g03180.1D2	BnaA09g01410.1D2
Wotan	BnaC09g10780.1D2	BnaA09g11300.1D2

Wotan	BnaC09g10800.1D2	BnaA09g11280.1D2
Wotan	BnaC09g11010.1D2	BnaA09g11370.1D2
Wotan	BnaC09g11040.1D2	BnaA09g11380.1D2
Wotan	BnaC09g11060.1D2	BnaA09g11390.1D2
Wotan	BnaC09g11200.1D2	BnaA09g11510.1D2
H149	BnaC09g02690.1D2	BnaA09g04330.1D2
H149	BnaC09g03340.1D2	BnaA09g04750.1D2
H149	BnaC09g03380.1D2	BnaA09g04770.1D2
H149	BnaC09g03410.1D2	BnaA09g04790.1D2
H149	BnaC09g03500.1D2	BnaA09g04890.1D2
H149	BnaC09g03710.1D2	BnaA09g05120.1D2
H149	BnaC09g03730.1D2	BnaA09g05130.1D2
H149	BnaC09g03750.1D2	BnaA09g05140.1D2
H149	BnaC09g03760.1D2	BnaA09g05150.1D2
H149	BnaC09g03940.1D2	BnaA09g05350.1D2
H149	BnaC09g04160.1D2	BnaA09g05540.1D2
H149	BnaC09g04650.1D2	BnaA09g06030.1D2
H149	BnaC09g04660.1D2	BnaA09g06040.1D2
H149	BnaC09g04730.1D2	BnaA09g06100.1D2
H149	BnaC09g04740.1D2	BnaA09g06110.1D2
H149	BnaC09g04890.1D2	BnaA09g06280.1D2
H149	BnaC09g04900.1D2	BnaA09g06290.1D2
H149	BnaC09g05030.1D2	BnaA09g06350.1D2
H149	BnaC09g05080.1D2	BnaA09g06450.1D2
H149	BnaC09g05290.1D2	BnaA09g06650.1D2
H149	BnaC09g05360.1D2	BnaA09g06700.1D2
H149	BnaC09g05430.1D2	BnaA09g06810.1D2
H149	BnaC09g05470.1D2	BnaA09g06850.1D2
H44	BnaC09g00190.1D2	BnaA09g01750.1D2
H44	BnaC09g00200.1D2	BnaA09g01760.1D2
H44	BnaC09g00220.1D2	BnaA09g01780.1D2
H44	BnaC09g00250.1D2	BnaA09g01790.1D2
H44	BnaC09g00360.1D2	BnaA09g01890.1D2
H44	BnaC09g00440.1D2	BnaA09g01920.1D2
H44	BnaC09g00480.1D2	BnaA09g01960.1D2
H44	BnaC09g00520.1D2	BnaA09g02000.1D2
H44	BnaC09g00600.1D2	BnaA09g02090.1D2
H44	BnaC09g00640.1D2	BnaA09g02130.1D2
H44	BnaC09g00800.1D2	BnaA09g02280.1D2
H44	BnaC09g00810.1D2	BnaA09g02290.1D2
H44	BnaC09g00820.1D2	BnaA09g02300.1D2
H44	BnaC09g00900.1D2	BnaA09g02050.1D2
H44	BnaC09g01160.1D2	BnaA09g00920.1D2
H44	BnaC09g01190.1D2	BnaA09g00950.1D2
H44	BnaC09g01190.1D2	BnaA09g00960.1D2
H44	BnaC09g01210.1D2	BnaA09g00960.1D2
H44	BnaC09g01470.1D2	BnaA09g02740.1D2
H44	BnaC09g01490.1D2	BnaA09g02780.1D2
H44	BnaC09g01740.1D2	BnaA09g03000.1D2
H44	BnaC09g01830.1D2	BnaA09g03120.1D2
H44	BnaC09g01910.1D2	BnaA09g03210.1D2

H44	BnaC09g01930.1D2	BnaA09g03230.1D2
H44	BnaC09g02070.1D2	BnaA09g03460.1D2
H44	BnaC09g02180.1D2	BnaA09g03610.1D2
H44	BnaC09g02240.1D2	BnaA09g03670.1D2
H44	BnaC09g02260.1D2	BnaA09g03690.1D2
H44	BnaC09g02270.1D2	BnaA09g03700.1D2
H44	BnaC09g02360.1D2	BnaA09g03770.1D2
H44	BnaC09g02440.1D2	BnaA09g03890.1D2
H44	BnaC09g02670.1D2	BnaA09g04300.1D2
H44	BnaC09g02690.1D2	BnaA09g04330.1D2
H44	BnaC09g02890.1D2	BnaA09g04520.1D2
H44	BnaC09g02950.1D2	BnaA09g04570.1D2
H44	BnaC09g03040.1D2	BnaA09g03300.1D2
H44	BnaC09g03120.1D2	BnaA09g03360.1D2
H44	BnaC09g03130.1D2	BnaA09g03310.1D2
H44	BnaC09g03170.1D2	BnaA09g03350.1D2
H44	BnaC09g03180.1D2	BnaA09g01410.1D2
H44	BnaC09g03340.1D2	BnaA09g04750.1D2
H44	BnaC09g03380.1D2	BnaA09g04770.1D2
H44	BnaC09g03410.1D2	BnaA09g04790.1D2
H44	BnaC09g03500.1D2	BnaA09g04890.1D2
H44	BnaC09g03540.1D2	BnaA09g04920.1D2
H44	BnaC09g03690.1D2	BnaA09g05100.1D2
H44	BnaC09g03710.1D2	BnaA09g05120.1D2
H44	BnaC09g03750.1D2	BnaA09g05140.1D2
H44	BnaC09g03760.1D2	BnaA09g05150.1D2
H44	BnaC09g03850.1D2	BnaA09g05280.1D2
H44	BnaC09g03960.1D2	BnaA09g05380.1D2
H44	BnaC09g03980.1D2	BnaA09g05410.1D2
H44	BnaC09g04010.1D2	BnaA09g05440.1D2
H44	BnaC09g04080.1D2	BnaA09g05460.1D2
H44	BnaC09g04140.1D2	BnaA09g05510.1D2
H44	BnaC09g04160.1D2	BnaA09g05540.1D2
H44	BnaC09g04200.1D2	BnaA09g05580.1D2
H44	BnaC09g04270.1D2	BnaA09g05630.1D2
H44	BnaC09g04660.1D2	BnaA09g06040.1D2
H44	BnaC09g04720.1D2	BnaA09g06090.1D2
H44	BnaC09g04730.1D2	BnaA09g06100.1D2
H44	BnaC09g04890.1D2	BnaA09g06280.1D2
H44	BnaC09g04900.1D2	BnaA09g06290.1D2
H44	BnaC09g05210.1D2	BnaA09g06570.1D2
H44	BnaC09g05230.1D2	BnaA09g06590.1D2
H44	BnaC09g05260.1D2	BnaA09g06630.1D2
H44	BnaC09g05360.1D2	BnaA09g06700.1D2
H44	BnaC09g05390.1D2	BnaA09g06720.1D2
H44	BnaC09g05430.1D2	BnaA09g06810.1D2
H44	BnaC09g05470.1D2	BnaA09g06850.1D2
H44	BnaC09g05600.1D2	BnaA09g06750.1D2
H44	BnaC09g05780.1D2	BnaA09g06930.1D2
H44	BnaC09g05870.1D2	BnaA09g06990.1D2
H44	BnaC09g05930.1D2	BnaA09g07050.1D2

H44	BnaC09g06250.1D2	BnaA09g07250.1D2
H44	BnaC09g06260.1D2	BnaA09g07260.1D2
H44	BnaC09g06590.1D2	BnaA09g07540.1D2
H44	BnaC09g06720.1D2	BnaA09g07650.1D2
H44	BnaC09g06760.1D2	BnaA09g07690.1D2
H44	BnaC09g06780.1D2	BnaA09g07700.1D2
H44	BnaC09g06790.1D2	BnaA09g07710.1D2
H44	BnaC09g06830.1D2	BnaA09g07760.1D2
H44	BnaC09g06890.1D2	BnaA09g08900.1D2
H44	BnaC09g06980.1D2	BnaA09g07840.1D2
H44	BnaC09g06990.1D2	BnaA09g07850.1D2
H44	BnaC09g07310.1D2	BnaA09g08120.1D2
H44	BnaC09g07330.1D2	BnaA09g08140.1D2
H44	BnaC09g07340.1D2	BnaA09g08150.1D2
H44	BnaC09g07350.1D2	BnaA09g08170.1D2
H44	BnaC09g07430.1D2	BnaA09g08250.1D2
H44	BnaC09g07590.1D2	BnaA09g08840.1D2
H44	BnaC09g07620.1D2	BnaA09g08290.1D2
H44	BnaC09g07890.1D2	BnaA09g08490.1D2
H44	BnaC09g07910.1D2	BnaA09g08500.1D2
H44	BnaC09g08040.1D2	BnaA09g08650.1D2
H44	BnaC09g08050.1D2	BnaA09g08660.1D2
H44	BnaC09g08170.1D2	BnaA09g09020.1D2
H44	BnaC09g08210.1D2	BnaA09g09050.1D2
H44	BnaC09g08510.1D2	BnaA09g07790.1D2
Resyn_Go_S4	BnaC09g00040.1D2	BnaA09g01590.1D2
Resyn_Go_S4	BnaC09g00060.1D2	BnaA09g01630.1D2
Resyn_Go_S4	BnaC09g00070.1D2	BnaA09g01640.1D2
Resyn_Go_S4	BnaC09g00080.1D2	BnaA09g01650.1D2
Resyn_Go_S4	BnaC09g00110.1D2	BnaA09g01670.1D2
Resyn_Go_S4	BnaC09g00120.1D2	BnaA09g01680.1D2
Resyn_Go_S4	BnaC09g00180.1D2	BnaA09g01740.1D2
Resyn_Go_S4	BnaC09g00190.1D2	BnaA09g01750.1D2
Resyn_Go_S4	BnaC09g00200.1D2	BnaA09g01760.1D2
Resyn_Go_S4	BnaC09g00220.1D2	BnaA09g01780.1D2
Resyn_Go_S4	BnaC09g00250.1D2	BnaA09g01790.1D2
Resyn_Go_S4	BnaC09g00270.1D2	BnaA09g01810.1D2
Resyn_Go_S4	BnaC09g00320.1D2	BnaA09g01850.1D2
Resyn_Go_S4	BnaC09g00440.1D2	BnaA09g01920.1D2
Resyn_Go_S4	BnaC09g00460.1D2	BnaA09g01950.1D2
Resyn_Go_S4	BnaC09g00480.1D2	BnaA09g01960.1D2
Resyn_Go_S4	BnaC09g00520.1D2	BnaA09g02000.1D2
Resyn_Go_S4	BnaC09g00790.1D2	BnaA09g02270.1D2
Resyn_Go_S4	BnaC09g00800.1D2	BnaA09g02280.1D2
Resyn_Go_S4	BnaC09g00810.1D2	BnaA09g02290.1D2
Resyn_Go_S4	BnaC09g00990.1D2	BnaA09g02430.1D2
Resyn_Go_S4	BnaC09g01140.1D2	BnaA09g01440.1D2
Resyn_Go_S4	BnaC09g01180.1D2	BnaA09g00940.1D2
Resyn_Go_S4	BnaC09g01190.1D2	BnaA09g00950.1D2
Resyn_Go_S4	BnaC09g01190.1D2	BnaA09g00960.1D2
Resyn_Go_S4	BnaC09g01210.1D2	BnaA09g00960.1D2

Resyn_Go_S4	BnaC09g01470.1D2	BnaA09g02740.1D2
Resyn_Go_S4	BnaC09g01480.1D2	BnaA09g02770.1D2
Resyn_Go_S4	BnaC09g01490.1D2	BnaA09g02780.1D2
Resyn_Go_S4	BnaC09g01500.1D2	BnaA09g02790.1D2
Resyn_Go_S4	BnaC09g01660.1D2	BnaA09g02930.1D2
Resyn_Go_S4	BnaC09g01670.1D2	BnaA09g02940.1D2
Resyn_Go_S4	BnaC09g01720.1D2	BnaA09g02980.1D2
Resyn_Go_S4	BnaC09g01730.1D2	BnaA09g02990.1D2
Resyn_Go_S4	BnaC09g01740.1D2	BnaA09g03000.1D2
Resyn_Go_S4	BnaC09g01750.1D2	BnaA09g03010.1D2
Resyn_Go_S4	BnaC09g01780.1D2	BnaA09g03030.1D2
Resyn_Go_S4	BnaC09g01790.1D2	BnaA09g03090.1D2
Resyn_Go_S4	BnaC09g01850.1D2	BnaA09g03150.1D2
Resyn_Go_S4	BnaC09g01860.1D2	BnaA09g03160.1D2
Resyn_Go_S4	BnaC09g01910.1D2	BnaA09g03210.1D2
Resyn_Go_S4	BnaC09g01920.1D2	BnaA09g03220.1D2
Resyn_Go_S4	BnaC09g01930.1D2	BnaA09g03230.1D2
Resyn_Go_S4	BnaC09g02010.1D2	BnaA09g03400.1D2
Resyn_Go_S4	BnaC09g02030.1D2	BnaA09g03420.1D2
Resyn_Go_S4	BnaC09g02200.1D2	BnaA09g03630.1D2
Resyn_Go_S4	BnaC09g02290.1D2	BnaA09g03730.1D2
Resyn_Go_S4	BnaC09g02310.1D2	BnaA09g03750.1D2
Resyn_Go_S4	BnaC09g02360.1D2	BnaA09g03770.1D2
Resyn_Go_S4	BnaC09g02480.1D2	BnaA09g03930.1D2
Resyn_Go_S4	BnaC09g02580.1D2	BnaA09g04130.1D2
Resyn_Go_S4	BnaC09g02630.1D2	BnaA09g00840.1D2
Resyn_Go_S4	BnaC09g02650.1D2	BnaA09g04280.1D2
Resyn_Go_S4	BnaC09g02920.1D2	BnaA09g04540.1D2
Resyn_Go_S4	BnaC09g02960.1D2	BnaA09g04590.1D2
Resyn_Go_S4	BnaC09g03000.1D2	BnaA09g04640.1D2
Resyn_Go_S4	BnaC09g03040.1D2	BnaA09g03300.1D2
Resyn_Go_S4	BnaC09g03090.1D2	BnaA09g03380.1D2
Resyn_Go_S4	BnaC09g03130.1D2	BnaA09g03310.1D2
Resyn_Go_S4	BnaC09g03170.1D2	BnaA09g03350.1D2
Resyn_Go_S4	BnaC09g03180.1D2	BnaA09g01410.1D2
Resyn_Go_S4	BnaC09g03220.1D2	BnaA09g01370.1D2
Resyn_Go_S4	BnaC09g03480.1D2	BnaA09g04870.1D2
Resyn_Go_S4	BnaC09g03690.1D2	BnaA09g05100.1D2
Resyn_Go_S4	BnaC09g03710.1D2	BnaA09g05120.1D2
Resyn_Go_S4	BnaC09g03730.1D2	BnaA09g05130.1D2
Resyn_Go_S4	BnaC09g03960.1D2	BnaA09g05380.1D2
Resyn_Go_S4	BnaC09g04270.1D2	BnaA09g05630.1D2
Resyn_Go_S4	BnaC09g04280.1D2	BnaA09g05630.1D2
Resyn_Go_S4	BnaC09g04640.1D2	BnaA09g06020.1D2
Resyn_Go_S4	BnaC09g04650.1D2	BnaA09g06030.1D2
Resyn_Go_S4	BnaC09g04660.1D2	BnaA09g06040.1D2
Resyn_Go_S4	BnaC09g04690.1D2	BnaA09g06070.1D2
Resyn_Go_S4	BnaC09g04730.1D2	BnaA09g06100.1D2
Resyn_Go_S4	BnaC09g04870.1D2	BnaA09g06260.1D2
Resyn_Go_S4	BnaC09g04890.1D2	BnaA09g06280.1D2
Resyn_Go_S4	BnaC09g04900.1D2	BnaA09g06290.1D2

Resyn_Go_S4	BnaC09g04950.1D2	BnaA09g06330.1D2
Resyn_Go_S4	BnaC09g05080.1D2	BnaA09g06450.1D2
Resyn_Go_S4	BnaC09g05160.1D2	BnaA09g06530.1D2
Resyn_Go_S4	BnaC09g05170.1D2	BnaA09g06540.1D2
Resyn_Go_S4	BnaC09g05200.1D2	BnaA09g06560.1D2
Resyn_Go_S4	BnaC09g05210.1D2	BnaA09g06570.1D2
Resyn_Go_S4	BnaC09g05250.1D2	BnaA09g06620.1D2
Resyn_Go_S4	BnaC09g05280.1D2	BnaA09g06640.1D2
Resyn_Go_S4	BnaC09g05290.1D2	BnaA09g06650.1D2
Resyn_Go_S4	BnaC09g05730.1D2	BnaA09g04700.1D2
Resyn_Go_S4	BnaC09g06970.1D2	BnaA09g07830.1D2
Resyn_Go_S4	BnaC09g07350.1D2	BnaA09g08170.1D2
Resyn_Go_S4	BnaC09g07360.1D2	BnaA09g08180.1D2
Resyn_Go_S4	BnaC09g07430.1D2	BnaA09g08250.1D2
Resyn_Go_S4	BnaC09g07470.1D2	BnaA09g08790.1D2
Resyn_Go_S4	BnaC09g07520.1D2	BnaA09g08780.1D2
Resyn_Go_S4	BnaC09g07590.1D2	BnaA09g08840.1D2
Resyn_Go_S4	BnaC09g07640.1D2	BnaA09g08310.1D2
Resyn_Go_S4	BnaC09g07710.1D2	BnaA09g08360.1D2
Resyn_Go_S4	BnaC09g07730.1D2	BnaA09g08380.1D2
Resyn_Go_S4	BnaC09g07770.1D2	BnaA09g08410.1D2
Resyn_Go_S4	BnaC09g07820.1D2	BnaA09g08450.1D2
Resyn_Go_S4	BnaC09g07850.1D2	BnaA09g08460.1D2
Resyn_Go_S4	BnaC09g07890.1D2	BnaA09g08490.1D2
Resyn_Go_S4	BnaC09g07920.1D2	BnaA09g08510.1D2
RS_7_6	BnaC09g00060.1D2	BnaA09g01630.1D2
RS_7_6	BnaC09g00180.1D2	BnaA09g01740.1D2
RS_7_6	BnaC09g00200.1D2	BnaA09g01760.1D2
RS_7_6	BnaC09g00220.1D2	BnaA09g01780.1D2
RS_7_6	BnaC09g00440.1D2	BnaA09g01920.1D2
RS_7_6	BnaC09g00520.1D2	BnaA09g02000.1D2
RS_7_6	BnaC09g00600.1D2	BnaA09g02090.1D2
RS_7_6	BnaC09g00640.1D2	BnaA09g02130.1D2
RS_7_6	BnaC09g00900.1D2	BnaA09g02050.1D2
RS_7_6	BnaC09g00990.1D2	BnaA09g02430.1D2
RS_7_6	BnaC09g01190.1D2	BnaA09g00950.1D2
RS_7_6	BnaC09g01190.1D2	BnaA09g00960.1D2
RS_7_6	BnaC09g01490.1D2	BnaA09g02780.1D2
RS_7_6	BnaC09g01740.1D2	BnaA09g03000.1D2
RS_7_6	BnaC09g01830.1D2	BnaA09g03120.1D2
RS_7_6	BnaC09g01910.1D2	BnaA09g03210.1D2
RS_7_6	BnaC09g01960.1D2	BnaA09g03260.1D2
RS_7_6	BnaC09g02060.1D2	BnaA09g03450.1D2
RS_7_6	BnaC09g02070.1D2	BnaA09g03460.1D2
RS_7_6	BnaC09g02180.1D2	BnaA09g03610.1D2
RS_7_6	BnaC09g02240.1D2	BnaA09g03670.1D2
RS_7_6	BnaC09g02260.1D2	BnaA09g03690.1D2
RS_7_6	BnaC09g02270.1D2	BnaA09g03700.1D2
RS_7_6	BnaC09g02310.1D2	BnaA09g03750.1D2
RS_7_6	BnaC09g02360.1D2	BnaA09g03770.1D2
RS_7_6	BnaC09g02630.1D2	BnaA09g00840.1D2

RS_7_6	BnaC09g02670.1D2	BnaA09g04300.1D2
RS_7_6	BnaC09g02690.1D2	BnaA09g04330.1D2
RS_7_6	BnaC09g02950.1D2	BnaA09g04570.1D2
RS_7_6	BnaC09g03040.1D2	BnaA09g03300.1D2
RS_7_6	BnaC09g03130.1D2	BnaA09g03310.1D2
RS_7_6	BnaC09g03170.1D2	BnaA09g03350.1D2
RS_7_6	BnaC09g03180.1D2	BnaA09g01410.1D2
RS_7_6	BnaC09g03220.1D2	BnaA09g01370.1D2
RS_7_6	BnaC09g03340.1D2	BnaA09g04750.1D2
RS_7_6	BnaC09g03380.1D2	BnaA09g04770.1D2
RS_7_6	BnaC09g03500.1D2	BnaA09g04890.1D2
RS_7_6	BnaC09g03540.1D2	BnaA09g04920.1D2
RS_7_6	BnaC09g03690.1D2	BnaA09g05100.1D2
RS_7_6	BnaC09g03710.1D2	BnaA09g05120.1D2
RS_7_6	BnaC09g03750.1D2	BnaA09g05140.1D2
RS_7_6	BnaC09g03960.1D2	BnaA09g05380.1D2
RS_7_6	BnaC09g03980.1D2	BnaA09g05410.1D2
RS_7_6	BnaC09g04080.1D2	BnaA09g05460.1D2
RS_7_6	BnaC09g04190.1D2	BnaA09g05560.1D2
RS_7_6	BnaC09g04270.1D2	BnaA09g05630.1D2
RS_7_6	BnaC09g04640.1D2	BnaA09g06020.1D2
RS_7_6	BnaC09g04730.1D2	BnaA09g06100.1D2
RS_7_6	BnaC09g04890.1D2	BnaA09g06280.1D2
RS_7_6	BnaC09g05030.1D2	BnaA09g06350.1D2
RS_7_6	BnaC09g05200.1D2	BnaA09g06560.1D2
RS_7_6	BnaC09g05210.1D2	BnaA09g06570.1D2
RS_7_6	BnaC09g05230.1D2	BnaA09g06590.1D2
RS_7_6	BnaC09g05260.1D2	BnaA09g06630.1D2
RS_7_6	BnaC09g05280.1D2	BnaA09g06640.1D2
RS_7_6	BnaC09g05360.1D2	BnaA09g06700.1D2
RS_7_6	BnaC09g05390.1D2	BnaA09g06720.1D2
RS_7_6	BnaC09g05470.1D2	BnaA09g06850.1D2
RS_7_6	BnaC09g05600.1D2	BnaA09g06750.1D2
RS_7_6	BnaC09g05930.1D2	BnaA09g07050.1D2
RS_7_6	BnaC09g06250.1D2	BnaA09g07250.1D2
RS_7_6	BnaC09g06280.1D2	BnaA09g07270.1D2
RS_7_6	BnaC09g06440.1D2	BnaA09g07430.1D2
RS_7_6	BnaC09g06720.1D2	BnaA09g07650.1D2
RS_7_6	BnaC09g06760.1D2	BnaA09g07690.1D2
RS_7_6	BnaC09g06780.1D2	BnaA09g07700.1D2
RS_7_6	BnaC09g06790.1D2	BnaA09g07710.1D2
RS_7_6	BnaC09g06830.1D2	BnaA09g07760.1D2
RS_7_6	BnaC09g06890.1D2	BnaA09g08900.1D2
RS_7_6	BnaC09g06910.1D2	BnaA09g08890.1D2
RS_7_6	BnaC09g06930.1D2	BnaA09g08870.1D2
RS_7_6	BnaC09g07310.1D2	BnaA09g08120.1D2
RS_7_6	BnaC09g07330.1D2	BnaA09g08140.1D2
RS_7_6	BnaC09g07340.1D2	BnaA09g08150.1D2
RS_7_6	BnaC09g07350.1D2	BnaA09g08170.1D2
RS_7_6	BnaC09g07360.1D2	BnaA09g08180.1D2
RS_7_6	BnaC09g07470.1D2	BnaA09g08790.1D2

RS_7_6	BnaC09g07590.1D2	BnaA09g08840.1D2
RS_7_6	BnaC09g07620.1D2	BnaA09g08290.1D2
RS_7_6	BnaC09g07910.1D2	BnaA09g08500.1D2
RS_7_6	BnaC09g08040.1D2	BnaA09g08650.1D2
RS_7_6	BnaC09g08060.1D2	BnaA09g08670.1D2
RS_7_6	BnaC09g08510.1D2	BnaA09g07790.1D2
English_Giant_194	BnaC09g00040.1D2	BnaA09g01590.1D2
English_Giant_194	BnaC09g00060.1D2	BnaA09g01630.1D2
English_Giant_194	BnaC09g00080.1D2	BnaA09g01650.1D2
English_Giant_194	BnaC09g01090.1D2	BnaA09g01510.1D2
English_Giant_194	BnaC09g01130.1D2	BnaA09g01460.1D2
English_Giant_194	BnaC09g01140.1D2	BnaA09g01440.1D2
English_Giant_194	BnaC09g01160.1D2	BnaA09g00920.1D2
English_Giant_194	BnaC09g01170.1D2	BnaA09g00930.1D2
English_Giant_194	BnaC09g01180.1D2	BnaA09g00940.1D2
English_Giant_194	BnaC09g01190.1D2	BnaA09g00950.1D2
English_Giant_194	BnaC09g01190.1D2	BnaA09g00960.1D2
English_Giant_194	BnaC09g01210.1D2	BnaA09g00960.1D2
English_Giant_194	BnaC09g01360.1D2	BnaA09g02550.1D2
English_Giant_194	BnaC09g01470.1D2	BnaA09g02740.1D2
English_Giant_194	BnaC09g01480.1D2	BnaA09g02770.1D2
English_Giant_194	BnaC09g01490.1D2	BnaA09g02780.1D2
English_Giant_194	BnaC09g01500.1D2	BnaA09g02790.1D2
English_Giant_194	BnaC09g01530.1D2	BnaA09g02820.1D2
English_Giant_194	BnaC09g01540.1D2	BnaA09g02840.1D2
English_Giant_194	BnaC09g01610.1D2	BnaA09g02910.1D2
English_Giant_194	BnaC09g01660.1D2	BnaA09g02930.1D2
English_Giant_194	BnaC09g01720.1D2	BnaA09g02980.1D2
English_Giant_194	BnaC09g01740.1D2	BnaA09g03000.1D2
English_Giant_194	BnaC09g01750.1D2	BnaA09g03010.1D2
English_Giant_194	BnaC09g01770.1D2	BnaA09g03020.1D2
English_Giant_194	BnaC09g01780.1D2	BnaA09g03030.1D2
English_Giant_194	BnaC09g01790.1D2	BnaA09g03090.1D2
English_Giant_194	BnaC09g01830.1D2	BnaA09g03120.1D2
English_Giant_194	BnaC09g01850.1D2	BnaA09g03150.1D2
English_Giant_194	BnaC09g01860.1D2	BnaA09g03160.1D2
English_Giant_194	BnaC09g01880.1D2	BnaA09g03180.1D2
English_Giant_194	BnaC09g01910.1D2	BnaA09g03210.1D2
English_Giant_194	BnaC09g01930.1D2	BnaA09g03230.1D2
English_Giant_194	BnaC09g02030.1D2	BnaA09g03420.1D2
English_Giant_194	BnaC09g02070.1D2	BnaA09g03460.1D2
English_Giant_194	BnaC09g02110.1D2	BnaA09g03550.1D2
English_Giant_194	BnaC09g02270.1D2	BnaA09g03700.1D2
English_Giant_194	BnaC09g02290.1D2	BnaA09g03730.1D2
English_Giant_194	BnaC09g02310.1D2	BnaA09g03750.1D2
English_Giant_194	BnaC09g02360.1D2	BnaA09g03770.1D2
English_Giant_194	BnaC09g02440.1D2	BnaA09g03890.1D2
English_Giant_194	BnaC09g02600.1D2	BnaA09g00870.1D2
English_Giant_194	BnaC09g02610.1D2	BnaA09g00860.1D2
English_Giant_194	BnaC09g02760.1D2	BnaA09g04380.1D2
English_Giant_194	BnaC09g02860.1D2	BnaA09g04470.1D2

English_Giant_194	BnaC09g02890.1D2	BnaA09g04520.1D2
English_Giant_194	BnaC09g02920.1D2	BnaA09g04540.1D2
English_Giant_194	BnaC09g02950.1D2	BnaA09g04570.1D2
English_Giant_194	BnaC09g03000.1D2	BnaA09g04640.1D2
English_Giant_194	BnaC09g03040.1D2	BnaA09g03300.1D2
English_Giant_194	BnaC09g03090.1D2	BnaA09g03380.1D2
English_Giant_194	BnaC09g03170.1D2	BnaA09g03350.1D2
English_Giant_194	BnaC09g03180.1D2	BnaA09g01410.1D2
English_Giant_194	BnaC09g03250.1D2	BnaA09g01340.1D2
English_Giant_194	BnaC09g03270.1D2	BnaA09g01320.1D2
English_Giant_194	BnaC09g03280.1D2	BnaA09g01310.1D2
English_Giant_194	BnaC09g03340.1D2	BnaA09g04750.1D2
English_Giant_194	BnaC09g03360.1D2	BnaA09g04760.1D2
English_Giant_194	BnaC09g03380.1D2	BnaA09g04770.1D2
English_Giant_194	BnaC09g03390.1D2	BnaA09g04770.1D2
English_Giant_194	BnaC09g03410.1D2	BnaA09g04790.1D2
English_Giant_194	BnaC09g03460.1D2	BnaA09g04840.1D2
English_Giant_194	BnaC09g03480.1D2	BnaA09g04870.1D2
English_Giant_194	BnaC09g03490.1D2	BnaA09g04880.1D2
English_Giant_194	BnaC09g03500.1D2	BnaA09g04890.1D2
English_Giant_194	BnaC09g03520.1D2	BnaA09g04910.1D2
English_Giant_194	BnaC09g03540.1D2	BnaA09g04920.1D2
English_Giant_194	BnaC09g03690.1D2	BnaA09g05100.1D2
English_Giant_194	BnaC09g03710.1D2	BnaA09g05120.1D2
English_Giant_194	BnaC09g03730.1D2	BnaA09g05130.1D2
English_Giant_194	BnaC09g03750.1D2	BnaA09g05140.1D2
English_Giant_194	BnaC09g03960.1D2	BnaA09g05380.1D2
English_Giant_194	BnaC09g04030.1D2	BnaA09g05450.1D2
English_Giant_194	BnaC09g04260.1D2	BnaA09g05610.1D2
English_Giant_194	BnaC09g04270.1D2	BnaA09g05630.1D2
English_Giant_194	BnaC09g04590.1D2	BnaA09g05950.1D2
English_Giant_194	BnaC09g04600.1D2	BnaA09g05960.1D2
English_Giant_194	BnaC09g04650.1D2	BnaA09g06030.1D2
English_Giant_194	BnaC09g04660.1D2	BnaA09g06040.1D2
English_Giant_194	BnaC09g04720.1D2	BnaA09g06090.1D2
English_Giant_194	BnaC09g04740.1D2	BnaA09g06110.1D2
English_Giant_194	BnaC09g04890.1D2	BnaA09g06280.1D2
English_Giant_194	BnaC09g04900.1D2	BnaA09g06290.1D2
English_Giant_194	BnaC09g04920.1D2	BnaA09g06300.1D2
English_Giant_194	BnaC09g04950.1D2	BnaA09g06330.1D2
English_Giant_194	BnaC09g05080.1D2	BnaA09g06450.1D2
English_Giant_194	BnaC09g05170.1D2	BnaA09g06540.1D2
English_Giant_194	BnaC09g05200.1D2	BnaA09g06560.1D2
English_Giant_194	BnaC09g05230.1D2	BnaA09g06590.1D2
English_Giant_194	BnaC09g05250.1D2	BnaA09g06620.1D2
English_Giant_194	BnaC09g05290.1D2	BnaA09g06650.1D2
English_Giant_194	BnaC09g05360.1D2	BnaA09g06700.1D2
English_Giant_194	BnaC09g05600.1D2	BnaA09g06750.1D2
English_Giant_194	BnaC09g05630.1D2	BnaA09g06170.1D2
English_Giant_194	BnaC09g05700.1D2	BnaA09g06010.1D2
English_Giant_194	BnaC09g05720.1D2	BnaA09g04690.1D2

English_Giant_194	BnaC09g09810.1D2	BnaA09g28480.1D2

List of HE genes (C09 to A10)

**Accession**	**Lost gene**	**Duplicated HE gene**
H149	BnaC09g43430.1D2	BnaA10g23620.1D2
H149	BnaC09g43490.1D2	BnaA10g23680.1D2
H149	BnaC09g43560.1D2	BnaA10g23750.1D2
H149	BnaC09g43620.1D2	BnaA10g23830.1D2
H149	BnaC09g43790.1D2	BnaA10g26570.1D2
H149	BnaC09g43830.1D2	BnaA10g26540.1D2
H149	BnaC09g43840.1D2	BnaA10g26530.1D2
H149	BnaC09g43990.1D2	BnaA10g23920.1D2
H149	BnaC09g44010.1D2	BnaA10g23930.1D2
H149	BnaC09g44040.1D2	BnaA10g23960.1D2
H149	BnaC09g44070.1D2	BnaA10g24000.1D2
H149	BnaC09g44080.1D2	BnaA10g24010.1D2
H149	BnaC09g44140.1D2	BnaA10g24070.1D2
H149	BnaC09g44200.1D2	BnaA10g24130.1D2
H149	BnaC09g44210.1D2	BnaA10g24130.1D2
H149	BnaC09g44240.1D2	BnaA10g24170.1D2
H149	BnaC09g44280.1D2	BnaA10g24210.1D2
H149	BnaC09g44340.1D2	BnaA10g24270.1D2
H149	BnaC09g44440.1D2	BnaA10g24360.1D2
H149	BnaC09g44450.1D2	BnaA10g24370.1D2
H149	BnaC09g44530.1D2	BnaA10g24450.1D2
H149	BnaC09g44560.1D2	BnaA10g24480.1D2
H149	BnaC09g44610.1D2	BnaA10g24540.1D2
H149	BnaC09g44700.1D2	BnaA10g24640.1D2
H149	BnaC09g44770.1D2	BnaA10g24730.1D2
H149	BnaC09g44810.1D2	BnaA10g24760.1D2
H149	BnaC09g45190.1D2	BnaA10g27510.1D2
H149	BnaC09g45200.1D2	BnaA10g27500.1D2
H149	BnaC09g45240.1D2	BnaA10g27460.1D2
H149	BnaC09g45350.1D2	BnaA10g25290.1D2
H149	BnaC09g45380.1D2	BnaA10g25270.1D2
H149	BnaC09g45450.1D2	BnaA10g25310.1D2
H149	BnaC09g45490.1D2	BnaA10g25350.1D2
H149	BnaC09g45550.1D2	BnaA10g25410.1D2
H149	BnaC09g45750.1D2	BnaA10g25220.1D2
H149	BnaC09g45760.1D2	BnaA10g25210.1D2
H149	BnaC09g45940.1D2	BnaA10g25020.1D2
H149	BnaC09g45980.1D2	BnaA10g24980.1D2
H149	BnaC09g46070.1D2	BnaA10g24900.1D2
