# Supplementary material for: Homoeologous exchange is a major cause of gene presence/absence variation in the amphidiploid Brassica napus
Source: Plant Biotechnol J. 2018 Jan 10;16(7):1265–74. doi: 10.1111/pbi.12867 (PMC5999312; doi:10.1111/pbi.12867)
Supplement: Supplementary file 5 — Data S2 Gene PAV of the flowering time regulators FLC, PHYA and GA3ox1 in the ERANET‐ASSYST B. napus diversity set. Swede lines are shown in red. [file PBI-16-1265-s001.pdf]

Supplementary File 3: Gene PAV of the flowering time regulators *FLC* , *PHYA* and *GA3ox1* in the ERANET-ASSYST *B. napus* diversity set. Swede lines are shown in red.

Gene name	Gene ID from public Darmor-*bzh* reference (v4.0)	Gene ID from Darmor-*bzh* assembly (v8.1) used as basis for this pangenome study
Bna.PHYA.chrA09	BnaA09g48410D	BnaA09g49460.1D2
Bna.GA3ox.chrA09.random	BnaA09g57140D	BnaA09g49330.1D2
Bna.FLC.chrA10	BnaA10g22080D	BnaA10g21650.1D2
Bna.CCR1.chrC08	BnaC08g38580D	BnaC08g34000.1D2
Bna.GA3ox.chrC08	BnaC08g38810D	BnaC08g34160.1D2
Bna.PHYA.chrC08	BnaC08g42660D	BnaC08g37880.1D2
germin like protein	BnaC08g42670D	BnaC08g37890.1D2
Bna.FLC.chrC09	BnaC09g46500D	BnaC09g41360.1D2
Bna.FLC.chrC09	BnaC09g46540D	BnaC09g41390.1D2

Assyst name	BnaA09g49460.1D2	BnaA09g49330.1D2	BnaA10g21650.1D2	BnaC08g34000.1D2	BnaC08g34160.1D2	BnaC08g37880.1D2
ASSYST419	PRESENT	PRESENT	PRESENT	LOST	LOST	LOST
ASSYST424	PRESENT	PRESENT	PRESENT	LOST	LOST	LOST
ASSYST427	PRESENT	PRESENT	PRESENT	PRESENT	LOST	LOST
ASSYST430	PRESENT	PRESENT	PRESENT	LOST	LOST	LOST
ASSYST431	PRESENT	PRESENT	PRESENT	LOST	LOST	LOST
ASSYST433	PRESENT	PRESENT	PRESENT	LOST	LOST	LOST
ASSYST436	PRESENT	PRESENT	PRESENT	LOST	LOST	LOST
ASSYST447	PRESENT	PRESENT	PRESENT	LOST	LOST	LOST
ASSYST453	PRESENT	PRESENT	PRESENT	LOST	LOST	LOST
ASSYST454	PRESENT	PRESENT	PRESENT	PRESENT	PRESENT	PRESENT
ASSYST001	PRESENT	PRESENT	PRESENT	PRESENT	PRESENT	PRESENT
ASSYST002	PRESENT	PRESENT	PRESENT	PRESENT	PRESENT	PRESENT
ASSYST003	PRESENT	PRESENT	PRESENT	PRESENT	PRESENT	PRESENT
ASSYST004	PRESENT	PRESENT	PRESENT	PRESENT	PRESENT	PRESENT

ASSYST005	LOST	PRESENT	PRESENT	PRESENT	PRESENT	PRESENT
ASSYST006	PRESENT	PRESENT	PRESENT	PRESENT	PRESENT	PRESENT
ASSYST007	PRESENT	PRESENT	PRESENT	PRESENT	PRESENT	PRESENT
ASSYST008	PRESENT	PRESENT	PRESENT	PRESENT	PRESENT	PRESENT
ASSYST009	PRESENT	PRESENT	PRESENT	PRESENT	PRESENT	PRESENT
ASSYST011	PRESENT	PRESENT	PRESENT	PRESENT	PRESENT	PRESENT
ASSYST012	PRESENT	PRESENT	PRESENT	PRESENT	PRESENT	PRESENT
ASSYST013	PRESENT	PRESENT	PRESENT	PRESENT	PRESENT	PRESENT
ASSYST015	PRESENT	PRESENT	PRESENT	PRESENT	PRESENT	PRESENT
ASSYST016	PRESENT	PRESENT	PRESENT	PRESENT	PRESENT	PRESENT
ASSYST017	PRESENT	PRESENT	PRESENT	PRESENT	PRESENT	PRESENT
ASSYST018	PRESENT	PRESENT	PRESENT	PRESENT	PRESENT	PRESENT
ASSYST020	PRESENT	PRESENT	PRESENT	PRESENT	PRESENT	PRESENT
ASSYST021	PRESENT	PRESENT	PRESENT	PRESENT	PRESENT	PRESENT
ASSYST022	PRESENT	PRESENT	PRESENT	PRESENT	PRESENT	PRESENT
ASSYST023	PRESENT	PRESENT	PRESENT	PRESENT	PRESENT	PRESENT
ASSYST024	PRESENT	PRESENT	PRESENT	PRESENT	PRESENT	PRESENT
ASSYST025	PRESENT	PRESENT	PRESENT	PRESENT	PRESENT	PRESENT
ASSYST026	PRESENT	PRESENT	PRESENT	LOST	LOST	LOST
ASSYST027	PRESENT	PRESENT	PRESENT	PRESENT	PRESENT	PRESENT
ASSYST029	PRESENT	PRESENT	PRESENT	PRESENT	PRESENT	PRESENT
ASSYST030	PRESENT	PRESENT	PRESENT	PRESENT	PRESENT	PRESENT
ASSYST031	PRESENT	PRESENT	PRESENT	PRESENT	PRESENT	PRESENT
ASSYST032	PRESENT	PRESENT	PRESENT	PRESENT	PRESENT	PRESENT
ASSYST033	PRESENT	PRESENT	PRESENT	PRESENT	PRESENT	PRESENT
ASSYST034	PRESENT	PRESENT	PRESENT	PRESENT	PRESENT	PRESENT
ASSYST036	PRESENT	PRESENT	PRESENT	PRESENT	PRESENT	PRESENT
ASSYST037	PRESENT	PRESENT	PRESENT	PRESENT	PRESENT	PRESENT
ASSYST038	PRESENT	PRESENT	PRESENT	PRESENT	PRESENT	PRESENT
ASSYST039	PRESENT	PRESENT	PRESENT	PRESENT	PRESENT	PRESENT
ASSYST040	PRESENT	PRESENT	PRESENT	PRESENT	PRESENT	PRESENT
ASSYST041	PRESENT	PRESENT	PRESENT	PRESENT	PRESENT	PRESENT
ASSYST044	PRESENT	PRESENT	PRESENT	PRESENT	PRESENT	PRESENT
ASSYST045	PRESENT	PRESENT	PRESENT	PRESENT	PRESENT	PRESENT
ASSYST046	PRESENT	PRESENT	PRESENT	PRESENT	PRESENT	PRESENT

ASSYST047	PRESENT	PRESENT	PRESENT	PRESENT	PRESENT	PRESENT
ASSYST048	PRESENT	PRESENT	PRESENT	PRESENT	PRESENT	PRESENT
ASSYST049	PRESENT	PRESENT	PRESENT	PRESENT	PRESENT	PRESENT
ASSYST050	PRESENT	PRESENT	PRESENT	PRESENT	PRESENT	PRESENT
ASSYST052	PRESENT	PRESENT	PRESENT	PRESENT	PRESENT	PRESENT
ASSYST053	PRESENT	PRESENT	PRESENT	PRESENT	PRESENT	PRESENT
ASSYST054	PRESENT	PRESENT	PRESENT	PRESENT	PRESENT	PRESENT
ASSYST055	PRESENT	PRESENT	PRESENT	PRESENT	PRESENT	PRESENT
ASSYST057	PRESENT	PRESENT	PRESENT	PRESENT	PRESENT	PRESENT
ASSYST058	PRESENT	PRESENT	PRESENT	PRESENT	PRESENT	PRESENT
ASSYST059	PRESENT	PRESENT	PRESENT	PRESENT	PRESENT	PRESENT
ASSYST060	PRESENT	PRESENT	PRESENT	PRESENT	PRESENT	PRESENT
ASSYST061	PRESENT	PRESENT	PRESENT	PRESENT	PRESENT	PRESENT
ASSYST062	PRESENT	PRESENT	PRESENT	PRESENT	PRESENT	PRESENT
ASSYST063	PRESENT	PRESENT	PRESENT	PRESENT	PRESENT	PRESENT
ASSYST064	PRESENT	PRESENT	PRESENT	PRESENT	PRESENT	PRESENT
ASSYST065	PRESENT	PRESENT	PRESENT	PRESENT	PRESENT	PRESENT
ASSYST066	PRESENT	PRESENT	PRESENT	PRESENT	PRESENT	PRESENT
ASSYST067	PRESENT	PRESENT	PRESENT	PRESENT	PRESENT	PRESENT
ASSYST068	PRESENT	PRESENT	PRESENT	PRESENT	PRESENT	PRESENT
ASSYST069	PRESENT	PRESENT	PRESENT	PRESENT	PRESENT	PRESENT
ASSYST070	PRESENT	PRESENT	PRESENT	PRESENT	PRESENT	PRESENT
ASSYST071	PRESENT	PRESENT	PRESENT	PRESENT	PRESENT	PRESENT
ASSYST072	PRESENT	PRESENT	PRESENT	PRESENT	PRESENT	PRESENT
ASSYST073	PRESENT	PRESENT	PRESENT	PRESENT	PRESENT	PRESENT
ASSYST074	PRESENT	PRESENT	PRESENT	PRESENT	PRESENT	PRESENT
ASSYST075	PRESENT	PRESENT	PRESENT	PRESENT	PRESENT	PRESENT
ASSYST076	PRESENT	PRESENT	PRESENT	PRESENT	PRESENT	PRESENT
ASSYST077	PRESENT	PRESENT	PRESENT	PRESENT	PRESENT	PRESENT
ASSYST078	PRESENT	PRESENT	PRESENT	PRESENT	PRESENT	PRESENT
ASSYST079	PRESENT	PRESENT	PRESENT	PRESENT	PRESENT	PRESENT
ASSYST080	PRESENT	PRESENT	PRESENT	PRESENT	PRESENT	PRESENT
ASSYST081	PRESENT	PRESENT	PRESENT	PRESENT	PRESENT	PRESENT
ASSYST082	PRESENT	PRESENT	PRESENT	PRESENT	PRESENT	PRESENT
ASSYST083	PRESENT	PRESENT	PRESENT	PRESENT	PRESENT	PRESENT

ASSYST084	PRESENT	PRESENT	PRESENT	PRESENT	PRESENT	PRESENT
ASSYST085	PRESENT	PRESENT	PRESENT	PRESENT	PRESENT	PRESENT
ASSYST086	PRESENT	PRESENT	PRESENT	PRESENT	PRESENT	PRESENT
ASSYST087	PRESENT	PRESENT	PRESENT	PRESENT	PRESENT	PRESENT
ASSYST088	PRESENT	PRESENT	PRESENT	PRESENT	PRESENT	PRESENT
ASSYST112	PRESENT	PRESENT	PRESENT	PRESENT	PRESENT	PRESENT
ASSYST113	PRESENT	PRESENT	PRESENT	PRESENT	PRESENT	PRESENT
ASSYST114	PRESENT	PRESENT	PRESENT	PRESENT	PRESENT	PRESENT
ASSYST115	PRESENT	PRESENT	PRESENT	PRESENT	PRESENT	PRESENT
ASSYST116	PRESENT	PRESENT	PRESENT	PRESENT	PRESENT	PRESENT
ASSYST117	PRESENT	PRESENT	PRESENT	PRESENT	PRESENT	PRESENT
ASSYST118	PRESENT	PRESENT	PRESENT	PRESENT	PRESENT	PRESENT
ASSYST119	PRESENT	PRESENT	PRESENT	PRESENT	PRESENT	PRESENT
ASSYST120	PRESENT	PRESENT	PRESENT	PRESENT	PRESENT	PRESENT
ASSYST121	PRESENT	PRESENT	PRESENT	PRESENT	PRESENT	PRESENT
ASSYST122	PRESENT	PRESENT	PRESENT	PRESENT	PRESENT	PRESENT
ASSYST123	PRESENT	PRESENT	PRESENT	PRESENT	PRESENT	PRESENT
ASSYST124	PRESENT	PRESENT	PRESENT	PRESENT	PRESENT	PRESENT
ASSYST125	PRESENT	PRESENT	PRESENT	PRESENT	PRESENT	PRESENT
ASSYST126	PRESENT	PRESENT	PRESENT	PRESENT	PRESENT	PRESENT
ASSYST127	PRESENT	PRESENT	PRESENT	PRESENT	PRESENT	PRESENT
ASSYST128	PRESENT	PRESENT	PRESENT	PRESENT	PRESENT	PRESENT
ASSYST129	PRESENT	PRESENT	PRESENT	PRESENT	PRESENT	PRESENT
ASSYST130	PRESENT	PRESENT	PRESENT	PRESENT	PRESENT	PRESENT
ASSYST131	PRESENT	PRESENT	PRESENT	PRESENT	PRESENT	PRESENT
ASSYST132	PRESENT	PRESENT	PRESENT	PRESENT	PRESENT	PRESENT
ASSYST133	PRESENT	PRESENT	PRESENT	PRESENT	PRESENT	PRESENT
ASSYST134	PRESENT	PRESENT	PRESENT	PRESENT	PRESENT	PRESENT
ASSYST135	PRESENT	PRESENT	PRESENT	PRESENT	PRESENT	PRESENT
ASSYST136	PRESENT	PRESENT	PRESENT	PRESENT	PRESENT	PRESENT
ASSYST137	PRESENT	PRESENT	PRESENT	PRESENT	PRESENT	PRESENT
ASSYST138	LOST	LOST	LOST	LOST	LOST	LOST
ASSYST139	PRESENT	PRESENT	PRESENT	PRESENT	PRESENT	PRESENT
ASSYST140	PRESENT	PRESENT	PRESENT	PRESENT	PRESENT	PRESENT
ASSYST141	PRESENT	PRESENT	PRESENT	PRESENT	PRESENT	PRESENT

ASSYST142	PRESENT	PRESENT	PRESENT	PRESENT	PRESENT	PRESENT
ASSYST143	PRESENT	PRESENT	PRESENT	PRESENT	PRESENT	PRESENT
ASSYST144	PRESENT	PRESENT	PRESENT	PRESENT	PRESENT	PRESENT
ASSYST145	PRESENT	PRESENT	PRESENT	PRESENT	PRESENT	PRESENT
ASSYST146	PRESENT	PRESENT	PRESENT	PRESENT	PRESENT	PRESENT
ASSYST147	PRESENT	PRESENT	PRESENT	PRESENT	PRESENT	PRESENT
ASSYST149	PRESENT	PRESENT	PRESENT	PRESENT	PRESENT	PRESENT
ASSYST150	PRESENT	PRESENT	PRESENT	PRESENT	PRESENT	PRESENT
ASSYST151	PRESENT	PRESENT	PRESENT	PRESENT	PRESENT	PRESENT
ASSYST153	PRESENT	PRESENT	PRESENT	PRESENT	PRESENT	PRESENT
ASSYST154	PRESENT	PRESENT	PRESENT	PRESENT	PRESENT	PRESENT
ASSYST155	PRESENT	PRESENT	PRESENT	PRESENT	PRESENT	PRESENT
ASSYST156	PRESENT	PRESENT	PRESENT	PRESENT	PRESENT	PRESENT
ASSYST157	PRESENT	PRESENT	PRESENT	PRESENT	PRESENT	PRESENT
ASSYST158	PRESENT	PRESENT	PRESENT	PRESENT	PRESENT	PRESENT
ASSYST159	PRESENT	PRESENT	PRESENT	PRESENT	PRESENT	PRESENT
ASSYST160	PRESENT	PRESENT	PRESENT	PRESENT	PRESENT	PRESENT
ASSYST162	PRESENT	PRESENT	PRESENT	PRESENT	PRESENT	PRESENT
ASSYST164	PRESENT	PRESENT	PRESENT	PRESENT	PRESENT	PRESENT
ASSYST165	PRESENT	PRESENT	PRESENT	PRESENT	PRESENT	PRESENT
ASSYST166	PRESENT	PRESENT	PRESENT	PRESENT	PRESENT	PRESENT
ASSYST167	PRESENT	PRESENT	PRESENT	PRESENT	PRESENT	PRESENT
ASSYST170	PRESENT	PRESENT	PRESENT	PRESENT	PRESENT	PRESENT
ASSYST171	PRESENT	PRESENT	PRESENT	PRESENT	PRESENT	PRESENT
ASSYST172	PRESENT	PRESENT	PRESENT	PRESENT	PRESENT	PRESENT
ASSYST174	PRESENT	PRESENT	PRESENT	PRESENT	PRESENT	PRESENT
ASSYST175	PRESENT	PRESENT	PRESENT	PRESENT	PRESENT	PRESENT
ASSYST176	PRESENT	PRESENT	PRESENT	PRESENT	PRESENT	PRESENT
ASSYST178	PRESENT	PRESENT	PRESENT	PRESENT	PRESENT	PRESENT
ASSYST179	PRESENT	PRESENT	PRESENT	PRESENT	PRESENT	PRESENT
ASSYST180	PRESENT	PRESENT	PRESENT	PRESENT	PRESENT	PRESENT
ASSYST181	LOST	PRESENT	PRESENT	PRESENT	PRESENT	PRESENT
ASSYST183	PRESENT	PRESENT	PRESENT	PRESENT	PRESENT	PRESENT
ASSYST190	PRESENT	PRESENT	PRESENT	PRESENT	PRESENT	PRESENT
ASSYST200	PRESENT	PRESENT	PRESENT	PRESENT	PRESENT	PRESENT

ASSYST203	PRESENT	PRESENT	PRESENT	PRESENT	PRESENT	PRESENT
ASSYST206	PRESENT	PRESENT	PRESENT	PRESENT	PRESENT	PRESENT
ASSYST228	PRESENT	PRESENT	PRESENT	PRESENT	PRESENT	PRESENT
ASSYST229	LOST	PRESENT	PRESENT	PRESENT	PRESENT	PRESENT
ASSYST230	PRESENT	PRESENT	PRESENT	PRESENT	LOST	LOST
ASSYST232	PRESENT	PRESENT	PRESENT	PRESENT	PRESENT	PRESENT
ASSYST234	PRESENT	PRESENT	PRESENT	PRESENT	PRESENT	PRESENT
ASSYST236	PRESENT	PRESENT	PRESENT	PRESENT	PRESENT	PRESENT
ASSYST237	PRESENT	PRESENT	PRESENT	PRESENT	PRESENT	PRESENT
ASSYST238	PRESENT	PRESENT	PRESENT	PRESENT	PRESENT	PRESENT
ASSYST239	PRESENT	PRESENT	PRESENT	PRESENT	PRESENT	PRESENT
ASSYST240	PRESENT	PRESENT	PRESENT	PRESENT	PRESENT	PRESENT
ASSYST241	PRESENT	PRESENT	PRESENT	PRESENT	PRESENT	PRESENT
ASSYST242	LOST	PRESENT	PRESENT	PRESENT	PRESENT	PRESENT
ASSYST244	PRESENT	PRESENT	PRESENT	PRESENT	PRESENT	PRESENT
ASSYST251	PRESENT	PRESENT	PRESENT	PRESENT	PRESENT	PRESENT
ASSYST252	PRESENT	PRESENT	PRESENT	PRESENT	PRESENT	PRESENT
ASSYST253	PRESENT	PRESENT	PRESENT	PRESENT	PRESENT	PRESENT
ASSYST254	PRESENT	PRESENT	PRESENT	PRESENT	PRESENT	PRESENT
ASSYST256	PRESENT	PRESENT	PRESENT	PRESENT	PRESENT	PRESENT
ASSYST257	PRESENT	PRESENT	PRESENT	PRESENT	PRESENT	PRESENT
ASSYST258	PRESENT	PRESENT	PRESENT	PRESENT	PRESENT	PRESENT
ASSYST259	PRESENT	PRESENT	PRESENT	PRESENT	PRESENT	PRESENT
ASSYST260	PRESENT	PRESENT	PRESENT	PRESENT	PRESENT	PRESENT
ASSYST261	PRESENT	PRESENT	PRESENT	PRESENT	PRESENT	PRESENT
ASSYST263	LOST	PRESENT	PRESENT	PRESENT	PRESENT	PRESENT
ASSYST264	PRESENT	PRESENT	PRESENT	PRESENT	PRESENT	PRESENT
ASSYST265	PRESENT	PRESENT	PRESENT	PRESENT	PRESENT	PRESENT
ASSYST267	LOST	PRESENT	PRESENT	PRESENT	PRESENT	PRESENT
ASSYST268	PRESENT	PRESENT	PRESENT	PRESENT	PRESENT	PRESENT
ASSYST270	PRESENT	PRESENT	PRESENT	PRESENT	PRESENT	PRESENT
ASSYST271	PRESENT	PRESENT	PRESENT	PRESENT	PRESENT	PRESENT
ASSYST272	PRESENT	PRESENT	PRESENT	PRESENT	PRESENT	PRESENT
ASSYST273	PRESENT	PRESENT	PRESENT	PRESENT	PRESENT	PRESENT
ASSYST274	LOST	PRESENT	PRESENT	PRESENT	PRESENT	PRESENT

ASSYST275	PRESENT	PRESENT	PRESENT	PRESENT	PRESENT	PRESENT
ASSYST276	PRESENT	PRESENT	PRESENT	PRESENT	PRESENT	PRESENT
ASSYST277	PRESENT	PRESENT	PRESENT	PRESENT	PRESENT	PRESENT
ASSYST278	PRESENT	PRESENT	PRESENT	PRESENT	PRESENT	PRESENT
ASSYST280	PRESENT	PRESENT	PRESENT	PRESENT	PRESENT	PRESENT
ASSYST281	LOST	PRESENT	PRESENT	PRESENT	PRESENT	PRESENT
ASSYST282	PRESENT	PRESENT	PRESENT	PRESENT	PRESENT	PRESENT
ASSYST283	PRESENT	PRESENT	PRESENT	PRESENT	PRESENT	PRESENT
ASSYST284	PRESENT	PRESENT	PRESENT	PRESENT	PRESENT	PRESENT
ASSYST285	PRESENT	PRESENT	PRESENT	PRESENT	PRESENT	PRESENT
ASSYST286	PRESENT	PRESENT	PRESENT	PRESENT	PRESENT	PRESENT
ASSYST287	PRESENT	PRESENT	PRESENT	PRESENT	PRESENT	PRESENT
ASSYST289	PRESENT	PRESENT	PRESENT	PRESENT	PRESENT	PRESENT
ASSYST290	PRESENT	PRESENT	PRESENT	PRESENT	PRESENT	PRESENT
ASSYST299	PRESENT	PRESENT	PRESENT	PRESENT	PRESENT	PRESENT
ASSYST300	PRESENT	PRESENT	PRESENT	PRESENT	PRESENT	PRESENT
ASSYST302	PRESENT	PRESENT	PRESENT	PRESENT	PRESENT	PRESENT
ASSYST305	PRESENT	PRESENT	PRESENT	PRESENT	PRESENT	PRESENT
ASSYST306	PRESENT	PRESENT	PRESENT	PRESENT	PRESENT	PRESENT
ASSYST307	PRESENT	PRESENT	PRESENT	PRESENT	PRESENT	PRESENT
ASSYST309	PRESENT	PRESENT	PRESENT	PRESENT	PRESENT	PRESENT
ASSYST311	PRESENT	PRESENT	PRESENT	PRESENT	PRESENT	PRESENT
ASSYST312	PRESENT	PRESENT	LOST	PRESENT	PRESENT	PRESENT
ASSYST313	PRESENT	PRESENT	PRESENT	PRESENT	PRESENT	PRESENT
ASSYST314	PRESENT	PRESENT	PRESENT	PRESENT	PRESENT	PRESENT
ASSYST315	PRESENT	PRESENT	PRESENT	PRESENT	PRESENT	PRESENT
ASSYST316	LOST	PRESENT	PRESENT	PRESENT	PRESENT	PRESENT
ASSYST317	PRESENT	PRESENT	PRESENT	PRESENT	PRESENT	PRESENT
ASSYST318	LOST	PRESENT	PRESENT	PRESENT	PRESENT	PRESENT
ASSYST320	PRESENT	PRESENT	PRESENT	PRESENT	PRESENT	PRESENT
ASSYST321	PRESENT	PRESENT	PRESENT	PRESENT	PRESENT	LOST
ASSYST322	PRESENT	PRESENT	PRESENT	PRESENT	PRESENT	PRESENT
ASSYST323	PRESENT	PRESENT	PRESENT	PRESENT	PRESENT	PRESENT
ASSYST325	LOST	PRESENT	PRESENT	PRESENT	PRESENT	PRESENT
ASSYST327	PRESENT	PRESENT	PRESENT	PRESENT	PRESENT	PRESENT

ASSYST328	PRESENT	PRESENT	PRESENT	PRESENT	PRESENT	PRESENT
ASSYST329	PRESENT	PRESENT	LOST	PRESENT	PRESENT	PRESENT
ASSYST330	PRESENT	PRESENT	PRESENT	PRESENT	PRESENT	PRESENT
ASSYST333	PRESENT	PRESENT	PRESENT	PRESENT	PRESENT	PRESENT
ASSYST334	PRESENT	PRESENT	PRESENT	PRESENT	PRESENT	PRESENT
ASSYST335	PRESENT	PRESENT	PRESENT	PRESENT	PRESENT	PRESENT
ASSYST339	PRESENT	PRESENT	PRESENT	PRESENT	PRESENT	PRESENT
ASSYST340	PRESENT	PRESENT	PRESENT	PRESENT	PRESENT	PRESENT
ASSYST341	PRESENT	PRESENT	PRESENT	PRESENT	PRESENT	PRESENT
ASSYST342	PRESENT	PRESENT	PRESENT	PRESENT	PRESENT	PRESENT
ASSYST344	PRESENT	PRESENT	PRESENT	PRESENT	PRESENT	PRESENT
ASSYST345	PRESENT	PRESENT	PRESENT	PRESENT	PRESENT	PRESENT
ASSYST346	PRESENT	PRESENT	PRESENT	PRESENT	PRESENT	PRESENT
ASSYST347	PRESENT	PRESENT	PRESENT	PRESENT	PRESENT	PRESENT
ASSYST348	LOST	PRESENT	PRESENT	PRESENT	PRESENT	PRESENT
ASSYST349	PRESENT	PRESENT	PRESENT	PRESENT	PRESENT	PRESENT
ASSYST350	PRESENT	PRESENT	PRESENT	PRESENT	PRESENT	PRESENT
ASSYST352	PRESENT	PRESENT	PRESENT	PRESENT	PRESENT	PRESENT
ASSYST354	PRESENT	PRESENT	PRESENT	PRESENT	PRESENT	PRESENT
ASSYST355	PRESENT	PRESENT	PRESENT	PRESENT	PRESENT	PRESENT
ASSYST357	PRESENT	PRESENT	PRESENT	PRESENT	PRESENT	PRESENT
ASSYST359	PRESENT	PRESENT	PRESENT	PRESENT	PRESENT	PRESENT
ASSYST360	PRESENT	PRESENT	PRESENT	PRESENT	PRESENT	PRESENT
ASSYST361	PRESENT	PRESENT	PRESENT	PRESENT	PRESENT	PRESENT
ASSYST362	PRESENT	PRESENT	PRESENT	PRESENT	PRESENT	PRESENT
ASSYST363	PRESENT	PRESENT	PRESENT	LOST	LOST	LOST
ASSYST364	PRESENT	PRESENT	PRESENT	PRESENT	PRESENT	PRESENT
ASSYST365	PRESENT	PRESENT	PRESENT	LOST	LOST	LOST
ASSYST366	PRESENT	PRESENT	PRESENT	PRESENT	PRESENT	PRESENT
ASSYST367	PRESENT	PRESENT	PRESENT	PRESENT	PRESENT	PRESENT
ASSYST368	PRESENT	PRESENT	PRESENT	PRESENT	PRESENT	PRESENT
ASSYST369	PRESENT	PRESENT	PRESENT	PRESENT	PRESENT	PRESENT
ASSYST371	LOST	PRESENT	PRESENT	PRESENT	PRESENT	PRESENT
ASSYST372	PRESENT	PRESENT	PRESENT	PRESENT	PRESENT	PRESENT
ASSYST373	PRESENT	PRESENT	PRESENT	PRESENT	PRESENT	PRESENT

ASSYST374	PRESENT	PRESENT	PRESENT	PRESENT	PRESENT	PRESENT
ASSYST377	PRESENT	PRESENT	PRESENT	PRESENT	PRESENT	PRESENT
ASSYST378	PRESENT	PRESENT	PRESENT	PRESENT	PRESENT	PRESENT
ASSYST379	PRESENT	PRESENT	PRESENT	PRESENT	PRESENT	PRESENT
ASSYST380	PRESENT	PRESENT	PRESENT	PRESENT	PRESENT	PRESENT
ASSYST381	PRESENT	PRESENT	PRESENT	PRESENT	PRESENT	PRESENT
ASSYST382	PRESENT	PRESENT	PRESENT	PRESENT	PRESENT	PRESENT
ASSYST383	LOST	PRESENT	PRESENT	PRESENT	PRESENT	PRESENT
ASSYST384	LOST	PRESENT	PRESENT	PRESENT	PRESENT	PRESENT
ASSYST385	PRESENT	PRESENT	PRESENT	PRESENT	PRESENT	PRESENT
ASSYST386	PRESENT	PRESENT	PRESENT	PRESENT	PRESENT	PRESENT
ASSYST389	PRESENT	PRESENT	PRESENT	PRESENT	PRESENT	PRESENT
ASSYST390	PRESENT	PRESENT	PRESENT	PRESENT	PRESENT	PRESENT
ASSYST391	PRESENT	PRESENT	PRESENT	PRESENT	PRESENT	PRESENT
ASSYST392	PRESENT	PRESENT	PRESENT	PRESENT	PRESENT	PRESENT
ASSYST393	PRESENT	PRESENT	PRESENT	PRESENT	PRESENT	PRESENT
ASSYST394	LOST	PRESENT	PRESENT	PRESENT	PRESENT	PRESENT
ASSYST397	PRESENT	PRESENT	PRESENT	PRESENT	PRESENT	PRESENT
ASSYST398	PRESENT	PRESENT	PRESENT	PRESENT	PRESENT	PRESENT
ASSYST399	PRESENT	PRESENT	PRESENT	PRESENT	PRESENT	PRESENT
ASSYST446	PRESENT	PRESENT	PRESENT	LOST	LOST	LOST

**Assyst name**	**BnaC09g41390.1D2**	**BnaC09g41360.1D2**	**BnaC08g37890.1D2**
ASSYST419	LOST	LOST	LOST
ASSYST424	LOST	LOST	LOST
ASSYST427	LOST	LOST	LOST
ASSYST430	LOST	LOST	LOST
ASSYST431	LOST	LOST	LOST
ASSYST433	LOST	LOST	LOST
ASSYST436	LOST	LOST	LOST
ASSYST447	LOST	LOST	LOST
ASSYST453	LOST	LOST	LOST
ASSYST454	PRESENT	PRESENT	PRESENT
ASSYST001	PRESENT	PRESENT	PRESENT
ASSYST002	PRESENT	PRESENT	PRESENT
ASSYST003	PRESENT	PRESENT	PRESENT

ASSYST004	PRESENT	PRESENT	PRESENT
ASSYST005	PRESENT	PRESENT	PRESENT
ASSYST006	PRESENT	PRESENT	PRESENT
ASSYST007	PRESENT	PRESENT	PRESENT
ASSYST008	PRESENT	PRESENT	PRESENT
ASSYST009	PRESENT	PRESENT	PRESENT
ASSYST011	PRESENT	PRESENT	PRESENT
ASSYST012	PRESENT	PRESENT	PRESENT
ASSYST013	PRESENT	PRESENT	PRESENT
ASSYST015	PRESENT	PRESENT	PRESENT
ASSYST016	PRESENT	PRESENT	PRESENT
ASSYST017	PRESENT	PRESENT	PRESENT
ASSYST018	PRESENT	PRESENT	PRESENT
ASSYST020	PRESENT	PRESENT	PRESENT
ASSYST021	PRESENT	PRESENT	PRESENT
ASSYST022	PRESENT	PRESENT	PRESENT
ASSYST023	PRESENT	PRESENT	PRESENT
ASSYST024	PRESENT	PRESENT	PRESENT
ASSYST025	PRESENT	PRESENT	PRESENT
ASSYST026	PRESENT	PRESENT	LOST
ASSYST027	PRESENT	PRESENT	PRESENT
ASSYST029	PRESENT	PRESENT	PRESENT
ASSYST030	PRESENT	PRESENT	PRESENT
ASSYST031	PRESENT	PRESENT	PRESENT
ASSYST032	PRESENT	PRESENT	PRESENT
ASSYST033	PRESENT	PRESENT	PRESENT
ASSYST034	PRESENT	PRESENT	PRESENT
ASSYST036	PRESENT	PRESENT	PRESENT
ASSYST037	PRESENT	PRESENT	PRESENT
ASSYST038	PRESENT	PRESENT	PRESENT
ASSYST039	PRESENT	PRESENT	PRESENT
ASSYST040	PRESENT	PRESENT	PRESENT
ASSYST041	PRESENT	PRESENT	PRESENT
ASSYST044	PRESENT	PRESENT	PRESENT
ASSYST045	PRESENT	PRESENT	PRESENT

ASSYST046	PRESENT	PRESENT	PRESENT
ASSYST047	PRESENT	PRESENT	PRESENT
ASSYST048	PRESENT	PRESENT	PRESENT
ASSYST049	PRESENT	PRESENT	PRESENT
ASSYST050	PRESENT	PRESENT	PRESENT
ASSYST052	PRESENT	PRESENT	PRESENT
ASSYST053	PRESENT	PRESENT	PRESENT
ASSYST054	PRESENT	PRESENT	PRESENT
ASSYST055	PRESENT	PRESENT	PRESENT
ASSYST057	PRESENT	PRESENT	PRESENT
ASSYST058	PRESENT	PRESENT	PRESENT
ASSYST059	PRESENT	PRESENT	PRESENT
ASSYST060	PRESENT	PRESENT	PRESENT
ASSYST061	PRESENT	PRESENT	PRESENT
ASSYST062	PRESENT	PRESENT	PRESENT
ASSYST063	PRESENT	PRESENT	PRESENT
ASSYST064	PRESENT	PRESENT	PRESENT
ASSYST065	PRESENT	PRESENT	PRESENT
ASSYST066	PRESENT	PRESENT	PRESENT
ASSYST067	PRESENT	PRESENT	PRESENT
ASSYST068	PRESENT	PRESENT	PRESENT
ASSYST069	PRESENT	PRESENT	PRESENT
ASSYST070	LOST	PRESENT	PRESENT
ASSYST071	PRESENT	PRESENT	PRESENT
ASSYST072	PRESENT	PRESENT	PRESENT
ASSYST073	PRESENT	PRESENT	PRESENT
ASSYST074	PRESENT	PRESENT	PRESENT
ASSYST075	PRESENT	PRESENT	PRESENT
ASSYST076	PRESENT	PRESENT	PRESENT
ASSYST077	PRESENT	PRESENT	PRESENT
ASSYST078	PRESENT	PRESENT	PRESENT
ASSYST079	PRESENT	PRESENT	PRESENT
ASSYST080	PRESENT	PRESENT	PRESENT
ASSYST081	PRESENT	PRESENT	PRESENT
ASSYST082	PRESENT	PRESENT	PRESENT

ASSYST083	PRESENT	PRESENT	PRESENT
ASSYST084	PRESENT	PRESENT	PRESENT
ASSYST085	PRESENT	PRESENT	PRESENT
ASSYST086	PRESENT	PRESENT	PRESENT
ASSYST087	PRESENT	PRESENT	PRESENT
ASSYST088	PRESENT	PRESENT	PRESENT
ASSYST112	PRESENT	PRESENT	PRESENT
ASSYST113	PRESENT	PRESENT	PRESENT
ASSYST114	PRESENT	PRESENT	PRESENT
ASSYST115	PRESENT	PRESENT	PRESENT
ASSYST116	PRESENT	PRESENT	PRESENT
ASSYST117	PRESENT	PRESENT	PRESENT
ASSYST118	PRESENT	PRESENT	PRESENT
ASSYST119	PRESENT	PRESENT	PRESENT
ASSYST120	PRESENT	PRESENT	PRESENT
ASSYST121	PRESENT	PRESENT	PRESENT
ASSYST122	PRESENT	PRESENT	PRESENT
ASSYST123	PRESENT	PRESENT	PRESENT
ASSYST124	PRESENT	PRESENT	PRESENT
ASSYST125	PRESENT	PRESENT	PRESENT
ASSYST126	PRESENT	PRESENT	PRESENT
ASSYST127	PRESENT	PRESENT	PRESENT
ASSYST128	PRESENT	PRESENT	PRESENT
ASSYST129	PRESENT	PRESENT	PRESENT
ASSYST130	PRESENT	PRESENT	PRESENT
ASSYST131	PRESENT	PRESENT	PRESENT
ASSYST132	PRESENT	PRESENT	PRESENT
ASSYST133	PRESENT	PRESENT	PRESENT
ASSYST134	PRESENT	PRESENT	PRESENT
ASSYST135	PRESENT	PRESENT	PRESENT
ASSYST136	PRESENT	PRESENT	PRESENT
ASSYST137	PRESENT	PRESENT	PRESENT
ASSYST138	LOST	LOST	LOST
ASSYST139	PRESENT	PRESENT	PRESENT
ASSYST140	PRESENT	PRESENT	PRESENT

ASSYST141	PRESENT	PRESENT	PRESENT
ASSYST142	PRESENT	PRESENT	PRESENT
ASSYST143	PRESENT	PRESENT	PRESENT
ASSYST144	PRESENT	PRESENT	PRESENT
ASSYST145	PRESENT	PRESENT	PRESENT
ASSYST146	PRESENT	PRESENT	PRESENT
ASSYST147	PRESENT	PRESENT	PRESENT
ASSYST149	PRESENT	PRESENT	PRESENT
ASSYST150	PRESENT	PRESENT	PRESENT
ASSYST151	PRESENT	PRESENT	PRESENT
ASSYST153	PRESENT	PRESENT	PRESENT
ASSYST154	PRESENT	PRESENT	PRESENT
ASSYST155	PRESENT	PRESENT	PRESENT
ASSYST156	PRESENT	PRESENT	PRESENT
ASSYST157	PRESENT	PRESENT	PRESENT
ASSYST158	PRESENT	PRESENT	PRESENT
ASSYST159	PRESENT	PRESENT	PRESENT
ASSYST160	PRESENT	PRESENT	PRESENT
ASSYST162	PRESENT	PRESENT	PRESENT
ASSYST164	PRESENT	PRESENT	PRESENT
ASSYST165	PRESENT	PRESENT	PRESENT
ASSYST166	PRESENT	PRESENT	PRESENT
ASSYST167	PRESENT	PRESENT	PRESENT
ASSYST170	PRESENT	PRESENT	PRESENT
ASSYST171	PRESENT	PRESENT	PRESENT
ASSYST172	PRESENT	PRESENT	PRESENT
ASSYST174	PRESENT	PRESENT	PRESENT
ASSYST175	PRESENT	PRESENT	PRESENT
ASSYST176	PRESENT	PRESENT	PRESENT
ASSYST178	PRESENT	PRESENT	PRESENT
ASSYST179	PRESENT	PRESENT	PRESENT
ASSYST180	PRESENT	PRESENT	PRESENT
ASSYST181	PRESENT	PRESENT	PRESENT
ASSYST183	PRESENT	PRESENT	PRESENT
ASSYST190	PRESENT	PRESENT	PRESENT

ASSYST200	PRESENT	PRESENT	PRESENT
ASSYST203	PRESENT	PRESENT	PRESENT
ASSYST206	PRESENT	PRESENT	PRESENT
ASSYST228	PRESENT	PRESENT	PRESENT
ASSYST229	PRESENT	PRESENT	PRESENT
ASSYST230	PRESENT	PRESENT	LOST
ASSYST232	PRESENT	PRESENT	PRESENT
ASSYST234	PRESENT	PRESENT	PRESENT
ASSYST236	PRESENT	PRESENT	PRESENT
ASSYST237	PRESENT	PRESENT	PRESENT
ASSYST238	PRESENT	PRESENT	PRESENT
ASSYST239	PRESENT	PRESENT	PRESENT
ASSYST240	PRESENT	PRESENT	PRESENT
ASSYST241	PRESENT	PRESENT	PRESENT
ASSYST242	PRESENT	PRESENT	PRESENT
ASSYST244	PRESENT	PRESENT	PRESENT
ASSYST251	PRESENT	PRESENT	PRESENT
ASSYST252	PRESENT	PRESENT	PRESENT
ASSYST253	PRESENT	PRESENT	PRESENT
ASSYST254	PRESENT	PRESENT	PRESENT
ASSYST256	PRESENT	PRESENT	PRESENT
ASSYST257	PRESENT	PRESENT	PRESENT
ASSYST258	PRESENT	PRESENT	PRESENT
ASSYST259	PRESENT	PRESENT	PRESENT
ASSYST260	PRESENT	PRESENT	PRESENT
ASSYST261	PRESENT	PRESENT	PRESENT
ASSYST263	PRESENT	PRESENT	PRESENT
ASSYST264	PRESENT	PRESENT	PRESENT
ASSYST265	PRESENT	PRESENT	PRESENT
ASSYST267	PRESENT	PRESENT	PRESENT
ASSYST268	PRESENT	PRESENT	PRESENT
ASSYST270	PRESENT	PRESENT	PRESENT
ASSYST271	PRESENT	PRESENT	PRESENT
ASSYST272	PRESENT	PRESENT	PRESENT
ASSYST273	PRESENT	PRESENT	PRESENT

ASSYST274	PRESENT	PRESENT	PRESENT
ASSYST275	PRESENT	PRESENT	PRESENT
ASSYST276	PRESENT	PRESENT	PRESENT
ASSYST277	PRESENT	PRESENT	PRESENT
ASSYST278	PRESENT	PRESENT	PRESENT
ASSYST280	PRESENT	PRESENT	PRESENT
ASSYST281	PRESENT	PRESENT	PRESENT
ASSYST282	PRESENT	PRESENT	PRESENT
ASSYST283	PRESENT	PRESENT	PRESENT
ASSYST284	PRESENT	PRESENT	PRESENT
ASSYST285	PRESENT	PRESENT	PRESENT
ASSYST286	PRESENT	PRESENT	PRESENT
ASSYST287	PRESENT	PRESENT	PRESENT
ASSYST289	PRESENT	PRESENT	PRESENT
ASSYST290	PRESENT	PRESENT	PRESENT
ASSYST299	PRESENT	PRESENT	PRESENT
ASSYST300	PRESENT	PRESENT	PRESENT
ASSYST302	PRESENT	PRESENT	PRESENT
ASSYST305	PRESENT	PRESENT	PRESENT
ASSYST306	PRESENT	PRESENT	PRESENT
ASSYST307	PRESENT	PRESENT	PRESENT
ASSYST309	PRESENT	PRESENT	PRESENT
ASSYST311	PRESENT	PRESENT	PRESENT
ASSYST312	PRESENT	PRESENT	PRESENT
ASSYST313	PRESENT	PRESENT	PRESENT
ASSYST314	PRESENT	PRESENT	PRESENT
ASSYST315	PRESENT	PRESENT	PRESENT
ASSYST316	LOST	PRESENT	PRESENT
ASSYST317	PRESENT	PRESENT	PRESENT
ASSYST318	PRESENT	PRESENT	PRESENT
ASSYST320	PRESENT	PRESENT	PRESENT
ASSYST321	PRESENT	PRESENT	LOST
ASSYST322	PRESENT	PRESENT	PRESENT
ASSYST323	PRESENT	PRESENT	PRESENT
ASSYST325	PRESENT	PRESENT	PRESENT

ASSYST327	PRESENT	PRESENT	PRESENT
ASSYST328	PRESENT	PRESENT	PRESENT
ASSYST329	PRESENT	PRESENT	PRESENT
ASSYST330	PRESENT	PRESENT	PRESENT
ASSYST333	PRESENT	PRESENT	PRESENT
ASSYST334	PRESENT	PRESENT	PRESENT
ASSYST335	PRESENT	PRESENT	PRESENT
ASSYST339	PRESENT	PRESENT	PRESENT
ASSYST340	PRESENT	PRESENT	PRESENT
ASSYST341	PRESENT	PRESENT	PRESENT
ASSYST342	PRESENT	PRESENT	PRESENT
ASSYST344	PRESENT	PRESENT	PRESENT
ASSYST345	PRESENT	PRESENT	PRESENT
ASSYST346	PRESENT	PRESENT	PRESENT
ASSYST347	PRESENT	PRESENT	PRESENT
ASSYST348	PRESENT	PRESENT	PRESENT
ASSYST349	PRESENT	PRESENT	PRESENT
ASSYST350	PRESENT	PRESENT	PRESENT
ASSYST352	PRESENT	PRESENT	PRESENT
ASSYST354	PRESENT	PRESENT	PRESENT
ASSYST355	PRESENT	PRESENT	PRESENT
ASSYST357	PRESENT	PRESENT	PRESENT
ASSYST359	PRESENT	PRESENT	PRESENT
ASSYST360	PRESENT	PRESENT	PRESENT
ASSYST361	PRESENT	PRESENT	PRESENT
ASSYST362	PRESENT	PRESENT	PRESENT
ASSYST363	PRESENT	PRESENT	LOST
ASSYST364	PRESENT	PRESENT	PRESENT
ASSYST365	PRESENT	PRESENT	LOST
ASSYST366	PRESENT	PRESENT	PRESENT
ASSYST367	PRESENT	PRESENT	PRESENT
ASSYST368	PRESENT	PRESENT	PRESENT
ASSYST369	PRESENT	PRESENT	PRESENT
ASSYST371	PRESENT	PRESENT	PRESENT
ASSYST372	LOST	LOST	PRESENT

ASSYST373	PRESENT	PRESENT	PRESENT
ASSYST374	PRESENT	PRESENT	PRESENT
ASSYST377	PRESENT	PRESENT	PRESENT
ASSYST378	PRESENT	PRESENT	PRESENT
ASSYST379	PRESENT	PRESENT	PRESENT
ASSYST380	PRESENT	PRESENT	PRESENT
ASSYST381	PRESENT	PRESENT	PRESENT
ASSYST382	PRESENT	PRESENT	PRESENT
ASSYST383	PRESENT	PRESENT	PRESENT
ASSYST384	LOST	LOST	PRESENT
ASSYST385	PRESENT	PRESENT	PRESENT
ASSYST386	PRESENT	PRESENT	PRESENT
ASSYST389	PRESENT	PRESENT	PRESENT
ASSYST390	PRESENT	PRESENT	PRESENT
ASSYST391	PRESENT	PRESENT	PRESENT
ASSYST392	PRESENT	PRESENT	PRESENT
ASSYST393	PRESENT	PRESENT	PRESENT
ASSYST394	PRESENT	PRESENT	PRESENT
ASSYST397	PRESENT	PRESENT	PRESENT
ASSYST398	PRESENT	PRESENT	PRESENT
ASSYST399	PRESENT	PRESENT	PRESENT
ASSYST446	LOST	LOST	LOST
